# Biocatalytic Alkylation of Ambident Nucleophiles Enables Selective *N*‐Functionalization of Heterocycles and Late‐Stage Modifications

**DOI:** 10.1002/anie.202510300

**Published:** 2025-07-17

**Authors:** Felipe Ospina, Kai H. Schülke, Marius Schnutenhaus, Alina Klein, Om Desai, Shubhanshu Jain, Christine Krofta, Lukas Stratmann, Jianing Yang, Harald Gröger, Stephan C. Hammer

**Affiliations:** ^1^ Research Group for Organic Chemistry and Biocatalysis Faculty of Chemistry Bielefeld University Universitätsstraße 25 33615 Bielefeld Germany; ^2^ Chair of Industrial Organic Chemistry and Biotechnology Faculty of Chemistry Bielefeld University Universitätsstraße 25 33615 Bielefeld Germany

**Keywords:** Alkylation, Biocatalysis, Heterocycles, Enzymes, Synthetic methods

## Abstract

The alkylation with electrophilic haloalkanes is a key methodology in chemical synthesis to build desired molecules. Although alkylation of compounds bearing a single nucleophilic site is routine, the selective alkylation of polyfunctional molecules with multiple competing nucleophilic positions of comparable reactivity is often very challenging. In this work, we report a generalizable solution for selective alkylation chemistry that combines the selectivity of enzyme catalysis with the reactivity of off‐the‐shelf alkylation reagents. We employ engineered transferases in a modular cyclic cascade and use functionalized *N*‐heteroarenes as challenging proof‐of‐concept substrates. This catalytic alkylation approach is mild, highly chemo‐ and regioselective, proceeds on gram‐scale, provides rapid access to important *N*‐alkylated heterocyclic building blocks and enables challenging late‐stage alkylations. This study demonstrates a generalizable strategy to streamline synthetic routes to many pharmaceutically important compounds by selective biocatalytic alkylation of polyfunctional molecules and ambident nucleophiles.

## Graphical abstract

*N*‐alkylated heteroarenes are key structural motifs in bioactive compounds, but their regioselective synthesis via coupling of readily available azoles with haloalkanes remains very challenging. Here, we present a mild biocatalytic approach that proceeds on gram‐scale, is highly chemo‐ and regioselective, offering rapid access to valuable *N*‐alkylated building blocks and enabling demanding late‐stage alkylations.

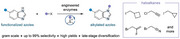

## Introduction

The nucleophilic substitution with off‐the‐shelf alkylation reagents is a key method in synthesis. Although essential for molecules with a single nucleophilic site, such alkylations frequently suffer from low selectivity in polyfunctional compounds, where competing nucleophiles of similar reactivity lead to product mixtures and low yields. This issue is particularly prominent in polyols, polyamines, peptides, or *N*‐heterocycles due to multiple reactive sites that complicate the control of selectivity and lead to unpredictable reaction outcomes. Similar to steroid synthesis, where selective enzymatic late‐stage oxidations have enabled the production of key steroidal intermediates for a billion‐dollar market,^[^ ^1^ ^]^ selective late‐stage alkylations of polyfunctional molecules hold significant potential. These transformations could radically streamline synthetic routes to many valuable compounds.

**Figure 1:**
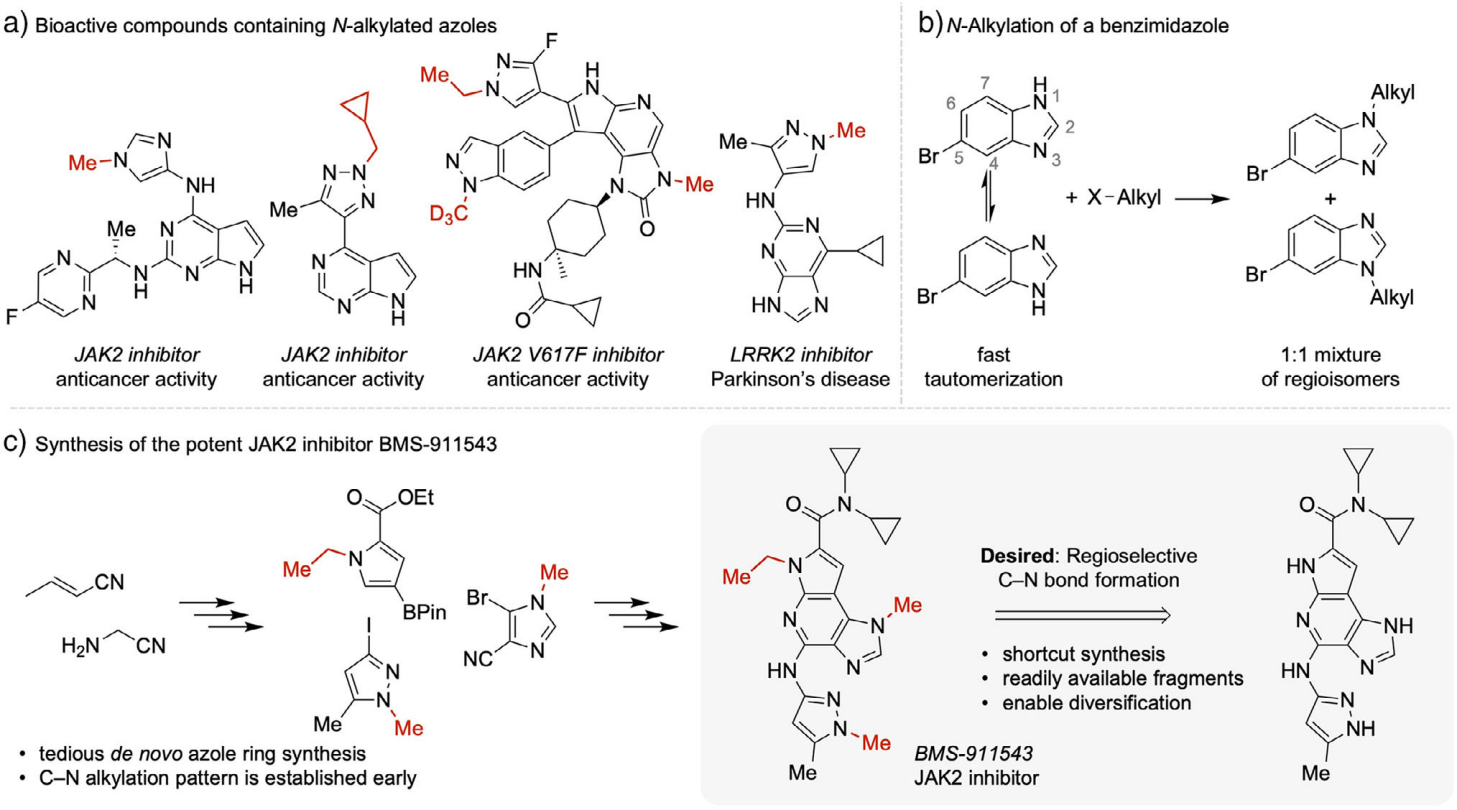
Importance and synthesis of *N*‐alkylated heteroarenes. a) *N*‐alkylated heteroarenes are paramount motifs in bioactive compounds such as pharmaceuticals.^[^ ^16^, ^17^, ^18^, ^19^ ^]^ b) Selective synthesis of *N*‐alkylated heteroarenes from readily available starting materials is frequently hampered by annular tautomerism generating constitutional isomers with similar reactivity. c) Summarized synthetic route for the synthesis of an *N*‐alkylated heteroarene drug (BMS‐911543).^[^ ^20^ ^]^ Synthesis typically depends on tedious *de novo* ring construction with the *N*‐alkylation pattern being set early in synthesis. In contrast, C─N bond formation through direct regioselective *N*‐alkylation could potentially shortcut synthesis and enable efficient diversification of lead structures on a late stage.

*N*‐Alkylated heteroarenes, including imidazoles, purines, pyrazoles and triazoles, are essential structural motifs in agrochemicals and pharmaceuticals (Figures 1a and S1).^[^ ^2^, ^3^ ^]^ Heteroarenes are found in more than 20% of FDA‐approved drugs.^[^ ^4^ ^]^ Because *N*‐methylation and *N*‐alkylation patterns of these molecules critically determine bioactivity (Figure S2),^[^ ^5^, ^6^, ^7^ ^]^ efficient synthetic access to specific regioisomers is utmost importance. Ideally, low‐cost *N*‐heteroarenes and readily available electrophilic alkylation reagents could be used to form desired electrophile–nucleophile bonds with high selectively. However, due to annular tautomerism, *N*‐heteroarenes possessing *N*‐atoms of ambident reactivity (Figures 1b and S3) typically produce mixtures of regioisomers that are difficult to separate. Currently, there is no generalizable catalytic method, reagent, or protecting group strategy to control regioselectivity in such C─N bond forming reactions (Figure S4). Despite significant progress, regioselectivity remains difficult to predict and is typically dictated by substrate‐specific steric and electronic effects.^[^ ^8^, ^9^, ^10^, ^11^ ^]^ The lack of general regioselective methods often necessitates tedious *de novo* ring synthesis to access the desired *N*‐alkylated motifs (Figure S5). This results in lengthy synthetic routes that are major hurdles in the diversification of lead structures to develop more bioactive derivatives (Figures 1c and S6). A general catalytic approach that can selectively differentiate between such ambident *N*‐atoms remains largely elusive at the building‐block level, is highly desirable for late‐stage functionalization and on the wish list of many chemists.^[^ ^12^, ^13^, ^14^, ^15^ ^]^

In this work, we report a generalizable synthetic approach harnessing engineered enzymes for selective alkylation of polyfunctional molecules containing multiple competing nucleophilic sites. As a proof of concept, we demonstrate the selective *N*‐alkylation of functionalized heteroarenes using readily available reagents. These alkylation reactions exhibit excellent chemo‐ and regioselectivity, tolerate a wide range of functional groups, are applicable on gram scale, and enable orthogonal modification of multiple heteroatoms with similar reactivity in a late‐stage setting.

## Results and Discussion

### Enabling Preparative Scale Methylation of Functionalized Azoles

**Figure 2:**
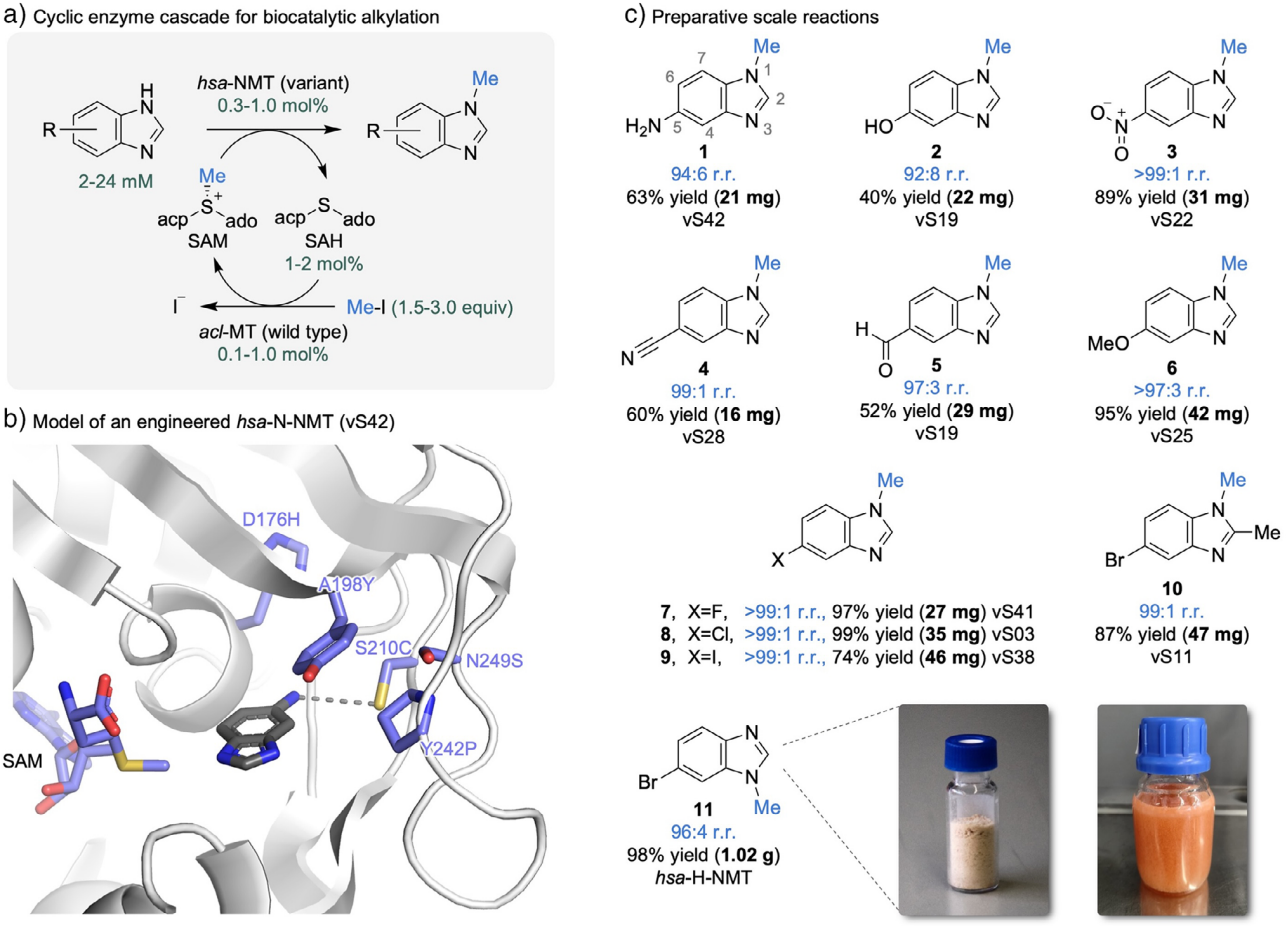
Regioselective *N*‐methylation of functionalized benzimidazoles on a preparative scale. a) Cyclic two‐enzyme cascade of two SAM‐dependent transferases for the selective methylation of 5‐substituted benzimidazoles. acp = (*S*)‐3‐amino‐3‐carboxypropyl. ado = adenosyl. b) Modelled interactions of vS42 (*hsa*‐N‐NMT D176H, A198Y, S210C, Y242P, and N249S) with 5‐aminobenzimidazole using Chai‐1.^[^ ^28^ ^]^ The model of the active site reveals how the engineered transferase could control selectivity. c) Preparative‐scale reactions. Typical reaction conditions: 2 mM substrate, 3 equiv MeI, 1 mol% *hsa*‐NMT (variant), 0.1 mol% *acl*‐MT wild‐type, and 1 mol% SAH. Gram‐scale reaction condition: 24 mM substrate, 1.5 equiv MeI, 0.3 mol% *hsa*‐H‐NMT, 0.2 mol% *acl*‐MT wild‐type, and 2 mol% SAH (see Supporting Information for details).

We have recently started to explore the use of engineered enzymes for regioselective and regiodivergent *N*‐methylation of azole building blocks, including pyrazoles, imidazoles, and triazoles.^[^ ^21^, ^22^ ^]^ This biocatalytic approach utilizes a cyclic enzyme cascade that employs *S*‐adenosyl‐l‐homocysteine (SAH) as a methyl transfer cocatalyst (Figure 2a).^[^ ^23^ ^]^ The biocatalytic system integrates two distinct transferases: one catalyzing the chemo‐ and stereoselective methylation of SAH to (re)generate *S*‐adenosyl‐l‐methionine (SAM), and another utilizing SAM as an electrophilic sulfonium ion cosubstrate in the enzyme‐controlled methylation reaction. Although SAM‐dependent transferases have been studied for decades, their application in preparative organic synthesis remains underdeveloped and is mainly limited to analytical scale.^[^ ^24^, ^25^, ^26^ ^]^ While we identified an efficient, promiscuous transferase from *Aspergillus clavatus* (*acl*‐MT) for SAM recycling (Figure 2a),^[^ ^21^, ^27^ ^]^ the regioselective methylation of non‐natural azole substrates required enzyme engineering.^[^ ^21^, ^22^ ^]^ This first generation of engineered azole *N*‐methyltransferases (MTs) exhibited high selectivity, but their low activity limited this approach to nonfunctionalized azoles and analytical‐scale reactions. To address the synthetic problem of selective alkylation, we first explored the potential of transferases for preparative chemistry using 5‐substituted benzimidazoles as proof‐of‐concept substrates. The two ambident *N*‐atoms in such azoles are only barely differentiated by steric and electronic effects from the distal substitutions, making such heteroarenes particularly difficult targets for regioselective *N*‐methylation and *N*‐alkylation.

We first set out to engineer a panel of transferases with improved activity to enable preparative‐scale reactions, even on functionalized azoles bearing additional reactive centers. We used a previously engineered transferase as a starting point for enzyme engineering, namely the *homo sapiens* nicotinamide *N*‐methyltransferase (*hsa*‐N‐NMT) that contained five active‐site mutations (v31 = *hsa*‐N‐NMT D167H, A198M, S201C, Y242F, and N249S). To generate a diverse panel of catalysts for the conversion of 5‐substituted benzimidazoles, we constructed a combinatorial mutant library incorporating various activating mutations identified through site‐saturation mutagenesis at twelve active‐site residues (Y20, Y24, L164, H167, D197, M198, C201, Y204, S213, F242, A247, and S249). The designed combinatorial library consisted of 432 individual variants and was bought as synthetic DNA spread‐out low diversity (SOLD) library from Twist Bioscience (Figure S7). After screening using 5‐bromobenzimidazole as substrate, we selected the best‐performing variants resulting in a final mutant library of 46 active transferases (vS01–vS46). This second generation of azole NMTs contained five to eight active‐site mutations compared to the *hsa*‐N‐NMT wild type (Table S1).

The generated enzyme panel is highly regioselective and efficient in the *N*‐methylation of 5‐substituted benzimidazoles (Figures 2 and S8), enabling preparative‐scale synthesis with a broad functional group tolerance. Many derivatives (**1**–**10**) have been synthesized with good yields of isolated product (up to 99%) and very high regioselectivity (up to >99:1 r.r.). This includes for example benzimidazoles (**1** and **2**) with unprotected amino and hydroxy moieties, that typically require protecting group strategies during the complex *de novo* synthesis. Further, nitro (**3**), cyano (**4**), and aldehyde (**5**) groups at the 5‐position as well as a substitution at the 2‐position (**10**) were tolerated. Although all reactions were conducted on a preparative milligram level, we simultaneously developed a protocol for gram‐scale synthesis. Key steps in developing a practical process were increasing substrate loading by more than tenfold (24 mM compared to originally 2 mM) and utilizing efficient transferases with low catalyst loading (≤0.3 mol%). The gram‐scale reaction was carried out with *homo sapiens* histamine NMT (*hsa*‐H‐NMT), which produces the opposite regioisomer compared to the 2nd generation azole *N*‐methyltransferases. Using 1.5 equiv iodomethane, the building block **11** was synthesized with high isolated yield (98%) and regioselectivity (96:4 r.r.) (Figure 2c). These improvements not only provide simple access to important building blocks from cheap starting materials but also prove that selective biocatalytic methylation chemistry can be performed beyond the milligram scale.^[^ ^29^, ^30^, ^31^, ^32^, ^33^, ^34^ ^]^

### Orthogonal Late‐Stage Methylation

**Figure 3:**
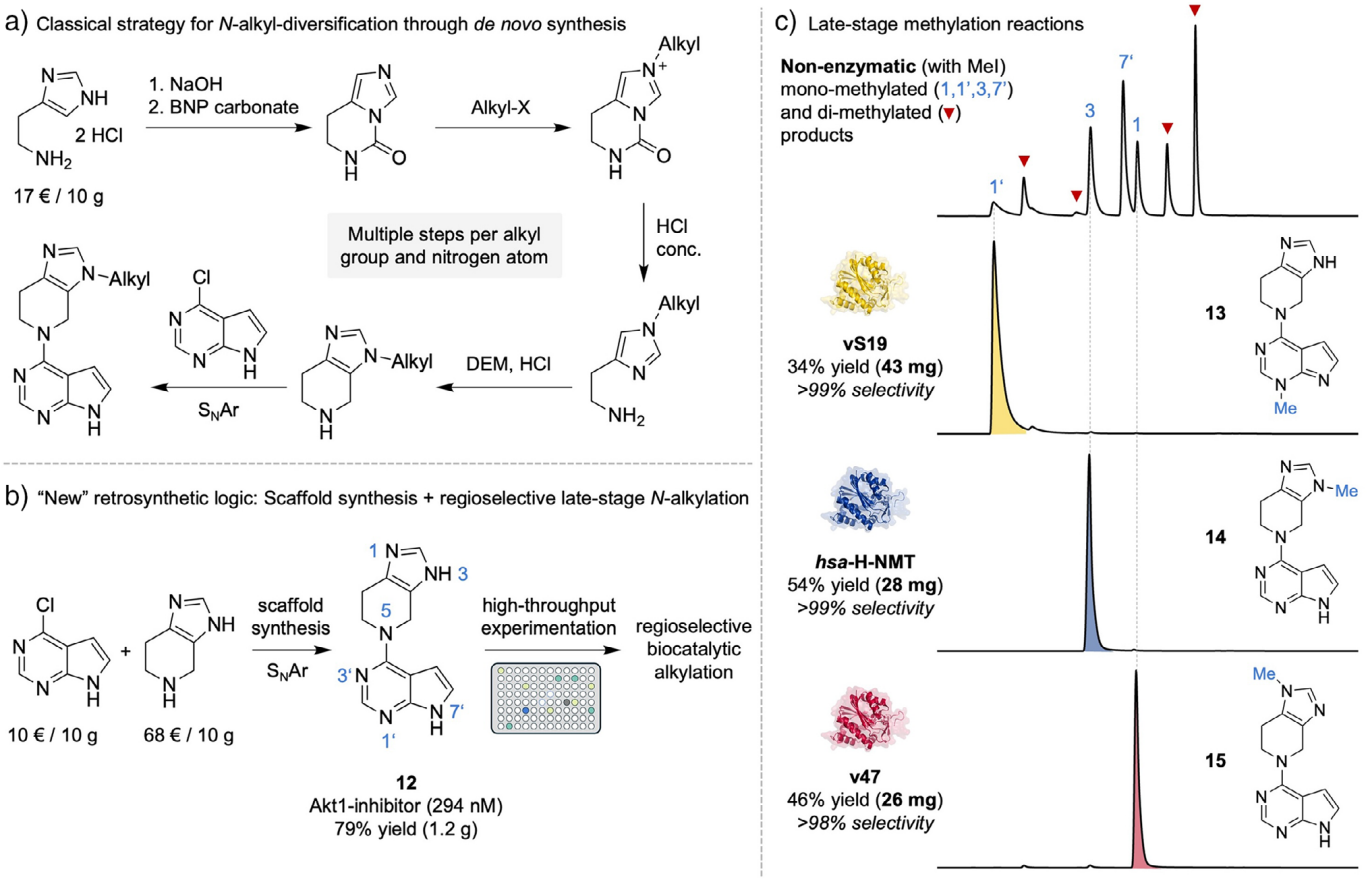
Late‐stage diversification of an Akt1‐kinase inhibitor **12** ^[^ ^35^ ^]^ through regioselective methylation. a) Envisioned synthetic sequence for alkyl‐diversification at the *N*3‐position.^[^ ^36^, ^37^, ^38^ ^]^ Typically, such C─N bonds are set early in synthesis, often through complex *de novo* ring construction with each *N*‐atom depending on a different synthetic route.^[^ ^13^ ^]^ b) Proposed new retrosynthetic logic that builds on regioselective biocatalytic alkylation in combination with scaffold synthesis from readily available *N*‐heteroarene building blocks. c) Nonenzymatic methylation of **12** generates a mixture mono‐ and di‐methylated products in comparison to highly regioselective and regiodivergent enzymatic methylation. The figure compares LC/MS chromatograms resulting from nonenzymatic and enzymatic alkylation reactions. BNP carbonate: bis(4‐nitrophenyl) carbonate. DEM: diethoxymethane.

Having established regioselective *N*‐methylation of functionalized benzimidazoles on a preparative scale, we next aimed to apply this approach on a drug‐like molecule. To explore enzymatic late‐stage diversification and avoid complex *de novo* ring synthesis, we selected compound **12** from a recent medicinal chemistry campaign. This Pfizer‐investigated anticancer agent inhibits the serine/threonine kinase Akt‐1 with high potency in the nanomolar range.^[^ ^35^ ^]^ The pharmacologically active entity **12** serves as an ideal test case to explore biocatalytic late‐stage methylation, as it consists of two competing *N*‐heterocycles, a pyrrolopyrimidine and an imidazopiperidine, overall featuring six potential nucleophilic *N*‐methylation sites. While *N*‐alkyl‐diversification of such heteroarene‐rich compounds typically requires multistep synthetic routes (Figure 3a), direct alkylation would offer a more efficient approach and streamlined way for preparation. We synthesized **12** from readily available starting materials in a single step (Figure 3b). Subsequent application of a conventional chemical methylation protocol (nonenzymatic) with iodomethane as reagent yielded multiple mono‐ and di‐methylated products, reflecting the molecule's multident reactivity (Figure 3c, top).

For enzymatic late‐stage methylation, we utilized a panel of 146 methyltransferases (MTs), produced in two 96‐deep well plates. This enzyme library included 96 mutants of *hsa*‐N‐NMT (vS01‐vS46 and 50 designs from an earlier engineering campaign^[^ ^21^ ^]^) along with 50 wild‐type MTs sourced from a natural MT pool (Table S2). We screened for selective methylation using a simple liquid chromatography setup coupled to mass spectrometry detection (LC/MS). Screening identified various active enzymes for this bulky substrate enabling highly selective late‐stage methylation at the *N*1‐, *N*1’‐, and *N*3‐positions (**13**–**15**, see Figure 3c). Notably, the orthogonal derivatization reactions were performed on a milligram scale with useful isolated yields and excellent regioselectivity (>98%). Next to modifications at the imidazole unit, selective C─N bond formation was also achieved on the pyrimidine ring of the bicyclic pyrrolopyrimidine heterocycle (**13**). Taken together, this provides the first proof‐of‐concept for the sought‐after^[^ ^13^ ^]^ regioselective alkylation of multident azole‐type nucleophiles. Moreover, it unlocks a new retrosynthetic logic to access complex *N*‐alkylated heteroarenes by combining “scaffold” synthesis from readily available azoles with selective biocatalytic late‐stage alkylation (Figure 3b).

### Regioselective Modification with an Expanded Alkyl Diversity

With a method for regioselective methylation in hand, we next aimed to demonstrate the generality of this approach by broadening the haloalkane scope. Although conceptually simple, this relies on transferases that efficiently utilize haloalkanes for SAM analog synthesis and enzymes that accept these analogs for selective C─N bond formation (Figure 2a). Biocatalysis with SAM‐dependent transferases is recently flourishing,^[^ ^23^, ^31^, ^34^, ^39^, ^40^, ^41^, ^42^, ^43^, ^44^, ^45^ ^]^ however, practical alkylations using simple off‐the‐shelf reagents are underdeveloped (Figure S9).^[^ ^21^, ^22^, ^33^, ^39^ ^]^

**Figure 4:**
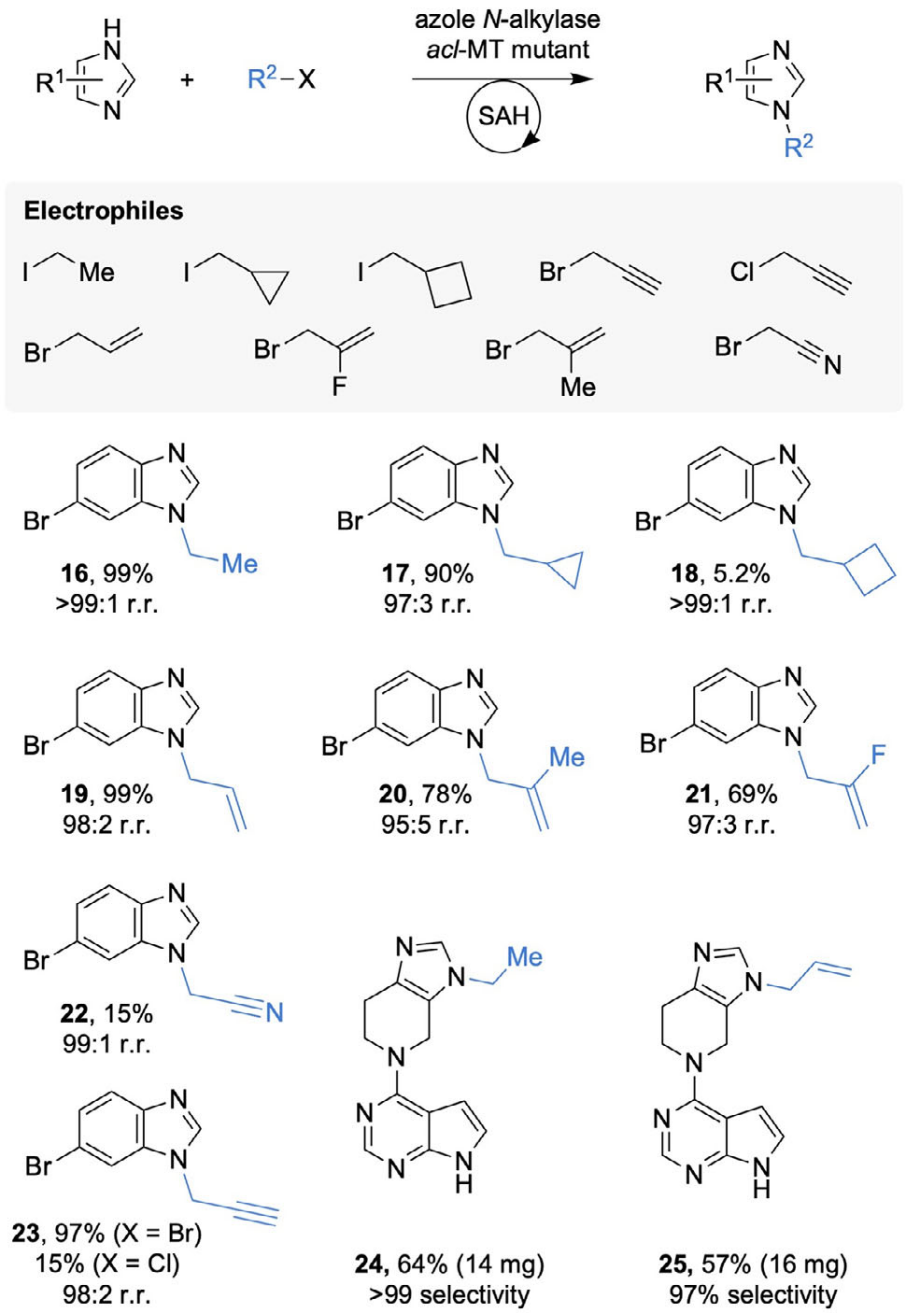
Regioselective alkylation including allylation and propargylation of azoles. The reactions are reported as quantified HPLC yield (**16**–**23**) and isolated yield (**24** and **25**). Typical reaction conditions: 2 mM azole, 3 equiv haloalkane, 1 mol% of both transferases, and 1 or 10 mol% SAH (see Supporting Information for details).

To build a regioselective azole alkylation platform, we aimed to combine engineered enzymes for SAM analog synthesis with biocatalysts that promote selective C─N bond formation. Having a diverse selection of readily available electrophiles in mind (Figure 4), we first set out to find NMTs capable of selective azole *N*‐alkylation with a “bulkier” alkyl group. To this end, we screened the transferase library of 146 enzymes (see above) using (iodomethyl)cyclopropane as “bulky” alkylating reagent and 5‐bromobenzimidazole as a challenging ambident nucleophile. We identified a histamine NMT from zebrafish (*Danio rerio*, *dre*‐H‐NMT) that exhibited initial alkylation activity. Through two rounds of directed evolution, we engineered *dre*‐H‐NMT into an efficient and selective azole *N*‐alkylase (*dre*‐H‐NMT Y15A, C198Y) (Figure S10). Next, we turned our attention to the diverse set of electrophiles and identified efficient *acl*‐MT mutants using enzymes from a recent engineering campaign.^[^ ^46^ ^]^ We focused on promiscuous *acl*‐MT mutants (*acl*‐MT v02, v15, v16, v31, v55, and v63), each containing two to four active site mutations (Table S3). Careful analysis revealed that these six *acl*‐MT mutants were sufficient to utilize the diverse selection of electrophiles, regardless of the electrophile's nature (Figures 4 and S11).

The synergistic use of both engineered transferases (azole *N*‐alkylase and an *acl*‐MT mutant) in a cyclic enzyme cascade enabled highly regioselective (>99% r.r.) and efficient (up to 99% yield) *N*‐alkylation with various electrophiles (Figures 4 and S12). Please note that the non‐enzymatic alkylations produce a 1:1 mixture of regioisomers (Figure S12), highlighting the advantage and selectivity of the enzymatic approach. The engineered enzymes enabled regioselective alkylation with both linear and cyclic alkyl groups (**16**–**18**) as well as allylation (**19**, **20**), including fluoro‐allylation to generate a monofluoroalkene (**21**). In addition to alkylation and allylation, we explored broader azole *N*‐modification with functionalized electrophiles to enable straightforward downstream transformations. For example, 2‐bromoacetonitrile was used to install a reactive nitrile group (**22**), potentially facilitating the formation of carboxylic acids, amides, or amines in subsequent reactions. Additionally, 3‐bromo‐1‐propyne enabled efficient and highly selective *N*‐propargylation (**23**). This successful transformation is unexpected, as propargyl‐SAM analogs are unstable and undergo rapid decomposition at physiological pH. To overcome this limitation, more stable selenium‐based cosubstrate analogs have been developed in the past.^[^ ^47^, ^48^ ^]^ The successful *N*‐propargylation reported here is likely attributed to the efficiency of the azole *N*‐alkylase in converting the reactive propargyl sulfonium ion before it can decompose.

Finally, we applied this approach beyond building blocks and targeted selective late‐stage alkylation of compound **12**. These reactions proceeded with very high selectivity on preparative scale (**24** and **25**), suggesting that this method could readily provide dozens of structural analogs of lead compounds. Importantly, this study revealed that, in addition to iodoalkanes, even weaker and more economically attractive electrophiles, such as bromo‐ and chloroalkanes, were successfully converted (Figure 4). This underscores the general applicability of this approach with off‐the‐shelf reagents and suggests many new avenues for reaction development with engineered and tailor‐made enzymes.

## Conclusion

We report an efficient solution addressing a long‐standing challenge in synthetic chemistry, namely the regioselective *N*‐alkylation and *N*‐functionalization of ambident heterocycles. The developed biocatalytic system combines the excellent selectivity of enzyme catalysis with the high reactivity of non‐natural haloalkane reagents. This opens the door to shortcut synthetic routes to many important molecules by providing a new retrosynthetic logic that can evade tedious *de novo* azole construction and build on thousands of readily available *N*‐heterocycles and haloalkanes. This study points toward a potent methodology that can accelerate diversification of lead structures in medicinal chemistry and speed up the iterative design of new drug candidates while minimizing waste, synthetic steps, and protecting group applications.

Although our study focused on regioselective alkylation of selected but challenging azoles, we anticipate that this approach is broadly applicable. Using the enzyme engineering strategies reported here, diverse biocatalysts can be developed to enable selective alkylation, not only of 1,3‐azoles but of a wide range of *N*‐heteroarenes, including pyrazoles, triazoles, tetrazoles, purines, pyridones, or imidazolones. As SAM‐dependent transferases are a versatile enzyme class,^[^ ^25^, ^26^, ^40^, ^49^, ^50^, ^51^, ^52^, ^53^, ^54^, ^55^ ^]^ this reported progress in synthesis can potentially be extended to a broad range of C─C, C─N, and C─O bond‐forming reactions. Particularly compelling are applications toward ambident nucleophiles and polyfunctional molecules, where conventional alkylation methods struggle to achieve high selectivity.

## Supporting Information

The authors have cited additional references within the Supporting Information.^[^ ^56^, ^57^, ^58^, ^59^, ^60^, ^61^, ^62^, ^63^, ^64^, ^65^, ^66^, ^67^, ^68^, ^69^, ^70^, ^71^, ^72^, ^73^, ^74^, ^75^, ^76^, ^77^, ^78^, ^79^, ^80^, ^81^, ^82^ ^]^

## Author Contributions

S.C.H., H.G., F.O., and K.H.S designed the research. F.O. and K.H.S. were the primary researchers in the laboratory responsible for conducting the vast majority of the experiments, as well as leading data analysis and interpretation. F.O. was the main responsible person for preparative scale as well as late‐stage methylation. K.H.S was the main responsible person for selective alkylation. M.S. contributed to build the second‐generation mutant library of azole *N*‐methyltransferases and cloned the library of 50 wildtype MTs. A.K. synthesized multiple product standards and helped with enzyme purification. O.M. helped with synthesizing product standards. S.J. helped with preparative scale enzyme reactions. C.K. and L.S. supported in HTS of mutant libraries. J.Y. and H.G. conceptualized and developed a protocol for gram‐scale synthesis. S.C.H., F.O., and K.H.S wrote the manuscript. All authors contributed to optimize the manuscript.

## Conflict of Interests

The authors declare no conflict of interest.

## Supporting information



Supporting Information

## Data Availability

The data that support the findings of this study are available in the Supporting Information of this article. Relevant data for reuse and primary raw data have been deposited in PUB (Universität Bielefeld) under https://doi.org/10.4119/unibi/3004864.
