# Supplementary material for: Biocatalytic Alkylation of Ambident Nucleophiles Enables Selective N‐Functionalization of Heterocycles and Late‐Stage Modifications
Source: Angew Chem Int Ed Engl. 2025 Jul 17;64(36):e202510300. doi: 10.1002/anie.202510300 (PMC12402838; doi:10.1002/anie.202510300)
Supplement: Supplementary file 1 — Supporting Information [file ANIE-64-e202510300-s001.pdf]

Supplementary Materials for

# Biocatalytic alkylation of ambident nucleophiles enables selective *N*-functionalization of heterocycles and late-stage modifications

Felipe Ospina[a,§], Kai H. Schülke[a,§], Marius Schnutenhaus[a], Alina Klein[a], Om Desai[a], Shubhanshu Jain[a], Christine Krofta[a], Lukas Stratmann[a], Jianing Yang[b], Harald Gröger[b], Stephan C. Hammer[a*]

## Affiliations

[a] Research group for Organic Chemistry and Biocatalysis, Faculty of Chemistry, Bielefeld University, Universitätsstraße 25, 33615 Bielefeld (Germany)

[b] Chair of Industrial Organic Chemistry and Biotechnology, Faculty of Chemistry, Bielefeld University, Universitätsstraße 25, 33615 Bielefeld, Germany

[§] These authors contributed equally

* Email: stephan.hammer@uni-bielefeld.de

## Author contributions

S.C.H., H.G., F.O. and K.H.S designed the research. F.O. and K.H.S. were the primary researchers in the laboratory responsible for conducting the vast majority of the experiments, as well as leading data analysis and interpretation. F.O. was the main responsible person for preparative scale as well as late-stage methylation. K.H.S was the main responsible person for selective alkylation. M.S. contributed to build the second-generation mutant library of azole *N*-methyltransferases and cloned the library of 50 wildtype MTs. A.K. synthesized multiple product standards and helped with enzyme purification. O.M. helped with synthesizing product standards. S.J. helped with preparative scale enzyme reactions. C.K. and L.S. supported in HTS of mutant libraries. J.Y. and H.G. conceptualized and developed a protocol for gram-scale synthesis. S.C.H., F.O. and K.H.S wrote the manuscript. All authors contributed to optimize the manuscript.

**This PDF file includes:**


I. Materials and methods ........ 3

II. Supporting figures ........ 17

III. Supporting tables ........ 35

IV. Calibration curves ........ 51

V. Chemical synthesis of substrates and products standards ........ 54

VI. Enzymatic preparative scale reactions ........ 66

VII. NMR spectra ........ 81

VIII. DNA and amino acid sequences ........ 142

IX. References ........ 179

# I. Materials and methods

**A) Chemicals and enzymes:** All chemicals and solvents were purchased from commercial suppliers (Sigma Aldrich, abcr GmbH, Carl Roth, TCI, BLDpharm, Chiramer Inc, and Enamine) and used without further purification. *S*-adenosyl-L-methionine (SAM) disulfate tosylate was purchased from abcr GmbH (cat-#: AB436584). *S*-adenosyl-L-homocysteine (SAH) was purchased from Chiramer, Inc (cat-#: CR-1273-CV). Lysozyme was purchased from Carl Roth (cat-#: 8259.2) and Pierce™ Universal Nuclease from Thermo Fisher Scientific Inc (cat-#: 88700). T5 exonuclease, DpnI, Phusion HF polymerase and *Taq* DNA ligase were purchased from New England Biolabs (NEB).

**B) NMR spectroscopy:** $^{1}H$ and $^{13}C$ NMR spectra were recorded on a Bruker Avance III 500 HD (at 500 MHz for $^{1}H$ and 126 MHz for $^{13}C$) and a Bruker Avance NEO 600 (at 600 MHz for $^{1}H$ and 151 MHz for $^{13}C$). Chemical shifts ($\delta$) are given in ppm relative to the residual signal pick of the solvent. Coupling constants ($J$) are given in Hz and the signal multiplicity is indicated using the following abbreviations: s = singlet, d = doublet, dd = doublet of doublets, dt = doublet of triplets, ddt = doublet of doublets of triplets, t = triplet, m = multiplet. Two-dimensional correlation measurements, including heteronuclear multiple quantum coherence (HMQC), heteronuclear multiple bond coherence (HMBC), homonuclear correlation spectroscopy (COSY), and nuclear overhauser effect spectroscopy (NOESY), confirmed the correct assignment of the signals.

**C) High resolution mass spectrometry (HRMS):** ESI accurate experiments were obtained using a Waters Synapt G2Si Q-TOF mass spectrometer (nano ESI and MALDI). Mass spectrometry parameters: positive ion polarity; nano ESI ion source type. HRMS data are reported as follows: HRMS (ESI) (m/z) calculated for $[M+H]^{+}$, found exact mass.

**D) Cloning and enzyme engineering of *hsa*-N-NMT and *dre*-H-NMT:** The employed *acl*-MTs (see **Table S3**) and FuncLib-derived *hsa*-N-NMT variants were already cloned in our group and reported in previous works.[1–3] pBAD33 ($Cm^{R}$) containing a C-terminal $His_6$-tag with LE-linker was used as the cloning and expression vector for all *acl*-MT and *dre*-H-NMT variants. These plasmids were transformed by electroporation into the SAHN-knockout *E. coli* strain JW0155 (Keio-collection).[4] Methylthioadenosine/SAH nucleosidase (MTAN) derived from *E. coli* was inserted into the pProEx vector ($Amp^{R}$) and transformed by electroporation into a BL21(DE3) strain.[5] Genes coding for the wild type MTs (see **Table S2**) and all *hsa*-N-NMT mutant variants (including variants derived from the FuncLib[1] and the combinatorial libraries) were inserted into a pET28a(+) vector ($Kan^{R}$) containing a C-terminal $His_6$-tag with LE-linker and transformed by electroporation into *E. coli* strain BL21(DE3). Genes of the wild type NMT enzyme panel were codon optimized and purchased from Twist Bioscience. The synthetic DNA fragments and the pET28a(+) backbone were amplified by PCR (see **Table S14** for the complete primers list) and DpnI digested. The resulting PCR products were separated through agarose gel electrophoresis and isolated using the Zymoclean™ Gel DNA Recovery Kit (Cat. No.: D4002, Zymo Research Corp.). Subsequently, a Gibson Assembly was performed to ligate the backbone with the fragments containing the genes of interest.[6] The resulting ligated products were purified using a DNA cleanup kit (DNA Clean & Concentrator™-5, Cat. No.: D4004, Zymo Research Corp).

Mutagenesis of *hsa*-N-NMT: Site-saturation mutagenesis (SSM) of v31 (*hsa*-N-NMT D167H, A198M, S201C, Y242F, N249S) was performed using the "22c-trick"[7] addressing a total of twelve active-site residues forming the substrate binding pocket (Y20, Y24, L164, H167, D197, M198, C201, Y204, S213, F242, A247, S249). PCR amplified products were *Dpn*I digested and purified by agarose gel electrophoresis using the Zymoclean™ Gel DNA Recovery Kit and ligated using Gibson Assembly.[6] The resulting products were purified using a DNA cleanup kit and transformed through electroporation into a 5'-methylthio-adenosine/*S*-adenosylhomocysteine nucleosidase (mtn) deficient *E. coli* BL21(DE3) strain. After screening (see section **H**) beneficial mutations at five positions were identified. Although positions A74 and A134 were not targeted, the A74G and A134V mutations emerged as polymerase-induced errors during PCR leading to the double mutants A34V/M198H and A74G/H167L (see **Figure S7A**).

Combinatorial mutant library: After the activity confirmation of the beneficial mutations (see section **H**), we decided to explore possible combinatorial effects by recombining the ten most beneficial mutations in a combinatorial mutant library (see **Figure S7B**). This library was purchased as a commercial Spread-Out Low Diversity (SOLD) DNA library from Twist Bioscience. The synthetic gene of the SOLD DNA library and the pET28a(+) backbone were amplified by PCR (see **Table S14** for the complete primer list), and DpnI digested. The amplified DNA products were purified by agarose gel electrophoresis using the Zymoclean™ Gel DNA Recovery Kit. The fragments were ligated using Gibson assembly,[6] and the resulting products were purified using a DNA cleanup kit. The combinatorial SOLD library was transformed through electroporation into an *E. coli* Bl21 (DE3) strain.

Mutagenesis of *dre*-H-NMT: For site-saturation mutagenesis, the gene of *dre*-H-NMT was cloned from pET28a(+) into the pBAD33 system to enable engineering under cascade conditions using the SAHN-knockout JW0155 *E. coli* strain. First shell positions of the active site were selected based on a 3D structure generated by AlphaFold3[8]. SAH and substrate binding sites were identified by comparing this generated structure with reported crystal structures of *hsa*-H-NMT wild type (Sequence identity: 43.8%, Sequence similarity: 66.4%, pdb: 1JQD, 1JQE, 2AOU, 2AOT)[9] and *hsa*-N-NMT wild type (Sequence identity: 13.9%, Sequence similarity: 25.9%, pdb: 2IIP). Site-saturation was performed using the "22c-trick" (see **Table S14** for the complete primer list).[7] PCR amplified products were DpnI digested and purified by agarose gel electrophoresis using the Zymoclean™ Gel DNA Recovery Kit and ligated using Gibson Assembly.[6] The resulting products were purified using a DNA cleanup kit and transformed through electroporation into the JW0155 *E. coli* Strain (Keio Collection).

**E) Protein expression in 96 deep-well plates (DWPs):**

Expression of *hsa*-N-NMT variants and NMT wild types: Either freshly transformed BL21 (DE3) cells (for screening) or glycerol stocks (for rescreening) containing *hsa*-N-NMT variants or NMT wild types were used to inoculate TB medium in 96-DWPs: 700 μL/well TB medium (24 g/L yeast extract, 12 g/L tryptone, 5 g/L glycerol, 89 mM KPi pH 7.4, 35 μg/mL kanamycin). DWPs were covered through a breathable sealing film (Europe Cat. No.: 731-0316, VWR) and incubated at 37°C and 250 rpm overnight. On the next morning, a sterile DWP was filled with 610 μL/well TB medium (see above), supplemented with 35 μg/mL kanamycin and inoculated with 50 μL of the DWPs precultures. DWPs were covered with a breathable sealing film and incubated for 4 h at 37°C and 250 rpm. Then, after cooling on an ice bath for 15 min, the DWPs were induced by adding 40 μL/well of TB medium supplemented with 3.5 mM IPTG (final concentration: 0.2 mM). Protein production was carried out at 20°C and 250 rpm over 20 h. The cells were harvested through centrifugation (4°C, 4300 rcf, 15 min) and stored at -20°C for at least one night before further use.

Expression of *dre*-H-NMT variants: Freshly transformed JW0155 cells containing *dre*-H-NMT variants (see section I, D) were used to inoculate TB medium in 96-DWPs: 700 μL/well TB medium (24 g/L yeast extract, 12 g/L tryptone, 5 g/L glycerol, 89 mM KPi pH 7.4, 34 μg/mL chloramphenicol). DWPs were covered through a breathable sealing film and incubated at 37°C and 250 rpm overnight. On the next morning, a sterile DWP was filled with 610 μL/well TB media (see above), supplemented with 34 μg/mL chloramphenicol and inoculated with 50 μL of the DWPs precultures. DWPs were covered with a breathable sealing film and incubated for 3 h at 37°C and 250 rpm before the DWPs were cooled in an ice bath for 15 min. For induction, 40 μL/well of TB medium supplemented with 0.35% (w/v) L-arabinose (final concentration: 0.02% w/v) was added. Protein production was carried out at 20°C and 250 rpm over 20 h. The cells were harvested through centrifugation (4°C, 4300 rcf, 15 min) and stored at -20°C for at least one night before further use. Each plate contained three wells w/o cells (growth controls) and wells three *dre*-H-NMT wild type as controls.

**F) Protein expression at 500 mL scale for NMT and *acl*-MT variants:** For the purification of NMTs and *acl*-MT variants, protein expression was carried out in 2 L culture flasks with baffles containing each 500 mL TB medium. Therefore, 5 mL LB (10 g/L tryptone, 5 g/L yeast extract, 5 g/L NaCl) cultures containing antibiotics (pET28a(+): 50 μg/mL Kanamycin, pBAD33: 34 μg/mL chloramphenicol) were inoculated from single colonies or from glycerol stocks (stored at -80°C) and incubated at 37°C and 180 rpm overnight. For the main cultures, 500 mL of TB medium (pET28a(+): 50 μg/mL kanamycin, pBAD33: 34 μg/mL chloramphenicol) was inoculated with the overnight cultures to a starting concentration of 1% (v/v). The main cultures were grown to an $OD_{600}$ value of 0.6–0.8 before being cooled on ice for 10 min. Gene expression was induced by adding either IPTG (pET28a(+) expression system) to a final concentration of 0.2 mM or L-arabinose (pBAD33 expression system) to a final concentration of 0.02% (w/v). Protein production was carried out at 20°C for 20 h at 100 rpm. Finally, the cells were harvested at 4300 rcf, 4°C for 15 min, frozen and stored at -20°C for at least one night.

**G) Protein purification and storage:** Frozen cells were thawed and resuspended in pre-cooled buffer A (composed of 50 mM KPi, 500 mM NaCl, 10 mM imidazole, 5% (v/v) glycerol, pH 7.5) at a ratio of 2-3 mL per 1 g of cell paste. The cell suspension was lysed through sonication (Bandlin Sonoplus HD 2070) employing a 5-minute-long 2 sec on-/off-cycle using 50% amplitude. Insoluble cell debris was separated by centrifugation (21000 rcf, 4°C, 10 min) and the cell-free extract was filtered through a 0.2 μm sterile filter. Enzyme purification was conducted at r.t. on a 1 mL Sepharose 6 column loaded with $Ni^{2+}$ (His GraviTrap, cytiva). The column was equilibrated with buffer A for 10 column volumes (CV) before the cell-free extract was loaded and washed with 10 CV of buffer A. Enzyme-containing fractions (4 CV) were eluted through a stepwise gradient using 20 mM, 40 mM, 70 mM, 100 mM and 500 mM imidazole. Protein containing fractions were identified through Pierce™ BCA Protein Assay Kit (Thermo Scientific, US), combined, concentrated by ultracentrifugation (Amicon® Ultra-4, 10 kDa Cut-off, Cat.No.: UFC801024, Sigma Aldrich), and dialyzed twice against storage buffer (ratio protein solution to buffer 1:100, 50 mM KPi buffer, 5% (v/v) glycerol, pH 7.5). Purity of the obtained protein fractions were verified through SDS-PAGE analysis. Protein concentrations were determined through absorbance at 280 nm before the purified proteins were aliquoted and stored at -20°C until further use.

**H) Screening in 96-DWPs using SAM stoichiometrically (HPLC-DAD/MS screening):**
General procedures:
*Lysis preparation:* Frozen cell pellets prepared as mentioned in section E were resuspended in 200 μL/well lysis buffer (50 mM KPi pH 7, 1 mg/mL lysozyme, 1.2 μL/100 mL Pierce™ Universal Nuclease). The cells were lysed at r.t. (room temperature) on a plate shaker at 450 rpm over 2 h. The cell debris was separated from the cell-free extract through centrifugation (4300 rcf, 15 min, 4°C).

*Reaction conditions 1:* Biotransformations (400 μL/well) were performed in 96-DWPs by mixing 202 μL/well reaction buffer (50 mM KPi pH 7), 150 μL/well of the cell-free extract (prepared as described above), 8 μL/well substrate stock solution (100 mM in DMSO, 2 mM final concentration), and 40 μL/well SAM stock solution (20 mM in reaction buffer, 2 mM final concentration). The reactions were sealed with an adhesive film (VWR polyester film, Cat.No.: 60941-062) and shaken on a plate shaker at 450 rpm, r.t. for 4, 20, or 24 h. Acetonitrile was added to the reaction mixture (600 μL/well) and the DWPs were shaken for another 30 mins. Precipitated proteins were separated from the supernatant through centrifugation (4°C, 10 min, 4300 rcf) and 200 μL of the supernatant were transferred to a 96-microtiter plate (Corning® 96-well Clear Round Bottom Polypropylene, Cat.No.: 3365) by centrifugal filtration (AcroPrep™ Advance 96 Filter Plate, 0.2 μm PTFE, Pall Corporation, 4°C, 10 min, 4300 rcf). The screening plates were heat-sealed (Vitl 38 μm Heat Sealing Foil, Cat.No.: V901003) for subsequent HPLC-DAD/MS analysis.

*Reaction conditions 2:* Biotransformations (200 μL/well) were performed in 96-DWPs by mixing 101 μL/well reaction buffer (50 mM KPi pH 7), 75 μL/well of the cell-free extract (prepared as described above), 8 μL/well substrate stock solution (50 mM in DMSO, 2 mM final concentration), and 20 μL/well SAM stock solution (20 mM in reaction buffer, 2 mM final concentration). The reactions were sealed with an adhesive film and shaken on a plate shaker

at r.t. and 450 rpm for 20 h. Acetonitrile was added to the reaction mixture (300 μL/well) and the DWPs were shaken for another 30 mins. Precipitated proteins were separated from the supernatant through centrifugation (4°C, 10 min, 4300 rcf) and 200 μL of the supernatant were transferred to a 96-microtiter plate by centrifugal filtration. The screening plates were heat-sealed for subsequent HPLC-DAD/MS analysis.

Screening of the v31 SSM libraries in DWPs using GC-MS: A total of twelve SSM libraries were screened, targeting the active-site amino acids residues forming the substrate-binding pocket of v31 (Y20, Y24, L164, H167, D197, M198, C201, Y204, S213, F242, A247, S249). 5-bromo-1*H*-benzo[*d*]imidazole was used as the model substrate. Biotransformations (400 μl/well) were performed in 96-DWPs by mixing 202 μL/well reaction buffer (50 mM KPi pH 7), 150 μl/well of the cell-free extract (prepared as described in the general procedures of this section), 8 μL/well substrate stock solution (100 mM in DMSO, 2 mM final concentration), and 40 μL/well SAM stock solution (20 mM in reaction buffer, 2 mM final concentration). The 96-DWPs were sealed with an adhesive film and shaken on a plate shaker at 450 rpm, r.t. for 6 h. The biotransformations were stopped by adding 900 μl/well of an EtOAc / cyclohexane 1:1 (v/v) mixture containing 2-phenyl-1*H*-imidazole (0.5 mM) as an internal standard. After mixing, the 96-DWPs were centrifuged (4300 rcf. 10 min, 4°C) and the organic layers (600 μL) were transferred to GC-vials for GC-MS analysis (see section **Q**). Beneficial mutants identified during the screening were sequenced. To confirm activity, cell-free extract reactions were performed in 96-DWPs using ten replicas for each variant. Reactions using 5-bromo-1*H*-benzo[*d*]imidazole as substrate were conducted under the same conditions described above for the SSM libraries screening. The products were analyzed by GC-MS (see **Figure S7A**).

Screening of the SOLD library with 5-bromo-1*H*-benzo[*d*]imidazole and construction of the final combinatorial library (vS01-vS46): The SOLD library was screened following reaction conditions 1 described in the general procedures (**H**). Biotransformations were stopped after 4 h and analyzed by HPLC-DAD (described in section **O i.**). A total of fourteen 96-DWPs were screened. Variants exhibiting improved activity relative to the parent (v31) were collected and sequenced, resulting in 46 unique mutants (vS01-vS46) that formed the combinatorial library used in subsequent screenings.

Screening of the final combinatorial library for *N*-methylation of functionalized azoles (products 1-10): The final combinatorial library (vS01-vS46) was screened following the general procedures (**H**), with reaction conditions 2 applied in 20 h biotransformations. The 96-DWPs were analyzed by HPLC-MS (detailed methods are described in section **O i.**).

Screening for late-stage *N*-methylation (products 13-15)**:** Variants from the final combinatorial library (vS01-vS46), the FuncLib-derived *hsa*-N-NMT variants (v01-v50) and wild type MTs (50 variants, see **table S2**) were screened using compound **12** as substrate. The biotransformations were conducted following reaction conditions 1 described in the general procedures (**H**) for 24 h. Product formation was analyzed by HPLC-MS (described in section **O iv.**).

**I) 96-DWP rescreening of the combinatorial library (vS01-vS46) using the cyclic two-enzyme cascade:** The best performing variants found during the screening of the SOLD library with 5-bromo-1*H*-benzo[*d*]imidazole (section **H**) were rescreened using the same substrate in a 96-DWP to confirm the activity. To eliminate potential inhibitory effects caused by SAH accumulation, the reactions were conducted as free cell lysates (prepared as described in section **H**), using the cyclic enzyme cascade with purified *acl*-MT wild-type and iodomethane as methyl source. Biotransformations (400 μL/well) were performed by mixing 234 μL/well of purified *acl*-MT wild-typ (2 μM final concentration) in reaction buffer (50 mM KPi pH 7), 8 μL/well of a substrate/SAH stock solution (100 mM substrate, 2 mM final concentration, and 1 mM SAH, 2 μM final concentration in a 1:1 DMSO/buffer mixture), 150 μL/well of the cell-free extract, and 8 μL/well MeI stock solution (1 M in DMSO, 20 mM final concentration). The 96-DWP was sealed and shaken at 450 rpm at room temperature for 6 h. To quench the reactions, 900 μl/well of an EtOAc / cyclohexane 1:1 (v/v) mixture containing 1-indanone (0.5 mM, as internal standard) was added. After mixing, the plate was centrifuged (4300 rcf, 10 min, 4°C) and the organic layers (700 μL) were transferred to GC-vials for GC-MS analysis (see **Figure S7C**, and section **Q** for description of the GC method).

**J) Rescreening with purified enzymes using MeI in the cyclic two-enzyme cascade:** Analytical scale reactions were performed to determine the selectivity of variants found during the screening for selective *N*-methylation of functionalized azoles and late-stage *N*-methylation of substrate **12**. To discard potential inhibitory effects caused by the stoichiometric formation of SAH, the reactions were performed with the cyclic two-enzyme cascade using iodomethane as the methyl donor. Variants selected from the screening experiments and *acl*-MT wild-typ were purified as described in section **G**. Reactions without enzymes were prepared as controls. 500 μl reactions were prepared in triplicates using 2.5 mL flat-bottom glass test tubes (Carl Roth, No. 01.2400.257). Before starting, purified frozen enzymes were thawed rapidly in a water bath at room temperature and kept on ice. The respective *N*-heteroarene (2 mM final concentration) was mixed with SAH (1 mol%, 20 μM), the purified enzymes (1 mol % of both enzymes, *acl*-MT wild-type and the corresponding NMT), and the alkyl halide (5 equiv.) in a KPi buffer (50 mM, pH 7.0, with a final 2-3% (v/v) DMSO). The reaction vials were capped and shaken on a thermomixer at 450 rpm, 30°C for 20 h. They were quenched by adding acetonitrile (750 μL) and incubated at 450 rpm, r.t. for 30 min. The precipitated enzymes were separated through centrifugation (21000 rcf, 4°C, 15 min) and the cleared reaction solutions were transferred into glass vial for HPLC analysis (see methods in section **O ii.**, see HPLC traces in **figure 3** and **S8**).

**K) DWP screening of NMT in-house library for alkylation activity using HPLC-MS:** All in-house NMT variants / wild types (encoded on the pET28a(+) were produced using the BL21 strain) and screened for the transfer of (iodomethyl)cyclopropane, using *acl*-MT v02 for SAM analog (re)generation. The used BL21 *E. coli* strain contains enzymes for the SAH degradation, therefore, we developed a screening set up based on the preparation of cosubstrate analogs before the reaction to ensure that enough cosubstrate is present for the identification of alkyl transfer activity. Therefore, a cosubstrate analog master mix (6 mL / DWP) was prepared in advance to generate SAM analogs before the cell-free-extract containing the *N*-alkyltransferase was added.

Cosubstrate analog master mix: For 6 mL SAM analog master mix, 4.37 mL 50 mM KPi buffer pH 7.0, 240 μL 50 mM SAH stock solved in pure DMSO, and 1.15 mL of 156.6 μM *acl*-MT v02 stock was mixed in a 15 mL reaction tube and the SAM analog synthesis was started through the addition of freshly prepared 240 μL 150 mM (iodomethyl)cyclopropane DMSO stock. Cosubstrate analog master mix final concentration: 2 mM SAH, 6 mM (3 equiv.) (Iodomethyl)cyclopropane, 20 μM (1 mol%) *acl*-MT v02 and 8% DMSO. This prepared master mix was incubated at r.t. for 30 min on a bench top shaker at 450 rpm.

Cascade screening of in-house NMT library: For the preparation of the NMT cell-free extract, frozen cell pellets (prepared as indicated in section **G**) were resuspending in 400 μL/well lysis buffer (50 mM KPi, pH 7.0, 1 mg/mL Lysozyme, 3 U/mL Pierce™ Universal Nuclease) and enzymatically lysed over 2 h on a bench shaker at r.t. and 450 rpm. Insoluble cell debris was separated from cell-free extract through centrifugation (4300 rcf, 15 min, 4°C). NMT biotransformations using cell-free extract have been performed in DWP (200 μL/well). Each well contained, 50 μL of the SAM analog master mix, 140 μL of the cleared cell-free NMT extract, and the reaction was started through the addition of 10 μL 20 mM 5-bromo-1*H*-benzo[*d*]imidazole stock (1:9 DMSO/$H_2O$). This DWP cascade screening reaction finally contained: 2 mM 5-bromo-1*H*-benzo[*d*]imidazole, 3 mM (1.5 equiv.) haloalkane, 5 μM (0.25 mol%) *acl*-MT v02, and 3% (v/v) DMSO. The plates were tightly sealed (Sealing mats, AB-0675, Thermo Fisher Scientific Inc.) and shaken for 2 h and 450 rpm r.t. on a benchtop shaker before the reactions were stopped after 4 h through the addition of 300 μL acetonitrile and incubated at r.t. for 30 min on a benchtop shaker at 450 rpm. Precipitated proteins and reaction components were separated through centrifugation (4°C, 4300 rcf, 15 min) and 200 μL of the cleared reaction solution was transferred into a fresh round bottom polypropylene microtiterplate (Corning® 96-well Clear Round Bottom Polypropylene, Cat.No.: 3365, Corning Inc.) and heat-sealed (Vitl 38 μm Heat Sealing Foil, Cat.No.: V901003) for subsequent HPLC-MS analysis described in section **O vii**.

**L) Screening of *dre*-H-NMT SSM libraries in 96 DWP using HPLC-MS:** Frozen cell pellets (derived from section **G**) were resuspended in 200 μL/well lysis buffer, containing 50 mM KPi pH 7.5 (1 mg/mL lysozyme, 3 U/mL Pierce™ Universal-Nuclease) and incubated at r.t. for 2 h at 450 rpm on a benchtop shaker. Cell debris was separated from the soluble cell-free extract fraction through centrifugation (15 min, 4300 rcf, 4 °C). Biotransformations (200 μL/well) were performed in DWPs, therefore, 50 μL/well of a master mix containing 4 mM of 5-bromo-1*H*-benzo[*d*]imidazole, 40 μM of purified *acl*-MT v02, and 400 μM SAH was prepared in 50 mM KPi Buffer and aliquoted into the 96-DWP before 148 μL of the cleared cell-free extract was added and the reaction was started through the addition of 2 μL of a 300 mM (iodomethyl)cyclopropane DMSO stock. Final concentrations: 1 mM 5-bromo-1*H*-benzo[*d*]imidazole, 3 mM (3 equiv.) (iodomethyl)cyclopropane, 100 μM (10 mol%) SAH, 10 μM (1 mol%) purified *acl*-MT v02, 3% (v/v) DMSO. The plates were tightly sealed (Sealing mats, AB-0675, Thermo Fisher Scientific Inc.) and shaken for 2 h and 450 rpm r.t. on a benchtop shaker. After 4 h the reactions were stopped through the addition of 300 μL acetonitrile and proteins were allowed to precipitate for 30 min on a benchtop shaker (600 rpm). Precipitated proteins and reaction components were separated through centrifugation (4 °C, 4300 rcf, 15 min) and 200 μL of the cleared reaction solution was

transferred into a fresh round bottom polypropylene microtiterplate (Corning® 96-well Clear Round Bottom Polypropylene, Cat.No.: 3365, Corning Inc.) and heat-sealed (Vitl 38 μm Heat Sealing Foil, Cat.No.: V901003) for subsequent HPLC-MS analysis described in section **O vii**.

**M) Identification best performing *dre*-H-NMT hits:** After each round of engineering, the best performing *dre*-H-HNMT variants derived from the SSM DWP screening (see section **L**) were sequenced, expressed on a 500 mL scale (see section **F**), purified (see section **G**) and tested as purified enzyme to identify the best performing variant of each round.

*Reaction conditions for the identification of the 1st round variants:*

The first round of engineering had the aim to increase the selectivity of the enzyme while maintaining a high activity. Biotransformations were conducted on analytical scale as technical triplicates (n=3). 400 μL reactions were performed in a 2 mL glass vial containing: 50 mM KPi buffer, 20 μM (1 mol%) purified *dre*-H-NMT variant, 20 μM (1 mol%) *acl*-MT v02, 4 μL of a 2 mM SAH DMSO stock (final concentration: 20 μM, 1 mol%), and 4 μL of a 200 mM 5-bromo-1*H*-benzo[*d*]imidazole DMSO stock (final concentration: 2 mM) before the reaction was started through the addition of 4 μL of a freshly prepared 600 mM (iodomethyl)cyclopropane DMSO stock (final concentration: 6 mM, 3 equiv.). Reactions were incubated at 25°C for 20 h before 600 μL of acetonitrile was added to quench the enzymatic reaction, mixed, and incubated at r.t. for 30 min to allow the proteins to precipitate. The resulting precipitated reaction components were separated through centrifugation (21000 rcf, 4°C, 15 min) and the cleared reaction solution was transferred into a fresh glass vial for subsequent HPLC-MS analysis (for HPLC methods, see section **O viii**). The *dre*-H-NMT C198Y variant showed the highest selectivity r.e. 98% and was used as template for the second round of engineering.

*Reaction conditions for the identification of the 2nd round variants:*

The goal of the 2nd round of engineering was to increase the activity. Biotransformations were conducted on analytical scale as technical triplicates (n=3). 400 μL reactions were performed in a 2 mL glass vial containing: 50 mM KPi buffer, 20 μM (1 mol%) purified *dre*-H-NMT variant, 20 μM (1 mol%) *acl*-MT v02, 4 μL of a 2 mM SAH DMSO stock (final concentration: 20 μM, 1 mol%), and 4 μL of a 200 mM 5-bromo-1*H*-benzo[*d*]imidazole DMSO stock (final concentration: 2 mM) before the reaction was started through the addition of 4 μL of a freshly prepared 600 mM (iodomethyl)cyclopropane DMSO stock (final concentration: 6 mM, 3 equiv.). Reactions were incubated at 25°C for 1 h before 600 μL of acetonitrile was added to quench the enzymatic reaction, mixed, and incubated at r.t. for 30 min to allow the proteins to precipitate. The resulting precipitated reaction components were separated through centrifugation (21000 rcf, 4°C, 15 min) and the cleared reaction solution was transferred into a fresh glass vial for subsequent HPLC-MS analysis (for HPLC methods, see section **O viii**). The *dre*-H-NMT Y15A / C198Y variant maintained the high selectivity (r.e. 98%) and showed an activity increase.

**N) Alkylation substrate scope of *dre*-H-NMT Y15A / C198Y:** The engineered *dre*-H-NMT Y15A / C198Y was coupled with the previously engineered and identified *acl*-MT v16, serving as a promiscuous anion MT for cosubstrate regeneration using a diverse set of haloalkanes (Fig. 4). Single analytical scale reaction (400 μL) were performed in 1.5 mL glass vials with

screw caps for each tested haloalkane containing: 50 mM KPi pH 7.0, 4 µL of a 200 mM 5-bromo-1*H*-benzo[*d*]imidazole DMSO stock (final concentration: 2 mM), 4 µL of a 2 or 20 mM SAH DMSO stock (final concentration: 20 or 200 µM, 1 or 10 mol%), 20 µM (1 mol%) purified *dre*-H-NMT variant, and 20 µM (1 mol%) *acl*-MT v16 before the reaction were started trough the addition of 4 µL freshly prepared 600 mM haloalkane DMSO stock (final concentration: 6 mM, 3 equiv.). Reactions were incubated at r.t. on a benchtop shaker for 20 h at 450 rpm before 600 µL acetonitrile was added to quench the reaction. Proteins were allowed to denature for 30 min at r.t. before precipitated reaction compounds were separated through centrifugation (21000 rcf, 4°C, 15 min) and the cleared reaction solution was transferred into a fresh glass vial for subsequent HPLC-MS analysis (for HPLC methods, see section **O viii**).
After verifying the successful alkylation of 5-bromo-1*H*-benzo[*d*]imidazole through the bienzymatic cascade, the reactions were optimized by screening previously identified promiscuous *acl*-MT variants (v02, v15, v16, v31, v55, v63) to enhance the product formation (Fig. S10). Reactions conditions were kept the same as mentioned during the substrate scope screening while changing the employed *acl*-MT variant and concentration of SAH.
Final standard reaction conditions: 2 mM 5-bromo-1*H*-benzo[*d*]imidazole, 6 mM iodo-, bromo-, or chloro-alkanes, 20 or 200 µM (1-10 mol%) SAH, 20 µM (1 mol%) *dre*-H-NMT, 20 µM (1 mol%) *acl*-MT variant, r.t., 20 h. Calibration curves of synthesized product standards (see section **IV**) were used to quantify the obtained DAD areas and used to calculate the derived HPLC yields, recovery rate and selectivity.

| **Product** | **Final reaction condition** |
|---|---|
| 16 | 2 mM 5-bromo-1*H*-benzo[*d*]imidazole, 6 mM iodoethane, 20 µM (1 mol%) SAH, 20 µM (1 mol%) *dre*-H-NMT Y15A / C198Y, 20 µM (1 mol%) *acl*-MT v15, r.t., 20 h. |
| 17 | 2 mM 5-bromo-1*H*-benzo[*d*]imidazole, 6 mM (iodomethyl)cyclopropane, 20 µM (1 mol%) SAH, 20 µM (1 mol%) *dre*-H-NMT Y15A / C198Y, 20 µM (1 mol%) *acl*-MT v02, r.t., 20 h. |
| 18 | 2 mM 5-bromo-1*H*-benzo[*d*]imidazole, 6 mM (iodomethyl)cyclobutane, 20 µM (1 mol%) SAH, 20 µM (1 mol%) *dre*-H-NMT Y15A / C198Y, 20 µM (1 mol%) *acl*-MT v63, r.t., 20 h. |
| 19 | 2 mM 5-bromo-1*H*-benzo[*d*]imidazole, 6 mM 3-bromoprop-1-ene, 20 µM (1 mol%) SAH, 20 µM (1 mol%) *dre*-H-NMT Y15A / C198Y, 20 µM (1 mol%) *acl*-MT v02, r.t., 20 h. |
| 20 | 2 mM 5-bromo-1*H*-benzo[*d*]imidazole, 6 mM 3-bromo-2-methylprop-1-ene, 200 µM (10 mol%) SAH, 20 µM (1 mol%) *dre*-H-NMT Y15A / C198Y, 20 µM (1 mol%) *acl*-MT v55, r.t., 20 h. |
| 21 | 2 mM 5-bromo-1*H*-benzo[*d*]imidazole, 6 mM 3-bromo-2-fluoroprop-1-ene, 200 µM (10 mol%) SAH, 20 µM (1 mol%) *dre*-H-NMT Y15A / C198Y, 20 µM (1 mol%) *acl*-MT v02, r.t., 20 h. |
| 22 | 2 mM 5-bromo-1*H*-benzo[*d*]imidazole, 6 mM 2-bromoacetonitrile, 20 µM (1 mol%) SAH, 20 µM (1 mol%) *dre*-H-NMT Y15A / C198Y, 20 µM (1 mol%) *acl*-MT v15, r.t., 20 h. |
| 23 | 2 mM 5-bromo-1*H*-benzo[*d*]imidazole, 6 mM 3-bromoprop-1-yne, 20 µM (1 mol%) SAH, 20 µM (1 mol%) *dre*-H-NMT Y15A / C198Y, 20 µM (1 mol%) *acl*-MT v16, r.t., 20 h. |
| 23 | 2 mM 5-bromo-1*H*-benzo[*d*]imidazole, 6 mM 3-bromoprop-1-yne, 20 µM (1 mol%) SAH, 20 µM (1 mol%) *dre*-H-NMT Y15A / C198Y, 20 µM (1 mol%) *acl*-MT v16, r.t., 20 h. |

**O) High-performance liquid chromatography (HPLC) analysis:** Analytical HPLC and electron spray ionization (ESI) mass spectrometry were carried out using an Agilent Infinity II 1290 system equipped with a Flexible Pump (G71041), a multisampler with dual needle (G7167B), a DAD (G7117B) with a 10 mM Max-Light Cartridge Cell (G4212 60008), and an InfinityLab LC/MSD iQ Mass Selective detector (G6160A). For the detection of azoles, the wavelength and the m/z value of each molecular ion (M+H) in single ion monitoring (SIM) mode were selected based on the azole derivative. The separations were performed at a flow rate of 0.8 mL/min, and a temperature of 40°C, using a 0.5 μL injection on the following stationary systems:

Column **a**: Infinity Poroshell 120 Phenyl Hexyl column (3.0 x 50 mm, 2.7 μm, Part number: 699975-312) coupled to a corresponding pre-column (3.0 x 5 mm, 2.7 μm, Part number: 823750-914).

Column **b**: Infinity Poroshell 120 SB-C18 column (3.0 x 50 mm, 2.7 μm, Part number: 689975-302) coupled to a corresponding pre-column (3.0 x 5 mm, 2.7 μm, Part number: 823750-912).

Column **c**: Infinity Poroshell 120 EC-C18 column (2.1 x 50 mm, 2.7 μm, Part number: 699775-902) coupled to a corresponding pre-column (2.1 x 5 mm, 2.7 μm, Part number: 699775-902K).

Column **d**: Infinity Poroshell 120 EC-C18 column (2.1 x 50 mm, 1.9 μm, Part number: 699675-902) coupled to a corresponding pre-column (2.1 x 5 mm, 1.9 μm, Part number: 821725-940).

*i) HPLC-DAD methods for screening N-methylation of functionalized azoles*

1-Methyl-1*H*-benzo[*d*]imidazol-5-amine (**1**): Column **b**, DAD: 214 nm, mobile phase: water (10 mM ammonium formate pH 6.9, A) / acetonitrile (B). Gradient: start with 95% A, decrease to 90% A over 0.5 min, hold for 1.2 min, to 10% A over 0.8 min, return to 95% A over 0.8 min, and hold for 0.5 min.

1-Methyl-1*H*-benzo[*d*]imidazol-5-ol (**2**): Column **b**, DAD: 202 nm, mobile phase: water (10 mM ammonium formate pH 6.9, A) / acetonitrile (B). Gradient: start with 90% A, hold for 1.7 min, decrease to 10% A over 0.8 min, return to 90% A over 0.8 min, and hold for 0.5 min.

1-Methyl-5-nitro-1*H*-benzo[*d*]imidazole (**3**): Column **b**, DAD: 278 nm, mobile phase: water (0.1% v/v formic acid, A) / acetonitrile (B). Gradient: start with 88% A, decrease to 70% A over 2.2 min, to 10% A over 0.4 min, hold for 0.4 min, and return to 88% A over 0.2 min.

1-Methyl-1*H*-benzo[*d*]imidazole-5-carbonitrile (**4**): Column **b**, DAD: 212 nm, mobile phase: water (10 mM ammonium formate pH 2.7, A) / acetonitrile (B). Gradient: start with 90% A, decrease to 70% A over 1.4 min, to 10% A over 0.8 min, return to 90% A over 1.0 min, and hold 0.6 min.

1-Methyl-1*H*-benzo[*d*]imidazole-5-carbaldehyde (**5**): Column **c**, DAD: 210 nm, mobile phase: water (A) / acetonitrile (B). Gradient: start with 90% A, hold for 4 min, decrease to 10% A over 1 min, hold for 1 min, return to 90% A over 1 min, and hold for 0.5 min.

5-Methoxy-1-methyl-1*H*-benzo[*d*]imidazole (**6**): Column **d**, DAD: 278 nm, m/z: 162.9, mobile phase: water (0.1% v/v formic acid, A) / acetonitrile (B). Gradient: start with 95% A, decrease

to 92% A over 0.2 min, to 90% A over 1.8 min, to 10% A over 0.5 min, and return to 95% A over 0.3 min.

5-Fluoro-1-methyl-1*H*-benzo[*d*]imidazole (**7**): Column **b**, DAD: 278 nm, m/z: 150.9, mobile phase: water (0.1% v/v formic acid, A) / acetonitrile (B). Gradient: start with 92% A, hold for 3 min, decrease to 10% A over 0.8 min, and return to 92% A over 0.2 min.

5-Chloro-1-methyl-1*H*-benzo[*d*]imidazole (**8**): Column **c**, DAD: 278 nm, m/z: 166.8, mobile phase: water (0.1% v/v formic acid, A) / acetonitrile (B). Gradient: start with 88% A, hold for 0.5 min, decrease to 80% A over 0.3 min, to 60% A over 1.7 min, to 10% A over 0.3 min, and return to 88% over 0.2 min.

5-Iodo-1-methyl-1*H*-benzo[*d*]imidazole (**9**): Column **b**, DAD: 218 nm, mobile phase: water (10 mM ammonium formate pH 5.7, A) / acetonitrile (B). Gradient: start with 60% A, decrease to 55% A over 1.6 min, to 10% A over 0.5 min, and return to 60% A over 0.5 min.

5-Bromo-1,2-dimethyl-1*H*-benzo[*d*]imidazole (**10**): Column **b**, DAD: 278 nm, m/z: 225.0, mobile phase: water (0.1% v/v formic acid, A) / acetonitrile (B). Gradient: start with 88% A, hold for 0.5 min, decrease to 80% A over 0.3 min, to 60% A over 1.7 min, to 10% A over 0.3 min, and return to 88% A over 0.2 min.

1-Methyl-5-bromo-1*H*-benzo[*d*]imidazole (**11b**): Column **c**, 1 μL injection, flow rate: 1 mL/min, DAD: 210 nm, mobile phase: water (A) / acetonitrile (B). Gradient: start with 70% A, hold for 2.5 min, decrease to 10% A over 0.75 min, hold for 0.5 min, return to 70% A over 1.25 min, and hold for 0.5 min.

*ii) HPLC-MS methods for rescreening N-methylation of functionalized azoles*

1-Methyl-1*H*-benzo[*d*]imidazol-5-amine (**1**): Column **c**, DAD: 302 nm, m/z: 147.7, mobile phase: water (10 mM ammonium formate pH 5.7, A) / acetonitrile (B). Gradient: start with 95% A, hold for 1.0 min, decrease to 10% A over 2.0 min, and return to 95% A over 0.3 min.

1-Methyl-1*H*-benzo[*d*]imidazol-5-ol (**2**): Column **b**, DAD: 288 nm, m/z: 148.8, mobile phase: water (10 mM ammonium formate pH 5.7, A) / acetonitrile (B). Gradient: start with 95% A, decrease to 92% A over 1.7 min, to 90% A over 0.5 min, to 10% A over 0.3 min, and return to 95% A over 0.3 min.

1-Methyl-5-nitro-1*H*-benzo[*d*]imidazole (**3**): Column **b**, DAD: 278 nm, m/z: 178.0, mobile phase: water (0.1% v/v formic acid, A) / acetonitrile (B). Gradient: start with 88% A, decrease to 70% A over 2.2 min, to 10% A over 0.4 min, hold for 0.4 min, and return to 88% A over 0.2 min.

1-Methyl-1*H*-benzo[*d*]imidazole-5-carbonitrile (**4**): Column **b**, DAD: 212 nm, m/z: 157.8, mobile phase: water (0.1% v/v formic acid, A) / acetonitrile (B). Gradient: start with 90% A, decrease to 74% A over 1.4 min, to 10% A over 0.8 min, return to 90% A over 1.0 min, and hold for 0.6 min.

1-Methyl-1*H*-benzo[*d*]imidazole-5-carbaldehyde (**5**): Column **b**, DAD: 286 nm, m/z: 160.9, mobile phase: water (0.1% v/v formic acid, A) / acetonitrile (B). Gradient: start with 95% A, decrease to 85% A over 2.2 min, to 10% A over 0.1 min, and return to 95% A over 0.7 min.

5-Methoxy-1-methyl-1*H*-benzo[*d*]imidazole (**6**): Column **b**, DAD: 288 nm, m/z: 162.9, mobile phase: water (10 mM ammonium formate pH 3.1, A) / acetonitrile (B). Gradient: start with 95% A, hold for 4.0 min, decrease to 10% A over 0.5 min, and return to 95% A over 0.5 min.

5-Fluoro-1-methyl-1*H*-benzo[*d*]imidazole (**7**): Column **b**, DAD: 250 nm, m/z: 150.9, mobile phase: water (10 mM ammonium formate pH 5.7, A) / acetonitrile (B). Gradient: start with 90% A, decrease to 80% A over 1.7 min, hold for 0.5 min, decrease to 75% A over 0.8 min, to 65% A over 0.5 min, to 10% A over 0.1 min, and return to 90% A over 0.4 min.

5-Chloro-1-methyl-1*H*-benzo[*d*]imidazole (**8**): Column **b**, DAD: 218 nm, m/z: 166.8, mobile phase: (10 mM ammonium formate pH 5.7, A) / acetonitrile (B). Gradient: start with 75% A, decrease to 70% A over 1.7 min, hold for 0.5 min, decrease to 10% A over 0.3 min, return to 75% A over 0.5 min, and hold for 1.0 min.

5-Iodo-1-methyl-1*H*-benzo[*d*]imidazole (**9**): Column **b**, DAD: 218 nm, m/z: 258.7, mobile phase: water (10 mM ammonium formate pH 5.7, A) / acetonitrile (B). Gradient: start with 70% A, decrease to 65% A over 1.7 min, to 10% A over 0.8 min, return to 70% A over 0.5 min, and hold for 1.0 min.

5-Bromo-1,2-dimethyl-1*H*-benzo[*d*]imidazole (**10**): Column **b**, DAD: 284 nm, m/z: 224.8, mobile phase: water (10 mM ammonium formate pH 5.7, A) / acetonitrile (B). Gradient: start with 75% A, decrease to 70% A over 1.7 min, hold for 0.5 min, decrease to 10% A over 0.8 min, return to 77% A over 0.5 min.

*iii) HPLC-MS method for screening late-stage N-methylation:*

Column **a**, DAD: 280 nm, m/z: 255.2 (mono-methylation), 269.2 (di-methylation), mobile phase: water (10 mM ammonium formate pH 5.7, A) / acetonitrile (B). Gradient: start with 94% A, decrease to 85% A over 3.0 min, to 50% A over 0.5 min, return to 94% A over 1.0 min, and hold for 1.4 min.

*iv) HPLC-MS method for rescreening and selectivity determination in late-stage N-methylation:*

Column **a**, DAD: 280 nm, m/z: 255.2 (mono-methylation), 269.2 (di-methylation), mobile phase: water (10 mM ammonium formate pH 5.7, A) / acetonitrile (B). Gradient: start with 94% A, decrease to 90% A over 1.8 min, to 75% A over 1.0 min, to 50% A over 0.2 min, and return to 94% A over 1.3 min.

*v) HPLC-MS method for selectivity determination in late-stage N-ethylation:*

Column **a**, DAD: 245 nm, m/z: 269.3 (mono-ethylation), 297.4 (di-ethylation), mobile phase: water (10 mM ammonium formate pH 5.7, A) / acetonitrile (B). Gradient: start with 95% A, decrease to 80% A over 3.0 min, to 30% A over 3.0 min, to 10% A over 0.5 min, hold for 1.0 min, and return to 95% A over 1.0 min.

*vi) HPLC-MS method for selectivity determination in late-stage N-allylation:*

Column **a**, DAD: 245 nm, m/z: 281.3 (mono-allylation), 321.4 (di-allylation), mobile phase: water (10 mM ammonium formate pH 5.7, A) / acetonitrile (B). Gradient: start with 90% A, decrease to 75% A over 3.0 min, to 30% A over 3.0 min, to 10% A over 0.5 min, hold for 1.0 min, and return to 90% A over 1.0 min.

*vii) HPLC-MS screening for enzymatic methylcyclopropyl transfer:*

Column **a**, m/z: 252.1, mobile phase: 35:65 aqueous (0.1% v/v formic acid (A)) in methanol (B) for 1.3 min.

*viii) HPLC quantification and selectivity determination of enzymatic 5-bromo-1H-benzo[d]imidazole alkylation:*

For each of the alkylated product **16-23** dynamic methods were developed to allow separation of the alkylated regio isomers using the column **a** with 0.1% v/v formic acid (A) and methanol (B) as mobile phase.

5-Bromo-1-ethyl-1*H*-benzo[*d*]imidazole and 6-bromo-1-ethyl-1*H*-benzo[*d*]imidazole (**16**): m/z: 226.1, DAD: 245 nm, Gradient: start with 70% A, decrease to 50% A over 3.75 min, to 10% A over 0.25 min, hold for 0.75 min, return to 70% A over 0.5 min, and hold for 0.75 min.

5-Bromo-1-(cyclopropylmethyl)-1*H*-benzo[*d*]imidazole and 6-bromo-1-(cyclopropylmethyl)-1*H*-benzo[*d*]imidazole (**17**): m/z: 252.1, DAD: 245 nm, Gradient: start with 70% A, decrease to 50% A over 3.75 min, to 10% A over 0.25 min, hold for 0.75 min, return to 70% A over 0.5 min, and hold for 0.75 min.

5-Bromo-1-(cyclobutylmethyl)-1*H*-benzo[*d*]imidazole and 6-bromo-1-(cyclobutylmethyl)-1*H*-benzo[*d*]imidazole (**18**): m/z: 266.1, DAD: 245 nm, Gradient: start with 55% A, decrease to 45% A over 3.75 min, to 10% A over 0.25 min, hold for 0.75 min, return to 55% A over 0.5 min, and hold for 0.75 min.

1-Allyl-5-bromo-1*H*-benzo[*d*]imidazole and 1-allyl-6-bromo-1*H*-benzo[*d*]imidazole (**19**): m/z: 238.1, DAD: 245 nm, Gradient: start with 70% A, decrease to 50% A over 3.75 min, to 10% A over 0.25 min, hold for 0.75 min, return to 70% A over 0.5 min, and hold for 0.75 min.

5-Bromo-1-(2-methylallyl)-1*H*-benzo[*d*]imidazole and 6-bromo-1-(2-methylallyl)-1*H*-benzo[*d*]imidazole (**20**): m/z: 252.1, DAD: 245 nm, Gradient: start with 60% A, decrease to 50% A over 6.0 min, to 10% A over 0.5 min, hold for 1.0 min, return to 60% A over 0.75 min, and hold for 0.5 min.

5-Bromo-1-(2-fluoroallyl)-1*H*-benzo[*d*]imidazole and 6-bromo-1-(2-fluoroallyl)-1*H*-benzo[*d*]imidazole (**21**): m/z: 256.1, DAD: 245 nm, Gradient: start with 60% A, decrease to 50% A over 6.0 min, to 10% A over 0.5 min, hold for 1.0 min, return to 60% A over 0.75 min, and hold for 0.5 min.

5-Bromo-1-(prop-2-yn-1-yl)-1*H*-benzo[*d*]imidazole and 6-bromo-1-(prop-2-yn-1-yl)-1*H*-benzo[*d*]imidazole (**23**): m/z: 238.1, DAD: 245 nm, Gradient: start with 70% A, decrease to 50% A over 3.75 min, to 10% A over 0.25 min, hold for 0.75 min, return to 70% A over 0.5 min, and hold for 0.75 min.

**P) Preparative HPLC methods:** Preparative HPLC purifications were performed on a Shimadzu SCL-4 system equipped with a diode array detector (DAD) SPD-40V. Separation was monitored at 254 nm using a Luna C18(2) 100 Å column (250 × 21.2 mm, 5 μm) coupled with a corresponding pre-column.

i) Mobile phase: A: water (0.1% v/v formic acid):acetonitrile 95:5 (v/v), B: water (0.1% v/v formic acid):acetonitrile 5:95 (v/v). Gradient: start with 60% A, hold for 10 min, decrease to 30% A over 50 min, to 0% A over 10 min, and hold for 10 min.

iI) Mobile phase: A: water (0.1% v/v TFA):acetonitrile 95:5 (v/v), B: water (0.1% v/v TFA):acetonitrile 5:95 (v/v). Gradient: start with 85% A, hold for 1 min, decrease to 50% A over 50 min, to 0% A over 25 min, and hold for 10 min.

iii) Mobile phase: A: water (0.1% v/v TFA):acetonitrile 95:5 (v/v), B: water (0.1% v/v TFA):acetonitrile 5:95 (v/v). Gradient: start with 100% A, decrease to 90% A over 25 min, to 0% A over 1 min, and hold for 10 min.

**Q) Gas Chromatography-mass spectroscopy (GC-MS) analysis:** Gas chromatography-mass spectrometry was carried out on an Agilent 8860 GC system equipped with an 7693A Autosampler and coupled with an Agilent 5977B GC/MSD series mass selective detector (MSD). An Agilent DB-5MS UI (30 m × 0.25 mm × 0.25 μM, part number 122-5532UI) column or an HP-5MS UI (30 m × 0.25 mm × 0.25 μM, part number 19091S-433UI) was operated with helium as the carrier gas at a flow rate of 2.1 mL/min. The injector temperature was set at 280°C, with a split ratio of 15:1, and electron ionization at 70 eV. Detection and quantification were performed using a mass spectrometer in scan or SIM mode.

i) 2-(5-Bromo-1*H*-benzo[*d*]imidazol-1-yl)acetonitrile and 2-(6-bromo-1*H*-benzo[*d*]imidazol-1-yl)acetonitrile (**22**): The Agilent DB-5MS UI column was operated with the following temperature program: start with 100°C and hold for 1.0 min, increase to 250°C at a rate of 20°C/min, then to 310°C at a rate of 50°C/min, and hold for 0.3 min. (Internal standard: 2,6-dichloropyridine, scan mode)

ii) 1-Methyl-5-bromo-1*H*-benzo[*d*]imidazole (**11b**): The Agilent HP-5MS UI column was operated with the following temperature program for the screening of the v31 SSM libraries: start with 160°C and hold for 1.0 min, increase to 240°C at a rate of 30°C/min, and hold for 0.6 min. (Internal standard: 2-phenyl-1H-imidazole, SIM mode: 212.0 m/z).
The same column was operated with the following temperature program for the rescreening of the combinatorial library in the cyclic two-enzyme cascade: start with 130°C, hold for 1.0 min, and increase to 250°C at a rate of 30°C/min. (Internal standard: 1-indanone, SIM mode: 212.0 m/z).

## II. Supporting figures

**Figure S1.** Examples of different types of *N*-alkylated heteroarenes present in important approved drugs**.**

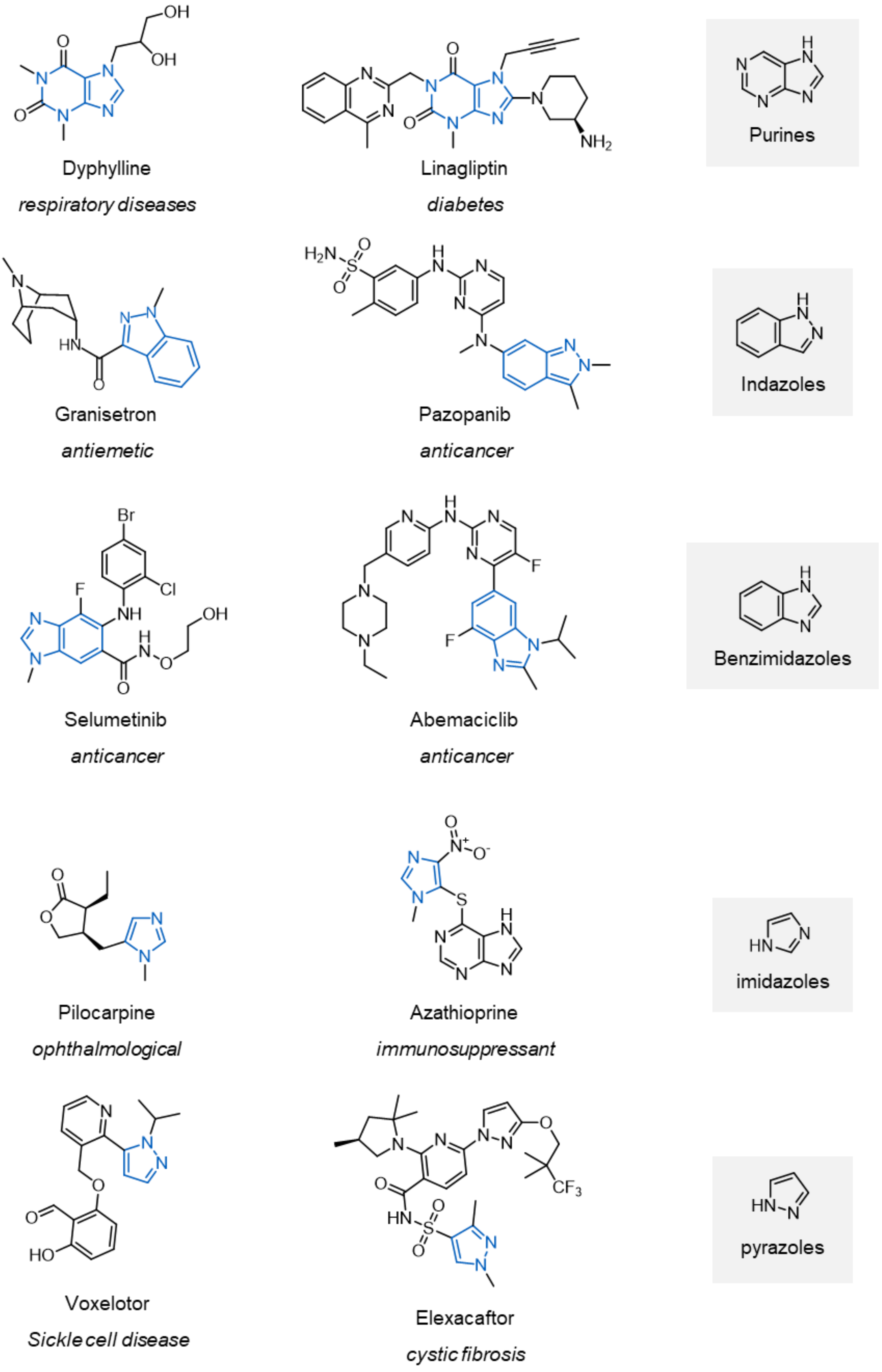

**Figure S2.** *N*-alkylation patterns influence the bioactivity of ambident heteroarenes.

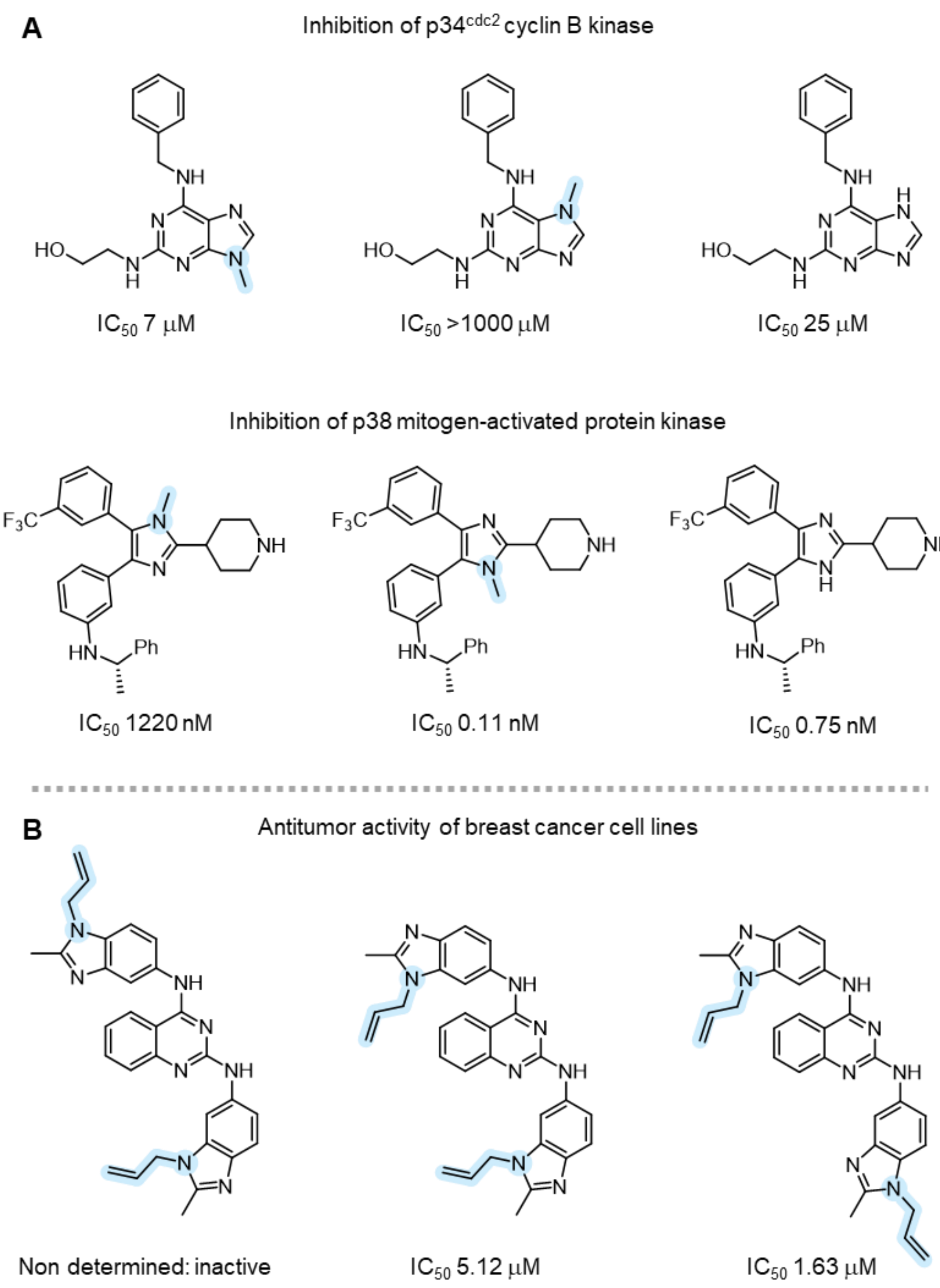


**A)** Structure-activity relationship studies highlight the critical impact of *N*-methylation on bioactivity. In both cases, *N*-methylation can enhance inhibitory activity. However, the most pronounced effect was observed when comparing the *N*-methylated regioisomers, with activity differences of over two orders in magnitude in the top case (>140-fold) and four orders of magnitude in the bottom case (>11000-fold). **B)** The activity anticancer profile varied among the *N*-allyl regioisomers shown. Notably, the alkylation pattern of the top-benzimidazole was crucial for activity. While the 1,5-disubstituted regioisomer (left) showed no growth inhibition against the tested cancer cell lines, the 1,6-regioisomers (middle and right) were the most active compounds in the study.[10–12]

**Figure S3.** Annular tautomerism equilibria of common *N*-heteroarene scaffolds.

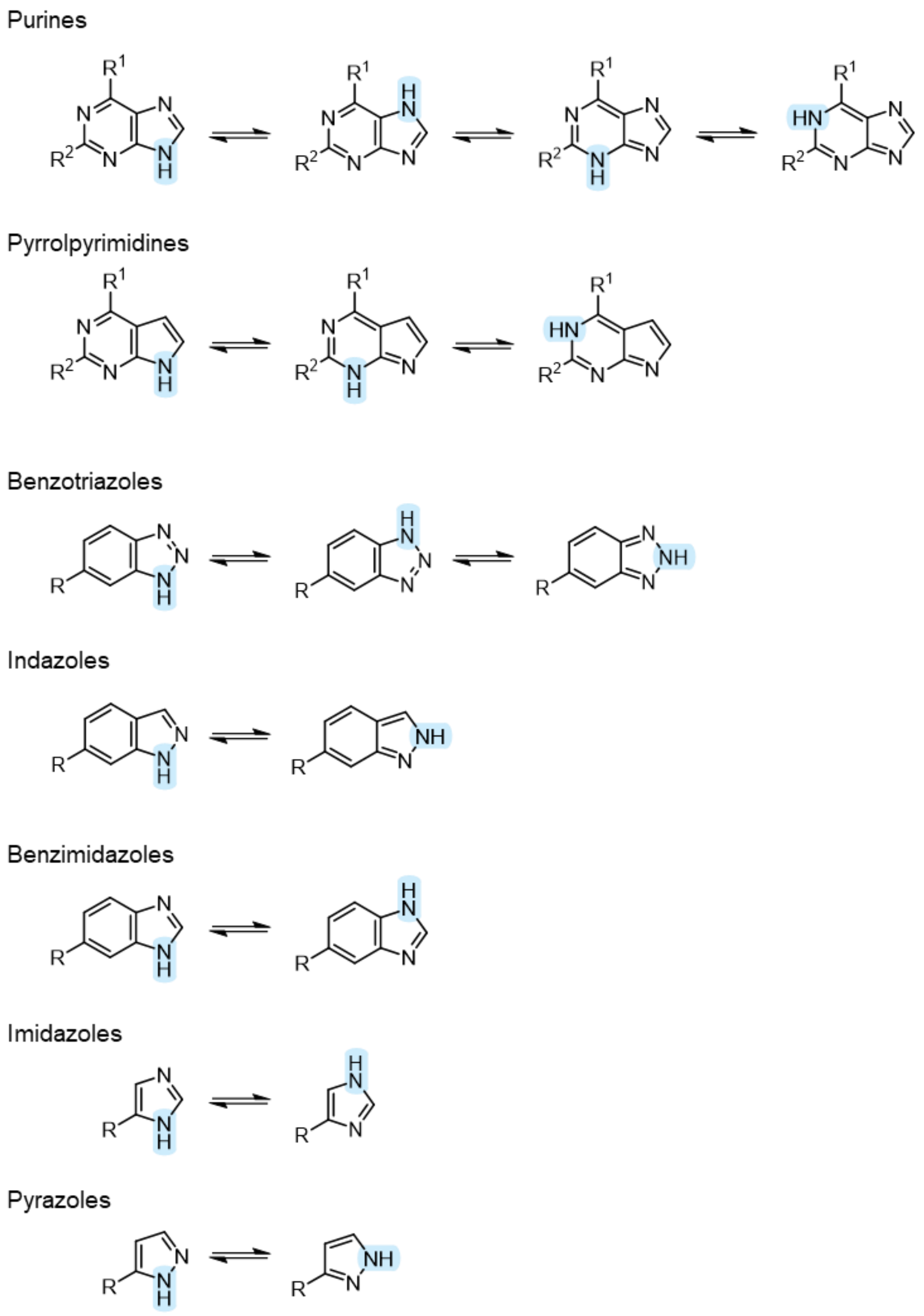


The fast proton migration renders nitrogen atoms with similar reactivity.

**Figure S4:** Summary of strategies for regioselective direct alkylation of *N*-heteroarenes.

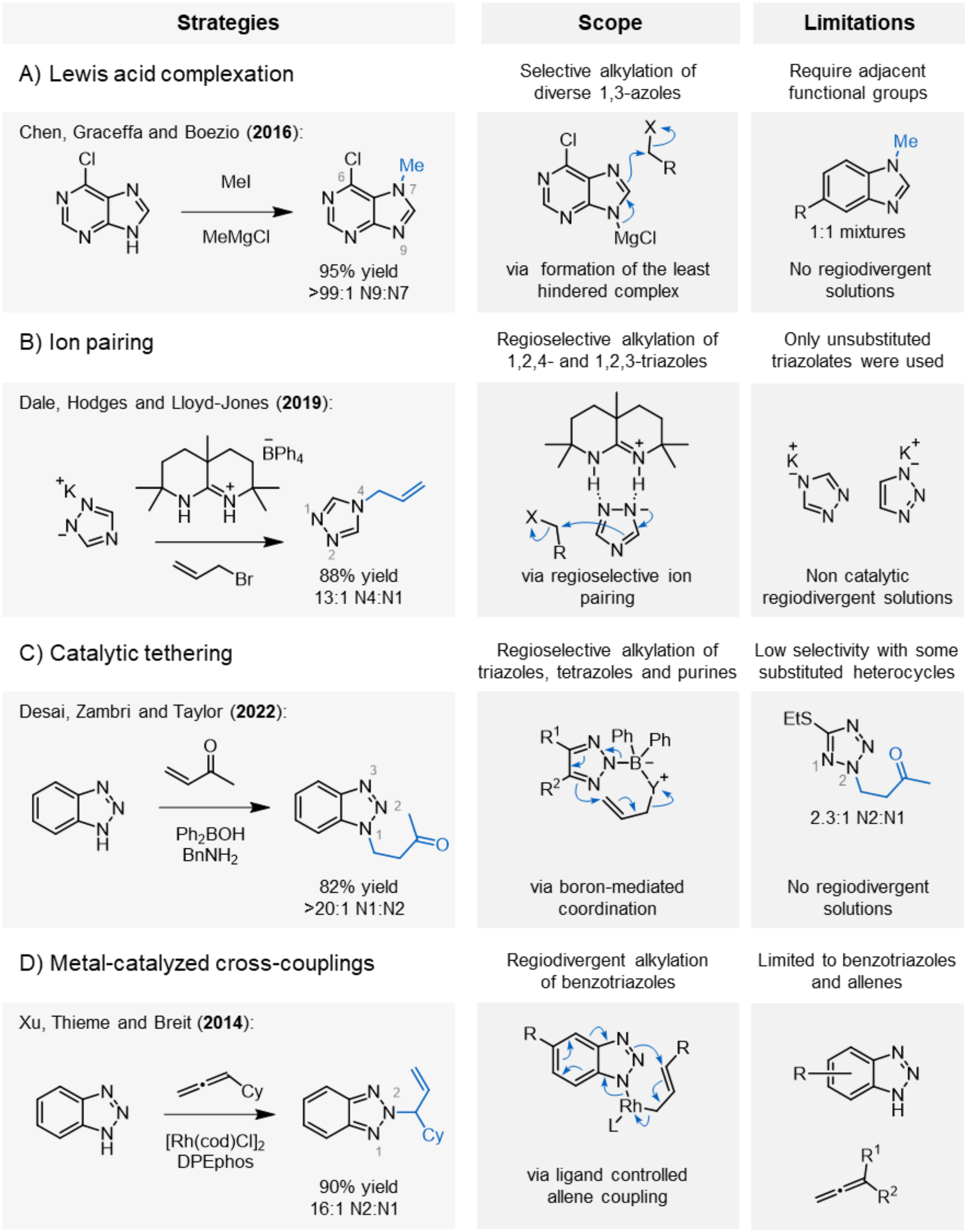


**A)**. Lewis acid complexation has been exploited as transient protecting group for selective alkylation of azoles, including purines, imidazoles, and pyrazoles. High selectivity requires proximal substituents near the competing nitrogen sites.[13–15] **B)** Amidinium organocatalysts act as *in situ* noncovalent protecting groups, forming tight ion pairs that enable selective alkylation of unsubstituted triazole anions.[16] **C)** Organoboron catalysis facilitates selective *N*-alkylation of ambident heteroarenes with electron-deficient alkenes via simultaneous coordination of both species. Highly selective examples are largely limited to unsubstituted *N*-heteroarenes.[17] **D)** Metal-catalyzed cross-coupling provides partial solutions for selective *N*-alkylation. Selectivity is dictated by ligand choice, but these methods are limited by the use of specific alkylation reagents such as allenes, diazo compounds, and certain olefins.[18–25]

**Figure S5.** Synthetic strategies to obtain *N*-alkylated azoles frequently depends on complex *de novo* ring synthesis as exemplified with 6-bromo-1*H*-benzo[*d*]imidazole.

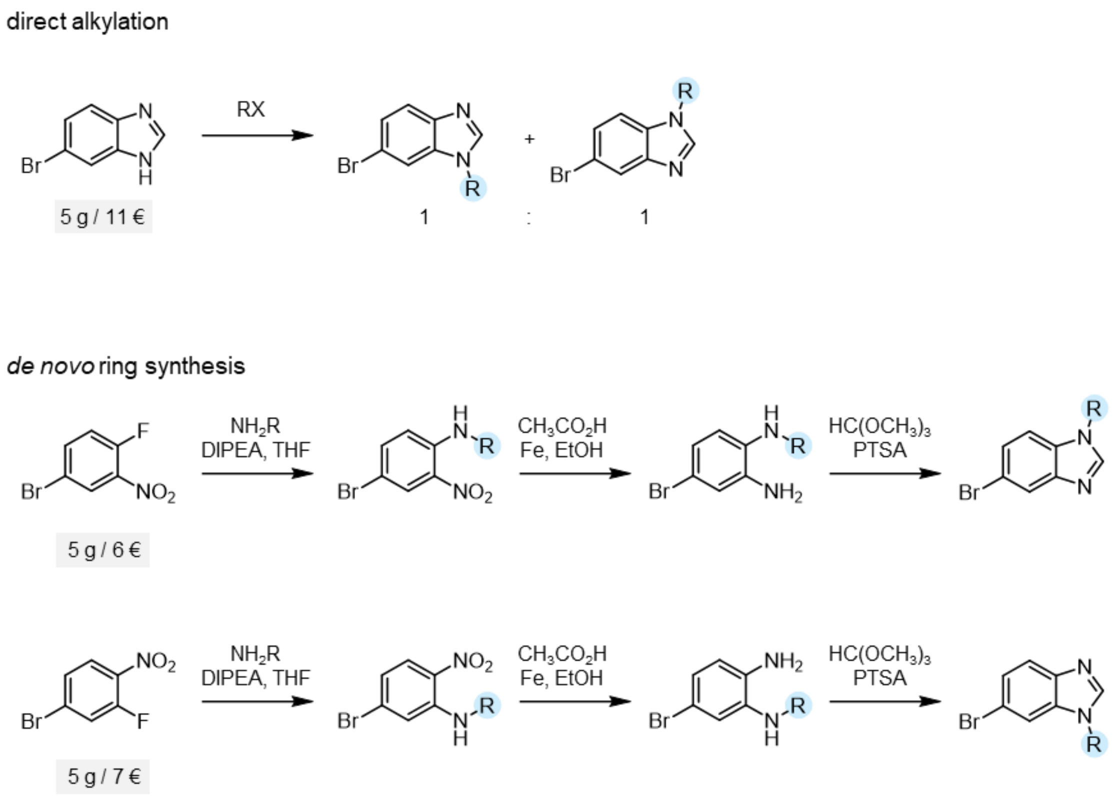


Since the starting materials are readily available at low prices (taken from BLD-pharma), direct alkylation using “off the shelf” alkylating reagents would be a strongly preferred route. However, due to lack of selectivity, the access of *N*-alkylated azoles frequently requires lengthy *de novo* ring synthesis for each regioisomer.[26] The alkylation pattern is frequently set early in the *de novo* ring synthesis which becomes tedious for the creation of a panel of *N*-alkyl analogs.

**Figure S6.** Synthesis of the JAK2 inhibitor BMS-911543, as reported by Fitzgerald *et al.*[27]

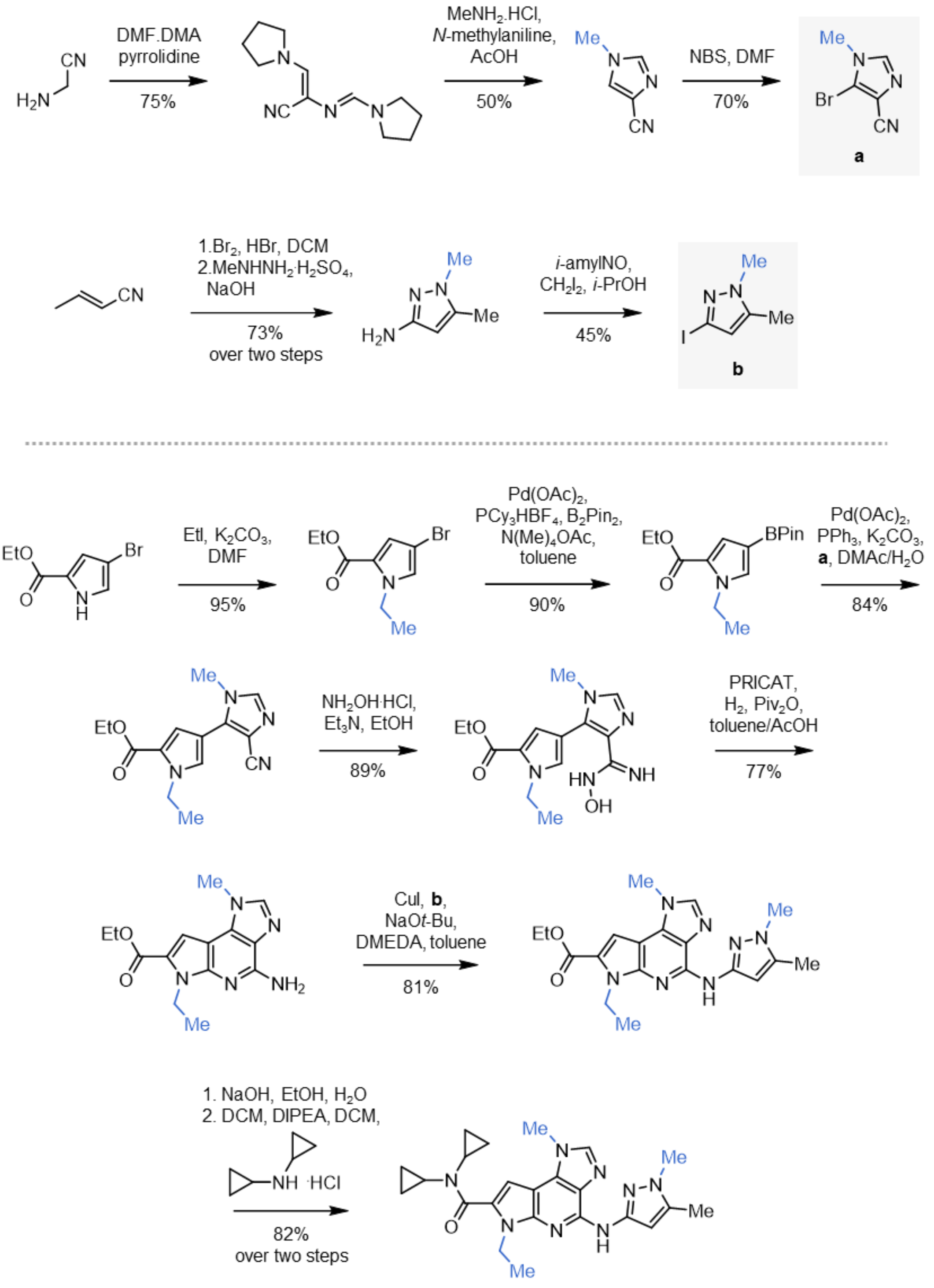


A key Ni-mediated cyclization enabled the pyridine ring formation in this convergent synthetic route. The *N*-alkylation patterns (highlighted in blue) were predefined during the early synthesis in the *de novo* construction of the *N*-alkylated azoles, rendering synthesis of analogs very laborious. This challenge is a common limitation when synthesizing such azole rich compounds.

**Figure S7.** Summary of the SSM libraries of v31 and the strategy for generating the combinatorial mutant library.

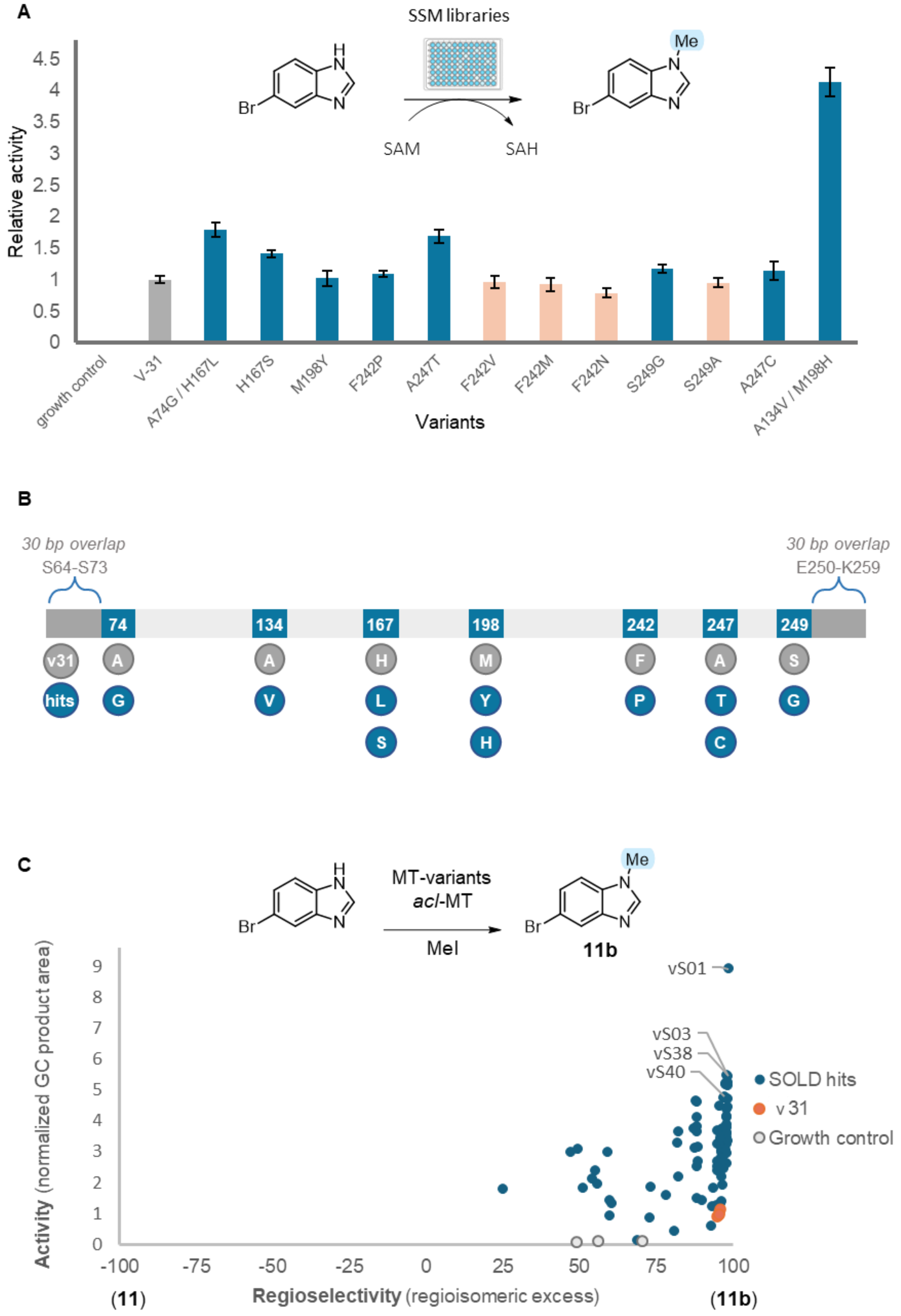

**A)** v31 (*hsa*-N-NMT D167H, A198M, S201C, Y242F, N249S) was selected as starting point to screen for improved variants. A GC-MS method (see section **Q** for details) was employed using 5-bromo-1*H*-benzo[*d*]imidazole as model substrate. SSM was applied (see section **D**) targeting the twelve active-site residues forming the substrate binding pocket of v31 (Y20, Y24, L164, H167, D197, M198, C201, Y204, S213, F242, A247, S249). The figure shows the confirmation of the hits from the SSM experiments using cell-free extracts and SAM as methyl donor. Reactions were made in 96-DWP, and reported values represent the average of 10 replicas for each beneficial mutation (see section **H** for details). The parent enzyme v31 is shown in gray. Mutants are highlighted in blue (higher activity than the parent) and peach color (exhibiting lower activity than the parent). The A74G and A134V mutations emerged as polymerase-induced errors during PCR, leading to the double mutants A134V/M198H and A74G/H167L.

**B)** Representation of the designed combinatorial mutant library. After identifying the key mutations in the best-performing enzyme variants, up to two mutations per amino acid site were selected alongside the original residues from v31. The designed combinatorial library consists of 432 possible variants and was ordered from Twist Bioscience as SOLD (Spread-Out Low diversity) synthetic DNA library. The DNA fragment encoded amino acids 64 to 259 of v31. The DNA regions highlighted in dark-grey have been designed for Gibson Assembly of the synthetic fragments with the backbone of the pET-28a(+) plasmid containing the remaining codons for v31. Targeted amino acid positions for mutagenesis are highlighted in blue boxes and the chosen amino acid combinations are shown directly below inside the gray and blue circles. The designed DNA fragment looks as follows (primer sites are highlighted in light blue and the mutations sites are indicated in red):

```
TCAGGTCCCACAATTTACCAACTTCTGTCCXXXTGTGAGAGTTTTAAGGAGATCGTCGTGACGGACTATTCAGATCAAAATCT
GCAAGAACTGGAGAAATGGTTAAAGAAAGAACCAGAGGCGTTCGATTGGAGCCCAGTTGTGACCTATGTCTGTGATCTTGAAG
GAAACCGCGTAAAGGGTCCTGAAAAGGAGGAAAAGCTGCGCCAAXXXGTAAAGCAAGTATTAAAATGTGATGTTACTCAAAGC
CAACCATTAGGAGCCGTCCCACTGCCCCCGGCGGATTGTGTATTGTCAACCCTTTGCTTGXXXGCAGCTTGTCCTGACTTGCC
TACATATTGTCGTGCATTACGCAACTTAGGAAGTTTGCTTAAACCAGGAGGGTTTCTGGTCATCATGGATXXXTTGAAGTgcT
CCTACTACATGATTGGGGAACAGAAGTTCTCATCTTTACCTTTGGGCCGTGAAGCCGTAGAAGCGGCCGTAAAGGAGGCGGGG
TACACCATTGAATGGTTCGAGGTTATTTCACAGTCCXXXAGCTCCACAATGXXXAATXXXGAGGGCTTATTTTCGCTGGTTGC
TCGCAAA
```

**C)** The library was expressed and screened in 96-DWPs with 5-bromobenzimidazole as substrate using HPLC-DAD (see section **H** for reaction details, see section **O i.** for the HPLC-DAD method). The best performing variants from the screening were collected and sequenced, resulting in 46 unique mutants (vS01 – vS46). The plot shows the activity/selectivity profile from the rescreening of the 46 unique mutants (in comparison to the parent v31) using the cyclic enzyme cascade with 5-bromobenzimidazole as the substrate and MeI as reagent. All the variants exhibited selectivity towards 5-bromo-1-methyl-1*H*-benzo[*d*]imidazole with activities normalized to v31. The variants exhibiting the best combination of activity and selectivity are labeled in the plot.

**Figure S8.** HPLC-DAD analysis for regioselective enzymatic *N*-methylation of functionalized azoles.

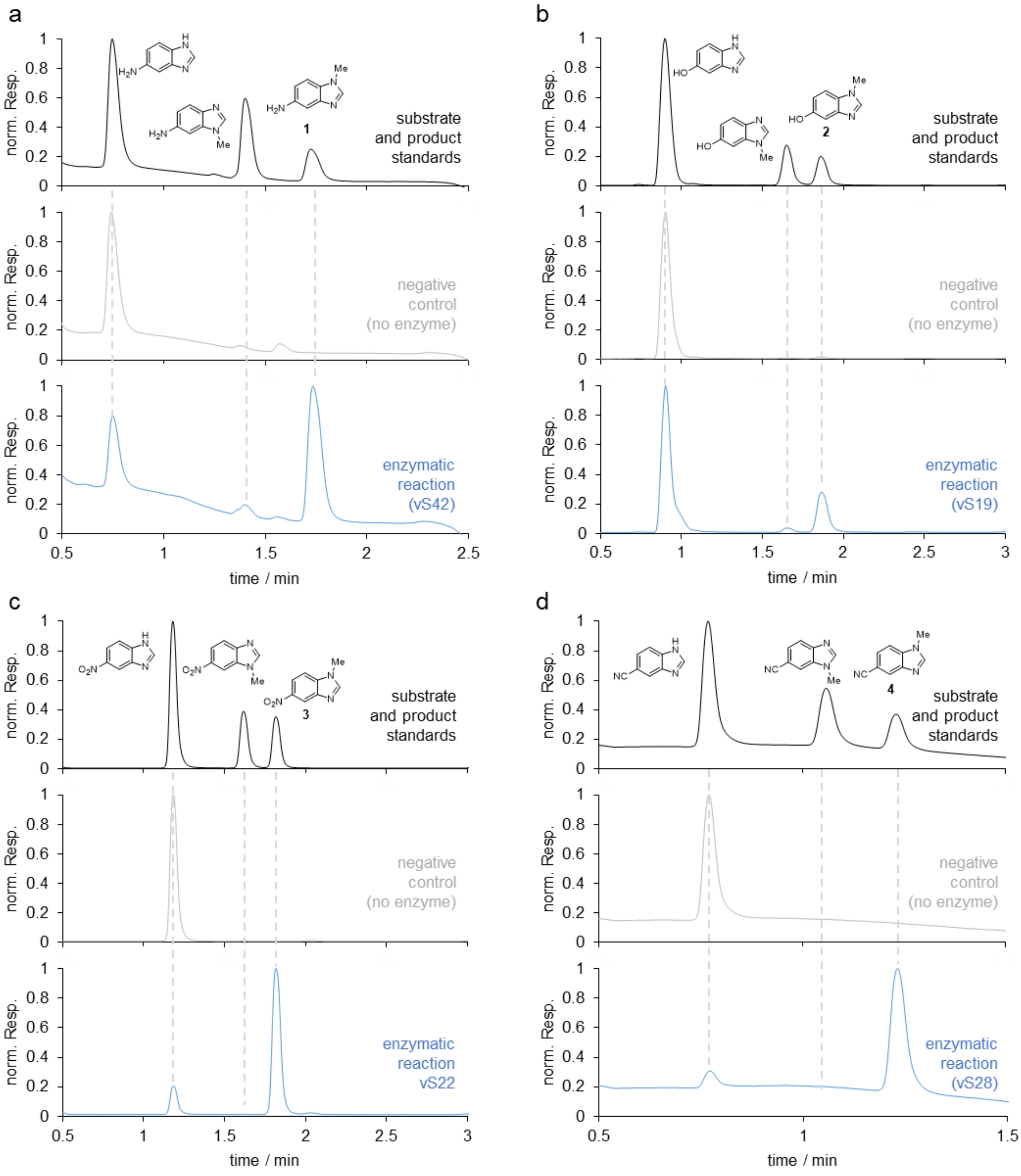


HPLC traces of synthesized product standards (in black), negative controls without enzyme (in grey) and analytical scale reactions (in blue, see section **J**) for the regioselective *N*-methylation of functionalized benzimidazoles employing *hsa*-N-NMT (variant) and *acl*-MT using iodomethane as alkylating agent. Chromatograms are given as normalized response. (a) 1-Methyl-1*H*-benzo[*d*]imidazol-5-amine **1** 94:6 r.r., (b) 1-methyl-1*H*-benzo[*d*]imidazol-5-ol **2** 92:8 r.r., (c) 1-methyl-5-nitro-1*H*-benzo[*d*]imidazole **3** >99:1 r.r., (d) 1-methyl-1*H*-benzo[*d*]imidazole-5-carbonitrile **4** 99:1 r.r. HPLC Methods are described in section **O ii.**

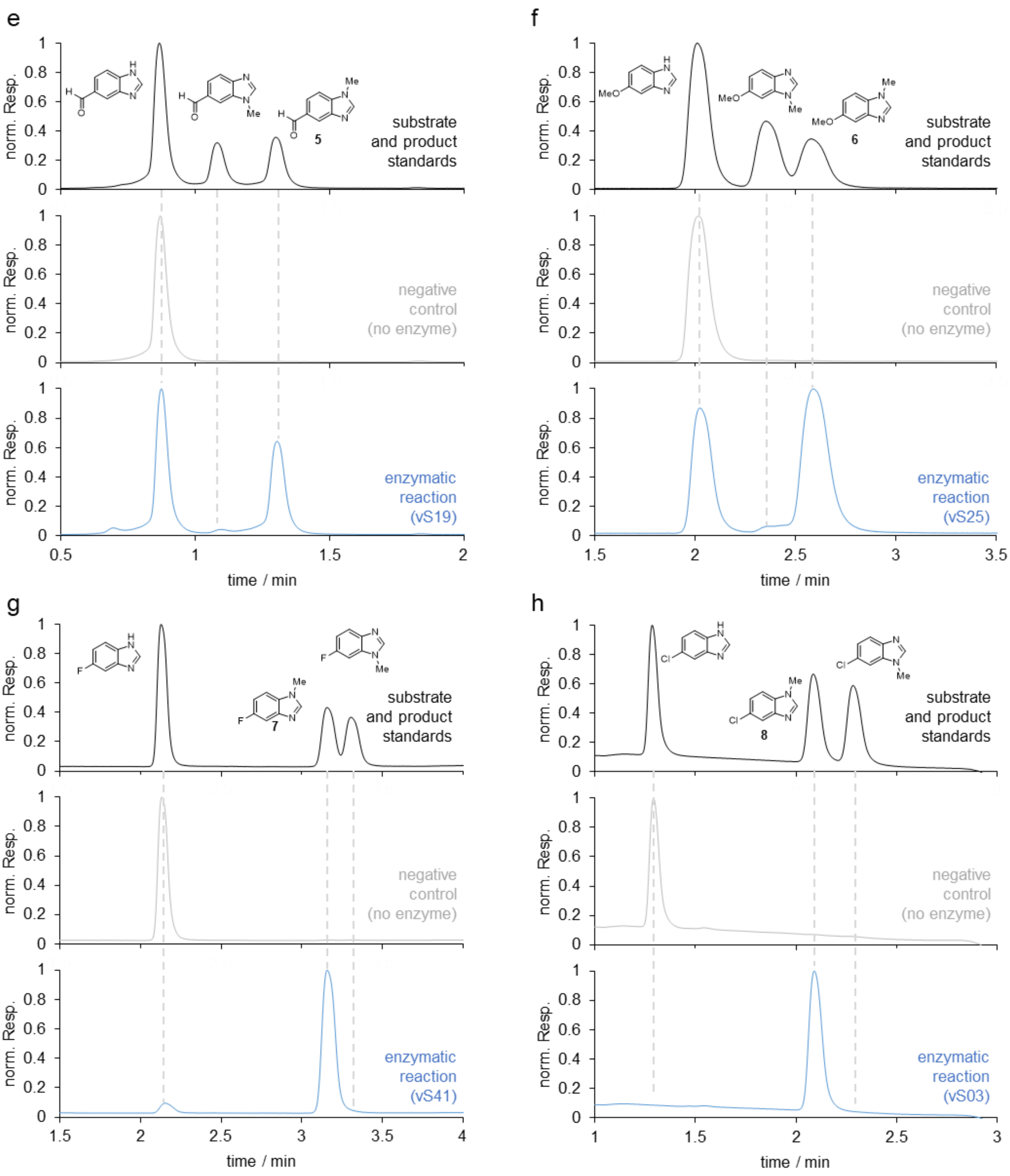


HPLC traces of synthesized product standards (in black), negative controls without enzyme (in grey) and analytical scale reactions (in blue, see section **J**) for the regioselective *N*-methylation of functionalized benzimidazoles employing *hsa*-N-NMT (variant) and *acl*-MT using iodomethane as alkylating agent. Chromatograms are given as normalized response. (e) 1-Methyl-1*H*-benzo[*d*]imidazole-5-carbaldehyde **5** 97:3 r.r., (f) 5-methoxy-1-methyl-1*H*-benzo[*d*]imidazole **6** >97:3 r.r., (g) 5-fluoro-1-methyl-1*H*-benzo[*d*]imidazole **7** >99:1 r.r., (h) 5-chloro-1-methyl-1*H*-benzo[*d*]imidazole **8** >99:1 r.r., HPLC Methods are described in section **O ii.**

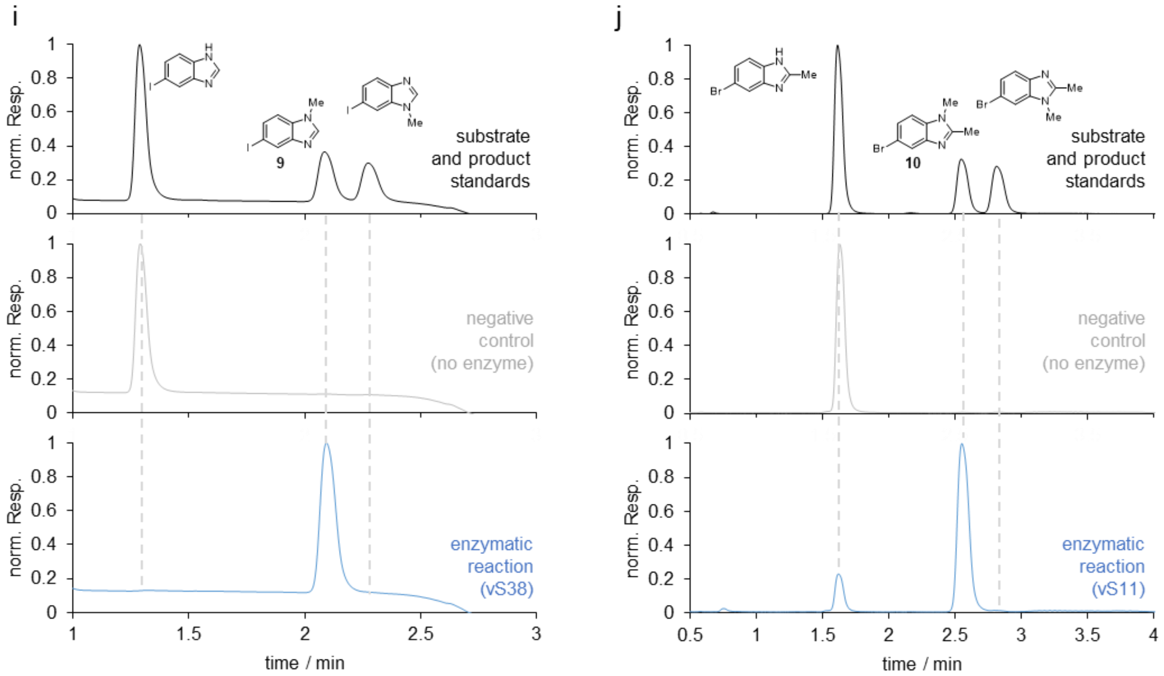


HPLC traces of synthesized product standards (in black), negative controls without enzyme (in grey) and analytical scale reactions (in blue, see section **J**) for the regioselective *N*-methylation of functionalized benzimidazoles employing *hsa*-N-NMT (variant) and *acl*-MT using iodomethane as alkylating agent. Chromatograms are given as normalized response. (i) 5-Iodo-1-methyl-1*H*-benzo[*d*]imidazole **9** >99:1 r.r., (j) 5-bromo-1,2-dimethyl-1*H*-benzo[*d*]imidazole **10** 99:1 r.r., HPLC Methods are described in section **O ii.**

**Figure S9.** Published examples of enzymatic alkylation with off the shelf reagents.

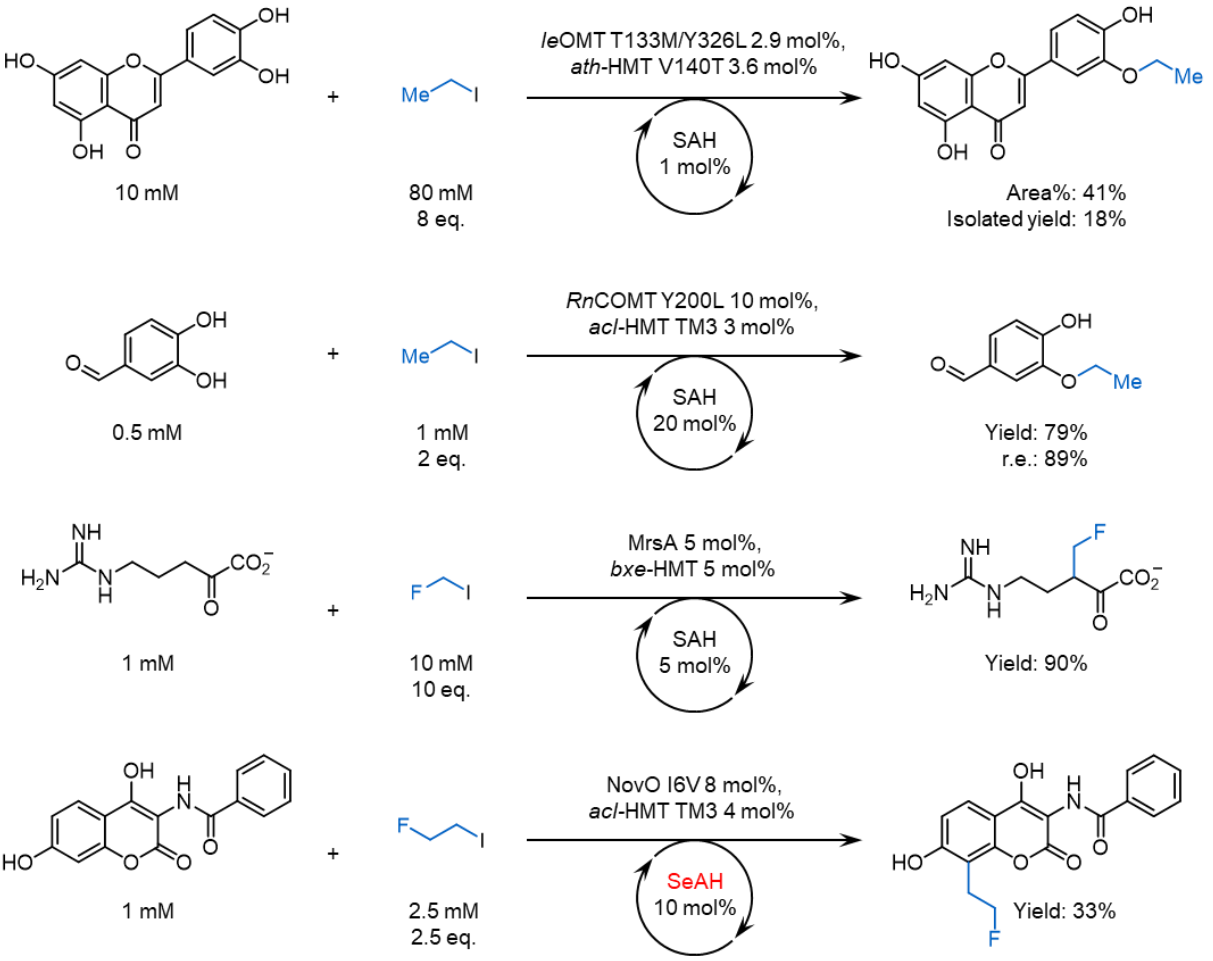


Enzymatic alkylation is currently restricted by several factors, including the reliance on iodoalkanes, limited diversity of alkyl groups, high of SAH and enzyme loadings, and the use of natural products as nucleophiles.[2,28–32] Please note the fluoroethylation depends on the selenium analog of SAH.

**Figure S10.** Identification of the best performing enzyme combinations for azole alkylation.

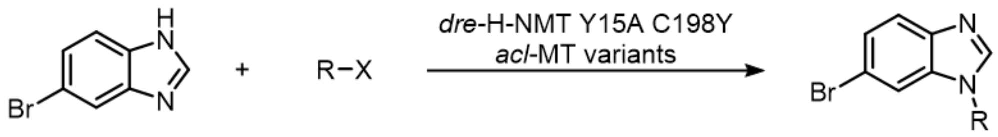


| R-X | *acl*-MT | HPLC Product area 1 mol% | HPLC Product area 10 mol% | R-X | *acl*-MT | HPLC Product area 1 mol% | HPLC Product area 10 mol% | R-X | *acl*-MT | HPLC Product area 1 mol% | HPLC Product area 10 mol% |
|---|---|---|---|---|---|---|---|---|---|---|---|
| Iodoethane | v02 | 79 | 99 | Bromoallyl | v02 | 102 | 95 | 2-bromoacetonitrile | v02 | 8 | 17 |
| | v15 | 92 | 94 | | v15 | 101 | 96 | | v15 | 17 | 13 |
| | v16 | 75 | 96 | | v16 | 100 | 92 | | v16 | 4 | 14 |
| | v31 | 90 | 100 | | v31 | 99 | 93 | | v31 | 7 | 8 |
| | v55 | 64 | 86 | | v55 | 98 | 92 | | v55 | 3 | 9 |
| | v63 | 66 | 81 | | v63 | 99 | 91 | | v63 | 2 | 3 |
| | no *acl*-MT | 0 | 0 | | no *acl*-MT | 0.6 | 1.5 | | no *acl*-MT | 0.1 | 0.0 |
| | no *dre*-H-NMT | 0 | 0 | | no *dre*-H-NMT | 0.5 | 0.5 | | no *dre*-H-NMT | 0.1 | 0.1 |
| Iodomethylcyclopropane | v02 | 74 | 92 | 3-bromo-2-fluoroprop-1-ene | v02 | 64 | 117 | 3-bromoprop-1-yne | v02 | 69 | 57 |
| | v15 | 7 | 8 | | v15 | 44 | 58 | | v15 | 59 | 45 |
| | v16 | 65 | 91 | | v16 | 59 | 113 | | v16 | 73 | 58 |
| | v31 | 43 | 67 | | v31 | 37 | 89 | | v31 | 66 | 48 |
| | v55 | 71 | 91 | | v55 | 54 | 110 | | v55 | 67 | 49 |
| | v63 | 33 | 52 | | v63 | 15 | 39 | | v63 | 31 | 29 |
| | no *acl*-MT | 0 | 0 | | no *acl*-MT | 0 | 0 | | no *acl*-MT | 0.0 | 0.1 |
| | no *dre*-H-NMT | 0 | 0 | | no *dre*-H-NMT | 0 | 0 | | no *dre*-H-NMT | 0.4 | 0.4 |
| Iodomethylcyclobutane | v02 | 4 | 5 | 3-bromo-2-methylprop-1-ene | V02 | 39 | 48 | 3-chloroprop-1-yne | v02 | 7 | 9 |
| | v15 | 1 | 0 | | V15 | 29 | 44 | | v15 | 1 | 0 |
| | v16 | 5 | 4 | | V16 | 41 | 48 | | v16 | 10 | 10 |
| | v31 | 3 | 1 | | V31 | 41 | 49 | | v31 | 4 | 3 |
| | v55 | 5 | 5 | | V55 | 42 | 49 | | v55 | 5 | 4 |
| | v63 | 6 | 5 | | V63 | 29 | 45 | | v63 | 1 | 0 |
| | no *acl*-MT | 0 | 0 | | no *acl*-MT | 0.8 | 1.0 | | no *acl*-MT | 0.4 | 0.3 |
| | no *dre*-H-NMT | 0 | 0 | | no *dre*-H-NMT | 0.5 | 0.5 | | no *dre*-H-NMT | 0.3 | 0.3 |

Single reactions were carried out to identify the identify transferases for efficient alkylation. Further optimization involved the variation of SAH concentration. In most cases the additional supply of SAH didn't yield significantly higher product formation. Reaction conditions: 2 mM 5-bromo-1*H*-benzo[*d*]imidazole, 6 mM iodo-, bromo-, or chloro-alkane, 20 or 200 $\mu$M (1-10 mol%) SAH, 20 $\mu$M (1 mol%) *dre*-H-NMT (Y15A, C198Y), 20 $\mu$M (1 mol%) *acl*-MT variant, r.t., 20 h

**Figure S11.** HPLC and GC traces for regioselective enzymatic *N*-alkylation of azoles.

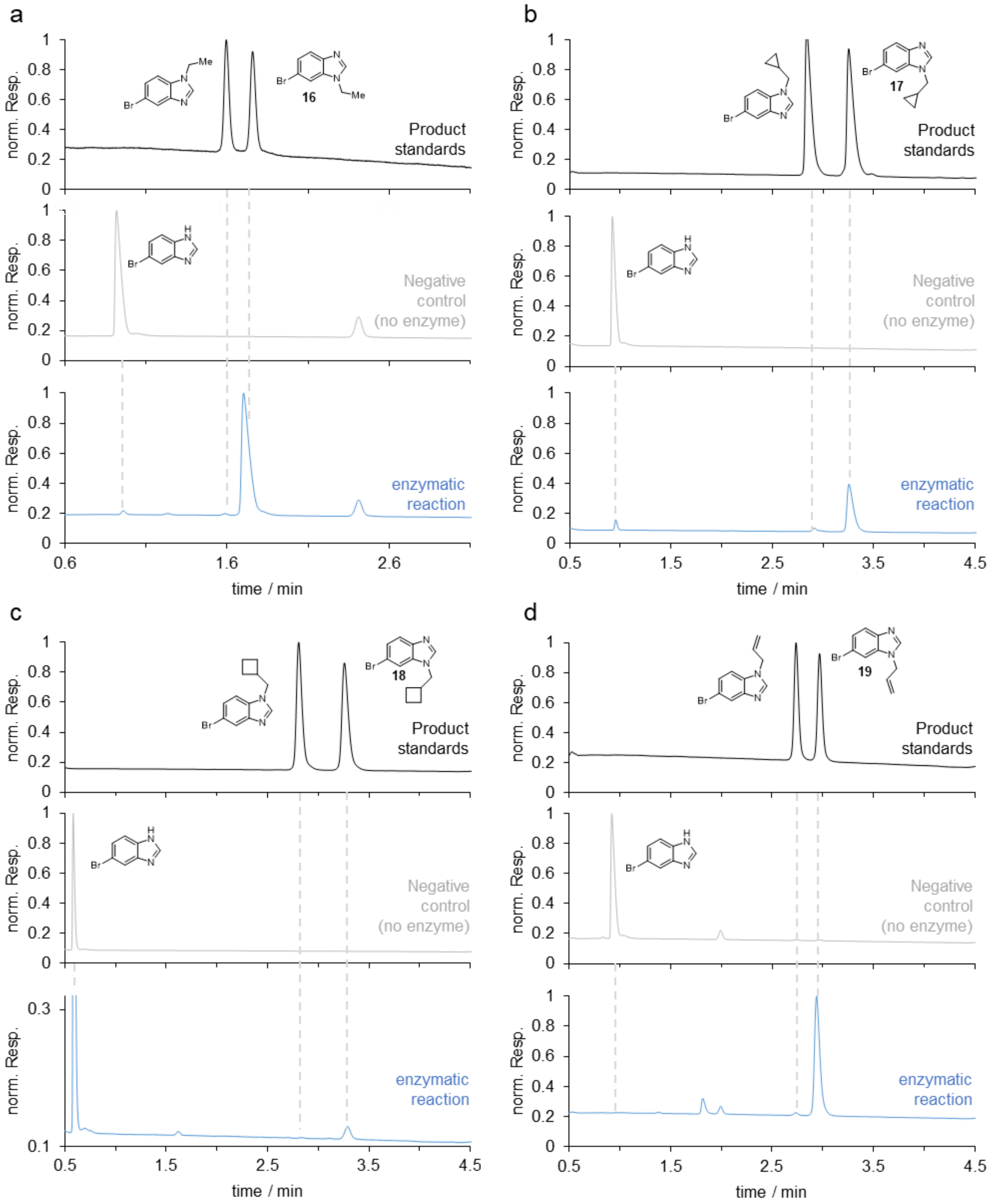

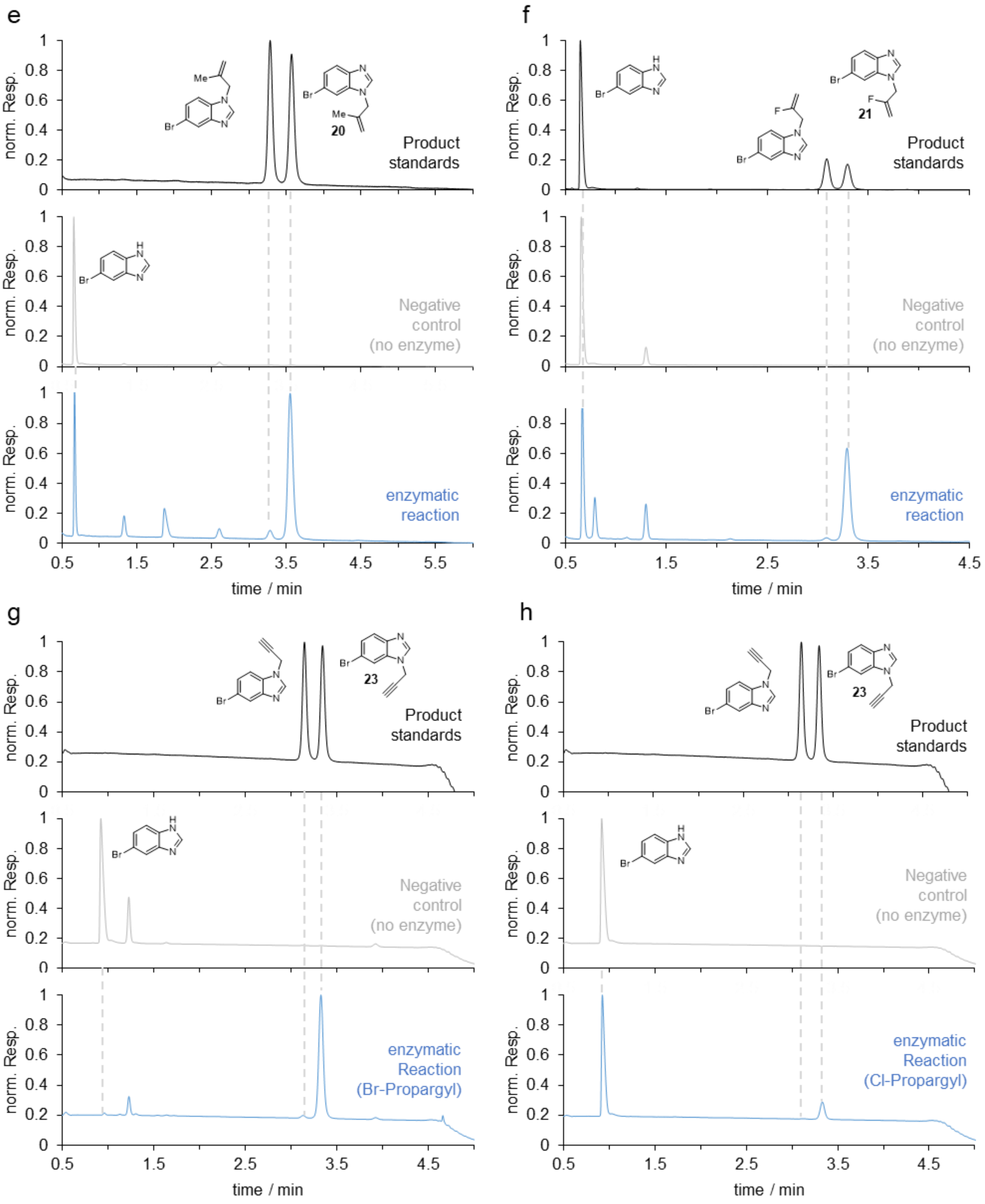


Please note the HPLC DAD chromatograms from the allylation show additional peaks. From quantification of the analytical reactions we know that these are minor unknown side products as recovery is around 100%. Recovery is determined by (measured concentration of substrate + measured concentration of products)/theoretical concentration of the staring material in %.

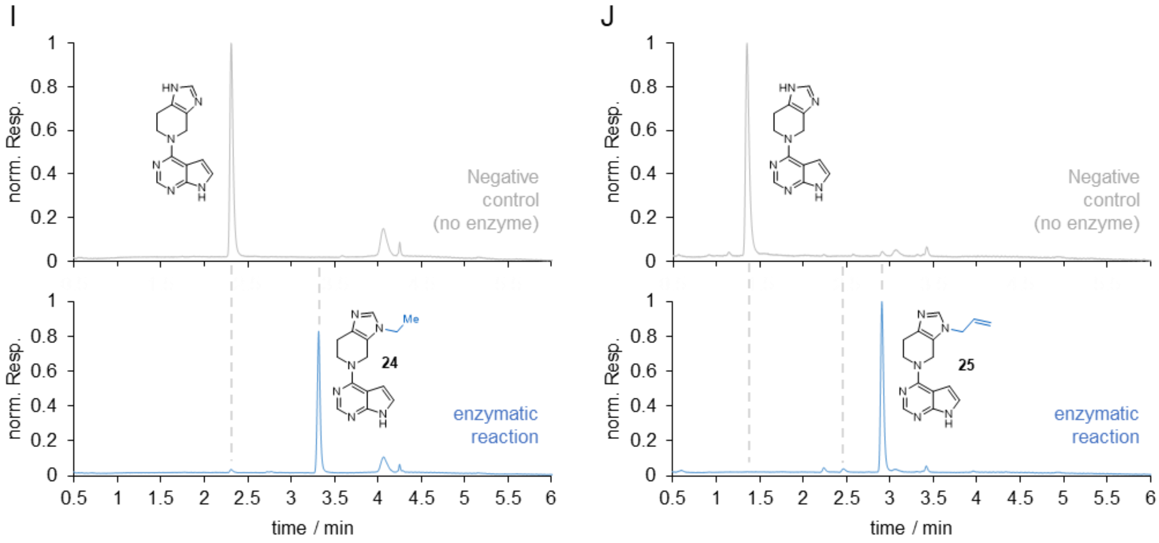


HPLC-DAD traces of synthesized product standards (in black), negative controls without enzyme (in grey) and analytical scale reactions (in blue) for the regioselective alkylation of 5-bromo-1*H*-benzo[*d*]imidazole. Standard reaction condition for 5-bromo-1H-benzo[d]imidazole alkylation: 2 mM 5-bromo-1*H*-benzo[*d*]imidazole, 6 mM iodo-, bromo-, or chloro-alkane, 20 or 200 µM (1-10 mol%) SAH, 20 µM (1 mol%) *dre*-H-NMT, 20 µM (1 mol%) *acl*-MT variant, r.t., 20 h (for reaction conditions: see section **N** for more details, for HPLC methods: see section **O viii** for 5-bromo-1H-benzo[d]imidazole alkylation and section **O v** - **vi** for late state alkylation). (a) **16** with >99:1 r.r., (b) **17** with 97:3 r.r., (c) **18** with >99:1 r.r., (d) **19** with 98:2 r.r., (e) **20** with 95:5 r.r., (f) **21** with 97:3 r.r., (g) **23** with 98:2 r.r., (h) **23** with 98:2 r.r.; (i) **24** with >99% selectivity, (j) **25** with 97% selectivity.

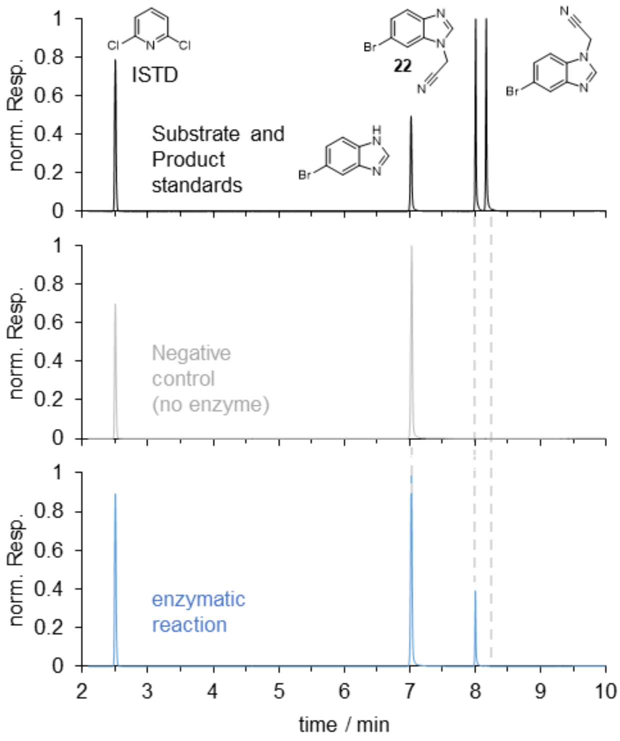


GC/MS traces of synthesized product standards (in black), negative controls without enzyme (in grey) and analytical scale reactions (in blue) for the regioselective functionalization of 5-bromo-1*H*-benzo[*d*]imidazole employing the engineered *dre*-H-NMT (Y15A C198Y) and *acl*-MT using 2-bromoacetonitrile as alkylation agent affording **22** with 99:1 r.r.. For details of the used GC method, see section **Q i.** Chromatograms are given as normalized response, and areas were normalized on the internal standard (2,6-dichloropyridine). Reaction conditions: 2 mM 5-bromo-1*H*-benzo[*d*]imidazole, 6 mM 2-bromoacetonitrile, 20 µM (10 mol%) SAH, 20 µM (1 mol%) *dre*-H-NMT (Y15A, C198Y), 20 µM (1 mol%) *acl*-MT v15, r.t., 20 h.

**Figure S12.** SDS PAGE analysis of the purified enzymes used for the rescreening and upscaling reactions.

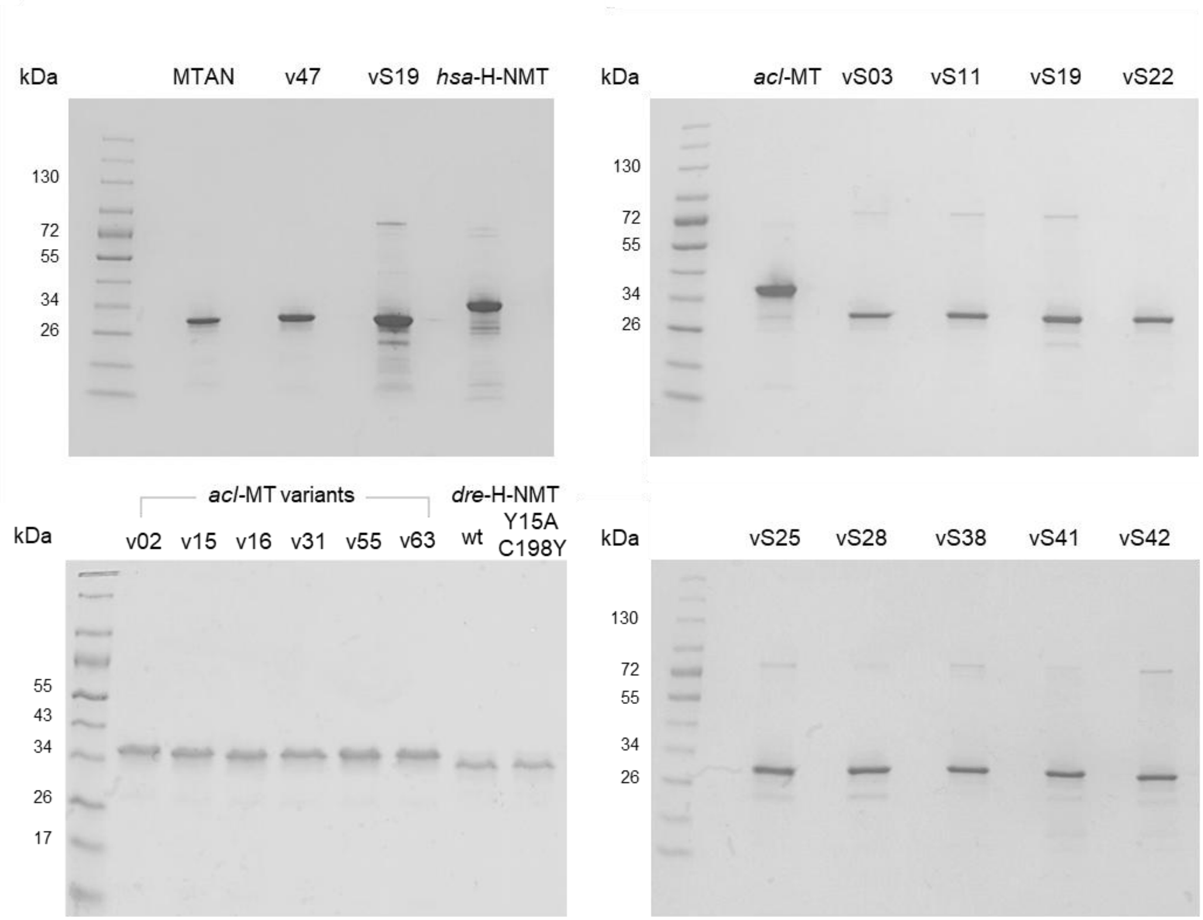


The marker *Color Pre-stained Protein Standard* (Broad Range, 10-250 kDa) was used as the size standard and protein separation was carried out on a pre-cast gel (Mini-Protean TGX gel, 4-20%, 12-well comb) run at 200 V for 30 min.

## III. Supporting tables

**Table S1.** Combinatorial SOLD library containing *hsa*-N-NMT mutants used in the regioselective methylation of functionalized benzimidazoles and the screening for late-stage methylation and regioselective azole alkylation.

| Variant | Introduced mutations (*hsa*-N-NMT) |
|---|---|
| vS01 | A74G, A134V, D167S, A198H, S201C, Y242F, N249S |
| vS02 | A74G, A134V, D167H, A198H, S201C, Y242P, N249S |
| vS03 | A74G, A134V, D167H, A198H, S201C, Y242F, A247T, N249S |
| vS04 | A74G, A134V, D167H, A198Y, S201C, Y242P, A247C, N249G |
| vS05 | A74G, A134V, D167H, A198Y, S201C, Y242P, N249G |
| vS06 | A74G, L154Q, D167H, A198H, S201C, Y242F, N249S |
| vS07 | A74G, D167S, A198H, S201C, Y242F, N249S |
| vS08 | A74G, D167S, A198H, S201C, Y242P, N249S |
| vS09 | A74G, D167S, A198Y, S201C, Y242P, A247C, N249G |
| vS10 | A74G, D167H, A198H, S201C, Y242F, N249S |
| vS11 | A74G, D167H, A198H, S201C, Y242P, N249S |
| vS12 | A74G, D167H, A198H, S201C, Y242P, A247T, N249S |
| vS13 | A74G, D167H, A198H, S201C, Y242F, A247T, N249S |
| vS14 | A74G, D167H, A198Y, S201C, Y242P, A247C, N249G |
| vS15 | A74G, D167H, A198Y, S201C, Y242P, N249G |
| vS16 | V82F, D167S, A198H, S201C, Y242P, N249S |
| vS17 | A134V, D167S, A198H, S201C, Y242F, N249S |
| vS18 | A134V, D167S, A198H, S201C, Y242P, N249S |
| vS19 | A134V, D167S, A198Y, S201C, Y242P, N249G |
| vS20 | A134V, D167H, P171L, A198Y, S201C, Y242F, A247C, N249G |
| vS21 | A134V, D167H, A198H, S201C, Y242F, N249S |
| vS22 | A134V, D167H, A198H, S201C, Y242P, N249S |
| vS23 | A134V, D167H, A198H, S201C, Y242P, A247T, N249S |
| vS24 | A134V, D167H, A198H, S201C, Y242F, A247T, N249S |
| vS25 | A134V, D167H, A198Y, S201C, Y242P, N249S |
| vS26 | A134V, D167H, A198Y, S201C, Y242P, A247C, N249S |
| vS27 | A134V, D167H, A198Y, S201C, Y242P, A247C, N249G |
| vS28 | A134V, D167H, A198Y, S201C, Y242P, N249G |
| vS29 | A134V, D167H, A198M, S201C, Y242P, N249G |
| vS30 | D167L, A198H, S201C, Y242F, A247T, N249S |
| vS31 | D167L, A198Y, S201C, Y242P, N249G |
| vS32 | D167S, A198H, S201C, Y242F, N249S |
| vS33 | D167S, A198H, S201C, Y242P, N249S |
| vS34 | D167S, A198Y, S201C, Y242P, A247C, N249G |
| vS35 | D167S, A198Y, S201C, Y242P, A247T, N249G |
| vS36 | D167S, A198M, S201C, Y242F, A247C, N249S |
| vS37 | D167P, A198M, S201C, Y242F, A247T, N249S |
| vS38 | D167H, A198H, S201C, Y242F, N249S |
| vS39 | D167H, A198H, S201C, Y242P, N249S |
| vS40 | D167H, A198H, S201C, Y242P, A247T, N249S |
| vS41 | D167H, A198H, S201C, Y242F, A247T, N249S |
| vS42 | D167H, A198Y, S201C, Y242P, N249S |
| vS43 | D167H, A198Y, S201C, Y242P, A247C, N249G |
| vS44 | D167H, A198Y, S201C, Y242P, A247T, N249G |
| vS45 | D167H, A198Y, S201C, Y242P, N249G |
| vS46 | A74G, A134V, D167H, A198H, S201C, Y242F, N249S |

**Table S2.** Panel of wild type methyltransferases used in the screening for late-stage methylation and regioselective azole alkylation.

| Wild type | Enzyme | Organism | UniProt Accession Number |
|---|---|---|---|
| 1 | Coniferyl alcohol 9-*O*-methyltransferase | *Linum nodiflorum* | A6XNE6 |
| 2 | *S*-methyltransferase | *Catharanthus roseus* | Q1WMA5 |
| 3 | Phenazine-1-carboxylate *N*-methyltransferase | *Pseudomonas aeruginosa* | Q9HWH2 |
| 4 | Bergaptol *O*-methyltransferase | *Kitagawia praeruptora* | A0A166U5H3 |
| 5 | Phenylpyruvate *C*(3)-methyltransferase | *Streptomyces hygroscopicus* | Q643C8 |
| 6 | Putative *O*-methyltransferase | *Saccharomonospora glauca* K62 | I1D222 |
| 7 | 3-aminomethylindole *N*-methyltransferase | *Hordeum vulgare subsp. vulgare* | Q96565 |
| 8 | Nicotinate *N*-methyltransferase 1 | *Arabidopsis thaliana* | Q9SCP7 |
| 9 | Nicotinate *N*-methyltransferase 1 | *Oryza sativa subsp. japonica* | Q6K9X3 |
| 10 | Nicotinate *N*-methyltransferase 1 | *Glycine max* | I1M2U5 |
| 11 | (+)-*O*-methylkolavelool synthase | *Herpetosiphon aurantiacus* | A9AWD7 |
| 12 | Methyltransferase | *Burkholderia glumae* | Q65YP0 |
| 13 | Histamine *N*-methyltransferase | *Danio rerio* | Q6DC37 |
| 14 | Histamine *N*-methyltransferase | *Xenopus tropicalis* | Q0V9P1 |
| 15 | Histamine *N*-methyltransferase | *Mus musculus* | Q91VF2 |
| 16 | Histamine *N*-methyltransferase | *Bos taurus* | Q58DV7 |
| 17 | Histamine *N*-methyltransferase | *Cavia porcellus* | Q9EST2 |
| 18 | Histamine *N*-methyltransferase A | *Xenopus laevis* | Q5U4V2 |
| 19 | Histamine *N*-methyltransferase | *Tetraodon nigroviridis* | Q4SBY6 |
| 20 | Histamine *N*-methyltransferase | *Tursiops truncatus* | A0A2U4AJA4 |
| 21 | Nicotinamide *N*-methyltransferase | *Mus musculus* | O55239 |
| 22 | Nicotinamide *N*-methyltransferase | *Caenorhabditis elegans* | P34254 |
| 23 | Phenylethanolamine *N*-methyltransferase | *Mus musculus* | P40935 |
| 24 | Phenylethanolamine *N*-methyltransferase | *Bos taurus* | P10938 |
| 25 | Indolethylamine *N*-methyltransferase | *Homo sapiens* | O95050 |
| 26 | Indolethylamine *N*-methyltransferase | *Mus musculus* | P40936 |
| 27 | Methyltransferase | *Actinomadura sp.* KC06 | A0A4R4Y3T3 |
| 28 | Methyltransferase | *Streptomyces sp.* NRRL WC-3605 | A0A0X3XHI7 |
| 29 | Methyltransferase | *Streptomyces varsoviensis* | A0A0L8QTT5 |
| 30 | Methyltransferase | *Ranitomeya imitator* | A0A821K491 |
| 31 | Nicotinamide *N*-methyltransferase, putative | *Ixodes scapularis* | B7Q5F2 |
| 32 | Uncharacterized protein | *Branchiostoma floridae* | C3Z5R9 |
| 33 | Methyltransferase | *Streptomyces afghaniensis* 772 | S4N1R9 |

| | | | |
|---|---|---|---|
| 34 | *N*-methyltransferase | *Streptomyces sviceus* | B5I9B3 |
| 35 | Methyltransferase, putative | *Xenopus tropicalis* | A9JTQ8 |
| 36 | Methyltransferase, putative | *Rotaria magnacalcarata* | A0A816AWZ6 |
| 37 | *N*-methyltransferase | *Dictyocaulus viviparus* | Q53CN2 |
| 38 | NNMT/PNMT/TEMT family protein | *Necator americanus* | W2TA20 |
| 39 | Methyltransferase, putative | *Cavia porcellus* | H0W3I3 |
| 40 | Phenylethanolamine *N*-methyltransferase | *Myotis lucifugus* | G1PR98 |
| 41 | NNMT/PNMT/TEMT family protein | *Pleurocapsa sp.* PCC 7327 | K9T4R2 |
| 42 | Phenylethanolamine *N*-methyltransferase | *Petromyzon marinus* | S4RYD2 |
| 43 | Phenylethanolamine *N*-methyltransferase | *Anolis carolinensis* | H9G6B1 |
| 44 | Nicotinamide N-methyltransferase | *Pteropus alecto* | L5KJT9 |
| 45 | Phenylethanolamine *N*-methyltransferase | *Ailuropoda melanoleuca* | D2I2G0 |
| 46 | Phenylethanolamine *N*-methyltransferase | *Callithrix jacchus* | F6ZPQ7 |
| 47 | Histamine *N*-methyltransferase | *Homo Sapiens* | P50135 |
| 48 | Indolethylamine *N*-methyltransferase | *Oryctolagus cuniculus* | O97972 |
| 49 | Nicotinamide *N*-methyltransferase | *Homo Sapiens* | P40261 |
| 50 | Phenylethanolamine *N*-methyltransferase | *Homo Sapiens* | P11086 |

**Table S3.** Panel of previously reported *acl*-MT wild type and promiscuous variants used for regioselective azole alkylation.[3]

| Variant | Introduced mutations (*acl*-MT) |
|---|---|
| *acl*-MT | wild type (uniport: A1CIS5) |
| *acl*-MT v02 | V11F, L30I, L39D, W41F |
| *acl*-MT v15 | V11F, L30M, W41F |
| *acl*-MT v16 | V11F, L30I, L39D |
| *acl*-MT v31 | V11F, L30I, L39H |
| *acl*-MT v55 | V11F, L39D |
| *acl*-MT v63 | L30M, L39H |

**Table S4.** Screening results for the methylation of 1*H*-benzo[*d*]imidazol-5-amine using the combinatorial SOLD library.

| Name | Mutations based on *hsa*-N-NMT | relative activity | selectivity (r.e.) |
|---|---|---|---|
| Empy vector | | 0.13 | n.d. |
| Empy vector | | 0.19 | n.d. |
| Empy vector | | 0.19 | n.d. |
| Empy vector | | 0.18 | n.d. |
| Growth control | | 0.02 | n.d. |
| Growth control | | 0.03 | n.d. |
| Growth control | | 0.02 | n.d. |
| Growth control | | 0.01 | n.d. |
| *hsa-N-NMT* | wild type | 1.08 | -55.43 |
| *hsa-N-NMT* | wild type | 0.92 | -63.37 |
| v31 | D167H, A198M, S201C, Y242F, N249S | 3.37 | 76.56 |
| v31 | D167H, A198M, S201C, Y242F, N249S | 3.10 | 93.59 |
| v31 | D167H, A198M, S201C, Y242F, N249S | 3.09 | 88.92 |
| vS01 | A74G, A134V, D167S, A198H, S201C, Y242F, N249S | 9.84 | 66.70 |
| vS02 | A74G, A134V, D167H, A198H, S201C, Y242P, N249S | 9.12 | 93.63 |
| vS03 | A74G, A134V, D167H, A198H, S201C, Y242F, A247T, N249S | 11.87 | 89.16 |
| vS04 | A74G, A134V, D167H, A198Y, S201C, Y242P, A247C, N249G | 16.26 | 64.56 |
| vS05 | A74G, A134V, D167H, A198Y, S201C, Y242P, N249G | 25.47 | 79.03 |
| vS06 | A74G, L154Q, D167H, A198H, S201C, Y242F, N249S | 5.42 | 89.48 |
| vS07 | A74G, D167S, A198H, S201C, Y242F, N249S | 7.20 | 75.16 |
| vS08 | A74G, D167S, A198H, S201C, Y242P, N249S | 10.38 | 84.35 |
| vS09 | A74G, D167S, A198Y, S201C, Y242P, A247C, N249G | 20.73 | 84.43 |
| vS10 | A74G, D167H, A198H, S201C, Y242F, N249S | 2.16 | 95.61 |
| vS11 | A74G, D167H, A198H, S201C, Y242P, N249S | 10.20 | 89.14 |
| vS12 | A74G, D167H, A198H, S201C, Y242P, A247T, N249S | 5.87 | 83.97 |
| vS13 | A74G, D167H, A198H, S201C, Y242F, A247T, N249S | 10.38 | 90.80 |
| vS14 | A74G, D167H, A198Y, S201C, Y242P, A247C, N249G | 26.00 | 65.61 |
| vS15 | A74G, D167H, A198Y, S201C, Y242P, N249G | 26.83 | 79.45 |
| vS16 | V82F, D167S, A198H, S201C, Y242P, N249S | 1.87 | 76.29 |
| vS17 | A134V, D167S, A198H, S201C, Y242F, N249S | 5.85 | 64.15 |
| vS18 | A134V, D167S, A198H, S201C, Y242P, N249S | 8.86 | 79.73 |
| vS19 | A134V, D167S, A198Y, S201C, Y242P, N249G | 21.06 | 83.89 |
| vS20 | A134V, D167H, P171L, A198Y, S201C, Y242F, A247C, N249G | 10.78 | 62.74 |
| vS21 | A134V, D167H, A198H, S201C, Y242F, N249S | 7.94 | 86.57 |
| vS22 | A134V, D167H, A198H, S201C, Y242P, N249S | 13.48 | 88.46 |
| vS23 | A134V, D167H, A198H, S201C, Y242P, A247T, N249S | 5.61 | 82.01 |
| vS24 | A134V, D167H, A198H, S201C, Y242F, A247T, N249S | 10.33 | 92.04 |
| vS25 | A134V, D167H, A198Y, S201C, Y242P, N249S | 30.61 | 91.93 |
| vS26 | A134V, D167H, A198Y, S201C, Y242P, A247C, N249S | 15.55 | 85.88 |
| vS27 | A134V, D167H, A198Y, S201C, Y242P, A247C, N249G | 20.02 | 68.14 |
| vS28 | A134V, D167H, A198Y, S201C, Y242P, N249G | 32.34 | 79.38 |
| vS29 | A134V, D167H, A198M, S201C, Y242P, N249G | 14.43 | 67.89 |
| vS30 | D167L, A198H, S201C, Y242F, A247T, N249S | 17.65 | 52.45 |
| vS31 | D167L, A198Y, S201C, Y242P, N249G | 15.76 | 42.50 |
| vS32 | D167S, A198H, S201C, Y242F, N249S | 7.54 | 67.85 |
| vS33 | D167S, A198H, S201C, Y242P, N249S | 9.96 | 79.66 |
| vS34 | D167S, A198Y, S201C, Y242P, A247C, N249G | 20.68 | 87.74 |
| vS35 | D167S, A198Y, S201C, Y242P, A247T, N249G | 13.47 | 78.52 |
| vS36 | D167S, A198M, S201C, Y242F, A247C, N249S | 6.88 | 72.30 |
| vS37 | D167P, A198M, S201C, Y242F, A247T, N249S | 2.31 | -99.67 |
| vS38 | D167H, A198H, S201C, Y242F, N249S | 7.69 | 86.30 |
| vS39 | D167H, A198H, S201C, Y242P, N249S | 9.86 | 91.10 |
| vS40 | D167H, A198H, S201C, Y242P, A247T, N249S | 2.73 | 87.72 |
| vS41 | D167H, A198H, S201C, Y242F, A247T, N249S | 12.36 | 93.49 |
| vS42 | D167H, A198Y, S201C, Y242P, N249S | 28.88 | 93.76 |
| vS43 | D167H, A198Y, S201C, Y242P, A247C, N249G | 26.86 | 67.01 |
| vS44 | D167H, A198Y, S201C, Y242P, A247T, N249G | 21.50 | 73.34 |
| vS45 | D167H, A198Y, S201C, Y242P, N249G | 31.57 | 79.91 |
| vS46 | A74G, A134V, D167H, A198H, S201C, Y242F, N249S | 2.81 | 87.44 |

**Table S5.** Screening results for the methylation of 1*H*-benzo[*d*]imidazol-5-ol using the combinatorial SOLD library.

| Name | Mutations based on *hsa*-N-NMT | relative activity | selectivity (r.e.) |
|---|---|---|---|
| Empy vector | | 0.01 | n.d. |
| Empy vector | | 0.02 | n.d. |
| Empy vector | | 0.01 | n.d. |
| Empy vector | | 0.01 | n.d. |
| Growth control | | 0.00 | n.d. |
| Growth control | | 0.01 | n.d. |
| Growth control | | 0.00 | n.d. |
| Growth control | | 0.00 | n.d. |
| *hsa-N-NMT* | wild type | 1.02 | 80.92 |
| *hsa-N-NMT* | wild type | 0.98 | 80.19 |
| v31 | D167H, A198M, S201C, Y242F, N249S | 1.59 | 70.10 |
| v31 | D167H, A198M, S201C, Y242F, N249S | 1.32 | 70.40 |
| v31 | D167H, A198M, S201C, Y242F, N249S | 1.24 | 69.99 |
| vS01 | A74G, A134V, D167S, A198H, S201C, Y242F, N249S | 1.57 | 69.56 |
| vS02 | A74G, A134V, D167H, A198H, S201C, Y242P, N249S | 2.50 | 51.03 |
| vS03 | A74G, A134V, D167H, A198H, S201C, Y242F, A247T, N249S | 1.42 | 17.19 |
| vS04 | A74G, A134V, D167H, A198Y, S201C, Y242P, A247C, N249G | 2.61 | -51.20 |
| vS05 | A74G, A134V, D167H, A198Y, S201C, Y242P, N249G | 3.90 | 68.02 |
| vS06 | A74G, L154Q, D167H, A198H, S201C, Y242F, N249S | 1.29 | 35.12 |
| vS07 | A74G, D167S, A198H, S201C, Y242F, N249S | 1.30 | 70.30 |
| vS08 | A74G, D167S, A198H, S201C, Y242P, N249S | 1.88 | 81.12 |
| vS09 | A74G, D167S, A198Y, S201C, Y242P, A247C, N249G | 2.72 | 72.56 |
| vS10 | A74G, D167H, A198H, S201C, Y242F, N249S | 0.56 | 37.97 |
| vS11 | A74G, D167H, A198H, S201C, Y242P, N249S | 2.16 | 50.21 |
| vS12 | A74G, D167H, A198H, S201C, Y242P, A247T, N249S | 1.04 | 12.60 |
| vS13 | A74G, D167H, A198H, S201C, Y242F, A247T, N249S | 1.12 | 19.03 |
| vS14 | A74G, D167H, A198Y, S201C, Y242P, A247C, N249G | 2.63 | -50.17 |
| vS15 | A74G, D167H, A198Y, S201C, Y242P, N249G | 2.94 | 69.28 |
| vS16 | V82F, D167S, A198H, S201C, Y242P, N249S | 0.30 | 88.92 |
| vS17 | A134V, D167S, A198H, S201C, Y242F, N249S | 0.92 | 72.35 |
| vS18 | A134V, D167S, A198H, S201C, Y242P, N249S | 1.70 | 82.35 |
| vS19 | A134V, D167S, A198Y, S201C, Y242P, N249G | 4.07 | 90.00 |
| vS20 | A134V, D167H, P171L, A198Y, S201C, Y242F, A247C, N249G | 2.04 | -60.86 |
| vS21 | A134V, D167H, A198H, S201C, Y242F, N249S | 1.84 | 31.43 |
| vS22 | A134V, D167H, A198H, S201C, Y242P, N249S | 2.75 | 49.76 |
| vS23 | A134V, D167H, A198H, S201C, Y242P, A247T, N249S | 1.10 | 16.30 |
| vS24 | A134V, D167H, A198H, S201C, Y242F, A247T, N249S | 1.22 | 18.84 |
| vS25 | A134V, D167H, A198Y, S201C, Y242P, N249S | 4.67 | 73.89 |
| vS26 | A134V, D167H, A198Y, S201C, Y242P, A247C, N249S | 2.41 | -0.11 |
| vS27 | A134V, D167H, A198Y, S201C, Y242P, A247C, N249G | 3.70 | -62.11 |
| vS28 | A134V, D167H, A198Y, S201C, Y242P, N249G | 4.67 | 62.45 |
| vS29 | A134V, D167H, A198M, S201C, Y242P, N249G | 3.38 | 88.18 |
| vS30 | D167L, A198H, S201C, Y242F, A247T, N249S | 2.42 | -21.20 |
| vS31 | D167L, A198Y, S201C, Y242P, N249G | 3.03 | -7.02 |
| vS32 | D167S, A198H, S201C, Y242F, N249S | 1.18 | 71.65 |
| vS33 | D167S, A198H, S201C, Y242P, N249S | 1.57 | 81.61 |
| vS34 | D167S, A198Y, S201C, Y242P, A247C, N249G | 2.71 | 73.73 |
| vS35 | D167S, A198Y, S201C, Y242P, A247T, N249G | 2.18 | 77.13 |
| vS36 | D167S, A198M, S201C, Y242F, A247C, N249S | 0.52 | 80.99 |
| vS37 | D167P, A198M, S201C, Y242F, A247T, N249S | 0.09 | -39.91 |
| vS38 | D167H, A198H, S201C, Y242F, N249S | 1.68 | 32.42 |
| vS39 | D167H, A198H, S201C, Y242P, N249S | 2.28 | 49.88 |
| vS40 | D167H, A198H, S201C, Y242P, A247T, N249S | 0.52 | 22.81 |
| vS41 | D167H, A198H, S201C, Y242F, A247T, N249S | 1.42 | 18.99 |
| vS42 | D167H, A198Y, S201C, Y242P, N249S | 4.90 | 73.72 |
| vS43 | D167H, A198Y, S201C, Y242P, A247C, N249G | 3.18 | -58.41 |
| vS44 | D167H, A198Y, S201C, Y242P, A247T, N249G | 3.02 | 16.62 |
| vS45 | D167H, A198Y, S201C, Y242P, N249G | 3.65 | 64.09 |
| vS46 | A74G, A134V, D167H, A198H, S201C, Y242F, N249S | 0.55 | 79.04 |

**Table S6.** Screening results for the methylation of 5-nitro-1*H*-benzo[*d*]imidazole using the combinatorial SOLD library.

| Name | Mutations based on *hsa*-N-NMT | relative activity | selectivity (r.e.) |
|---|---|---|---|
| Empy vector | | 0.05 | n.d. |
| Empy vector | | 0.03 | n.d. |
| *hsa-H-NMT* | wild type | 13.34 | 99.49 |
| Growth control | | 0.05 | n.d. |
| Growth control | | 0.09 | n.d. |
| Growth control | | 0.01 | n.d. |
| *hsa-N-NMT* | wild type | 1.09 | 46.52 |
| *hsa-N-NMT* | wild type | 0.91 | 49.98 |
| v31 | D167H, A198M, S201C, Y242F, N249S | 15.37 | 97.55 |
| v31 | D167H, A198M, S201C, Y242F, N249S | 14.60 | 97.49 |
| v31 | D167H, A198M, S201C, Y242F, N249S | 14.33 | 97.40 |
| vS01 | A74G, A134V, D167S, A198H, S201C, Y242F, N249S | 60.51 | 99.47 |
| vS02 | A74G, A134V, D167H, A198H, S201C, Y242P, N249S | 87.51 | 99.90 |
| vS03 | A74G, A134V, D167H, A198H, S201C, Y242F, A247T, N249S | 57.09 | 98.62 |
| vS04 | A74G, A134V, D167H, A198Y, S201C, Y242P, A247C, N249G | 21.40 | 70.72 |
| vS05 | A74G, A134V, D167H, A198Y, S201C, Y242P, N249G | 77.81 | 97.83 |
| vS06 | A74G, L154Q, D167H, A198H, S201C, Y242F, N249S | 54.61 | 99.78 |
| vS07 | A74G, D167S, A198H, S201C, Y242F, N249S | 57.71 | 99.15 |
| vS08 | A74G, D167S, A198H, S201C, Y242P, N249S | 68.69 | 99.71 |
| vS09 | A74G, D167S, A198Y, S201C, Y242P, A247C, N249G | 25.54 | 69.44 |
| vS10 | A74G, D167H, A198H, S201C, Y242F, N249S | 29.49 | 99.77 |
| vS11 | A74G, D167H, A198H, S201C, Y242P, N249S | 82.64 | 99.93 |
| vS12 | A74G, D167H, A198H, S201C, Y242P, A247T, N249S | 26.01 | 98.70 |
| vS13 | A74G, D167H, A198H, S201C, Y242F, A247T, N249S | 50.99 | 98.90 |
| vS14 | A74G, D167H, A198Y, S201C, Y242P, A247C, N249G | 26.53 | 71.60 |
| vS15 | A74G, D167H, A198Y, S201C, Y242P, N249G | 81.17 | 97.84 |
| vS16 | V82F, D167S, A198H, S201C, Y242P, N249S | 47.95 | 99.77 |
| vS17 | A134V, D167S, A198H, S201C, Y242F, N249S | 43.53 | 99.30 |
| vS18 | A134V, D167S, A198H, S201C, Y242P, N249S | 57.87 | 99.65 |
| vS19 | A134V, D167S, A198Y, S201C, Y242P, N249G | 69.17 | 97.02 |
| vS20 | A134V, D167H, P171L, A198Y, S201C, Y242F, A247C, N249G | 9.44 | 46.47 |
| vS21 | A134V, D167H, A198H, S201C, Y242F, N249S | 65.89 | 99.72 |
| vS22 | A134V, D167H, A198H, S201C, Y242P, N249S | 90.01 | 99.93 |
| vS23 | A134V, D167H, A198H, S201C, Y242P, A247T, N249S | 23.44 | 98.79 |
| vS24 | A134V, D167H, A198H, S201C, Y242F, A247T, N249S | 47.68 | 98.98 |
| vS25 | A134V, D167H, A198Y, S201C, Y242P, N249S | 53.00 | 99.15 |
| vS26 | A134V, D167H, A198Y, S201C, Y242P, A247C, N249S | 29.30 | 95.04 |
| vS27 | A134V, D167H, A198Y, S201C, Y242P, A247C, N249G | 26.80 | 72.80 |
| vS28 | A134V, D167H, A198Y, S201C, Y242P, N249G | 80.06 | 97.91 |
| vS29 | A134V, D167H, A198M, S201C, Y242P, N249G | 70.09 | 98.20 |
| vS30 | D167L, A198H, S201C, Y242F, A247T, N249S | 56.25 | 95.53 |
| vS31 | D167L, A198Y, S201C, Y242P, N249G | 65.71 | 96.27 |
| vS32 | D167S, A198H, S201C, Y242F, N249S | 54.74 | 99.26 |
| vS33 | D167S, A198H, S201C, Y242P, N249S | 62.53 | 99.66 |
| vS34 | D167S, A198Y, S201C, Y242P, A247C, N249G | 23.92 | 71.26 |
| vS35 | D167S, A198Y, S201C, Y242P, A247T, N249G | 24.42 | 89.51 |
| vS36 | D167S, A198M, S201C, Y242F, A247C, N249S | 12.64 | 59.66 |
| vS37 | D167P, A198M, S201C, Y242F, A247T, N249S | 0.18 | -62.72 |
| vS38 | D167H, A198H, S201C, Y242F, N249S | 62.13 | 99.96 |
| vS39 | D167H, A198H, S201C, Y242P, N249S | 83.02 | 99.99 |
| vS40 | D167H, A198H, S201C, Y242P, A247T, N249S | 11.57 | 98.92 |
| vS41 | D167H, A198H, S201C, Y242F, A247T, N249S | 57.49 | 98.93 |
| vS42 | D167H, A198Y, S201C, Y242P, N249S | 62.73 | 99.10 |
| vS43 | D167H, A198Y, S201C, Y242P, A247C, N249G | 22.89 | 73.84 |
| vS44 | D167H, A198Y, S201C, Y242P, A247T, N249G | 32.80 | 92.05 |
| vS45 | D167H, A198Y, S201C, Y242P, N249G | 82.36 | 97.84 |
| vS46 | A74G, A134V, D167H, A198H, S201C, Y242F, N249S | 6.13 | 99.39 |
| v47 | D167E, D197G, S201N, S213A, Y242F | 4.38 | 70.20 |
| v49 | D167C, S201C, S213H, Y242W, N249A | 3.11 | 77.28 |

**Table S7.** Screening results for the methylation of 1*H*-benzo[*d*]imidazole-5-carbonitrile using the combinatorial SOLD library.

| Name | Mutations based on *hsa*-N-NMT | relative activity |
|---|---|---|
| Empy vector | | 0.58 |
| Empy vector | | 1.04 |
| Empy vector | | 0.84 |
| Empy vector | | 0.21 |
| Growth control | | 0.25 |
| Growth control | | 0.59 |
| Growth control | | 0.44 |
| Growth control | | 0.34 |
| *hsa-N-NMT* | wild type | 1.47 |
| *hsa-N-NMT* | wild type | 0.53 |
| v31 | D167H, A198M, S201C, Y242F, N249S | 32.55 |
| v31 | D167H, A198M, S201C, Y242F, N249S | 32.31 |
| v31 | D167H, A198M, S201C, Y242F, N249S | 26.92 |
| vS01 | A74G, A134V, D167S, A198H, S201C, Y242F, N249S | 210.64 |
| vS02 | A74G, A134V, D167H, A198H, S201C, Y242P, N249S | 215.31 |
| vS03 | A74G, A134V, D167H, A198H, S201C, Y242F, A247T, N249S | 185.20 |
| vS04 | A74G, A134V, D167H, A198Y, S201C, Y242P, A247C, N249G | 183.27 |
| vS05 | A74G, A134V, D167H, A198Y, S201C, Y242P, N249G | 160.31 |
| vS06 | A74G, L154Q, D167H, A198H, S201C, Y242F, N249S | 177.87 |
| vS07 | A74G, D167S, A198H, S201C, Y242F, N249S | 140.77 |
| vS08 | A74G, D167S, A198H, S201C, Y242P, N249S | 207.47 |
| vS09 | A74G, D167S, A198Y, S201C, Y242P, A247C, N249G | 169.58 |
| vS10 | A74G, D167H, A198H, S201C, Y242F, N249S | 76.07 |
| vS11 | A74G, D167H, A198H, S201C, Y242P, N249S | 206.56 |
| vS12 | A74G, D167H, A198H, S201C, Y242P, A247T, N249S | 140.37 |
| vS13 | A74G, D167H, A198H, S201C, Y242F, A247T, N249S | 182.68 |
| vS14 | A74G, D167H, A198Y, S201C, Y242P, A247C, N249G | 206.79 |
| vS15 | A74G, D167H, A198Y, S201C, Y242P, N249G | 174.37 |
| vS16 | V82F, D167S, A198H, S201C, Y242P, N249S | 81.75 |
| vS17 | A134V, D167S, A198H, S201C, Y242F, N249S | 194.98 |
| vS18 | A134V, D167S, A198H, S201C, Y242P, N249S | 178.66 |
| vS19 | A134V, D167S, A198Y, S201C, Y242P, N249G | 193.79 |
| vS20 | A134V, D167H, P171L, A198Y, S201C, Y242F, A247C, N249G | 92.23 |
| vS21 | A134V, D167H, A198H, S201C, Y242F, N249S | 203.13 |
| vS22 | A134V, D167H, A198H, S201C, Y242P, N249S | 192.36 |
| vS23 | A134V, D167H, A198H, S201C, Y242P, A247T, N249S | 123.88 |
| vS24 | A134V, D167H, A198H, S201C, Y242F, A247T, N249S | 208.10 |
| vS25 | A134V, D167H, A198Y, S201C, Y242P, N249S | 113.20 |
| vS26 | A134V, D167H, A198Y, S201C, Y242P, A247C, N249S | 82.55 |
| vS27 | A134V, D167H, A198Y, S201C, Y242P, A247C, N249G | 181.57 |
| vS28 | A134V, D167H, A198Y, S201C, Y242P, N249G | 221.87 |
| vS29 | A134V, D167H, A198M, S201C, Y242P, N249G | 184.03 |
| vS30 | D167L, A198H, S201C, Y242F, A247T, N249S | 170.65 |
| vS31 | D167L, A198Y, S201C, Y242P, N249G | 189.86 |
| vS32 | D167S, A198H, S201C, Y242F, N249S | 209.57 |
| vS33 | D167S, A198H, S201C, Y242P, N249S | 179.72 |
| vS34 | D167S, A198Y, S201C, Y242P, A247C, N249G | 144.89 |
| vS35 | D167S, A198Y, S201C, Y242P, A247T, N249G | 97.13 |
| vS36 | D167S, A198M, S201C, Y242F, A247C, N249S | 176.71 |
| vS37 | D167P, A198M, S201C, Y242F, A247T, N249S | 1.16 |
| vS38 | D167H, A198H, S201C, Y242F, N249S | 161.22 |
| vS39 | D167H, A198H, S201C, Y242P, N249S | 179.68 |
| vS40 | D167H, A198H, S201C, Y242P, A247T, N249S | 94.61 |
| vS41 | D167H, A198H, S201C, Y242F, A247T, N249S | 177.52 |
| vS42 | D167H, A198Y, S201C, Y242P, N249S | 126.07 |
| vS43 | D167H, A198Y, S201C, Y242P, A247C, N249G | 185.03 |
| vS44 | D167H, A198Y, S201C, Y242P, A247T, N249G | 114.24 |
| vS45 | D167H, A198Y, S201C, Y242P, N249G | 182.09 |
| vS46 | A74G, A134V, D167H, A198H, S201C, Y242F, N249S | 9.77 |

**Table S8.** Screening results for the methylation of 1*H*-benzo[*d*]imidazole-5-carbaldehyde using the combinatorial SOLD library.

| Name | Mutations based on *hsa*-N-NMT | relative activity | selectivity (r.e.) |
|---|---|---|---|
| Empy vector | | 1.13 | -7.45 |
| Empy vector | | 0.79 | -9.92 |
| Empy vector | | 0.65 | -42.61 |
| Growth control | | 0.41 | -10.99 |
| Growth control | | 1.01 | -42.39 |
| *hsa-N-NMT* | wild type | 1.06 | -27.14 |
| *hsa-N-NMT* | wild type | 0.94 | -10.84 |
| v31 | D167H, A198M, S201C, Y242F, N249S | 12.85 | 92.38 |
| v31 | D167H, A198M, S201C, Y242F, N249S | 8.42 | 93.51 |
| v31 | D167H, A198M, S201C, Y242F, N249S | 8.28 | 96.32 |
| vS01 | A74G, A134V, D167S, A198H, S201C, Y242F, N249S | 111.28 | 94.89 |
| vS02 | A74G, A134V, D167H, A198H, S201C, Y242P, N249S | 99.42 | 99.60 |
| vS03 | A74G, A134V, D167H, A198H, S201C, Y242F, A247T, N249S | 117.11 | 97.67 |
| vS04 | A74G, A134V, D167H, A198Y, S201C, Y242P, A247C, N249G | 58.06 | 86.57 |
| vS05 | A74G, A134V, D167H, A198Y, S201C, Y242P, N249G | 145.42 | 96.49 |
| vS05 | A74G, A134V, D167H, A198Y, S201C, Y242P, N249G | 125.45 | 96.67 |
| vS06 | A74G, L154Q, D167H, A198H, S201C, Y242F, N249S | 41.12 | 99.55 |
| vS07 | A74G, D167S, A198H, S201C, Y242F, N249S | 72.27 | 94.40 |
| vS08 | A74G, D167S, A198H, S201C, Y242P, N249S | 98.63 | 93.90 |
| vS09 | A74G, D167S, A198Y, S201C, Y242P, A247C, N249G | 81.47 | 86.43 |
| vS10 | A74G, D167H, A198H, S201C, Y242F, N249S | 19.82 | 98.24 |
| vS11 | A74G, D167H, A198H, S201C, Y242P, N249S | 94.32 | 99.40 |
| vS12 | A74G, D167H, A198H, S201C, Y242P, A247T, N249S | 37.87 | 96.50 |
| vS13 | A74G, D167H, A198H, S201C, Y242F, A247T, N249S | 114.20 | 98.75 |
| vS14 | A74G, D167H, A198Y, S201C, Y242P, A247C, N249G | 72.56 | 84.87 |
| vS15 | A74G, D167H, A198Y, S201C, Y242P, N249G | 125.23 | 96.20 |
| vS16 | V82F, D167S, A198H, S201C, Y242P, N249S | 33.11 | 96.38 |
| vS17 | A134V, D167S, A198H, S201C, Y242F, N249S | 53.07 | 91.99 |
| vS18 | A134V, D167S, A198H, S201C, Y242P, N249S | 77.01 | 90.77 |
| vS19 | A134V, D167S, A198Y, S201C, Y242P, N249G | 142.21 | 99.01 |
| vS20 | A134V, D167H, P171L, A198Y, S201C, Y242F, A247C, N249G | 21.37 | 92.46 |
| vS21 | A134V, D167H, A198H, S201C, Y242F, N249S | 74.29 | 98.99 |
| vS21 | A134V, D167H, A198H, S201C, Y242F, N249S | 65.52 | 99.50 |
| vS22 | A134V, D167H, A198H, S201C, Y242P, N249S | 105.12 | 99.92 |
| vS23 | A134V, D167H, A198H, S201C, Y242P, A247T, N249S | 38.91 | 97.66 |
| vS24 | A134V, D167H, A198H, S201C, Y242F, A247T, N249S | 117.22 | 98.32 |
| vS25 | A134V, D167H, A198Y, S201C, Y242P, N249S | 86.01 | 98.38 |
| vS26 | A134V, D167H, A198Y, S201C, Y242P, A247C, N249S | 47.28 | 99.10 |
| vS27 | A134V, D167H, A198Y, S201C, Y242P, A247C, N249G | 67.43 | 86.66 |
| vS28 | A134V, D167H, A198Y, S201C, Y242P, N249G | 151.50 | 96.59 |
| vS29 | A134V, D167H, A198M, S201C, Y242P, N249G | 96.04 | 90.39 |
| vS30 | D167L, A198H, S201C, Y242F, A247T, N249S | 153.84 | 92.77 |
| vS31 | D167L, A198Y, S201C, Y242P, N249G | 128.00 | 94.30 |
| vS32 | D167S, A198H, S201C, Y242F, N249S | 72.82 | 93.51 |
| vS33 | D167S, A198H, S201C, Y242P, N249S | 98.51 | 91.35 |
| vS34 | D167S, A198Y, S201C, Y242P, A247C, N249G | 79.60 | 85.97 |
| vS35 | D167S, A198Y, S201C, Y242P, A247T, N249G | 38.12 | 82.93 |
| vS36 | D167S, A198M, S201C, Y242F, A247C, N249S | 33.37 | 67.03 |
| vS37 | D167P, A198M, S201C, Y242F, A247T, N249S | 1.69 | 16.41 |
| vS38 | D167H, A198H, S201C, Y242F, N249S | 67.35 | 99.20 |
| vS39 | D167H, A198H, S201C, Y242P, N249S | 109.80 | 99.51 |
| vS40 | D167H, A198H, S201C, Y242P, A247T, N249S | 22.36 | 99.52 |
| vS41 | D167H, A198H, S201C, Y242F, A247T, N249S | 129.41 | 98.39 |
| vS42 | D167H, A198Y, S201C, Y242P, N249S | 108.15 | 99.09 |
| vS43 | D167H, A198Y, S201C, Y242P, A247C, N249G | 76.44 | 85.77 |
| vS44 | D167H, A198Y, S201C, Y242P, A247T, N249G | 56.86 | 92.24 |
| vS45 | D167H, A198Y, S201C, Y242P, N249G | 156.24 | 96.14 |
| vS46 | A74G, A134V, D167H, A198H, S201C, Y242F, N249S | 5.54 | 74.77 |

**Table S9.** Screening results for the methylation of 5-methoxy-1*H*-benzo[*d*]imidazole using the combinatorial SOLD library.

| Name | Mutations based on *hsa*-N-NMT | relative activity | selectivity (r.e.) |
|---|---|---|---|
| Empy vector | | 0.98 | -7.80 |
| Empy vector | | 0.65 | 36.32 |
| *hsa-H-NMT* | wild type | 177.63 | -84.92 |
| Growth control | | 0.98 | 25.39 |
| Growth control | | 1.29 | -11.53 |
| Growth control | | 0.95 | 37.27 |
| *hsa-N-NMT* | wild type | 1.11 | 8.54 |
| *hsa-N-NMT* | wild type | 0.89 | 22.52 |
| v31 | D167H, A198M, S201C, Y242F, N249S | 260.07 | 73.33 |
| v31 | D167H, A198M, S201C, Y242F, N249S | 239.86 | 73.87 |
| v31 | D167H, A198M, S201C, Y242F, N249S | 235.93 | 73.65 |
| vS01 | A74G, A134V, D167S, A198H, S201C, Y242F, N249S | 369.78 | 81.38 |
| vS02 | A74G, A134V, D167H, A198H, S201C, Y242P, N249S | 576.51 | 90.48 |
| vS03 | A74G, A134V, D167H, A198H, S201C, Y242F, A247T, N249S | 511.52 | 83.44 |
| vS04 | A74G, A134V, D167H, A198Y, S201C, Y242P, A247C, N249G | 497.92 | -35.71 |
| vS05 | A74G, A134V, D167H, A198Y, S201C, Y242P, N249G | 549.62 | 72.67 |
| vS06 | A74G, L154Q, D167H, A198H, S201C, Y242F, N249S | 403.27 | 89.26 |
| vS07 | A74G, D167S, A198H, S201C, Y242F, N249S | 381.33 | 81.64 |
| vS08 | A74G, D167S, A198H, S201C, Y242P, N249S | 437.01 | 86.18 |
| vS09 | A74G, D167S, A198Y, S201C, Y242P, A247C, N249G | 315.05 | -39.82 |
| vS10 | A74G, D167H, A198H, S201C, Y242F, N249S | 196.26 | 89.61 |
| vS11 | A74G, D167H, A198H, S201C, Y242P, N249S | 526.51 | 90.67 |
| vS12 | A74G, D167H, A198H, S201C, Y242P, A247T, N249S | 302.99 | 77.98 |
| vS13 | A74G, D167H, A198H, S201C, Y242F, A247T, N249S | 503.68 | 83.87 |
| vS14 | A74G, D167H, A198Y, S201C, Y242P, A247C, N249G | 458.28 | -37.86 |
| vS15 | A74G, D167H, A198Y, S201C, Y242P, N249G | 506.99 | 71.73 |
| vS16 | V82F, D167S, A198H, S201C, Y242P, N249S | 257.11 | 84.74 |
| vS17 | A134V, D167S, A198H, S201C, Y242F, N249S | 224.60 | 80.51 |
| vS18 | A134V, D167S, A198H, S201C, Y242P, N249S | 332.18 | 85.76 |
| vS19 | A134V, D167S, A198Y, S201C, Y242P, N249G | 350.55 | 79.05 |
| vS20 | A134V, D167H, P171L, A198Y, S201C, Y242F, A247C, N249G | 259.21 | -9.28 |
| vS21 | A134V, D167H, A198H, S201C, Y242F, N249S | 370.78 | 89.26 |
| vS22 | A134V, D167H, A198H, S201C, Y242P, N249S | 476.86 | 90.79 |
| vS23 | A134V, D167H, A198H, S201C, Y242P, A247T, N249S | 290.95 | 77.41 |
| vS24 | A134V, D167H, A198H, S201C, Y242F, A247T, N249S | 436.90 | 83.63 |
| vS25 | A134V, D167H, A198Y, S201C, Y242P, N249S | 471.51 | 94.29 |
| vS26 | A134V, D167H, A198Y, S201C, Y242P, A247C, N249S | 441.64 | 71.70 |
| vS27 | A134V, D167H, A198Y, S201C, Y242P, A247C, N249G | 410.02 | -33.50 |
| vS28 | A134V, D167H, A198Y, S201C, Y242P, N249G | 455.23 | 73.09 |
| vS29 | A134V, D167H, A198M, S201C, Y242P, N249G | 417.51 | 51.90 |
| vS30 | D167L, A198H, S201C, Y242F, A247T, N249S | 459.75 | 69.17 |
| vS31 | D167L, A198Y, S201C, Y242P, N249G | 437.56 | 68.42 |
| vS32 | D167S, A198H, S201C, Y242F, N249S | 314.27 | 82.20 |
| vS33 | D167S, A198H, S201C, Y242P, N249S | 335.81 | 86.78 |
| vS34 | D167S, A198Y, S201C, Y242P, A247C, N249G | 244.43 | -35.32 |
| vS35 | D167S, A198Y, S201C, Y242P, A247T, N249G | 312.33 | 73.22 |
| vS36 | D167S, A198M, S201C, Y242F, A247C, N249S | 252.82 | -15.81 |
| vS37 | D167P, A198M, S201C, Y242F, A247T, N249S | 1.96 | 3.09 |
| vS38 | D167H, A198H, S201C, Y242F, N249S | 380.28 | 89.28 |
| vS39 | D167H, A198H, S201C, Y242P, N249S | 442.63 | 90.87 |
| vS40 | D167H, A198H, S201C, Y242P, A247T, N249S | 154.44 | 79.72 |
| vS41 | D167H, A198H, S201C, Y242F, A247T, N249S | 421.41 | 84.31 |
| vS42 | D167H, A198Y, S201C, Y242P, N249S | 427.82 | 94.29 |
| vS43 | D167H, A198Y, S201C, Y242P, A247C, N249G | 386.92 | -34.43 |
| vS44 | D167H, A198Y, S201C, Y242P, A247T, N249G | 426.53 | 60.80 |
| vS45 | D167H, A198Y, S201C, Y242P, N249G | 436.74 | 72.70 |
| vS46 | A74G, A134V, D167H, A198H, S201C, Y242F, N249S | 78.60 | 93.61 |
| v47 | D167E, D197G, S201N, S213A, Y242F | 418.61 | -61.76 |
| v49 | D167C, S201C, S213H, Y242W, N249A | 15.61 | 5.26 |

**Table S10.** Screening results for the methylation of 5-fluoro-1*H*-benzo[*d*]imidazole using the combinatorial SOLD library.

| Name | Mutations based on *hsa*-N-NMT | relative activity | selectivity (r.e.) |
|---|---|---|---|
| Empy vector | | 0.09 | n.d. |
| Empy vector | | 0.01 | n.d. |
| *hsa-H-NMT* | wild type | 26.41 | -71.91 |
| Growth control | | 0.10 | n.d. |
| Growth control | | 0.02 | n.d. |
| Growth control | | 0.02 | n.d. |
| *hsa-N-NMT* | wild type | 1.08 | 24.10 |
| *hsa-N-NMT* | wild type | 0.92 | 30.26 |
| v31 | D167H, A198M, S201C, Y242F, N249S | 10.28 | 97.43 |
| v31 | D167H, A198M, S201C, Y242F, N249S | 10.15 | 97.43 |
| v31 | D167H, A198M, S201C, Y242F, N249S | 9.87 | 97.37 |
| vS01 | A74G, A134V, D167S, A198H, S201C, Y242F, N249S | 33.29 | 99.57 |
| vS02 | A74G, A134V, D167H, A198H, S201C, Y242P, N249S | 34.28 | 99.60 |
| vS03 | A74G, A134V, D167H, A198H, S201C, Y242F, A247T, N249S | 44.87 | 99.85 |
| vS04 | A74G, A134V, D167H, A198Y, S201C, Y242P, A247C, N249G | 28.66 | 87.21 |
| vS05 | A74G, A134V, D167H, A198Y, S201C, Y242P, N249G | 34.05 | 97.54 |
| vS06 | A74G, L154Q, D167H, A198H, S201C, Y242F, N249S | 29.63 | 99.76 |
| vS07 | A74G, D167S, A198H, S201C, Y242F, N249S | 35.60 | 99.79 |
| vS08 | A74G, D167S, A198H, S201C, Y242P, N249S | 30.31 | 99.99 |
| vS09 | A74G, D167S, A198Y, S201C, Y242P, A247C, N249G | 30.71 | 98.89 |
| vS10 | A74G, D167H, A198H, S201C, Y242F, N249S | 14.31 | 99.92 |
| vS11 | A74G, D167H, A198H, S201C, Y242P, N249S | 33.65 | 99.90 |
| vS12 | A74G, D167H, A198H, S201C, Y242P, A247T, N249S | 25.76 | 99.79 |
| vS13 | A74G, D167H, A198H, S201C, Y242F, A247T, N249S | 43.18 | 99.68 |
| vS14 | A74G, D167H, A198Y, S201C, Y242P, A247C, N249G | 29.05 | 86.26 |
| vS15 | A74G, D167H, A198Y, S201C, Y242P, N249G | 34.74 | 97.92 |
| vS16 | V82F, D167S, A198H, S201C, Y242P, N249S | 22.79 | 99.81 |
| vS17 | A134V, D167S, A198H, S201C, Y242F, N249S | 30.04 | 99.91 |
| vS18 | A134V, D167S, A198H, S201C, Y242P, N249S | 23.82 | 99.40 |
| vS19 | A134V, D167S, A198Y, S201C, Y242P, N249G | 41.97 | 100.00 |
| vS20 | A134V, D167H, P171L, A198Y, S201C, Y242F, A247C, N249G | 26.98 | 88.44 |
| vS21 | A134V, D167H, A198H, S201C, Y242F, N249S | 32.92 | 99.79 |
| vS22 | A134V, D167H, A198H, S201C, Y242P, N249S | 41.90 | 99.96 |
| vS23 | A134V, D167H, A198H, S201C, Y242P, A247T, N249S | 27.05 | 99.83 |
| vS24 | A134V, D167H, A198H, S201C, Y242F, A247T, N249S | 51.06 | 99.88 |
| vS25 | A134V, D167H, A198Y, S201C, Y242P, N249S | 21.18 | 95.45 |
| vS26 | A134V, D167H, A198Y, S201C, Y242P, A247C, N249S | 19.25 | 83.73 |
| vS27 | A134V, D167H, A198Y, S201C, Y242P, A247C, N249G | 31.18 | 87.64 |
| vS28 | A134V, D167H, A198Y, S201C, Y242P, N249G | 39.65 | 98.24 |
| vS29 | A134V, D167H, A198M, S201C, Y242P, N249G | 43.96 | 99.70 |
| vS30 | D167L, A198H, S201C, Y242F, A247T, N249S | 49.20 | 99.78 |
| vS31 | D167L, A198Y, S201C, Y242P, N249G | 40.38 | 98.05 |
| vS32 | D167S, A198H, S201C, Y242F, N249S | 46.97 | 99.35 |
| vS33 | D167S, A198H, S201C, Y242P, N249S | 32.35 | 99.56 |
| vS34 | D167S, A198Y, S201C, Y242P, A247C, N249G | 35.23 | 98.64 |
| vS35 | D167S, A198Y, S201C, Y242P, A247T, N249G | 34.24 | 99.60 |
| vS36 | D167S, A198M, S201C, Y242F, A247C, N249S | 41.77 | 97.58 |
| vS37 | D167P, A198M, S201C, Y242F, A247T, N249S | 2.79 | -93.20 |
| vS38 | D167H, A198H, S201C, Y242F, N249S | 43.98 | 99.36 |
| vS39 | D167H, A198H, S201C, Y242P, N249S | 42.89 | 99.47 |
| vS40 | D167H, A198H, S201C, Y242P, A247T, N249S | 22.72 | 97.20 |
| vS41 | D167H, A198H, S201C, Y242F, A247T, N249S | 53.16 | 99.57 |
| vS42 | D167H, A198Y, S201C, Y242P, N249S | 31.71 | 94.77 |
| vS43 | D167H, A198Y, S201C, Y242P, A247C, N249G | 37.78 | 88.25 |
| vS44 | D167H, A198Y, S201C, Y242P, A247T, N249G | 45.21 | 97.02 |
| vS45 | D167H, A198Y, S201C, Y242P, N249G | 21.24 | 98.15 |
| vS46 | A74G, A134V, D167H, A198H, S201C, Y242F, N249S | 2.36 | 95.49 |
| v47 | D167E, D197G, S201N, S213A, Y242F | 6.86 | 63.09 |
| v49 | D167C, S201C, S213H, Y242W, N249A | 1.34 | 86.00 |

**Table S11.** Screening results for the methylation of 5-chloro-1*H*-benzo[*d*]imidazole using the combinatorial SOLD library.

| Name | Mutations based on *hsa*-N-NMT | relative activity | selectivity (r.e.) |
|---|---|---|---|
| Empy vector | | 0.10 | n.d. |
| Empy vector | | 0.14 | n.d. |
| *hsa-H-NMT* | wild type | 138.77 | -94.42 |
| Growth control | | 0.14 | n.d. |
| Growth control | | 0.31 | n.d. |
| Growth control | | 0.05 | n.d. |
| *hsa-N-NMT* | wild type | 1.10 | 8.26 |
| *hsa-N-NMT* | wild type | 0.90 | 20.14 |
| v31 | D167H, A198M, S201C, Y242F, N249S | 75.83 | 97.47 |
| v31 | D167H, A198M, S201C, Y242F, N249S | 66.51 | 97.63 |
| v31 | D167H, A198M, S201C, Y242F, N249S | 66.50 | 97.44 |
| vS01 | A74G, A134V, D167S, A198H, S201C, Y242F, N249S | 122.12 | 99.48 |
| vS02 | A74G, A134V, D167H, A198H, S201C, Y242P, N249S | 125.40 | 99.69 |
| vS03 | A74G, A134V, D167H, A198H, S201C, Y242F, A247T, N249S | 137.45 | 99.82 |
| vS04 | A74G, A134V, D167H, A198Y, S201C, Y242P, A247C, N249G | 115.01 | 59.90 |
| vS05 | A74G, A134V, D167H, A198Y, S201C, Y242P, N249G | 115.69 | 91.31 |
| vS06 | A74G, L154Q, D167H, A198H, S201C, Y242F, N249S | 128.10 | 99.95 |
| vS07 | A74G, D167S, A198H, S201C, Y242F, N249S | 131.77 | 99.65 |
| vS08 | A74G, D167S, A198H, S201C, Y242P, N249S | 123.99 | 99.64 |
| vS09 | A74G, D167S, A198Y, S201C, Y242P, A247C, N249G | 91.63 | 70.80 |
| vS10 | A74G, D167H, A198H, S201C, Y242F, N249S | 113.67 | 99.80 |
| vS11 | A74G, D167H, A198H, S201C, Y242P, N249S | 123.76 | 99.80 |
| vS12 | A74G, D167H, A198H, S201C, Y242P, A247T, N249S | 117.08 | 99.42 |
| vS13 | A74G, D167H, A198H, S201C, Y242F, A247T, N249S | 124.45 | 99.89 |
| vS14 | A74G, D167H, A198Y, S201C, Y242P, A247C, N249G | 102.33 | 61.12 |
| vS15 | A74G, D167H, A198Y, S201C, Y242P, N249G | 110.28 | 91.45 |
| vS16 | V82F, D167S, A198H, S201C, Y242P, N249S | 69.68 | 98.85 |
| vS17 | A134V, D167S, A198H, S201C, Y242F, N249S | 127.83 | 99.72 |
| vS18 | A134V, D167S, A198H, S201C, Y242P, N249S | 106.99 | 99.47 |
| vS19 | A134V, D167S, A198Y, S201C, Y242P, N249G | 94.55 | 96.31 |
| vS20 | A134V, D167H, P171L, A198Y, S201C, Y242F, A247C, N249G | 101.76 | 33.80 |
| vS21 | A134V, D167H, A198H, S201C, Y242F, N249S | 113.04 | 99.84 |
| vS22 | A134V, D167H, A198H, S201C, Y242P, N249S | 106.24 | 99.82 |
| vS23 | A134V, D167H, A198H, S201C, Y242P, A247T, N249S | 112.45 | 99.68 |
| vS24 | A134V, D167H, A198H, S201C, Y242F, A247T, N249S | 111.59 | 99.56 |
| vS25 | A134V, D167H, A198Y, S201C, Y242P, N249S | 101.09 | 96.58 |
| vS26 | A134V, D167H, A198Y, S201C, Y242P, A247C, N249S | 96.12 | 86.23 |
| vS27 | A134V, D167H, A198Y, S201C, Y242P, A247C, N249G | 97.84 | 63.38 |
| vS28 | A134V, D167H, A198Y, S201C, Y242P, N249G | 101.52 | 92.63 |
| vS29 | A134V, D167H, A198M, S201C, Y242P, N249G | 103.39 | 97.28 |
| vS30 | D167L, A198H, S201C, Y242F, A247T, N249S | 103.33 | 98.67 |
| vS31 | D167L, A198Y, S201C, Y242P, N249G | 93.97 | 82.20 |
| vS32 | D167S, A198H, S201C, Y242F, N249S | 102.07 | 99.89 |
| vS33 | D167S, A198H, S201C, Y242P, N249S | 99.94 | 99.59 |
| vS34 | D167S, A198Y, S201C, Y242P, A247C, N249G | 74.38 | 73.89 |
| vS35 | D167S, A198Y, S201C, Y242P, A247T, N249G | 89.64 | 91.31 |
| vS36 | D167S, A198M, S201C, Y242F, A247C, N249S | 84.39 | 76.97 |
| vS37 | D167P, A198M, S201C, Y242F, A247T, N249S | 4.55 | -68.50 |
| vS38 | D167H, A198H, S201C, Y242F, N249S | 98.79 | 99.81 |
| vS39 | D167H, A198H, S201C, Y242P, N249S | 102.84 | 99.62 |
| vS40 | D167H, A198H, S201C, Y242P, A247T, N249S | 90.26 | 99.54 |
| vS41 | D167H, A198H, S201C, Y242F, A247T, N249S | 101.87 | 99.97 |
| vS42 | D167H, A198Y, S201C, Y242P, N249S | 93.48 | 95.55 |
| vS43 | D167H, A198Y, S201C, Y242P, A247C, N249G | 87.95 | 63.44 |
| vS44 | D167H, A198Y, S201C, Y242P, A247T, N249G | 91.96 | 88.54 |
| vS45 | D167H, A198Y, S201C, Y242P, N249G | 96.34 | 92.27 |
| vS46 | A74G, A134V, D167H, A198H, S201C, Y242F, N249S | 16.05 | 96.33 |
| v47 | D167E, D197G, S201N, S213A, Y242F | 33.78 | 60.21 |
| v49 | D167C, S201C, S213H, Y242W, N249A | 9.75 | -51.36 |

**Table S12.** Screening results for the methylation of 5-iodo-1*H*-benzo[*d*]imidazole using the combinatorial SOLD library.

| Name | Mutations based on *hsa*-N-NMT | relative activity | selectivity (r.e.) |
|---|---|---|---|
| Empy vector | | 0.05 | n.d. |
| Empy vector | | 0.00 | n.d. |
| Empy vector | | 0.00 | n.d. |
| Growth control | | 0.00 | n.d. |
| Growth control | | 0.00 | n.d. |
| *hsa-N-NMT* | wild type | 1.00 | n.d. |
| *hsa-N-NMT* | wild type | 1.00 | n.d. |
| v31 | D167H, A198M, S201C, Y242F, N249S | 116.32 | 87.81 |
| v31 | D167H, A198M, S201C, Y242F, N249S | 105.15 | 87.90 |
| v31 | D167H, A198M, S201C, Y242F, N249S | 72.37 | 100.00 |
| vS01 | A74G, A134V, D167S, A198H, S201C, Y242F, N249S | 230.26 | 100.00 |
| vS02 | A74G, A134V, D167H, A198H, S201C, Y242P, N249S | 228.44 | 100.00 |
| vS03 | A74G, A134V, D167H, A198H, S201C, Y242F, A247T, N249S | 195.88 | 100.00 |
| vS04 | A74G, A134V, D167H, A198Y, S201C, Y242P, A247C, N249G | 185.90 | 7.43 |
| vS05 | A74G, A134V, D167H, A198Y, S201C, Y242P, N249G | 219.77 | 79.35 |
| vS06 | A74G, L154Q, D167H, A198H, S201C, Y242F, N249S | 228.84 | 100.00 |
| vS07 | A74G, D167S, A198H, S201C, Y242F, N249S | 225.95 | 100.00 |
| vS08 | A74G, D167S, A198H, S201C, Y242P, N249S | 189.39 | 100.00 |
| vS09 | A74G, D167S, A198Y, S201C, Y242P, A247C, N249G | 187.14 | 0.63 |
| vS10 | A74G, D167H, A198H, S201C, Y242F, N249S | 205.15 | 100.00 |
| vS11 | A74G, D167H, A198H, S201C, Y242P, N249S | 206.44 | 100.00 |
| vS12 | A74G, D167H, A198H, S201C, Y242P, A247T, N249S | 204.05 | 92.25 |
| vS13 | A74G, D167H, A198H, S201C, Y242F, A247T, N249S | 201.63 | 100.00 |
| vS14 | A74G, D167H, A198Y, S201C, Y242P, A247C, N249G | 161.35 | 15.25 |
| vS15 | A74G, D167H, A198Y, S201C, Y242P, N249G | 204.55 | 78.92 |
| vS16 | V82F, D167S, A198H, S201C, Y242P, N249S | 53.06 | 100.00 |
| vS17 | A134V, D167S, A198H, S201C, Y242F, N249S | 184.44 | 100.00 |
| vS18 | A134V, D167S, A198H, S201C, Y242P, N249S | 174.07 | 100.00 |
| vS19 | A134V, D167S, A198Y, S201C, Y242P, N249G | 154.94 | 77.53 |
| vS20 | A134V, D167H, P171L, A198Y, S201C, Y242F, A247C, N249G | 166.82 | -22.64 |
| vS21 | A134V, D167H, A198H, S201C, Y242F, N249S | 190.46 | 100.00 |
| vS22 | A134V, D167H, A198H, S201C, Y242P, N249S | 156.69 | 100.00 |
| vS23 | A134V, D167H, A198H, S201C, Y242P, A247T, N249S | 160.79 | 90.63 |
| vS24 | A134V, D167H, A198H, S201C, Y242F, A247T, N249S | 171.47 | 100.00 |
| vS25 | A134V, D167H, A198Y, S201C, Y242P, N249S | 203.84 | 93.59 |
| vS26 | A134V, D167H, A198Y, S201C, Y242P, A247C, N249S | 191.64 | 79.52 |
| vS27 | A134V, D167H, A198Y, S201C, Y242P, A247C, N249G | 174.49 | 14.93 |
| vS28 | A134V, D167H, A198Y, S201C, Y242P, N249G | 201.66 | 80.04 |
| vS29 | A134V, D167H, A198M, S201C, Y242P, N249G | 202.89 | 85.00 |
| vS30 | D167L, A198H, S201C, Y242F, A247T, N249S | 419.74 | 85.32 |
| vS31 | D167L, A198Y, S201C, Y242P, N249G | 181.70 | 48.88 |
| vS32 | D167S, A198H, S201C, Y242F, N249S | 217.64 | 100.00 |
| vS33 | D167S, A198H, S201C, Y242P, N249S | 200.89 | 100.00 |
| vS34 | D167S, A198Y, S201C, Y242P, A247C, N249G | 145.34 | 0.17 |
| vS35 | D167S, A198Y, S201C, Y242P, A247T, N249G | 177.89 | 74.28 |
| vS36 | D167S, A198M, S201C, Y242F, A247C, N249S | 32.26 | 100.00 |
| vS37 | D167P, A198M, S201C, Y242F, A247T, N249S | 0.00 | n.d. |
| vS38 | D167H, A198H, S201C, Y242F, N249S | 237.82 | 100.00 |
| vS39 | D167H, A198H, S201C, Y242P, N249S | 206.85 | 100.00 |
| vS40 | D167H, A198H, S201C, Y242P, A247T, N249S | 141.27 | 91.28 |
| vS41 | D167H, A198H, S201C, Y242F, A247T, N249S | 22.48 | 100.00 |
| vS42 | D167H, A198Y, S201C, Y242P, N249S | 225.43 | 92.95 |
| vS43 | D167H, A198Y, S201C, Y242P, A247C, N249G | 164.37 | 11.95 |
| vS44 | D167H, A198Y, S201C, Y242P, A247T, N249G | 194.49 | 72.09 |
| vS45 | D167H, A198Y, S201C, Y242P, N249G | 0.00 | n.d. |
| vS46 | A74G, A134V, D167H, A198H, S201C, Y242F, N249S | 61.55 | 100.00 |

**Table S13.** Screening results for the methylation of 5-bromo-2-methyl-1*H*-benzo[*d*]imidazole using the combinatorial SOLD library.

| Name | Mutations based on *hsa*-N-NMT | relative activity | selectivity (r.e.) |
|---|---|---|---|
| Empy vector | | 0.12 | n.d. |
| Empy vector | | 0.08 | n.d. |
| *hsa-H-NMT* | wild type | 0.19 | n.d. |
| Growth control | | 0.14 | n.d. |
| Growth control | | 0.22 | n.d. |
| Growth control | | 0.04 | n.d. |
| *hsa-N-NMT* | wild type | 1.01 | -24.57 |
| *hsa-N-NMT* | wild type | 0.99 | -16.16 |
| v31 | D167H, A198M, S201C, Y242F, N249S | 8.92 | 98.37 |
| v31 | D167H, A198M, S201C, Y242F, N249S | 8.91 | 98.00 |
| v31 | D167H, A198M, S201C, Y242F, N249S | 8.88 | 98.06 |
| vS01 | A74G, A134V, D167S, A198H, S201C, Y242F, N249S | 54.93 | 99.98 |
| vS02 | A74G, A134V, D167H, A198H, S201C, Y242P, N249S | 54.62 | 100.00 |
| vS03 | A74G, A134V, D167H, A198H, S201C, Y242F, A247T, N249S | 53.54 | 99.97 |
| vS04 | A74G, A134V, D167H, A198Y, S201C, Y242P, A247C, N249G | 47.44 | 50.54 |
| vS05 | A74G, A134V, D167H, A198Y, S201C, Y242P, N249G | 46.53 | 92.51 |
| vS06 | A74G, L154Q, D167H, A198H, S201C, Y242F, N249S | 50.89 | 99.95 |
| vS07 | A74G, D167S, A198H, S201C, Y242F, N249S | 56.44 | 99.87 |
| vS08 | A74G, D167S, A198H, S201C, Y242P, N249S | 53.73 | 99.94 |
| vS09 | A74G, D167S, A198Y, S201C, Y242P, A247C, N249G | 40.61 | 87.34 |
| vS10 | A74G, D167H, A198H, S201C, Y242F, N249S | 34.15 | 99.81 |
| vS11 | A74G, D167H, A198H, S201C, Y242P, N249S | 50.38 | 99.90 |
| vS12 | A74G, D167H, A198H, S201C, Y242P, A247T, N249S | 49.27 | 99.92 |
| vS13 | A74G, D167H, A198H, S201C, Y242F, A247T, N249S | 44.62 | 99.91 |
| vS14 | A74G, D167H, A198Y, S201C, Y242P, A247C, N249G | 43.50 | 50.40 |
| vS15 | A74G, D167H, A198Y, S201C, Y242P, N249G | 42.42 | 92.11 |
| vS16 | V82F, D167S, A198H, S201C, Y242P, N249S | 21.25 | 99.98 |
| vS17 | A134V, D167S, A198H, S201C, Y242F, N249S | 43.22 | 100.00 |
| vS18 | A134V, D167S, A198H, S201C, Y242P, N249S | 46.31 | 99.95 |
| vS19 | A134V, D167S, A198Y, S201C, Y242P, N249G | 42.95 | 99.08 |
| vS20 | A134V, D167H, P171L, A198Y, S201C, Y242F, A247C, N249G | 26.46 | 16.38 |
| vS21 | A134V, D167H, A198H, S201C, Y242F, N249S | 45.87 | 99.98 |
| vS22 | A134V, D167H, A198H, S201C, Y242P, N249S | 45.35 | 99.92 |
| vS23 | A134V, D167H, A198H, S201C, Y242P, A247T, N249S | 44.97 | 99.93 |
| vS24 | A134V, D167H, A198H, S201C, Y242F, A247T, N249S | 44.41 | 99.98 |
| vS25 | A134V, D167H, A198Y, S201C, Y242P, N249S | 35.32 | 97.01 |
| vS26 | A134V, D167H, A198Y, S201C, Y242P, A247C, N249S | 28.71 | 85.12 |
| vS27 | A134V, D167H, A198Y, S201C, Y242P, A247C, N249G | 41.05 | 52.76 |
| vS28 | A134V, D167H, A198Y, S201C, Y242P, N249G | 41.03 | 91.91 |
| vS29 | A134V, D167H, A198M, S201C, Y242P, N249G | 32.25 | 98.18 |
| vS30 | D167L, A198H, S201C, Y242F, A247T, N249S | 41.20 | 99.74 |
| vS31 | D167L, A198Y, S201C, Y242P, N249G | 20.07 | 86.30 |
| vS32 | D167S, A198H, S201C, Y242F, N249S | 45.83 | 99.89 |
| vS33 | D167S, A198H, S201C, Y242P, N249S | 45.92 | 99.99 |
| vS34 | D167S, A198Y, S201C, Y242P, A247C, N249G | 34.59 | 86.80 |
| vS35 | D167S, A198Y, S201C, Y242P, A247T, N249G | 31.68 | 97.21 |
| vS36 | D167S, A198M, S201C, Y242F, A247C, N249S | 35.38 | 84.98 |
| vS37 | D167P, A198M, S201C, Y242F, A247T, N249S | 0.44 | -4.56 |
| vS38 | D167H, A198H, S201C, Y242F, N249S | 48.14 | 99.90 |
| vS39 | D167H, A198H, S201C, Y242P, N249S | 43.99 | 99.85 |
| vS40 | D167H, A198H, S201C, Y242P, A247T, N249S | 34.58 | 99.84 |
| vS41 | D167H, A198H, S201C, Y242F, A247T, N249S | 45.42 | 99.96 |
| vS42 | D167H, A198Y, S201C, Y242P, N249S | 37.70 | 97.28 |
| vS43 | D167H, A198Y, S201C, Y242P, A247C, N249G | 38.14 | 52.02 |
| vS44 | D167H, A198Y, S201C, Y242P, A247T, N249G | 28.93 | 84.58 |
| vS45 | D167H, A198Y, S201C, Y242P, N249G | 38.05 | 91.51 |
| vS46 | A74G, A134V, D167H, A198H, S201C, Y242F, N249S | 5.80 | 96.94 |
| v47 | D167E, D197G, S201N, S213A, Y242F | 42.98 | 70.92 |
| v49 | D167C, S201C, S213H, Y242W, N249A | 0.21 | n.d. |

**Table S14** Primer Sequences

| Wild type MTs panel | Sequence 5´→ 3´ |
|---|---|
| pET28-T7-fwd | TTGTTTAACTTTAAGAAGGAGATATACCATG |
| pET28-T7-rev | CATGGTATATCTCCTTCTTAAAGTTAAACAA |
| pET28-Term-fwd | CTGAGATCCGGCTGCTAAC |
| pET28-Term-rev | GTTAGCAGCCGGATCTCAG |

| SOLD library | Sequence 5´→ 3´ |
|---|---|
| Fragment_fwd | GGTCCCACAATTTACCAACTTC |
| Fragment_rev | CGAGCAACCAGCGAAAATAAG |
| Backbone_fwd | CTTATTTTCGCTGGTTGCTCG |
| Backbone_rev | GAAGTTGGTAAATTGTGGGACC |

| SSM v31 | Sequence 5´→ 3´ |
|---|---|
| H167_NDT_fwd | TATTGTCAACCCTTTGCTTGNDTGCAGCTTGTCCTGAC |
| H167_VHG_fwd | TATTGTCAACCCTTTGCTTGVHGGCAGCTTGTCCTGAC |
| H167_TGG_fwd | TATTGTCAACCCTTTGCTTGTGGGCAGCTTGTCCTGAC |
| H167_rev | CAAGCAAAGGGTTGACAATACACAATCCGCCGGGG |
| M198_NDT_fwd | GGTTTCTGGTCATCATGGATNDTTTGAAGTGCTCCTACTACATG |
| M198_VHG_fwd | GGTTTCTGGTCATCATGGATVHGTTGAAGTGCTCCTACTACATG |
| M198_TGG_fwd | GGTTTCTGGTCATCATGGATTGGTTGAAGTGCTCCTACTACATG |
| M198_rev | ATCCATGATGACCAGAAACCCTCCTGGTTTAAGCAAACTTCCTAAG |
| C201_NDT_fwd | TCATCATGGATATGTTGAAGNDTTCCTACTACATGATTGGGG |
| C201_VHG_fwd | TCATCATGGATATGTTGAAGVHGTCCTACTACATGATTGGGG |
| C201_TGG_fwd | TCATCATGGATATGTTGAAGTGGTCCTACTACATGATTGGGG |
| C201_rev | CTTCAACATATCCATGATGACCAGAAACCCTCCTGGTTTAAG |
| F242_NDT_fwd | TCGAGGTTATTTCACAGTCCNDTAGCTCCACAATGGCAAATAAC |
| F242_VHG_fwd | TCGAGGTTATTTCACAGTCCVHGAGCTCCACAATGGCAAATAAC |
| F242_TGG_fwd | TCGAGGTTATTTCACAGTCCTGGAGCTCCACAATGGCAAATAAC |
| F242_rev | GGACTGTGAAATAACCTCGAACCATTCAATGGTGTACCCCG |
| S249_NDT_fwd | TCAGCTCCACAATGGCAAATNDTGAGGGCTTATTTTCGC |
| S249_VHG_fwd | TCAGCTCCACAATGGCAAATVHGGAGGGCTTATTTTCGC |
| S249_TGG_fwd | TCAGCTCCACAATGGCAAATTGGGAGGGCTTATTTTCGC |
| S249_rev | ATTTGCCATTGTGGAGCTGAAGGACTGTGAAATAACCTCGAAC |
| S213_NDT_fwd | TTGGGGAACAGAAGTTCTCANDTTTACCTTTGGGCCGTGAAG |
| S213_VHG_fwd | TTGGGGAACAGAAGTTCTCAVHGTTACCTTTGGGCCGTGAAG |
| S213_TGG_fwd | TTGGGGAACAGAAGTTCTCATGGTTACCTTTGGGCCGTGAAG |
| S213_rev | TGAGAACTTCTGTTCCCCAATCATGTAGTAGGAGCACTTCAACATATC |
| Y20_NDT_fwd | GTCATTTTAACCCGCGCGACNDTTTAGAAAAATACTACAAGTTTGGAAGCC |
| Y20_VHG_fwd | GTCATTTTAACCCGCGCGACVHGTTAGAAAAATACTACAAGTTTGGAAGCC |
| Y20_TGG_fwd | GTCATTTTAACCCGCGCGACTGGTTAGAAAAATACTACAAGTTTGGAAGCC |
| Y20_rev | GTCGCGCGGGTTAAAATGACTCAAATAAGTGTCCTTACTCGTAAATCC |
| Y24_NDT_fwd | CGCGCGACTACTTAGAAAAANDTTACAAGTTTGGAAGCCGC |
| Y24_VHG_fwd | CGCGCGACTACTTAGAAAAAVHGTACAAGTTTGGAAGCCGC |
| Y24_TGG_fwd | CGCGCGACTACTTAGAAAAATGGTACAAGTTTGGAAGCCGC |
| Y24_rev | TTTTTCTAAGTAGTCGCGCGGGTTAAAATGACTCAAATAAGTGTC |
| L164_NDT_fwd | CGGATTGTGTATTGTCAACCNDTTGCTTGCACGCAGC |
| L164_VHG_fwd | CGGATTGTGTATTGTCAACCVHGTGCTTGCACGCAGC |
| L164_TGG_fwd | CGGATTGTGTATTGTCAACCTGGTGCTTGCACGCAGC |
| L164_rev | GGTTGACAATACACAATCCGCCGGGGGCAGTGGG |
| D197_NDT_fwd | GAGGGTTTCTGGTCATCATGNDTATGTTGAAGTGCTCCTACTACATG |
| D197_VHG_fwd | GAGGGTTTCTGGTCATCATGVHGATGTTGAAGTGCTCCTACTACATG |
| D197_TGG_fwd | GAGGGTTTCTGGTCATCATGTGGATGTTGAAGTGCTCCTACTACATG |
| D197_rev | CATGATGACCAGAAACCCTCCTGGTTTAAGCAAACTTCCTAAGTTG |
| Y204_NDT_fwd | ATATGTTGAAGTGCTCCTACNDTATGATTGGGGAACAGAAGTTCT |
| Y204_VHG_fwd | ATATGTTGAAGTGCTCCTACVHGATGATTGGGGAACAGAAGTTCT |
| Y204_TGG_fwd | ATATGTTGAAGTGCTCCTACTGGATGATTGGGGAACAGAAGTTCT |
| Y204_rev | GTAGGAGCACTTCAACATATCCATGATGACCAGAAACCCTC |
| A247_NDT_fwd | AGTCCTTCAGCTCCACAATGNDTAATTCCGAGGGCTTATTTTC |
| A247_VHG_fwd | AGTCCTTCAGCTCCACAATGVHGAATTCCGAGGGCTTATTTTC |
| A247_TGG_fwd | AGTCCTTCAGCTCCACAATGTGGAATTCCGAGGGCTTATTTTC |
| A247_rev | CATTGTGGAGCTGAAGGACTGTGAAATAACCTCGAACCATTCAATG |

| **SSM *dre*-H-NMT 1st round** | **Sequence 5´→ 3´** |
|---|---|
| dreHNMT_V9_rev | CAAAGTCTTGAAAGGTGCTGCCATATGTAATCCTCCTGGTACCA |
| dreHNMT_V9_NDT | CAGCACCTTTCAAGACTTTGNDTGAGGATTATCCACGTTATCTTAAGTC |
| dreHNMT_V9_VHG | CAGCACCTTTCAAGACTTTGVHGGAGGATTATCCACGTTATCTTAAGTC |
| dreHNMT_V9_TGG | CAGCACCTTTCAAGACTTTGTGGGAGGATTATCCACGTTATCTTAAGTC |
| dreHNMT_Y147_rev | ATACAACATTTGTATCATATGGATGAAGTCCATCTTCTTCTCG |
| dreHNMT_Y147_NDT | ATATGATACAAATGTTGTATNDTGTGAAGGATCCTAACGCTAC |
| dreHNMT_Y147_VHG | ATATGATACAAATGTTGTATVHGGTGAAGGATCCTAACGCTAC |
| dreHNMT_Y147_TGG | ATATGATACAAATGTTGTATTGGGTGAAGGATCCTAACGCTAC |
| dreHNMT_C198_rev | TTGTGACATCTCTGTATAACACAGTTGCTTACGAAATGTAGTCC |
| dreHNMT_C198_NDT | GTTATACAGAGATGTCACAANDTGTGACCATAGGAGAGATC |
| dreHNMT_C198_VHG | GTTATACAGAGATGTCACAAVHGGTGACCATAGGAGAGATC |
| dreHNMT_C198_TGG | GTTATACAGAGATGTCACAATGGGTGACCATAGGAGAGATC |
| dreHNMT_F243_rev | GTCAAGCAGCAGCTCACCTTCTTGGTCACCTTCGGTGAAG |
| dreHNMT_F243_NDT | AAGGTGAGCTGCTGCTTGACNDTTTAACAGAGGTTAAGGAATTCTCAAAG |
| dreHNMT_F243_VHG | AAGGTGAGCTGCTGCTTGACVHGTTAACAGAGGTTAAGGAATTCTCAAAG |
| dreHNMT_F243_TGG | AAGGTGAGCTGCTGCTTGACTGGTTAACAGAGGTTAAGGAATTCTCAAAG |
| dreHNMT_E246_rev | GGTGACCAAGAAGGTGAGCTGCTGCTTGACTTCTTAACA |
| dreHNMT_E246_NDT | TGCTGCTTGACTTCTTAACANDTGTTAAGGAATTCTCAAAGAACG |
| dreHNMT_E246_VHG | TGCTGCTTGACTTCTTAACAVHGGTTAAGGAATTCTCAAAGAACG |
| dreHNMT_E246_TGG | TGCTGCTTGACTTCTTAACATGGGTTAAGGAATTCTCAAAGAACG |

| **SSM *dre*-H-NMT 2nd round** | **Sequence 5´→ 3´** |
|---|---|
| dreHNMT_Y15_rev | ACGTGGATAATCCTCAACCAAAGTCTTGAAAGGTGCTGCC |
| dreHNMT_Y15_NDT | TGGTTGAGGATTATCCACGTNDTCTTAAGTCGTTCGAGCTTTTCTTAG |
| dreHNMT_Y15_VHG | TGGTTGAGGATTATCCACGTVHGCTTAAGTCGTTCGAGCTTTTCTTAG |
| dreHNMT_Y15_TGG | TGGTTGAGGATTATCCACGTTGGCTTAAGTCGTTCGAGCTTTTCTTAG |
| dreHNMT_F19_rev | CGACTTAAGATAACGTGGATAATCCTCAACCAAAGTCTTGAAAGG |
| dreHNMT_F19_NDT | ATCCACGTTATCTTAAGTCGNDTGAGCTTTTCTTAGAGCGTTC |
| dreHNMT_F19_VHG | ATCCACGTTATCTTAAGTCGVHGGAGCTTTTCTTAGAGCGTTC |
| dreHNMT_F19_TGG | ATCCACGTTATCTTAAGTCGTGGGAGCTTTTCTTAGAGCGTTC |
| dreHNMT_F22_rev | AAGCTCGAACGACTTAAGATAACGTGGATAATCCTCAACCAAAG |
| dreHNMT_F22_NDT | ATCTTAAGTCGTTCGAGCTTNDTTTAGAGCGTTCGTCTGAAC |
| dreHNMT_F22_VHG | ATCTTAAGTCGTTCGAGCTTVHGTTAGAGCGTTCGTCTGAAC |
| dreHNMT_F22_TGG | ATCTTAAGTCGTTCGAGCTTTGGTTAGAGCGTTCGTCTGAAC |
| dreHNMT_E28_rev | AGACGAACGCTCTAAGAAAAGCTCGAACGACTTAAGATAACGTG |
| dreHNMT_E28_NDT | TTTTCTTAGAGCGTTCGTCTNDTCACCAATGCATGCAAGATTTC |
| dreHNMT_E28_VHG | TTTTCTTAGAGCGTTCGTCTVHGCACCAATGCATGCAAGATTTC |
| dreHNMT_E28_TGG | TTTTCTTAGAGCGTTCGTCTTGGCACCAATGCATGCAAGATTTC |
| dreHNMT_H29_rev | TTCAGACGAACGCTCTAAGAAAAGCTCGAACGACTTAAGATAACG |
| dreHNMT_H29_NDT | TCTTAGAGCGTTCGTCTGAANDTCAATGCATGCAAGATTTCATC |
| dreHNMT_H29_VHG | TCTTAGAGCGTTCGTCTGAAVHGCAATGCATGCAAGATTTCATC |
| dreHNMT_H29_TGG | TCTTAGAGCGTTCGTCTGAATGGCAATGCATGCAAGATTTCATC |
| dreHNMT_Q143_rev | TATCATATGGATGAAGTCCATCTTCTTCTCGGGGGTTTTCTC |
| dreHNMT_Q143_NDT | TGGACTTCATCCATATGATANDTATGTTGTATTACGTGAAGGATCC |
| dreHNMT_Q143_VHG | TGGACTTCATCCATATGATAVHGATGTTGTATTACGTGAAGGATCC |
| dreHNMT_Q143_TGG | TGGACTTCATCCATATGATATGGATGTTGTATTACGTGAAGGATCC |

## IV. Calibration curves

Calibration curves for the quantification of alkylated 5-bromo-1*H*-benzo[*d*]imidazole derivatives mediated through the enzymatic cascade employing engineered *dre*-H-NMT / *acl*-MT variants: All calibration curves for the panel of were made using nine serial dilutions of weighted standards and measured through HPLC-MS or GC-MS (see methods in section **O viii**).

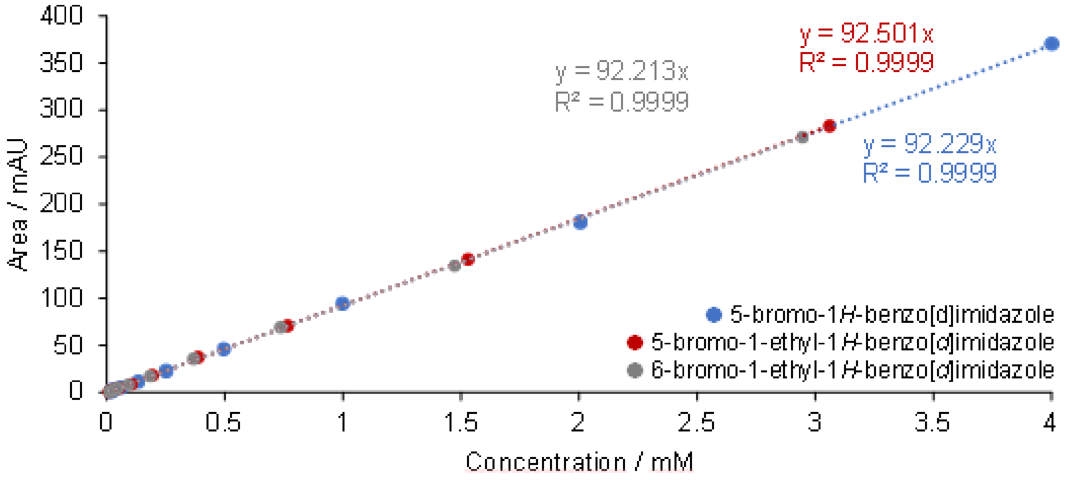


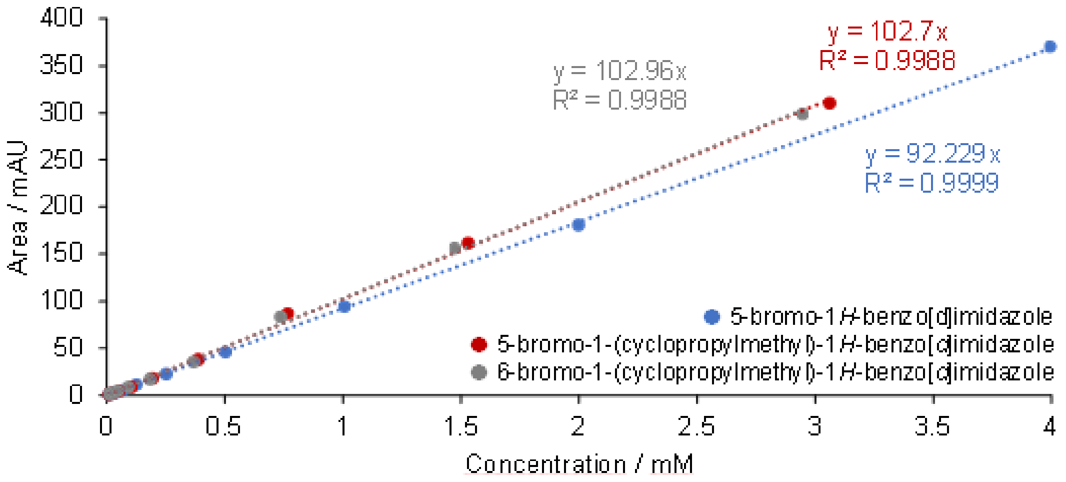


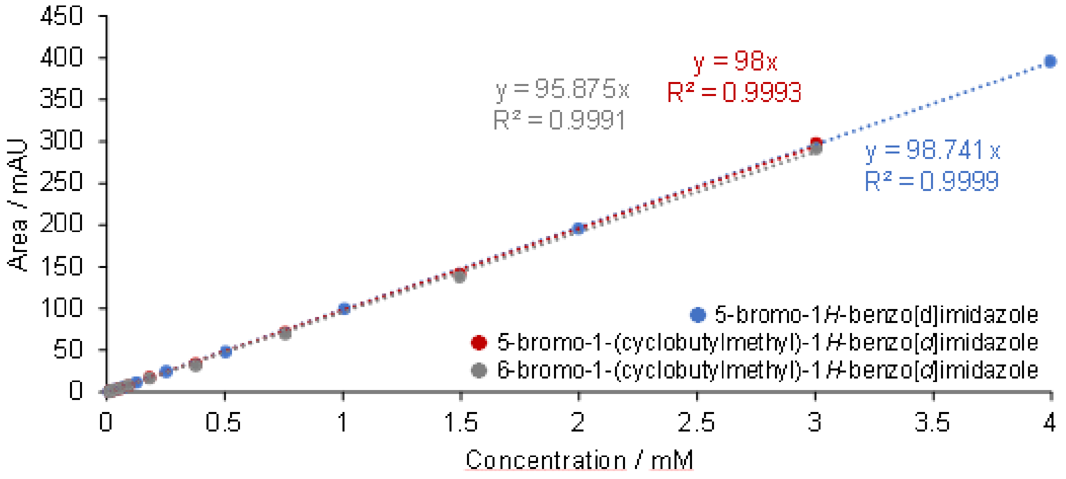

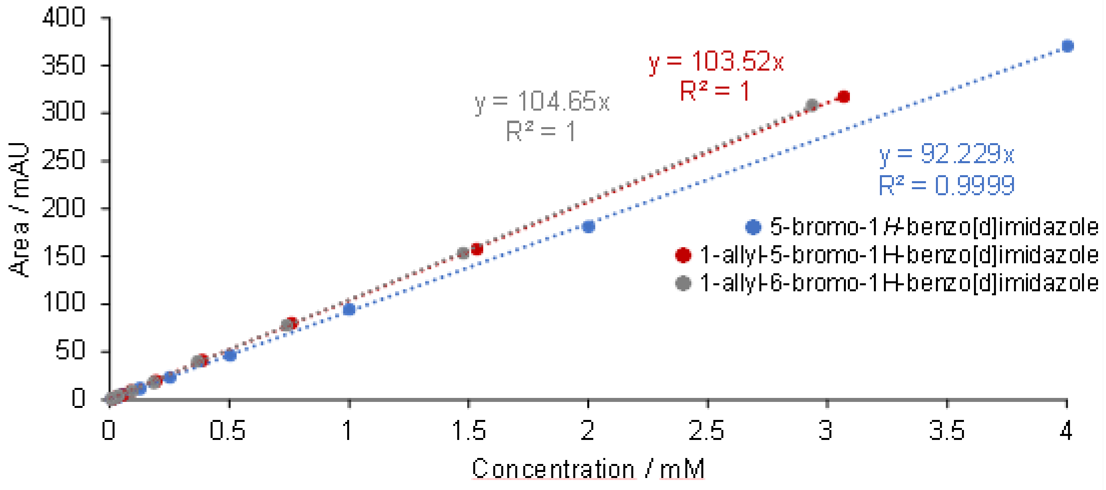
y = 103.52x
R² = 1
y = 104.65x
R² = 1
y = 92.229x
R² = 0.9999
5-bromo-1H-benzo[d]imidazole
1-allyl-5-bromo-1H-benzo[d]imidazole
1-allyl-6-bromo-1H-benzo[d]imidazole
Area / mAU
Concentration / mM

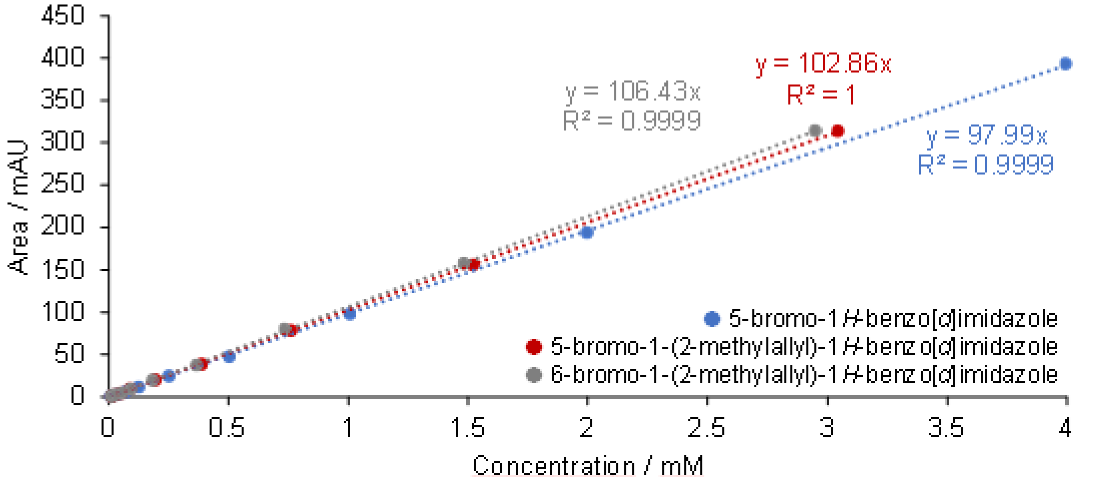
y = 102.86x
R² = 1
y = 106.43x
R² = 0.9999
y = 97.99x
R² = 0.9999
5-bromo-1H-benzo[d]imidazole
5-bromo-1-(2-methylallyl)-1H-benzo[d]imidazole
6-bromo-1-(2-methylallyl)-1H-benzo[d]imidazole
Area / mAU
Concentration / mM

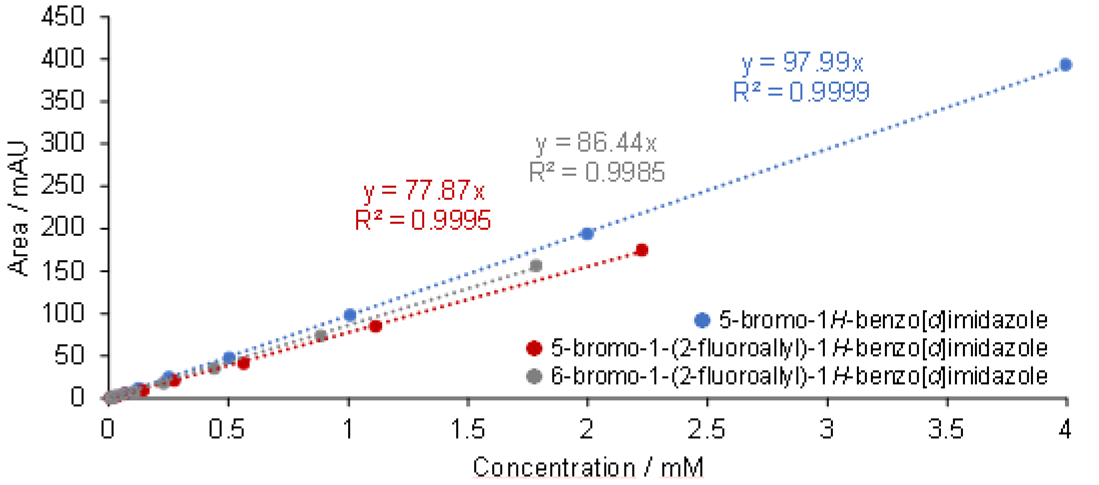
y = 97.99x
R² = 0.9999
y = 86.44x
R² = 0.9985
y = 77.87x
R² = 0.9995
5-bromo-1H-benzo[d]imidazole
5-bromo-1-(2-fluoroallyl)-1H-benzo[d]imidazole
6-bromo-1-(2-fluoroallyl)-1H-benzo[d]imidazole
Area / mAU
Concentration / mM

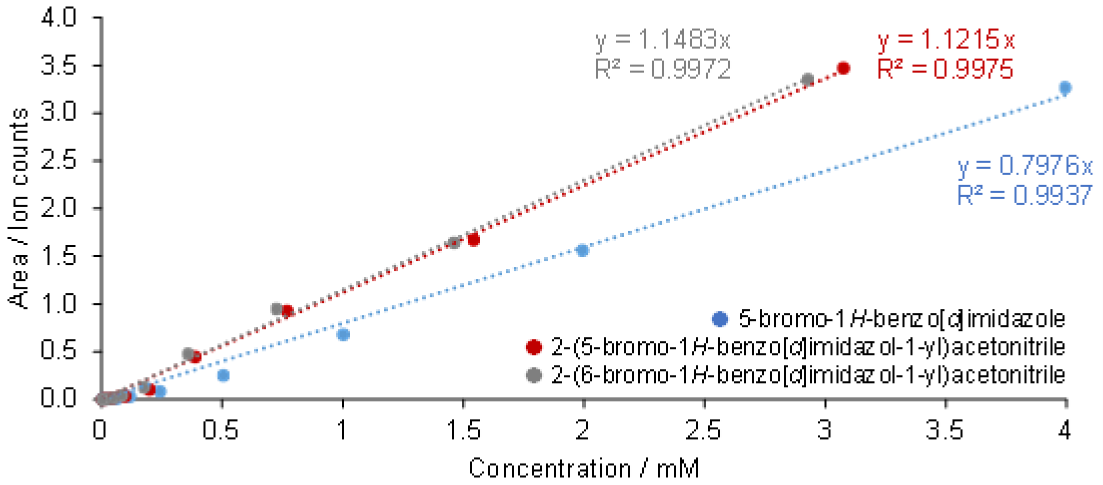

4.0
3.5
3.0
2.5
2.0
1.5
1.0
0.5
0.0
Area / Ion counts
y = 1.1483x
R² = 0.9972
y = 1.1215x
R² = 0.9975
y = 0.7976x
R² = 0.9937
5-bromo-1H-benzo[d]imidazole
2-(5-bromo-1H-benzo[d]imidazol-1-yl)acetonitrile
2-(6-bromo-1H-benzo[d]imidazol-1-yl)acetonitrile
0
0.5
1
1.5
2
2.5
3
3.5
4
Concentration / mM


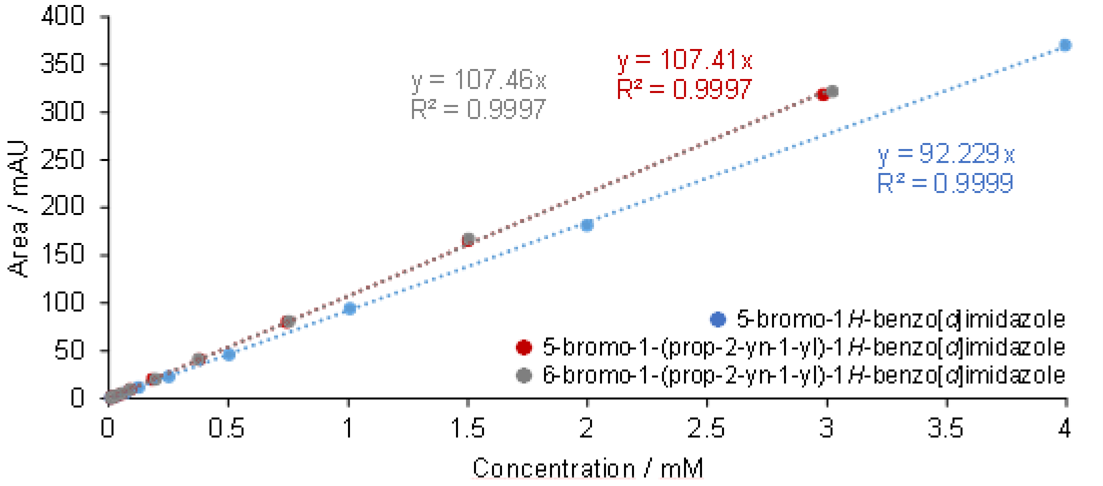

400
350
300
250
200
150
100
50
0
Area / mAU
y = 107.46x
R² = 0.9997
y = 107.41x
R² = 0.9997
y = 92.229x
R² = 0.9999
5-bromo-1H-benzo[d]imidazole
5-bromo-1-(prop-2-yn-1-yl)-1H-benzo[d]imidazole
6-bromo-1-(prop-2-yn-1-yl)-1H-benzo[d]imidazole
0
0.5
1
1.5
2
2.5
3
3.5
4
Concentration / mM

## V. Chemical synthesis of substrates and products standards

### General procedure for the synthesis of the alkylated product standards as regiosomeric mixtures.

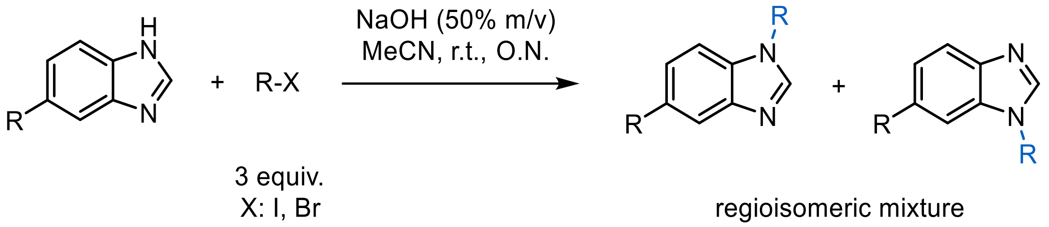


The methylated standards were synthesized according to a reported protocol.[33] In a round-bottom flask, the respective heterocycle (1 equiv.) was dissolved in MeCN (8.0 ml / mmol starting material). The resulting solution was stirred at 0°C, followed by the dropwise addition of a 50 % (m/v) NaOH solution (0.8 mL / mmol starting material). After 10 min, MeI (1.5 equiv.) was mixed in the flask. The reaction was allowed to reach room temperature. After 5 h, another addition of MeI (1.5 equiv.) was made, and the stirring continued overnight. The progress of the reaction was analyzed by thin layer chromatography. On the next day, the crude reaction was concentrated under reduced pressure. dd$H_2O$ (approx. 10 mL / mmol starting material) was added, and the crude mixture was extracted three times with EtOAc (1:1 v/v). The organic layers were combined and washed twice with dd$H_2O$ (1:1 v/v). The resulting organic phase was dried with $MgSO_4$, concentrated under reduced atmosphere, and purified by flash chromatography.

<u>5-Iodo-1-methyl-1*H*-benzo[*d*]imidazole, 6-Iodo-1-methyl-1*H*-benzo[*d*]imidazole mixture</u>:

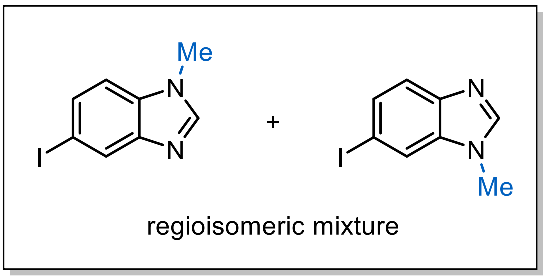


Methylation of 5-iodo-1*H*-benzo[*d*]imidazole (22.1 mg, 0.09 mmol) was made according to general procedure (**V**). After purification using EtOAc / MeOH (10:1 v/v) as mobile phase, the desired product mixture (16.2 mg, 62% yield) was obtained as a yellowish oil in a regioisomeric mixture of 48:52. $^1$H NMR (500 MHz, $CDCl_3$): δ 8.14 (d, $^4J_{H,H}$ = 1.4 Hz, 1H), 7.78 (s, 1H), 7.77 (s, 1H), 7.74 (s, 1H), 7.57 (dd, $^3J_{H,H}$ = 8.4 Hz, $^4J_{H,H}$ = 1.4 Hz, 1H), 7.53 - 7.55 (m, 2H), 7.14 (d, $^3J_{H,H}$ = 8.4 Hz, 1H), 3.80 (s, 3H), 3.79 (s, 3H); $^{13}$C NMR (126 MHz, $CDCl_3$): δ 145.7, 144.2, 144.0, 143.4, 136.3, 134.2, 131.6, 131.2, 129.4, 122.1, 118.7, 111.3, 86.6, 85.5, 31.3, 31.2.

5-Methoxy-1-methyl-1*H*-benzo[*d*]imidazole, 6-methoxy-1-methyl-1*H*-benzo[*d*]imidazole mixture:

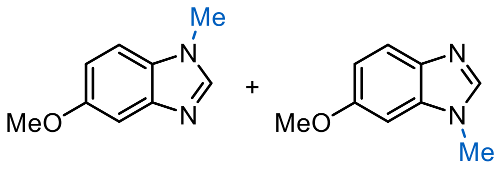

regioisomeric mixture

Methylation of 5-methoxy-1*H*-benzo[*d*]imidazole (207.4 mg, 1.40 mmol) was made according to general procedure (**V**). After purification using EtOAc / MeOH (10:1 v/v) as mobile phase, the desired product mixture (144.6 mg, 67% yield) was obtained as a pale cream solid in a regioisomeric mixture of 43:57. $^1$H NMR (600 MHz, $CDCl_3$): δ 7.75 (s, 1H), 7.71 (s, 1H), 7.64 (d, $^3J_{H,H}$ = 8.8 Hz, 1H), 7.25 (d, $^4J_{H,H}$ = 2.4 Hz, 1H), 7.21 (d, $^3J_{H,H}$ = 8.8 Hz, 1H), 6.93 (dd, $^3J_{H,H}$ = 8.8 Hz, $^4J_{H,H}$ = 2.4, 1H), 6.89 (dd, $^3J_{H,H}$ = 8.8 Hz, $^4J_{H,H}$ = 2.4 Hz, 1H), 6.78 (d, $^4J_{H,H}$ = 2.4 Hz, 1H), 3.85 (s, 3H), 3.84 (s, 3H), 3.75 (s, 3H), 3.73 (s, 3H); $^{13}$C NMR (151 MHz, $CDCl_3$): δ 156.9, 156.2, 144.6, 143.7, 142.8, 138.3, 135.2, 129.3, 120.8, 113.2, 111.5, 109.8, 102.3, 92.8, 55.9, 55.8, 31.1, 31.0.

5-Chloro-1-methyl-1*H*-benzo[*d*]imidazole, 6-chloro-1-methyl-1*H*-benzo[*d*]imidazole mixture:

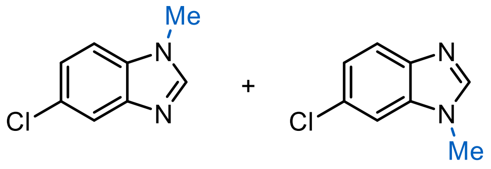

regioisomeric mixture

Methylation of 5-chloro-1*H*-benzo[*d*]imidazole (99.9 mg, 0.65 mmol) was made according to general procedure (**V**). After purification using EtOAc / MeOH (10:1 v/v) as mobile phase, the desired product mixture (72.0 mg, 66% yield) was obtained as a white solid in a regioisomeric mixture of 49:51. $^1$H NMR (500 MHz, $CDCl_3$): δ 7.85 (s, 1H), 7.84 (s, 1H), 7.77 (s, 1H), 7.70 (d, $^3J_{H,H}$ = 8.6 Hz, 1H), 7.38 (d, $^4J_{H,H}$ = 1.7 Hz, 1H), 7.23 – 7.30 (m, 3H), 3.83 (s, 3H), 3.81 (s, 3H); $^{13}$C NMR (126 MHz, $CDCl_3$): δ 144.6, 144.6, 144.3, 142.4, 135.2, 133.2, 128.85, 127.9, 123.5, 122.8, 121.2, 120.1, 110.1, 109.5, 31.2, 31.1.

5-Fluoro-1-methyl-1*H*-benzo[*d*]imidazole, 6-fluoro-1-methyl-1*H*-benzo[*d*]imidazole mixture:

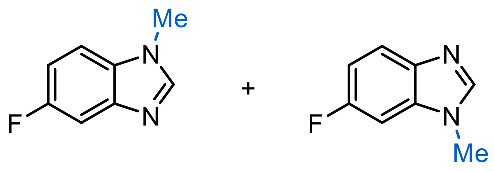

regioisomeric mixture

Methylation of 5-fluoro-1*H*-benzo[*d*]imidazole (50.9 mg, 0.37 mmol) was made according to general procedure (**V**). After purification using EtOAc / MeOH (10:1 v/v) as mobile phase, the desired product mixture (36.5 mg, 66% yield) was obtained as a white solid in a regioisomeric mixture of 50:50. $^1$H NMR (500 MHz, $CDCl_3$): δ 7.84 (s, 1H), 7.81 (s, 1H), 7.69 (dd, $^3J_{H,H}$ = 8.7, $^4J_{H,F}$ = 4.8 Hz, 1H), 7.44 (dd, $^3J_{H,F}$ = 9.4, $^4J_{H,H}$ = 2.3 Hz, 1H), 7.26 (dd, $^3J_{H,H}$ = 8.7, $^4J_{H,F}$ = 4.5 Hz, 1H), 6.97 - 7.07 (m, 3H), 3.80 (s, 3H), 3.76 (s, 3H); $^{13}$C NMR (126 MHz, $CDCl_3$): δ 160.0 (d, $J_{C,F}$ = 240.1 Hz), 159.4 (d, $J_{C,F}$ = 237.3 Hz), 144.9, 144.23 (d, $J_{C,F}$ = 12.8 Hz), 144.22 (d, $J_{C,F}$ = 3.1 Hz), 140.2, 134.8 (d, $J_{C,F}$ = 13.2 Hz), 131.2, 121.1 (d, $J_{C,F}$ = 10.1 Hz), 111.4 (d, $J_{C,F}$ = 26.4 Hz), 110.6 (d, $J_{C,F}$ = 25.3 Hz), 109.8 (d, $J_{C,F}$ = 10.4 Hz), 106.0 (d, $J_{C,F}$ = 24.1 Hz), 96.1 (d, $J_{C,F}$ = 27.3 Hz), 31.3, 31.2.

5-Bromo-1,2-dimethyl-1*H*-benzo[*d*]imidazole, 6-bromo-1,2-dimethyl-1*H*-benzo[*d*]imidazole mixture:

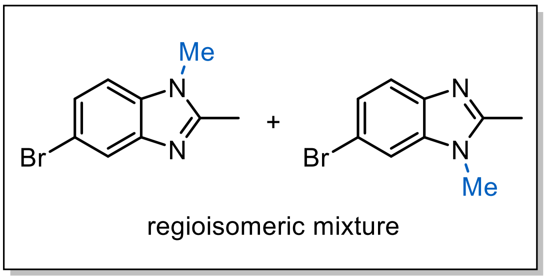


Methylation of 5-bromo-2-methyl-1*H*-benzo[*d*]imidazole (101.1 mg, 0.48 mmol) was made according to general procedure (**V**). After purification using EtOAc / MeOH (10:1 v/v) as mobile phase, the desired product mixture (87.6 mg, 82% yield) was obtained as a white solid in a regioisomeric mixture of 47:53. $^{1}$H NMR (600 MHz, $CDCl_3$): δ 7.79 (d, $^{4}J_{H,H}$ = 1.8 Hz, 1H), 7.52 (d, $^{3}J_{H,H}$ = 8.5 Hz, 1H), 7.41 (d, $^{4}J_{H,H}$ = 1.8 Hz, 1H), 7.32 (dd, $^{3}J_{H,H}$ = 8.5, $^{4}J_{H,H}$ = 1.8 Hz, 1H), 7.31 (dd, $^{3}J_{H,H}$ = 8.5, $^{4}J_{H,H}$ = 1.8 Hz, 1H), 7.12 (d, $^{3}J_{H,H}$ = 8.5 Hz, 1H), 3.68 (s, 3H), 3.67 (s, 3H), 2.58 (s, 3H), 2.57 (s, 3H); $^{13}$C NMR (151 MHz, $CDCl_3$): δ = 153.1, 152.7, 144.0, 141. 7, 137.0, 134.9, 125.2, 125.0 121.9, 120.4, 115.3, 114. 9, 112.2, 110.2, 30.13, 30.11, 13.98, 13.96.

1-Methyl-1*H*-benzo[*d*]imidazole-5-carbonitrile, 1-methyl-1*H*-benzo[*d*]imidazole-6-carbonitrile mixture:

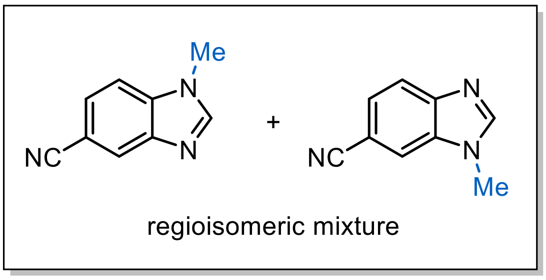


Methylation of 1*H*-benzo[*d*]imidazole-5-carbonitrile (98.9 mg, 0.69 mmol) was made according to general procedure (**V**). For the work-up, the concentrated reaction crude was mixed with $H_2O$ (20mL) and extracted twice with DCM (2 x 20 mL). The combined organic layers where combined, dried over $MgSO_4$ and concentrated under reduced pressure to afford the desired product mixture (108.4 mg, 98% yield) cream colored solid without further purification in a regioisomeric mixture of 41:59. $^{1}$H NMR (500 MHz, $CDCl_3$): δ 8.02 (d, $^{4}J_{H,H}$ = 1.4 Hz, 1H), 7.99 (s, 1H), 7.95 (s, 1H), 7.77 (d, $^{3}J_{H,H}$ = 8.4 Hz, 1H), 7.66 (d, $^{4}J_{H,H}$ = 1.3 Hz, 1H), 7.47 (dd, $^{3}J_{H,H}$ = 8.4, $^{4}J_{H,H}$ = 1.4 Hz, 1H), 7.44 (dd, $^{3}J_{H,H}$ = 8.4, $^{4}J_{H,H}$ = 1.4 Hz, 1H), 7.40 (d, $^{3}J_{H,H}$ = 8.4 Hz, 1H), 3.84 (s - overlapped, 3H), 3.83 (s - overlapped, 3H); $^{13}$C NMR (126 MHz, $CDCl_3$): δ 146.6, 146.5, 146.0, 143.2, 137.2, 134.1, 126.1, 125.4, 125.2, 121.2, 119.7, 119.7, 114.5, 110.6, 105.8, 105.2, 77.2, 31.3, 31.3.

1-Methyl-5-nitro-1*H*-benzo[*d*]imidazole, 1-methyl-6-nitro-1*H*-benzo[*d*]imidazole mixture:

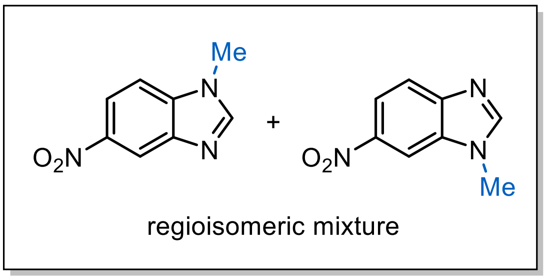


Methylation of 5-nitro-1*H*-benzo[*d*]imidazole (509.9 mg, 3.12 mmol) was made according to general procedure (**V**). The desired product mixture (486.3 mg, 90% yield) was obtained without purification as a sand colored solid in a regioisomeric mixture of 44:56. $^{1}$H NMR (600 MHz, $CDCl_3$): δ 8.70 (d, $^{4}J_{H,H}$ = 2.1 Hz, 1H), 8.37 (d, $^{4}J_{H,H}$ = 2.2 Hz, 1H), 8.25 (dd, $^{3}J_{H,H}$ = 8.9, $^{4}J_{H,H}$ = 2.1 Hz, 1H), 8.20 (dd, $^{3}J_{H,H}$ = 8.9, $^{4}J_{H,H}$ = 2.2 Hz, 1H), 8.12 (s, 1H), 8.06 (s, 1H), 7.85 (d, $^{3}J_{H,H}$ = 8.9 Hz, 1H), 7.46 (d, $^{3}J_{H,H}$ = 8.9 Hz, 1H), 3.96 (s, 3H), 3.93 (s, 3H); $^{13}$C NMR (151 MHz, $CDCl_3$): δ 148.1, 147.0, 144.03 143.9, 143.1, 138.7, 134.0, 120.6, 119.0, 118.1, 117.2, 109.6, 106.7, 31.7, 31.7.

1-Methyl-1*H*-benzo[*d*]imidazole-5-carbaldehyde, 1-methyl-1*H*-benzo[*d*]imidazole-6-carbaldehyde mixture:

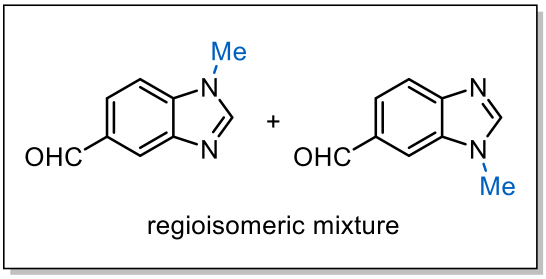


Methylation of 1*H*-benzo[*d*]imidazole-5-carbaldehyde (54.4 mg, 0.37 mmol) was made according to general procedure (**V**). After purification using EtOAc / MeOH (6:1 v/v) as mobile phase, the desired product mixture (44.5 mg, 81% yield) was obtained as a white solid in a regioisomeric mixture of 45:55. $^1$H NMR (500 MHz, $CDCl_3$): δ 10.06 (s, 1H), 10.05 (s, 1H), 8.25 (s, 1H), 8.01 (s, 1H), 7.96 (s, 1H), 7.93 (d, $^4J_{H,H}$ = 1.3 Hz, 1H), 7.863 (d, $^3J_{H,H}$ = 8.4 Hz, 1H), 7.860 (d, $^3J_{H,H}$ = 8.4 Hz, 1H), 7.78 (dd, $^3J_{H,H}$ = 8.4, $^4J_{H,H}$ = 1.3 Hz, 1H), 7.45 (d, $^3J_{H,H}$ = 8.4 Hz, 1H), 3.89 (s, 3H), 3.86 (s, 3H); $^{13}$C NMR (126 MHz, $CDCl_3$): δ 192.1, 191.9, 148.4, 147.0, 145.8, 143.6, 138.8, 134.8, 131.9, 131.7, 124.6, 124.4, 123.4, 120.7, 111.4, 110.2, 31.4, 31.4.

1-Methyl-1*H*-benzo[*d*]imidazol-5-amine, 1-methyl-1*H*-benzo[*d*]imidazol-6-amine mixture:

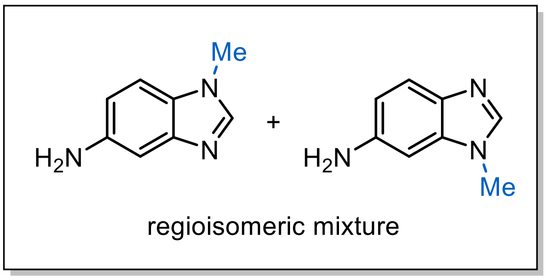


Reduction of the methylated 5-nitro-1*H*-benzo[*d*]imidazole regioisomeric mixture (386.9 mg, 2.18 mmol) was made according to a modified literature protocol[32] In a 100 mL round-bottom flask were refluxed under argon the methylated nitrobenzimidazole mixture with iron powder (856.1 mg, 15.33 mmol) and $HCl_{(aq)}$ (0.6 M, 3.2 mL) in EtOH (11.0 mL) overnight. The reaction crude was filtered through a pad of silica and eluted with EtOAc (250 mL). The organic phase was washed with saturated $K_2CO_{3(aq)}$ (100 mL) and brine (100 mL). The resulting organic layer was dried over $MgSO_4$ and concentrated under reduced pressure affording the desired product mixture (81.4 mg, 25% yield) as a dark red oil in a regioisomeric mixture of 29:71. $^1$H NMR (600 MHz, $CDCl_3$): δ 7.75 (s, 1H), 7.67 (s, 1H), 7.56 (d, $^3J_{H,H}$ = 8.5 Hz, 1H), 7.16 (d, $^3J_{H,H}$ = 8.5 Hz, 1H), 7.09 (d, $^4J_{H,H}$ = 2.1 Hz, 1H), 6.75 (dd, $^3J_{H,H}$ = 8.5, $^4J_{H,H}$ = 2.1 Hz, 1H), 6.68 (d, , $^3J_{H,H}$ = 8.5, $^4J_{H,H}$ = 2.1 Hz, 1H), 6.63 (dd, $^4J_{H,H}$ = 2.1 Hz, 1H), 3.77 (s, 3H), 3.72 (s, 3H); $^{13}$C NMR (151 MHz, $CDCl_3$): δ 144.9, 143.5, 143.1, 142.2, 142.1, 137.5, 135.8, 128.8, 120.9, 113.4, 112.4, 109.8, 105.2, 94.5, 31.2, 30.9.

5-Bromo-1-ethyl-1*H*-benzo[*d*]imidazole, 6-bromo-1-ethyl-1*H*-benzo[*d*]imidazole mixture:

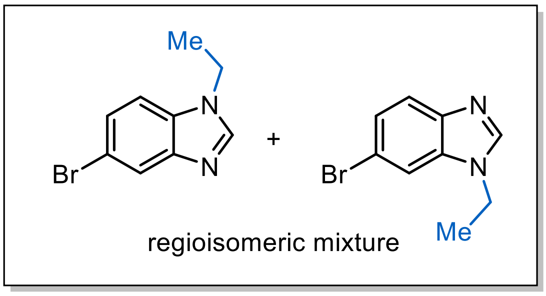


Ethylation of 5-bromo-1*H*-benzo[*d*]imidazole (100.7 mg, 0.52 mmol) was made according to general procedure (**V**) using iodoethane as alkylation reagent. Instead of EtOAc, DCM was used for extraction. The alkylation reagent was removed using flash chromatography DCM / MeOH (20:1 v/v) as mobile phase. The products were isolated by prep HPLC (see section **P i**), collected fractions were extracted three times 1:1 with EtOAc and after the solvent was removed under reduced pressure the desired product mixture was obtained as a yellowish oil (95.3 mg, 83% yield), analytical data is consistent with that reported in the literature.[34]

NMR of regioisomeric mixtures (51:49) used for calibration: $^{1}$H NMR (600 MHz, $CDCl_3$): δ 7.94 (d, $^{4}J_{H,H}$ = 1.8 Hz, 1H), 7.88 (d, $^{3}J_{H,H}$ = 6.8 Hz, 2H), 7.65 (d, $^{3}J_{H,H}$ = 8.6 Hz, 1H), 7.55 (d, $^{4}J_{H,H}$ = 1.8 Hz, 1H), 7.38 (ddd, $^{3}J_{H,H}$ = 13.4, 8.6, $^{4}J_{H,H}$ = 1.8 Hz, 2H), 7.26 (d, $^{3}J_{H,H}$ = 8.6 Hz, 1H), 4.19 (dq, $^{3}J_{H,H}$ = 10.7, 7.3 Hz, 5H), 1.52 (td, $^{3}J_{H,H}$ = 7.4, 0.9 Hz, 7H); $^{13}$C NMR (151 MHz, $CDCl_3$): δ 145.3, 143.3, 143.0, 142.9, 134.7, 132.6, 125.9, 125.5, 123.2, 121.7, 116.2, 115.2, 112.7, 110.8, 40.1 (d, J = 8.0 Hz), 15.2.

For identification of the enzymatic product configuration, 3 x 15 mg of the regiosiomeric mixture was separated on prep HPLC (see section **P ii**). The fractions contained TFA and were neutralized with 0.5 mL of 50% (w/v) NaOH, extracted 3 x with EtOAc (1:1 v/v) before the solvent was removed under reduced pressure affording an regioisomerically enriched product mixture (70:30, 5-bromo-1-ethyl-1*H*-benzo[*d*]imidazole : 6-bromo-1-ethyl-1*H*-benzo[*d*]imidazole).

NMR of 5-bromo-1-ethyl-1*H*-benzo[*d*]imidazole: $^{1}$H NMR (600 MHz, $CDCl_3$): δ 7.87 (d, $^{4}J_{H,H}$ = 1.8 Hz, 1H), 7.82 (s, 1H), 7.32 (dd, $^{3}J_{H,H}$ = 8.6, $^{4}J_{H,H}$ = 1.8 Hz, 1H), 7.20 (d, $^{3}J_{H,H}$ = 8.6 Hz, 1H), 4.13 (q, $^{3}J_{H,H}$ = 7.4 Hz, 2H), 1.46 (t, $^{3}J_{H,H}$ = 7.4, 1H). $^{13}$C NMR (151 MHz, $CDCl_3$): δ 144.2, 142.3, 131.6, 124.9, 122.2, 114.1, 109.8, 39.0 14.2.

NMR of 6-bromo-1-ethyl-1*H*-benzo[*d*]imidazole: $^{1}$H NMR (600 MHz, $CDCl_3$): δ 7.81 (s, 1H), 7.59 (d, *J* = 8.6 Hz, 1H), 7.49 (d, $^{4}J_{H,H}$ = 1.8 Hz, 1H), 7.30 (dd, $^{3}J_{H,H}$ = 8.6, $^{4}J_{H,H}$ = 1.8 Hz, 1H), 4.11 (q, $^{3}J_{H,H}$ = 7.4 Hz, 2H), 1.46 (t, $^{3}J_{H,H}$ = 7.4, 3H). $^{13}$C NMR (151 MHz, $CDCl_3$): δ 141.9, 141.9, 133.7, 124.4, 120.6, 115.1, 111.7, 39.0, 14.2. HRMS (ESI) (m/z) calculated for $[M+H]^+$ $C_9H_{10}BrN_2H^+$: 225.0027, found: 225.0023.

5-Bromo-1-(cyclopropylmethyl)-1*H*-benzo[*d*]imidazole, 6-bromo-1-(cyclopropylmethyl)-1*H*-benzo[*d*]imidazole mixture:

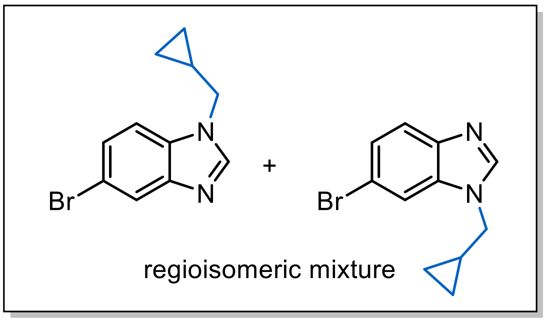


Methylcyclopropylation of 5-bromo-1*H*-benzo[*d*]imidazole (100.5 mg, 0.52 mmol) was made according to general procedure (**V**) using (iodomethyl)cyclopropane as alkylation reagent. Instead of EtOAc, DCM was used for the extraction. The alkylation reagent was removed using flash chromatography DCM / MeOH (20:1 v/v) as mobile. The products were isolated by prep HPLC (see section **P i**), collected fractions were extracted three times 1:1 with EtOAc and after the solvent was removed under reduced pressure affording the desired product mixture as a yellowish oil (106.8 mg, 83% yield).

NMR of regioisomeric mixtures (50:50) used for calibration: $^{1}$H NMR (600 MHz, $CDCl_3$): δ 7.99 (s, 1H), 7.97 (s, 1H), 7.94 (d, $^{4}J_{H,H}$ = 1.8 Hz, 1H), 7.66 (d, $^{3}J_{H,H}$ = 8.6 Hz, 1H), 7.57 (d, $^{4}J_{H,H}$ = 1.8 Hz, 1H), 7.39 (dd, $^{3}J_{H,H}$ = 8.6, $^{4}J_{H,H}$ = 1.8 Hz, 1H), 7.37 (dd, $^{4}J_{H,H}$ = 8.6, $^{4}J_{H,H}$ = 1.8 Hz, 1H), 7.28 (d, $^{3}J_{H,H}$ = 8.6 Hz, 1H), 3.98 (d, $^{3}J_{H,H}$ = 7.0 Hz, 2H), 3.96 (d, $^{3}J_{H,H}$ = 7.0 Hz, 2H), 1.30 (m, $^{3}J_{H,H}$ = 7.1 Hz, 2H), 0.71 (m, $^{3}J_{H,H}$ = 4.3 Hz, 4H), 0.41 (m, $^{3}J_{H,H}$ = 4.3 Hz, 4H); $^{13}$C NMR (151 MHz, $CDCl_3$): δ 145.3, 143.6, 143.3, 142.9, 135.2, 126.0, 125.5, 123.3, 121.7, 116.3, 115.3, 113.0, 111.0, 50.0, 49.9, 10.9, 10.9, 4.6, 4.6.

For identification of the enzymatic product configuration, 3 x 15 mg of the regiosiomeric mixture was separated on prep HPLC (see section **P ii**) and fractions for both regiosiomers were collected. The fractions contained TFA and were neutralized with 0.5 mL of 50% (w/v) NaOH, extracted 3 x with EtOAc (1:1 v/v) before the solvent was removed under reduced pressure.

NMR of 5-bromo-1-(cyclopropylmethyl)-1*H*-benzo[*d*]imidazole: $^{1}$H NMR (600 MHz, $CDCl_3$): δ 8.02 (s, 1H), 7.95 (d, $^{4}J_{H,H}$ = 1.8 Hz, 1H), 7.39 (dd, $^{3}J_{H,H}$ = 8.6, $^{4}J_{H,H}$ = 1.8 Hz, 1H), 7.29 (d, $^{3}J_{H,H}$ = 8.6 Hz, 1H), 3.99 (d, $^{3}J_{H,H}$ = 7.0 Hz, 2H), 1.36 – 1.20 (m, 1H), 0.75 – 0.66 (m, 2H), 0.41 (q, $J$ = 5.3 Hz, 2H); $^{13}$C NMR (151 MHz, $CDCl_3$): δ 145.1, 143.6, 133.1, 126.1, 123.2, 115.3, 111.1, 50.0, 10.9, 4.6.

NMR of 6-bromo-1-(cyclopropylmethyl)-1*H*-benzo[*d*]imidazole: $^{1}$H NMR (600 MHz, $CDCl_3$): δ 7.97 (s, 1H), 7.66 (d, $^{3}J_{H,H}$ = 8.6 Hz, 1H), 7.57 (d, $^{4}J_{H,H}$ = 1.8 Hz, 1H), 7.37 (dd, $^{3}J_{H,H}$ = 8.6, $^{4}J_{H,H}$ = 1.8 Hz, 1H), 3.96 (d, $^{3}J_{H,H}$ = 7.1 Hz, 2H), 1.34 – 1.27 (m, 1H), 0.72 (t, $J$ = 5.7 Hz, 1H), 0.41 (t, $J$ = 5.3 Hz, 2H); $^{13}$C NMR (151 MHz, $CDCl_3$): δ 143.3, 143.0, 135.2, 125.5, 121.8, 116.3, 113., 49.9, 10.9, 4.6. HRMS (ESI) (m/z) calculated for $[M+H]^+$ $C_{11}H_{12}BrN_2H^+$: 251.0184, found: 251.0177.

5-Bromo-1-(cyclobutylmethyl)-1*H*-benzo[*d*]imidazole, 6-bromo-1-(cyclobutylmethyl)-1*H*-benzo[*d*]imidazole mixture:

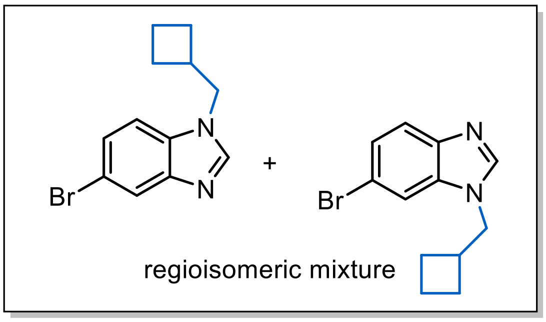


Methylcyclobutylation of 5-bromo-1*H*-benzo[*d*]imidazole (100.9 mg, 0.52 mmol) was made according to general procedure (**V**) using (bromomethyl)cyclobutene as alkylation reagent. Instead of EtOAc, DCM was used for the extraction. The alkylation reagent was removed using flash chromatography DCM / MeOH (20:1 v/v) as mobile phase. The products were isolated by prep HPLC (see section **P i**), collected fractions were extracted three times 1:1 with EtOAc and after the solvent was removed under reduced pressure affording the desired product mixture as a yellowish oil (98.4 mg, 83% yield).

NMR of regioisomeric mixtures (52:48) used for calibration: $^{1}$H NMR (600 MHz, $CDCl_3$): δ 7.94 (d, $^{4}J_{H,H}$ = 1.8 Hz, 1H), 7.85 (s, 1H), 7.84 (s, 1H), 7.65 (d, $^{3}J_{H,H}$ = 8.6 Hz, 1H), 7.54 (d, $^{4}J_{H,H}$ = 1.8 Hz, 1H), 7.38 (ddd, $^{3}J_{H,H}$ = 8.6, $^{4}J_{H,H}$ = 1.8 Hz, 2H), 7.26 (d, $^{3}J_{H,H}$ = 8.6 Hz, 1H), 4.13 (d, $^{3}J_{H,H}$ = 7.3 Hz, 2H), 4.11 (d, $^{3}J_{H,H}$ = 7.4 Hz, 2H), 2.85 (m, 2H), 2.09 (m, 4H), 1.94 (s, 4H), 1.79 (d, 4H); $^{13}$C NMR (151 MHz, $CDCl_3$): δ 145.1, 143.8, 143.5, 142.8, 135.2, 133.1, 126.0, 125.5, 123.3, 121.7, 116.3, 115.2, 113.0, 111.0, 50.5, 35.4, 35.3, 26.3, 18.2.

Through silica column (pure DCM), a fraction of pure 5-bromo-1-(cyclobutylmethyl)-1*H*-benzo[*d*]imidazole was obtained: $^{1}$H NMR (600 MHz, $CDCl_3$): δ 7.93 (d, $^{4}J_{H,H}$ = 1.8 Hz, 1H), 7.85 (s, 1H), 7.39 (dd, $^{3}J_{H,H}$ = 8.6, $^{4}J_{H,H}$ = 1.8 Hz, 1H), 7.26 (d, $^{3}J_{H,H}$ = 8.6 Hz, 1H), 4.13 (d, $^{3}J_{H,H}$ = 7.3 Hz, 2H), 2.84 (s, 1H), 2.08 (m, 2H), 1.94 (m, 2H), 1.81 (m, 2H); $^{13}$C NMR (151 MHz, $CDCl_3$): δ 145.2, 143.8, 133.2, 126.0, 123.3, 115.2, 111.1, 50.5, 35.4, 26.3, 18.2. HRMS (ESI) (m/z) calculated for $[M+H]^+$ $C_{12}H_{14}BrN_2H^+$: 265.0340, found: 265.0337.

1-allyl-5-bromo-1*H*-benzo[*d*]imidazole, 1-allyl-6-bromo-1*H*-benzo[*d*]imidazole mixture:

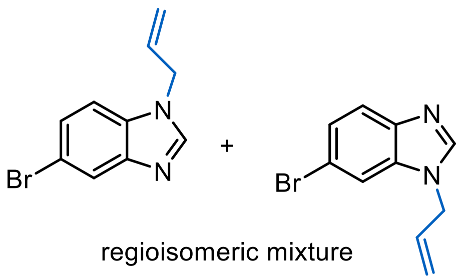


Allylation of 5-bromo-1*H*-benzo[*d*]imidazole (100.1 mg, 0.52 mmol) was made according to general procedure (**V**) using 3-bromoprop-1-ene as alkylation reagent. Instead of EtOAc, DCM was used for the extraction. The alkylation reagent was removed using flash chromatography DCM / MeOH (20:1 v/v) as mobile phase. The products were isolated by prep HPLC (see section **P i**), collected fractions were extracted three times 1:1 with EtOAc and after the solvent was removed under reduced pressure affording the desired product mixture as a yellowish oil (94.2 mg, 78% yield).

NMR of regioisomeric mixtures (50:50) used for calibration: $^{1}$H NMR (600 MHz, $CDCl_3$): δ 7.95 (d, $^{4}J_{H,H}$ = 1.8 Hz, 1H), 7.88 (s, 1H), 7.87 (s, 1H), 7.66 (d, $^{3}J_{H,H}$ = 8.6 Hz, 1H), 7.52 (d, $^{4}J_{H,H}$ = 1.8 Hz, 1H), 7.38 (m, 2H), 7.24 (d, $^{3}J_{H,H}$ = 8.5 Hz, 1H), 5.98 (m, 2H), 5.32 (m, 2H), 5.18 (m, 2H), 4.75 (m, 4H); $^{13}$C NMR (151 MHz, $CDCl_3$): δ 145.3, 144.0, 143.7, 143.0, 135.1, 133.0, 131.6, 131.5, 126.2, 125.7, 123.4, 121.8, 119.2, 119.1, 116.4, 115.5, 47.7 (d, *J* = 9.3 Hz).

5-bromo-1-(2-methylallyl)-1*H*-benzo[*d*]imidazole, 6-bromo-1-(2-methylallyl)-1*H*-benzo[*d*]imidazole mixture:

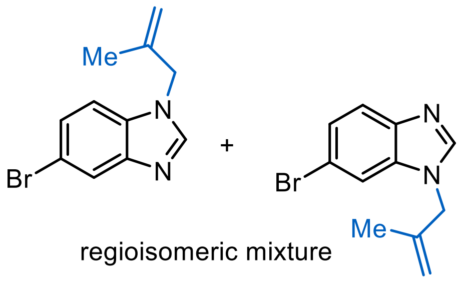


2-Methylallylation of 5-bromo-1*H*-benzo[*d*]imidazole (103.1 mg, 0.53 mmol) was made according to general procedure (**V**) using 3-bromo-2-methylprop-1-ene as alkylation reagent. Instead of EtOAc, DCM was used for the extraction. The alkylation reagent was removed using flash chromatography DCM / MeOH (20:1 v/v) as mobile phase. The products were isolated by prep HPLC (see section **P i**), collected fractions were extracted three times 1:1 with EtOAc and after the solvent was removed under reduced pressure affording the desired product mixture as a yellowish oil (99.8 mg, 76% yield).

NMR of regioisomeric mixtures (50:50) used for calibration: $^{1}$H NMR (600 MHz, $CDCl_3$): δ 7.95 (d, $^{4}J_{H,H}$ = 1.8 Hz, 1H), 7.87 (s, 1H), 7.86 (s, 1H), 7.66 (d, $^{3}J_{H,H}$ = 8.6 Hz, 1H), 7.51 (d, $^{4}J_{H,H}$ = 1.8 Hz, 1H), 7.38 (m, 2H), 7.24 (d, $^{3}J_{H,H}$ = 8.6 Hz, 1H), 5.01 (m, 2H), 4.81 (s, 2H), 4.66 (d, *J* = 11.9 Hz, 4H), 1.70 (m, 6H); $^{13}$C NMR (151 MHz, $CDCl_3$): δ 145.2, 144.3, 144.0, 142.8, 139.2, 139.0, 135.1, 133.0, 126.1, 125.6, 123.2, 121.7, 116.4, 115.3, 114.4, 114.3, 113.2, 111.4, 51.3, 51.2, 19.8, 19.8.

5-bromo-1-(2-fluoroallyl)-1*H*-benzo[*d*]imidazole, 6-bromo-1-(2-fluoroallyl)-1*H*-benzo[*d*]imidazole mixture:

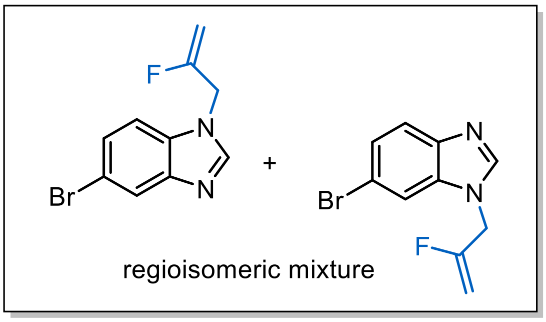


2-fluoroallylation of 5-bromo-1*H*-benzo[*d*]imidazole (80 mg, 0.41 mmol) was made according to general procedure (**V**) using 3-bromo-2-fluoroprop-1-ene as alkylation reagent. Instead of EtOAc, DCM was used for the extraction. The alkylation reagent was removed using flash chromatography DCM / MeOH (20:1 v/v) and further purified using preparative TLC (DCM - 1x and DCM:MeOH 95:5 - 2x). The desired product mixture was obtained as a yellowish oil (4.3 mg, 4% yield).

NMR of regioisomeric mixtures (56:44) used for calibration: $^{1}$H NMR (600 MHz, $CDCl_3$): δ 7.97 (d, $^{4}J_{H,H}$ = 1.8 Hz, 1H), 7.92 (s, 1H), 7.91 (s, 1H), 7.68 (d, $^{3}J_{H,H}$ = 8.6 Hz, 1H), 7.57 (d, $^{4}J_{H,H}$ = 1.8 Hz, 1H), 7.44 (dd, $^{3}J_{H,H}$ = 8.6, $^{4}J_{H,H}$ = 1.8 Hz, 1H), 7.41 (dd, $^{3}J_{H,H}$ = 8.6, 1.8 Hz, 1H), 7.29 (d, $^{3}J_{H,H}$ = 8.6 Hz, 1H), 4.84 – 4.9 (m, 2H), 4.45 – 4.57 (m, 2H); $^{13}$C NMR (151 MHz, $CDCl_3$): δ 160.0, 159.97, 158.3, 158.1, 145.3, 144.2, 143.8, 142.9, 134.8, 132.7, 126.7, 126.2, 123.7, 122.1, 116.9, 115.9, 113.0, 111.1, 94.4 (d, *J* = 17.0 Hz), 94.3 (d, *J* = 16.9 Hz), 45.6 (dd, *J* = 9.7, 2.5 Hz), 45.5 – 45.3 (m).

5-bromo-1-(prop-2-yn-1-yl)-1*H*-benzo[*d*]imidazole, 6-bromo-1-(prop-2-yn-1-yl)-1*H*-benzo[*d*]imidazole mixture:

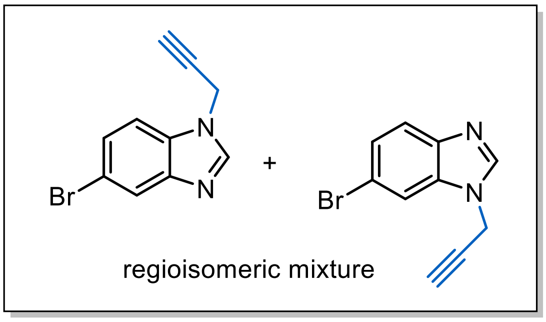


Propargylation of 5-bromo-1*H*-benzo[*d*]imidazole (80 mg, 0.41 mmol) was made according to general procedure (**V**) using 3-bromoprop-1-yne as alkylation reagent. Instead of EtOAc, DCM was used for the extraction and DCM / MeOH (20:1 v/v) was used as mobile phase for purification. The products were isolated by prep HPLC (see section **P i**), followed by extraction 3x 1:1 with EtOAc and after the solvent was removed under reduced pressure affording the desired product mixture as a yellowish oil (67.0 mg, 70% yield).

NMR of regioisomeric mixtures (50:50) used for calibration: $^{1}$H NMR (600 MHz, $CDCl_3$): δ 8.00 (s, 1H), 7.98 (s, 1H), 7.96 (d, $^{4}J_{H,H}$ = 1.8 Hz, 1H), 7.67 (d, $^{3}J_{H,H}$ = 8.6 Hz, 1H), 7.64 (d, $^{4}J_{H,H}$ = 1.8 Hz, 1H), 7.44 (dd, $^{3}J_{H,H}$ = 8.6, $^{4}J_{H,H}$ = 1.8 Hz, 1H), 7.41 (dd, $^{3}J_{H,H}$ = 8.6, $^{4}J_{H,H}$ = 1.8 Hz, 1H), 7.36 (d, $^{3}J_{H,H}$ = 8.6 Hz, 1H), 4.91 (d, $^{4}J_{H,H}$ = 2.6 Hz, 2H), 4.89 (d, $^{4}J_{H,H}$ = 2.6 Hz, 2H), 2.53 (t, $^{4}J_{H,H}$ = 2.6 Hz, 1H), 2.52 (t, $^{4}J_{H,H}$ = 2.6 Hz, 1H); $^{13}$C NMR (151 MHz, $CDCl_3$): δ 145.2, 143.3, 142.9 (d, *J* = 3.1 Hz), 134.4, 132.3, 126.4, 126.0, 123.4, 121.8, 116.7, 112.9, 111.0, 75.5, 75.4, 75.4, 75.3, 34.9, 34.8.

2-(5-bromo-1*H*-benzo[*d*]imidazol-1-yl)acetonitrile, 2-(6-bromo-1*H*-benzo[*d*]imidazol-1-yl)acetonitrile mixture:

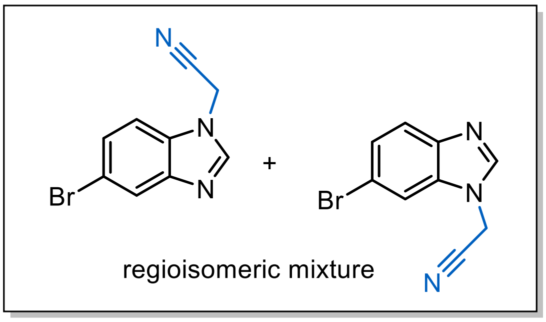


Alkylation of 5-bromo-1*H*-benzo[*d*]imidazole (99.4 mg, 0.51 mmol) with 2-bromoacetonitrile was made according to general procedure (**V**). Instead of EtOAc, DCM was used for the extraction and purification using DCM / MeOH (20:1 v/v) as mobile phase. After the solvent was removed under reduced pressure the desired product mixture was obtained as a yellowish oil (56.9 mg, 48% yield).

NMR of regioisomeric mixtures (65:1) used for calibration: $^{1}$H NMR (600 MHz, $CD_3OD$): δ 8.27 (s, 1H), 8.26 (s, 1H), 7.91 (d, $^{4}J_{H,H}$ = 1.8 Hz, 1H), 7.88 (d, $^{4}J_{H,H}$ = 1.8 Hz, 1H), 7.6 – 7.65 (m, 2H), 7.54 (dd, $^{3}J_{H,H}$ = 8.6, $^{4}J_{H,H}$ = 1.8 Hz, 1H), 7.49 (dd, $^{3}J_{H,H}$ = 8.6, $^{4}J_{H,H}$ = 1.8 Hz, 1H), 5.51 (d, *J* = 6.8 Hz, 4H); $^{13}$C NMR (151 MHz, $CD_3OD$): δ 145.9, 145.4, 145.4, 143.1, 135.4, 133.4, 128.1, 127.8, 123.4, 122.1, 118.3, 117.4, 115.5, 115.4, 114.5, 112.8, 33.5, 33.5.

**Synthesis and characterization of the late-stage methylation products and intermediates:**

5-(7*H*-Pyrrolo[2,3-*d*]pyrimidin-4-yl)-4,5,6,7-tetrahydro-3*H*-imidazo[4,5-*c*]pyridine (**12**):

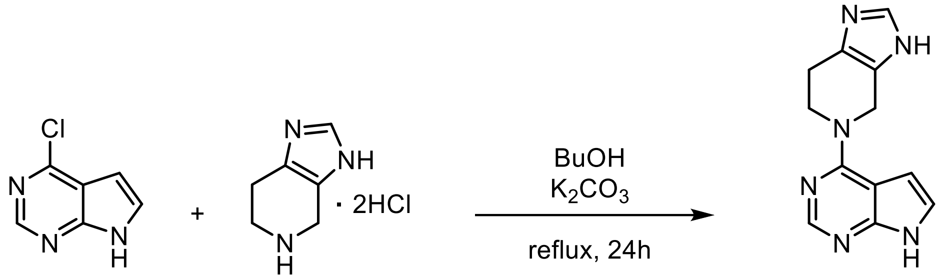


1.2 equiv.

1.2 g
79% yield

In a 50 mL round-bottom flask, 4-chloro-7*H*-pyrrolo[2,3-*d*]pyrimidine (1 g, 6.51 mmol), the dihydrochloride amine (1.5 g, 7.83 mmol, 1.2 equiv.), and $K_2CO_3$ (2.7 g, 19.7 mmol, 3 equiv.) were mixed with BuOH (30 mL) and a small amount of $H_2O$ (1 mL). The resulting turbid mixture was heated to reflux, turning deep yellow. After 24 h, the reaction was stopped by removing the BuOH with a rotary evaporator followed by filtration of the resulting reaction crude through a pad of silica using MeOH as eluent. The filtrate was purified by column chromatography using a DCM / MeOH (9:1 v/v) mixture as the mobile phase to afford the product (**12**, 1.2 g, 79% yield) as a white solid. TLC (DCM / MeOH, 9:1 v/v): $R_f$ = 0.29; $^1$H NMR (500 MHz, $D_2O$): δ 8.66 (s, 1H; H-2), 8.40 (s, 1H; H-2´), 7.46 (d, $^3J_{H,H}$ = 3.7 Hz, 1H; H-6´), 7.01 (d, $^3J_{H,H}$ = 3.7 Hz, 1H; H-5´), 5.24 (s, 2H; H-4), 4.38 (t, $^3J_{H,H}$ = 3.7 Hz, 2H; H-6), 3.13 (t, $^3J_{H,H}$ = 3.7 Hz, 2H; H-7); $^{13}$C NMR (126 MHz, $D_2O$): δ 153.8 (C-4´), 143.9 (C-7a´), 142.4 (C-2´), 133.1 (C-2), 126.3 (C-7a), 124.6 (C-6´), 123.1 (C-3a), 103.6 (C-5´), 102.7 (C-4a´), 44.9 (C-6), 43.5 (C-4), 20.6 (C-7). HRMS (ESI) (m/z) calculated for $[M+H]^+$ $C_{12}H_{12}N_6H^+$: 241.11962, found: 241.1207.

Synthesis of mono- and di-methylated mixtures of compound (**12**) for HPLC method development:

In a 5 mL pear shape flask compound **12** (20 mg, 0.08 mmol) were mixed with MeCN (2 mL), then a NaOH solution (50% (w/v), 200 µL) was added at 0 °C. The reaction was stirred for 10 min at 0°C before the addition of MeI (6 µL, 0.09 mmol). It was stirred at r.t. for 24 h. The solvent was removed using a rotary evaporator and the crude reaction was cleaned by column chromatography using a DCM / MeOH (9:1 v/v) mixture. A yellowish oil (7.4 mg) was obtained as a mixture of mainly di-methylated products. To obtain a mixture of mainly mono-methylated products, compound **12** (10 mg, 0.04 mmol) was dissolved in DMSO (800 µL) in a 2 mL screw top vial and MeI (2.5 µL, 0.04 mmol) was added. The reaction was stirred at r.t. overnight. The product mixture from this reaction was combined without further purification with the di-methylated mixtures previously obtained and used to develop screening and rescreening HPLC-DAD/MS methods. The HPLC traces are shown in **Figure 3**.

4-Chloro-7-methyl-7*H*-pyrrolo[2,3-*d*]pyrimidine:

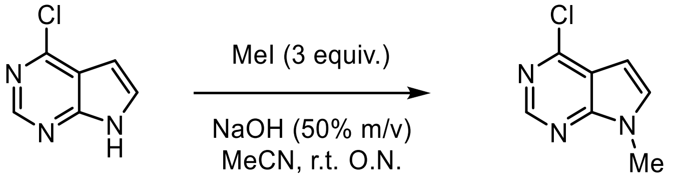


95% yield

Methylation of 4-chloro-7*H*-pyrrolo[2,3-*d*]pyrimidine (1.0 g, 6.53 mmol) was made according to general procedure (**V**). After purification using EtOAc / MeOH (6:1 v/v) as mobile phase, the desired product (1.04 g, 95% yield) was obtained as a yellowish solid. $^1$H NMR (600 MHz, $CDCl_3$): δ 8.58 (s, 1H; H-2), 7.15 (d, $^3J_{H,H}$ = 3.5 Hz, 1H; H-6), 6.54 (d, $^3J_{H,H}$ = 3.5 Hz, 1H; H-5), 3.83 (s, 3H; $NC\underline{H}_3$); $^{13}$C NMR (151 MHz, $CDCl_3$): δ 152.1 (C-4), 151.2 (C-7a), 150.7 (C-2), 130.1 (C-6), 117.5 (C-4a), 99.4 (C-5), 31.6 ($-N\underline{C}H_3$).

5-(7-methyl-7*H*-pyrrolo[2,3-*d*]pyrimidin-4-yl)-4,5,6,7-tetrahydro-3*H*-imidazo[4,5-*c*]pyridine:

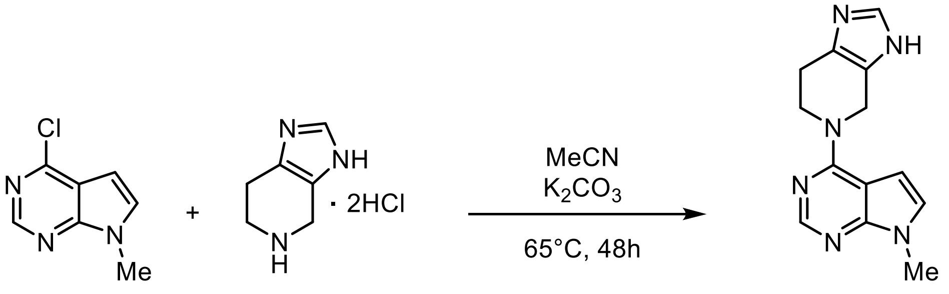


1 equiv.

9 mg
30% yield

In a 10 mL round-bottom flask were mixed 4-chloro-7-methyl-7*H*-pyrrolo[2,3-*d*]pyrimidine (19.5 mg, 0.13 mmol), the dihydrochloride amine (22.8 mg, 0.12 mmol, 1 equiv.) and $K_2CO_3$ (48.6 mg, 0.35 mmol, 3 equiv.) with MeCN (3 mL). The resulting suspension was heated at 65°C for 2 days (the reaction did not reach full conversion). The reaction crude was filtered through a column of silica packed with DCM and eluted using 2 column volumes of DCM followed by 4 column volumes of a DCM / MeOH (10:1 v/v) mixture. The purified product (9 mg, 30% yield) was obtained as a dark yellow solid. TLC (DCM / MeOH, 10:1 v/v): $R_f$ = 0.22; $^1$H NMR (600 MHz, $CDCl_3$): δ 8.36 (s, 1H; H-2´), 7.57 (s, 1H; H-2), 6.92 (d, $^3J_{H,H}$ = 3.6 Hz, 1H; H-6´), 6.58 (d, $^3J_{H,H}$ = 3.6 Hz, 1H; H-5´), 5.00 (t, $^5J_{H,H}$ = 1.5 Hz, 2H; H-4), 4.26 (t, $^3J_{H,H}$ = 5.6 Hz, 2H; H-6), 3.78 (s, 3H; $NC\underline{H}_3$), 2.88 (tt, $^3J_{H,H}$ = 5.6 Hz, $^5J_{H,H}$ = 1.5 Hz, 2H; H-7); $^{13}$C NMR (151 MHz, $CDCl_3$): δ 157.5 (C-4´), 151.4 (C-7a´), 151.1 (C-2´), 134.1 (C-2), 130.4 (C-3a), 126.0 (C-7a), 124.9 (C-6´), 103.5 (C-4a´), 100.7 (C-5´), 45.4 (C-4), 43.6 (C-6), 31.5 ($-N\underline{C}H_3$), 22.7 (C-7). HRMS (ESI) (m/z) calculated for $[M+H]^+$ $C_{13}H_{14}N_6H^+$: 255.13527, found: 255.1362.

## VI. Enzymatic preparative scale reactions

**General procedure for the preparative scale reactions using the cyclic two-enzyme cascade for the selective methylation of functionalized benzimidazoles:** To a Schott flask (100, 250 or 500 mL), the substrate (0.15 – 0.40 mmol, 2 mM final concentration) and SAH (10 µM, 1 mol%) were dissolved in a KPi buffer (50 mM, pH 7.0) containing 1% (v/v) *i*-PrOH as cosolvent. The mixture was shaken until getting a homogenous solution. When needed, the reaction flask was briefly immersed in an ultrasound bath (intervals of 10 seconds) until a clear solution, or a fine dispersion was obtained. To the resulting mixture, the purified enzymes (1 mol% *acl*-MT and 1 mol% of the respective NMT), previously thawed, were incorporated, followed by the addition of a MeI solution in *i*-PrOH (3 equiv. MeI, for a final 2% (v/v) *i*-PrOH). The reaction flask was covered with aluminum foil and shaken (in laid down position) using a Fisherbrand plate-shaker at 150 rpm and room temperature for 24 to 72 h. The reaction was extracted with EtOAc (1:1 v/v) three times or until the product was no longer detected in the aqueous phase. The organic layers were combined and concentrated using a rotary evaporator. The resulting reaction crude was purified by flash chromatography using DCM / MeOH (40:1) as the mobile phase (unless otherwise described).

<u>1-Methyl-1*H*-benzo[*d*]imidazol-5-amine (**1**):</u>

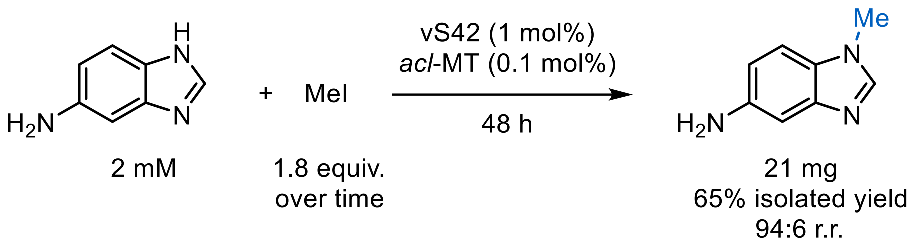


(**1**) was prepared following the general procedure (**VI**), but the addition of MeI (1.8 equiv. instead of 3 equiv.) was made over time (0.15 equiv. MeI mixed with 1.6 µL *i*-PrOH every hour). To a 250 mL Schott flask (final reaction volume: 112 mL) were added substrate (1*H*-benzo[*d*]imidazol-5-amine, 28.9 mg, 0.22 mmol, 2 mM), SAH (0.9 mg, 2.34 µmol, 1 mol%), *acl*-MT (0.22 µmol, 0.1 mol%), vS42 (2.24 µmol, 1 mol%), KPi buffer (50 mM, pH 7.0), *i*-PrOH (2.5 mL), and MeI (25 µL in total, 0.40 mmol, 1.8 equiv.). The reaction was shaken for 48 h. After purification using DCM / MeOH (10:1 v/v) as the mobile phase, the product (**1**, 20.7 mg, 65% yield) was obtained as a pale orange solid. TLC (DCM / MeOH, 10:1 v/v): $R_f$ = 0.55; $^1$H NMR (600 MHz, $CD_3OD$): δ 7.91 (s, 1H; H-2), 7.27 (d, $^3J_{H,H}$ = 8.6 Hz, 1H; H-7), 7.02 (d, $^4J_{H,H}$ = 2.0 Hz, 1H; H-4), 6.84 (dd, $^3J_{H,H}$ = 8.6 Hz, $^4J_{H,H}$ = 2.0 Hz, 1H; H-6), 3.79 (s, 3H; -NC<u>H</u>$_3$); $^{13}$C NMR (151 MHz, $CD_3OD$): δ 144.9 (C-3a), 144.6 (C-2), 144.3 (C-5), 129.8 (C-7a), 115.2 (C-6), 111.2 (C-7), 105.3 (C-4), 31.3 (-N<u>C</u>H$_3$). Analytical data is consistent with that reported in the literature.[35]

1-Methyl-1*H*-benzo[*d*]imidazol-5-ol (**2**):

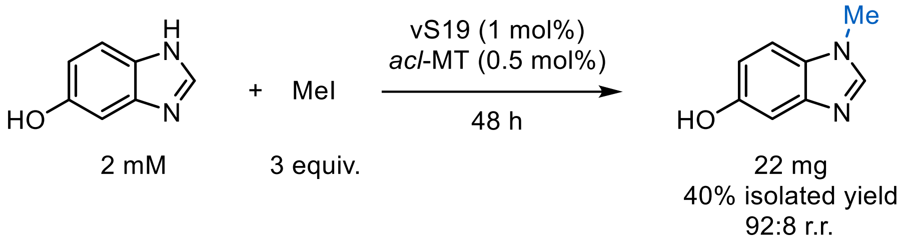


(**2**) was prepared according to the general procedure (**VI**). To a 250 mL Schott flask (final reaction volume: 185 mL) were added substrate (1*H*-benzo[*d*]imidazol-5-ol, 49.7 mg, 0.37 mmol, 2 mM), SAH (0.9 mg, 2.34 µmol, 0.6 mol%), *acl*-MT (1.81 µmol, 0.5 mol%), vS19 (3.70 µmol, 1 mol%), KPi buffer (50 mM, pH 7.0), *i*-PrOH (3.7 mL), and MeI (69.6 µL, 1.12 mmol, 3 equiv.). The reaction was shaken for 48 h. After purification using DCM / MeOH (20:1 v/v) as the mobile phase, the product (**2**, 21.8 mg, 40% yield) were obtained as a pastel orange solid. TLC (DCM / MeOH, 20:1 v/v): $R_f$ = 0.27; $^1$H NMR (600 MHz, DMSO-d$_6$): δ 9.04 (s, 1H; -O<u>H</u>), 8.00 (s, 1H; H-2), 7.32 (d, $^3J_{H,H}$ = 8.6 Hz 1H; H-7), 6.95 (d, $^4J_{H,H}$ = 2.2 Hz, 1H; H-4), 6.76 (dd, $^3J_{H,H}$ = 8.6 Hz, $^4J_{H,H}$ = 2.2 Hz, 1H; H-6), 3.75 (s, 3H; -NC<u>H</u>$_3$); $^{13}$C NMR (151 MHz, DMSO-d$_6$): δ 152.9 (C-5), 144.4 (C-2), 144.3 (C-3a), 128.5 (C-7a), 112.0 (C-6), 110.1 (C-7), 103.8 (C-4), 30.6 (-N<u>C</u>H$_3$). $^1$H NMR data is consistent with that reported in the literature.[36]

1-Methyl-5-nitro-1*H*-benzo[*d*]imidazole (**3**):

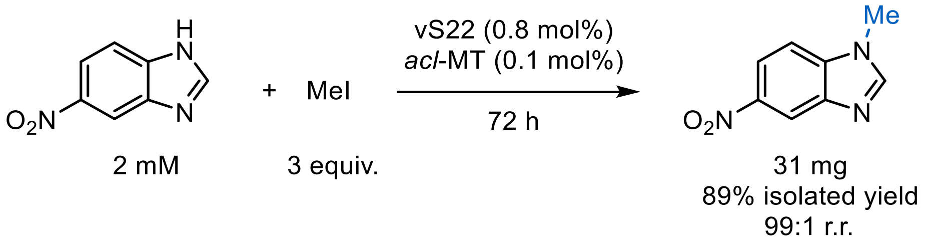


(**3**) was prepared according to the general procedure (**VI**). To a 100 mL Schott flask (final reaction volume: 98 mL) were added substrate (5-nitro-1*H*-benzo[*d*]imidazole, 32.4 mg, 0.20 mmol, 2 mM), SAH (0.6 mg, 1.56 µmol, 0.8 mol%), *acl*-MT (0.20 µmol, 0.1 mol%), vS22 (1.61 µmol, 0.8 mol%), KPi buffer (50 mM, pH 7.0), *i*-PrOH (2.0 mL), and MeI (36.6 µL, 0.59 mmol, 3 equiv.). The reaction was shaken for 72 h. After purification, the product (**3**, 31.4 mg, 89% yield) was obtained as a white solid. TLC (DCM / MeOH, 40:1 v/v): $R_f$ = 0.52; $^1$H NMR (500 MHz, $CD_3OD$ / $CDCl_3$): δ 8.56 (d, $^4J_{H,H}$ = 2.1, 1H; H-4), 8.16 (dd, $^3J_{H,H}$ = 8.9 Hz, $^4J_{H,H}$ = 2.1 Hz, 1H; H-6), 8.04 (s, 1H; H-2), 7.43 (d, $^3J_{H,H}$ = 8.9 Hz, 1H; H-7), 3.87 (s, 3H; -NC<u>H</u>$_3$); $^{13}$C NMR (126 MHz, $CD_3OD$ / $CDCl_3$): δ 147.1 (C-2), 143.8 (C-5), 142.4 (C-3a), 138.4 (C-7a), 118.9 (C-6), 116.5 (C-4), 109.8 (C-7), 31.5 (-N<u>C</u>H$_3$). HRMS (ESI) (m/z) calculated for $[M+H]^+$ $C_8H_7N_3O_2H^+$: 178.0611, found: 178.0622. Analytical data is consistent with that reported in the literature.[37]

1-Methyl-1*H*-benzo[*d*]imidazole-5-carbonitrile (**4**):

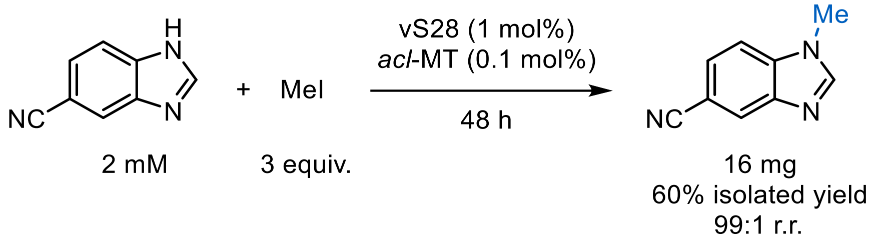


(**4**) was prepared according to the general procedure (**VI**). To a 250 mL Schott flask (final reaction volume: 87 mL) were added substrate (1*H*-benzo[*d*]imidazole-5-carbonitrile, 24.9 mg, 0.17 mmol, 2 mM), SAH (0.5 mg, 1.30 µmol, 0.8 mol%), *acl*-MT (0.17 µmol, 0.1 mol%), vS28 (1.73 µmol, 1 mol%), KPi buffer (50 mM, pH 7.0), *i*-PrOH (1.7 mL), and MeI (32.6 µL, 0.52 mmol, 3 equiv.). The reaction was shaken for 48 h. After purification, the product (**4**, 16.3 mg, 60% yield) was obtained as a white solid. TLC (DCM / MeOH, 40:1 v/v): $R_f$ = 0.26; $^{1}$H NMR (600 MHz, $CDCl_3$): δ 8.11 (dd~d, $^{4}J_{H,H}$ = 1.4 Hz, $^{5}J_{H,H}$ = 0.7 Hz, 1H; H-4), 7.99 (s, 1H; H-2), 7.55 (dd, $^{3}J_{H,H}$ = 8.4 Hz, $^{4}J_{H,H}$ = 1.4 Hz, 1H; H-6), 7.46 (dd, $^{3}J_{H,H}$ = 8.4 Hz, $^{5}J_{H,H}$ = 0.7 Hz, 1H; H-7), 3.89 (s, 3H; -NC$\underline{H}_3$); $^{13}$C NMR (151 MHz, $CDCl_3$): δ 146.0 (C-2), 143.4 (C-3a), 137.3 (C-7a), 126.4 (C-6), 125.6 (C-4), 119.9 (-$\underline{C}$N), 110.7 (C-7), 105.6 (C-5), 31.4 (-N$\underline{C}H_3$). Analytical data is consistent with that reported in the literature.[38]

1-Methyl-1*H*-benzo[*d*]imidazole-5-carbaldehyde (**5**):

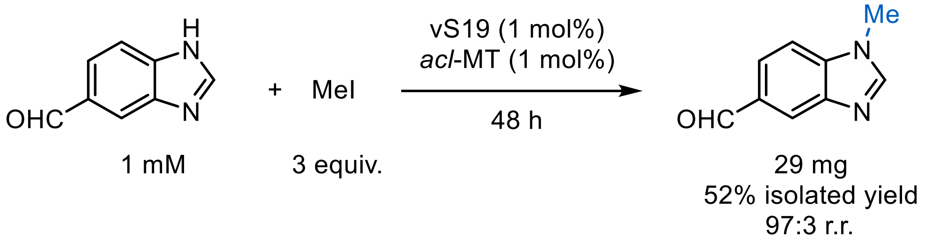


(**5**) was prepared according to the general procedure (**VI**). To a 500 mL Schott flask (final reaction volume: 342 mL) were added substrate (1*H*-benzo[*d*]imidazole-5-carbaldehyde, 50.4 mg, 0.34 mmol, 1 mM), SAH (1.0 mg, 2.60 µmol, 0.8 mol%), *acl*-MT (3.42 µmol, 1 mol%), vS19 (3.44 µmol, 1 mol%), KPi buffer (50 mM, pH 7.0), *i*-PrOH (6.8 mL), and MeI (63.8 µL, 1.02 mmol, 3 equiv.). The reaction was shaken for 48 h. After purification using DCM / MeOH (20:1 v/v) as the mobile phase, the product (**5**, 28.8 mg, 52% yield) was obtained as a white solid. TLC (DCM / MeOH, 20:1 v/v): $R_f$ = 0.38; $^{1}$H NMR (600 MHz, $CDCl_3$): δ 10.05 (s, 1H; -C$\underline{H}$O), 8.26 (d, $^{4}J_{H,H}$ = 1.3 Hz, 1H; H-4), 7.97 (s, 1H; H-2), 7.87 (dd, $^{3}J_{H,H}$ = 8.4 Hz, $^{4}J_{H,H}$ = 1.3 Hz, 1H; H-6), 7.46 (d, $^{3}J_{H,H}$ = 8.4 Hz, 1H; H-7), 3.87 (s, 3H; -NC$\underline{H}_3$); $^{13}$C NMR (151 MHz, $CDCl_3$): δ 192.2 (-$\underline{C}$HO), 145.8 (C-2), 143.6 (C-3a), 138.9 (C-7a), 131.8 (C-5), 124.6 (C-4), 123.5 (C-6), 110.2 (C-7), 31.4 (-N$\underline{C}H_3$). Analytical data is consistent with that reported in the literature.[39]

## 5-Methoxy-1-methyl-1*H*-benzo[*d*]imidazole (**6**):

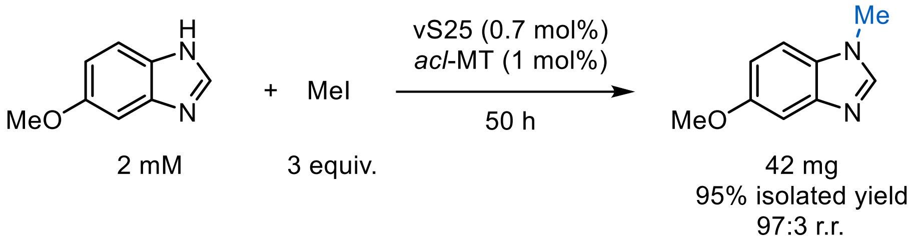


(**6**) was prepared according to the general procedure (**VI**). To a 250 mL Schott flask (final reaction volume: 134 mL) were added substrate (5-methoxy-1*H*-benzo[*d*]imidazole, 39.8 mg, 0.27 mmol, 2 mM), SAH (1.3 mg, 3.38 µmol, 1.2 mol%), *acl*-MT (2.68 µmol, 1 mol%), vS25 (1.98 µmol, 0.7 mol%), KPi buffer (50 mM, pH 7.0), *i*-PrOH (2.7 mL), and MeI (50.4 µL, 0.81 mmol, 3 equiv.). The reaction was shaken for 50 h. After purification, the product (**6**, 41.6 mg, 95% yield) was obtained as a cream colored solid. TLC (DCM / MeOH, 20:1 v/v): $R_f$ = 0.34; $^1$H NMR (600 MHz, $CDCl_3$): δ 7.75 (s, 1H; H-2), 7.25 (d, $^4J_{H,H}$ = 2.3 Hz, 1H; H-4), 7.21 (d, $^3J_{H,H}$ = 8.8 Hz, 1H; H-7), 6.93 (dd, $^3J_{H,H}$ = 8.8 Hz, $^4J_{H,H}$ = 2.3 Hz, 1H; H-6), 3.83 (s, 3H; -OC$\underline{H}_3$), 3.75 (s, 3H; -NC$\underline{H}_3$); $^{13}$C NMR (151 MHz, $CDCl_3$): δ 156.2 (C-5), 144.6 (C-3a), 143.7 (C-2), 129.3 (C-7a), 113.2 (C-6), 109.8 (C-7), 102.3 (C-4), 55.8 (-O$\underline{C}H_3$), 31.1 (-N$\underline{C}H_3$). Analytical data is consistent with that reported in the literature.[40]

## 5-Fluoro-1-methyl-1*H*-benzo[*d*]imidazole (**7**):

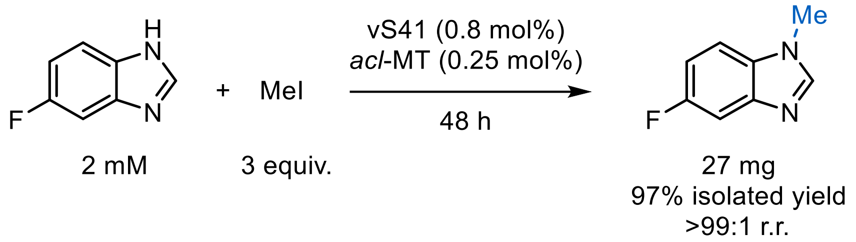


(**7**) was prepared according to the general procedure (**VI**). To a 250 mL Schott flask (final reaction volume: 92 mL) were added substrate (5-fluoro-1*H*-benzo[*d*]imidazole, 24.8 mg, 0.18 mmol, 2 mM), SAH (0.5 mg, 1.30 µmol), *acl*-MT (0.46 µmol, 0.25 mol%), vS41 (1.42 µmol, 0.8 mol%), KPi buffer (50 mM, pH 7.0), *i*-PrOH (1.8 mL), and MeI (34.3 µL, 0.55 mmol, 3 equiv.). The reaction was shaken for 48 h. The product (**7**, 26.8 mg, 97% yield) was obtained as a yellow oil directly after the work-up without purification. TLC (DCM / MeOH, 20:1 v/v): $R_f$ = 0.37; $^1$H NMR (500 MHz, $CDCl_3$): δ 7.85 (s, 1H; H-2), 7.45 (dd, $^3J_{H,F}$ = 9.4 Hz, $^4J_{H,H}$ = 2.4 Hz, 1H; H-4), 7.27 (dd, $^3J_{H,H}$ = 8.9 Hz, $^4J_{H,F}$ = 4.5 Hz, 1H; H-7), 7.05 (ddd~td, $^3J_{H,F}$ = 9.1 Hz, $^3J_{H,H}$ = 8.9 Hz, $^4J_{H,H}$ = 2.4 Hz, 1H; H-6), 3.81 (s, 3H; -NC$\underline{H}_3$); $^{13}$C NMR (126 MHz, $CDCl_3$): δ 159.5 (d, $^1J_{C,F}$ = 237.4 Hz; C-5), 144.9 (C-2), 144.2 (d, $^3J_{C,F}$ = 12.7 Hz; C-3a), 131.2 (C-7a), 111.5 (d, $^2J_{C,F}$ = 26.4 Hz; C-6), 109.8 (d, $^3J_{C,F}$ = 10.3 Hz; C-7), 106.0 (d, $^2J_{C,F}$ = 24.1 Hz; C-4), 31.3 (-N$\underline{C}H_3$). Analytical data is consistent with that reported in the literature.[26]

5-Chloro-1-methyl-1*H*-benzo[*d*]imidazole (**8**):

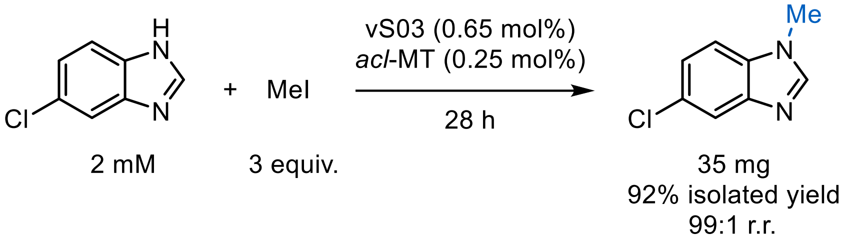


(**8**) was prepared according to the general procedure (**VI**). To a 250 mL Schott flask (final reaction volume: 100 mL) were added substrate (5-chloro-1*H*-benzo[*d*]imidazole, 35.3 mg, 0.23 mmol, 2.3 mM), SAH (0.7 mg, 1.82 μmol), *acl*-MT (0.57 μmol, 0.25 mol%), vS03 (1.51 μmol, 0.65 mol%), KPi buffer (50 mM, pH 7.0), *i*-PrOH (2.0 mL), and MeI (36.7 μL, 0.59 mmol, 2.6 equiv.). The reaction was shaken for 28 h. The product (**8**, 35.3 mg, 92% yield) was obtained as a beige oil directly after the work-up without purification. TLC (DCM / MeOH, 20:1 v/v): $R_f$ = 0.43; $^1$H NMR (600 MHz, $CD_3OD$ / $CDCl_3$): δ 7.81 (s, 1H; H-2), 7.67 (d, $^4J_{H,H}$ = 1.8 Hz, 1H; H-4), 7.24 (d, $^3J_{H,H}$ = 8.6 Hz, 1H; H-7), 7.21 (dd, $^3J_{H,H}$ = 8.6 Hz, $^4J_{H,H}$ = 1.8 Hz, 1H; H-6), 3.77 (s, 3H; -$NC\underline{H}_3$); $^{13}$C NMR (151 MHz, $CD_3OD$ / $CDCl_3$): δ 143.6 (C-2), 142.8 (C-3a), 132.0 (C-7a), 127.1 (C-5), 122.6 (C-6), 118.5 (C-4), 109.4 (C-7), 30.2 ($N\underline{C}H_3$). Analytical data is consistent with that reported in the literature.[26]

5-Iodo-1-methyl-1*H*-benzo[*d*]imidazole (**9**):

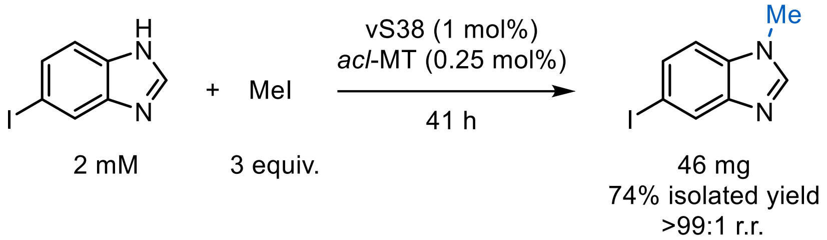


(**9**) was prepared according to the general procedure (**VI**). To a 250 mL Schott flask (final reaction volume: 106 mL) were added substrate (5-iodo-1*H*-benzo[*d*]imidazole, 59.9 mg, 0.24 mmol, 2.3 mM), SAH (0.9 mg, 2.34 μmol, 1 mol%), *acl*-MT (0.52 μmol, 0.22 mol%), vS38 (2.55 μmol, 1 mol%), KPi buffer (50 mM, pH 7.0), *i*-PrOH (1.4 mL), and MeI (45.9 μL, 0.74 mmol, 3 equiv.). The reaction was shaken for 41 h. After purification using DCM / MeOH (20:1 v/v) as the mobile phase, the product (**9**, 46 mg, 74% yield) was obtained as a cream-colored solid. TLC (DCM / MeOH, 20:1 v/v): $R_f$ = 0.41. $^1$H NMR (600 MHz, $CDCl_3$): δ 8.14 (d, $^4J_{H,H}$ = 1.4 Hz, 1H; H-4), 7.79 (s, 1H; H-2), 7.57 (dd, $^3J_{H,H}$ = 8.4 Hz, $^4J_{H,H}$ = 1.4 Hz, 1H; H-6), 7.15 (d, $^3J_{H,H}$ = 8.4 Hz, 1H; H-7), 3.81 (s, 3H; -$NC\underline{H}_3$); $^{13}$C NMR (151 MHz, $CDCl_3$): δ 145.7 (C-3a), 144.2 (C-2), 134.2 (C-7a), 131.6 (C-6), 129.4 (C-4), 111.3 (C-7), 85.5 (C-5), 31.3 ($N\underline{C}H_3$). HRMS (ESI) (m/z) calculated for $[M+H]^+$ $C_8H_7N_2H^+$: 258.9732, found: 258.9736.

<u>5-Bromo-1,2-dimethyl-1*H*-benzo[*d*]imidazole (**10**):</u>

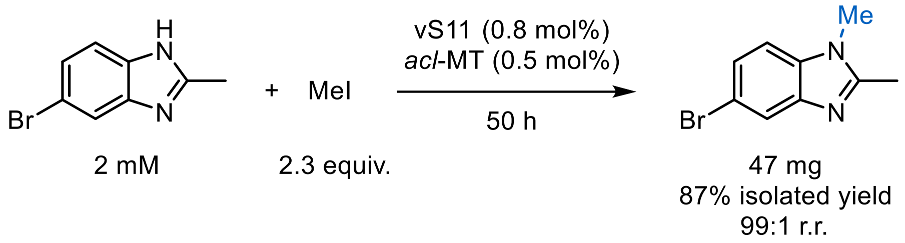

(**10**) was prepared according to the general procedure (**VI**). To a 250 mL Schott flask (final reaction volume: 120 mL) were added substrate (5-bromo-2-methyl-1*H*-benzo[*d*]imidazole, 50.5 mg, 0.24 mmol, 2 mM), SAH (0.8 mg, 2.08 μmol, 0.9 mol%), *acl*-MT (1.18 μmol, 0.5 mol%), vS11 (1.87 μmol, 0.8 mol%), KPi buffer (50 mM, pH 7.0), *i*-PrOH (2.4 mL), and MeI (34.3 μL, 0.55 mmol, 2.3 equiv.). The reaction was shaken for 50 h. After purification, the product (**10,** 47.1 mg, 87% yield) was obtained as a cream-colored solid. TLC (DCM / MeOH, 40:1 v/v): $R_f$ = 0.25; $^1$H NMR (600 MHz, $CDCl_3$): δ 7.70 (d, $^4J_{H,H}$ = 1.8 Hz, 1H; H-4), 7.21 (dd, $^3J_{H,H}$ = 8.5 Hz, $^4J_{H,H}$ = 1.8 Hz, 1H; H-6), 7.00 (d, $^3J_{H,H}$ = 8.5 Hz, 1H; H-7), 3.57 (s, 3H; -NC<u>H</u>$_3$), 2.48 (s, 3H; -C<u>H</u>$_3$); $^{13}$C NMR (151 MHz, $CDCl_3$): δ 153.1 (C-2), 143.9 (C-3a), 134.8 (C-7a), 124.9 (C-6), 121.8 (C-4), 114.7 (C-5), 110.1 (C-7), 30.0 (-N<u>C</u>H$_3$), 13.9 (-<u>C</u>H$_3$). Analytical data is consistent with that reported in the literature.[41]

Gram-scale enzymatic synthesis of 6-bromo-1-methyl-1*H*-benzo[*d*]imidazole (**11**):

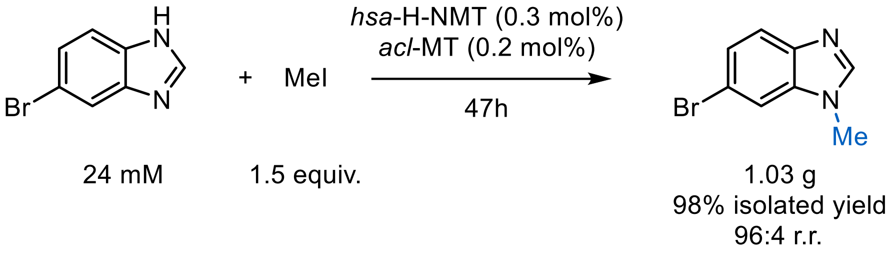


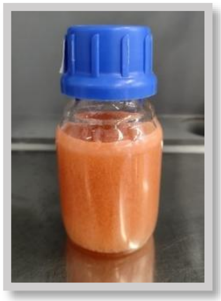

Prepared in one batch

The purified enzymes (*hsa*-H-NMT and *acl*-MT) were thawed and kept on ice before starting the enzymatic reaction (204 mL final volume). Stock solutions of the substrate (5-bromo-1*H*-benzo[*d*]imidazole, 980.8 mg dissolved in 5075 μL DMSO resulting in a reddish solution, 4.98 mmol, 24 mM final concentration) and MeI (5000 μL of a 1.5 M solution in DMSO, 7.50 mmol, 1.5 equiv.) were prepared and added over time. Additionally, an SAH stock solution (37.2 mg dissolved in 3000 μL DMSO, 0.09 mmol, 1.9 mol%) was made and incorporated into the reaction in two portions. In total, 20 mL of DMSO as cosolvent were used (10% (v/v) final concentration). The reaction started by mixing in a Schott flask (250 mL) purified *hsa*-H-NMT (15.22 μmol, 0.30 mol%), purified *acl*-MT (10.15 μmol, 0.20 mol%), KPi buffer (100 mM, pH 7.0), SAH (1500 μL of the stock solution, 0.04 mmol), the substrate (5-bromo-1*H*-benzo[*d*]imidazole, 1000 μL of the stock solution), DMSO (7.0 mL) and MeI (600 μL of the 1.5 M stock solution). The reaction flask was closed, covered with aluminum foil, and shaken (in vertical position) using a Fisherbrand plate-shaker at 150 rpm and room temperature for 47 h. Additions of the substrate (500 μL/h of the stock solution until all the 5075 μL were added) and MeI (300 μL of a freshly prepared 1.5 M solution in DMSO every 30 minutes until a total of 5000 μL were added) were made over time. The remaining SAH (1500 μL of the stock solution, 0.04 mmol) was incorporated with the last addition of substrate. Upon completion (47 h), the reaction was transferred to a 1 L round-bottom flask and mixed with 250 mL MeCN for one hour. The precipitated proteins were removed by centrifugation (4347 rcf, 4°C for 30 mins). The supernatant was collected and the MeCN was removed under reduced pressure using a rotary evaporator. To remove the DMSO from the resulting aqueous solution, a series of multiple extractions with $Et_2O$ (2 x 250 mL and 6 x 100 mL, 1100 mL in total), and back-washes (8 x 250 mL, 2000 mL in total) were made. The organic layers were collected and concentrated using a rotary evaporator. After purification of the reaction product by flash chromatography using DCM / MeOH (20:1 v/v) as the mobile phase, 1025.4 mg (98% yield) were obtained as a beige solid. TLC (DCM / MeOH, 20:1 v/v): $R_f$ = 0.28; $^{1}$H NMR (600 MHz, $CD_3OD$): δ 7.77 (s, 1H; H-2), 7.61 (d, $^{3}J_{H,H}$ = 8.6 Hz, 1H; H-4), 7.47 (d, $^{4}J_{H,H}$ = 1.8 Hz, 1H; H-7), 7.33 (dd, $^{3}J_{H,H}$ = 8.6 Hz, $^{4}J_{H,H}$ = 1.8 Hz, 1H; H-5), 3.73 (s, 3H; -NC<u>H</u>$_3$); $^{13}$C NMR (151 MHz, $CD_3OD$): δ 144.2 (C-2), 142.6 (C-3a), 135.6 (C-7a), 125.4 (C-5), 121.5 (C-4), 116.2 (C-6), 112.6 (C-7), 31.1 (-N<u>C</u>H$_3$). Analytical data is consistent with that reported in the literature.[32]

5-(1-methyl-1*H*-pyrrolo[2,3-*d*]pyrimidin-4-yl)-4,5,6,7-tetrahydro-1*H*-imidazo[4,5-*c*]pyridine (**13**):

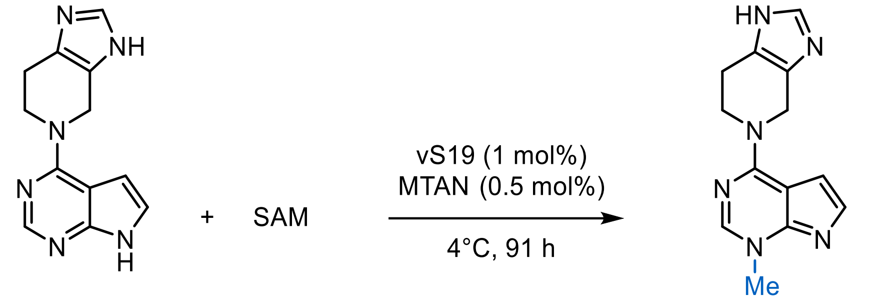


2 mM | 1.5 equiv. | 43 mg
34% isolated yield
>99% selectivity

A 250 mL reaction was made by mixing the substrate (**12**, 119.5 mg, 0.49 mmol, 2 mM) with SAM (578.8 mg, 0.75 mmol, 1.5 equiv.) and KPi buffer (50 mM, pH 6.0) in a 250 mL Schott flask. The reaction mixture was cooled on ice before adding the previously thawed purified enzymes: MTAN (2.48 μmol, 0.5 mol%) and vS19 (5.06 μmol, 1 mol%). The resulting cloudy suspension was covered with aluminum foil and shaken on a Fisherbrand plate-shaker at 150 rpm and 4°C for 91 h. The reaction mixture was transferred to a 1 L round-bottom flask and quenched by stirring with MeCN (200 mL) for one hour. Precipitated enzymes were removed by centrifugation (4347 rcf, 50 min, 4°C), the supernatant was collected in a round-bottom flask and concentrated under reduced pressure using a rotary evaporator. The resulting crude paste was washed with MeOH (100 mL used in total) and the combined washes were concentrated, affording a yellowish oil that was purified by column chromatography using a DCM / MeOH (10:1 v/v) mixture to remove most of the glycerol and the unreacted substrate, followed by MeOH to elute the product. The methylated product was obtained as a dark yellow solid (**13**, 48 mg, as a product / glycerol mixture with a mole ratio of 3:1, 34% yield). TLC (DCM / MeOH, 2:1 v/v): $R_f$ = 0.21; $^1$H NMR (500 MHz, $D_2O$ / $CD_3OD$): δ = 7.88 (s, 1H; H-2´), 7.60 (s, 1H; H-2), 7.23 (d, $^3J_{H,H}$ = 2.8 Hz, 1H; H-6´), 6.58 (d, $^3J_{H,H}$ = 2.8 Hz, 1H; H-5´), 4.75 (s, 2H; H-5), 4.05 (t, 2H, $^3J_{H,H}$ = 5.5 Hz, H-6), 3.81 (s, 3H; N$\underline{C}$$\underline{H}_3$), 2.73 (t, 2H, $^3J_{H,H}$ = 5.5 Hz, H-7); $^{13}$C NMR (151 MHz, $D_2O$ / $CD_3OD$): δ 156.6 (C-4´), 147.8 (C-7a´), 143.2 (C-2´), 136.4 (C-6´), 135.8 (C-2), 129.8 (C-3a), 126.9 (C-7a), 105.2 (C-4a´), 103.1 (C-5´), 45.8 (C-4), 45.2 (C-6), 36.9 (-N$\underline{C}H_3$), 23.0 (C-7). HRMS (ESI) (m/z) calculated for $[M+H]^+$ $C_{13}H_{14}N_6H^+$: 255.13527, found: 255.1362.

3-Methyl-5-(7*H*-pyrrolo[2,3-*d*]pyrimidin-4-yl)-4,5,6,7-tetrahydro-3*H*-imidazo[4,5-*c*]pyridine (**14**):

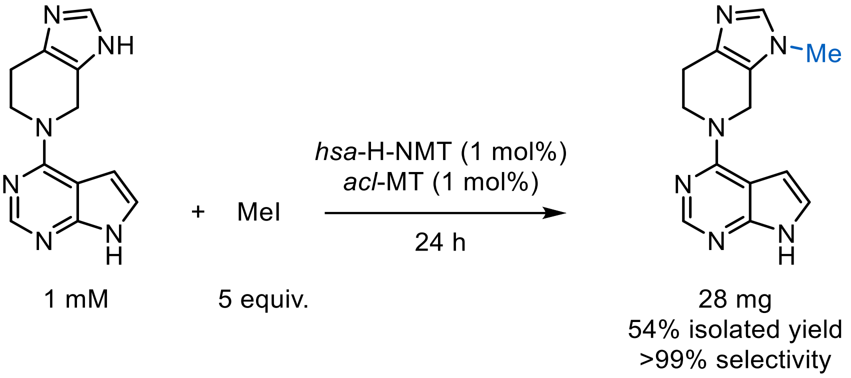


A 206 mL reaction was made by mixing the substrate (**12**, 49.6 mg, 0.21 mmol, 1 mM) with SAH (1.1 mg, 2.86 μmol, 1.3 mol%) in KPi buffer (50 mM, pH 7.0) containing *i*-PrOH (3.5 mL) as cosolvent in a 250 mL Schott flask. To the resulting suspension (the substrate was not completely solubilized), the purified *acl*-MT wild-type (2.07 μmol, 1 mol%) and purified *hsa*-H-NMT (2.08 μmol, 1 mol%) were incorporated after thawing, followed by the addition of a MeI solution (65.5 μL, 1.05 mmol, 5 equiv. mixed with 500 μL *i*-PrOH, 2% (v/v) final cosolvent concentration). The reaction flask was covered with aluminum foil and shaken on a Fisherbrand plate-shaker at 150 rpm and room temperature for 24 h. After completion, the reaction was saturated with NaCl and extracted 6 times with EtOAc (1:1 v/v). The organic layers were combined and concentrated using a rotary evaporator. The resulting product was washed with $H_2O$ and further dried to afford the methylated product (**14**, 28.2 mg, 54% yield) as a light cream-colored solid. TLC (DCM / MeOH, 5:1 v/v): $R_f$ = 0.56; $^1$H NMR (600 MHz, $CD_3OD$): δ 8.17 (s, 1H; H-2´), 7.50 (s, 1H; H-2), 7.17 (d, $^3J_{H,H}$ = 3.6 Hz, 1H; H-6´), 6.70 (d, $^3J_{H,H}$ = 3.6 Hz, 1H; H-5´), 4.91 (t, $^5J_{H,H}$ = 1.5 Hz, 2H; H-4), 4.18 (t, $^3J_{H,H}$ = 5.6 Hz, 2H; H-6), 3.66 (s, 3H; NC$\underline{H}_3$), 2.79 (tt, $^3J_{H,H}$ = 5.6 Hz, $^5J_{H,H}$ = 1.5 Hz, 2H; H-7); $^{13}$C NMR (151 MHz, $CD_3OD$): δ 158.6 (C-4´), 152.4 (C-7a´), 151.5 (C-2´), 138.0 (C-2), 135.5 (C-7a), 125.2 (C-3a), 122.8 (C-6´), 104.3 (C-4a´), 102.2 (C-5´), 45.9 (C-6), 43.0 (C-4), 31.5 (-N$\underline{C}H_3$), 25.5 (C-7). HRMS (ESI) (m/z) calculated for $[M+H]^+$ $C_{13}H_{14}N_6H^+$: 255.13527, found: 255.1357.

## 1-Methyl-5-(7*H*-pyrrolo[2,3-*d*]pyrimidin-4-yl)-4,5,6,7-tetrahydro-1*H*-imidazo[4,5-*c*]pyridine (**15**):

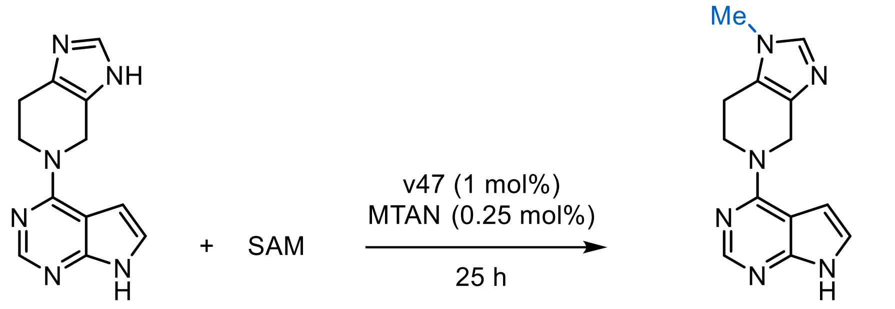


2 mM 3 equiv. 25 mg 46% isolated yield >98% selectivity

In a 250 mL Schott flask (final reaction volume: 118 mL), the substrate (**12**, 52.6 mg, 0.22 mmol, 2 mM) was mixed with SAM (482.0 mg, 0.63 mmol, 3 equiv.) and KPi buffer (50 mM, pH 7.0) containing *i*-PrOH (2.0 mL, 1.7% v/v final concentration) as cosolvent. To the resulting suspension (the substrate was not completely solubilized), MTAN (0.52 μmol, 0.24 mol%) and v47 (2.08 μmol, 0.9 mol%) were incorporated after thawing. The reaction flask was covered with aluminum foil and shaken in an Innova 42 incubator shaker at 70 rpm, 37°C for 25 h. After completion, the reaction was saturated with NaCl and extracted 6 times with EtOAc (1:1 v/v). The organic layers were combined and concentrated using a rotary evaporator furnishing an orange oil. The reaction crude was purified by preparative HPLC (see section P iii) affording 28 mg of product (**15**) as a tosylate adduct (in a mole ratio 8:1 for a 46% yield. The tosylate source was the SAM counterion). To remove the tosylate and obtain a clean NMR, the product was dissolved in 20 mL $H_2O$. The aqueous solution was basified using a NaOH solution (1 M) until pH 11 giving a white turbid dispersion that was extracted with DCM (4 x 20 mL). The organic layers were combined and concentrated with a rotary evaporator followed by acidification with HCl (1M, 2 mL) giving 17.1 mg of a pale-cream colored solid as the hydrochloride adduct (27% yield) after full removal of the $H_2O$. TLC (DCM / MeOH, 10:1 v/v): $R_f$ = 0.26; $^1$H NMR (600 MHz, $D_2O$ / $CD_3OD$): δ 8.70 (s, 1H; H-2), 8.43 (s, 1H; H-2´), 7.47 (d, $^3J_{H,H}$ = 3.7 Hz, 1H; H-6´), 7.03 (d, $^3J_{H,H}$ = 3.7 Hz, 1H; H-5´), 5.23 (t, $^5J_{H,H}$ = 1.3 Hz, 2H; H-4), 4.42 (t, $^3J_{H,H}$ = 5.6 Hz, 2H; H-6), 3.86 (s, 3H; $NC\underline{H}_3$), 3.10 (tt, $^3J_{H,H}$ = 5.6 Hz, $^5J_{H,H}$ = 1.3 Hz, 2H; H-7); $^{13}$C NMR (151 MHz, $D_2O$ / $CD_3OD$): δ 155.6 (C-4´), 144.5 (C-7a´), 143.9 (C-2´), 135.6 (C-2), 129.5 (C-7a), 125.6 (C-6´), 125.0 (C-3a), 104.5 (C-5´), 103.8 (C-4a´), 45.6 (C-6), 44.2 (C-4), 33.8 (-$N\underline{C}H_3$), 20.8 (C-7). HRMS (ESI) (m/z) calculated for $[M+H]^+$ $C_{13}H_{14}N_6H^+$: 255.13527, found: 255.1357.

3-Ethyl-5-(7*H*-pyrrolo[2,3-*d*]pyrimidin-4-yl)-4,5,6,7-tetrahydro-3*H*-imidazo[4,5-*c*]pyridine (**24**):

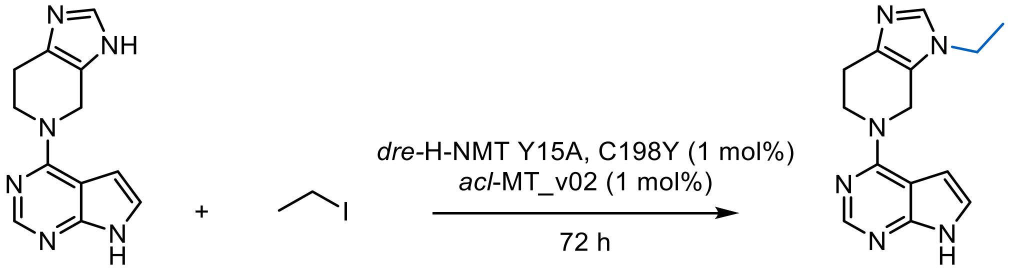


A 43 mL reaction was made by mixing the substrate (**12**, 20.4 mg, 0.08 mmol, 2 mM) with SAH (3.0 mg, 7.80 μmol, 9.8 mol%) in KPi buffer (50 mM, pH 7.0) in a 100 mL Schott flask. To the resulting suspension (the substrate was not completely solubilized), the purified *acl*-MT v02 (0.62 μmol, 0.8 mol%) and purified *dre*-H-NMT Y15A, C198Y (0.81 μmol, 1 mol%) were incorporated after thawing, followed by the addition of an Iodoethane solution (20.0 μL, 0.25 mmol, 3 equiv. mixed with 1000 μL *i*-PrOH). The reaction flask was covered with aluminum foil and shaken on a Fisherbrand plate-shaker at 150 rpm at room temperature. After 48 h, a second addition of Iodoethane in *i*-PrOH (20.0 μL, 0.25 mmol, 3 equiv. mixed with 1000 μL *i*-PrOH for a 4.6% (v/v) final cosolvent concentration) was performed. The reaction was shaken for another 24 h for a total reaction time of 72 h. After completion, the reaction was saturated with NaCl and extracted 10 times with EtOAc (1:1 v/v). The organic layers were combined and concentrated using a rotary evaporator. After purification by flash chromatography using a DCM / MeOH (20:1) as the mobile phase, the product (**24**, 18.4 mg as a product / glycerol mixture with a mole ratio of 1:0.7, 64% yield) was obtained as a white solid. TLC (DCM / MeOH 20:1 v/v): $R_f$ = 0.15; $^1$H NMR (600 MHz, $CDCl_3$ / $CD_3OD$): δ = 8.19 (s, 1H; H-2´), 7.47 (s, 1H, H-2), 7.12 (d, $^3J_{H,H}$ = 3.6 Hz, 1H; H-6´), 6.62 (d, $^3J_{H,H}$ = 3.6 Hz, 1H; H-5´), 4.93 (s, 2H; H-4), 4.20 (t, $^3J_{H,H}$ = 5.6 Hz, 2H; H-6), 3.99 (q, $^3J_{H,H}$ = 7.3 Hz, 2H; H-1´´), 2.86 (t, $^3J_{H,H}$ = 5.6 Hz, 2H; H-7), 1.46 (t, $^3J_{H,H}$ = 7.3 Hz, 3H; H-2´´); $^{13}$C NMR (151 MHz, $CDCl_3$ / $CD_3OD$): δ 158.0 (C-4´), 151.7 (C-7a´), 151.0 (C-2´), 136.0 (C-2), 135.2 (C-7a), 123.7 (C-3a), 122.3 (C-6´), 103.8 (C-4a´), 101.6 (C-5´), 45.7 (C-6), 42.7 (C-4), 40.8 (C-1´´), 25.1 (C-7), 16.4 (C-2´´). HRMS (ESI) (m/z) calculated for $[M+H]^+$ $C_{14}H_{16}N_6H^+$: 269.1509, found: 269.1516.

3-Allyl-5-(7*H*-pyrrolo[2,3-*d*]pyrimidin-4-yl)-4,5,6,7-tetrahydro-3*H*-imidazo[4,5-*c*]pyridine (**25**):

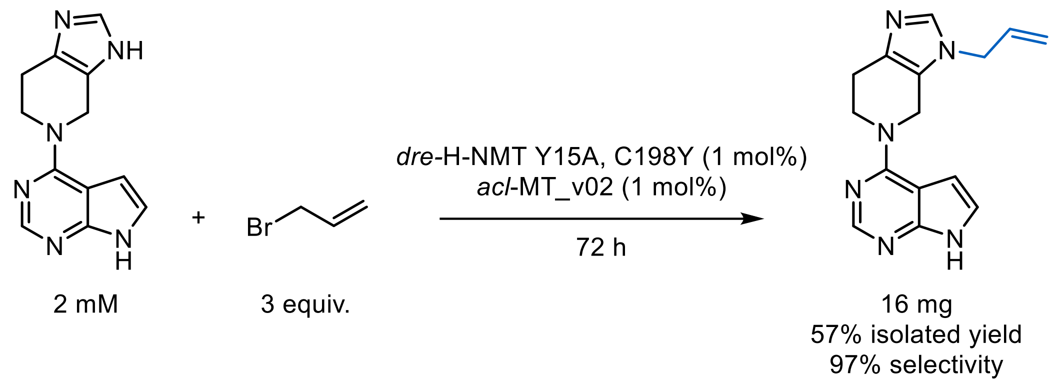


A 42 mL reaction was made by mixing substrate (**12**, 20.0 mg, 0.08 mmol, 2 mM) with SAH (2.8 mg, 7.28 μmol, 9 mol%) in KPi buffer (50 mM, 31 mL, pH 7.0) in a 100 mL Schott flask. To the resulting suspension (the substrate was not completely solubilized), the purified *acl*-MT v02 (0.62 μmol, 0.8 mol%) and purified *dre*-H-NMT Y15A, C198Y (0.84 μmol, 1 mol%) were incorporated after thawing, followed by the addition of an allyl bromide solution (22.0 μL, 0.25 mmol, 3 equiv. mixed with 1000 μL *i*-PrOH, 2.4% (v/v) final cosolvent concentration). The reaction flask was covered with aluminum foil and shaken on a Fisherbrand plate-shaker at 150 rpm and room temperature for 72 h. After completion, the reaction was saturated with NaCl and extracted 6 times with EtOAc (1:1 v/v). The organic layers were combined and concentrated using a rotary evaporator. After purification by flash chromatography using a DCM / MeOH (20:1) as the mobile phase, the product (**25**, 16 mg, 57% yield) was obtained as a white solid. TLC (DCM / MeOH 20:1 v/v): $R_f$ = 0.19; $^1$H NMR (600 MHz, $CDCl_3$ / $CD_3OD$): δ = 8.18 (s, 1H, H-2´), 7.47 (s, 1H, H-2), 7.13 (d, $^3J_{H,H}$ = 3.6 Hz, 1H, H-6´), 6.60 (d, $^3J_{H,H}$ = 3.6 Hz, 1H; H-5´), 5.99 (ddt, $^3J_{H,H}$ = 5.4 Hz, $^3J_{H,H\ cis}$ = 10.3 Hz, $^3J_{H,H\ trans}$ = 17.1 Hz, 1H; H-2´´), 5.30 (dd, $^3J_{H,H\ cis}$ = 10.3 Hz, $^2J_{H,H}$ = 0.9 Hz, 1H; H-3b´´), 5.15 (dd, $^3J_{H,H\ trans}$ 17.1 Hz, $^2J_{H,H}$ = 0.9 Hz, 1H; H-3a´´), 4.89 (s, 2H; H-4), 4.60 (d, $^3J_{H,H}$ = 5.4 Hz, 2H; H-1´´), 4.19 (t, $^3J_{H,H}$ = 5.6 Hz, 2H; H-6), 2.86 (t, $^3J_{H,H}$ = 5.6 Hz, 2H; H-7); $^{13}$C NMR (151 MHz, $CDCl_3$ / $CD_3OD$): δ 158.1 (C-4´), 151.8 (C-7a´), 151.1 (C-2´), 137.0 (C-2), 135.6 (C-7a), 133.4 (C-2´´), 124.3 (C-3a), 122.4 (C-6´), 118.7 (C-3´´), 103.9 (C-4a´), 101.7 (C-5´), 48.3 (C-1´´), 45.5 (C-6), 42.93 (C-4), 25.1 (C-7). HRMS (ESI) (m/z) calculated for $[M+H]^+$ $C_{15}H_{16}N_6H^+$: 281.1509, found: 281.1512.

1-Allyl-6-bromo-1*H*-benzo[*d*]imidazole (**19**):

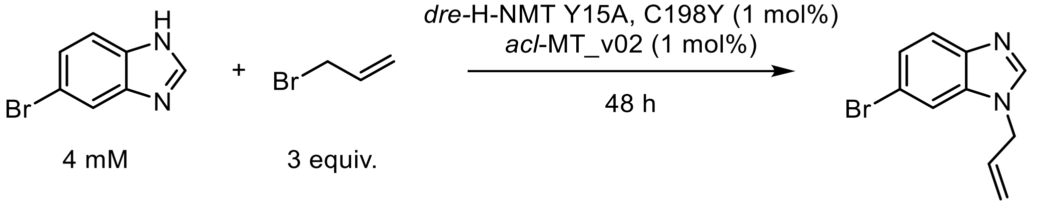


A 25 mL reaction was performed through solving 5-bromo-1*H*-benzo[*d*]imidazole (20.1 mg, 0.1 mmol, 4 mM, solved in 250 μL *i*-PrOH) with SAH (0.19 mg, 0.5 μmol, 0.5 mol%, solved in 250 μL *i*-PrOH) and mixed with KPi buffer (50 mM, 12.25 mL, pH 7.0) in a 25 mL Schott flask. Purified *acl*-MT v02 (1 μmol, 1 mol%) and purified *dre*-H-NMT Y15A, C198Y (1 μmol, 1 mol%) was added to the mixture, followed by the addition of an allyl bromide solution (26.2 μL, 0.3 mmol, 3 equiv. mixed with 250 μL DMSO). The reaction flask was tightly closed with a screw lid, covered with aluminum foil and shaken on a Fisherbrand plate-shaker at 150 rpm and room temperature for 48 h. After completion, the reaction was saturated with NaCl and extracted 3 times with EtOAc (1:1 v/v). The organic layers were combined, dried over $MgSO_4$, and concentrated under reduced pressure. After purification by flash chromatography using a DCM / MeOH (10:1 v/v) as the mobile phase, the product (**19**, 22 mg, 94% yield) was obtained as a yellowish solid with traces of DMSO. TLC (DCM / MeOH 10:1 v/v): $R_f$ = 0.5; $^1$H NMR (600 MHz, $CDCl_3$): δ 7.86 (s, 1H), 7.66 (d, $^3J_{H,H}$ = 8.6 Hz, 1H), 7.52 (d, $^4J_{H,H}$ = 1.8 Hz, 1H), 7.37 (dd, $^3J_{H,H}$ = 8.6, $^4J_{H,H}$ = 1.8 Hz, 1H), 6.10 – 5.88 (m, 1H), 5.36 – 5.06 (m, 1H), 4.71 - 4.74 (m, 2H); $^{13}$C NMR (151 MHz, $CDCl_3$): δ 143.7 (C-2), 143.0 (C-3a), 135.1 (C-7a), 131.5 (C-2'), 125.7 (C-5), 121.8 (C-4), 119.2 (C-3'), 116.4 (C-6), 113.2 (C-7), 47.6 (C-1'). HRMS (ESI) (m/z) calculated for $[M+H]^+$ $C_{10}H_{10}BrN_2H^+$: 237.0027, found: 237.0026.

6-Bromo-1-(2-methylallyl)-1*H*-benzo[*d*]imidazole (**20**):

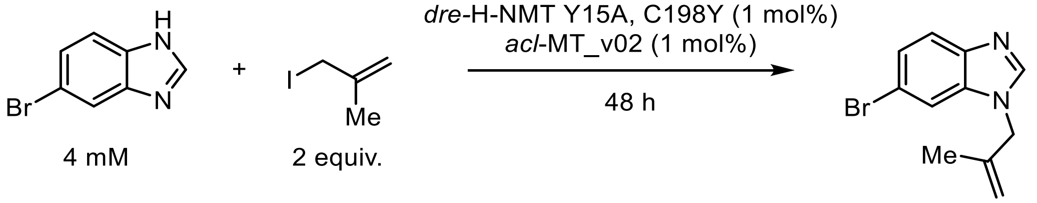


A 25 mL reaction was performed through solving 5-bromo-1*H*-benzo[*d*]imidazole (20.1 mg, 0.1 mmol, 4 mM, solved in 250 μL *i*-PrOH) with SAH (1.92 mg, 5 μmol, 5 mol%, solved in 250 μL *i*-PrOH) and mixed with KPi buffer (50 mM, 12.25 mL, pH 7.0) in a 25 mL Schott flask. Purified *acl*-MT v02 (1 μmol, 1 mol%) and purified *dre*-H-NMT Y15A, C198Y (1 μmol, 1 mol%) was added to the mixture, followed by the addition of a 3-iodo-2-methylprop-1-ene solution (21 μL, 0.2 mmol, 2 equiv. mixed with 250 μL DMSO). The reaction flask was tightly closed with a screw lid, covered with aluminum foil and shaken on a Fisherbrand plate-shaker at 150 rpm and room temperature for 48 h. After completion, the reaction was saturated with NaCl and extracted 3 times with EtOAc (1:1 v/v). The organic layers were combined, dried over $MgSO_4$, and concentrated under reduced pressure. After purification by flash chromatography using a DCM / MeOH (10:1 v/v) as the mobile phase, the product (**20**, 10.1 mg, 40% yield) was obtained as a yellowish solid with traces of DMSO. TLC (DCM /

MeOH 10:1 v/v): $R_f$ = 0.62; $^1$H NMR (600 MHz, $CDCl_3$): δ 7.86 (s, 1H), 7.66 (d, $^3J_{H,H}$ = 8.6 Hz, 1H), 7.51 (d, $^4J_{H,H}$ = 1.8 Hz, 1H), 7.38 (dd, $^3J_{H,H}$ = 8.6, $^4J_{H,H}$ = 1.8 Hz, 1H), 5.01 – 5.03 (m, 1H), 4.81 (s, 1H), 4.65 (s, 2H), 1.71 (s, 3H); $^{13}$C NMR (151 MHz, $CDCl_3$): δ 144.1 (C-2), 143.0 (C-3a), 139.2 (C-2'), 135.2 (C-7a), 125.7 (C-5), 121.8 (C-4), 116.5 (C-6), 114.5 (C-3'), 113.3 (C-7), 51.3 (C-1'), 19.9 (C-1''). HRMS (ESI) (m/z) calculated for $[M+H]^+$ $C_{11}H_{12}BrN_2H^+$: 251.0184, found: 251.0179.

6-Bromo-1-(2-fluoroallyl)-1*H*-benzo[*d*]imidazole (**21**):

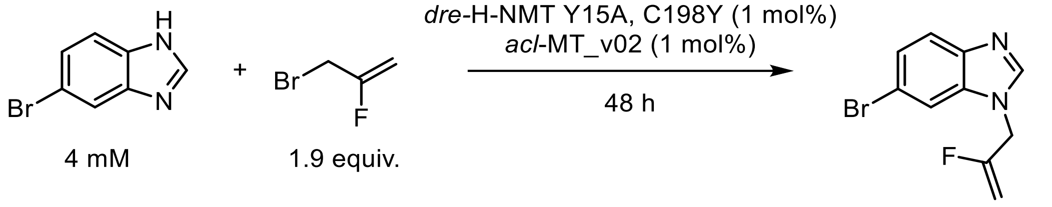


A 25 mL reaction was performed through solving 5-bromo-1*H*-benzo[*d*]imidazole (20.1 mg, 0.1 mmol, 4 mM, solved in 250 μL *i*-PrOH) with SAH (1.92 mg, 5 μmol, 5 mol%, solved in 250 μL *i*-PrOH) and mixed with KPi buffer (50 mM, 12.25 mL, pH 7.0) in a 25 mL Schott flask. Purified *acl*-MT v02 (1 μmol, 1 mol%) and purified *dre*-H-NMT Y15A, C198Y (1 μmol, 1 mol%) was added to the mixture, followed by the addition of a 3-bromo-2-fluoroprop-1-ene solution (17.5 μL, 0.188 mmol, 1.88 equiv. mixed with 250 μL DMSO). The reaction flask was tightly closed with a screw lid, covered with aluminum foil and shaken on a Fisherbrand plate-shaker at 150 rpm and room temperature for 48 h. After completion, the reaction was saturated with NaCl and extracted 3 times with EtOAc (1:1 v/v). The organic layers were combined, dried over $MgSO_4$, and concentrated under reduced pressure. After purification by flash chromatography using a DCM / MeOH (10:1 v/v) as the mobile phase, the product (**21**, 8.5 mg, 33% yield) was obtained as a yellowish solid with traces of DMSO. TLC (DCM / MeOH 10:1 v/v): $R_f$ = 0.57; $^1$H NMR (600 MHz, $CDCl_3$): δ 7.89 (s, 1H), 7.67 (d, $^3J_{H,H}$ = 8.6 Hz, 1H), 7.55 (d, $^4J_{H,H}$ = 1.8 Hz, 1H), 7.40 (dd, $^3J_{H,H}$ = 8.6, $^4J_{H,H}$ = 1.8 Hz, 1H), 4.87 (dd, $^3J_{H,F}$ = 15.9, $^2J_{H,H}$ = 3.8 Hz, 1H), 4.80 (d, $^3J_{H,F}$ = 11.5 Hz, 2H), 4.52 (dd, $^3J_{H,F}$ = 47.1, $^2J_{H,H}$ = 3.8 Hz, 1H); $^{13}$C NMR (151 MHz, $CDCl_3$): δ 159.9 (C-2'), 158.1 (C-2'), 143.8 (C-2), 142.9 (C-3a), 134.8 (C-7a), 126.1 (C-5), 122.0 (C-4), 116.9 (C-6), 112.9 (C-7), 94.4 (d, *J* = 16.8 Hz, C-3'), 45.4 (d, *J* = 34.8 Hz, C-1'). HRMS (ESI) (m/z) calculated for $[M+H]^+$ $C_{10}H_9BrFN_2H^+$: 254.9933, found: 254.9929.

2-(6-Bromo-1*H*-benzo[*d*]imidazol-1-yl)acetonitrile (**22**):

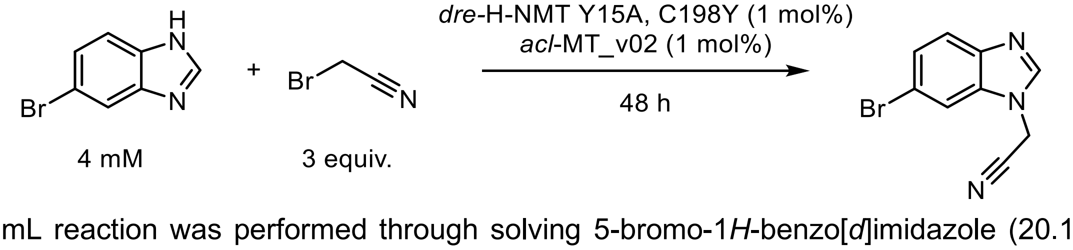


A 25 mL reaction was performed through solving 5-bromo-1*H*-benzo[*d*]imidazole (20.1 mg, 0.1 mmol, 4 mM, solved in 250 μL *i*-PrOH) with SAH (1.92 mg, 5 μmol, 5 mol%, solved in

250 μL *i*-PrOH) and mixed with KPi buffer (50 mM, 12.25 mL, pH 7.0) in a 25 mL Schott flask. Purified *acl*-MT v02 (1 μmol, 1 mol%) and purified *dre*-H-NMT Y15A, C198Y (1 μmol, 1 mol%) was added to the mixture, followed by the addition of a bromoacetonitril solution (21.5 μL, 0.3 mmol, 3 equiv. mixed with 250 μL DMSO). The reaction flask was tightly closed with a screw lid, covered with aluminum foil and shaken on a Fisherbrand plate-shaker at 150 rpm and room temperature for 48 h. After completion, the reaction was saturated with NaCl and extracted 3 times with EtOAc (1:1 v/v). The organic layers were combined, dried over $MgSO_4$, and concentrated under reduced pressure. After purification by flash chromatography using a DCM / MeOH (10:1 v/v) as the mobile phase, the product (**22**, 8.1 mg, 34% yield) was obtained as a yellowish solid with traces of DMSO. TLC (DCM / MeOH 10:1 v/v): $R_f$ = 0.53; $^1$H NMR (600 MHz, $CD_3OD$): δ 8.27 (s, 1H), 7.94 (d, $^4J_{H,H}$ = 1.8 Hz, 1H), 7.66 (d, $^4J_{H,H}$ = 8.6, 1H), 7.51 (dd, $^3J_{H,H}$ = 8.6, $^4J_{H,H}$ = 1.8 Hz, 1H), 5.50 (s, 2H); $^{13}$C NMR (151 MHz, $CD_3OD$): δ 145.5 (C-2), 143.1 (C-3a), 135.4 (C-7a), 127.8 (C-5), 122.1 (C-4), 118.3 (C-6), 115.5 (C-2‘), 114.5 (C-7), 33.5 (C-1‘). HRMS (ESI) (m/z) calculated for $[M+H]^+$ $C_9H_7BrN_3H^+$: 235.9823, found: 235.9822.

6-Bromo-1-(prop-2-yn-1-yl)-1*H*-benzo[*d*]imidazole (**23**):

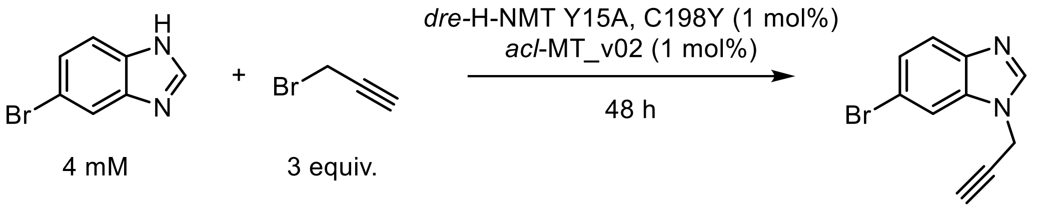


A 25 mL reaction was performed through solving 5-bromo-1*H*-benzo[*d*]imidazole (20.1 mg, 0.1 mmol, 4 mM, solved in 250 μL *i*-PrOH) with SAH (1.92 mg, 5 μmol, 5 mol%, solved in 250 μL *i*-PrOH) and mixed with KPi buffer (50 mM, 12.25 mL, pH 7.0) in a 25 mL Schott flask. Purified *acl*-MT v02 (1 μmol, 1 mol%) and purified *dre*-H-NMT Y15A, C198Y (1 μmol, 1 mol%) was added to the mixture, followed by the addition of a bromopropargyl solution (33.7 μL, 0.3 mmol, 3 equiv. mixed with 250 μL DMSO). The reaction flask was tightly closed with a screw lid, covered with aluminum foil and shaken on a Fisherbrand plate-shaker at 150 rpm and room temperature for 48 h. After completion, the reaction was saturated with NaCl and extracted 3 times with EtOAc (1:1 v/v). The organic layers were combined, dried over $MgSO_4$, and concentrated under reduced pressure. After purification by flash chromatography using a DCM / MeOH (10:1 v/v) as the mobile phase, the product (**23**, 20.3 mg, 86% yield) was obtained as a yellowish solid with traces of DMSO. TLC (DCM / MeOH 10:1 v/v): $R_f$ = 0.62; $^1$H NMR (600 MHz, $CDCl_3$): δ 7.97 (s, 1H), 7.65 (d, $^3J_{H,H}$ = 8.6 Hz, 0H), 7.63 (d, $^4J_{H,H}$ = 1.8 Hz, 1H), 7.39 (dd, $^3J_{H,H}$ = 8.6, $^4J_{H,H}$ = 1.8 Hz, 1H), 4.87 (d, $^4J_{H,H}$ = 2.6 Hz, 2H), 2.53 (t, $^4J_{H,H}$ = 2.6 Hz, 1H); $^{13}$C NMR (151 MHz, $CDCl_3$): δ 143.0 (C-2, C-3a), 134.5 (C-7a), 126.1 (C-5), 121.9 (C-4), 116.7 (C-6), 113.0 (C-7), 75.6 (C-2'), 75.5 (C-3'), 34.8 (C-1'). HRMS (ESI) (m/z) calculated for $[M+H]^+$ $C_{10}H_8BrN_2H^+$: 234.9871, found: 234.9867.

# VII. NMR spectra

NMR spectra from enzymatic synthesis of 1-methyl-1*H*-benzo[*d*]imidazol-5-amine (**1**).

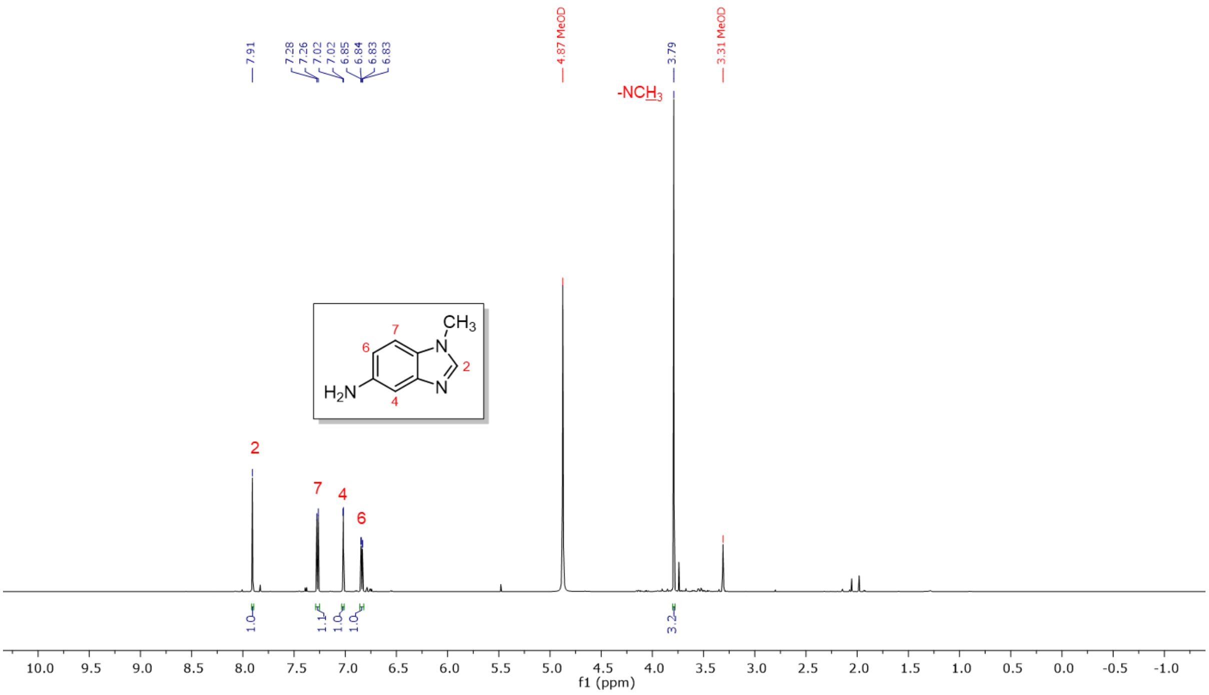


$^{1}$H-NMR (600 MHz, $CD_3OD$) spectrum

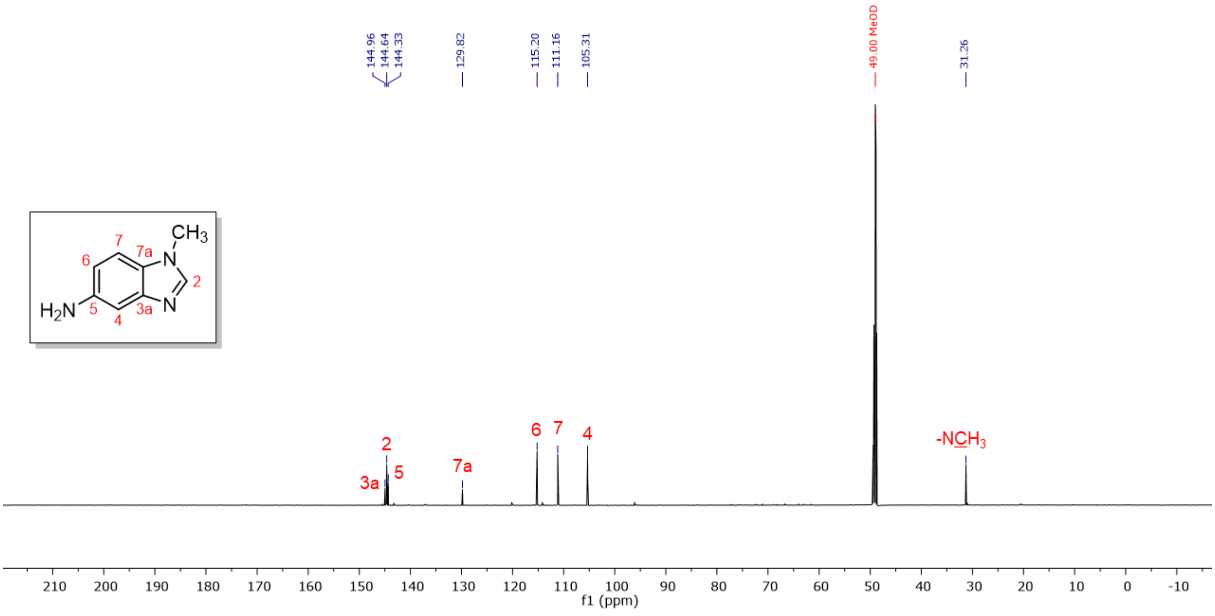


$^{13}$C-NMR (151 MHz, $CD_3OD$) spectrum.

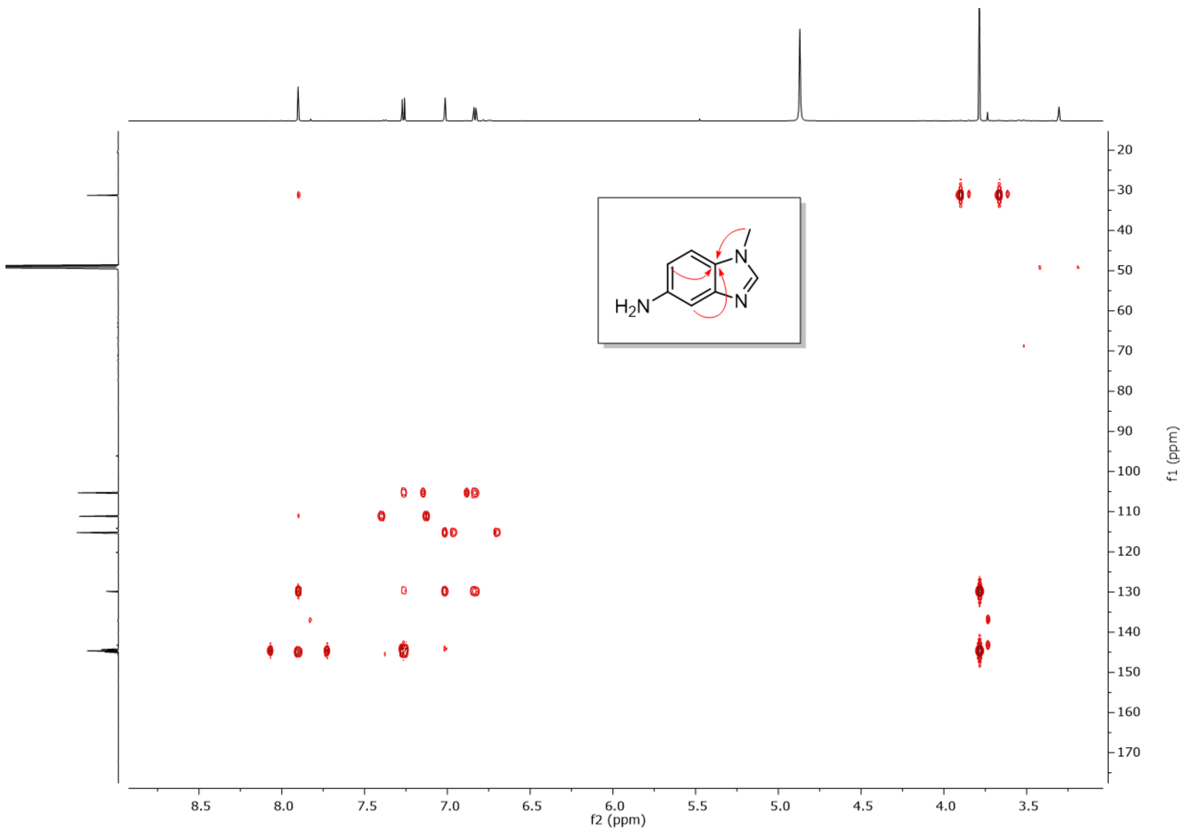


$^{1}H,^{13}C$ HMBC (600 MHz, $CD_3OD$) spectrum showing selected (H→C) correlations.

NMR spectra from enzymatic synthesis of 1-methyl-1*H*-benzo[*d*]imidazol-5-ol (**2**).

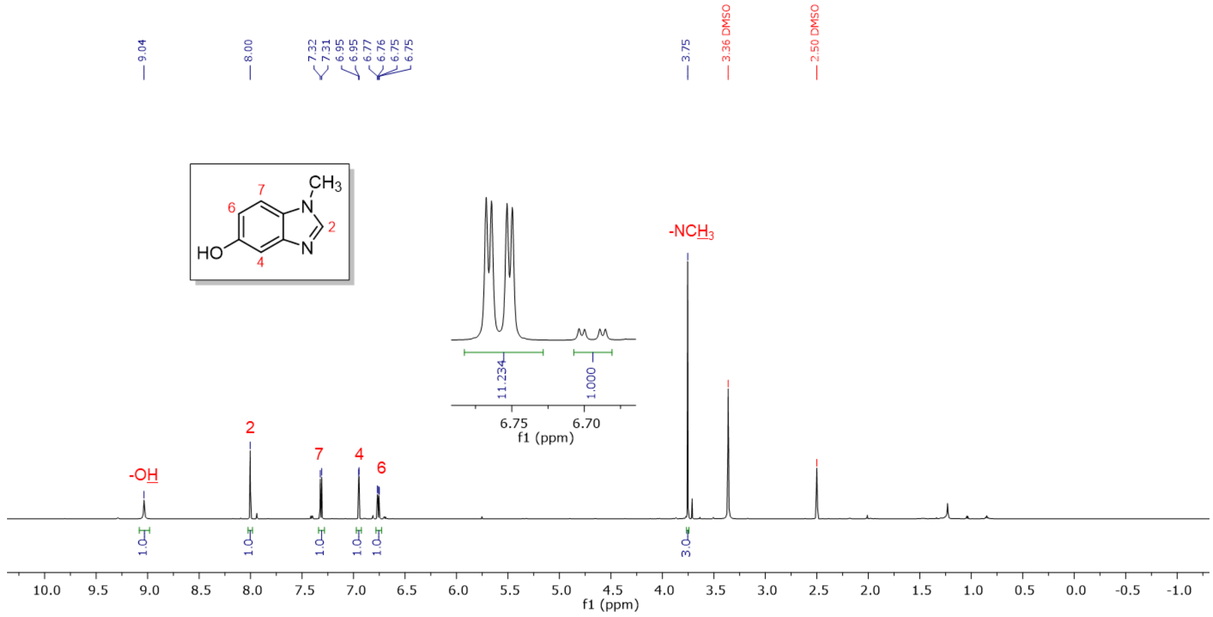


[1]H-NMR (600 MHz, DMSO-d6) spectrum.

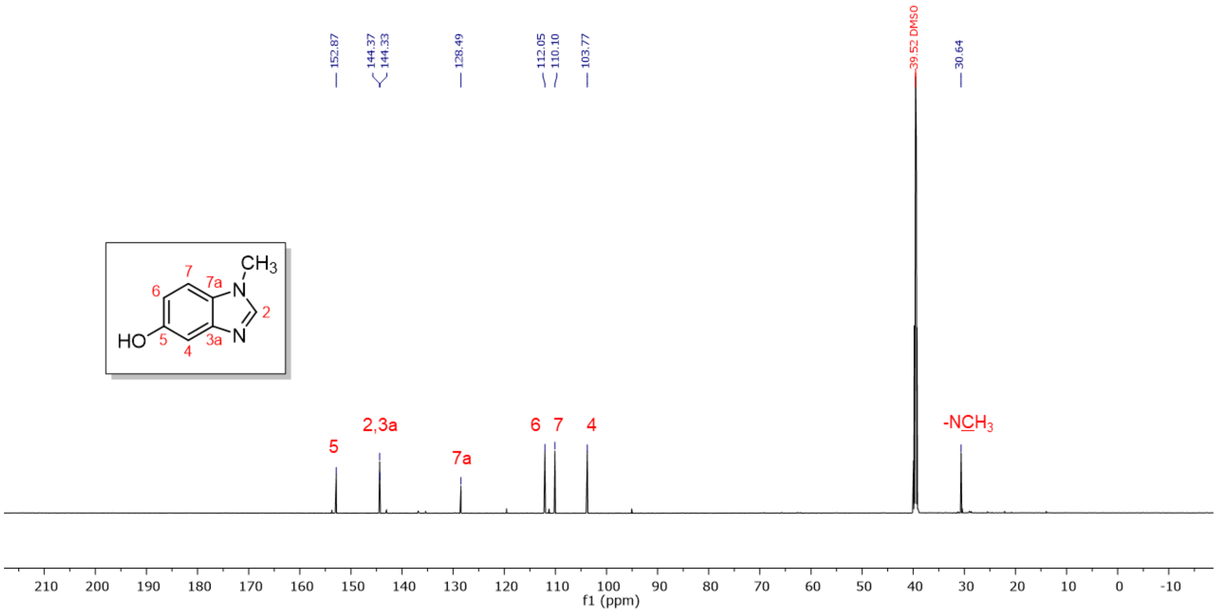


[13]C-NMR (151 MHz, DMSO-d6) spectrum.

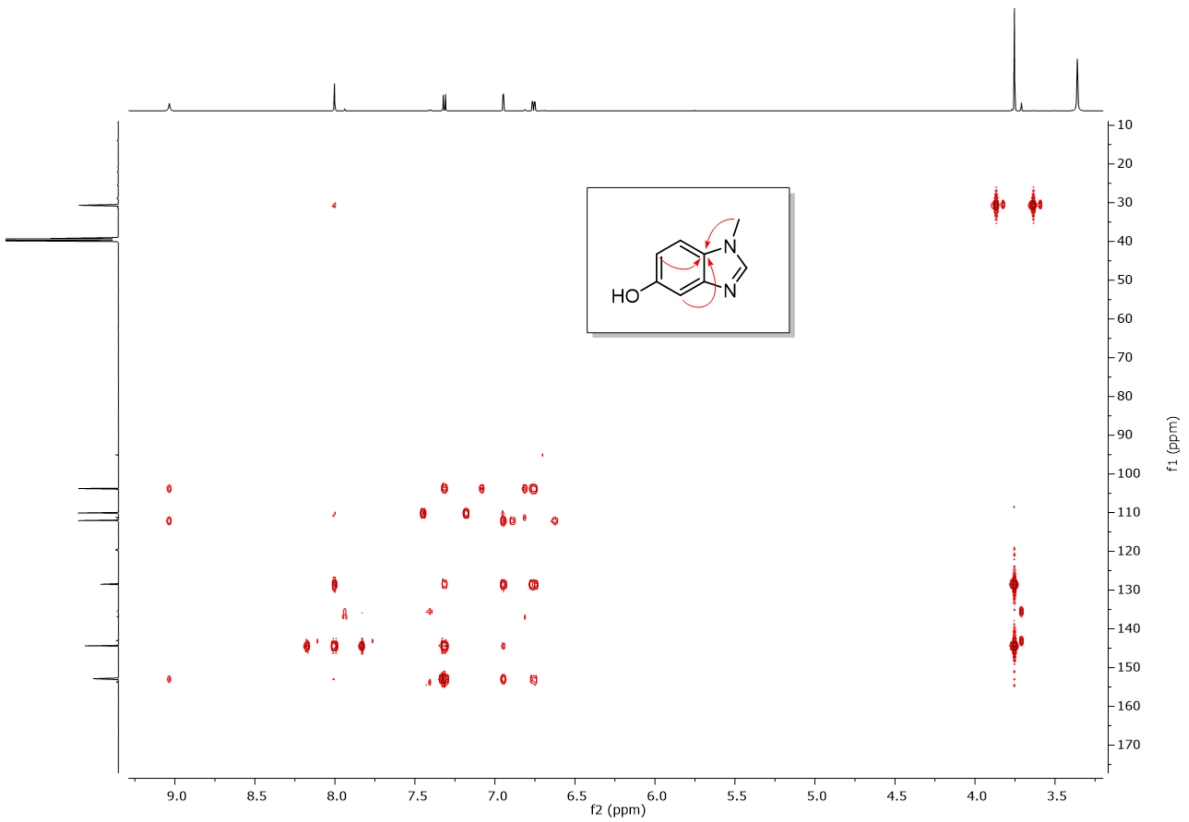


[1]H,[13]C HMBC (600 MHz, DMSO-d6) spectrum showing selected (H→C) correlations.

NMR spectra from enzymatic synthesis of 1-methyl-5-nitro-1H-benzo[d]imidazole (**3**).

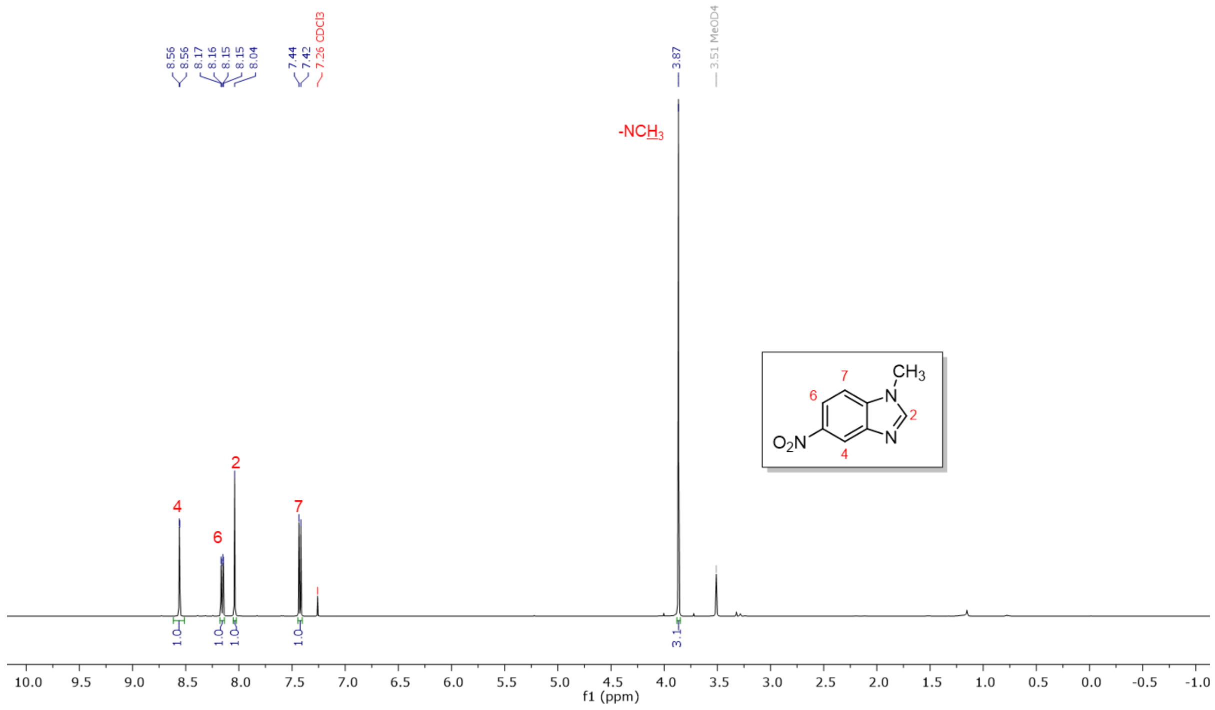


$^{1}$H-NMR (500 MHz, $CD_3OD$ / $CDCl_3$) spectrum.

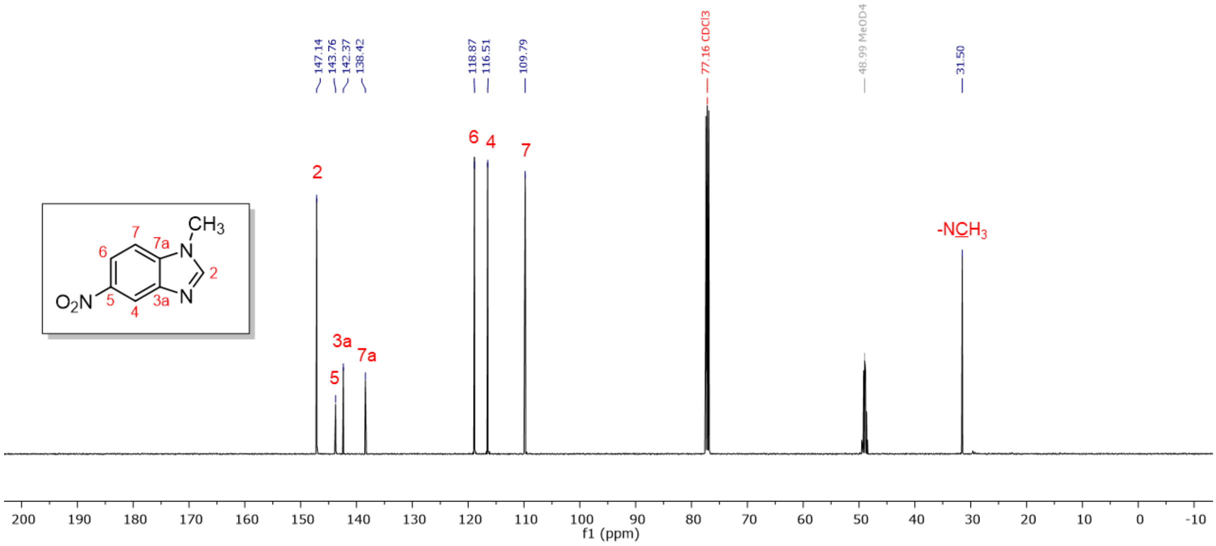


$^{13}$C-NMR (126 MHz, $CD_3OD$ / $CDCl_3$) spectrum.

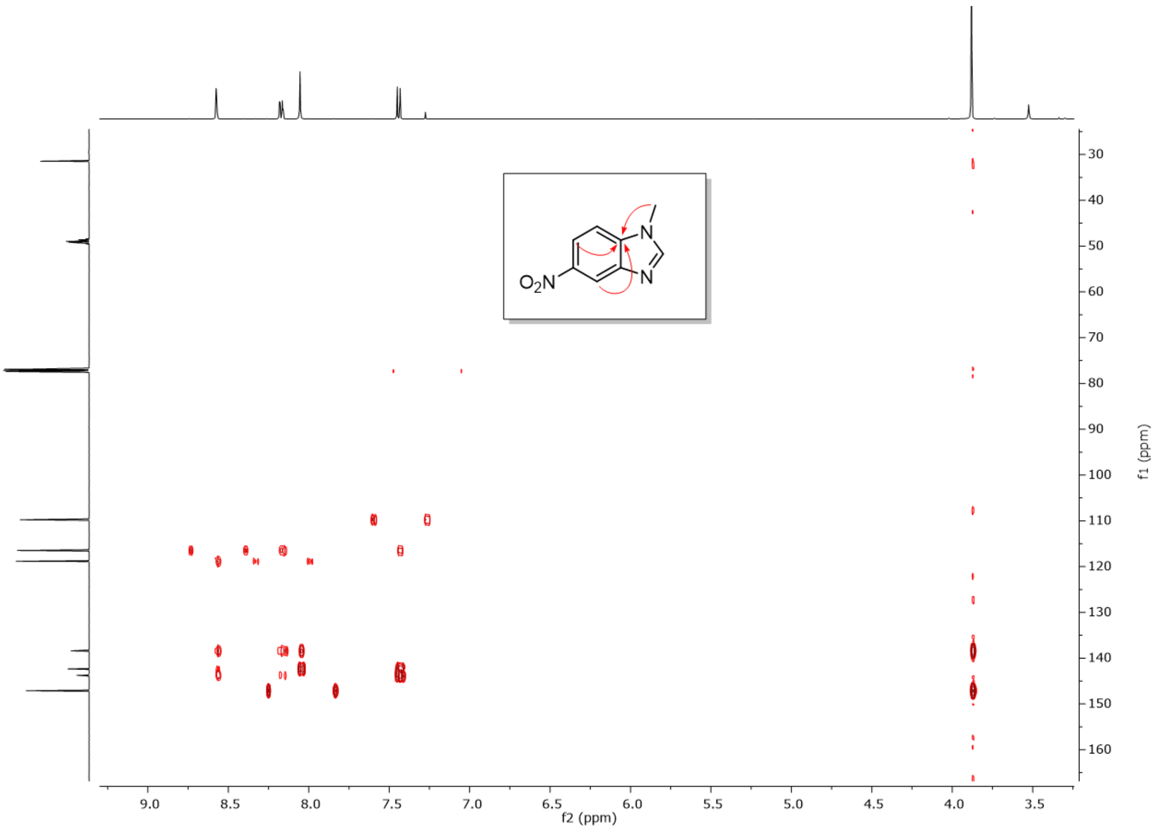


$^{1}H$,$^{13}C$ HMBC (500 MHz, $CD_3OD$ / $CDCl_3$) spectrum showing selected (H→C) correlations.

NMR spectra from enzymatic synthesis of 1-methyl-1*H*-benzo[*d*]imidazole-5-carbonitrile (**4**).

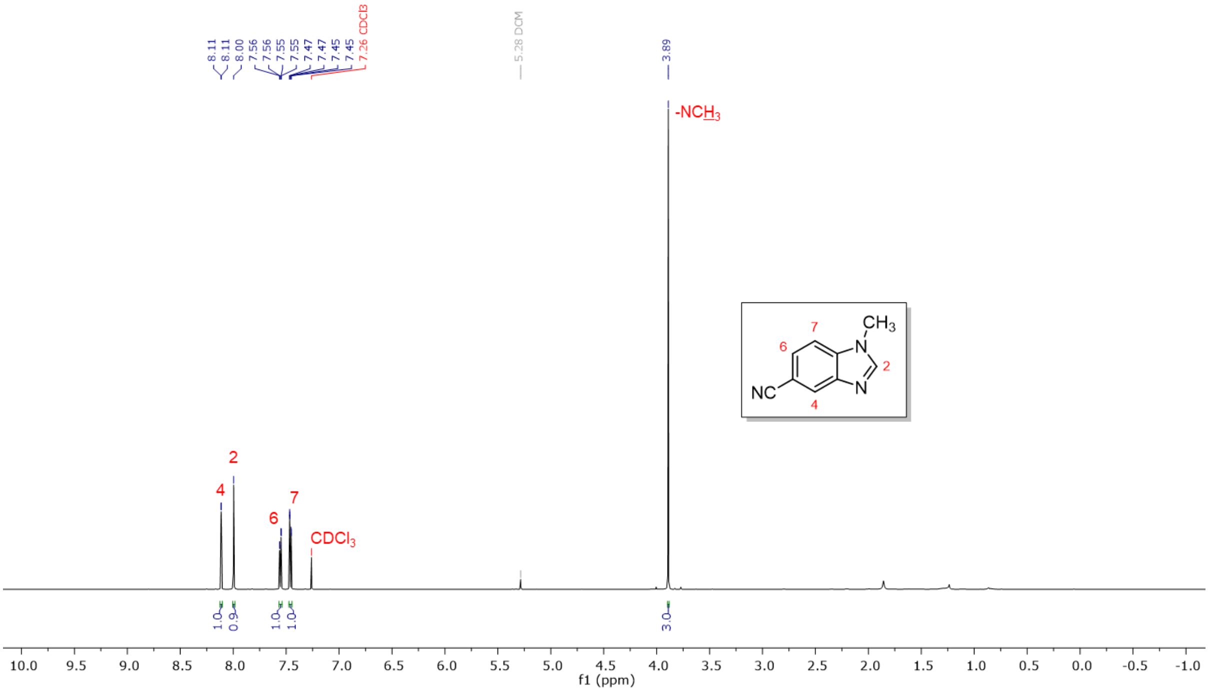


$^{1}$H-NMR (600 MHz, $CDCl_3$) spectrum.

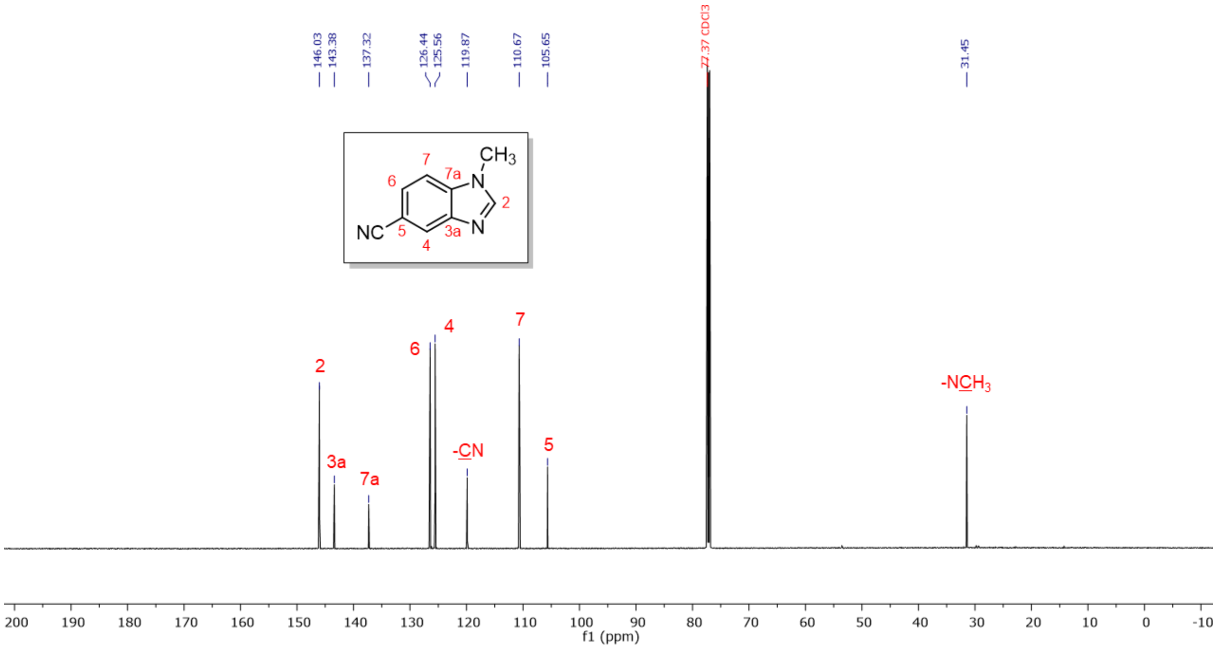


$^{13}$C-NMR (151 MHz, $CDCl_3$) spectrum.

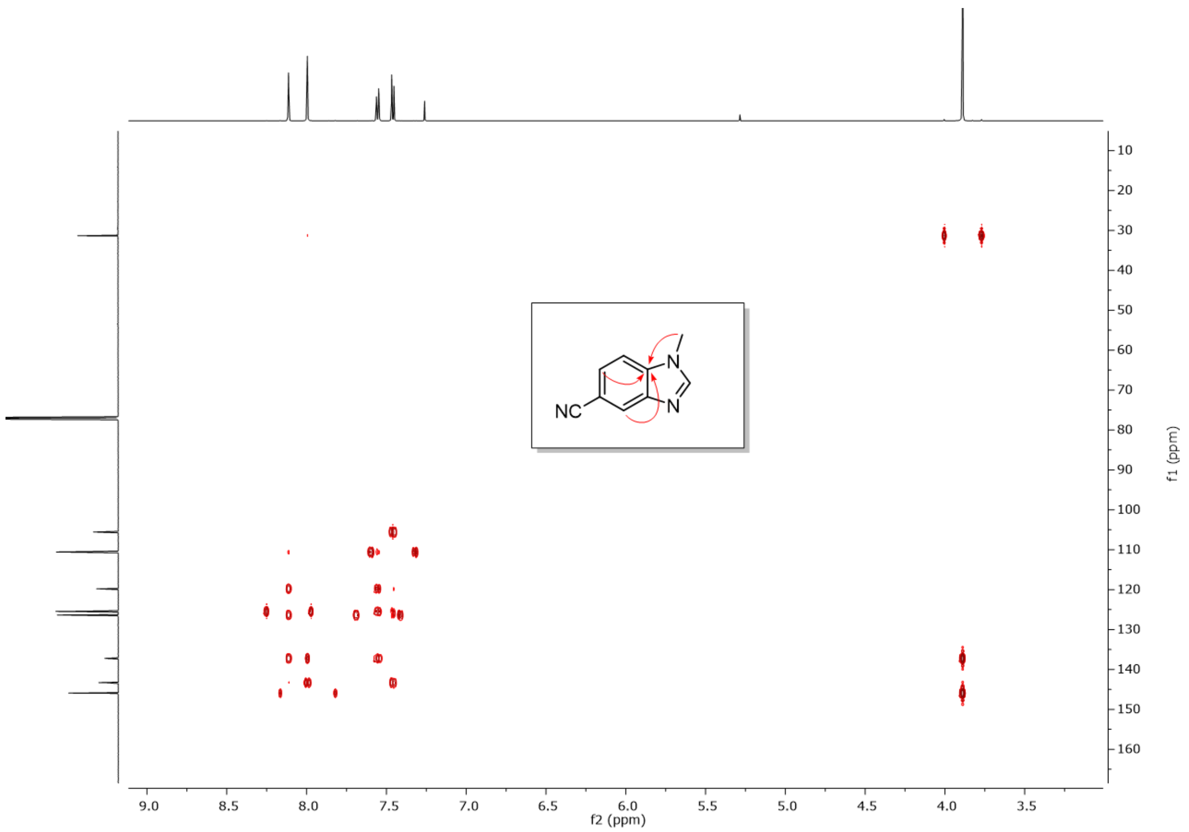


$^{1}H,^{13}C$ HMBC (600 MHz, $CDCl_3$) spectrum showing selected (H→C) correlations.

NMR spectra from enzymatic synthesis of 1-methyl-1*H*-benzo[*d*]imidazole-5-carbaldehyde (**5**).

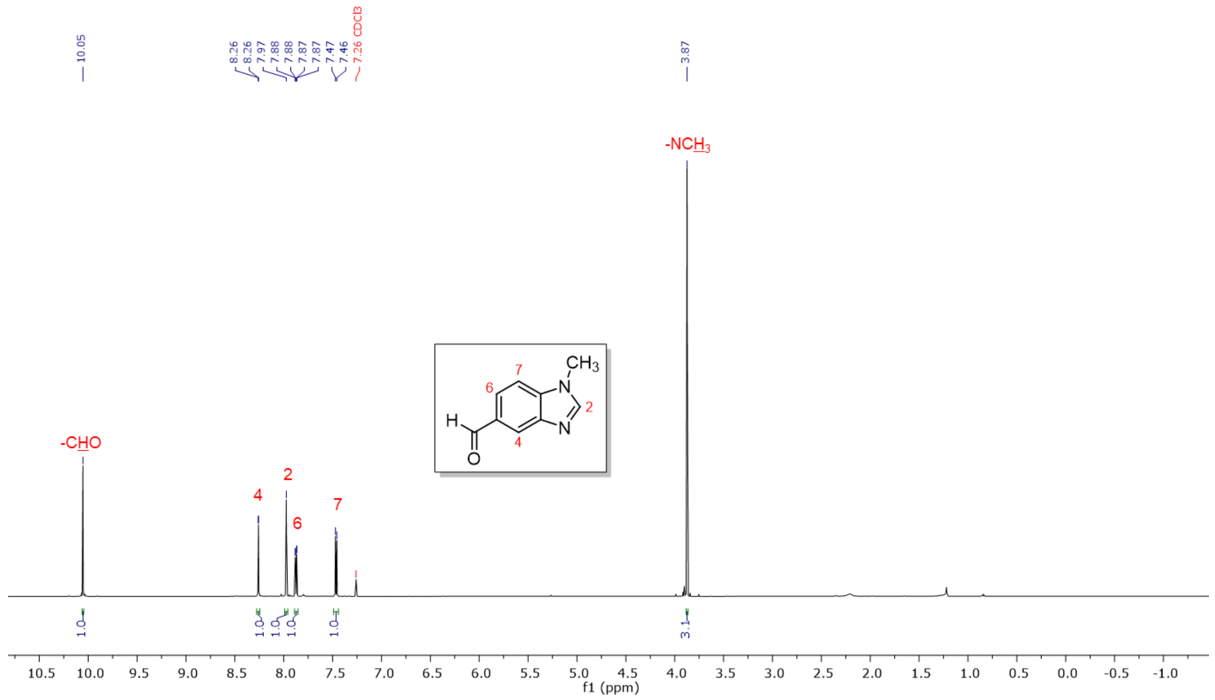


$^{1}$H-NMR (600 MHz, CDCl3) spectrum.

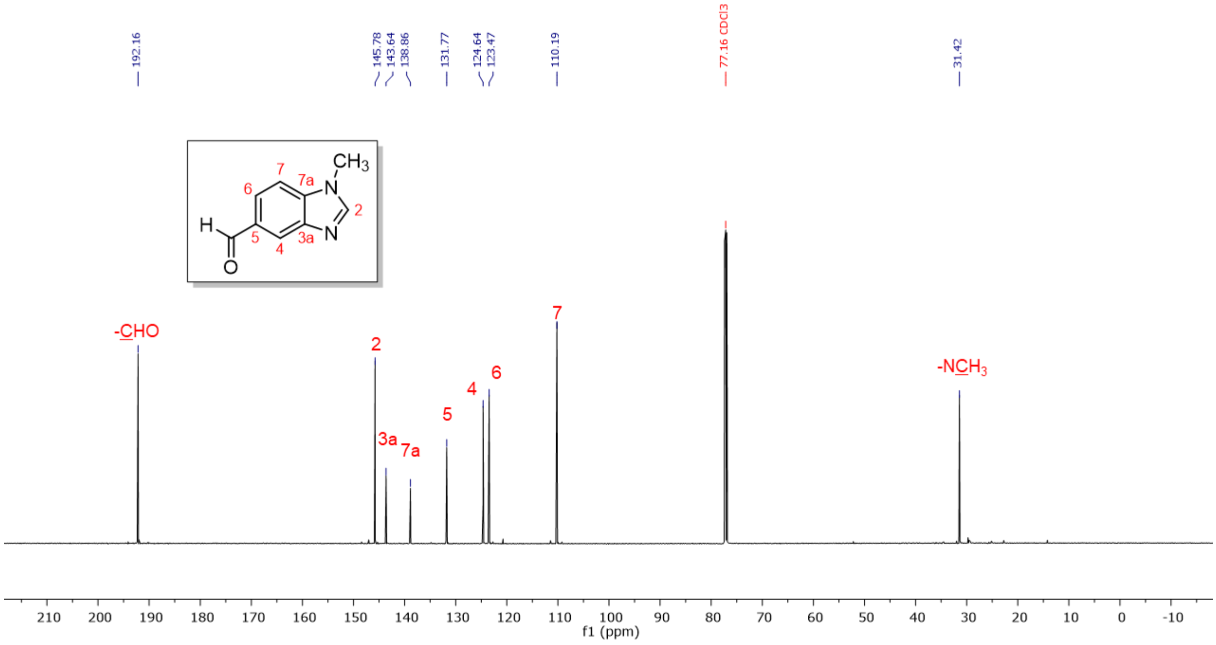


$^{13}$C-NMR (151 MHz, CDCl3) spectrum.

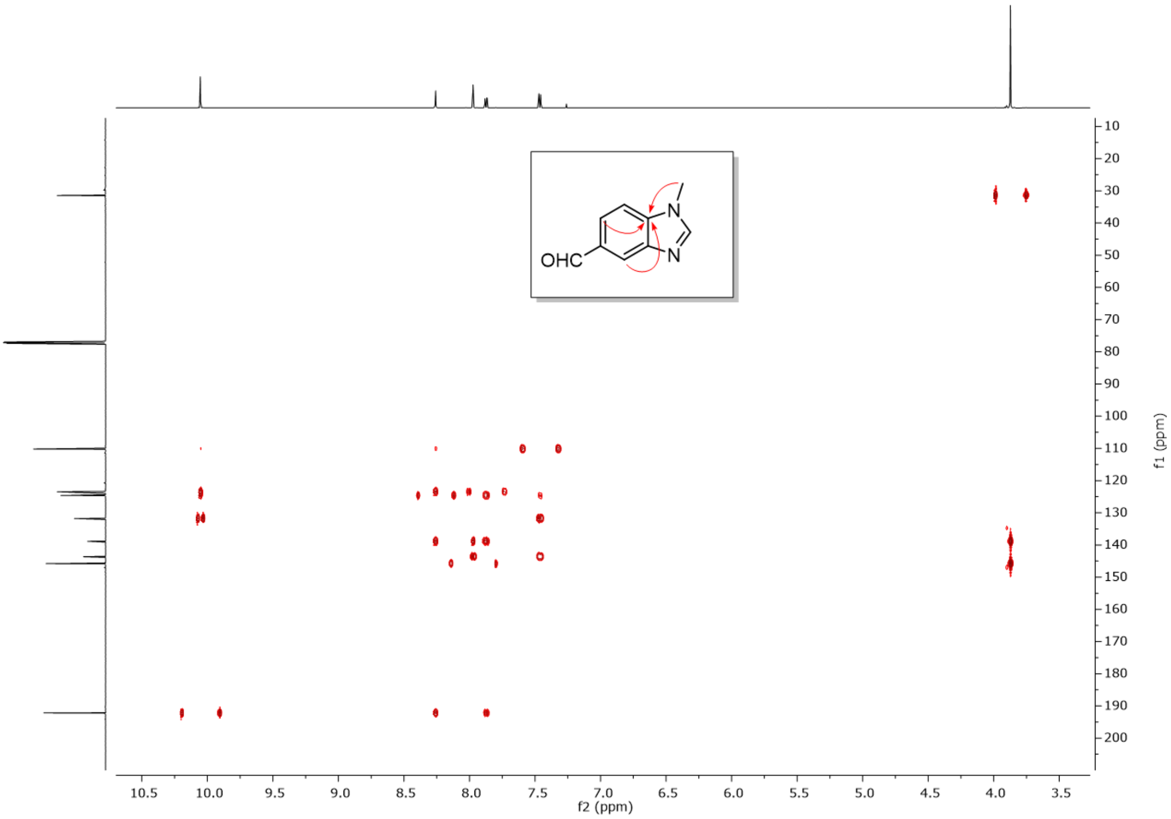


$^{1}H,^{13}C$ HMBC (600 MHz, $CDCl_3$) spectrum showing selected (H→C) correlations.

NMR spectra from enzymatic synthesis of 5-methoxy-1-methyl-1*H*-benzo[*d*]imidazole (**6**).

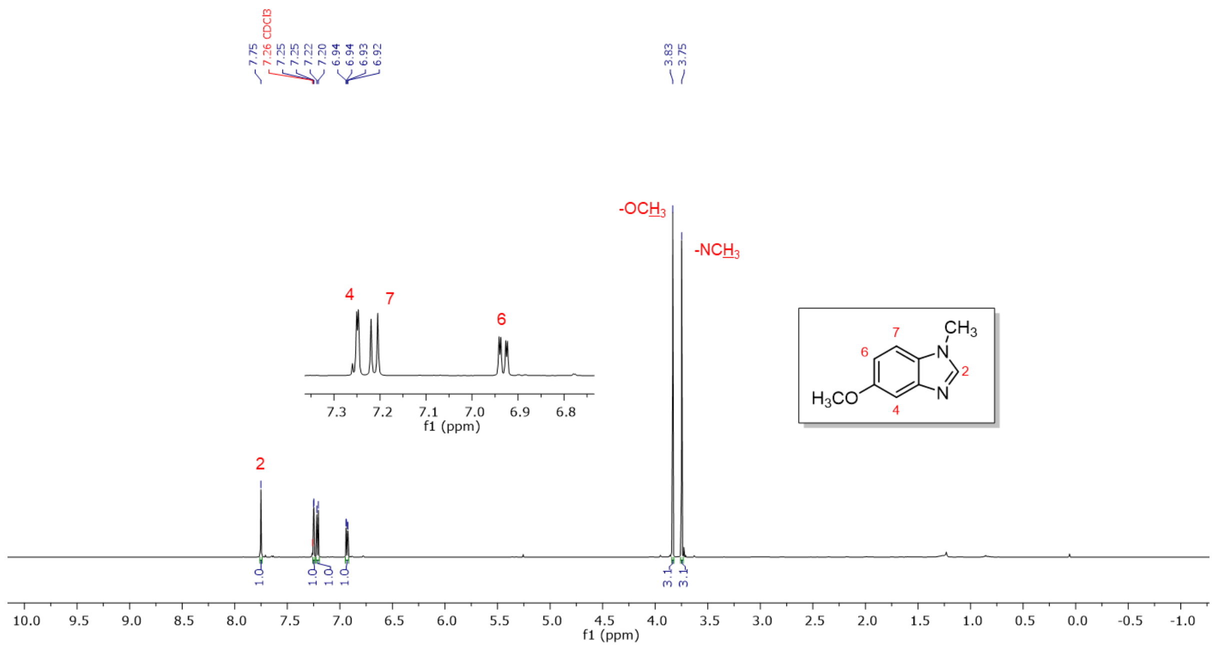


$^{1}$H-NMR (600 MHz, $CDCl_3$) spectrum.

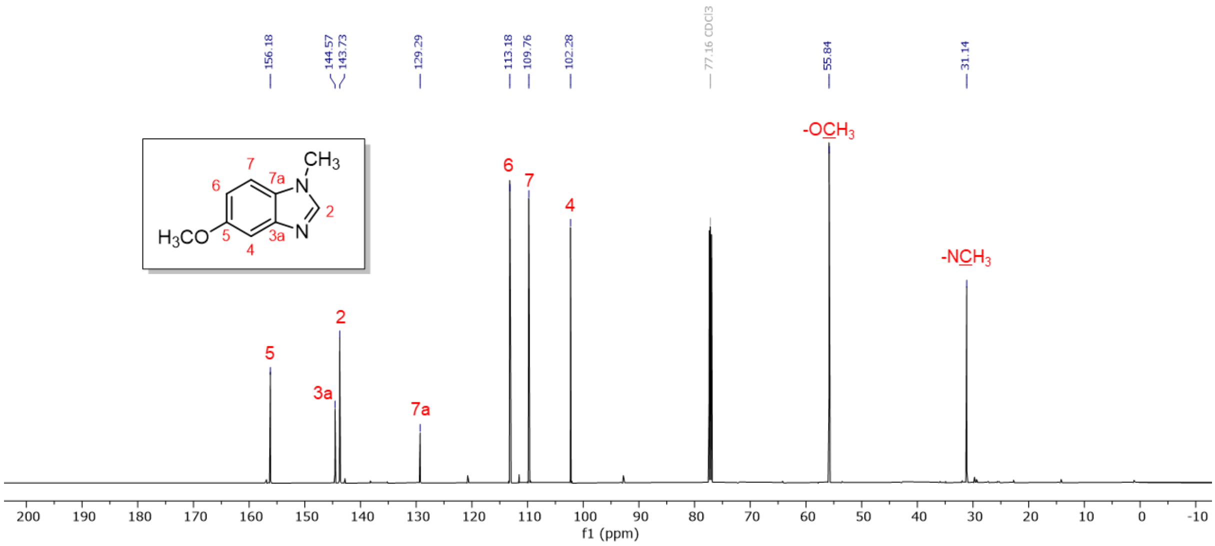


$^{13}$C-NMR (151 MHz, $CDCl_3$) spectrum.

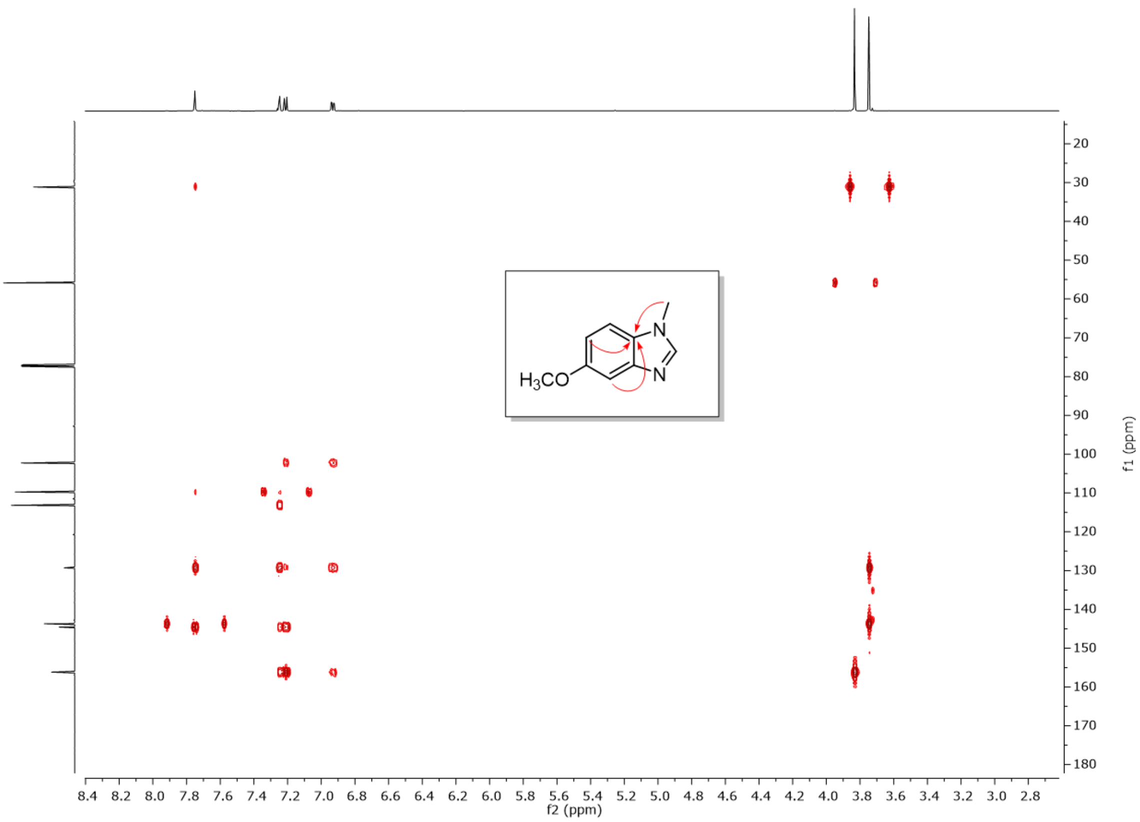


$^{1}H,^{13}C$ HMBC (600 MHz, $CDCl_3$) spectrum showing selected (H→C) correlations.

NMR spectra from enzymatic synthesis of 5-fluoro-1-methyl-1*H*-benzo[*d*]imidazole (**7**).

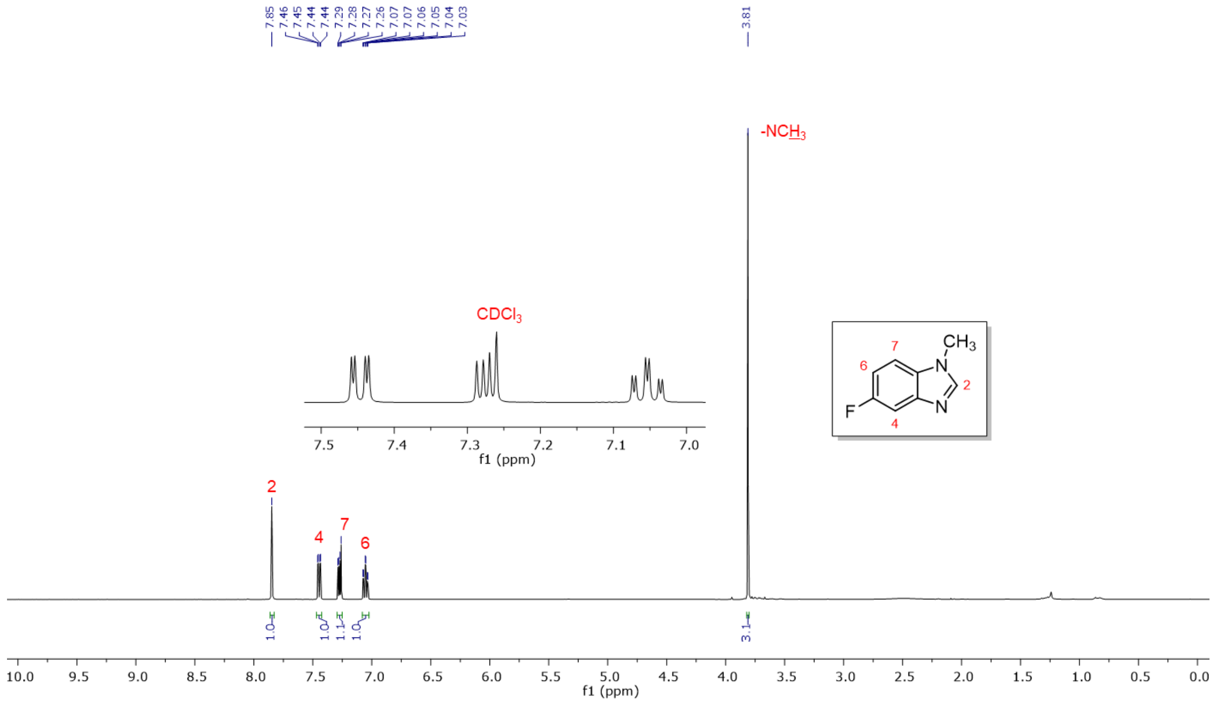


$^{1}$H-NMR (500 MHz, $CDCl_3$) spectrum.

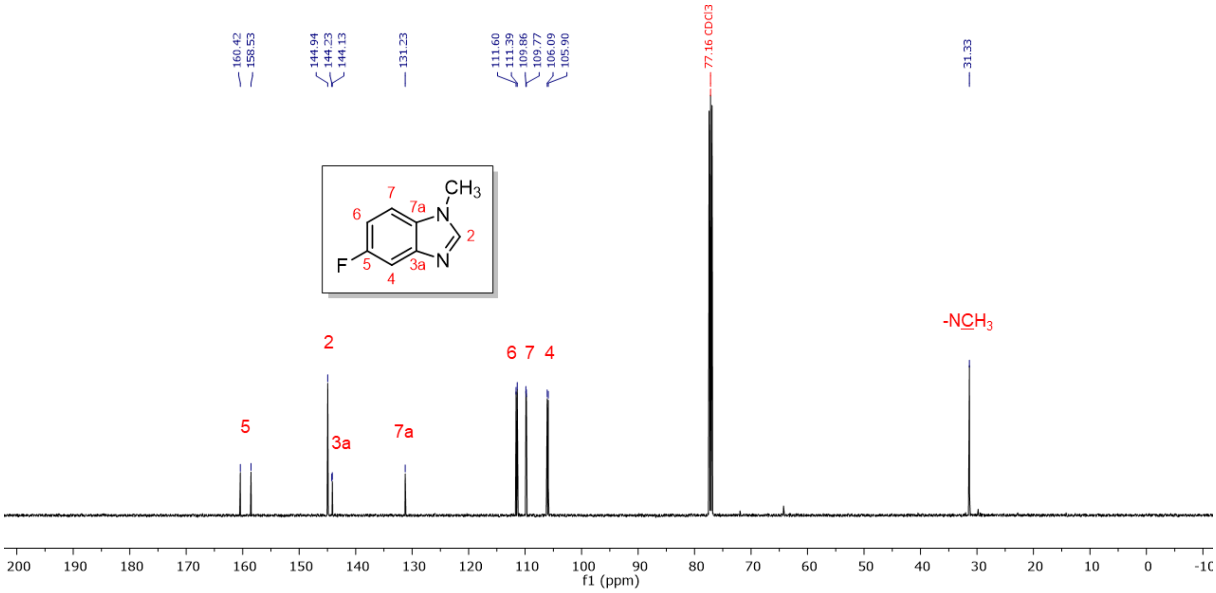


$^{13}$C-NMR (126 MHz, $CDCl_3$) spectrum.

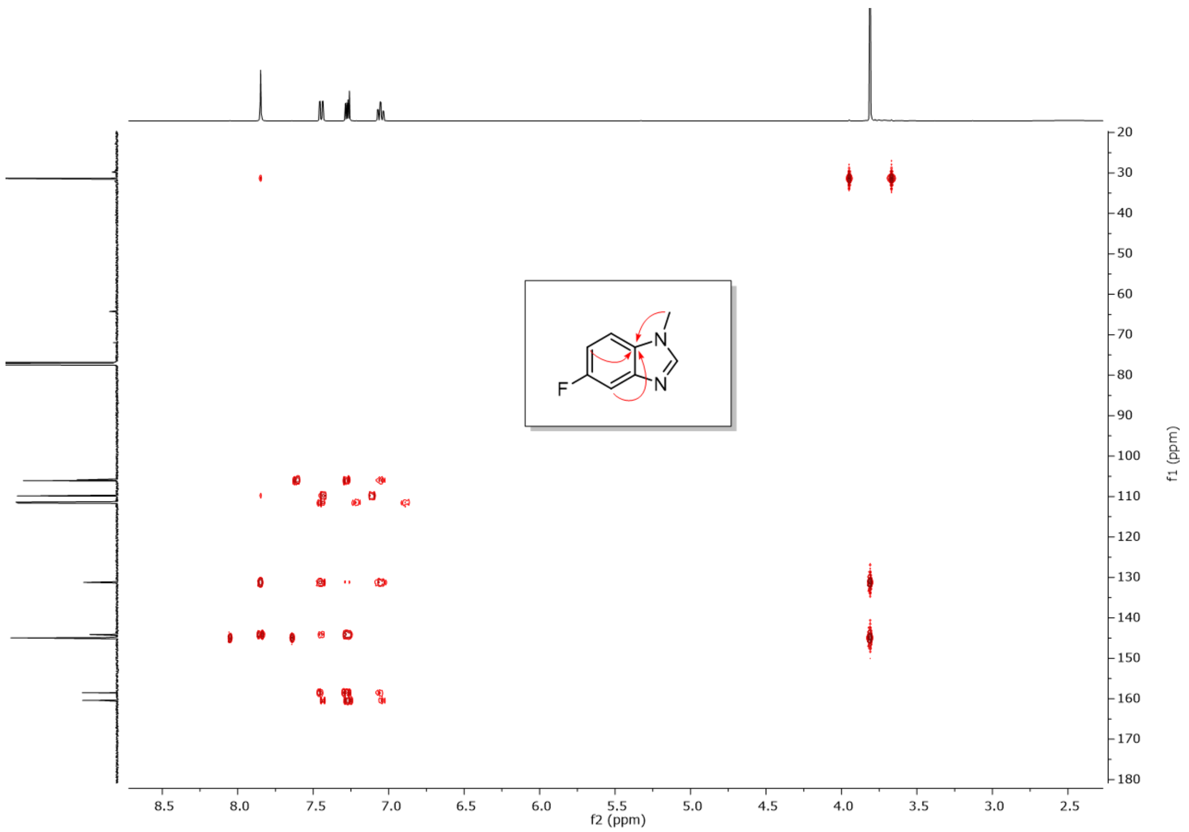


$^1H,^{13}C$ HMBC (500 MHz, $CDCl_3$) spectrum showing selected (H→C) correlations.

NMR spectra from enzymatic synthesis of 5-chloro-1-methyl-1*H*-benzo[*d*]imidazole (**8**)

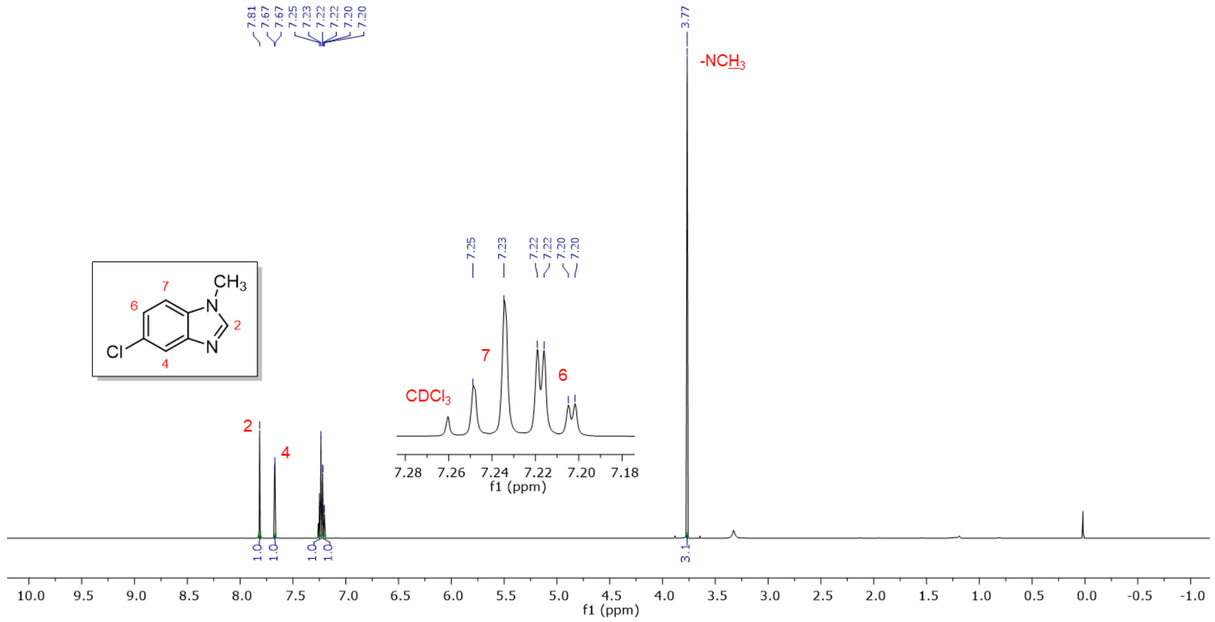


$^{1}$H-NMR (600 MHz, $CD_3OD$ / $CDCl_3$) spectrum.

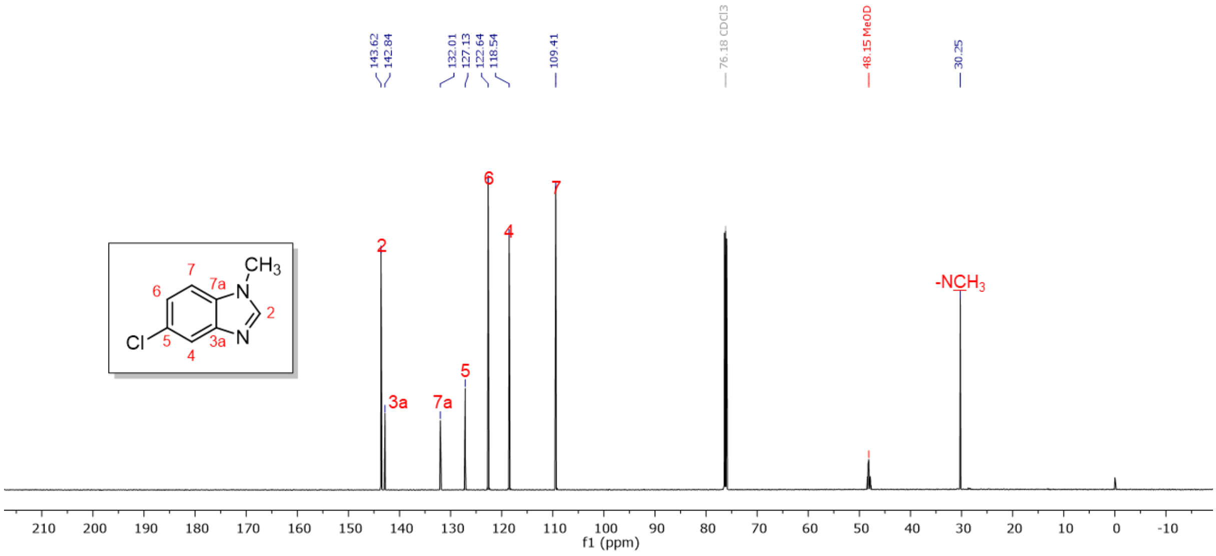


$^{13}$C-NMR (151 MHz, $CD_3OD$ / $CDCl_3$) spectrum.

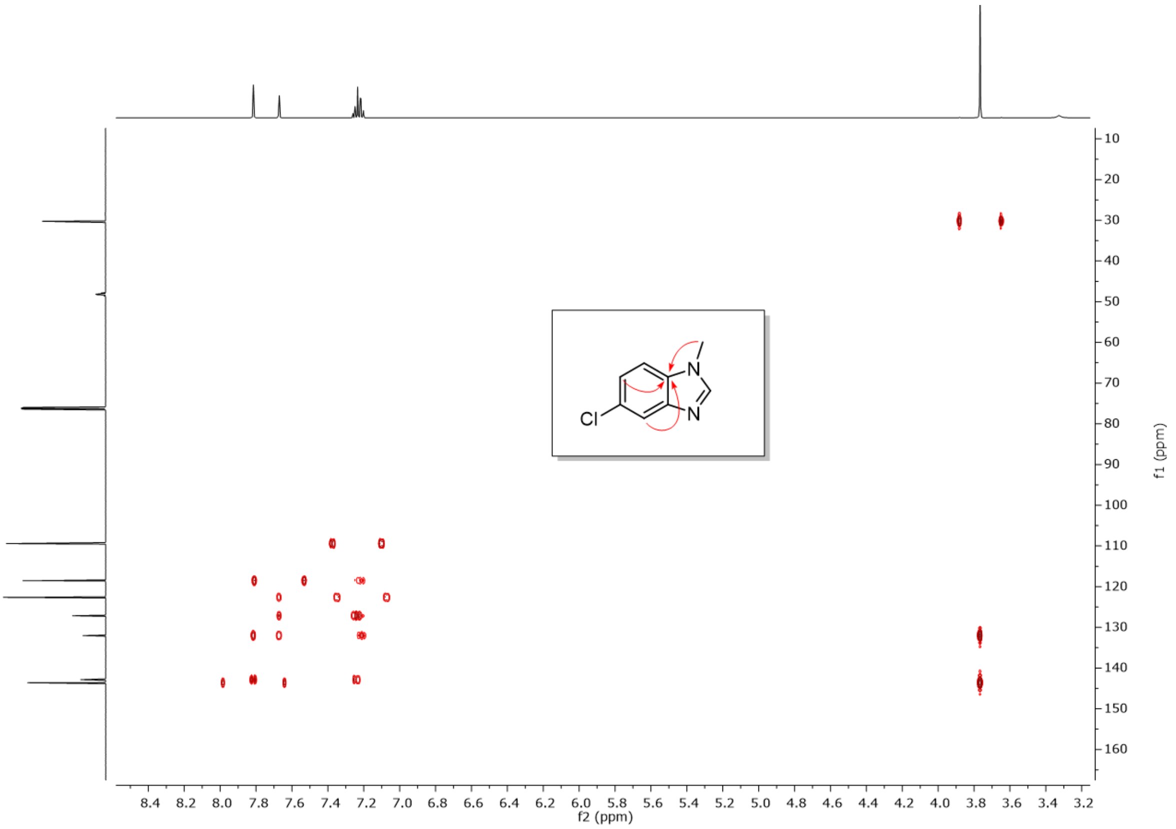


$^{1}H,^{13}C$ HMBC (600 MHz, $CD_3OD$ / $CDCl_3$) spectrum showing selected (H→C) correlations.

<u>NMR spectra from enzymatic synthesis of 5-iodo-1-methyl-1*H*-benzo[*d*]imidazole (**9**).</u>

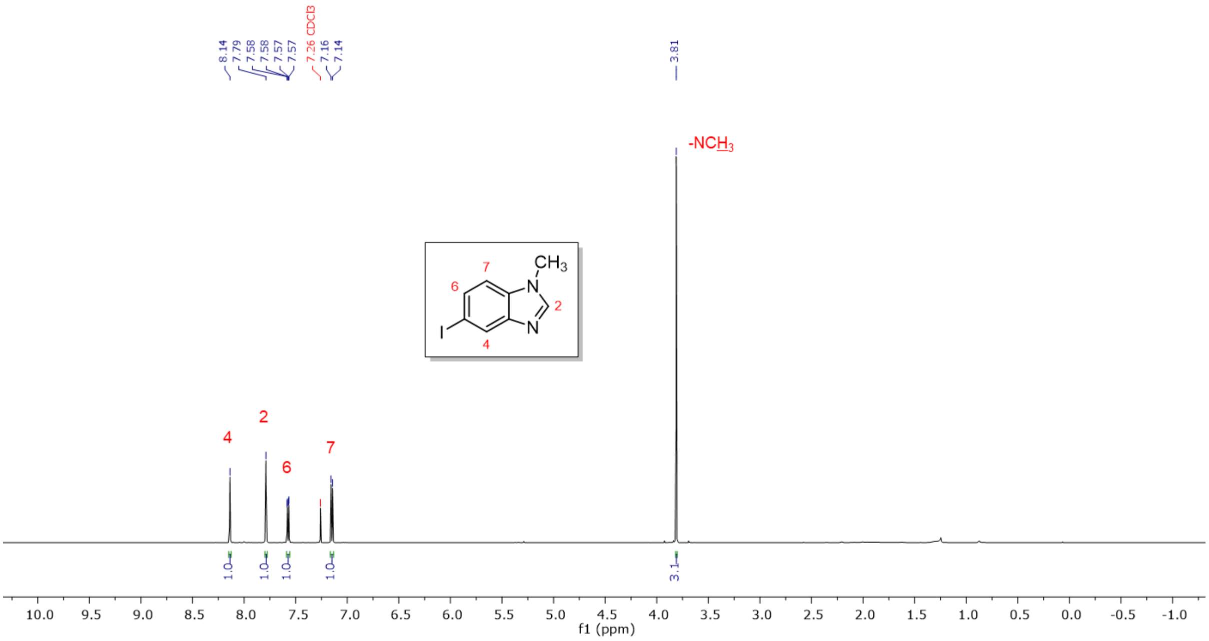


$^1$H-NMR (600 MHz, $CDCl_3$) spectrum.

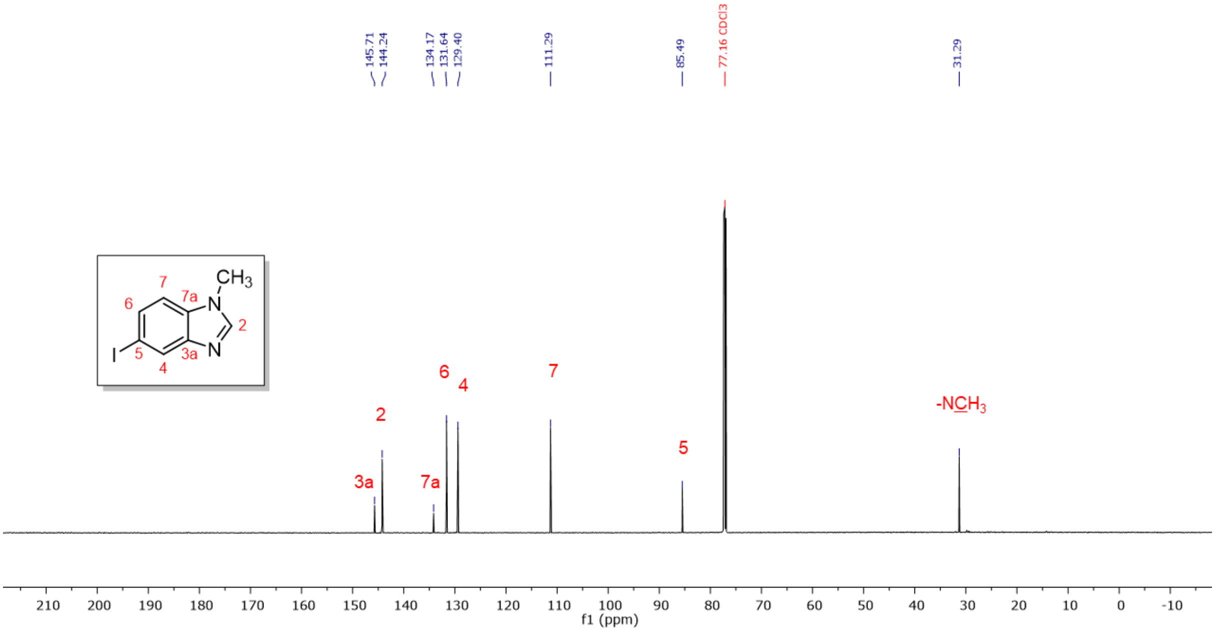


$^{13}$C-NMR (151 MHz, $CDCl_3$) spectrum.

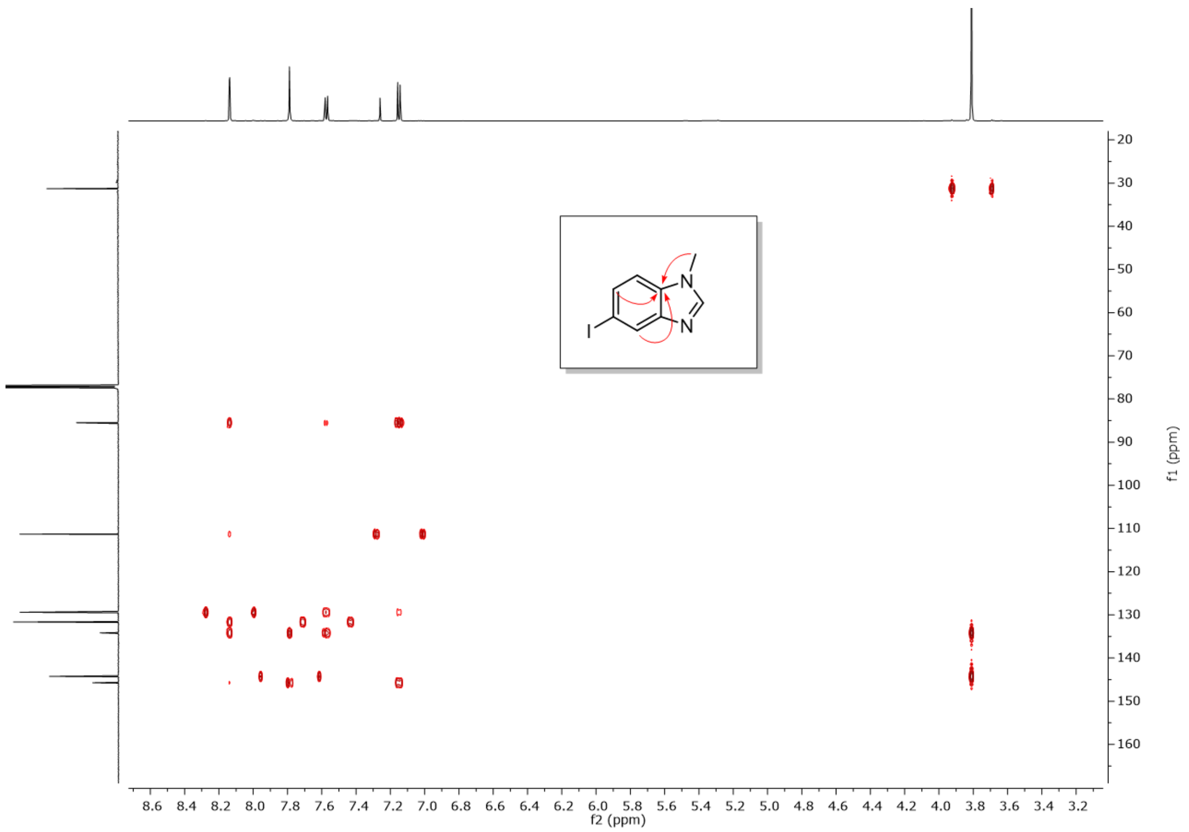


[1]H,[13]C HMBC (600 MHz, $CDCl_3$) spectrum showing selected (H→C) correlations.

NMR spectra from enzymatic synthesis of 5-bromo-1,2-dimethyl-1*H*-benzo[*d*]imidazole (**10**).

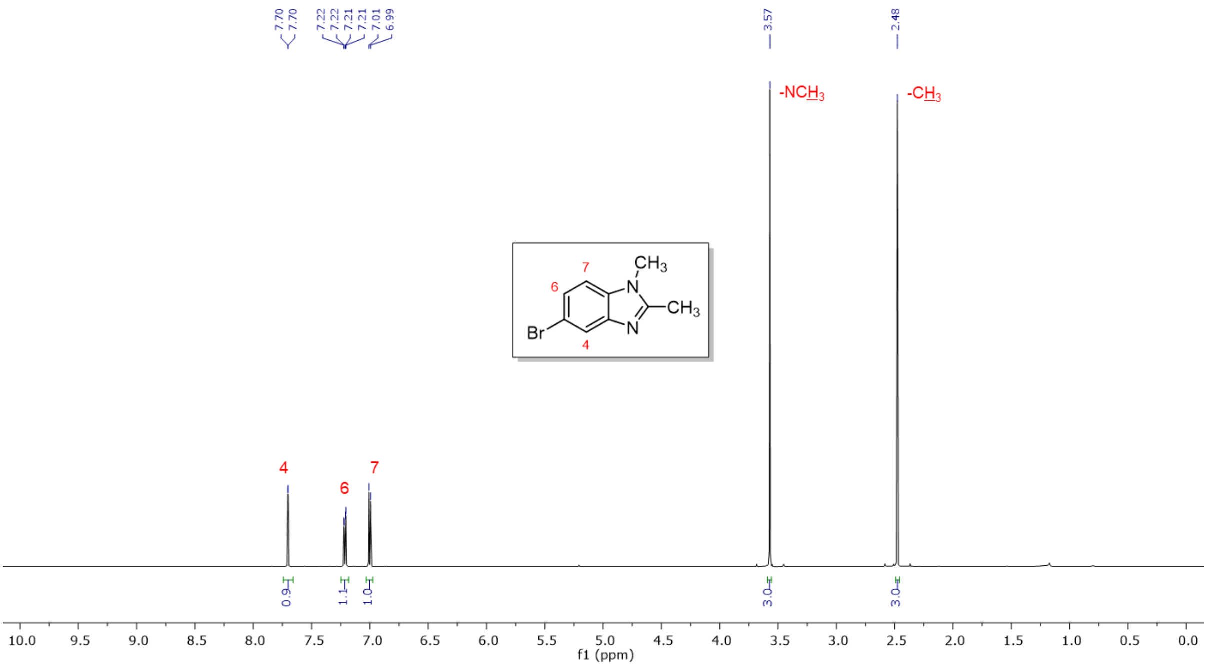


[1]H-NMR (600 MHz, $CDCl_3$) spectrum.

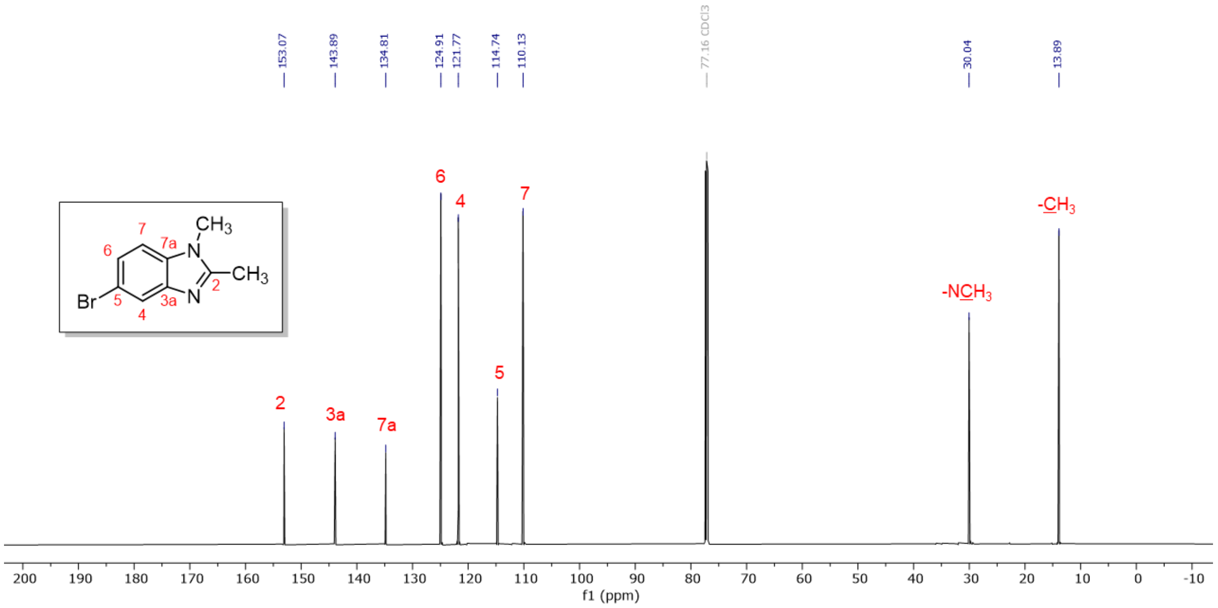


[13]C-NMR (151 MHz, $CDCl_3$) spectrum.

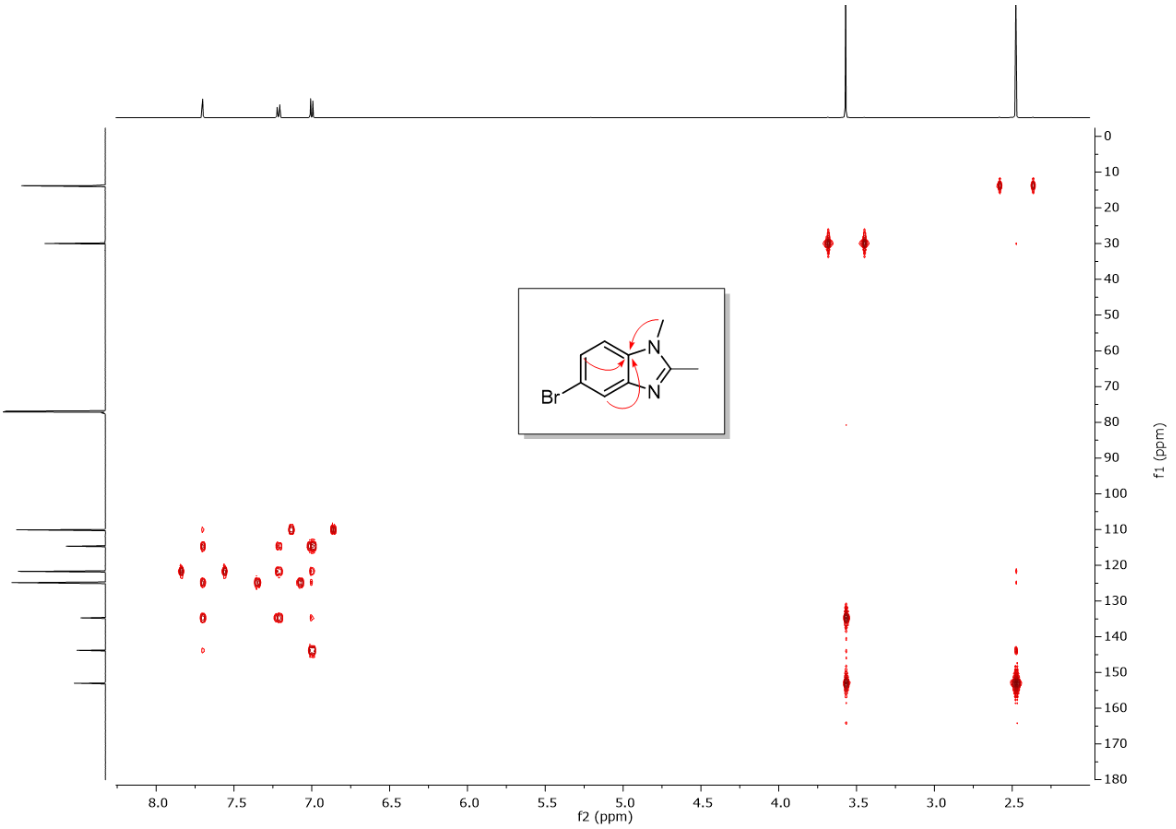


$^1H,^{13}C$ HMBC (600 MHz, $CDCl_3$) spectrum showing selected (H→C) correlations.

NMR spectra from enzymatic synthesis of 6-bromo-1-methyl-1*H*-benzo[*d*]imidazole (**11**).

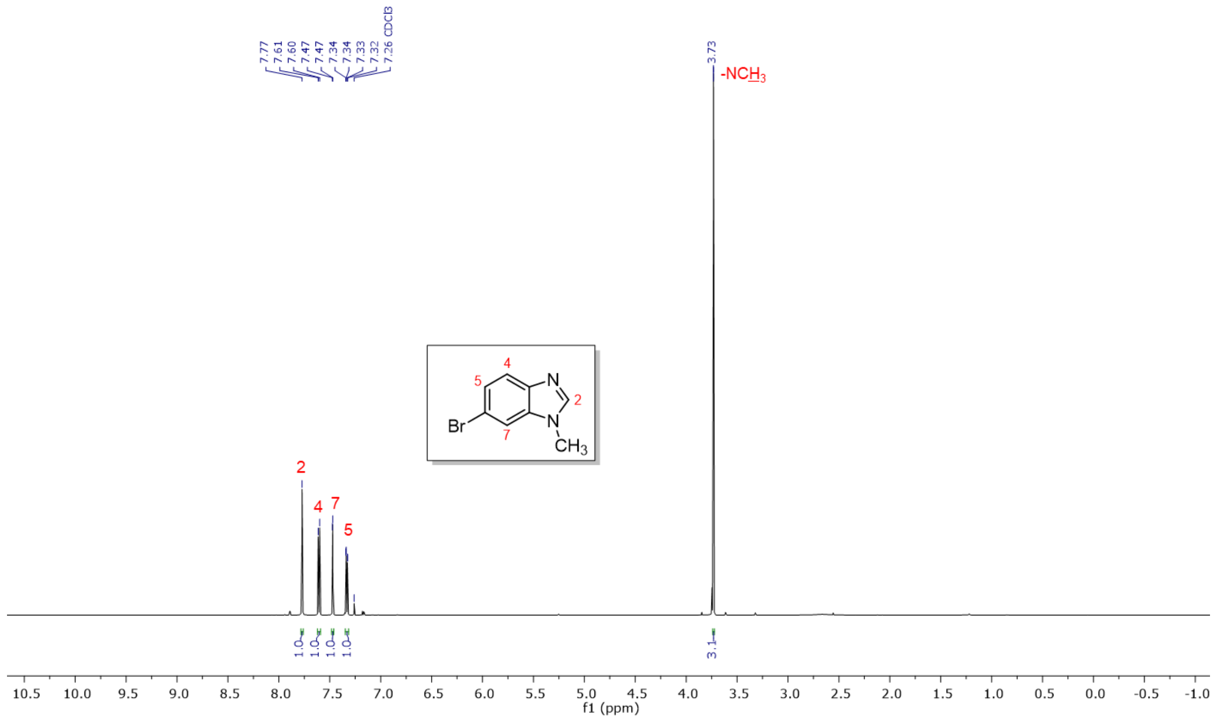


$^{1}$H-NMR (600 MHz, $CDCl_3$) spectrum.

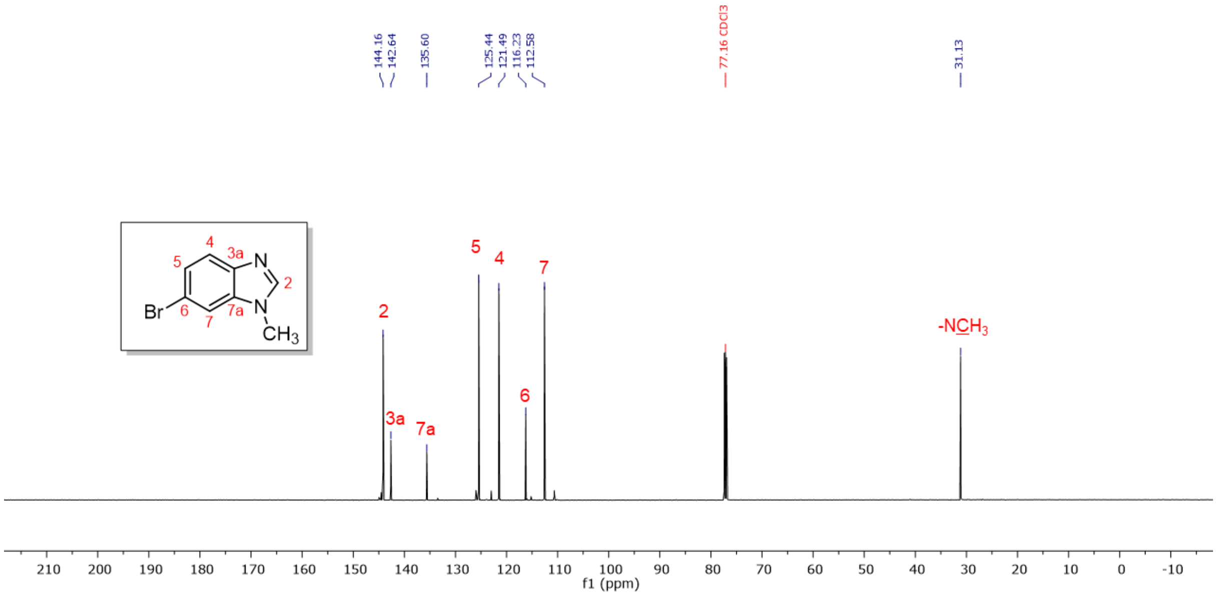


$^{13}$C-NMR (151 MHz, $CDCl_3$) spectrum.

NMR spectra from chemical synthesis of 5-(7*H*-pyrrolo[2,3-*d*]pyrimidin-4-yl)-4,5,6,7-tetrahydro-1*H*-imidazo[4,5-*c*]pyridine (**12**).

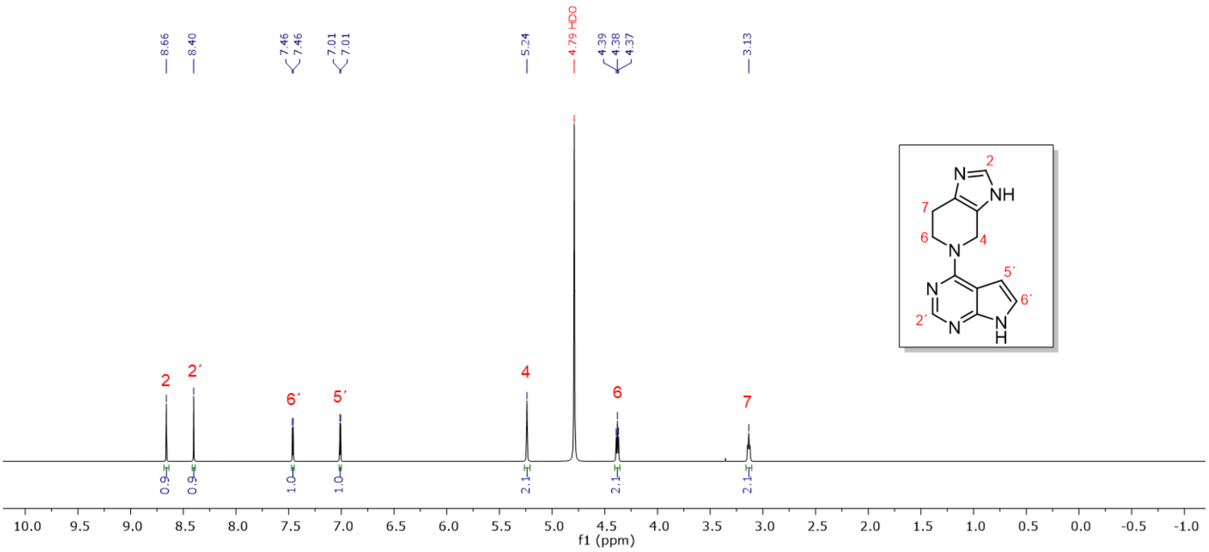


[1]H-NMR (500 MHz, $D_2O$) spectrum.

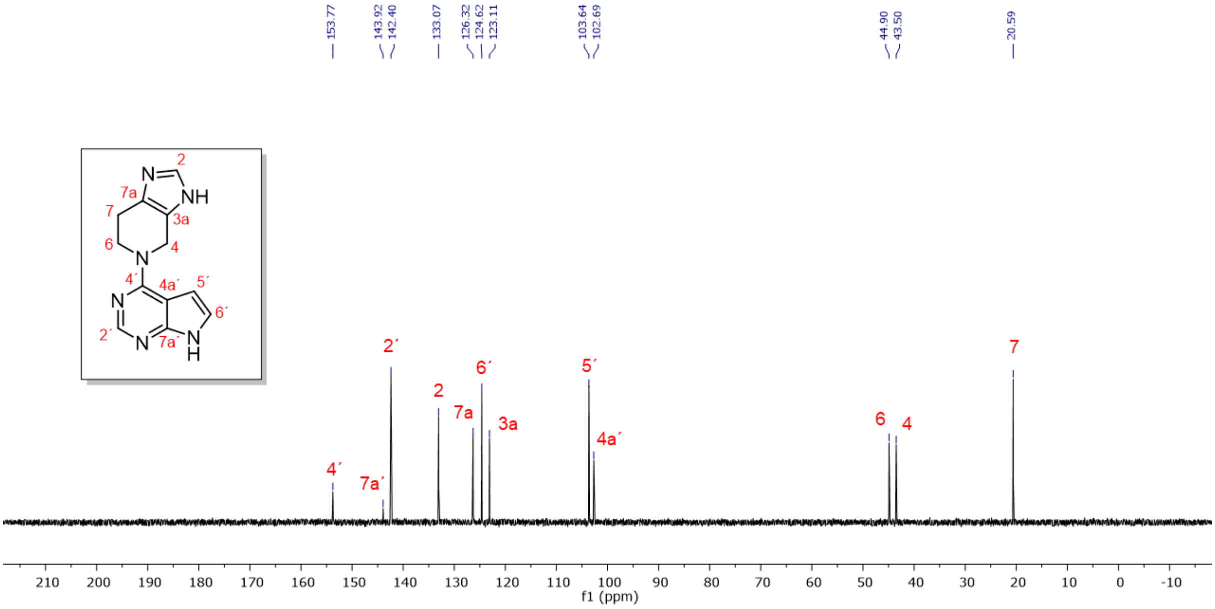


[13]C-NMR (126 MHz, $D_2O$) spectrum.

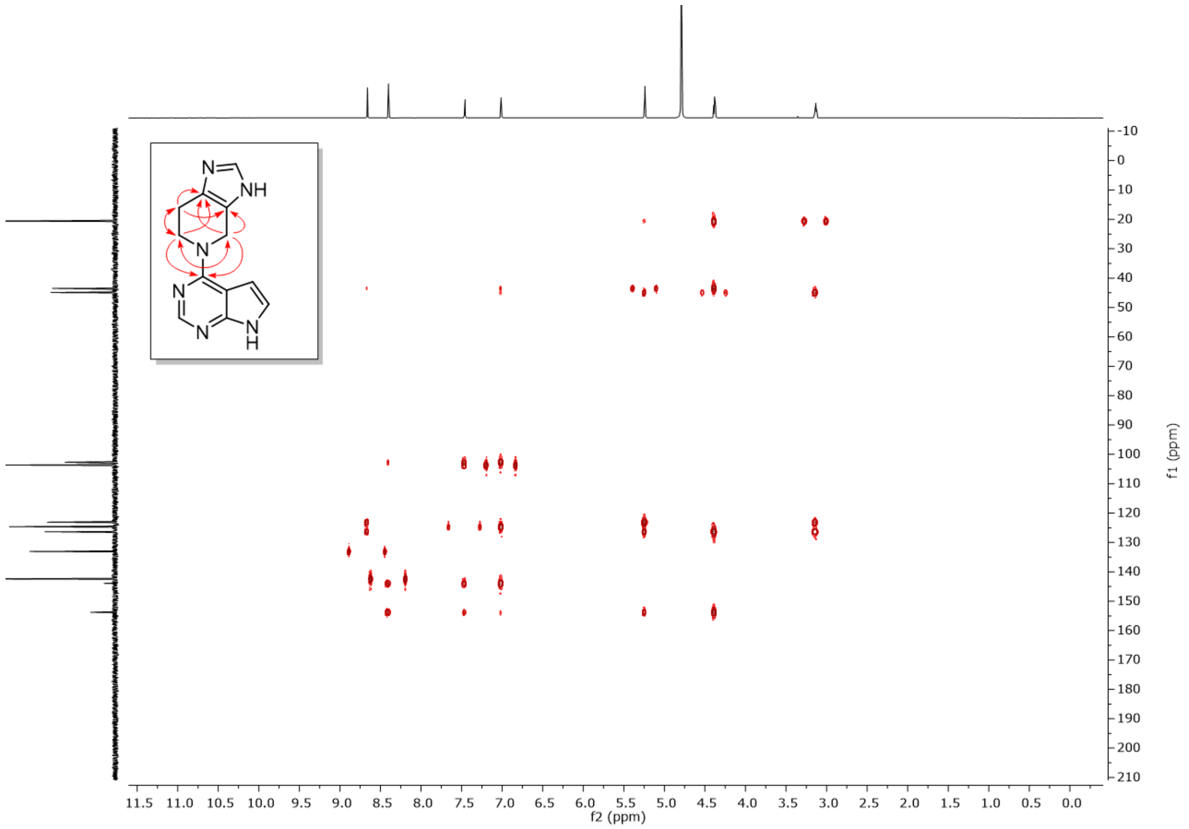

$^{1}H,^{13}C$ HMBC (500 MHz, $D_2O$) spectrum showing selected (H→C) correlations.

NMR spectra from enzymatic synthesis of 5-(1-methyl-1*H*-pyrrolo[2,3-*d*]pyrimidin-4-yl)-4,5,6,7-tetrahydro-1*H*-imidazo[4,5-*c*]pyridine (**13**).

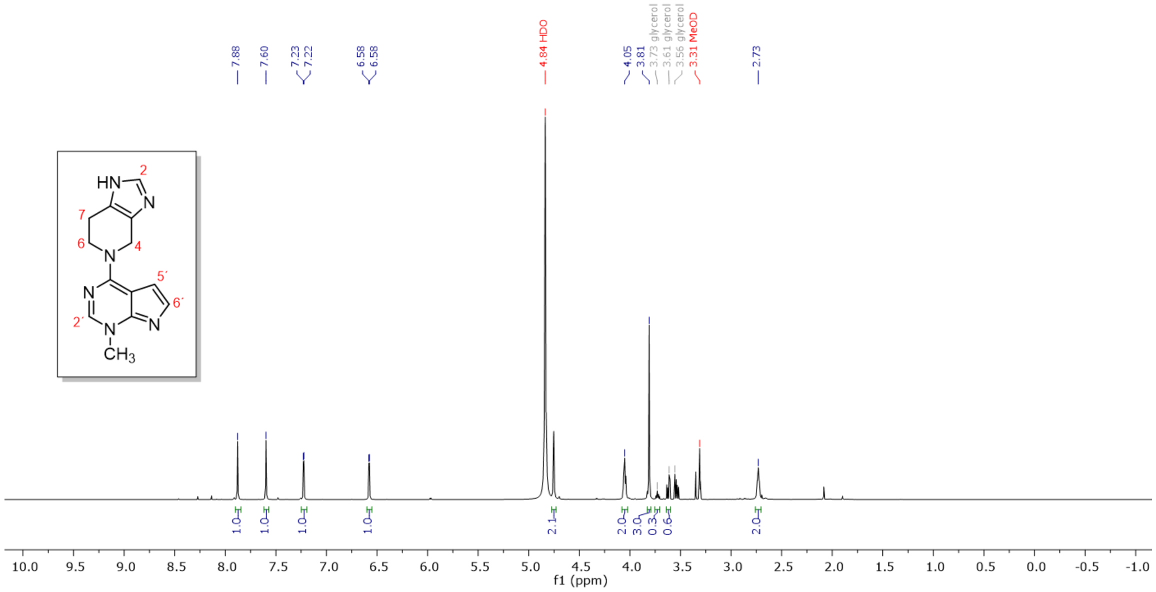


$^{1}$H-NMR (500 MHz, $D_2O$ / $CD_3OD$) spectrum.

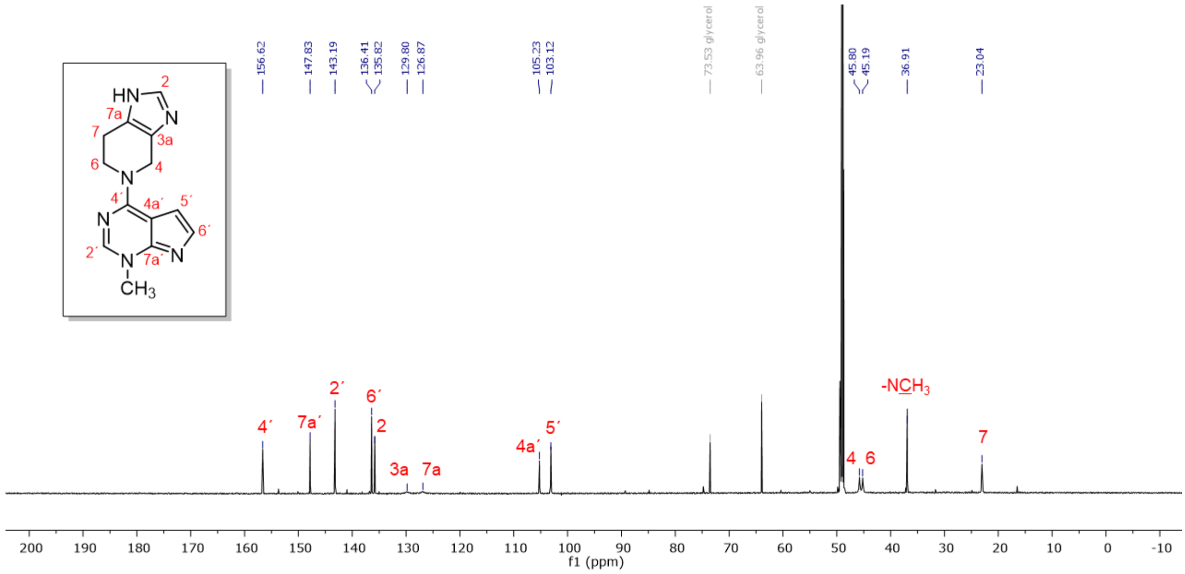


$^{13}$C-NMR (151 MHz, $D_2O$ / $CD_3OD$) spectrum.

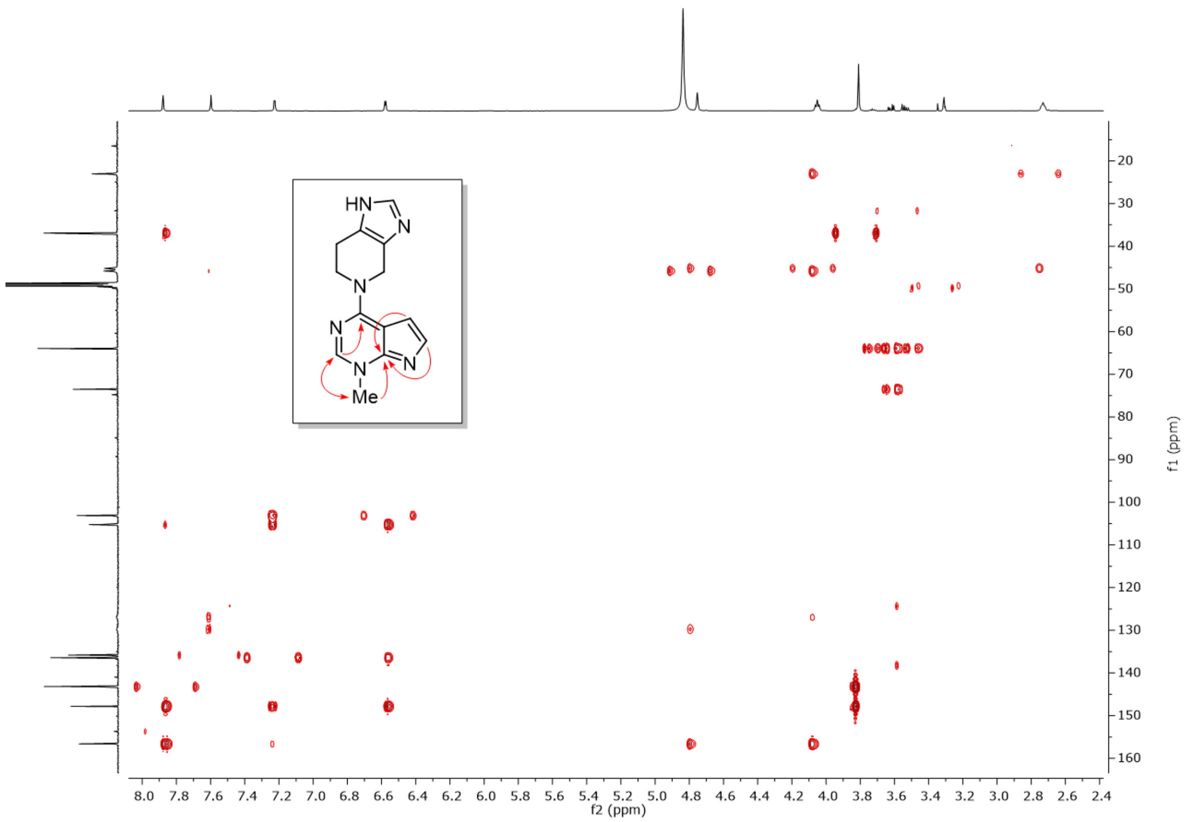


$^{1}H,^{13}C$ HMBC (600 MHz, $D_2O$ / $CD_3OD$) spectrum showing selected (H→C) correlations.

NMR spectra from enzymatic synthesis of 3-methyl-5-(7*H*-pyrrolo[2,3-*d*]pyrimidin-4-yl)-4,5,6,7-tetrahydro-3*H*-imidazo[4,5-*c*]pyridine (**14**)

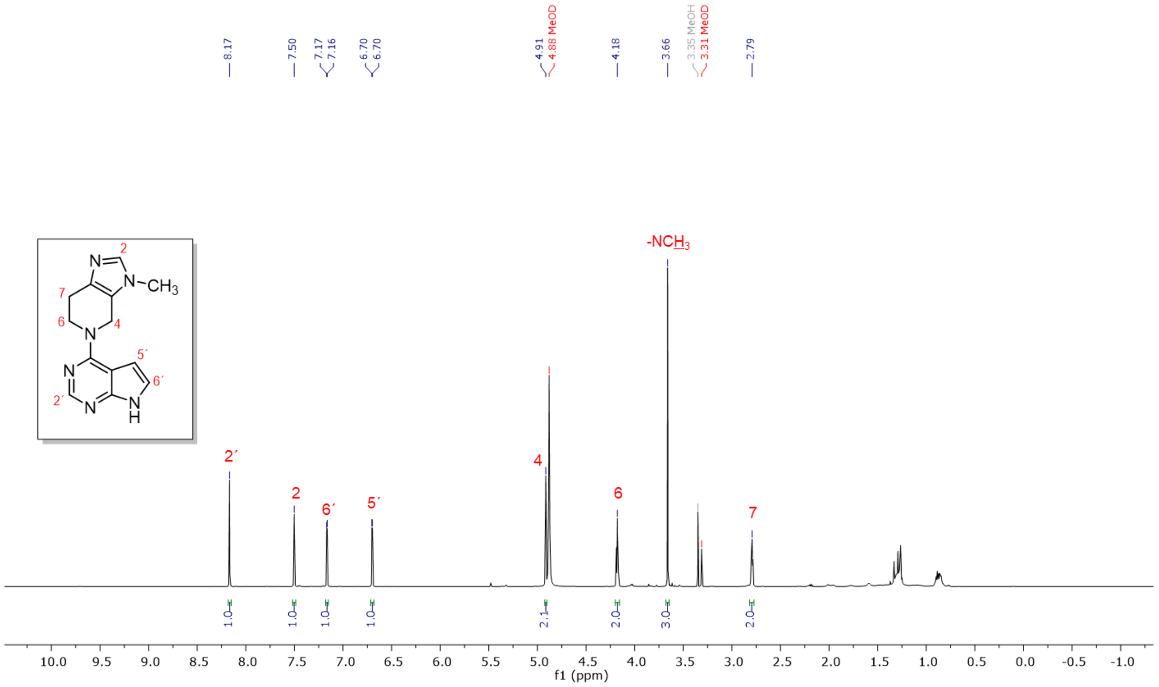


$^{1}$H-NMR (600 MHz, $CD_3OD$) spectrum.

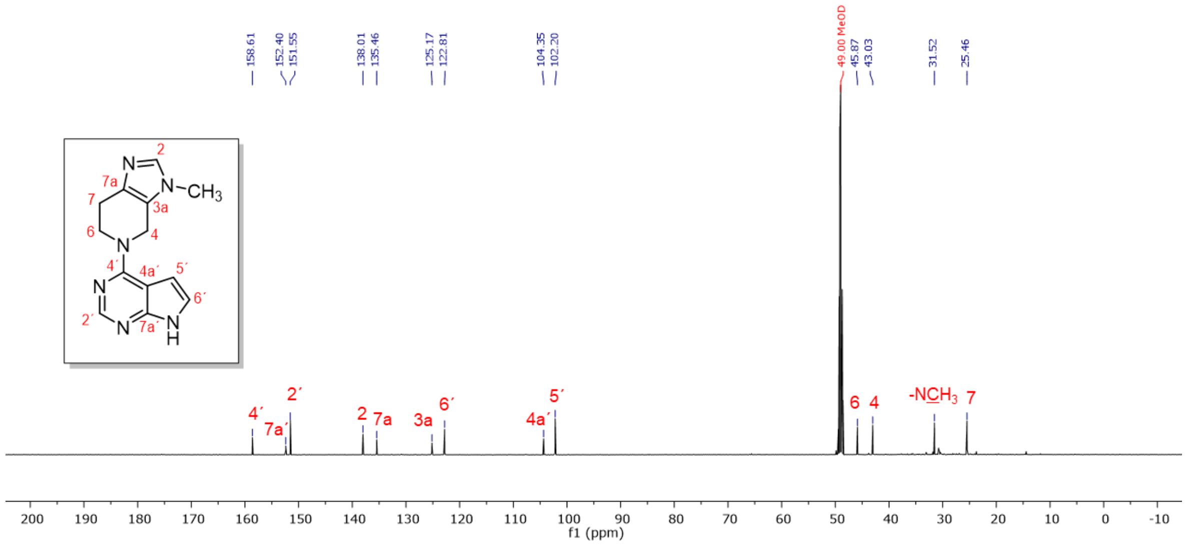


$^{13}$C-NMR (151 MHz, $CD_3OD$) spectrum.

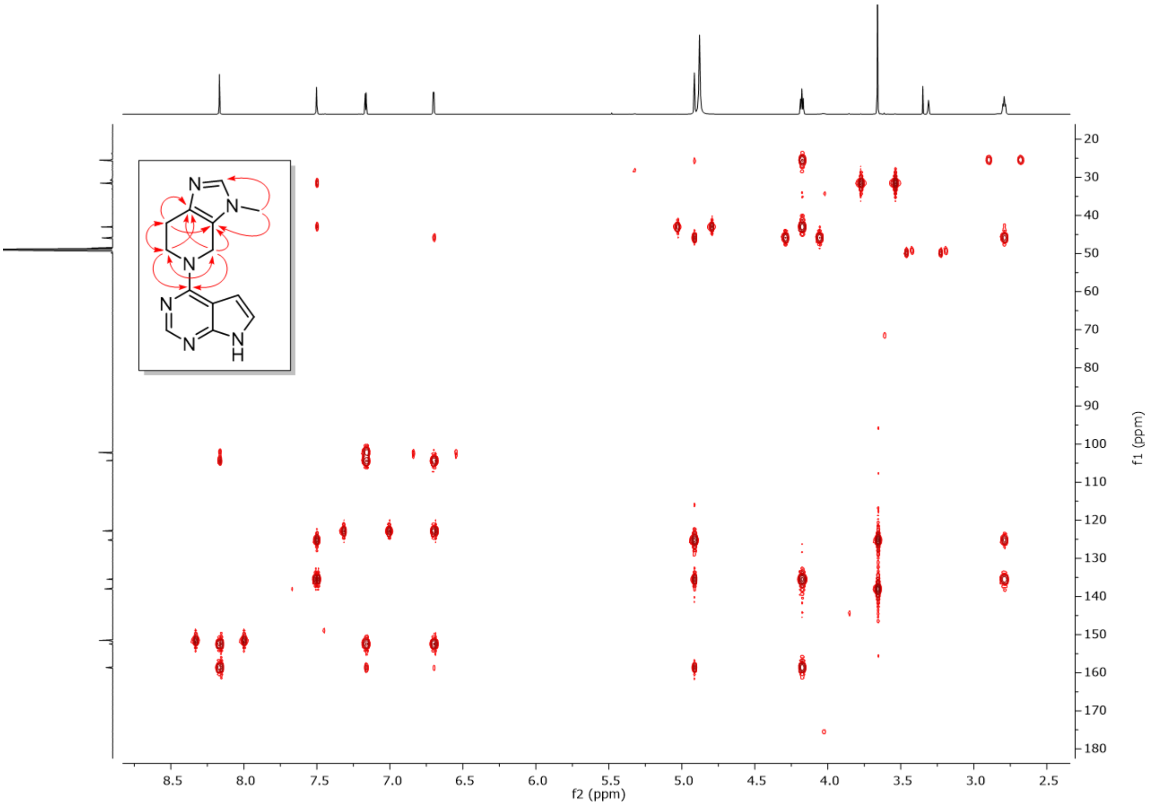


$^{1}H,^{13}C$ HMBC (600 MHz, $CD_3OD$) spectrum showing selected (H→C) correlations.

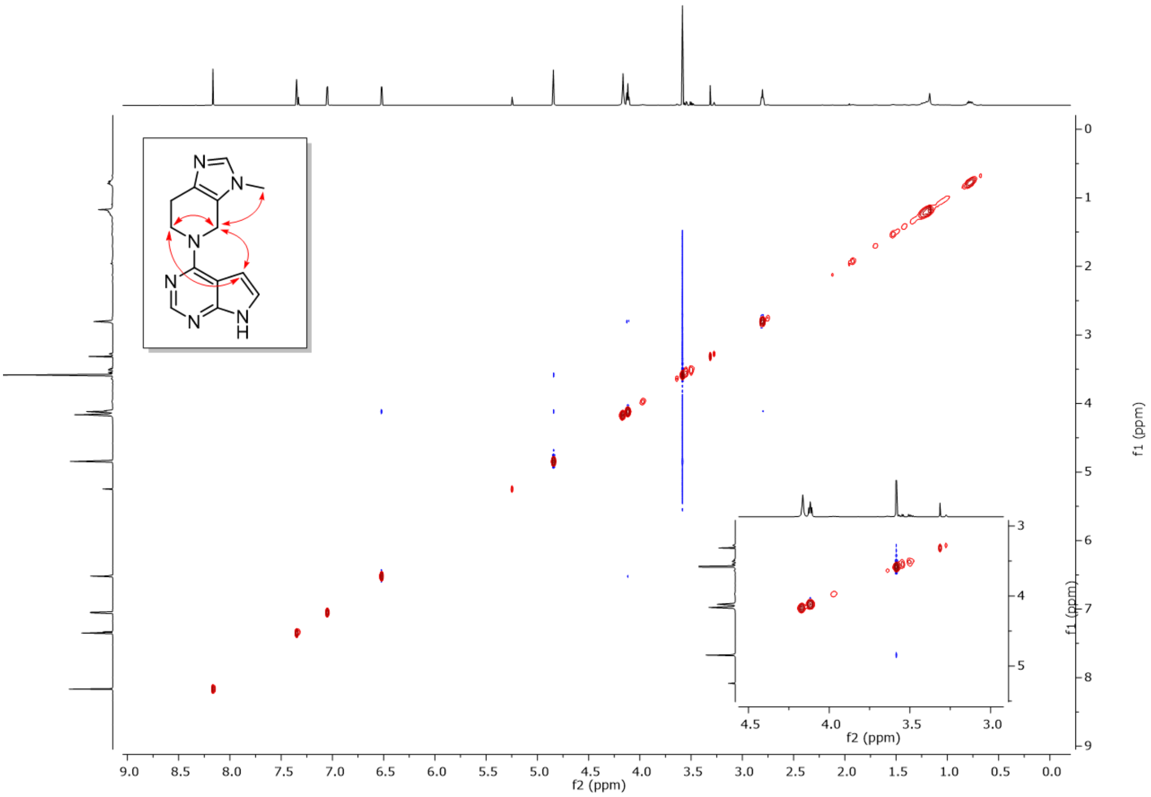


$^{1}H,^{1}H$ NOESY (600 MHz, $CD_3OD$) spectrum showing selected correlations.

NMR spectra from enzymatic synthesis of 1-methyl-5-(7*H*-pyrrolo[2,3-*d*]pyrimidin-4-yl)-4,5,6,7-tetrahydro-1*H*-imidazo[4,5-*c*]pyridine (**15**).

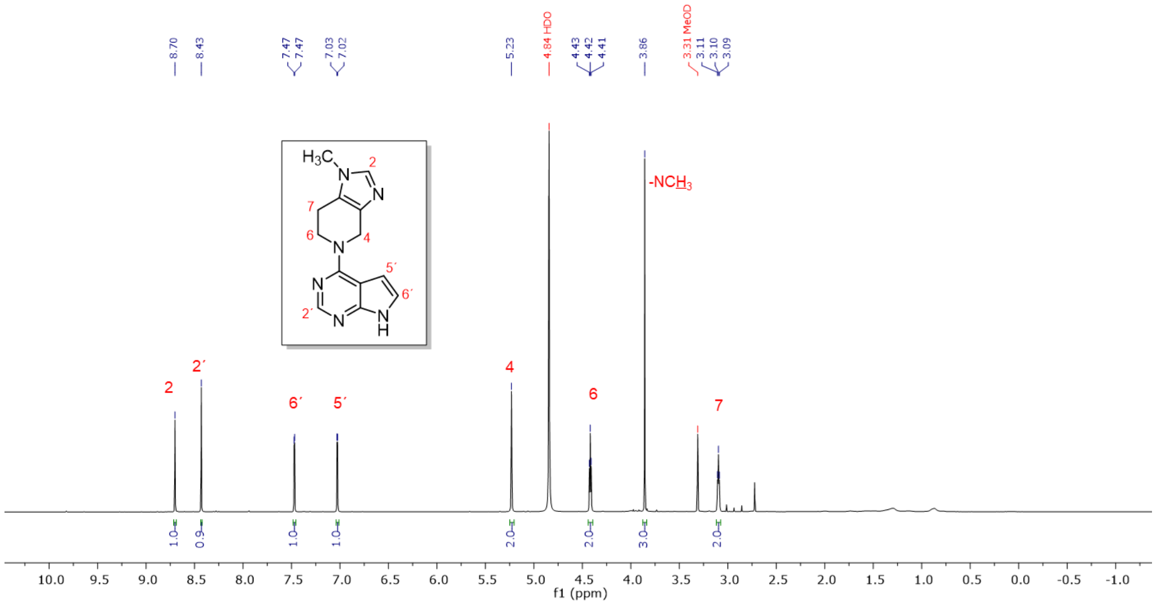


[1]H-NMR (600 MHz, $D_2O$ / $CD_3OD$) spectrum.

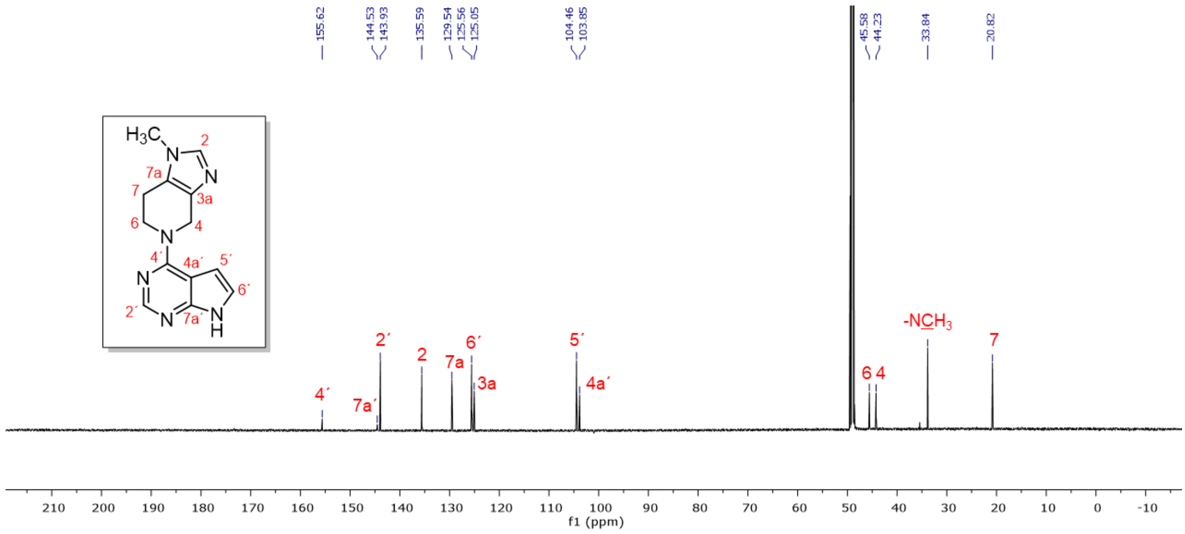


[13]C-NMR (151 MHz, $D_2O$ / $CD_3OD$) spectrum.

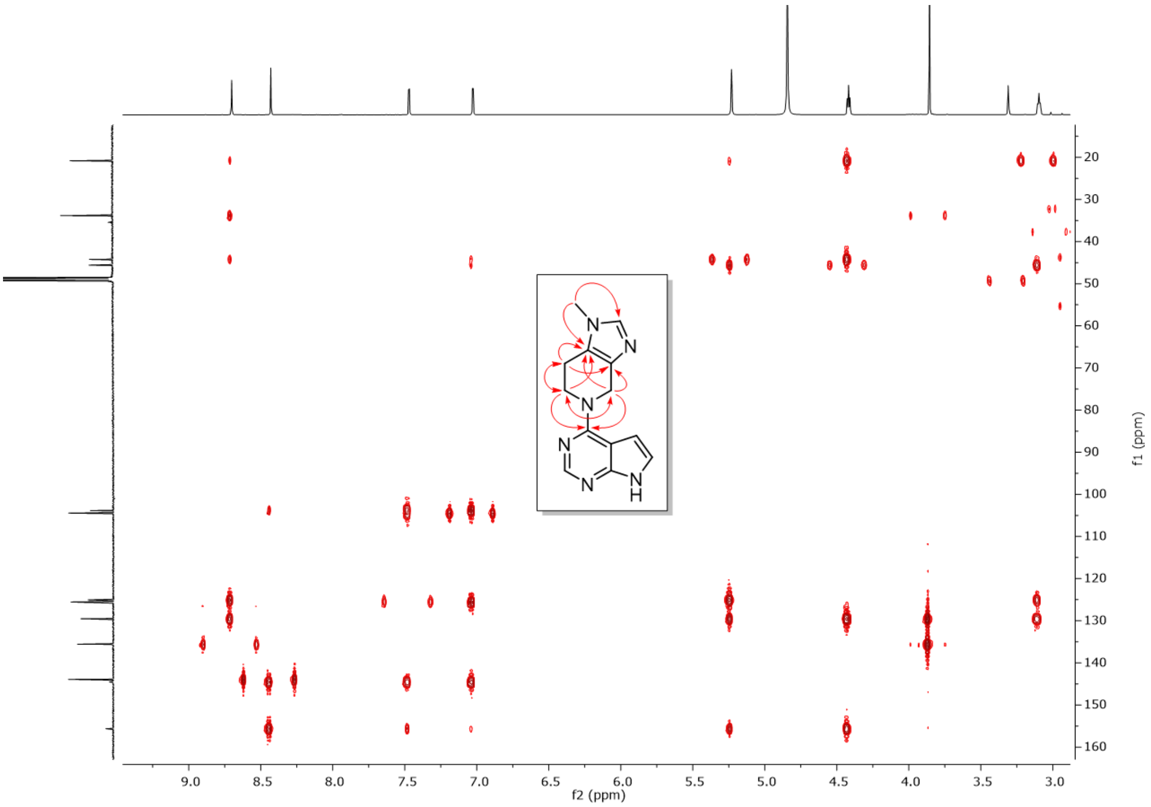

$^{1}H,^{13}C$ HMBC (600 MHz, $D_2O$ / $CD_3OD$) spectrum showing selected (H→C) correlations.

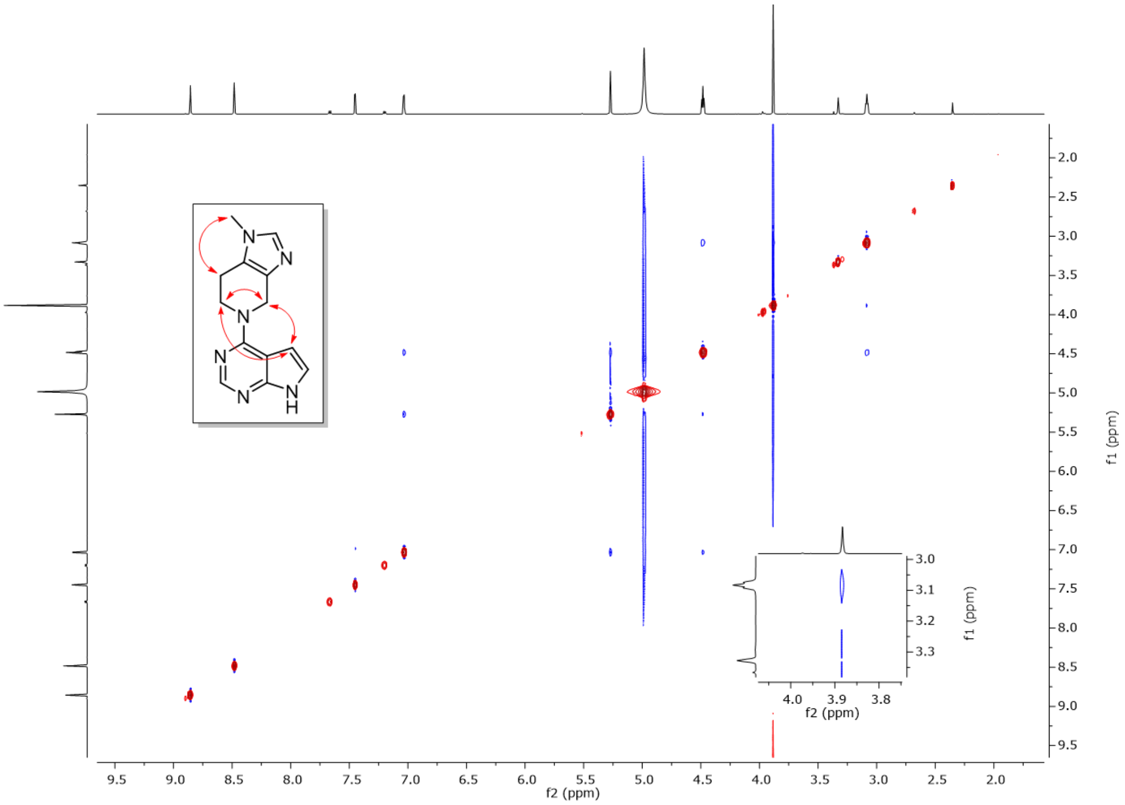

$^{1}H,^{1}H$ NOESY (600 MHz, $D_2O$ / $CD_3OD$) spectrum showing selected correlations.

NMR spectra from enzymatic synthesis of 3-ethyl-5-(7*H*-pyrrolo[2,3-*d*]pyrimidin-4-yl)-4,5,6,7-tetrahydro-3*H*-imidazo[4,5-*c*]pyridine (**24**).

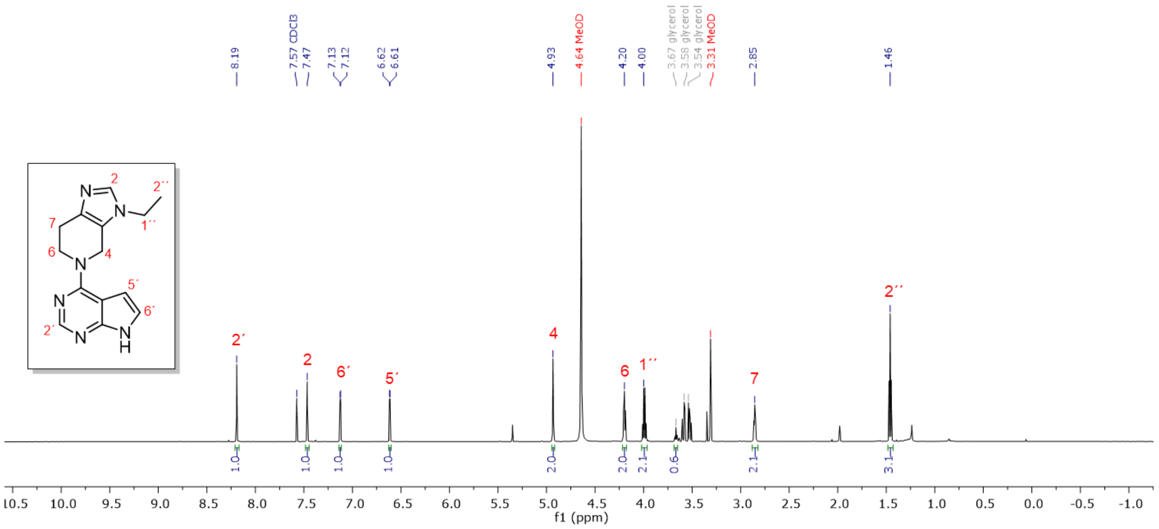


$^{1}$H-NMR (600 MHz, $CDCl_3$ / $CD_3OD$) spectrum.

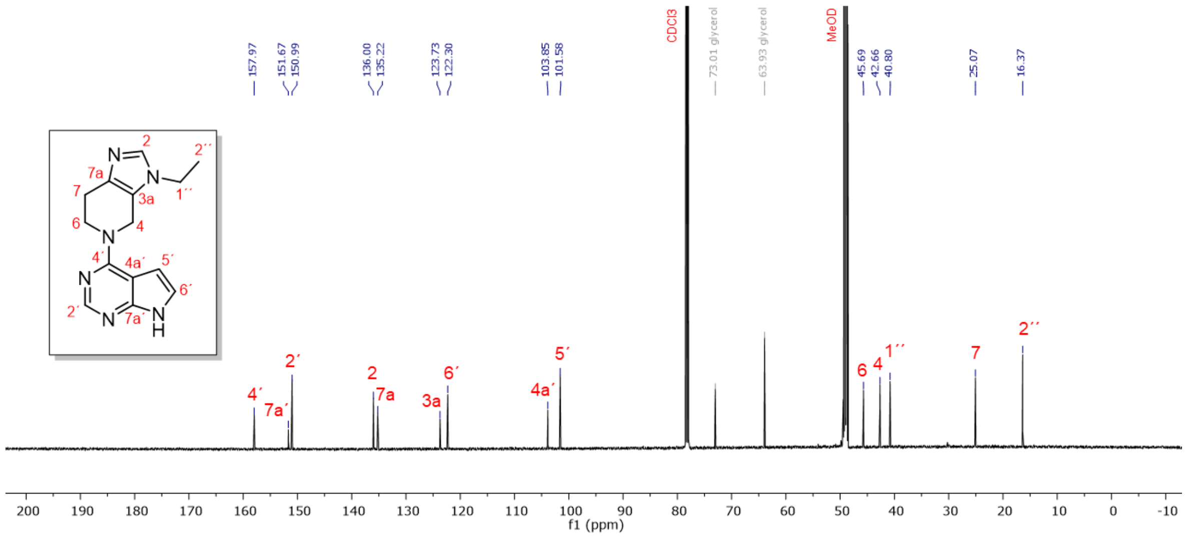


$^{13}$C-NMR (151 MHz, $CDCl_3$ / $CD_3OD$) spectrum.

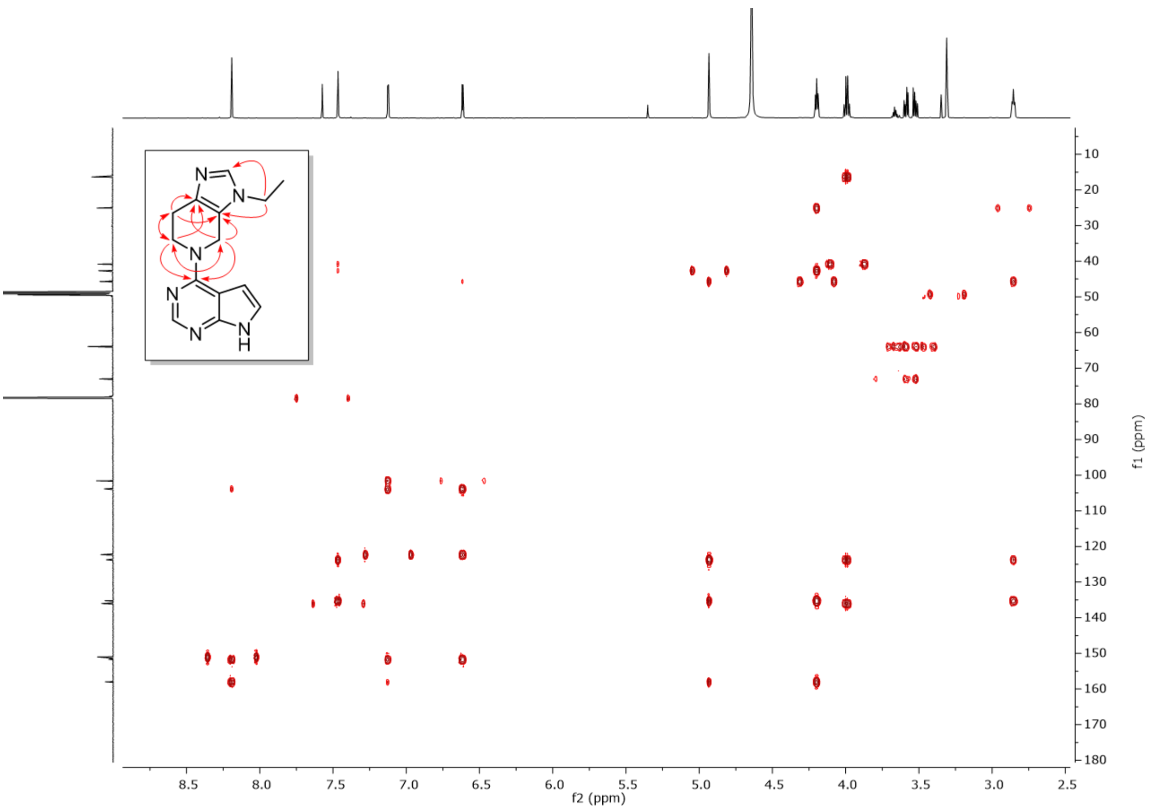


$^{1}H,^{13}C$ HMBC (600 MHz, $CDCl_3$ / $CD_3OD$) spectrum showing selected (H→C) correlations.

NMR spectra from enzymatic synthesis of 3-allyl-5-(7*H*-pyrrolo[2,3-*d*]pyrimidin-4-yl)-4,5,6,7-tetrahydro-3*H*-imidazo[4,5-*c*]pyridine (**25**).

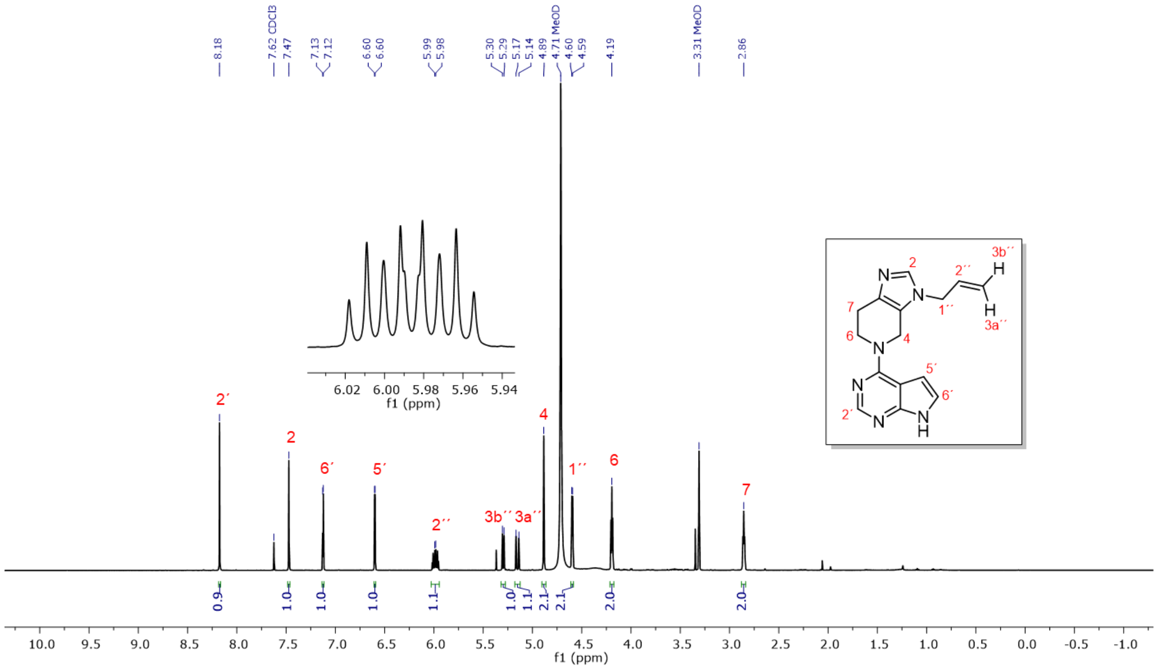

$^{1}$H-NMR (600 MHz, $CDCl_3$ / $CD_3OD$) spectrum.

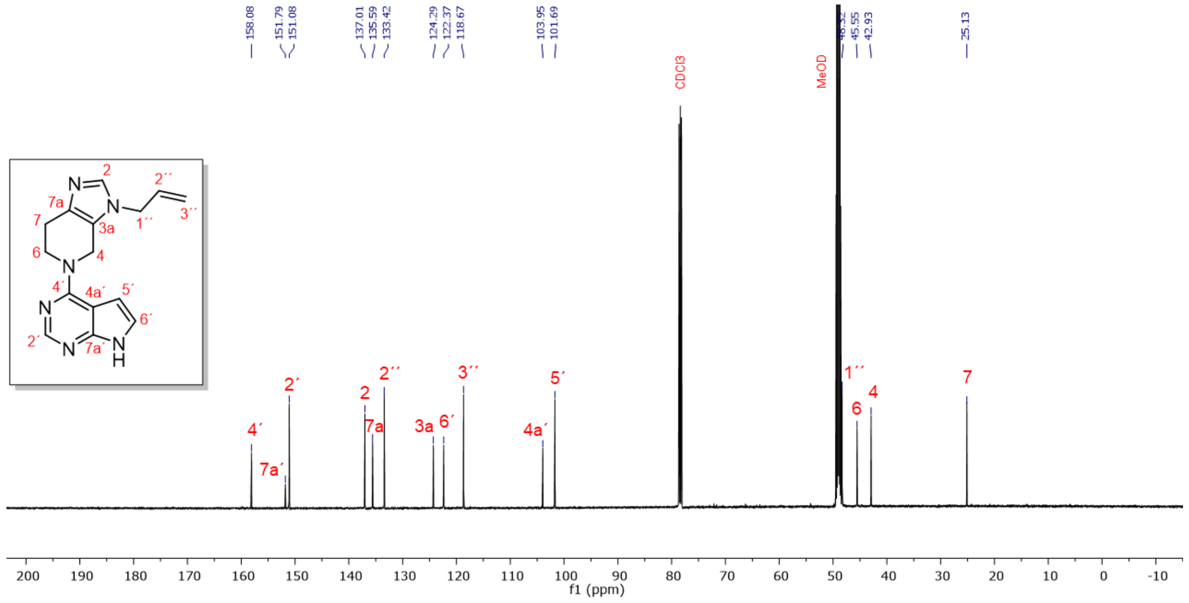

$^{13}$C-NMR (151 MHz, $CDCl_3$ / $CD_3OD$) spectrum.

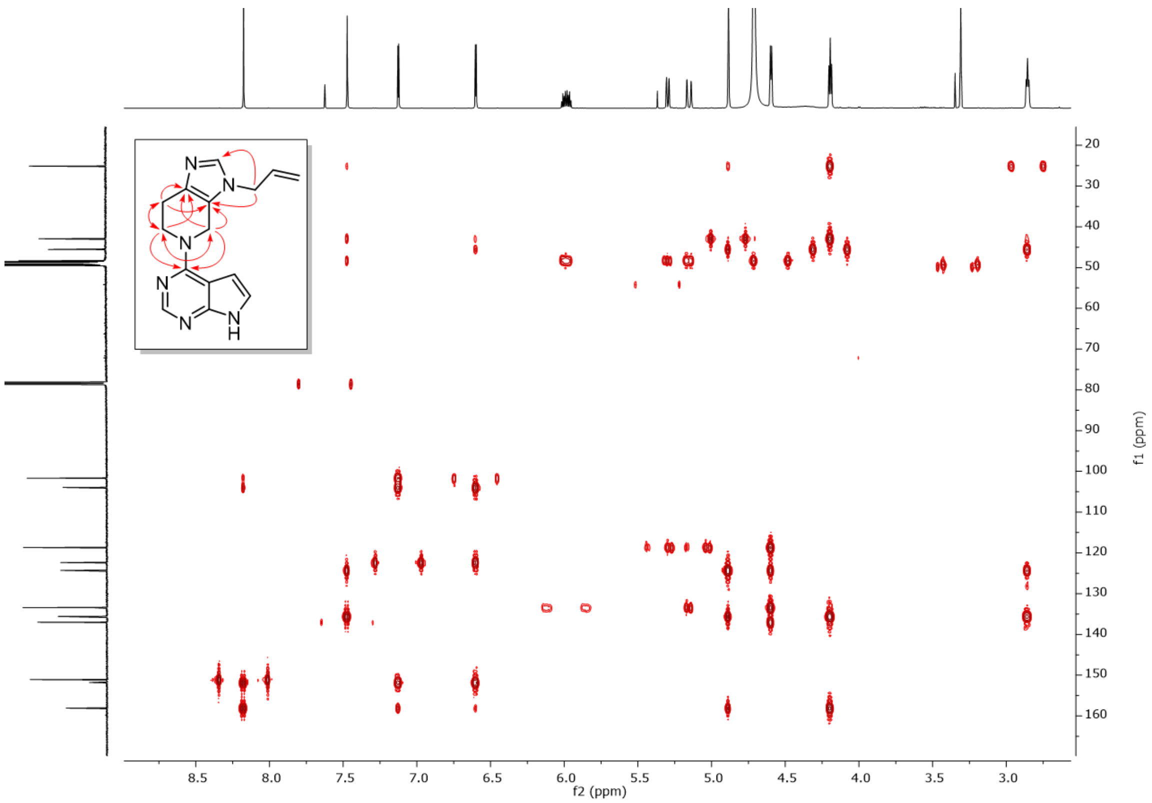


$^{1}H$,$^{13}C$ HMBC (600 MHz, $CDCl_3$ / $CD_3OD$) spectrum showing selected (H→C) correlations.

NMR spectra from chemical synthesis of 4-chloro-7-methyl-7*H*-pyrrolo[2,3-*d*]pyrimidine.

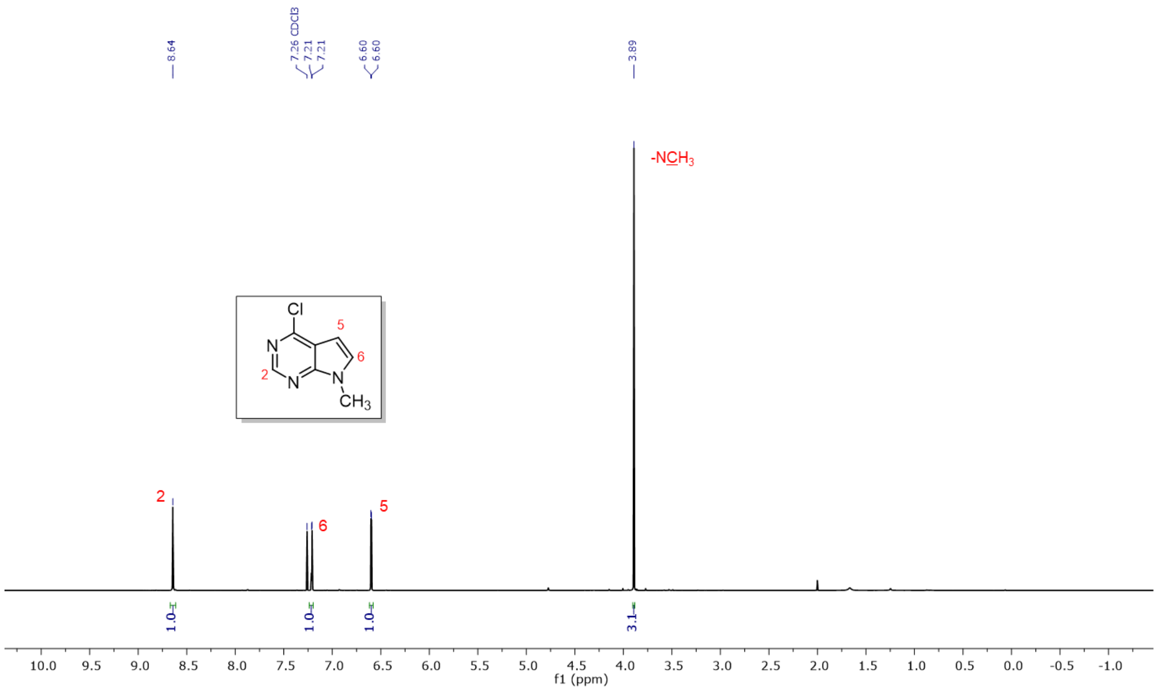


$^{1}$H-NMR (600 MHz, $CDCl_3$) spectrum.

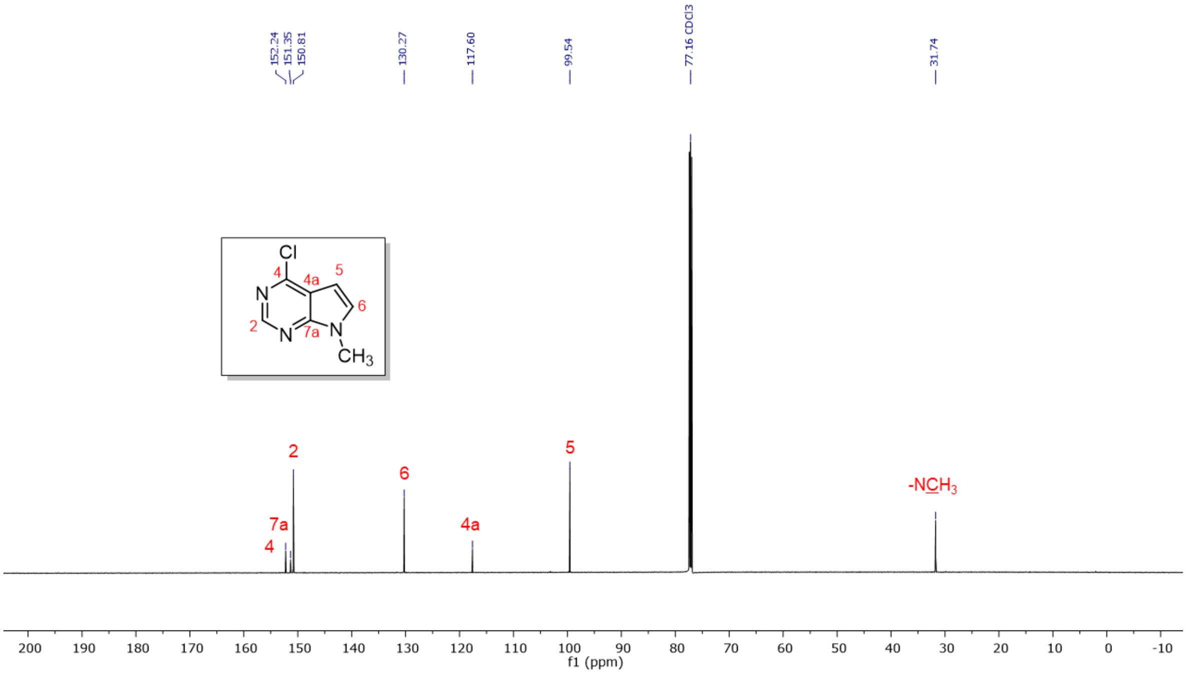


$^{13}$C-NMR (151 MHz, $CDCl_3$) spectrum.

NMR spectra from chemical synthesis of 5-(7-methyl-7*H*-pyrrolo[2,3-*d*]pyrimidin-4-yl)-4,5,6,7-tetrahydro-3*H*-imidazo[4,5-*c*]pyridine.

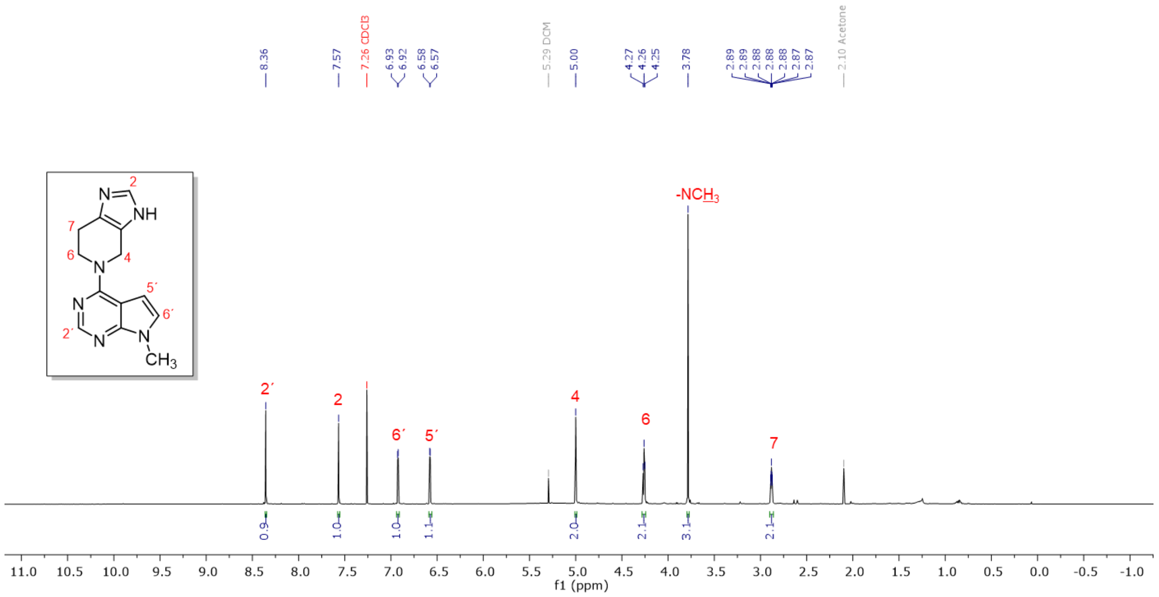


$^{1}$H-NMR (600 MHz, $CDCl_3$) spectrum.

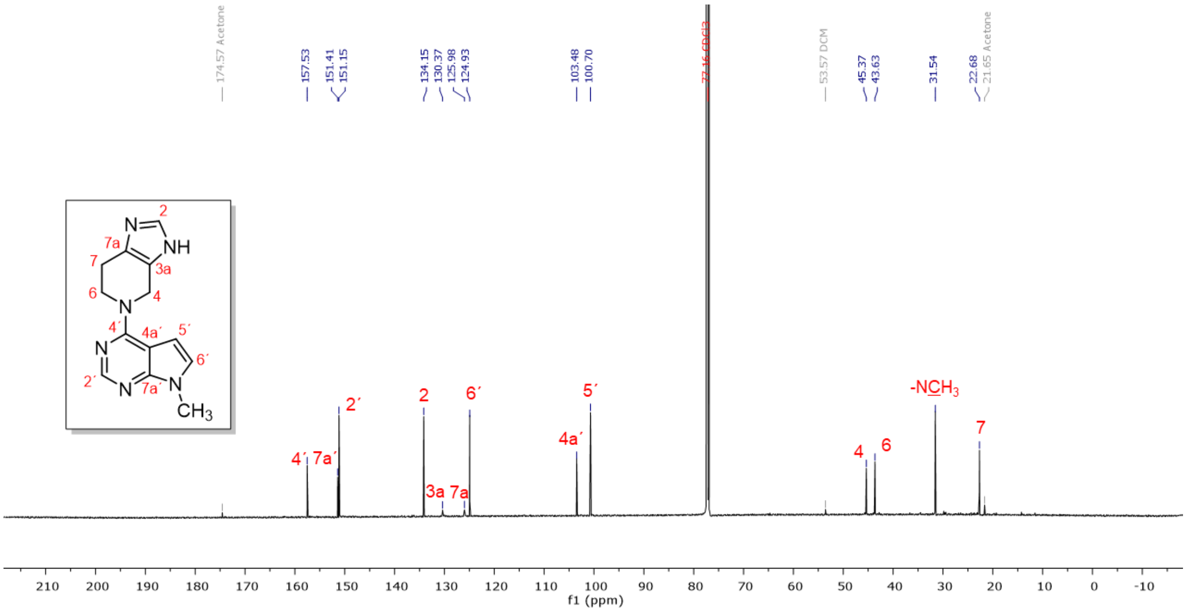


$^{13}$C-NMR (151 MHz, $CDCl_3$) spectrum.

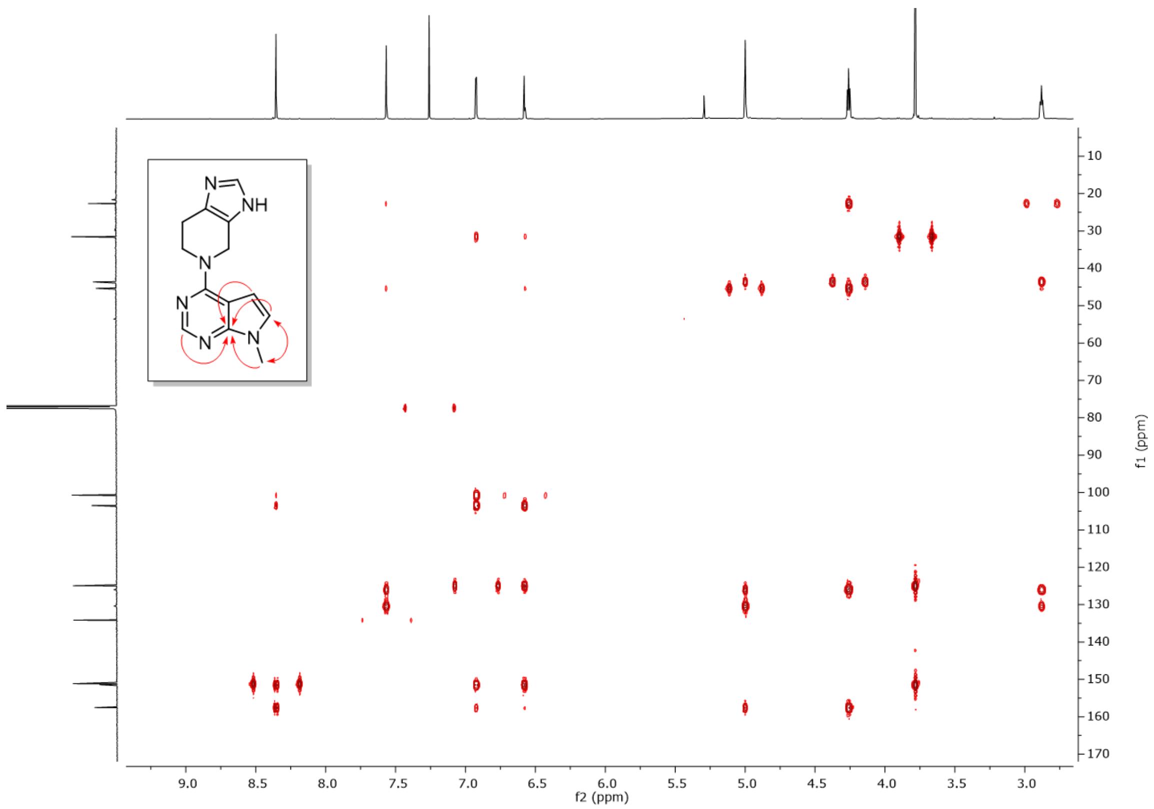


[1]H,[13]C HMBC (600 MHz, $CDCl_3$) spectrum showing selected (H→C) correlations.

NMR spectra from chemical synthesis of 5-bromo-1-ethyl-1*H*-benzo[*d*]imidazole and 6-bromo-1-ethyl-1*H*-benzo[*d*]imidazole as mixture of regioisomers.

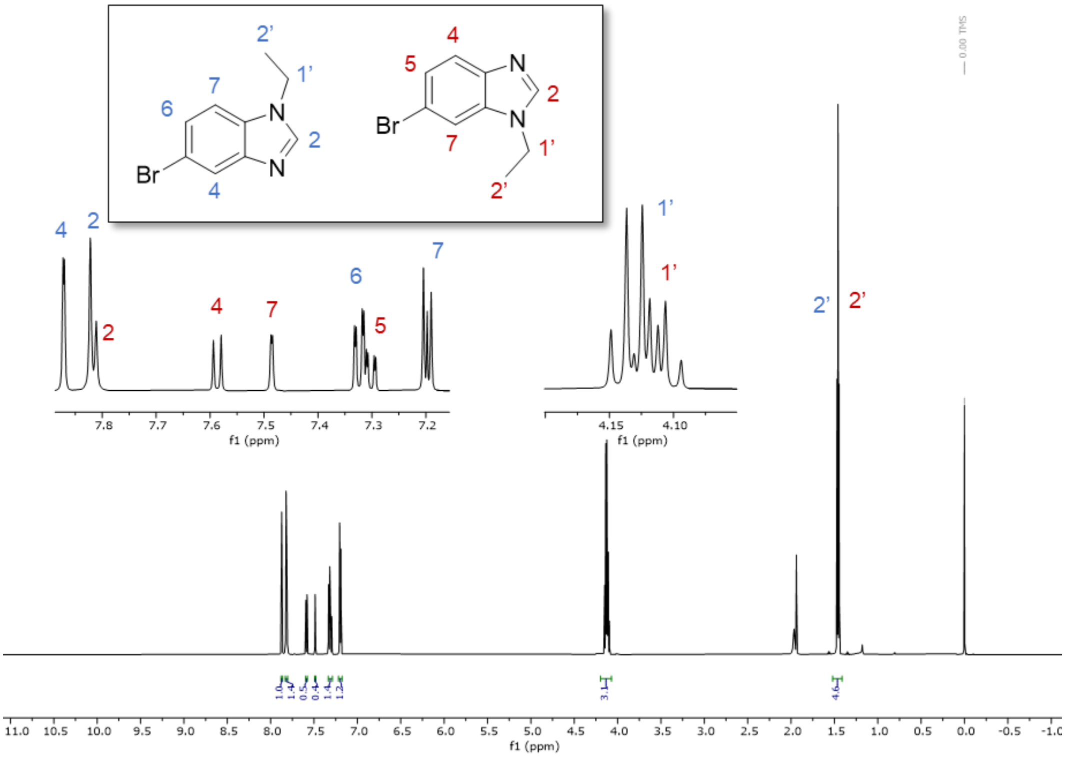


[1]H-NMR (600 MHz, $CDCl_3$) spectrum.

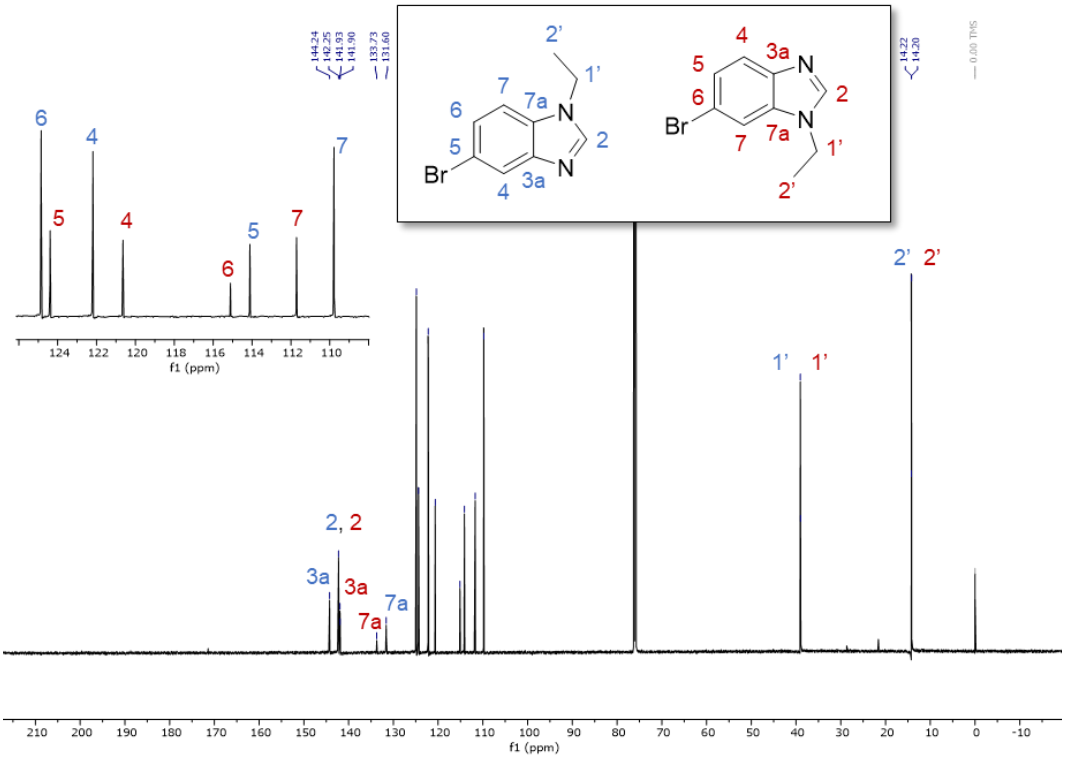


[13]C-NMR (151 MHz, $CDCl_3$) spectrum.

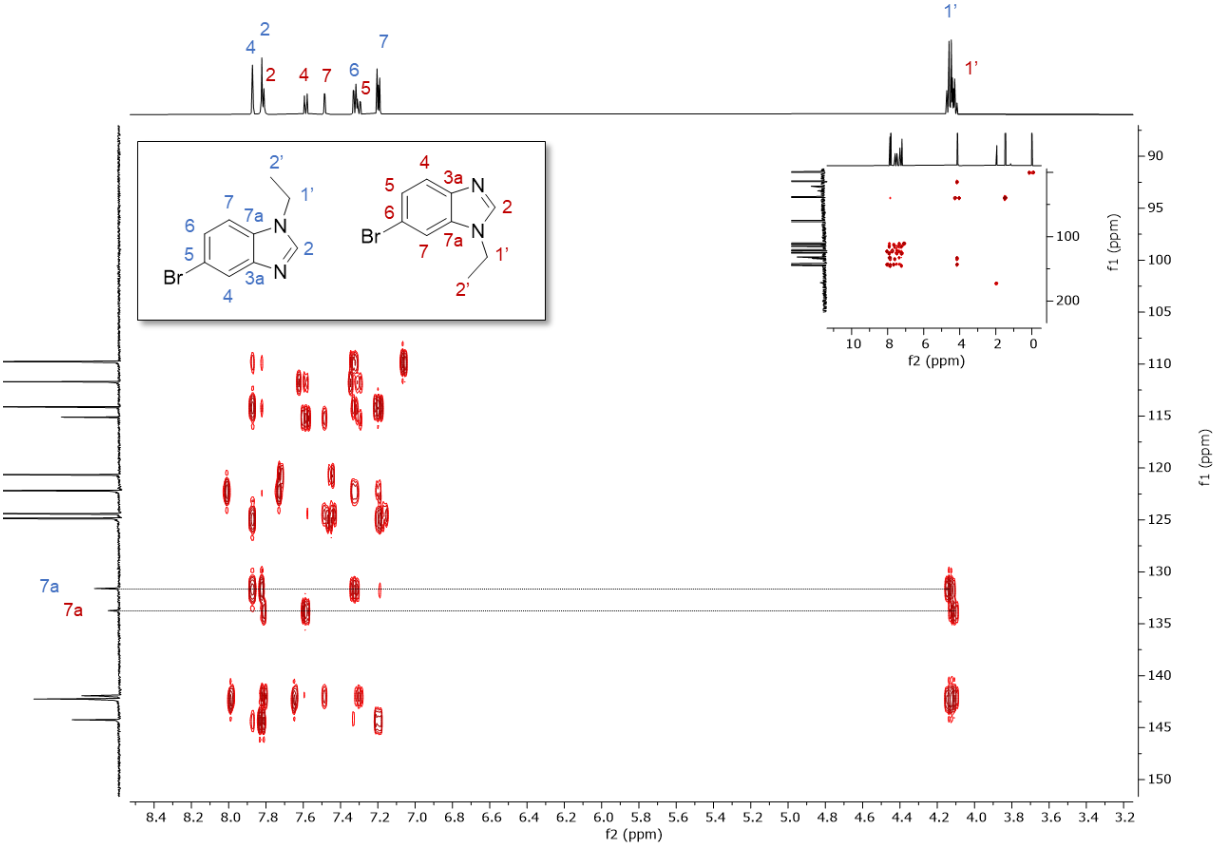


$^1H,^{13}C$ HMBC (600 MHz, $CDCl_3$).

NMR spectra from chemical synthesis of 5-bromo-1-(cyclopropylmethyl)-1*H*-benzo[*d*]imidazole and 6-bromo-1-(cyclopropylmethyl)-1*H*-benzo[*d*]imidazole as mixture of regioisomers.

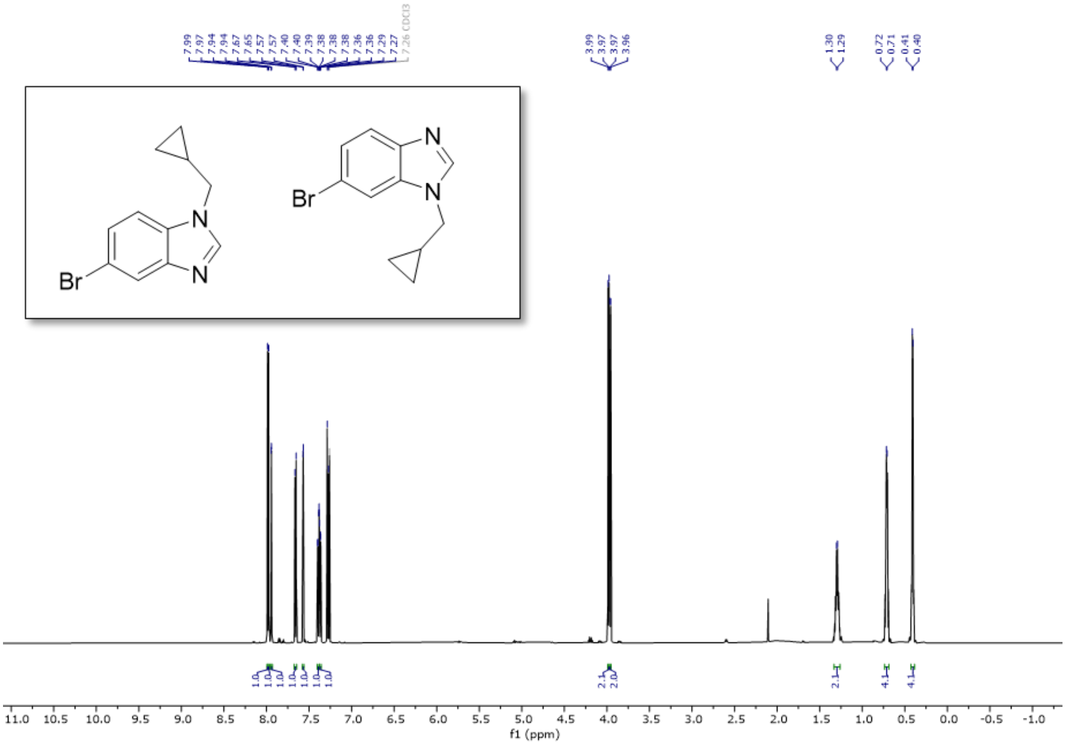


[1]H-NMR (600 MHz, $CDCl_3$) spectrum

.

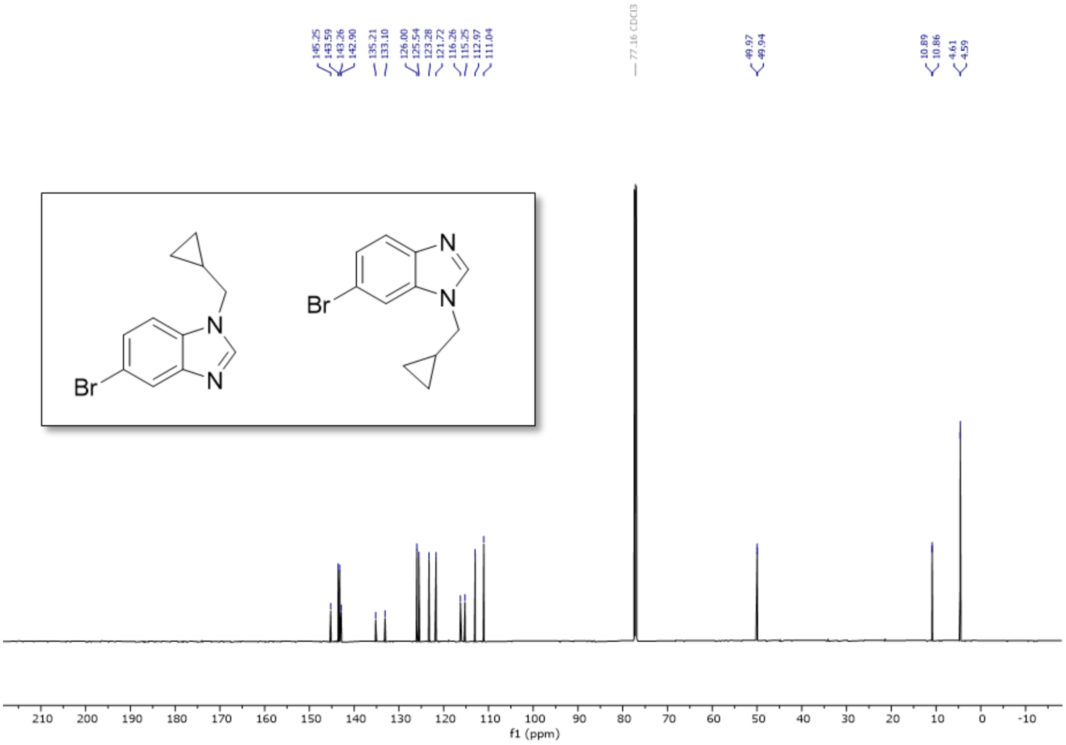


[13]C-NMR (151 MHz, $CDCl_3$) spectrum.

NMR spectra from chemical synthesis of 5-bromo-1-(cyclopropylmethyl)-1*H*-benzo[*d*]imidazole.

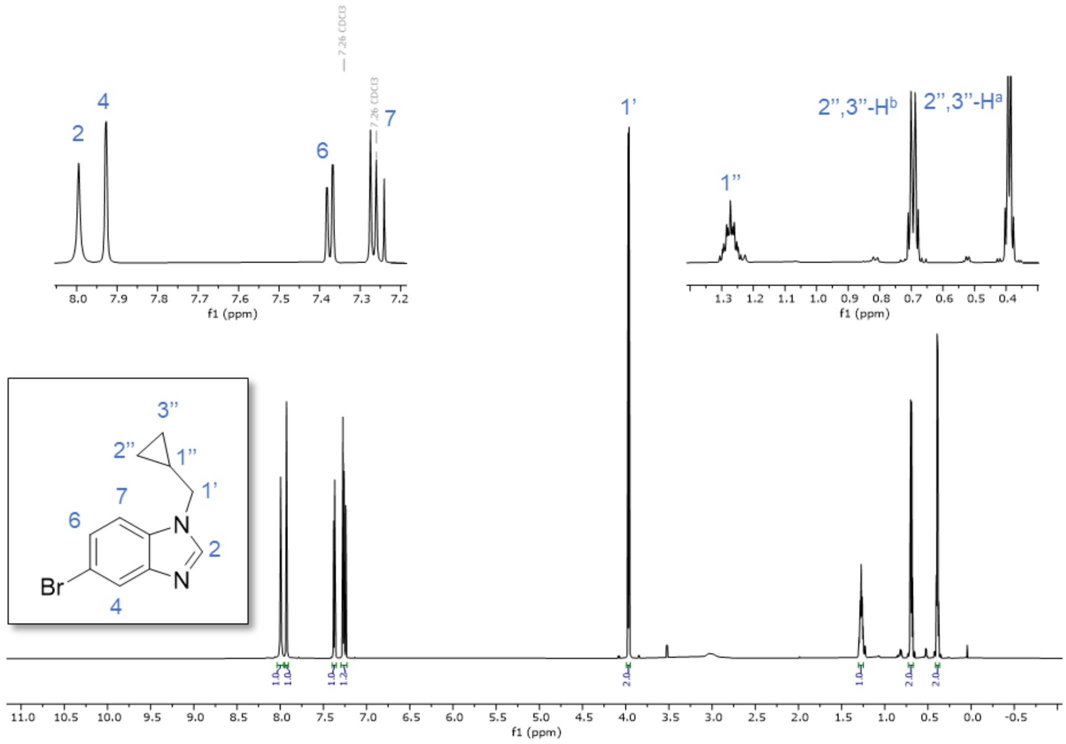


[1]H-NMR (600 MHz, $CDCl_3$) spectrum.

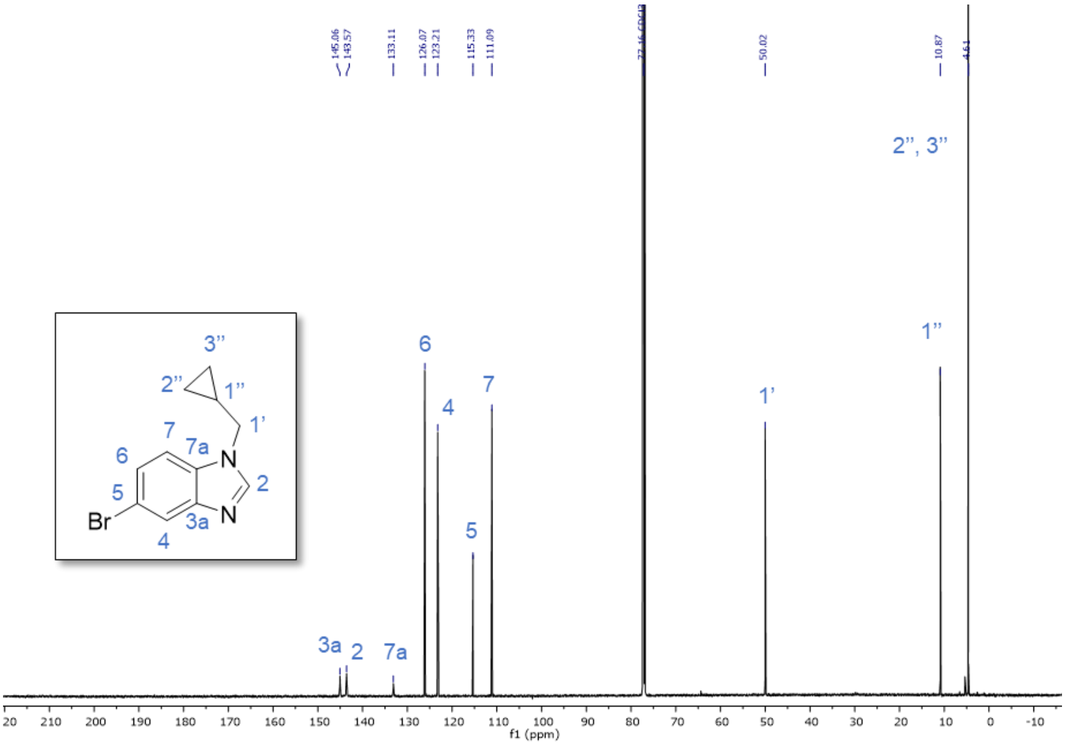


[13]C-NMR (151 MHz, $CDCl_3$) spectrum.

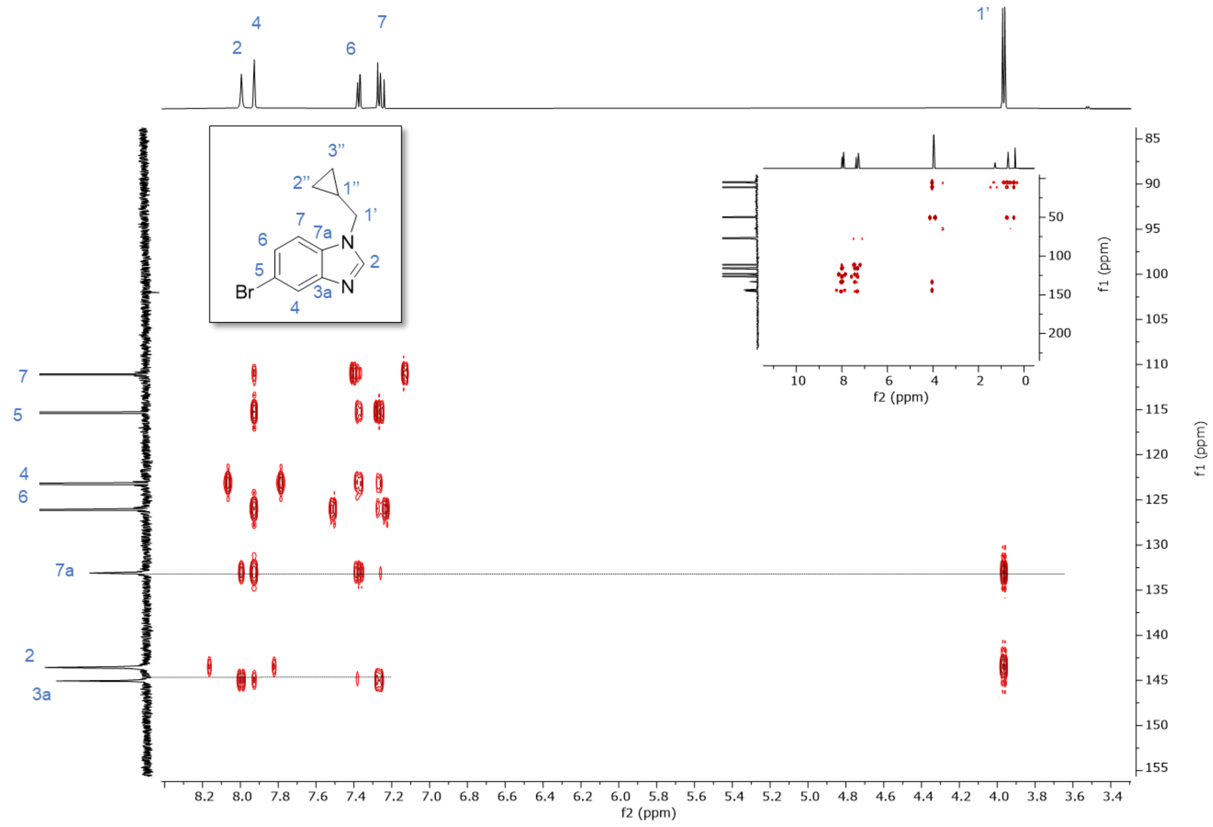


[1]H,[13]C HMBC (600 MHz, $CDCl_3$) spectrum.

NMR spectra from chemical synthesis of 6-bromo-1-(cyclopropylmethyl)-1*H*-benzo[*d*]imidazole (**17**).

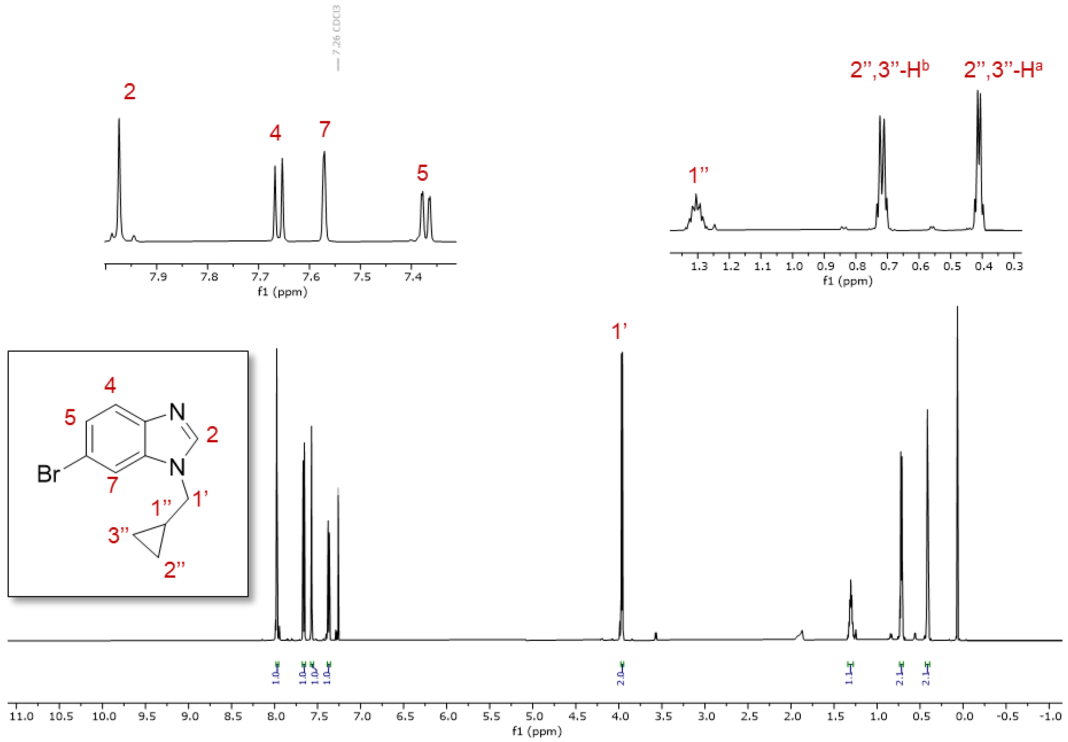


$^{1}$H-NMR (600 MHz, $CDCl_3$) spectrum.

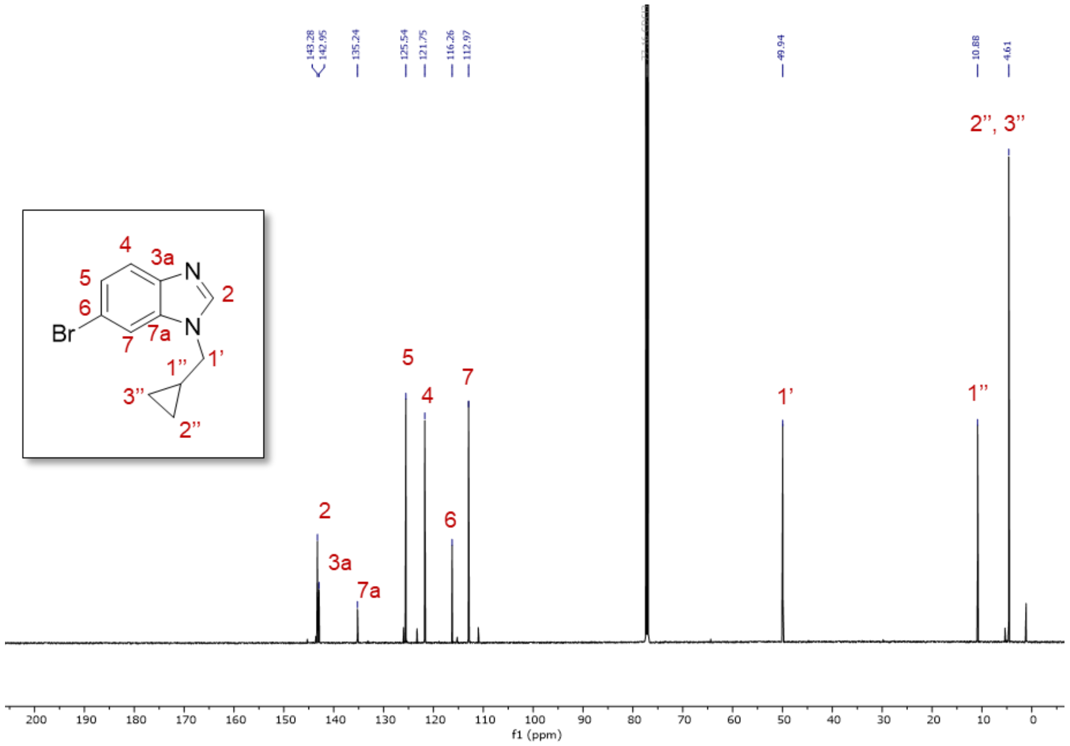


$^{13}$C-NMR (151 MHz, $CDCl_3$) spectrum.

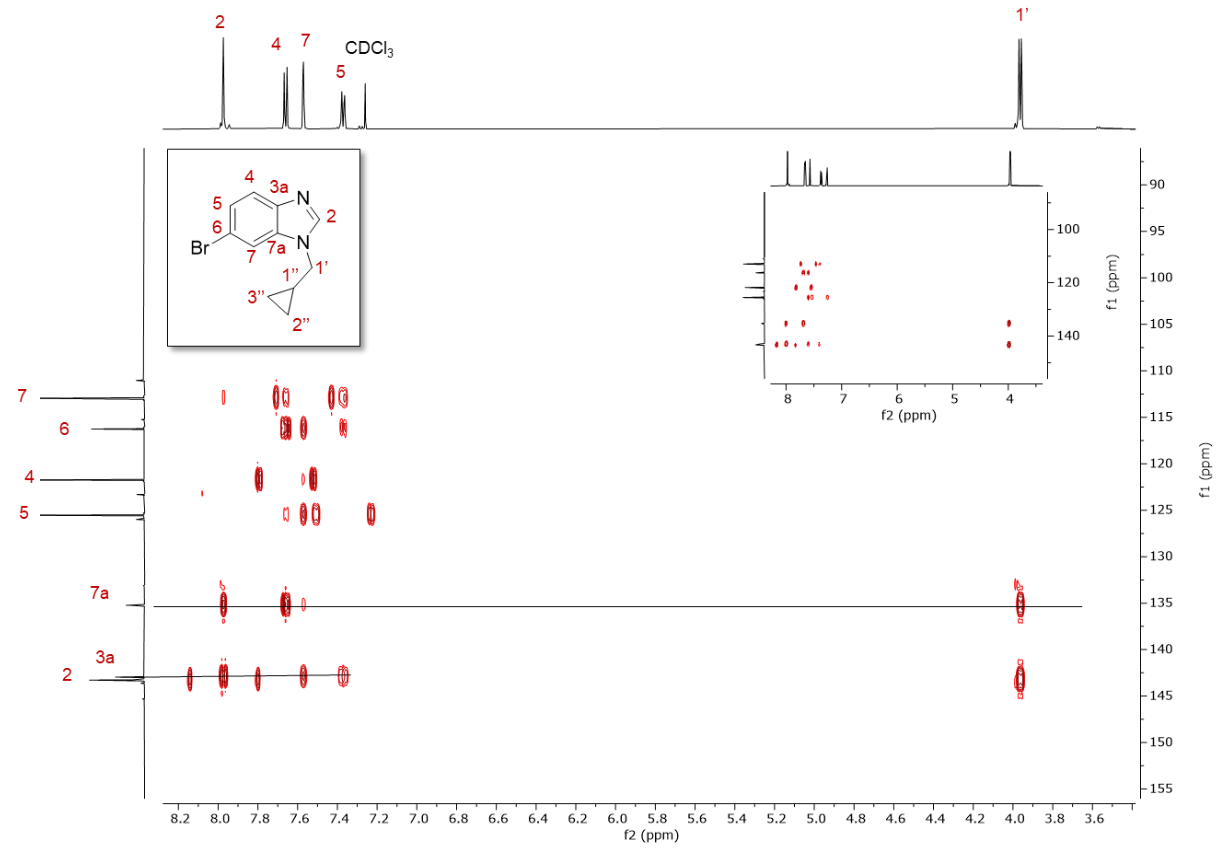


$^{1}H,^{13}C$ HMBC (600 MHz, $CDCl_3$) spectrum.

NMR spectra from chemical synthesis of 5-bromo-1-(cyclobutylmethyl)-1*H*-benzo[*d*]imidazole and 6-bromo-1-(cyclobutylmethyl)-1*H*-benzo[*d*]imidazole as mixture of regioisomers.

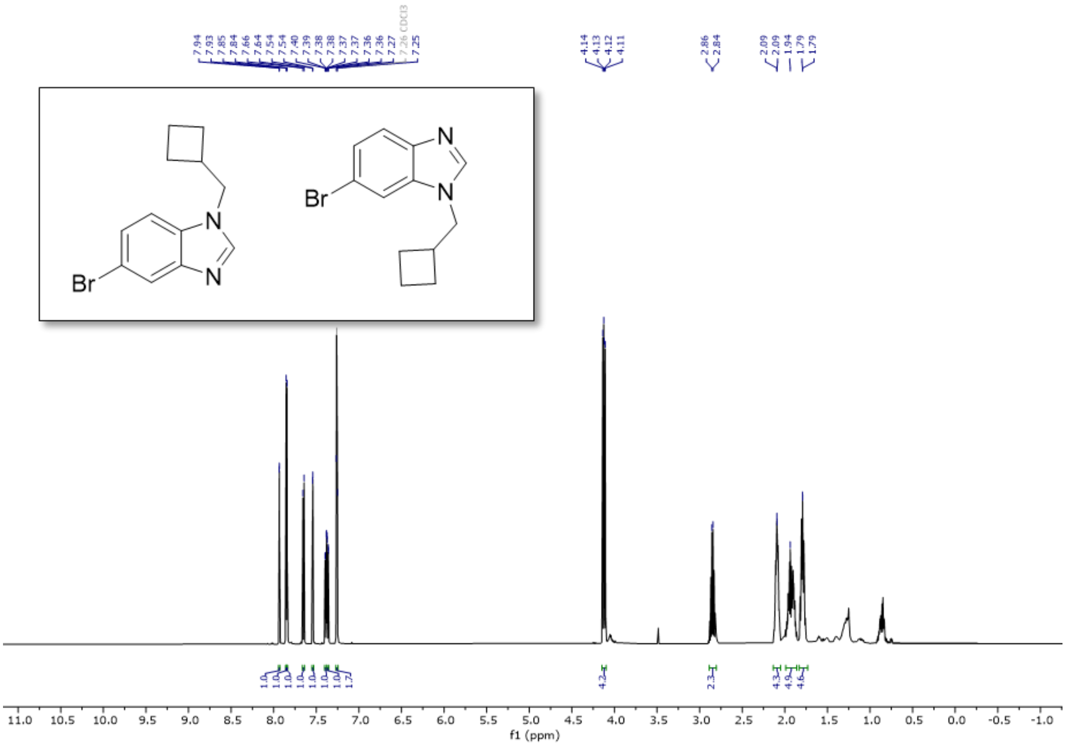


$^{1}$H-NMR (600 MHz, $CDCl_3$) spectrum.

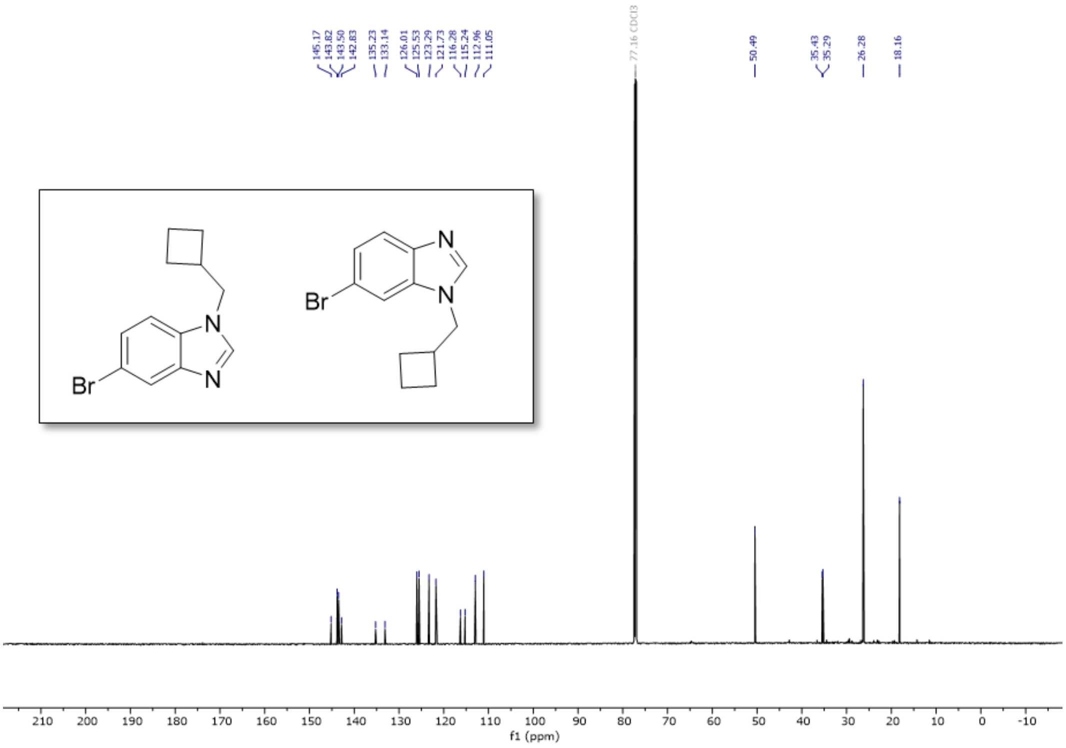


$^{13}$C-NMR (151 MHz, $CDCl_3$) spectrum.

NMR spectra from chemical synthesis of 5-bromo-1-(cyclobutylmethyl)-1*H*-benzo[*d*]imidazole.

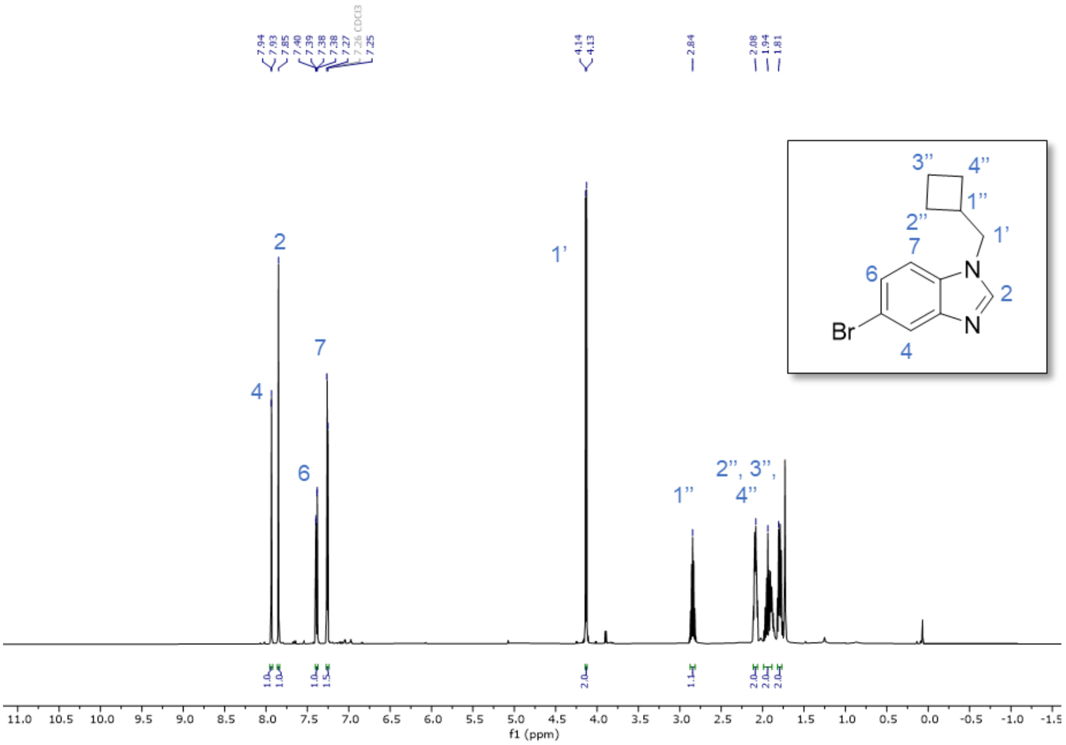

[1]H-NMR (600 MHz, $CDCl_3$) spectrum.

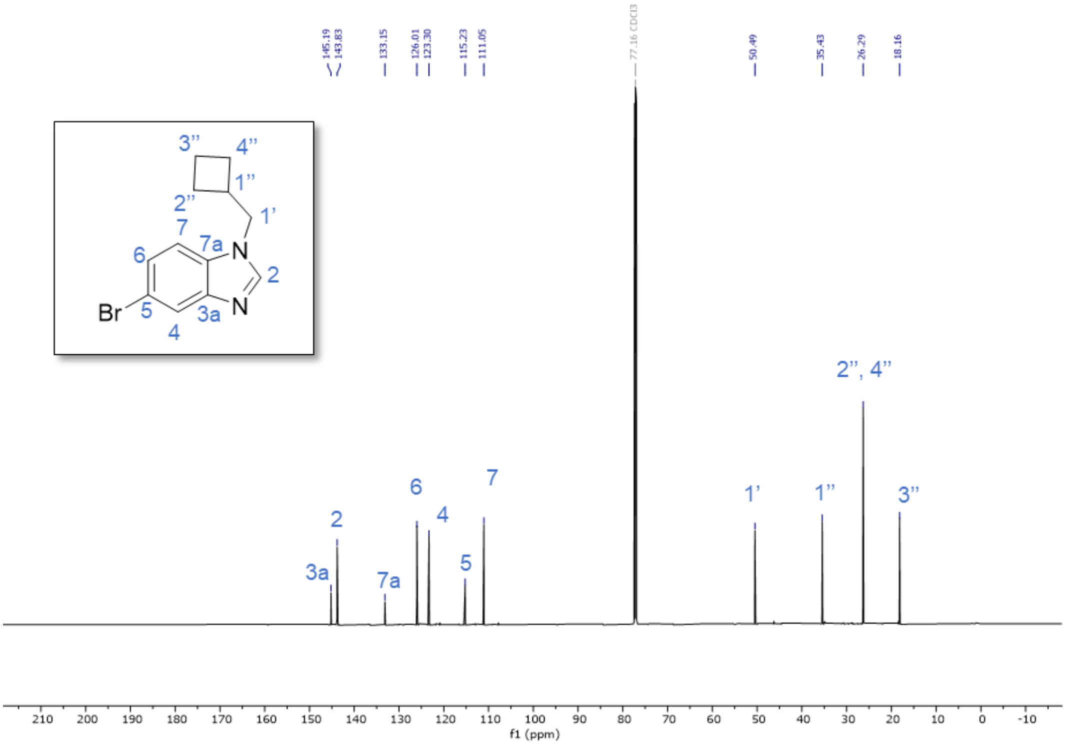

[13]C-NMR (151 MHz, $CDCl_3$) spectrum.

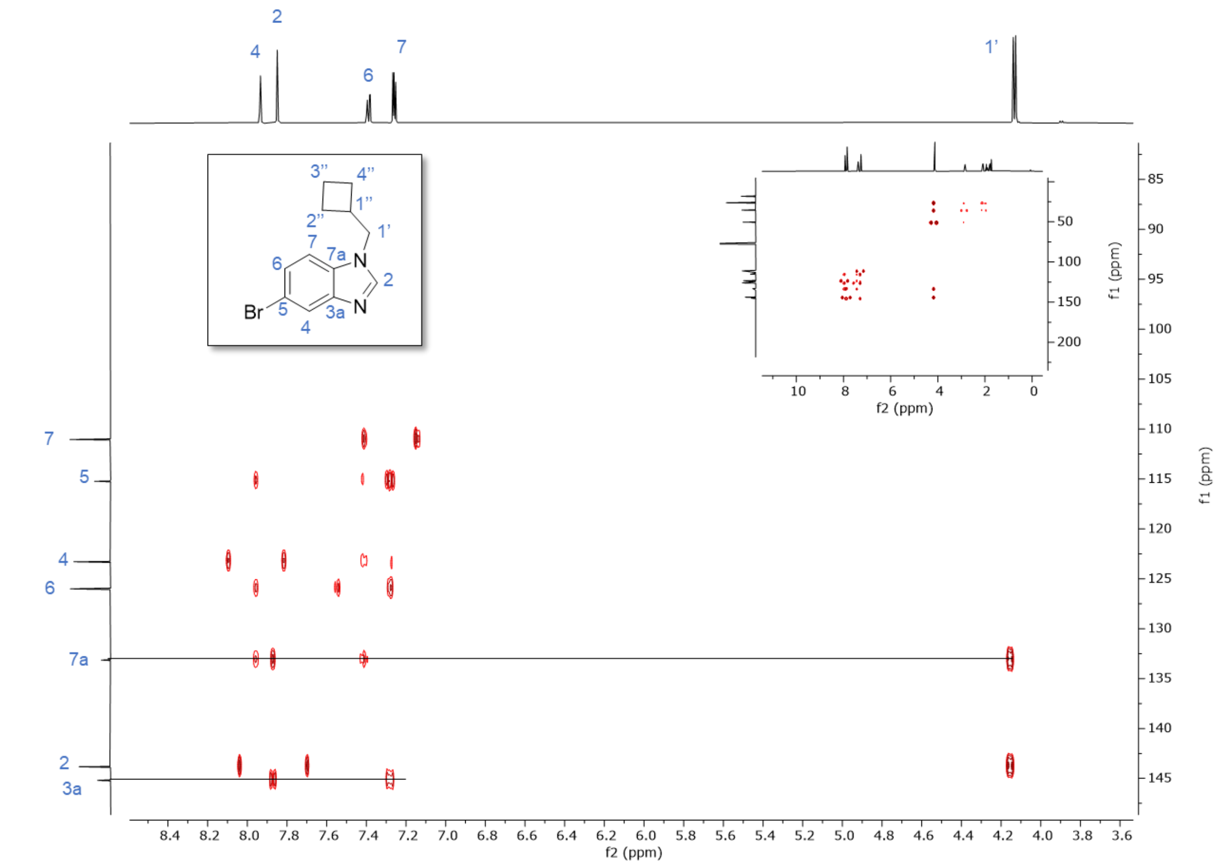


$^{1}H,^{13}C$ HMBC (600 MHz, $CDCl_3$) spectrum.

<u>NMR spectra from chemical synthesis of 5-bromo-1-allyl-1*H*-benzo[*d*]imidazole and 6-bromo-1-allyl-1*H*-benzo[*d*]imidazole as mixture of regioisomers.</u>

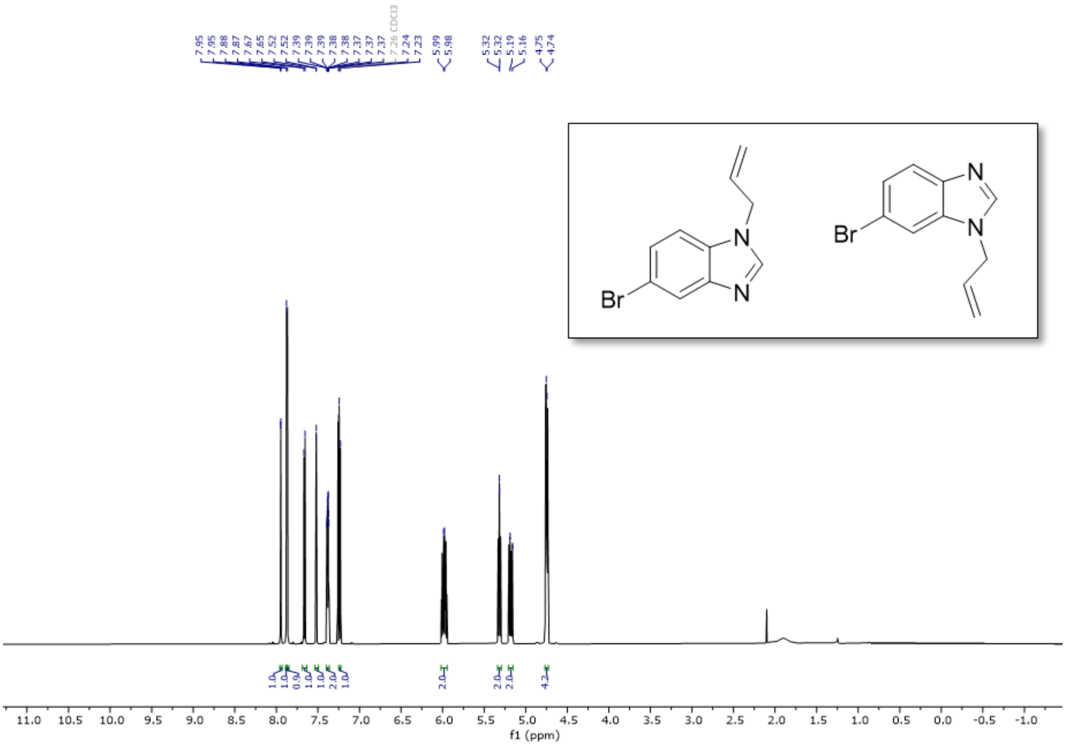


[1]H-NMR (600 MHz, $CDCl_3$) spectrum.

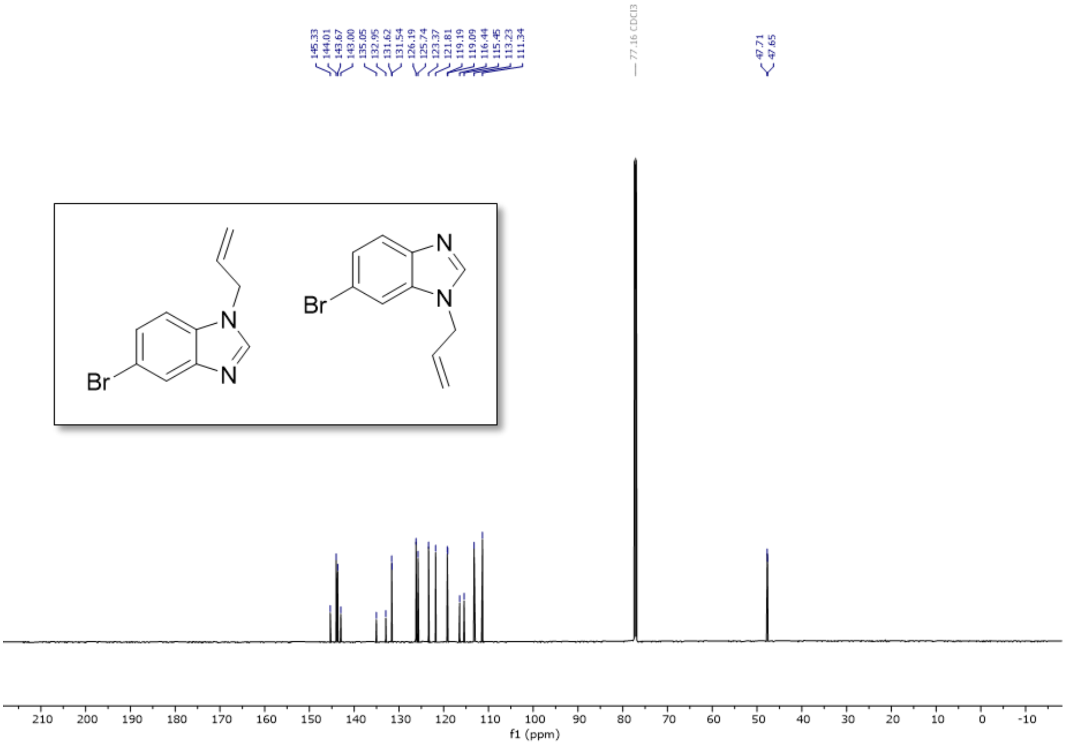


[13]C-NMR (151 MHz, $CDCl_3$) spectrum.

NMR spectra from enzymatic synthesis of 6-bromo-1-allyl-1*H*-benzo[*d*]imidazole (**19**).

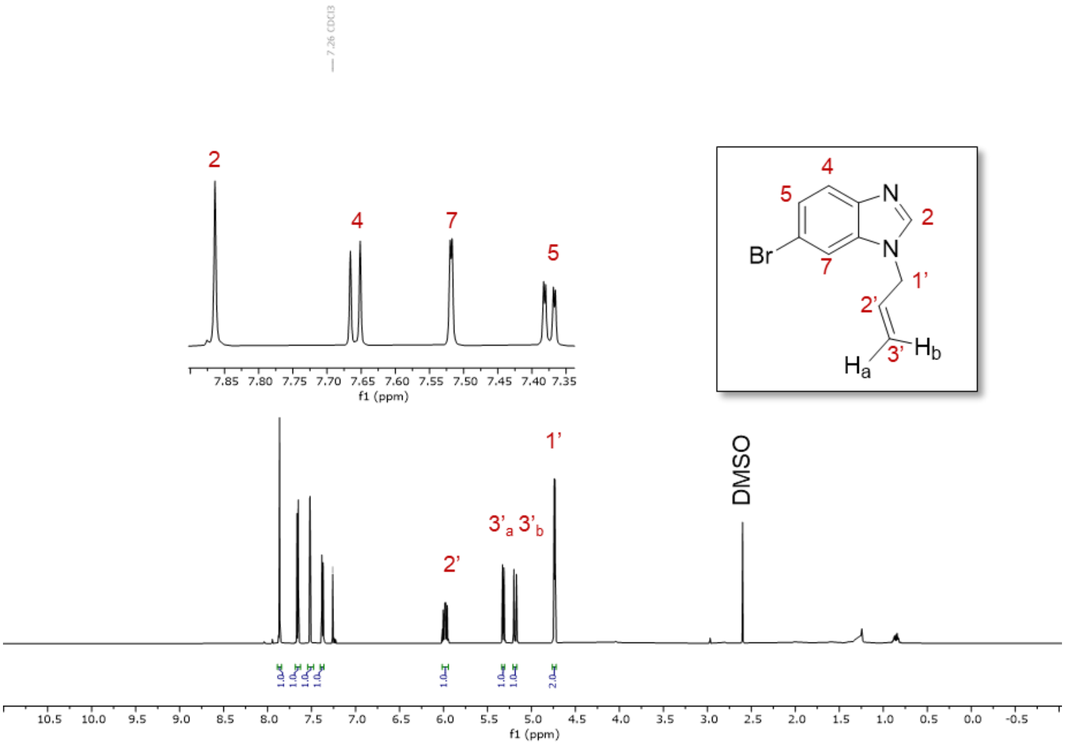


[1]H-NMR (600 MHz, $CDCl_3$) spectrum.

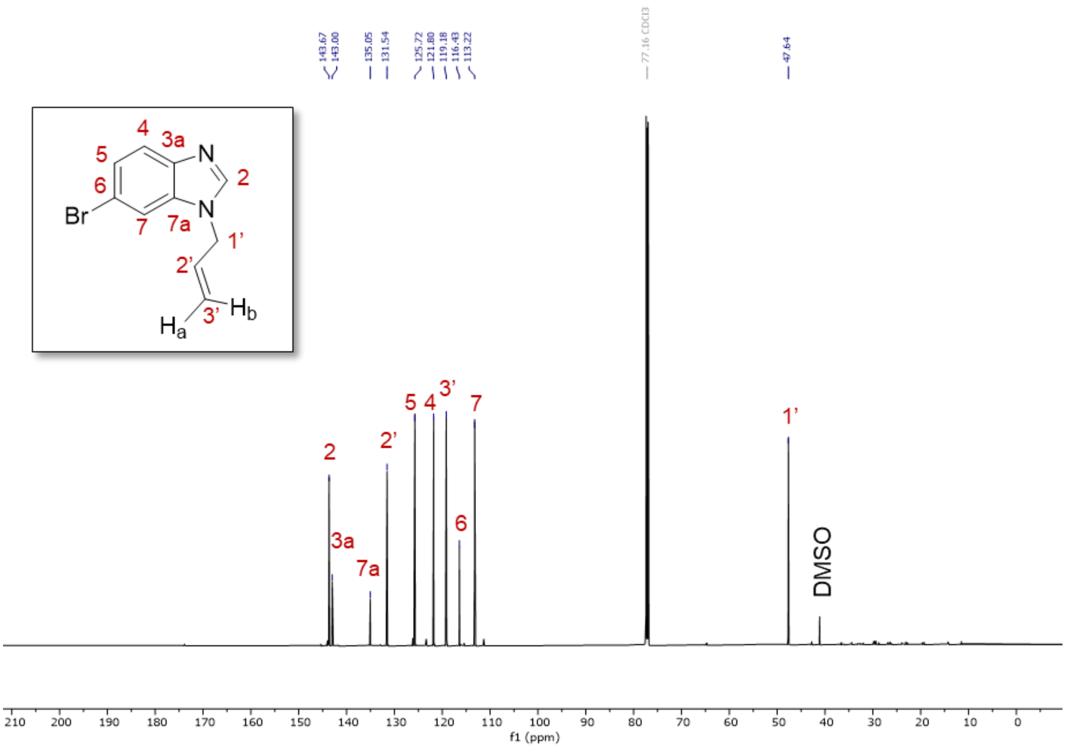


[13]C-NMR (151 MHz, $CDCl_3$) spectrum.

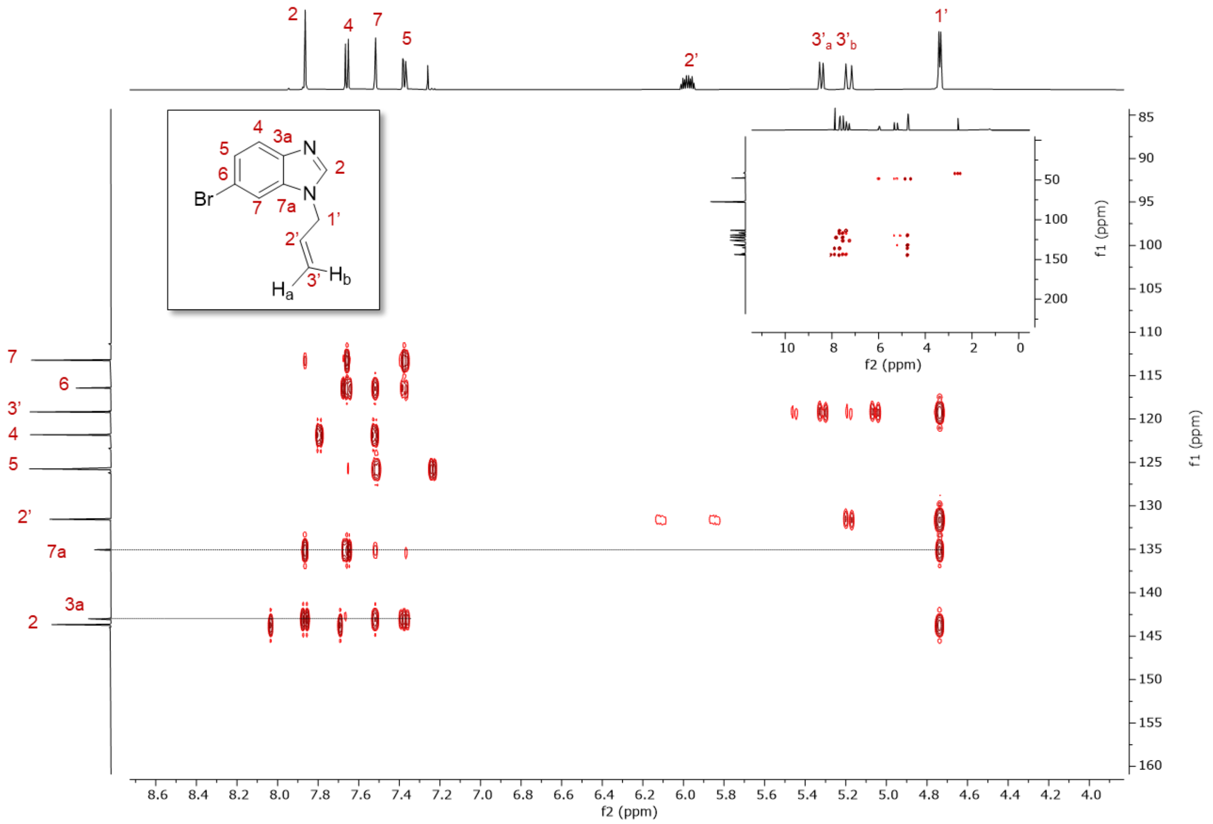


$^1H$,$^{13}C$ HMBC (600 MHz, $CDCl_3$) spectrum.

NMR spectra from chemical synthesis of 5-bromo-1-(2-methylallyl)-1*H*-benzo[*d*]imidazole and 6-bromo-1-(2-methylallyl)-1*H*-benzo[*d*]imidazole as mixture of regioisomers.

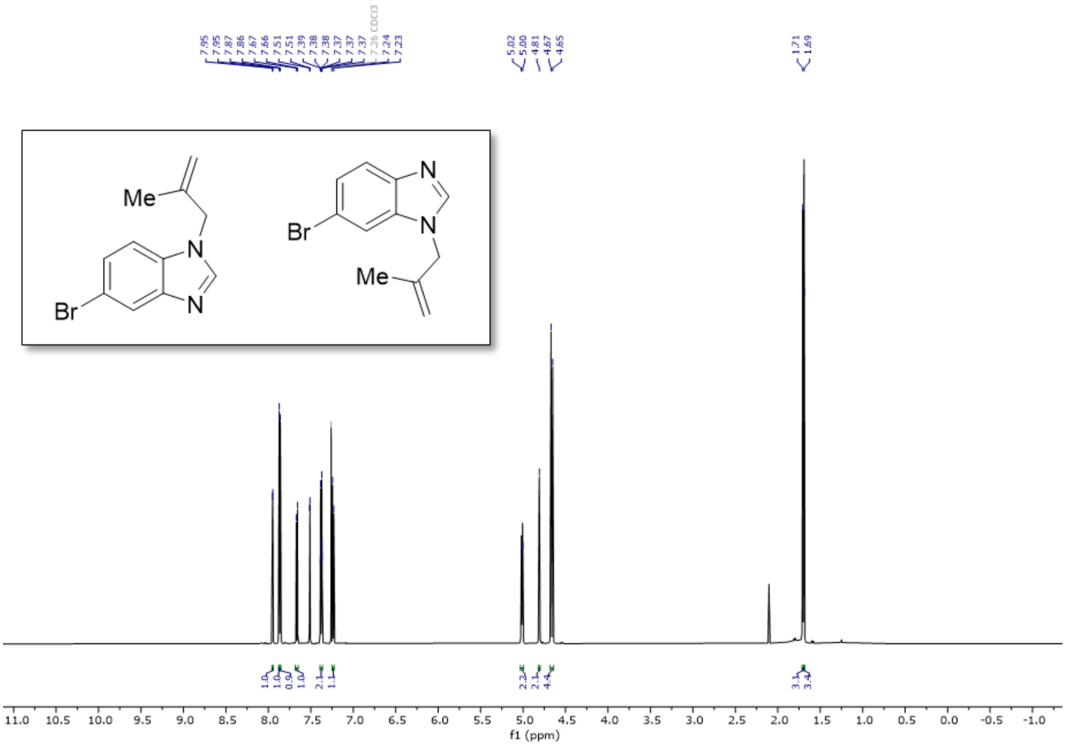


[1]H-NMR (600 MHz, $CDCl_3$) spectrum.

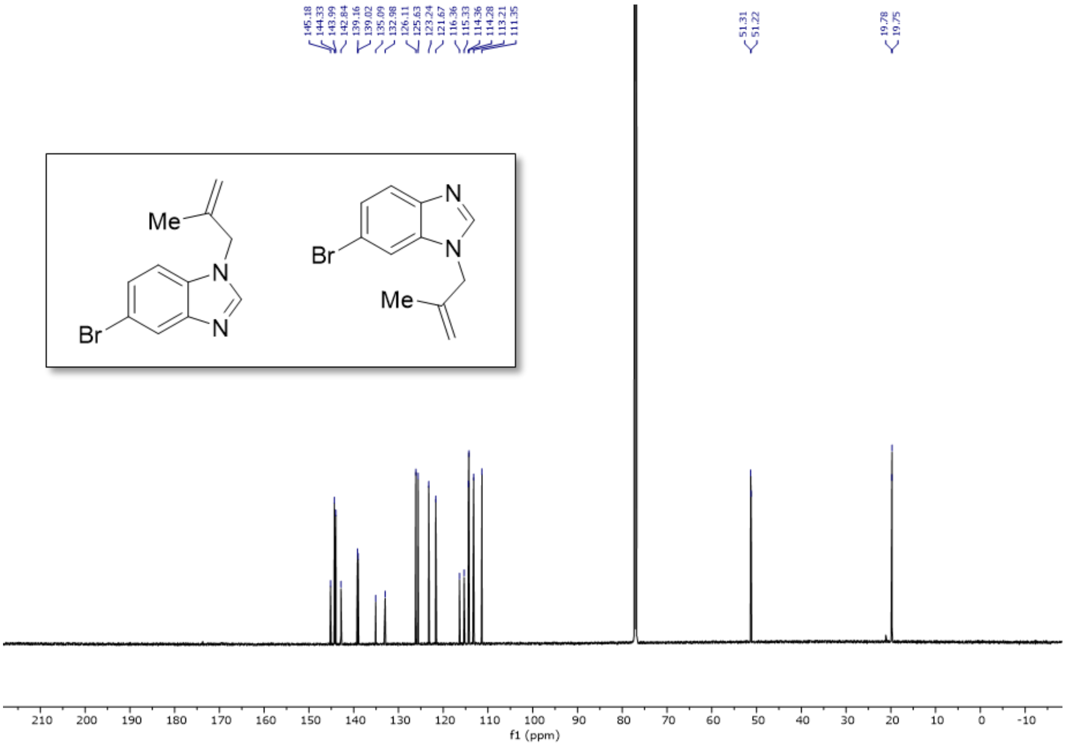


[13]C-NMR (151 MHz, $CDCl_3$) spectrum.

**Crude** NMR spectra from enzymatic synthesis of 6-bromo-1-(2-methylallyl)-1*H*-benzo[*d*]imidazole (**20**) to assign the regioisomer formed.

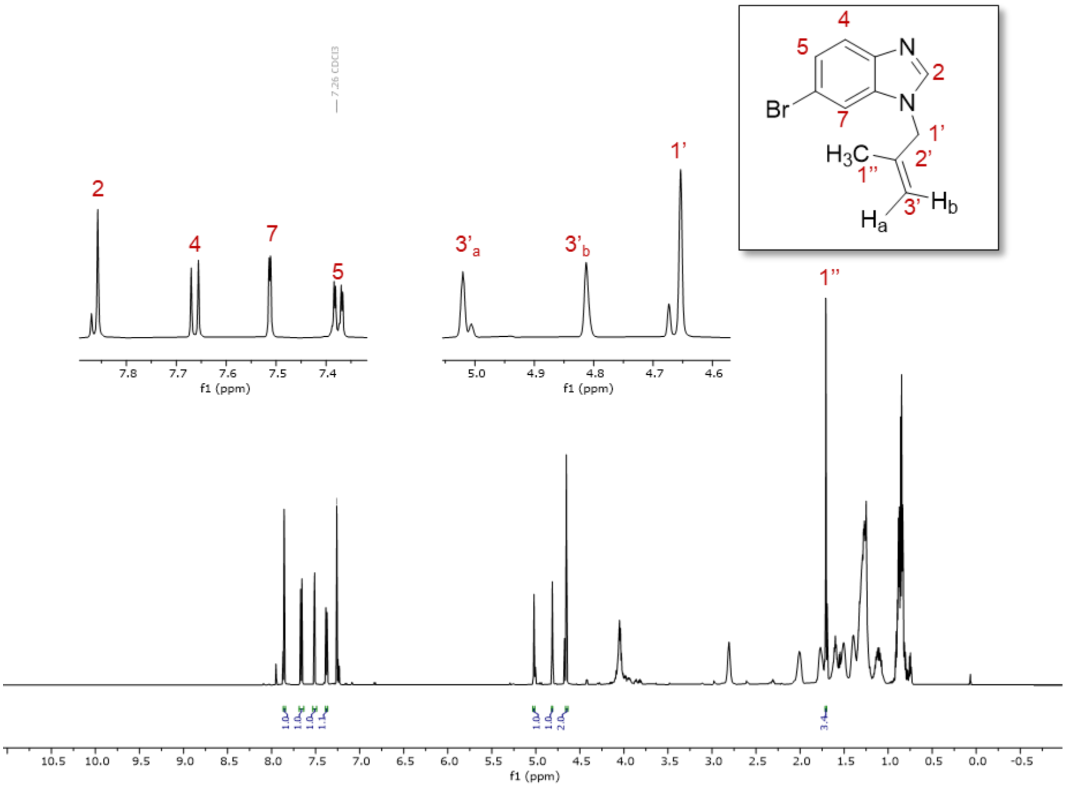


[1]H-NMR (600 MHz, $CDCl_3$) spectrum.

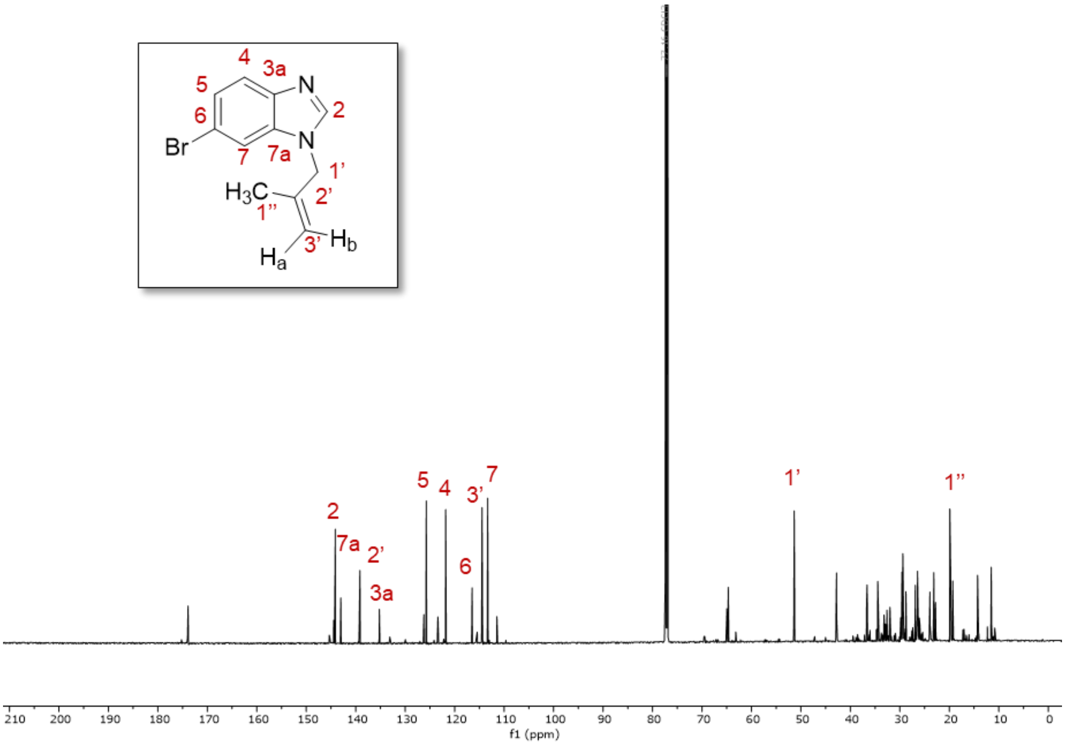


[13]C-NMR (151 MHz, $CDCl_3$) spectrum.

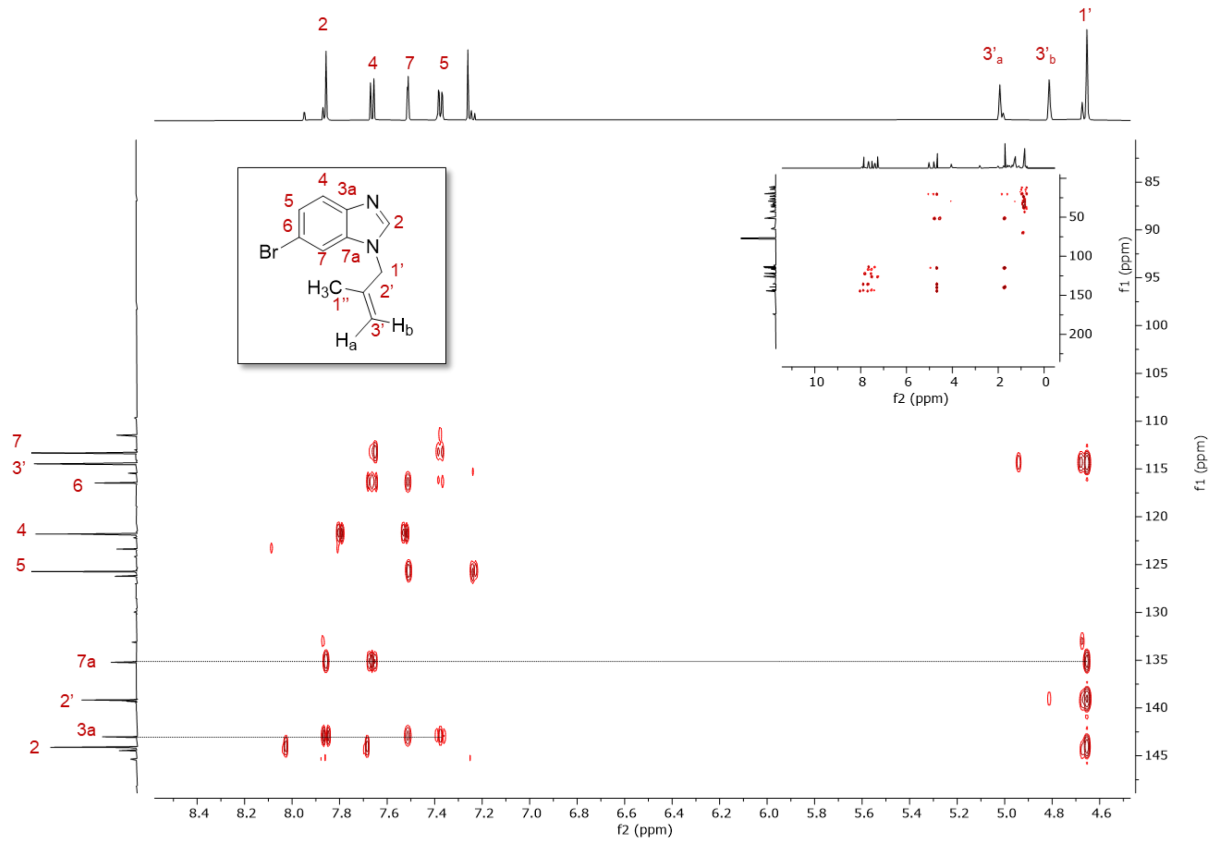


$^{1}H$,$^{13}C$ HMBC (600 MHz, $CDCl_3$) spectrum.

NMR spectra from chemical synthesis of 5-bromo-1-(2-fluoroallyl)-1*H*-benzo[*d*]imidazole and 6-bromo-1-(2-fluoroallyl)-1*H*-benzo[*d*]imidazole as mixture of regioisomers.

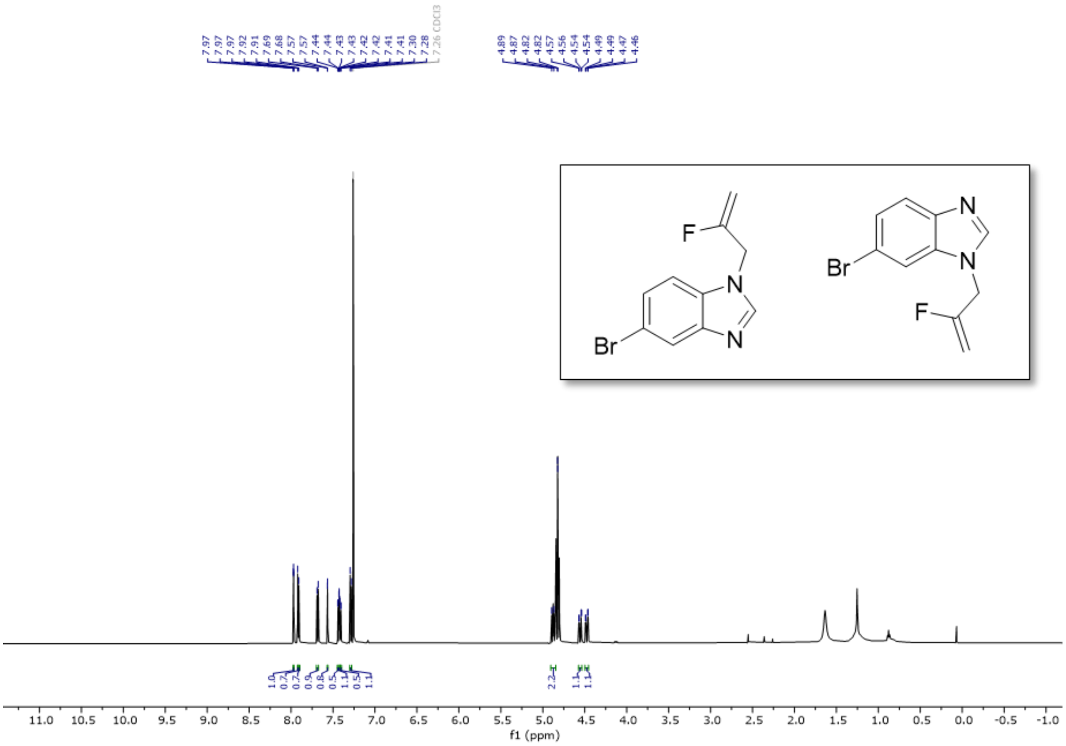


$^{1}$H-NMR (600 MHz, $CDCl_3$) spectrum.

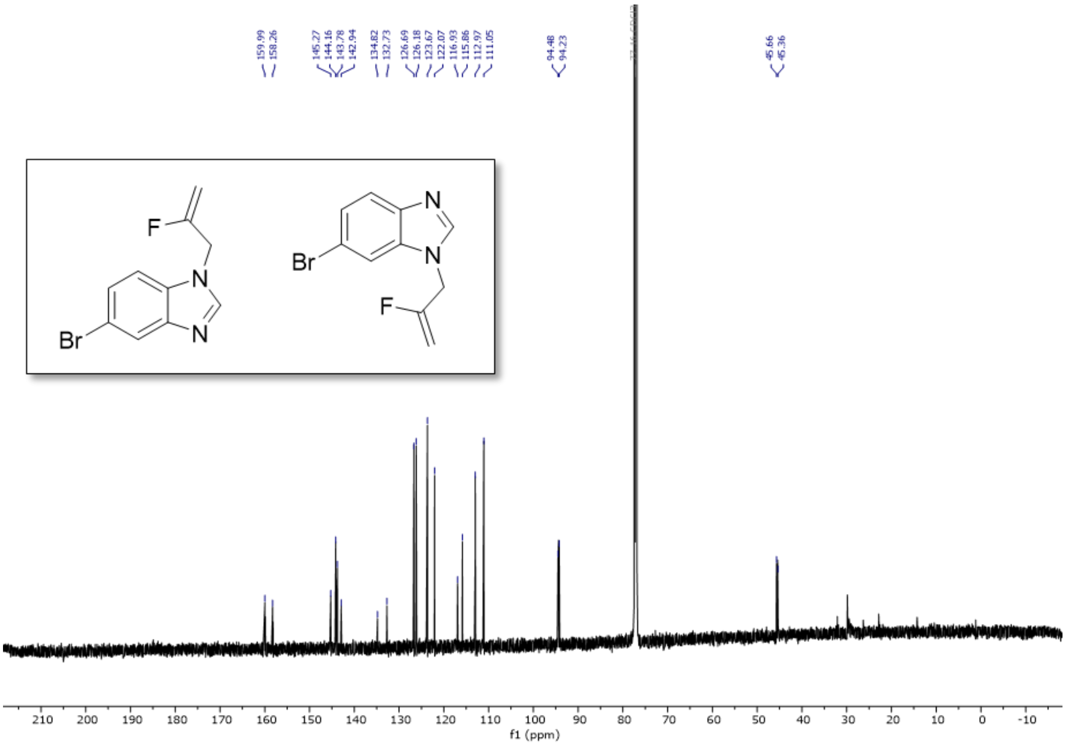


$^{13}$C-NMR (151 MHz, $CDCl_3$) spectrum.

<u>**Crude** NMR spectra from enzymatic synthesis of 6-bromo-1-(2-fluoroallyl)-1*H*-benzo[*d*]imidazole (**21**) to assign the regioisomer formed.</u>

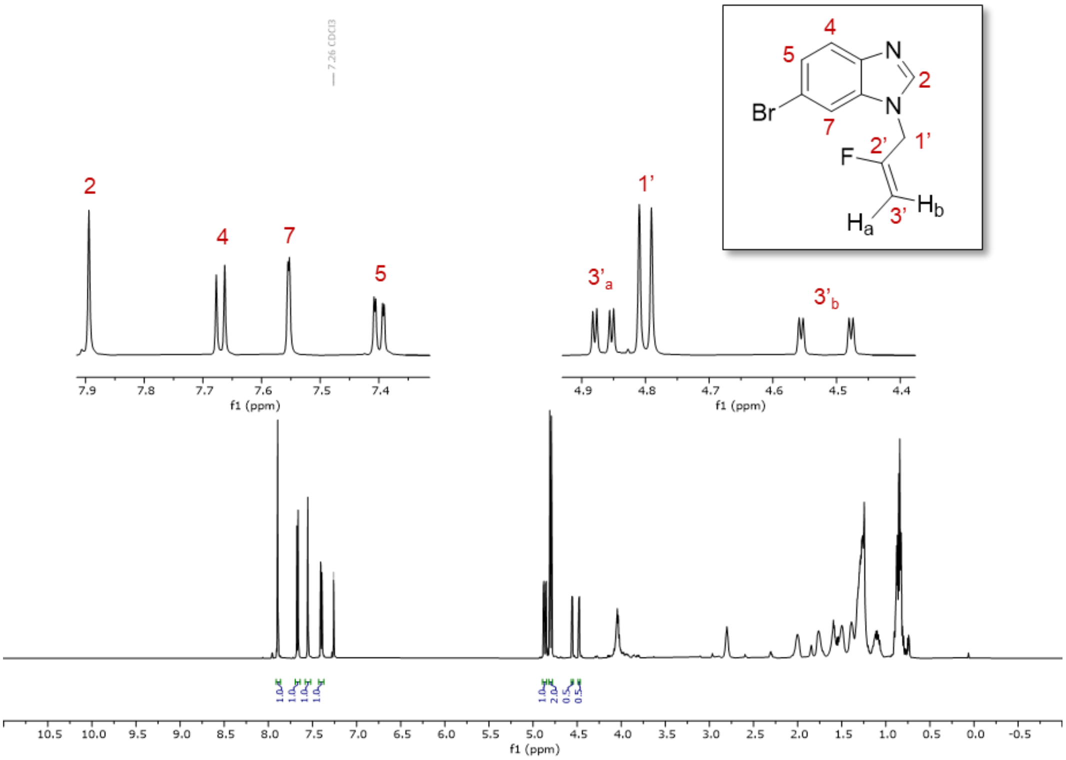


[1]H-NMR (600 MHz, $CDCl_3$) spectrum.

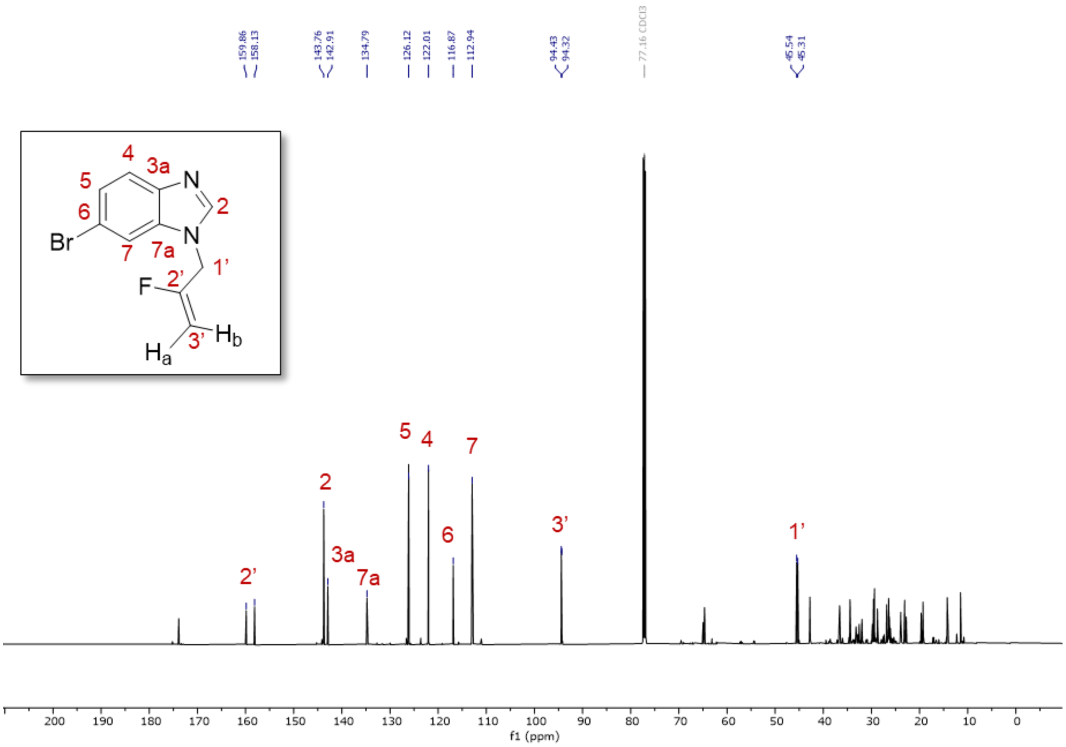


[13]C-NMR (151 MHz, $CDCl_3$) spectrum.

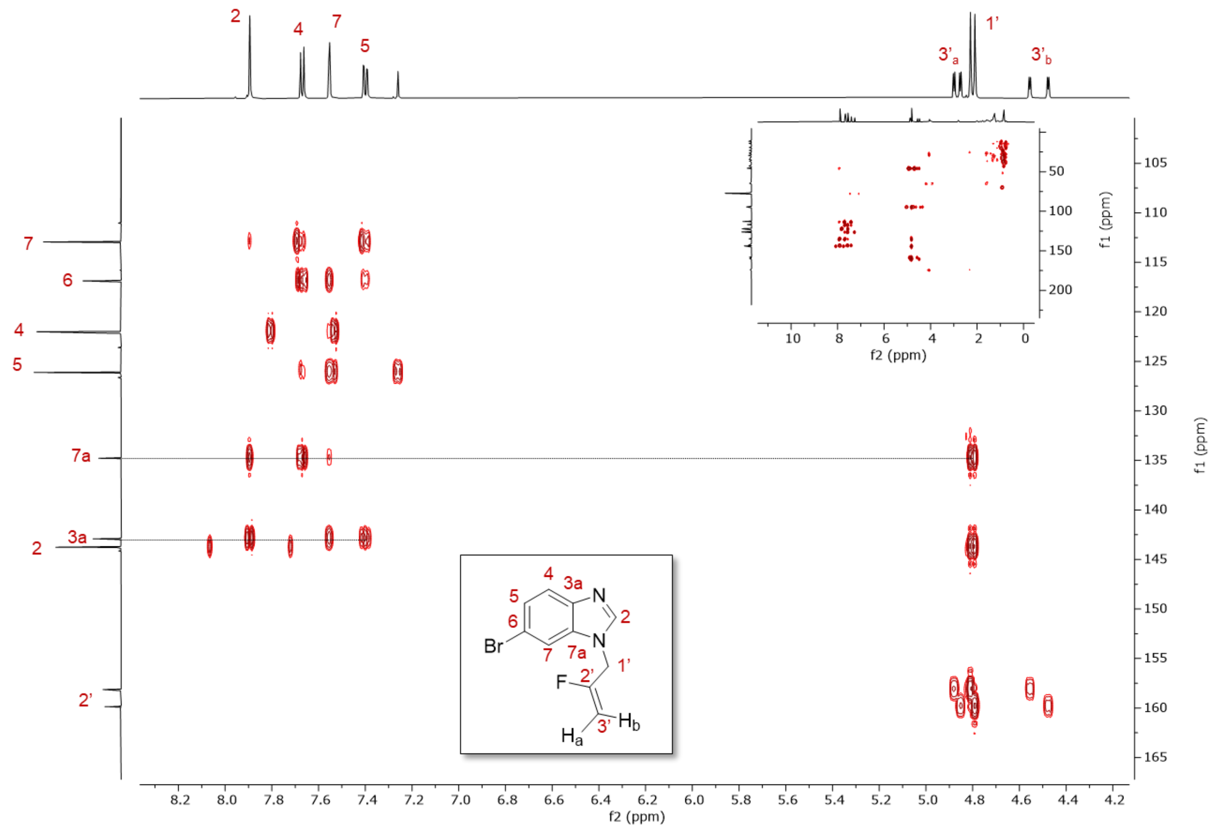


[1]H,[13]C HMBC (600 MHz, $CDCl_3$) spectrum.

NMR spectra from chemical synthesis of 2-(5-bromo-1*H*-benzo[*d*]imidazol-1-yl)acetonitrile and 2-(6-bromo-1*H*-benzo[*d*]imidazol-1-yl)acetonitrile as mixture of regioisomers.

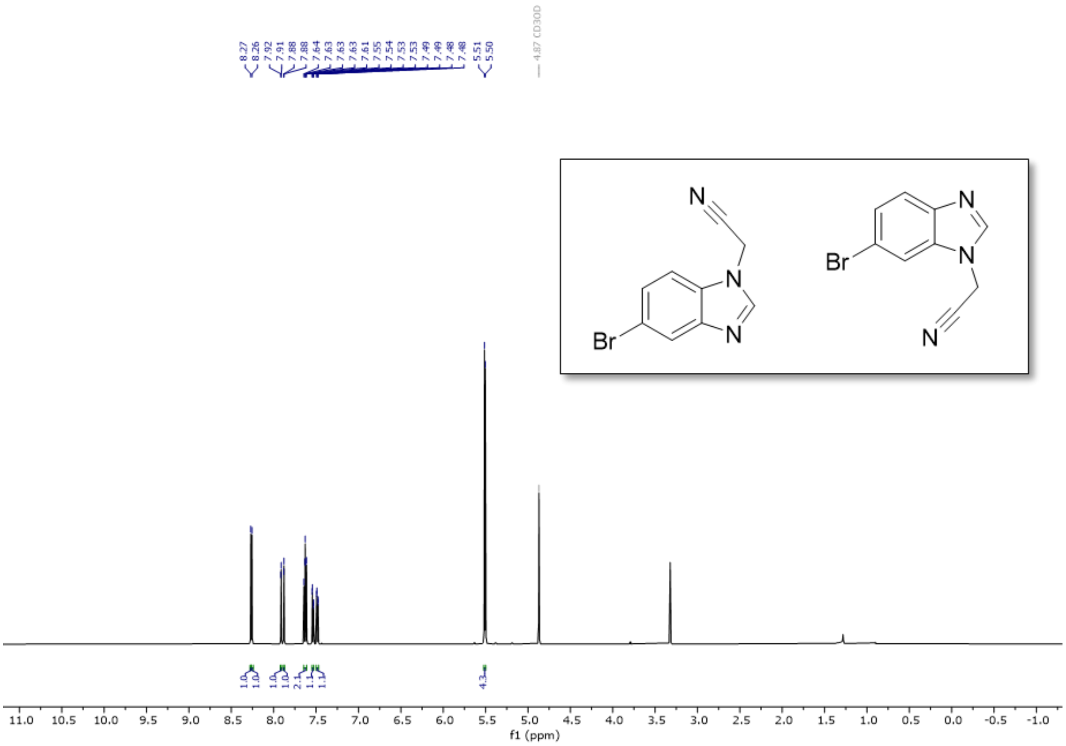


$^1$H-NMR (600 MHz, $CD_3OD$) spectrum.

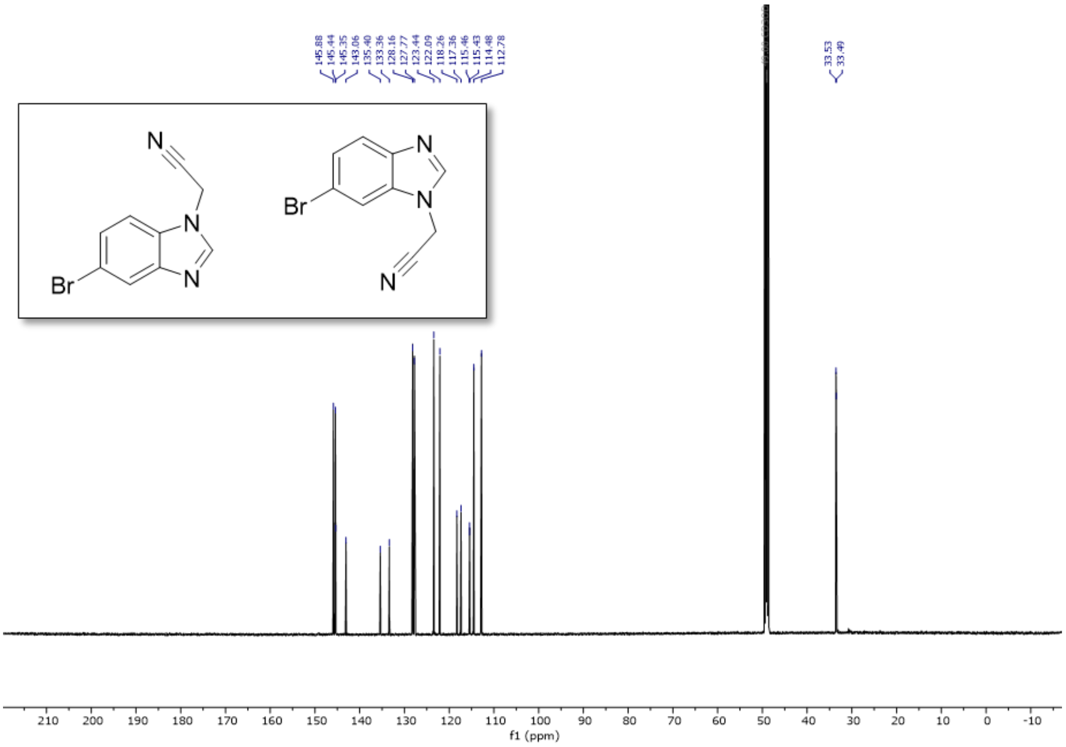


$^{13}$C-NMR (151 MHz, $CD_3OD$) spectrum.

NMR spectra from enzymatic synthesis of 2-(6-bromo-1*H*-benzo[*d*]imidazol-1-yl)acetonitrile (**22**).

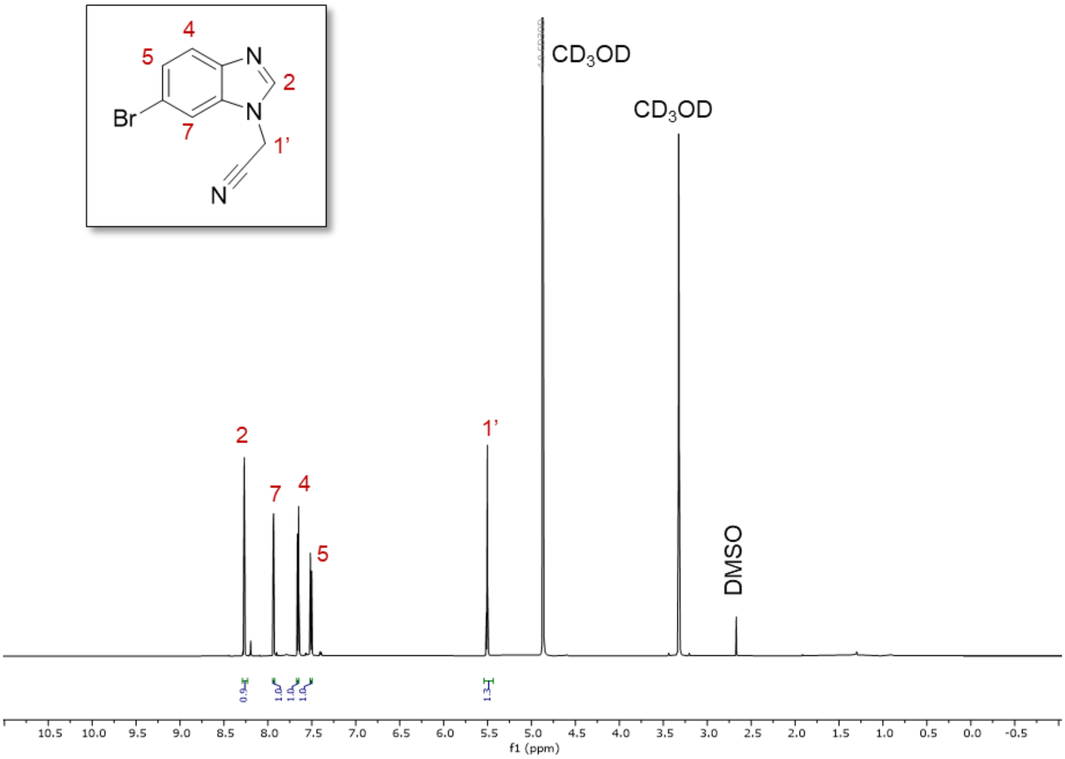


[1]H-NMR (600 MHz, $CD_3OD$) spectrum.

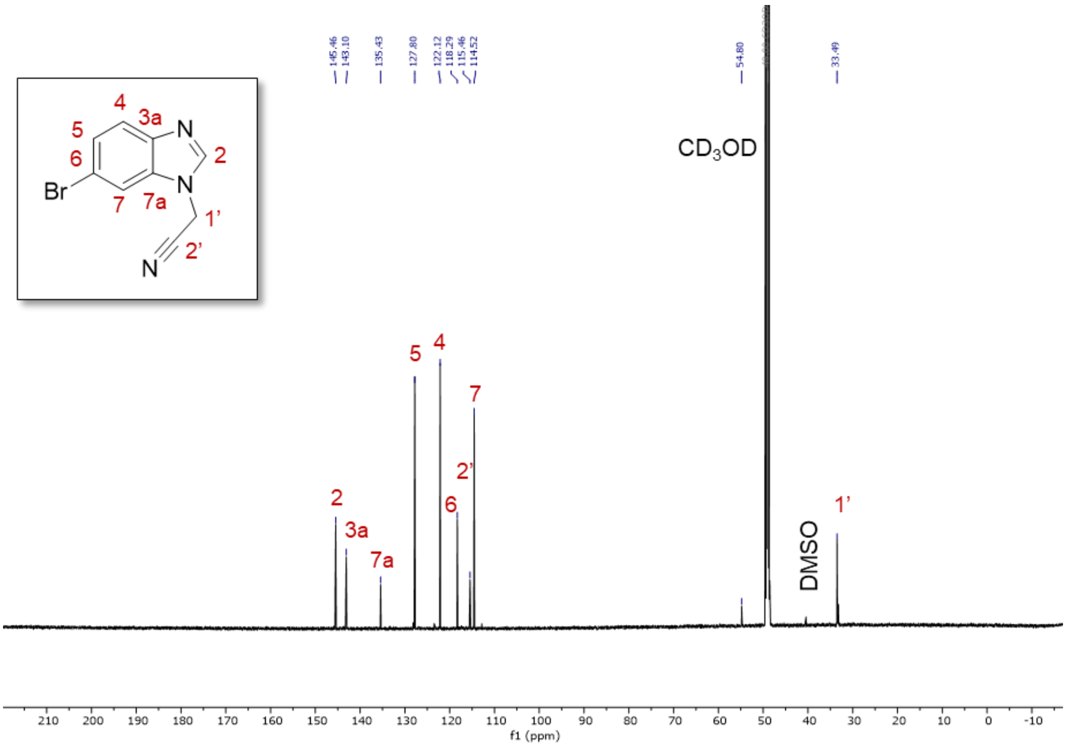


[13]C-NMR (151 MHz, $CD_3OD$) spectrum.

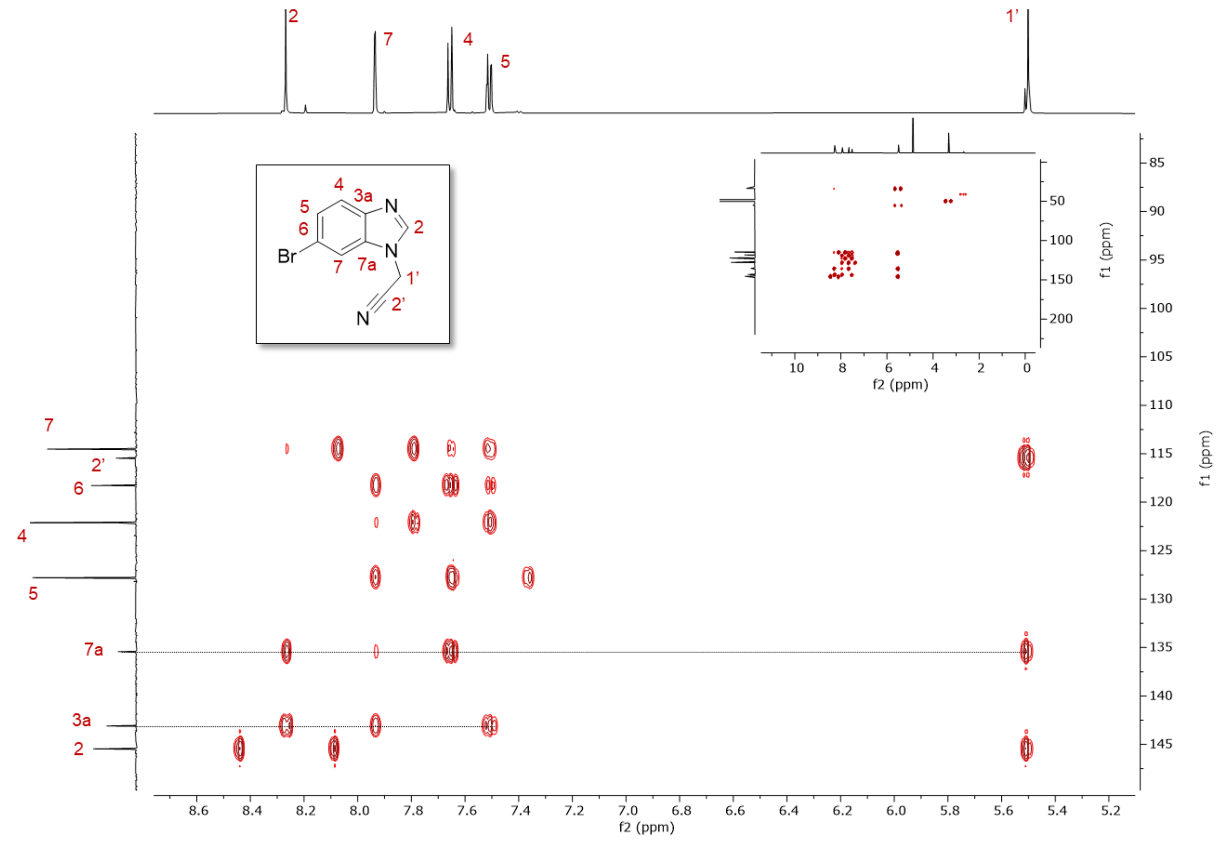


$^{1}H,^{13}C$ HMBC (600 MHz, $CD_3OD$) spectrum.

NMR spectra from chemical synthesis of 5-bromo-1-(prop-2-yn-1-yl)-1*H*-benzo[*d*]imidazole and 6-bromo-1-(prop-2-yn-1-yl)-1*H*-benzo[*d*]imidazole as mixture of regioisomers.

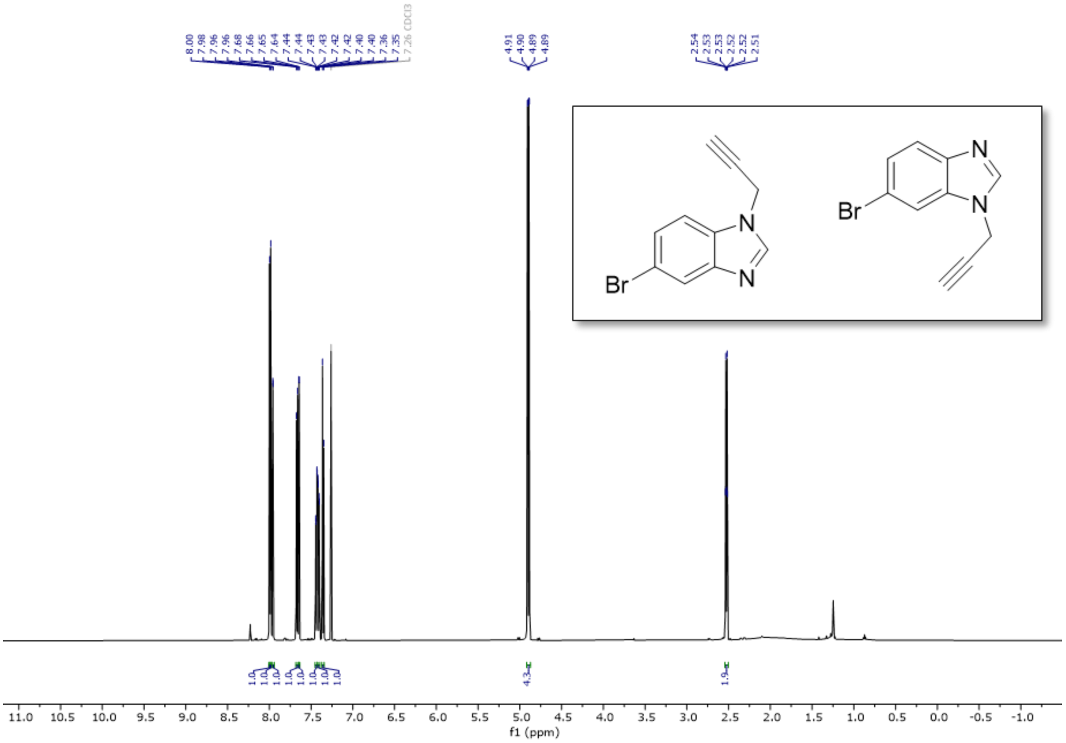


$^{1}H$-NMR (600 MHz, $CDCl_3$) spectrum.

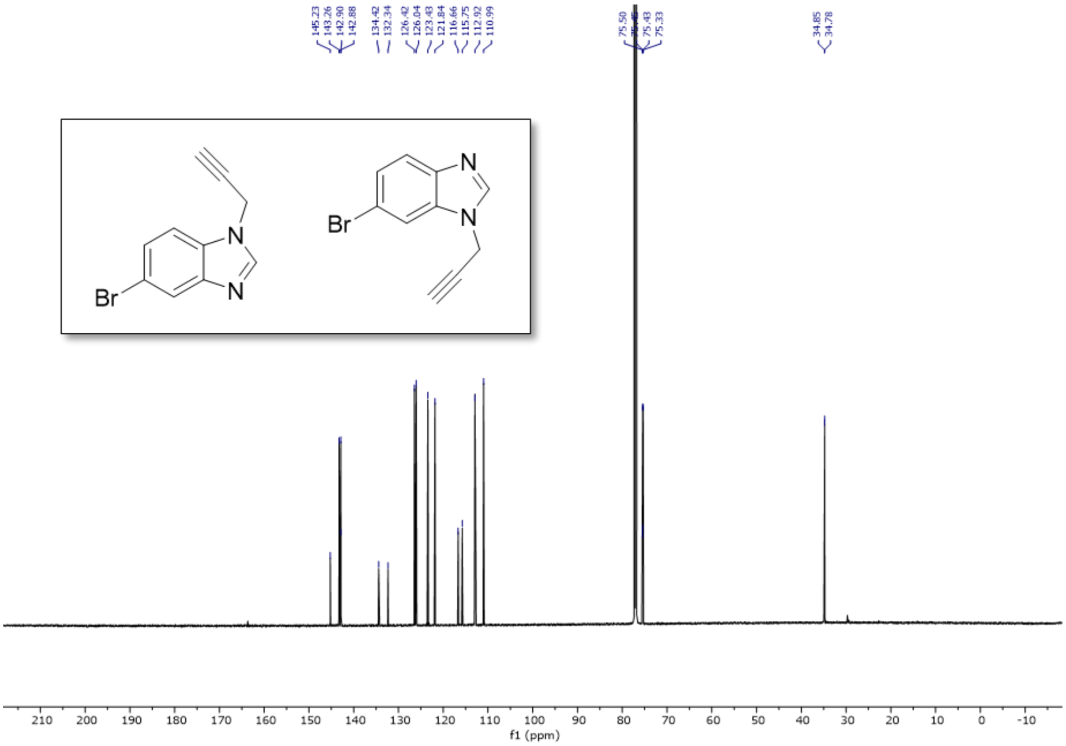


$^{13}C$-NMR (151 MHz, $CDCl_3$) spectrum.

NMR spectra from enzymatic synthesis of 6-bromo-1-(prop-2-yn-1-yl)-1*H*-benzo[*d*]imidazole (**23**).

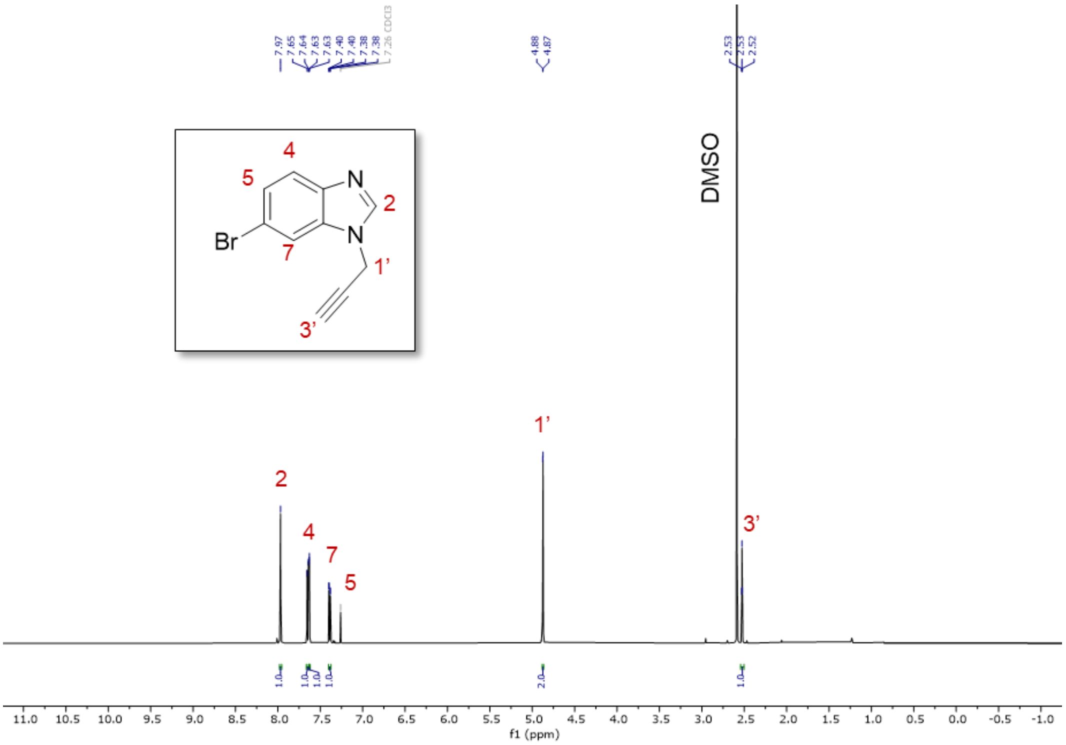


$^{1}$H-NMR (600 MHz, $CDCl_3$) spectrum.

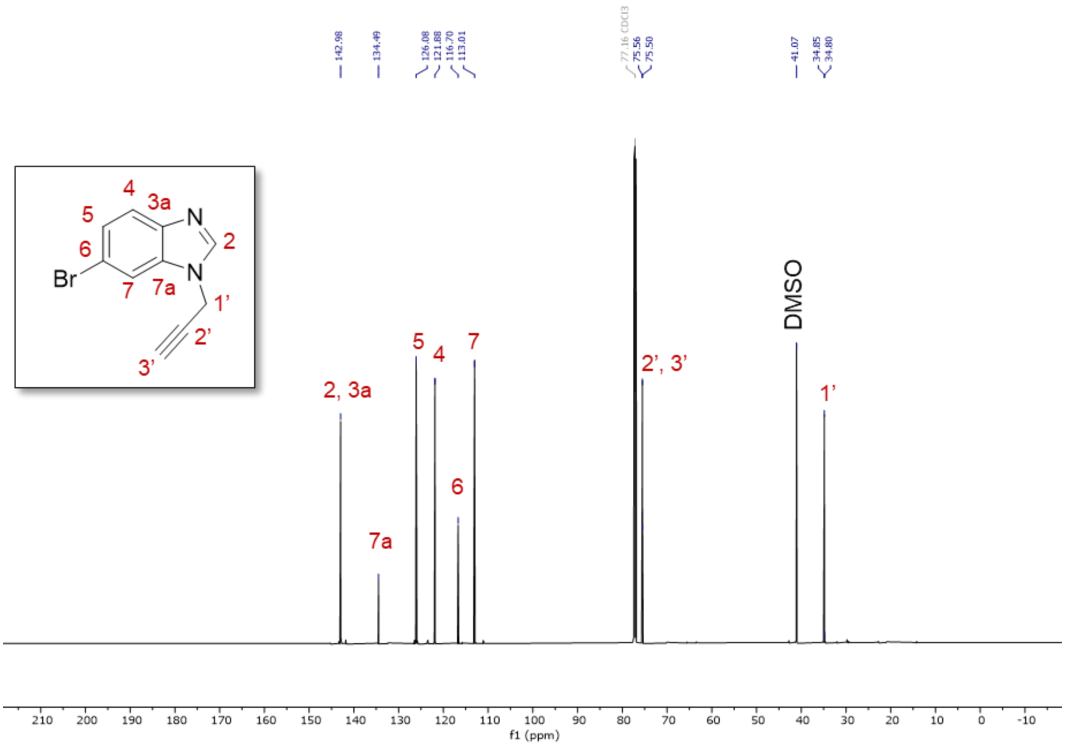


$^{13}$C-NMR (151 MHz, $CDCl_3$) spectrum.

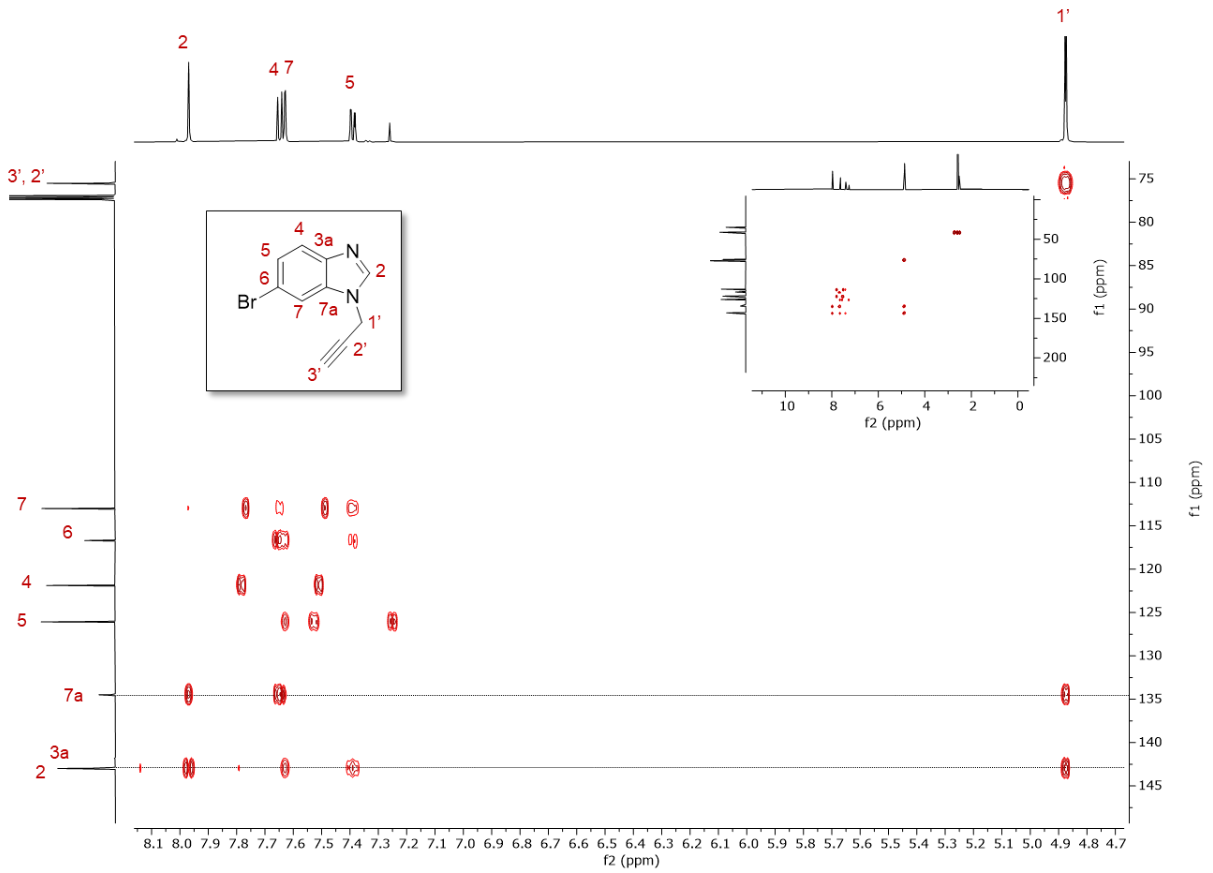


$^{1}H,^{13}C$ HMBC (600 MHz, $CDCl_3$) spectrum.

## VIII. DNA and amino acid sequences

### *acl*-MT

ATGTCAACGCCCAGTTTAATTCCATCCGGTGTGCACGAAGTATTAGCAAAATACAAAGACGGTAACTATGTCGAC
GGGTGGGCCGAACTGTGGGATAAATCTAAGGGTGACCGTCTTCCATGGGATCGTGGTTTTCCGAACCCTGCGTTG
GAAGATACCCTGATTCAGAAACGTGCCATCATCGGAGGTCCCTTGGGCCAAGACGCTCAGGGCAAGACATACCGC
AAAAAAGCTCTGGTCCCAGGCTGCGGTCGTGGAGTAGATGTGTTGTTATTGGCATCGTTCGGTTATGACGCTTAT
GGTTTAGAATACTCTGCAACGGCGGTCGATGTATGCCAAGAGGAGCAAGCCAAGAACGGGGATCAATATCCTGTA
CGCGATGCAGAAATCGGCCAGGGCAAGATTACGTTCGTTCAAGGTGATTTCTTTGAGGACACGTGGCTTGAAAAG
CTTAACCTTACCCGCAATTGTTTCGACGTTATCTACGACTACACGTTCTTCTGTGCATTGAACCCGAGCATGCGC
CCTCAGTGGGCTTTGCGCCATACCCAACTTCTTGCCGACTCTCCGCGCGGACATTTAATCTGTTTGGAGTTTCCC
CGCCATAAAGACCCTTCTGTACAGGGGCCACCATGGGGCTCGGCGTCAGAAGCCTACCGCGCCCATCTGTCACAT
CCAGGGGAGGAAATTCCCTACGATGCGTCACGTCAATGCCAGTTTGACAGCAGCAAAGCACCCAGTGCGCAGGGC
TTGGAACGTGTAGCATATTGGCAACCAGAGCGCACGCATGAAGTAGGGAAAAACGAGAAAGGTGAGGTTCAAGAT
CGCGTATCCATCTGGCAGCGTCCTCCCCAAAGTTCCCTGCTGGAACATCATCACCACCATCATTAA

MSTPSLIPSGVHEVLAKYKDGNYVDGWAELWDKSKGDRLPWDRGFPNPALEDTLIQKRAIIGGPLGQDAQGKTYR
KKALVPGCGRGVDVLLLASFGYDAYGLEYSATAVDVCQEEQAKNGDQYPVRDAEIGQGKITFVQGDFFEDTWLEK
LNLTRNCFDVIYDYTFFCALNPSMRPQWALRHTQLLADSPRGHLICLEFPRHKDPSVQGPPWGSASEAYRAHLSH
PGEEIPYDASRQCQFDSSKAPSAQGLERVAYWQPERTHEVGKNEKGEVQDRVSIWQRPPQSSLLEHHHHHH

### *hsa*-N-NMT

ATGGAGAGTGGATTTACGAGTAAGGACACTTATTTGAGTCATTTTAACCCGCGCGACTACTTAGAAAAATACTAC
AAGTTTGGAAGCCGCCATTCAGCAGAATCACAAATTCTGAAACACCTTTTAAAGAACCTGTTTAAAATCTTCTGT
TTAGATGGGGTCAAAGGAGATTTGTTGATTGACATCGGATCAGGTCCCACAATTTACCAACTTCTGTCCGCTTGT
GAGAGTTTTAAGGAGATCGTCGTGACGGACTATTCAGATCAAAATCTGCAAGAACTGGAGAAATGGTTAAAGAAA
GAACCAGAGGCGTTCGATTGGAGCCCAGTTGTGACCTATGTCTGTGATCTTGAAGGAAACCGCGTAAAGGGTCCT
GAAAAGGAGGAAAAGCTGCGCCAAGCCGTAAAGCAAGTATTAAAATGTGATGTTACTCAAAGCCAACCATTAGGA
GCCGTCCCACTGCCCCCGGCGGATTGTGTATTGTCAACCCTTTGCTTGGACGCAGCTTGTCCTGACTTGCCTACA
TATTGTCGTGCATTACGCAACTTAGGAAGTTTGCTTAAACCAGGAGGGTTTCTGGTCATCATGGATGCCTTGAAG
TCATCCTACTACATGATTGGGGAACAGAAGTTCTCATCTTTACCTTTGGGCCGTGAAGCCGTAGAAGCGGCCGTA
AAGGAGGCGGGGTACACCATTGAATGGTTCGAGGTTATTTCACAGTCCTACAGCTCCACAATGGCAAATAACGAG
GGCTTATTTTCGCTGGTTGCTCGCAAACTGAGTCGTCCTCTTCTCGAGCACCACCACCACCACCACTGA

MESGFTSKDTYLSHFNPRDYLEKYYKFGSRHSAESQILKHLLKNLFKIFCLDGVKGDLLIDIGSGPTIYQLLSAC
ESFKEIVVTDYSDQNLQELEKWLKKEPEAFDWSPVVTYVCDLEGNRVKGPEKEEKLRQAVKQVLKCDVTQSQPLG
AVPLPPADCVLSTLCLDAACPDLPTYCRALRNLGSLLKPGGFLVIMDALKSSYYMIGEQKFSSLPLGREAVEAAV
KEAGYTIEWFEVISQSYSSTMANNEGLFSLVARKLSRPLLEHHHHHH

### *hsa*-H-NMT

ATGGCGAGCTCTATGCGCAGCCTGTTTTCGGATCATGGGAAGTATGTAGAAAGCTTCCGCCGCTTCCTGAACCAC
AGTACCGAGCATCAATGTATGCAGGAATTCATGGACAAAAAGCTTCCGGGAATTATCGGCCGCATCGGTGATACT
AAGTCGGAAATCAAAATTCTTTCTATCGGGGGCGGCGCTGGGGAGATTGACCTTCAGATCCTGTCTAAGGTACAG
GCACAATATCCTGGAGTATGTATCAATAATGAAGTGGTCGAGCCTAGTGCGGAGCAAATCGCGAAGTATAAAGAA
TTAGTAGCGAAAACTAGCAATCTGGAGAATGTCAAATTTGCGTGGCATAAAGAAACGTCGTCTGAATATCAATCC
CGCATGCTTGAAAAAAAGGAGTTACAGAAATGGGACTTCATCCACATGATTCAAATGCTTTATTACGTCAAGGAC
ATCCCTGCTACACTGAAATTCTTTCATTCTCTGTTGGGGACTAATGCTAAGATGCTTATCATTGTTGTTTCTGGT
AGTTCAGGCTGGGACAAACTGTGGAAGAAATATGGAAGTCGCTTTCCTCAGGATGATTTATGCCAGTATATCACA
TCGGACGACCTTACCCAGATGCTGGATAATTTGGGATTAAAGTATGAGTGCTATGACTTGCTGTCAACCATGGAC
ATTTCGGATTGCTTTATTGATGGCAATGAGAATGGTGATTTATTGTGGGACTTTTTAACGGAGACCTGTAATTTT
AATGCAACTGCCCCACCCGATTTACGCGCGGAATTGGGAAAAGATTTGCAGGAGCCGGAATTCTCCGCGAAGAAA
GAGGGCAAGGTTCTTTTTAATAACACATTATCTTTTATCGTGATCGAAGCTCTCGAGCACCACCACCACCACCAC
TGA
MASSMRSLFSDHGKYVESFRRFLNHSTEHQCMQEFMDKKLPGIIGRIGDTKSEIKILSIGGGAGEIDLQILSKVQ
AQYPGVCINNEVVEPSAEQIAKYKELVAKTSNLENVKFAWHKETSSEYQSRMLEKKELQKWDFIHMIQMLYYVKD
IPATLKFFHSLLGTNAKMLIIVVSGSSGWDKLWKKYGSRFPQDDLCQYITSDDLTQMLDNLGLKYECYDLLSTMD
ISDCFIDGNENGDLLWDFLTETCNFNATAPPDLRAELGKDLQEPEFSAKKEGKVLFNNTLSFIVIEALEHHHHHH

v31 (*hsa*-N-NMT_D167H, A198M, S201C, Y242F, N249S)

ATGGAGAGTGGATTTACGAGTAAGGACACTTATTTGAGTCATTTTAACCCGCGCGACTACTTAGAAAAATACTAC
AAGTTTGGAAGCCGCCATTCAGCAGAATCACAAATTCTGAAACACCTTTTAAAGAACCTGTTTAAAATCTTCTGT
TTAGATGGGGTCAAAGGAGATTTGTTGATTGACATCGGATCAGGTCCCACAATTTACCAACTTCTGTCCGCTTGT
GAGAGTTTTAAGGAGATCGTCGTGACGGACTATTCAGATCAAAATCTGCAAGAACTGGAGAAATGGTTAAAGAAA
GAACCAGAGGCGTTCGATTGGAGCCCAGTTGTGACCTATGTCTGTGATCTTGAAGGAAACCGCGTAAAGGGTCCT
GAAAAGGAGGAAAAGCTGCGCCAAGCCGTAAAGCAAGTATTAAAATGTGATGTTACTCAAAGCCAACCATTAGGA
GCCGTCCCACTGCCCCCGGCGGATTGTGTATTGTCAACCCTTTGCTTGcACGCAGCTTGTCCTGACTTGCCTACA
TATTGTCGTGCATTACGCAACTTAGGAAGTTTGCTTAAACCAGGAGGGTTTCTGGTCATCATGGATatgTTGAAG
TgcTCCTACTACATGATTGGGGAACAGAAGTTCTCATCTTTACCTTTGGGCCGTGAAGCCGTAGAAGCGGCCGTA
AAGGAGGCGGGGTACACCATTGAATGGTTCGAGGTTATTTCACAGTCCTtCAGCTCCACAATGGCAAATtcCGAG
GGCTTATTTTCGCTGGTTGCTCGCAAACTGAGTCGTCCTCTTCTCGAGCACCACCACCACCACCACTGA

MESGFTSKDTYLSHFNPRDYLEKYYKFGSRHSAESQILKHLLKNLFKIFCLDGVKGDLLIDIGSGPTIYQLLSAC
ESFKEIVVTDYSDQNLQELEKWLKKEPEAFDWSPVVTYVCDLEGNRVKGPEKEEKLRQAVKQVLKCDVTQSQPLG
AVPLPPADCVLSTLCLHAACPDLPTYCRALRNLGSLLKPGGFLVIMDMLKCSYYMIGEQKFSSLPLGREAVEAAV
KEAGYTIEWFEVISQSFSSTMANSEGLFSLVARKLSRPLLEHHHHHH

v47 (*hsa*-N-NMT_D167E, D197G, S201N, S213A, Y242F)

ATGGAGAGTGGATTTACGAGTAAGGACACTTATTTGAGTCATTTTAACCCGCGCGACTACTTAGAAAAATACTAC
AAGTTTGGAAGCCGCCATTCAGCAGAATCACAAATTCTGAAACACCTTTTAAAGAACCTGTTTAAAATCTTCTGT
TTAGATGGGGTCAAAGGAGATTTGTTGATTGACATCGGATCAGGTCCCACAATTTACCAACTTCTGTCCGCTTGT
GAGAGTTTTAAGGAGATCGTCGTGACGGACTATTCAGATCAAAATCTGCAAGAACTGGAGAAATGGTTAAAGAAA
GAACCAGAGGCGTTCGATTGGAGCCCAGTTGTGACCTATGTCTGTGATCTTGAAGGAAACCGCGTAAAGGGTCCT
GAAAAGGAGGAAAAGCTGCGCCAAGCCGTAAAGCAAGTATTAAAATGTGATGTTACTCAAAGCCAACCATTAGGA
GCCGTCCCACTGCCCCCGGCGGATTGTGTATTGTCAACCCTTTGCTTGGAaGCAGCTTGTCCTGACTTGCCTACA
TATTGTCGTGCATTACGCAACTTAGGAAGTTTGCTTAAACCAGGAGGGTTTCTGGTCATCATGGgTGCCTTGAAG
aacTCCTACTACATGATTGGGGAACAGAAGTTCTCAgCgTTACCTTTGGGCCGTGAAGCCGTAGAAGCGGCCGTA
AAGGAGGCGGGGTACACCATTGAATGGTTCGAGGTTATTTCACAGTCCTtCAGCTCCACAATGGCAAATAACGAG
GGCTTATTTTCGCTGGTTGCTCGCAAACTGAGTCGTCCTCTTCTCGAGCACCACCACCACCACCACTGA

MESGFTSKDTYLSHFNPRDYLEKYYKFGSRHSAESQILKHLLKNLFKIFCLDGVKGDLLIDIGSGPTIYQLLSAC
ESFKEIVVTDYSDQNLQELEKWLKKEPEAFDWSPVVTYVCDLEGNRVKGPEKEEKLRQAVKQVLKCDVTQSQPLG
AVPLPPADCVLSTLCLEAACPDLPTYCRALRNLGSLLKPGGFLVIMGALKNSYYMIGEQKFSALPLGREAVEAAV
KEAGYTIEWFEVISQSFSSTMANNEGLFSLVARKLSRPLLEHHHHHH

vS01 (*hsa*-N-NMT_A74G, A134V, D167S, A198H, S201C, Y242F, N249S)

ATGGAGAGTGGATTTACGAGTAAGGACACTTATTTGAGTCATTTTAACCCGCGCGACTACTTAGAAAAATACTAC
AAGTTTGGAAGCCGCCATTCAGCAGAATCACAAATTCTGAAACACCTTTTAAAGAACCTGTTTAAAATCTTCTGT
TTAGATGGGGTCAAAGGAGATTTGTTGATTGACATCGGATCAGGTCCCACAATTTACCAACTTCTGTCCGGGTGT
GAGAGTTTTAAGGAGATCGTCGTGACGGACTATTCAGATCAAAATCTGCAAGAACTGGAGAAATGGTTAAAGAAA
GAACCAGAGGCGTTCGATTGGAGCCCAGTTGTGACCTATGTCTGTGATCTTGAAGGAAACCGCGTAAAGGGTCCT
GAAAAGGAGGAAAAGCTGCGCCAAGTGGTAAAGCAAGTATTAAAATGTGATGTTACTCAAAGCCAACCATTAGGA
GCCGTCCCACTGCCCCCGGCGGATTGTGTATTGTCAACCCTTTGCTTGTCGGCAGCTTGTCCTGACTTGCCTACA
TATTGTCGTGCATTACGCAACTTAGGAAGTTTGCTTAAACCAGGAGGGTTTCTGGTCATCATGGATCACTTGAAG
TGCTCCTACTACATGATTGGGGAACAGAAGTTCTCATCTTTACCTTTGGGCCGTGAAGCCGTAGAAGCGGCCGTA
AAGGAGGCGGGGTACACCATTGAATGGTTCGAGGTTATTTCACAGTCCTTCAGCTCCACAATGGCAAATTCCGAG
GGCTTATTTTCGCTGGTTGCTCGCAAACTGAGTCGTCCTCTTCTCGAGCACCACCACCACCACCACTGA

MESGFTSKDTYLSHFNPRDYLEKYYKFGSRHSAESQILKHLLKNLFKIFCLDGVKGDLLIDIGSGPTIYQLLSGC
ESFKEIVVTDYSDQNLQELEKWLKKEPEAFDWSPVVTYVCDLEGNRVKGPEKEEKLRQVVKQVLKCDVTQSQPLG
AVPLPPADCVLSTLCLSAACPDLPTYCRALRNLGSLLKPGGFLVIMDHLKCSYYMIGEQKFSSLPLGREAVEAAV
KEAGYTIEWFEVISQSFSSTMANSEGLFSLVARKLSRPLLEHHHHHH

vS02 (*hsa*-N-NMT_A74G, A134V, D167H, A198H, S201C, Y242P, N249S)
ATGGAGAGTGGATTTACGAGTAAGGACACTTATTTGAGTCATTTTAACCCGCGCGACTACTTAGAAAAATACTAC
AAGTTTGGAAGCCGCCATTCAGCAGAATCACAAATTCTGAAACACCTTTTAAAGAACCTGTTTAAAATCTTCTGT
TTAGATGGGGTCAAAGGAGATTTGTTGATTGACATCGGATCAGGTCCCACAATTTACCAACTTCTGTCCGGGTGT
GAGAGTTTTAAGGAGATCGTCGTGACGGACTATTCAGATCAAAATCTGCAAGAACTGGAGAAATGGTTAAAGAAA
GAACCAGAGGCGTTCGATTGGAGCCCAGTTGTGACCTATGTCTGTGATCTTGAAGGAAACCGCGTAAAGGGTCCT
GAAAAGGAGGAAAAGCTGCGCCAAGTGGTAAAGCAAGTATTAAAATGTGATGTTACTCAAAGCCAACCATTAGGA
GCCGTCCCACTGCCCCCGGCGGATTGTGTATTGTCAACCCTTTGCTTGCACGCAGCTTGTCCTGACTTGCCTACA
TATTGTCGTGCATTACGCAACTTAGGAAGTTTGCTTAAACCAGGAGGGTTTCTGGTCATCATGGATCACTTGAAG
TGCTCCTACTACATGATTGGGGAACAGAAGTTCTCATCTTTACCTTTGGGCCGTGAAGCCGTAGAAGCGGCCGTA
AAGGAGGCGGGGTACACCATTGAATGGTTCGAGGTTATTTCACAGTCCCCGAGCTCCACAATGGCAAATTCCGAG
GGCTTATTTTCGCTGGTTGCTCGCAAACTGAGTCGTCCTCTTCTCGAGCACCACCACCACCACCACTGA

MESGFTSKDTYLSHFNPRDYLEKYYKFGSRHSAESQILKHLLKNLFKIFCLDGVKGDLLIDIGSGPTIYQLLSGC
ESFKEIVVTDYSDQNLQELEKWLKKEPEAFDWSPVVTYVCDLEGNRVKGPEKEEKLRQVVKQVLKCDVTQSQPLG
AVPLPPADCVLSTLCLHAACPDLPTYCRALRNLGSLLKPGGFLVIMDHLKCSYYMIGEQKFSSLPLGREAVEAAV
KEAGYTIEWFEVISQSPSSTMANSEGLFSLVARKLSRPLLEHHHHHH

vS03 (*hsa*-N-NMT_A74G, A134V, D167H, A198H, S201C, Y242F, A247T, N249S)
ATGGAGAGTGGATTTACGAGTAAGGACACTTATTTGAGTCATTTTAACCCGCGCGACTACTTAGAAAAATACTAC
AAGTTTGGAAGCCGCCATTCAGCAGAATCACAAATTCTGAAACACCTTTTAAAGAACCTGTTTAAAATCTTCTGT
TTAGATGGGGTCAAAGGAGATTTGTTGATTGACATCGGATCAGGTCCCACAATTTACCAACTTCTGTCCGGGTGT
GAGAGTTTTAAGGAGATCGTCGTGACGGACTATTCAGATCAAAATCTGCAAGAACTGGAGAAATGGTTAAAGAAA
GAACCAGAGGCGTTCGATTGGAGCCCAGTTGTGACCTATGTCTGTGATCTTGAAGGAAACCGCGTAAAGGGTCCT
GAAAAGGAGGAAAAGCTGCGCCAAGTGGTAAAGCAAGTATTAAAATGTGATGTTACTCAAAGCCAACCATTAGGA
GCCGTCCCACTGCCCCCGGCGGATTGTGTATTGTCAACCCTTTGCTTGCACGCAGCTTGTCCTGACTTGCCTACA
TATTGTCGTGCATTACGCAACTTAGGAAGTTTGCTTAAACCAGGAGGGTTTCTGGTCATCATGGATCACTTGAAG
TGCTCCTACTACATGATTGGGGAACAGAAGTTCTCATCTTTACCTTTGGGCCGTGAAGCCGTAGAAGCGGCCGTA
AAGGAGGCGGGGTACACCATTGAATGGTTCGAGGTTATTTCACAGTCCTTCAGCTCCACAATGACAAATTCCGAG
GGCTTATTTTCGCTGGTTGCTCGCAAACTGAGTCGTCCTCTTCTCGAGCACCACCACCACCACCACTGA

MESGFTSKDTYLSHFNPRDYLEKYYKFGSRHSAESQILKHLLKNLFKIFCLDGVKGDLLIDIGSGPTIYQLLSGC
ESFKEIVVTDYSDQNLQELEKWLKKEPEAFDWSPVVTYVCDLEGNRVKGPEKEEKLRQVVKQVLKCDVTQSQPLG
AVPLPPADCVLSTLCLHAACPDLPTYCRALRNLGSLLKPGGFLVIMDHLKCSYYMIGEQKFSSLPLGREAVEAAV
KEAGYTIEWFEVISQSFSSTMTNSEGLFSLVARKLSRPLLEHHHHHH

vS04 (*hsa*-N-NMT_A74G, A134V, D167H, A198Y, S201C, Y242P, A247C, N249G)
ATGGAGAGTGGATTTACGAGTAAGGACACTTATTTGAGTCATTTTAACCCGCGCGACTACTTAGAAAAATACTAC
AAGTTTGGAAGCCGCCATTCAGCAGAATCACAAATTCTGAAACACCTTTTAAAGAACCTGTTTAAAATCTTCTGT
TTAGATGGGGTCAAAGGAGATTTGTTGATTGACATCGGATCAGGTCCCACAATTTACCAACTTCTGTCCGGGTGT
GAGAGTTTTAAGGAGATCGTCGTGACGGACTATTCAGATCAAAATCTGCAAGAACTGGAGAAATGGTTAAAGAAA
GAACCAGAGGCGTTCGATTGGAGCCCAGTTGTGACCTATGTCTGTGATCTTGAAGGAAACCGCGTAAAGGGTCCT
GAAAAGGAGGAAAAGCTGCGCCAAGTGGTAAAGCAAGTATTAAAATGTGATGTTACTCAAAGCCAACCATTAGGA
GCCGTCCCACTGCCCCCGGCGGATTGTGTATTGTCAACCCTTTGCTTGCACGCAGCTTGTCCTGACTTGCCTACA
TATTGTCGTGCATTACGCAACTTAGGAAGTTTGCTTAAACCAGGAGGGTTTCTGGTCATCATGGATTATTTGAAG
TGCTCCTACTACATGATTGGGGAACAGAAGTTCTCATCTTTACCTTTGGGCCGTGAAGCCGTAGAAGCGGCCGTA
AAGGAGGCGGGGTACACCATTGAATGGTTCGAGGTTATTTCACAGTCCCCGAGCTCCACAATGTGTAATGGGGAG
GGCTTATTTTCGCTGGTTGCTCGCAAACTGAGTCGTCCTCTTCTCGAGCACCACCACCACCACCACTGA

MESGFTSKDTYLSHFNPRDYLEKYYKFGSRHSAESQILKHLLKNLFKIFCLDGVKGDLLIDIGSGPTIYQLLSGC
ESFKEIVVTDYSDQNLQELEKWLKKEPEAFDWSPVVTYVCDLEGNRVKGPEKEEKLRQVVKQVLKCDVTQSQPLG
AVPLPPADCVLSTLCLHAACPDLPTYCRALRNLGSLLKPGGFLVIMDYLKCSYYMIGEQKFSSLPLGREAVEAAV
KEAGYTIEWFEVISQSPSSTMCNGEGLFSLVARKLSRPLLEHHHHHH

vS05 (*hsa*-N-NMT_A74G, A134V, D167H, A198Y, S201C, Y242P, N249G)
ATGGAGAGTGGATTTACGAGTAAGGACACTTATTTGAGTCATTTTAACCCGCGCGACTACTTAGAAAAATACTAC
AAGTTTGGAAGCCGCCATTCAGCAGAATCACAAATTCTGAAACACCTTTTAAAGAACCTGTTTAAAATCTTCTGT
TTAGATGGGGTCAAAGGAGATTTGTTGATTGACATCGGATCAGGTCCCACAATTTACCAACTTCTGTCCGGGTGT
GAGAGTTTTAAGGAGATCGTCGTGACGGACTATTCAGATCAAAATCTGCAAGAACTGGAGAAATGGTTAAAGAAA
GAACCAGAGGCGTTCGATTGGAGCCCAGTTGTGACCTATGTCTGTGATCTTGAAGGAAACCGCGTAAAGGGTCCT
GAAAAGGAGGAAAAGCTGCGCCAAGTGGTAAAGCAAGTATTAAAATGTGATGTTACTCAAAGCCAACCATTAGGA
GCCGTCCCACTGCCCCCGGCGGATTGTGTATTGTCAACCCTTTGCTTGCACGCAGCTTGTCCTGACTTGCCTACA
TATTGTCGTGCATTACGCAACTTAGGAAGTTTGCTTAAACCAGGAGGGTTTCTGGTCATCATGGATTATTTGAAG
TGCTCCTACTACATGATTGGGGAACAGAAGTTCTCATCTTTACCTTTGGGCCGTGAAGCCGTAGAAGCGGCCGTA
AAGGAGGCGGGGTACACCATTGAATGGTTCGAGGTTATTTCACAGTCCCCGAGCTCCACAATGGCAAATGGGGAG
GGCTTATTTTCGCTGGTTGCTCGCAAACTGAGTCGTCCTCTTCTCGAGCACCACCACCACCACCACTGA

MESGFTSKDTYLSHFNPRDYLEKYYKFGSRHSAESQILKHLLKNLFKIFCLDGVKGDLLIDIGSGPTIYQLLSGC
ESFKEIVVTDYSDQNLQELEKWLKKEPEAFDWSPVVTYVCDLEGNRVKGPEKEEKLRQVVKQVLKCDVTQSQPLG
AVPLPPADCVLSTLCLHAACPDLPTYCRALRNLGSLLKPGGFLVIMDYLKCSYYMIGEQKFSSLPLGREAVEAAV
KEAGYTIEWFEVISQSPSSTMANGEGLFSLVARKLSRPLLEHHHHHH

vS06 (*hsa*-N-NMT_A74G, L154Q, D167H, A198H, S201C, Y242F, N249S)
ATGGAGAGTGGATTTACGAGTAAGGACACTTATTTGAGTCATTTTAACCCGCGCGACTACTTAGAAAAATACTAC
AAGTTTGGAAGCCGCCATTCAGCAGAATCACAAATTCTGAAACACCTTTTAAAGAACCTGTTTAAAATCTTCTGT
TTAGATGGGGTCAAAGGAGATTTGTTGATTGACATCGGATCAGGTCCCACAATTTACCAACTTCTGTCCGGGTGT
GAGAGTTTTAAGGAGATCGTCGTGACGGACTATTCAGATCAAAATCTGCAAGAACTGGAGAAATGGTTAAAGAAA
GAACCAGAGGCGTTCGATTGGAGCCCAGTTGTGACCTATGTCTGTGATCTTGAAGGAAACCGCGTAAAGGGTCCT
GAAAAGGAGGAAAAGCTGCGCCAAGCCGTAAAGCAAGTATTAAAATGTGATGTTACTCAAAGCCAACCATTAGGA
GCCGTCCCACAGCCCCCGGCGGATTGTGTATTGTCAACCCTTTGCTTGCACGCAGCTTGTCCTGACTTGCCTACA
TATTGTCGTGCATTACGCAACTTAGGAAGTTTGCTTAAACCAGGAGGGTTTCTGGTCATCATGGATCACTTGAAG
TGCTCCTACTACATGATTGGGGAACAGAAGTTCTCATCTTTACCTTTGGGCCGTGAAGCCGTAGAAGCGGCCGTA
AAGGAGGCGGGGTACACCATTGAATGGTTCGAGGTTATTTCACAGTCCTTCAGCTCCACAATGGCAAATTCCGAG
GGCTTATTTTCGCTGGTTGCTCGCAAACTGAGTCGTCCTCTTCTCGAGCACCACCACCACCACCACTGA

MESGFTSKDTYLSHFNPRDYLEKYYKFGSRHSAESQILKHLLKNLFKIFCLDGVKGDLLIDIGSGPTIYQLLSGC
ESFKEIVVTDYSDQNLQELEKWLKKEPEAFDWSPVVTYVCDLEGNRVKGPEKEEKLRQAVKQVLKCDVTQSQPLG
AVPQPPADCVLSTLCLHAACPDLPTYCRALRNLGSLLKPGGFLVIMDHLKCSYYMIGEQKFSSLPLGREAVEAAV
KEAGYTIEWFEVISQSFSSTMANSEGLFSLVARKLSRPLLEHHHHHH

vS07 (*hsa*-N-NMT_A74G, D167S, A198H, S201C, Y242F, N249S)
ATGGAGAGTGGATTTACGAGTAAGGACACTTATTTGAGTCATTTTAACCCGCGCGACTACTTAGAAAAATACTAC
AAGTTTGGAAGCCGCCATTCAGCAGAATCACAAATTCTGAAACACCTTTTAAAGAACCTGTTTAAAATCTTCTGT
TTAGATGGGGTCAAAGGAGATTTGTTGATTGACATCGGATCAGGTCCCACAATTTACCAACTTCTGTCCGGGTGT
GAGAGTTTTAAGGAGATCGTCGTGACGGACTATTCAGATCAAAATCTGCAAGAACTGGAGAAATGGTTAAAGAAA
GAACCAGAGGCGTTCGATTGGAGCCCAGTTGTGACCTATGTCTGTGATCTTGAAGGAAACCGCGTAAAGGGTCCT
GAAAAGGAGGAAAAGCTGCGCCAAGCCGTAAAGCAAGTATTAAAATGTGATGTTACTCAAAGCCAACCATTAGGA
GCCGTCCCACTGCCCCCGGCGGATTGTGTATTGTCAACCCTTTGCTTGTCGGCAGCTTGTCCTGACTTGCCTACA
TATTGTCGTGCATTACGCAACTTAGGAAGTTTGCTTAAACCAGGAGGGTTTCTGGTCATCATGGATCACTTGAAG
TGCTCCTACTACATGATTGGGGAACAGAAGTTCTCATCTTTACCTTTGGGCCGTGAAGCCGTAGAAGCGGCCGTA
AAGGAGGCGGGGTACACCATTGAATGGTTCGAGGTTATTTCACAGTCCTTCAGCTCCACAATGGCAAATTCCGAG
GGCTTATTTTCGCTGGTTGCTCGCAAACTGAGTCGTCCTCTTCTCGAGCACCACCACCACCACCACTGA

MESGFTSKDTYLSHFNPRDYLEKYYKFGSRHSAESQILKHLLKNLFKIFCLDGVKGDLLIDIGSGPTIYQLLSGC
ESFKEIVVTDYSDQNLQELEKWLKKEPEAFDWSPVVTYVCDLEGNRVKGPEKEEKLRQAVKQVLKCDVTQSQPLG
AVPLPPADCVLSTLCLSAACPDLPTYCRALRNLGSLLKPGGFLVIMDHLKCSYYMIGEQKFSSLPLGREAVEAAV
KEAGYTIEWFEVISQSFSSTMANSEGLFSLVARKLSRPLLEHHHHHH

vS08 (*hsa*-N-NMT_A74G, D167S, A198H, S201C, Y242P, N249S)
ATGGAGAGTGGATTTACGAGTAAGGACACTTATTTGAGTCATTTTAACCCGCGCGACTACTTAGAAAAATACTAC
AAGTTTGGAAGCCGCCATTCAGCAGAATCACAAATTCTGAAACACCTTTTAAAGAACCTGTTTAAAATCTTCTGT
TTAGATGGGGTCAAAGGAGATTTGTTGATTGACATCGGATCAGGTCCCACAATTTACCAACTTCTGTCCGGGTGT
GAGAGTTTTAAGGAGATCGTCGTGACGGACTATTCAGATCAAAATCTGCAAGAACTGGAGAAATGGTTAAAGAAA
GAACCAGAGGCGTTCGATTGGAGCCCAGTTGTGACCTATGTCTGTGATCTTGAAGGAAACCGCGTAAAGGGTCCT
GAAAAGGAGGAAAAGCTGCGCCAAGCCGTAAAGCAAGTATTAAAATGTGATGTTACTCAAAGCCAACCATTAGGA
GCCGTCCCACTGCCCCCGGCGGATTGTGTATTGTCAACCCTTTGCTTGTCGGCAGCTTGTCCTGACTTGCCTACA
TATTGTCGTGCATTACGCAACTTAGGAAGTTTGCTTAAACCAGGAGGGTTTCTGGTCATCATGGATCACTTGAAG
TGCTCCTACTACATGATTGGGGAACAGAAGTTCTCATCTTTACCTTTGGGCCGTGAAGCCGTAGAAGCGGCCGTA
AAGGAGGCGGGGTACACCATTGAATGGTTCGAGGTTATTTCACAGTCCCCGAGCTCCACAATGGCAAATTCCGAG
GGCTTATTTTCGCTGGTTGCTCGCAAACTGAGTCGTCCTCTTCTCGAGCACCACCACCACCACCACTGA

MESGFTSKDTYLSHFNPRDYLEKYYKFGSRHSAESQILKHLLKNLFKIFCLDGVKGDLLIDIGSGPTIYQLLSGC
ESFKEIVVTDYSDQNLQELEKWLKKEPEAFDWSPVVTYVCDLEGNRVKGPEKEEKLRQAVKQVLKCDVTQSQPLG
AVPLPPADCVLSTLCLSAACPDLPTYCRALRNLGSLLKPGGFLVIMDHLKCSYYMIGEQKFSSLPLGREAVEAAV
KEAGYTIEWFEVISQSPSSTMANSEGLFSLVARKLSRPLLEHHHHHH

vS09 (*hsa*-N-NMT_A74G, D167S, A198Y, S201C, Y242P, A247C, N249G)
ATGGAGAGTGGATTTACGAGTAAGGACACTTATTTGAGTCATTTTAACCCGCGCGACTACTTAGAAAAATACTAC
AAGTTTGGAAGCCGCCATTCAGCAGAATCACAAATTCTGAAACACCTTTTAAAGAACCTGTTTAAAATCTTCTGT
TTAGATGGGGTCAAAGGAGATTTGTTGATTGACATCGGATCAGGTCCCACAATTTACCAACTTCTGTCCGGGTGT
GAGAGTTTTAAGGAGATCGTCGTGACGGACTATTCAGATCAAAATCTGCAAGAACTGGAGAAATGGTTAAAGAAA
GAACCAGAGGCGTTCGATTGGAGCCCAGTTGTGACCTATGTCTGTGATCTTGAAGGAAACCGCGTAAAGGGTCCT
GAAAAGGAGGAAAAGCTGCGCCAAGCCGTAAAGCAAGTATTAAAATGTGATGTTACTCAAAGCCAACCATTAGGA
GCCGTCCCACTGCCCCCGGCGGATTGTGTATTGTCAACCCTTTGCTTGTCGGCAGCTTGTCCTGACTTGCCTACA
TATTGTCGTGCATTACGCAACTTAGGAAGTTTGCTTAAACCAGGAGGGTTTCTGGTCATCATGGATTATTTGAAG
TGCTCCTACTACATGATTGGGGAACAGAAGTTCTCATCTTTACCTTTGGGCCGTGAAGCCGTAGAAGCGGCCGTA
AAGGAGGCGGGGTACACCATTGAATGGTTCGAGGTTATTTCACAGTCCCCGAGCTCCACAATGTGTAATGGGGAG
GGCTTATTTTCGCTGGTTGCTCGCAAACTGAGTCGTCCTCTTCTCGAGCACCACCACCACCACCACTGA

MESGFTSKDTYLSHFNPRDYLEKYYKFGSRHSAESQILKHLLKNLFKIFCLDGVKGDLLIDIGSGPTIYQLLSGC
ESFKEIVVTDYSDQNLQELEKWLKKEPEAFDWSPVVTYVCDLEGNRVKGPEKEEKLRQAVKQVLKCDVTQSQPLG
AVPLPPADCVLSTLCLSAACPDLPTYCRALRNLGSLLKPGGFLVIMDYLKCSYYMIGEQKFSSLPLGREAVEAAV
KEAGYTIEWFEVISQSPSSTMCNGEGLFSLVARKLSRPLLEHHHHHH

vS10 (*hsa*-N-NMT_A74G, D167H, A198H, S201C, Y242F, N249S)
ATGGAGAGTGGATTTACGAGTAAGGACACTTATTTGAGTCATTTTAACCCGCGCGACTACTTAGAAAAATACTAC
AAGTTTGGAAGCCGCCATTCAGCAGAATCACAAATTCTGAAACACCTTTTAAAGAACCTGTTTAAAATCTTCTGT
TTAGATGGGGTCAAAGGAGATTTGTTGATTGACATCGGATCAGGTCCCACAATTTACCAACTTCTGTCCGGGTGT
GAGAGTTTTAAGGAGATCGTCGTGACGGACTATTCAGATCAAAATCTGCAAGAACTGGAGAAATGGTTAAAGAAA
GAACCAGAGGCGTTCGATTGGAGCCCAGTTGTGACCTATGTCTGTGATCTTGAAGGAAACCGCGTAAAGGGTCCT
GAAAAGGAGGAAAAGCTGCGCCAAGCCGTAAAGCAAGTATTAAAATGTGATGTTACTCAAAGCCAACCATTAGGA
GCCGTCCCACTGCCCCCGGCGGATTGTGTATTGTCAACCCTTTGCTTGCACGCAGCTTGTCCTGACTTGCCTACA
TATTGTCGTGCATTACGCAACTTAGGAAGTTTGCTTAAACCAGGAGGGTTTCTGGTCATCATGGATCACTTGAAG
TGCTCCTACTACATGATTGGGGAACAGAAGTTCTCATCTTTACCTTTGGGCCGTGAAGCCGTAGAAGCGGCCGTA
AAGGAGGCGGGGTACACCATTGAATGGTTCGAGGTTATTTCACAGTCCTTCAGCTCCACAATGGCAAATTCCGAG
GGCTTATTTTCGCTGGTTGCTCGCAAACTGAGTCGTCCTCTTCTCGAGCACCACCACCACCACCACTGA

MESGFTSKDTYLSHFNPRDYLEKYYKFGSRHSAESQILKHLLKNLFKIFCLDGVKGDLLIDIGSGPTIYQLLSGC
ESFKEIVVTDYSDQNLQELEKWLKKEPEAFDWSPVVTYVCDLEGNRVKGPEKEEKLRQAVKQVLKCDVTQSQPLG
AVPLPPADCVLSTLCLHAACPDLPTYCRALRNLGSLLKPGGFLVIMDHLKCSYYMIGEQKFSSLPLGREAVEAAV
KEAGYTIEWFEVISQSFSSTMANSEGLFSLVARKLSRPLLEHHHHHH

vS11 (*hsa*-N-NMT_A74G, D167H, A198H, S201C, Y242P, N249S)

ATGGAGAGTGGATTTACGAGTAAGGACACTTATTTGAGTCATTTTAACCCGCGCGACTACTTAGAAAAATACTAC
AAGTTTGGAAGCCGCCATTCAGCAGAATCACAAATTCTGAAACACCTTTTAAAGAACCTGTTTAAAATCTTCTGT
TTAGATGGGGTCAAAGGAGATTTGTTGATTGACATCGGATCAGGTCCCACAATTTACCAACTTCTGTCCGGGTGT
GAGAGTTTTAAGGAGATCGTCGTGACGGACTATTCAGATCAAAATCTGCAAGAACTGGAGAAATGGTTAAAGAAA
GAACCAGAGGCGTTCGATTGGAGCCCAGTTGTGACCTATGTCTGTGATCTTGAAGGAAACCGCGTAAAGGGTCCT
GAAAAGGAGGAAAAGCTGCGCCAAGCCGTAAAGCAAGTATTAAAATGTGATGTTACTCAAAGCCAACCATTAGGA
GCCGTCCCACTGCCCCCGGCGGATTGTGTATTGTCAACCCTTTGCTTGCACGCAGCTTGTCCTGACTTGCCTACA
TATTGTCGTGCATTACGCAACTTAGGAAGTTTGCTTAAACCAGGAGGGTTTCTGGTCATCATGGATCACTTGAAG
TGCTCCTACTACATGATTGGGGAACAGAAGTTCTCATCTTTACCTTTGGGCCGTGAAGCCGTAGAAGCGGCCGTA
AAGGAGGCGGGGTACACCATTGAATGGTTCGAGGTTATTTCACAGTCCCCGAGCTCCACAATGGCAAATTCCGAG
GGCTTATTTTCGCTGGTTGCTCGCAAACTGAGTCGTCCTCTTCTCGAGCACCACCACCACCACCACTGA

MESGFTSKDTYLSHFNPRDYLEKYYKFGSRHSAESQILKHLLKNLFKIFCLDGVKGDLLIDIGSGPTIYQLLSGC
ESFKEIVVTDYSDQNLQELEKWLKKEPEAFDWSPVVTYVCDLEGNRVKGPEKEEKLRQAVKQVLKCDVTQSQPLG
AVPLPPADCVLSTLCLHAACPDLPTYCRALRNLGSLLKPGGFLVIMDHLKCSYYMIGEQKFSSLPLGREAVEAAV
KEAGYTIEWFEVISQSPSSTMANSEGLFSLVARKLSRPLLEHHHHHH

vS12 (*hsa*-N-NMT_A74G, D167H, A198H, S201C, Y242P, A247T, N249S)

ATGGAGAGTGGATTTACGAGTAAGGACACTTATTTGAGTCATTTTAACCCGCGCGACTACTTAGAAAAATACTAC
AAGTTTGGAAGCCGCCATTCAGCAGAATCACAAATTCTGAAACACCTTTTAAAGAACCTGTTTAAAATCTTCTGT
TTAGATGGGGTCAAAGGAGATTTGTTGATTGACATCGGATCAGGTCCCACAATTTACCAACTTCTGTCCGGGTGT
GAGAGTTTTAAGGAGATCGTCGTGACGGACTATTCAGATCAAAATCTGCAAGAACTGGAGAAATGGTTAAAGAAA
GAACCAGAGGCGTTCGATTGGAGCCCAGTTGTGACCTATGTCTGTGATCTTGAAGGAAACCGCGTAAAGGGTCCT
GAAAAGGAGGAAAAGCTGCGCCAAGCCGTAAAGCAAGTATTAAAATGTGATGTTACTCAAAGCCAACCATTAGGA
GCCGTCCCACTGCCCCCGGCGGATTGTGTATTGTCAACCCTTTGCTTGCACGCAGCTTGTCCTGACTTGCCTACA
TATTGTCGTGCATTACGCAACTTAGGAAGTTTGCTTAAACCAGGAGGGTTTCTGGTCATCATGGATCACTTGAAG
TGCTCCTACTACATGATTGGGGAACAGAAGTTCTCATCTTTACCTTTGGGCCGTGAAGCCGTAGAAGCGGCCGTA
AAGGAGGCGGGGTACACCATTGAATGGTTCGAGGTTATTTCACAGTCCCCGAGCTCCACAATGACAAATTCCGAG
GGCTTATTTTCGCTGGTTGCTCGCAAACTGAGTCGTCCTCTTCTCGAGCACCACCACCACCACCACTGA

MESGFTSKDTYLSHFNPRDYLEKYYKFGSRHSAESQILKHLLKNLFKIFCLDGVKGDLLIDIGSGPTIYQLLSGC
ESFKEIVVTDYSDQNLQELEKWLKKEPEAFDWSPVVTYVCDLEGNRVKGPEKEEKLRQAVKQVLKCDVTQSQPLG
AVPLPPADCVLSTLCLHAACPDLPTYCRALRNLGSLLKPGGFLVIMDHLKCSYYMIGEQKFSSLPLGREAVEAAV
KEAGYTIEWFEVISQSPSSTMTNSEGLFSLVARKLSRPLLEHHHHHH

vS13 (*hsa*-N-NMT_A74G, D167H, A198H, S201C, Y242F, A247T, N249S)

ATGGAGAGTGGATTTACGAGTAAGGACACTTATTTGAGTCATTTTAACCCGCGCGACTACTTAGAAAAATACTAC
AAGTTTGGAAGCCGCCATTCAGCAGAATCACAAATTCTGAAACACCTTTTAAAGAACCTGTTTAAAATCTTCTGT
TTAGATGGGGTCAAAGGAGATTTGTTGATTGACATCGGATCAGGTCCCACAATTTACCAACTTCTGTCCGGGTGT
GAGAGTTTTAAGGAGATCGTCGTGACGGACTATTCAGATCAAAATCTGCAAGAACTGGAGAAATGGTTAAAGAAA
GAACCAGAGGCGTTCGATTGGAGCCCAGTTGTGACCTATGTCTGTGATCTTGAAGGAAACCGCGTAAAGGGTCCT
GAAAAGGAGGAAAAGCTGCGCCAAGCCGTAAAGCAAGTATTAAAATGTGATGTTACTCAAAGCCAACCATTAGGA
GCCGTCCCACTGCCCCCGGCGGATTGTGTATTGTCAACCCTTTGCTTGCACGCAGCTTGTCCTGACTTGCCTACA
TATTGTCGTGCATTACGCAACTTAGGAAGTTTGCTTAAACCAGGAGGGTTTCTGGTCATCATGGATCACTTGAAG
TGCTCCTACTACATGATTGGGGAACAGAAGTTCTCATCTTTACCTTTGGGCCGTGAAGCCGTAGAAGCGGCCGTA
AAGGAGGCGGGGTACACCATTGAATGGTTCGAGGTTATTTCACAGTCCTTCAGCTCCACAATGACAAATTCCGAG
GGCTTATTTTCGCTGGTTGCTCGCAAACTGAGTCGTCCTCTTCTCGAGCACCACCACCACCACCACTGA

MESGFTSKDTYLSHFNPRDYLEKYYKFGSRHSAESQILKHLLKNLFKIFCLDGVKGDLLIDIGSGPTIYQLLSGC
ESFKEIVVTDYSDQNLQELEKWLKKEPEAFDWSPVVTYVCDLEGNRVKGPEKEEKLRQAVKQVLKCDVTQSQPLG
AVPLPPADCVLSTLCLHAACPDLPTYCRALRNLGSLLKPGGFLVIMDHLKCSYYMIGEQKFSSLPLGREAVEAAV
KEAGYTIEWFEVISQSFSSTMTNSEGLFSLVARKLSRPLLEHHHHHH

### vS14 (*hsa*-N-NMT_A74G, D167H, A198Y, S201C, Y242P, A247C, N249G)

ATGGAGAGTGGATTTACGAGTAAGGACACTTATTTGAGTCATTTTAACCCGCGCGACTACTTAGAAAAATACTAC
AAGTTTGGAAGCCGCCATTCAGCAGAATCACAAATTCTGAAACACCTTTTAAAGAACCTGTTTAAAATCTTCTGT
TTAGATGGGGTCAAAGGAGATTTGTTGATTGACATCGGATCAGGTCCCACAATTTACCAACTTCTGTCCGGGTGT
GAGAGTTTTAAGGAGATCGTCGTGACGGACTATTCAGATCAAAATCTGCAAGAACTGGAGAAATGGTTAAAGAAA
GAACCAGAGGCGTTCGATTGGAGCCCAGTTGTGACCTATGTCTGTGATCTTGAAGGAAACCGCGTAAAGGGTCCT
GAAAAGGAGGAAAAGCTGCGCCAAGCCGTAAAGCAAGTATTAAAATGTGATGTTACTCAAAGCCAACCATTAGGA
GCCGTCCCACTGCCCCCGGCGGATTGTGTATTGTCAACCCTTTGCTTGCACGCAGCTTGTCCTGACTTGCCTACA
TATTGTCGTGCATTACGCAACTTAGGAAGTTTGCTTAAACCAGGAGGGTTTCTGGTCATCATGGATTATTTGAAG
TGCTCCTACTACATGATTGGGGAACAGAAGTTCTCATCTTTACCTTTGGGCCGTGAAGCCGTAGAAGCGGCCGTA
AAGGAGGCGGGGTACACCATTGAATGGTTCGAGGTTATTTCACAGTCCCCGAGCTCCACAATGTGTAATGGGGAG
GGCTTATTTTCGCTGGTTGCTCGCAAACTGAGTCGTCCTCTTCTCGAGCACCACCACCACCACCACTGA

MESGFTSKDTYLSHFNPRDYLEKYYKFGSRHSAESQILKHLLKNLFKIFCLDGVKGDLLIDIGSGPTIYQLLSGC
ESFKEIVVTDYSDQNLQELEKWLKKEPEAFDWSPVVTYVCDLEGNRVKGPEKEEKLRQAVKQVLKCDVTQSQPLG
AVPLPPADCVLSTLCLHAACPDLPTYCRALRNLGSLLKPGGFLVIMDYLKCSYYMIGEQKFSSLPLGREAVEAAV
KEAGYTIEWFEVISQSPSSTMCNGEGLFSLVARKLSRPLLEHHHHHH

### vS15 (*hsa*-N-NMT_A74G, D167H, A198Y, S201C, Y242P, N249G)

ATGGAGAGTGGATTTACGAGTAAGGACACTTATTTGAGTCATTTTAACCCGCGCGACTACTTAGAAAAATACTAC
AAGTTTGGAAGCCGCCATTCAGCAGAATCACAAATTCTGAAACACCTTTTAAAGAACCTGTTTAAAATCTTCTGT
TTAGATGGGGTCAAAGGAGATTTGTTGATTGACATCGGATCAGGTCCCACAATTTACCAACTTCTGTCCGGGTGT
GAGAGTTTTAAGGAGATCGTCGTGACGGACTATTCAGATCAAAATCTGCAAGAACTGGAGAAATGGTTAAAGAAA
GAACCAGAGGCGTTCGATTGGAGCCCAGTTGTGACCTATGTCTGTGATCTTGAAGGAAACCGCGTAAAGGGTCCT
GAAAAGGAGGAAAAGCTGCGCCAAGCCGTAAAGCAAGTATTAAAATGTGATGTTACTCAAAGCCAACCATTAGGA
GCCGTCCCACTGCCCCCGGCGGATTGTGTATTGTCAACCCTTTGCTTGCACGCAGCTTGTCCTGACTTGCCTACA
TATTGTCGTGCATTACGCAACTTAGGAAGTTTGCTTAAACCAGGAGGGTTTCTGGTCATCATGGATTATTTGAAG
TGCTCCTACTACATGATTGGGGAACAGAAGTTCTCATCTTTACCTTTGGGCCGTGAAGCCGTAGAAGCGGCCGTA
AAGGAGGCGGGGTACACCATTGAATGGTTCGAGGTTATTTCACAGTCCCCGAGCTCCACAATGGCAAATGGGGAG
GGCTTATTTTCGCTGGTTGCTCGCAAACTGAGTCGTCCTCTTCTCGAGCACCACCACCACCACCACTGA

MESGFTSKDTYLSHFNPRDYLEKYYKFGSRHSAESQILKHLLKNLFKIFCLDGVKGDLLIDIGSGPTIYQLLSGC
ESFKEIVVTDYSDQNLQELEKWLKKEPEAFDWSPVVTYVCDLEGNRVKGPEKEEKLRQAVKQVLKCDVTQSQPLG
AVPLPPADCVLSTLCLHAACPDLPTYCRALRNLGSLLKPGGFLVIMDYLKCSYYMIGEQKFSSLPLGREAVEAAV
KEAGYTIEWFEVISQSPSSTMANGEGLFSLVARKLSRPLLEHHHHHH

### vS16 (*hsa*-N-NMT_V82F, D167S, A198H, S201C, Y242P, N249S)

ATGGAGAGTGGATTTACGAGTAAGGACACTTATTTGAGTCATTTTAACCCGCGCGACTACTTAGAAAAATACTAC
AAGTTTGGAAGCCGCCATTCAGCAGAATCACAAATTCTGAAACACCTTTTAAAGAACCTGTTTAAAATCTTCTGT
TTAGATGGGGTCAAAGGAGATTTGTTGATTGACATCGGATCAGGTCCCACAATTTACCAACTTCTGTCCGCTTGT
GAGAGTTTTAAGGAGATCTTCGTGACGGACTATTCAGATCAAAATCTGCAAGAACTGGAGAAATGGTTAAAGAAA
GAACCAGAGGCGTTCGATTGGAGCCCAGTTGTGACCTATGTCTGTGATCTTGAAGGAAACCGCGTAAAGGGTCCT
GAAAAGGAGGAAAAGCTGCGCCAAGCCGTAAAGCAAGTATTAAAATGTGATGTTACTCAAAGCCAACCATTAGGA
GCCGTCCCACTGCCCCCGGCGGATTGTGTATTGTCAACCCTTTGCTTGTCGGCAGCTTGTCCTGACTTGCCTACA
TATTGTCGTGCATTACGCAACTTAGGAAGTTTGCTTAAACCAGGAGGGTTTCTGGTCATCATGGATCACTTGAAG
TGCTCCTACTACATGATTGGGGAACAGAAGTTCTCATCTTTACCTTTGGGCCGTGAAGCCGTAGAAGCGGCCGTA
AAGGAGGCGGGGTACACCATTGAATGGTTCGAGGTTATTTCACAGTCCCCGAGCTCCACAATGGCAAATTCCGAG
GGCTTATTTTCGCTGGTTGCTCGCAAACTGAGTCGTCCTCTTCTCGAGCACCACCACCACCACCACTGA

MESGFTSKDTYLSHFNPRDYLEKYYKFGSRHSAESQILKHLLKNLFKIFCLDGVKGDLLIDIGSGPTIYQLLSAC
ESFKEIFVTDYSDQNLQELEKWLKKEPEAFDWSPVVTYVCDLEGNRVKGPEKEEKLRQAVKQVLKCDVTQSQPLG
AVPLPPADCVLSTLCLSAACPDLPTYCRALRNLGSLLKPGGFLVIMDHLKCSYYMIGEQKFSSLPLGREAVEAAV
KEAGYTIEWFEVISQSPSSTMANSEGLFSLVARKLSRPLLEHHHHHH

vS17 (*hsa*-N-NMT_A134V, D167S, A198H, S201C, Y242F, N249S)
ATGGAGAGTGGATTTACGAGTAAGGACACTTATTTGAGTCATTTTAACCCGCGCGACTACTTAGAAAAATACTAC
AAGTTTGGAAGCCGCCATTCAGCAGAATCACAAATTCTGAAACACCTTTTAAAGAACCTGTTTAAAATCTTCTGT
TTAGATGGGGTCAAAGGAGATTTGTTGATTGACATCGGATCAGGTCCCACAATTTACCAACTTCTGTCCGCTTGT
GAGAGTTTTAAGGAGATCGTCGTGACGGACTATTCAGATCAAAATCTGCAAGAACTGGAGAAATGGTTAAAGAAA
GAACCAGAGGCGTTCGATTGGAGCCCAGTTGTGACCTATGTCTGTGATCTTGAAGGAAACCGCGTAAAGGGTCCT
GAAAAGGAGGAAAAGCTGCGCCAAGTGGTAAAGCAAGTATTAAAATGTGATGTTACTCAAAGCCAACCATTAGGA
GCCGTCCCACTGCCCCCGGCGGATTGTGTATTGTCAACCCTTTGCTTGTCGGCAGCTTGTCCTGACTTGCCTACA
TATTGTCGTGCATTACGCAACTTAGGAAGTTTGCTTAAACCAGGAGGGTTTCTGGTCATCATGGATCACTTGAAG
TGCTCCTACTACATGATTGGGGAACAGAAGTTCTCATCTTTACCTTTGGGCCGTGAAGCCGTAGAAGCGGCCGTA
AAGGAGGCGGGGTACACCATTGAATGGTTCGAGGTTATTTCACAGTCCTTCAGCTCCACAATGGCAAATTCCGAG
GGCTTATTTTCGCTGGTTGCTCGCAAACTGAGTCGTCCTCTTCTCGAGCACCACCACCACCACCACTGA

MESGFTSKDTYLSHFNPRDYLEKYYKFGSRHSAESQILKHLLKNLFKIFCLDGVKGDLLIDIGSGPTIYQLLSAC
ESFKEIVVTDYSDQNLQELEKWLKKEPEAFDWSPVVTYVCDLEGNRVKGPEKEEKLRQVVKQVLKCDVTQSQPLG
AVPLPPADCVLSTLCLSAACPDLPTYCRALRNLGSLLKPGGFLVIMDHLKCSYYMIGEQKFSSLPLGREAVEAAV
KEAGYTIEWFEVISQSFSSTMANSEGLFSLVARKLSRPLLEHHHHHH

vS18 (*hsa*-N-NMT_A134V, D167S, A198H, S201C, Y242P, N249S)
ATGGAGAGTGGATTTACGAGTAAGGACACTTATTTGAGTCATTTTAACCCGCGCGACTACTTAGAAAAATACTAC
AAGTTTGGAAGCCGCCATTCAGCAGAATCACAAATTCTGAAACACCTTTTAAAGAACCTGTTTAAAATCTTCTGT
TTAGATGGGGTCAAAGGAGATTTGTTGATTGACATCGGATCAGGTCCCACAATTTACCAACTTCTGTCCGCTTGT
GAGAGTTTTAAGGAGATCGTCGTGACGGACTATTCAGATCAAAATCTGCAAGAACTGGAGAAATGGTTAAAGAAA
GAACCAGAGGCGTTCGATTGGAGCCCAGTTGTGACCTATGTCTGTGATCTTGAAGGAAACCGCGTAAAGGGTCCT
GAAAAGGAGGAAAAGCTGCGCCAAGTGGTAAAGCAAGTATTAAAATGTGATGTTACTCAAAGCCAACCATTAGGA
GCCGTCCCACTGCCCCCGGCGGATTGTGTATTGTCAACCCTTTGCTTGTCGGCAGCTTGTCCTGACTTGCCTACA
TATTGTCGTGCATTACGCAACTTAGGAAGTTTGCTTAAACCAGGAGGGTTTCTGGTCATCATGGATCACTTGAAG
TGCTCCTACTACATGATTGGGGAACAGAAGTTCTCATCTTTACCTTTGGGCCGTGAAGCCGTAGAAGCGGCCGTA
AAGGAGGCGGGGTACACCATTGAATGGTTCGAGGTTATTTCACAGTCCCCGAGCTCCACAATGGCAAATTCCGAG
GGCTTATTTTCGCTGGTTGCTCGCAAACTGAGTCGTCCTCTTCTCGAGCACCACCACCACCACCACTGA

MESGFTSKDTYLSHFNPRDYLEKYYKFGSRHSAESQILKHLLKNLFKIFCLDGVKGDLLIDIGSGPTIYQLLSAC
ESFKEIVVTDYSDQNLQELEKWLKKEPEAFDWSPVVTYVCDLEGNRVKGPEKEEKLRQVVKQVLKCDVTQSQPLG
AVPLPPADCVLSTLCLSAACPDLPTYCRALRNLGSLLKPGGFLVIMDHLKCSYYMIGEQKFSSLPLGREAVEAAV
KEAGYTIEWFEVISQSPSSTMANSEGLFSLVARKLSRPLLEHHHHHH

vS19 (*hsa*-N-NMT_A134V, D167S, A198Y, S201C, Y242P, N249G)
ATGGAGAGTGGATTTACGAGTAAGGACACTTATTTGAGTCATTTTAACCCGCGCGACTACTTAGAAAAATACTAC
AAGTTTGGAAGCCGCCATTCAGCAGAATCACAAATTCTGAAACACCTTTTAAAGAACCTGTTTAAAATCTTCTGT
TTAGATGGGGTCAAAGGAGATTTGTTGATTGACATCGGATCAGGTCCCACAATTTACCAACTTCTGTCCGCTTGT
GAGAGTTTTAAGGAGATCGTCGTGACGGACTATTCAGATCAAAATCTGCAAGAACTGGAGAAATGGTTAAAGAAA
GAACCAGAGGCGTTCGATTGGAGCCCAGTTGTGACCTATGTCTGTGATCTTGAAGGAAACCGCGTAAAGGGTCCT
GAAAAGGAGGAAAAGCTGCGCCAAGTGGTAAAGCAAGTATTAAAATGTGATGTTACTCAAAGCCAACCATTAGGA
GCCGTCCCACTGCCCCCGGCGGATTGTGTATTGTCAACCCTTTGCTTGTCGGCAGCTTGTCCTGACTTGCCTACA
TATTGTCGTGCATTACGCAACTTAGGAAGTTTGCTTAAACCAGGAGGGTTTCTGGTCATCATGGATTATTTGAAG
TGCTCCTACTACATGATTGGGGAACAGAAGTTCTCATCTTTACCTTTGGGCCGTGAAGCCGTAGAAGCGGCCGTA
AAGGAGGCGGGGTACACCATTGAATGGTTCGAGGTTATTTCACAGTCCCCGAGCTCCACAATGGCAAATGGGGAG
GGCTTATTTTCGCTGGTTGCTCGCAAACTGAGTCGTCCTCTTCTCGAGCACCACCACCACCACCACTGA

MESGFTSKDTYLSHFNPRDYLEKYYKFGSRHSAESQILKHLLKNLFKIFCLDGVKGDLLIDIGSGPTIYQLLSAC
ESFKEIVVTDYSDQNLQELEKWLKKEPEAFDWSPVVTYVCDLEGNRVKGPEKEEKLRQVVKQVLKCDVTQSQPLG
AVPLPPADCVLSTLCLSAACPDLPTYCRALRNLGSLLKPGGFLVIMDYLKCSYYMIGEQKFSSLPLGREAVEAAV
KEAGYTIEWFEVISQSPSSTMANGEGLFSLVARKLSRPLLEHHHHHH

vS20 (*hsa*-N-NMT_A134V, D167H, P171L, A198Y, S201C, Y242F, A247C, N249G)
ATGGAGAGTGGATTTACGAGTAAGGACACTTATTTGAGTCATTTTAACCCGCGCGACTACTTAGAAAAATACTAC
AAGTTTGGAAGCCGCCATTCAGCAGAATCACAAATTCTGAAACACCTTTTAAAGAACCTGTTTAAAATCTTCTGT
TTAGATGGGGTCAAAGGAGATTTGTTGATTGACATCGGATCAGGTCCCACAATTTACCAACTTCTGTCCGCTTGT
GAGAGTTTTAAGGAGATCGTCGTGACGGACTATTCAGATCAAAATCTGCAAGAACTGGAGAAATGGTTAAAGAAA
GAACCAGAGGCGTTCGATTGGAGCCCAGTTGTGACCTATGTCTGTGATCTTGAAGGAAACCGCGTAAAGGGTCCT
GAAAAGGAGGAAAAGCTGCGCCAAGTGGTAAAGCAAGTATTAAAATGTGATGTTACTCAAAGCCAACCATTAGGA
GCCGTCCCACTGCCCCCGGCGGATTGTGTATTGTCAACCCTTTGCTTGCACGCAGCTTGTCTTGACTTGCCTACA
TATTGTCGTGCATTACGCAACTTAGGAAGTTTGCTTAAACCAGGAGGGTTTCTGGTCATCATGGATTATTTGAAG
TGCTCCTACTACATGATTGGGGAACAGAAGTTCTCATCTTTACCTTTGGGCCGTGAAGCCGTAGAAGCGGCCGTA
AAGGAGGCGGGGTACACCATTGAATGGTTCGAGGTTATTTCACAGTCCTTCAGCTCCACAATGTGTAATGGGGAG
GGCTTATTTTCGCTGGTTGCTCGCAAACTGAGTCGTCCTCTTCTCGAGCACCACCACCACCACCACTGA

MESGFTSKDTYLSHFNPRDYLEKYYKFGSRHSAESQILKHLLKNLFKIFCLDGVKGDLLIDIGSGPTIYQLLSAC
ESFKEIVVTDYSDQNLQELEKWLKKEPEAFDWSPVVTYVCDLEGNRVKGPEKEEKLRQVVKQVLKCDVTQSQPLG
AVPLPPADCVLSTLCLHAACLDLPTYCRALRNLGSLLKPGGFLVIMDYLKCSYYMIGEQKFSSLPLGREAVEAAV
KEAGYTIEWFEVISQSFSSTMCNGEGLFSLVARKLSRPLLEHHHHHH

vS21 (*hsa*-N-NMT_A134V, D167H, A198H, S201C, Y242F, N249S)
ATGGAGAGTGGATTTACGAGTAAGGACACTTATTTGAGTCATTTTAACCCGCGCGACTACTTAGAAAAATACTAC
AAGTTTGGAAGCCGCCATTCAGCAGAATCACAAATTCTGAAACACCTTTTAAAGAACCTGTTTAAAATCTTCTGT
TTAGATGGGGTCAAAGGAGATTTGTTGATTGACATCGGATCAGGTCCCACAATTTACCAACTTCTGTCCGCTTGT
GAGAGTTTTAAGGAGATCGTCGTGACGGACTATTCAGATCAAAATCTGCAAGAACTGGAGAAATGGTTAAAGAAA
GAACCAGAGGCGTTCGATTGGAGCCCAGTTGTGACCTATGTCTGTGATCTTGAAGGAAACCGCGTAAAGGGTCCT
GAAAAGGAGGAAAAGCTGCGCCAAGTGGTAAAGCAAGTATTAAAATGTGATGTTACTCAAAGCCAACCATTAGGA
GCCGTCCCACTGCCCCCGGCGGATTGTGTATTGTCAACCCTTTGCTTGCACGCAGCTTGTCCTGACTTGCCTACA
TATTGTCGTGCATTACGCAACTTAGGAAGTTTGCTTAAACCAGGAGGGTTTCTGGTCATCATGGATCACTTGAAG
TGCTCCTACTACATGATTGGGGAACAGAAGTTCTCATCTTTACCTTTGGGCCGTGAAGCCGTAGAAGCGGCCGTA
AAGGAGGCGGGGTACACCATTGAATGGTTCGAGGTTATTTCACAGTCCTTCAGCTCCACAATGGCAAATTCCGAG
GGCTTATTTTCGCTGGTTGCTCGCAAACTGAGTCGTCCTCTTCTCGAGCACCACCACCACCACCACTGA

MESGFTSKDTYLSHFNPRDYLEKYYKFGSRHSAESQILKHLLKNLFKIFCLDGVKGDLLIDIGSGPTIYQLLSAC
ESFKEIVVTDYSDQNLQELEKWLKKEPEAFDWSPVVTYVCDLEGNRVKGPEKEEKLRQVVKQVLKCDVTQSQPLG
AVPLPPADCVLSTLCLHAACPDLPTYCRALRNLGSLLKPGGFLVIMDHLKCSYYMIGEQKFSSLPLGREAVEAAV
KEAGYTIEWFEVISQSFSSTMANSEGLFSLVARKLSRPLLEHHHHHH

vS22 (*hsa*-N-NMT_A134V, D167H, A198H, S201C, Y242P, N249S)
ATGGAGAGTGGATTTACGAGTAAGGACACTTATTTGAGTCATTTTAACCCGCGCGACTACTTAGAAAAATACTAC
AAGTTTGGAAGCCGCCATTCAGCAGAATCACAAATTCTGAAACACCTTTTAAAGAACCTGTTTAAAATCTTCTGT
TTAGATGGGGTCAAAGGAGATTTGTTGATTGACATCGGATCAGGTCCCACAATTTACCAACTTCTGTCCGCTTGT
GAGAGTTTTAAGGAGATCGTCGTGACGGACTATTCAGATCAAAATCTGCAAGAACTGGAGAAATGGTTAAAGAAA
GAACCAGAGGCGTTCGATTGGAGCCCAGTTGTGACCTATGTCTGTGATCTTGAAGGAAACCGCGTAAAGGGTCCC
GAAAAGGAGGAAAAGCTGCGCCAAGTGGTAAAGCAAGTATTAAAATGTGATGTTACTCAAAGCCAACCATTAGGA
GCCGTCCCACTGCCCCCGGCGGATTGTGTATTGTCAACCCTTTGCTTGCACGCAGCTTGTCCTGACTTGCCTACA
TATTGTCGTGCATTACGCAACTTAGGAAGTTTGCTTAAACCAGGAGGGTTTCTGGTCATCATGGATCACTTGAAG
TGCTCCTACTACATGATTGGGGAACAGAAGTTCTCATCTTTACCTTTGGGCCGTGAAGCCGTAGAAGCGGCCGTA
AAGGAGGCGGGGTACACCATTGAATGGTTCGAGGTTATTTCACAGTCCCCGAGCTCCACAATGGCAAATTCCGAG
GGCTTATTTTCGCTGGTTGCTCGCAAACTGAGTCGTCCTCTTCTCGAGCACCACCACCACCACCACTGA

MESGFTSKDTYLSHFNPRDYLEKYYKFGSRHSAESQILKHLLKNLFKIFCLDGVKGDLLIDIGSGPTIYQLLSAC
ESFKEIVVTDYSDQNLQELEKWLKKEPEAFDWSPVVTYVCDLEGNRVKGPEKEEKLRQVVKQVLKCDVTQSQPLG
AVPLPPADCVLSTLCLHAACPDLPTYCRALRNLGSLLKPGGFLVIMDHLKCSYYMIGEQKFSSLPLGREAVEAAV
KEAGYTIEWFEVISQSPSSTMANSEGLFSLVARKLSRPLLEHHHHHH

vS23 (*hsa*-N-NMT_A134V, D167H, A198H, S201C, Y242P, A247T, N249S)
ATGGAGAGTGGATTTACGAGTAAGGACACTTATTTGAGTCATTTTAACCCGCGCGACTACTTAGAAAAATACTAC
AAGTTTGGAAGCCGCCATTCAGCAGAATCACAAATTCTGAAACACCTTTTAAAGAACCTGTTTAAAATCTTCTGT
TTAGATGGGGTCAAAGGAGATTTGTTGATTGACATCGGATCAGGTCCCACAATTTACCAACTTCTGTCCGCTTGT
GAGAGTTTTAAGGAGATCGTCGTGACGGACTATTCAGATCAAAATCTGCAAGAACTGGAGAAATGGTTAAAGAAA
GAACCAGAGGCGTTCGATTGGAGCCCAGTTGTGACCTATGTCTGTGATCTTGAAGGAAACCGCGTAAAGGGTCCT
GAAAAGGAGGAAAAGCTGCGCCAAGTGGTAAAGCAAGTATTAAAATGTGATGTTACTCAAAGCCAACCATTAGGA
GCCGTCCCACTGCCCCCGGCGGATTGTGTATTGTCAACCCTTTGCTTGCACGCAGCTTGTCCTGACTTGCCTACA
TATTGTCGTGCATTACGCAACTTAGGAAGTTTGCTTAAACCAGGAGGGTTTCTGGTCATCATGGATCACTTGAAG
TGCTCCTACTACATGATTGGGGAACAGAAGTTCTCATCTTTACCTTTGGGCCGTGAAGCCGTAGAAGCGGCCGTA
AAGGAGGCGGGGTACACCATTGAATGGTTCGAGGTTATTTCACAGTCCCCGAGCTCCACAATGACAAATTCCGAG
GGCTTATTTTCGCTGGTTGCTCGCAAACTGAGTCGTCCTCTTCTCGAGCACCACCACCACCACCACTGA

MESGFTSKDTYLSHFNPRDYLEKYYKFGSRHSAESQILKHLLKNLFKIFCLDGVKGDLLIDIGSGPTIYQLLSAC
ESFKEIVVTDYSDQNLQELEKWLKKEPEAFDWSPVVTYVCDLEGNRVKGPEKEEKLRQVVKQVLKCDVTQSQPLG
AVPLPPADCVLSTLCLHAACPDLPTYCRALRNLGSLLKPGGFLVIMDHLKCSYYMIGEQKFSSLPLGREAVEAAV
KEAGYTIEWFEVISQSPSSTMTNSEGLFSLVARKLSRPLLEHHHHHH

vS24 (*hsa*-N-NMT_A134V, D167H, A198H, S201C, Y242F, A247T, N249S)
ATGGAGAGTGGATTTACGAGTAAGGACACTTATTTGAGTCATTTTAACCCGCGCGACTACTTAGAAAAATACTAC
AAGTTTGGAAGCCGCCATTCAGCAGAATCACAAATTCTGAAACACCTTTTAAAGAACCTGTTTAAAATCTTCTGT
TTAGATGGGGTCAAAGGAGATTTGTTGATTGACATCGGATCAGGTCCCACAATTTACCAACTTCTGTCCGCTTGT
GAGAGTTTTAAGGAGATCGTCGTGACGGACTATTCAGATCAAAATCTGCAAGAACTGGAGAAATGGTTAAAGAAA
GAACCAGAGGCGTTCGATTGGAGCCCAGTTGTGACCTATGTCTGTGATCTTGAAGGAAACCGCGTAAAGGGTCCT
GAAAAGGAGGAAAAGCTGCGCCAAGTGGTAAAGCAAGTATTAAAATGTGATGTTACTCAAAGCCAACCATTAGGA
GCCGTCCCACTGCCCCCGGCGGATTGTGTATTGTCAACCCTTTGCTTGCACGCAGCTTGTCCTGACTTGCCTACA
TATTGTCGTGCATTACGCAACTTAGGAAGTTTGCTTAAACCAGGAGGGTTTCTGGTCATCATGGATCACTTGAAG
TGCTCCTACTACATGATTGGGGAACAGAAGTTCTCATCTTTACCTTTGGGCCGTGAAGCCGTAGAAGCGGCCGTA
AAGGAGGCGGGGTACACCATTGAATGGTTCGAGGTTATTTCACAGTCCTTCAGCTCCACAATGACAAATTCCGAG
GGCTTATTTTCGCTGGTTGCTCGCAAACTGAGTCGTCCTCTTCTCGAGCACCACCACCACCACCACTGA

MESGFTSKDTYLSHFNPRDYLEKYYKFGSRHSAESQILKHLLKNLFKIFCLDGVKGDLLIDIGSGPTIYQLLSAC
ESFKEIVVTDYSDQNLQELEKWLKKEPEAFDWSPVVTYVCDLEGNRVKGPEKEEKLRQVVKQVLKCDVTQSQPLG
AVPLPPADCVLSTLCLHAACPDLPTYCRALRNLGSLLKPGGFLVIMDHLKCSYYMIGEQKFSSLPLGREAVEAAV
KEAGYTIEWFEVISQSFSSTMTNSEGLFSLVARKLSRPLLEHHHHHH

vS25 (*hsa*-N-NMT_A134V, D167H, A198Y, S201C, Y242P, N249S)
ATGGAGAGTGGATTTACGAGTAAGGACACTTATTTGAGTCATTTTAACCCGCGCGACTACTTAGAAAAATACTAC
AAGTTTGGAAGCCGCCATTCAGCAGAATCACAAATTCTGAAACACCTTTTAAAGAACCTGTTTAAAATCTTCTGT
TTAGATGGGGTCAAAGGAGATTTGTTGATTGACATCGGATCAGGTCCCACAATTTACCAACTTCTGTCCGCTTGT
GAGAGTTTTAAGGAGATCGTCGTGACGGACTATTCAGATCAAAATCTGCAAGAACTGGAGAAATGGTTAAAGAAA
GAACCAGAGGCGTTCGATTGGAGCCCAGTTGTGACCTATGTCTGTGATCTTGAAGGAAACCGCGTAAAGGGTCCT
GAAAAGGAGGAAAAGCTGCGCCAAGTGGTAAAGCAAGTATTAAAATGTGATGTTACTCAAAGCCAACCATTAGGA
GCCGTCCCACTGCCCCCGGCGGATTGTGTATTGTCAACCCTTTGCTTGCACGCAGCTTGTCCTGACTTGCCTACA
TATTGTCGTGCATTACGCAACTTAGGAAGTTTGCTTAAACCAGGAGGGTTTCTGGTCATCATGGATTATTTGAAG
TGCTCCTACTACATGATTGGGGAACAGAAGTTCTCATCTTTACCTTTGGGCCGTGAAGCCGTAGAAGCGGCCGTA
AAGGAGGCGGGGTACACCATTGAATGGTTCGAGGTTATTTCACAGTCCCCGAGCTCCACAATGGCAAATTCCGAG
GGCTTATTTTCGCTGGTTGCTCGCAAACTGAGTCGTCCTCTTCTCGAGCACCACCACCACCACCACTGA

MESGFTSKDTYLSHFNPRDYLEKYYKFGSRHSAESQILKHLLKNLFKIFCLDGVKGDLLIDIGSGPTIYQLLSAC
ESFKEIVVTDYSDQNLQELEKWLKKEPEAFDWSPVVTYVCDLEGNRVKGPEKEEKLRQVVKQVLKCDVTQSQPLG
AVPLPPADCVLSTLCLHAACPDLPTYCRALRNLGSLLKPGGFLVIMDYLKCSYYMIGEQKFSSLPLGREAVEAAV
KEAGYTIEWFEVISQSPSSTMANSEGLFSLVARKLSRPLLEHHHHHH

vS26 (*hsa*-N-NMT_A134V, D167H, A198Y, S201C, Y242P, A247C, N249S)
ATGGAGAGTGGATTTACGAGTAAGGACACTTATTTGAGTCATTTTAACCCGCGCGACTACTTAGAAAAATACTAC
AAGTTTGGAAGCCGCCATTCAGCAGAATCACAAATTCTGAAACACCTTTTAAAGAACCTGTTTAAAATCTTCTGT
TTAGATGGGGTCAAAGGAGATTTGTTGATTGACATCGGATCAGGTCCCACAATTTACCAACTTCTGTCCGCTTGT
GAGAGTTTTAAGGAGATCGTCGTGACGGACTATTCAGATCAAAATCTGCAAGAACTGGAGAAATGGTTAAAGAAA
GAACCAGAGGCGTTCGATTGGAGCCCAGTTGTGACCTATGTCTGTGATCTTGAAGGAAACCGCGTAAAGGGTCCT
GAAAAGGAGGAAAAGCTGCGCCAAGTGGTAAAGCAAGTATTAAAATGTGATGTTACTCAAAGCCAACCATTAGGA
GCCGTCCCACTGCCCCCGGCGGATTGTGTATTGTCAACCCTTTGCTTGCACGCAGCTTGTCCTGACTTGCCTACA
TATTGTCGTGCATTACGCAACTTAGGAAGTTTGCTTAAACCAGGAGGGTTTCTGGTCATCATGGATTATTTGAAG
TGCTCCTACTACATGATTGGGGAACAGAAGTTCTCATCTTTACCTTTGGGCCGTGAAGCCGTAGAAGCGGCCGTA
AAGGAGGCGGGGTACACCATTGAATGGTTCGAGGTTATTTCACAGTCCCCGAGCTCCACAATGTGTAATTCCGAG
GGCTTATTTTCGCTGGTTGCTCGCAAACTGAGTCGTCCTCTTCTCGAGCACCACCACCACCACCACTG

MESGFTSKDTYLSHFNPRDYLEKYYKFGSRHSAESQILKHLLKNLFKIFCLDGVKGDLLIDIGSGPTIYQLLSAC
ESFKEIVVTDYSDQNLQELEKWLKKEPEAFDWSPVVTYVCDLEGNRVKGPEKEEKLRQVVKQVLKCDVTQSQPLG
AVPLPPADCVLSTLCLHAACPDLPTYCRALRNLGSLLKPGGFLVIMDYLKCSYYMIGEQKFSSLPLGREAVEAAV
KEAGYTIEWFEVISQSPSSTMCNSEGLFSLVARKLSRPLLEHHHHHH

vS27 (*hsa*-N-NMT_A134V, D167H, A198Y, S201C, Y242P, A247C, N249G)
ATGGAGAGTGGATTTACGAGTAAGGACACTTATTTGAGTCATTTTAACCCGCGCGACTACTTAGAAAAATACTAC
AAGTTTGGAAGCCGCCATTCAGCAGAATCACAAATTCTGAAACACCTTTTAAAGAACCTGTTTAAAATCTTCTGT
TTAGATGGGGTCAAAGGAGATTTGTTGATTGACATCGGATCAGGTCCCACAATTTACCAACTTCTGTCCGCTTGT
GAGAGTTTTAAGGAGATCGTCGTGACGGACTATTCAGATCAAAATCTGCAAGAACTGGAGAAATGGTTAAAGAAA
GAACCAGAGGCGTTCGATTGGAGCCCAGTTGTGACCTATGTCTGTGATCTTGAAGGAAACCGCGTAAAGGGTCCT
GAAAAGGAGGAAAAGCTGCGCCAAGTGGTAAAGCAAGTATTAAAATGTGATGTTACTCAAAGCCAACCATTAGGA
GCCGTCCCACTGCCCCCGGCGGATTGTGTATTGTCAACCCTTTGCTTGCACGCAGCTTGTCCTGACTTGCCTACA
TATTGTCGTGCATTACGCAACTTAGGAAGTTTGCTTAAACCAGGAGGGTTTCTGGTCATCATGGATTATTTGAAG
TGCTCCTACTACATGATTGGGGAACAGAAGTTCTCATCTTTACCTTTGGGCCGTGAAGCCGTAGAAGCGGCCGTA
AAGGAGGCGGGGTACACCATTGAATGGTTCGAGGTTATTTCACAGTCCCCGAGCTCCACAATGTGTAATGGGGAG
GGCTTATTTTCGCTGGTTGCTCGCAAACTGAGTCGTCCTCTTCTCGAGCACCACCACCACCACCACTGA

MESGFTSKDTYLSHFNPRDYLEKYYKFGSRHSAESQILKHLLKNLFKIFCLDGVKGDLLIDIGSGPTIYQLLSAC
ESFKEIVVTDYSDQNLQELEKWLKKEPEAFDWSPVVTYVCDLEGNRVKGPEKEEKLRQVVKQVLKCDVTQSQPLG
AVPLPPADCVLSTLCLHAACPDLPTYCRALRNLGSLLKPGGFLVIMDYLKCSYYMIGEQKFSSLPLGREAVEAAV
KEAGYTIEWFEVISQSPSSTMCNGEGLFSLVARKLSRPLLEHHHHHH

vS28 (*hsa*-N-NMT_A134V, D167H, A198Y, S201C, Y242P, N249G)
ATGGAGAGTGGATTTACGAGTAAGGACACTTATTTGAGTCATTTTAACCCGCGCGACTACTTAGAAAAATACTAC
AAGTTTGGAAGCCGCCATTCAGCAGAATCACAAATTCTGAAACACCTTTTAAAGAACCTGTTTAAAATCTTCTGT
TTAGATGGGGTCAAAGGAGATTTGTTGATTGACATCGGATCAGGTCCCACAATTTACCAACTTCTGTCCGCTTGT
GAGAGTTTTAAGGAGATCGTCGTGACGGACTATTCAGATCAAAATCTGCAAGAACTGGAGAAATGGTTAAAGAAA
GAACCAGAGGCGTTCGATTGGAGCCCAGTTGTGACCTATGTCTGTGATCTTGAAGGAAACCGCGTAAAGGGTCCT
GAAAAGGAGGAAAAGCTGCGCCAAGTGGTAAAGCAAGTATTAAAATGTGATGTTACTCAAAGCCAACCATTAGGA
GCCGTCCCACTGCCCCCGGCGGATTGTGTATTGTCAACCCTTTGCTTGCACGCAGCTTGTCCTGACTTGCCTACA
TATTGTCGTGCATTACGCAACTTAGGAAGTTTGCTTAAACCAGGAGGGTTTCTGGTCATCATGGATTATTTGAAG
TGCTCCTACTACATGATTGGGGAACAGAAGTTCTCATCTTTACCTTTGGGCCGTGAAGCCGTAGAAGCGGCCGTA
AAGGAGGCGGGGTACACCATTGAATGGTTCGAGGTTATTTCACAGTCCCCGAGCTCCACAATGGCAAATGGGGAG
GGCTTATTTTCGCTGGTTGCTCGCAAACTGAGTCGTCCTCTTCTCGAGCACCACCACCACCACCACTGA

MESGFTSKDTYLSHFNPRDYLEKYYKFGSRHSAESQILKHLLKNLFKIFCLDGVKGDLLIDIGSGPTIYQLLSAC
ESFKEIVVTDYSDQNLQELEKWLKKEPEAFDWSPVVTYVCDLEGNRVKGPEKEEKLRQVVKQVLKCDVTQSQPLG
AVPLPPADCVLSTLCLHAACPDLPTYCRALRNLGSLLKPGGFLVIMDYLKCSYYMIGEQKFSSLPLGREAVEAAV
KEAGYTIEWFEVISQSPSSTMANGEGLFSLVARKLSRPLLEHHHHHH

vS29 (*hsa*-N-NMT_A134V, D167H, A198M, S201C, Y242P, N249G)
ATGGAGAGTGGATTTACGAGTAAGGACACTTATTTGAGTCATTTTAACCCGCGCGACTACTTAGAAAAATACTAC
AAGTTTGGAAGCCGCCATTCAGCAGAATCACAAATTCTGAAACACCTTTTAAAGAACCTGTTTAAAATCTTCTGT
TTAGATGGGGTCAAAGGAGATTTGTTGATTGACATCGGATCAGGTCCCACAATTTACCAACTTCTGTCCGCTTGT
GAGAGTTTTAAGGAGATCGTCGTGACGGACTATTCAGATCAAAATCTGCAAGAACTGGAGAAATGGTTAAAGAAA
GAACCAGAGGCGTTCGATTGGAGCCCAGTTGTGACCTATGTCTGTGATCTTGAAGGAAACCGCGTAAAGGGTCCT
GAAAAGGAGGAAAAGCTGCGCCAAGTGGTAAAGCAAGTATTAAAATGTGATGTTACTCAAAGCCAACCATTAGGA
GCCGTCCCACTGCCCCCGGCGGATTGTGTATTGTCAACCCTTTGCTTGCACGCAGCTTGTCCTGACTTGCCTACA
TATTGTCGTGCATTACGCAACTTAGGAAGTTTGCTTAAACCAGGAGGGTTTCTGGTCATCATGGATATGTTGAAG
TGCTCCTACTACATGATTGGGGAACAGAAGTTCTCATCTTTACCTTTGGGCCGTGAAGCCGTAGAAGCGGCCGTA
AAGGAGGCGGGGTACACCATTGAATGGTTCGAGGTTATTTCACAGTCCCCGAGCTCCACAATGGCAAATGGGGAG
GGCTTATTTTCGCTGGTTGCTCGCAAACTGAGTCGTCCTCTTCTCGAGCACCACCACCACCACCACTGA

MESGFTSKDTYLSHFNPRDYLEKYYKFGSRHSAESQILKHLLKNLFKIFCLDGVKGDLLIDIGSGPTIYQLLSAC
ESFKEIVVTDYSDQNLQELEKWLKKEPEAFDWSPVVTYVCDLEGNRVKGPEKEEKLRQVVKQVLKCDVTQSQPLG
AVPLPPADCVLSTLCLHAACPDLPTYCRALRNLGSLLKPGGFLVIMDMLKCSYYMIGEQKFSSLPLGREAVEAAV
KEAGYTIEWFEVISQSPSSTMANGEGLFSLVARKLSRPLLEHHHHHH

vS30 (*hsa*-N-NMT_D167L, A198H, S201C, Y242F, A247T, N249S)
ATGGAGAGTGGATTTACGAGTAAGGACACTTATTTGAGTCATTTTAACCCGCGCGACTACTTAGAAAAATACTAC
AAGTTTGGAAGCCGCCATTCAGCAGAATCACAAATTCTGAAACACCTTTTAAAGAACCTGTTTAAAATCTTCTGT
TTAGATGGGGTCAAAGGAGATTTGTTGATTGACATCGGATCAGGTCCCACAATTTACCAACTTCTGTCCGCTTGT
GAGAGTTTTAAGGAGATCGTCGTGACGGACTATTCAGATCAAAATCTGCAAGAACTGGAGAAATGGTTAAAGAAA
GAACCAGAGGCGTTCGATTGGAGCCCAGTTGTGACCTATGTCTGTGATCTTGAAGGAAACCGCGTAAAGGGTCCT
GAAAAGGAGGAAAAGCTGCGCCAAGCCGTAAAGCAAGTATTAAAATGTGATGTTACTCAAAGCCAACCATTAGGA
GCCGTCCCACTGCCCCCGGCGGATTGTGTATTGTCAACCCTTTGCTTGCTTGCAGCTTGTCCTGACTTGCCTACA
TATTGTCGTGCATTACGCAACTTAGGAAGTTTGCTTAAACCAGGAGGGTTTCTGGTCATCATGGATCACTTGAAG
TGCTCCTACTACATGATTGGGGAACAGAAGTTCTCATCTTTACCTTTGGGCCGTGAAGCCGTAGAAGCGGCCGTA
AAGGAGGCGGGGTACACCATTGAATGGTTCGAGGTTATTTCACAGTCCTTCAGCTCCACAATGACAAATTCCGAG
GGCTTATTTTCGCTGGTTGCTCGCAAACTGAGTCGTCCTCTTCTCGAGCACCACCACCACCACCACTGA

MESGFTSKDTYLSHFNPRDYLEKYYKFGSRHSAESQILKHLLKNLFKIFCLDGVKGDLLIDIGSGPTIYQLLSAC
ESFKEIVVTDYSDQNLQELEKWLKKEPEAFDWSPVVTYVCDLEGNRVKGPEKEEKLRQAVKQVLKCDVTQSQPLG
AVPLPPADCVLSTLCLLAACPDLPTYCRALRNLGSLLKPGGFLVIMDHLKCSYYMIGEQKFSSLPLGREAVEAAV
KEAGYTIEWFEVISQSFSSTMTNSEGLFSLVARKLSRPLLEHHHHHH

vS31 (*hsa*-N-NMT_D167L, A198Y, S201C, Y242P, N249G)
ATGGAGAGTGGATTTACGAGTAAGGACACTTATTTGAGTCATTTTAACCCGCGCGACTACTTAGAAAAATACTAC
AAGTTTGGAAGCCGCCATTCAGCAGAATCACAAATTCTGAAACACCTTTTAAAGAACCTGTTTAAAATCTTCTGT
TTAGATGGGGTCAAAGGAGATTTGTTGATTGACATCGGATCAGGTCCCACAATTTACCAACTTCTGTCCGCTTGT
GAGAGTTTTAAGGAGATCGTCGTGACGGACTATTCAGATCAAAATCTGCAAGAACTGGAGAAATGGTTAAAGAAA
GAACCAGAGGCGTTCGATTGGAGCCCAGTTGTGACCTATGTCTGTGATCTTGAAGGAAACCGCGTAAAGGGTCCT
GAAAAGGAGGAAAAGCTGCGCCAAGCCGTAAAGCAAGTATTAAAATGTGATGTTACTCAAAGCCAACCATTAGGA
GCCGTCCCACTGCCCCCGGCGGATTGTGTATTGTCAACCCTTTGCTTGCTTGCAGCTTGTCCTGACTTGCCTACA
TATTGTCGTGCATTACGCAACTTAGGAAGTTTGCTTAAACCAGGAGGGTTTCTGGTCATCATGGATTATTTGAAG
TGCTCCTACTACATGATTGGGGAACAGAAGTTCTCATCTTTACCTTTGGGCCGTGAAGCCGTAGAAGCGGCCGTA
AAGGAGGCGGGGTACACCATTGAATGGTTCGAGGTTATTTCACAGTCCCCGAGCTCCACAATGGCAAATGGGGAG
GGCTTATTTTCGCTGGTTGCTCGCAAACTGAGTCGTCCTCTTCTCGAGCACCACCACCACCACCACTGA

MESGFTSKDTYLSHFNPRDYLEKYYKFGSRHSAESQILKHLLKNLFKIFCLDGVKGDLLIDIGSGPTIYQLLSAC
ESFKEIVVTDYSDQNLQELEKWLKKEPEAFDWSPVVTYVCDLEGNRVKGPEKEEKLRQAVKQVLKCDVTQSQPLG
AVPLPPADCVLSTLCLLAACPDLPTYCRALRNLGSLLKPGGFLVIMDYLKCSYYMIGEQKFSSLPLGREAVEAAV
KEAGYTIEWFEVISQSPSSTMANGEGLFSLVARKLSRPLLEHHHHHH

vS32 (*hsa*-N-NMT_D167S, A198H, S201C, Y242F, N249S)
ATGGAGAGTGGATTTACGAGTAAGGACACTTATTTGAGTCATTTTAACCCGCGCGACTACTTAGAAAAATACTAC
AAGTTTGGAAGCCGCCATTCAGCAGAATCACAAATTCTGAAACACCTTTTAAAGAACCTGTTTAAAATCTTCTGT
TTAGATGGGGTCAAAGGAGATTTGTTGATTGACATCGGATCAGGTCCCACAATTTACCAACTTCTGTCCGCTTGT
GAGAGTTTTAAGGAGATCGTCGTGACGGACTATTCAGATCAAAATCTGCAAGAACTGGAGAAATGGTTAAAGAAA
GAACCAGAGGCGTTCGATTGGAGCCCAGTTGTGACCTATGTCTGTGATCTTGAAGGAAACCGCGTAAAGGGTCCT
GAAAAGGAGGAAAAGCTGCGCCAAGCCGTAAAGCAAGTATTAAAATGTGATGTTACTCAAAGCCAACCATTAGGA
GCCGTCCCACTGCCCCCGGCGGATTGTGTATTGTCAACCCTTTGCTTGTCGGCAGCTTGTCCTGACTTGCCTACA
TATTGTCGTGCATTACGCAACTTAGGAAGTTTGCTTAAACCAGGAGGGTTTCTGGTCATCATGGATCACTTGAAG
TGCTCCTACTACATGATTGGGGAACAGAAGTTCTCATCTTTACCTTTGGGCCGTGAAGCCGTAGAAGCGGCCGTA
AAGGAGGCGGGGTACACCATTGAATGGTTCGAGGTTATTTCACAGTCCTTCAGCTCCACAATGGCAAATTCCGAG
GGCTTATTTTCGCTGGTTGCTCGCAAACTGAGTCGTCCTCTTCTCGAGCACCACCACCACCACCACTGA

MESGFTSKDTYLSHFNPRDYLEKYYKFGSRHSAESQILKHLLKNLFKIFCLDGVKGDLLIDIGSGPTIYQLLSAC
ESFKEIVVTDYSDQNLQELEKWLKKEPEAFDWSPVVTYVCDLEGNRVKGPEKEEKLRQAVKQVLKCDVTQSQPLG
AVPLPPADCVLSTLCLSAACPDLPTYCRALRNLGSLLKPGGFLVIMDHLKCSYYMIGEQKFSSLPLGREAVEAAV
KEAGYTIEWFEVISQSFSSTMANSEGLFSLVARKLSRPLLEHHHHHH

vS33 (*hsa*-N-NMT_D167S, A198H, S201C, Y242P, N249S)
ATGGAGAGTGGATTTACGAGTAAGGACACTTATTTGAGTCATTTTAACCCGCGCGACTACTTAGAAAAATACTAC
AAGTTTGGAAGCCGCCATTCAGCAGAATCACAAATTCTGAAACACCTTTTAAAGAACCTGTTTAAAATCTTCTGT
TTAGATGGGGTCAAAGGAGATTTGTTGATTGACATCGGATCAGGTCCCACAATTTACCAACTTCTGTCCGCTTGT
GAGAGTTTTAAGGAGATCGTCGTGACGGACTATTCAGATCAAAATCTGCAAGAACTGGAGAAATGGTTAAAGAAA
GAACCAGAGGCGTTCGATTGGAGCCCAGTTGTGACCTATGTCTGTGATCTTGAAGGAAACCGCGTAAAGGGTCCT
GAAAAGGAGGAAAAGCTGCGCCAAGCCGTAAAGCAAGTATTAAAATGTGATGTTACTCAAAGCCAACCATTAGGA
GCCGTCCCACTGCCCCCGGCGGATTGTGTATTGTCAACCCTTTGCTTGTCGGCAGCTTGTCCTGACTTGCCTACA
TATTGTCGTGCATTACGCAACTTAGGAAGTTTGCTTAAACCAGGAGGGTTTCTGGTCATCATGGATCACTTGAAG
TGCTCCTACTACATGATTGGGGAACAGAAGTTCTCATCTTTACCTTTGGGCCGTGAAGCCGTAGAAGCGGCCGTA
AAGGAGGCGGGGTACACCATTGAATGGTTCGAGGTTATTTCACAGTCCCCGAGCTCCACAATGGCAAATTCCGAG
GGCTTATTTTCGCTGGTTGCTCGCAAACTGAGTCGTCCTCTTCTCGAGCACCACCACCACCACCACTGA

MESGFTSKDTYLSHFNPRDYLEKYYKFGSRHSAESQILKHLLKNLFKIFCLDGVKGDLLIDIGSGPTIYQLLSAC
ESFKEIVVTDYSDQNLQELEKWLKKEPEAFDWSPVVTYVCDLEGNRVKGPEKEEKLRQAVKQVLKCDVTQSQPLG
AVPLPPADCVLSTLCLSAACPDLPTYCRALRNLGSLLKPGGFLVIMDHLKCSYYMIGEQKFSSLPLGREAVEAAV
KEAGYTIEWFEVISQSPSSTMANSEGLFSLVARKLSRPLLEHHHHHH

vS34 (*hsa*-N-NMT_D167S, A198Y, S201C, Y242P, A247C, N249G)
ATGGAGAGTGGATTTACGAGTAAGGACACTTATTTGAGTCATTTTAACCCGCGCGACTACTTAGAAAAATACTAC
AAGTTTGGAAGCCGCCATTCAGCAGAATCACAAATTCTGAAACACCTTTTAAAGAACCTGTTTAAAATCTTCTGT
TTAGATGGGGTCAAAGGAGATTTGTTGATTGACATCGGATCAGGTCCCACAATTTACCAACTTCTGTCCGCTTGT
GAGAGTTTTAAGGAGATCGTCGTGACGGACTATTCAGATCAAAATCTGCAAGAACTGGAGAAATGGTTAAAGAAA
GAACCAGAGGCGTTCGATTGGAGCCCAGTTGTGACCTATGTCTGTGATCTTGAAGGAAACCGCGTAAAGGGTCCT
GAAAAGGAGGAAAAGCTGCGCCAAGCCGTAAAGCAAGTATTAAAATGTGATGTTACTCAAAGCCAACCATTAGGA
GCCGTCCCACTGCCCCCGGCGGATTGTGTATTGTCAACCCTTTGCTTGTCGGCAGCTTGTCCTGACTTGCCTACA
TATTGTCGTGCATTACGCAACTTAGGAAGTTTGCTTAAACCAGGAGGGTTTCTGGTCATCATGGATTATTTGAAG
TGCTCCTACTACATGATTGGGGAACAGAAGTTCTCATCTTTACCTTTGGGCCGTGAAGCCGTAGAAGCGGCCGTA
AAGGAGGCGGGGTACACCATTGAATGGTTCGAGGTTATTTCACAGTCCCCGAGCTCCACAATGTGTAATGGGGAG
GGCTTATTTTCGCTGGTTGCTCGCAAACTGAGTCGTCCTCTTCTCGAGCACCACCACCACCACCACTGA

MESGFTSKDTYLSHFNPRDYLEKYYKFGSRHSAESQILKHLLKNLFKIFCLDGVKGDLLIDIGSGPTIYQLLSAC
ESFKEIVVTDYSDQNLQELEKWLKKEPEAFDWSPVVTYVCDLEGNRVKGPEKEEKLRQAVKQVLKCDVTQSQPLG
AVPLPPADCVLSTLCLSAACPDLPTYCRALRNLGSLLKPGGFLVIMDYLKCSYYMIGEQKFSSLPLGREAVEAAV
KEAGYTIEWFEVISQSPSSTMCNGEGLFSLVARKLSRPLLEHHHHHH

vS35 (*hsa*-N-NMT_D167S, A198Y, S201C, Y242P, A247T, N249G)
ATGGAGAGTGGATTTACGAGTAAGGACACTTATTTGAGTCATTTTAACCCGCGCGACTACTTAGAAAAATACTAC
AAGTTTGGAAGCCGCCATTCAGCAGAATCACAAATTCTGAAACACCTTTTAAAGAACCTGTTTAAAATCTTCTGT
TTAGATGGGGTCAAAGGAGATTTGTTGATTGACATCGGATCAGGTCCCACAATTTACCAACTTCTGTCCGCTTGT
GAGAGTTTTAAGGAGATCGTCGTGACGGACTATTCAGATCAAAATCTGCAAGAACTGGAGAAATGGTTAAAGAAA
GAACCAGAGGCGTTCGATTGGAGCCCAGTTGTGACCTATGTCTGTGATCTTGAAGGAAACCGCGTAAAGGGTCCT
GAAAAGGAGGAAAAGCTGCGCCAAGCCGTAAAGCAAGTATTAAAATGTGATGTTACTCAAAGCCAACCATTAGGA
GCCGTCCCACTGCCCCCGGCGGATTGTGTATTGTCAACCCTTTGCTTGTCGGCAGCTTGTCCTGACTTGCCTACA
TATTGTCGTGCATTACGCAACTTAGGAAGTTTGCTTAAACCAGGAGGGTTTCTGGTCATCATGGATTATTTGAAG
TGCTCCTACTACATGATTGGGGAACAGAAGTTCTCATCTTTACCTTTGGGCCGTGAAGCCGTAGAAGCGGCCGTA
AAGGAGGCGGGGTACACCATTGAATGGTTCGAGGTTATTTCACAGTCCCCGAGCTCCACAATGACAAATGGGGAG
GGCTTATTTTCGCTGGTTGCTCGCAAACTGAGTCGTCCTCTTCTCGAGCACCACCACCACCACCACTGA

MESGFTSKDTYLSHFNPRDYLEKYYKFGSRHSAESQILKHLLKNLFKIFCLDGVKGDLLIDIGSGPTIYQLLSAC
ESFKEIVVTDYSDQNLQELEKWLKKEPEAFDWSPVVTYVCDLEGNRVKGPEKEEKLRQAVKQVLKCDVTQSQPLG
AVPLPPADCVLSTLCLSAACPDLPTYCRALRNLGSLLKPGGFLVIMDYLKCSYYMIGEQKFSSLPLGREAVEAAV
KEAGYTIEWFEVISQSPSSTMTNGEGLFSLVARKLSRPLLEHHHHHH

vS36 (*hsa*-N-NMT_D167S, A198M, S201C, Y242F, A247C, N249S)
ATGGAGAGTGGATTTACGAGTAAGGACACTTATTTGAGTCATTTTAACCCGCGCGACTACTTAGAAAAATACTAC
AAGTTTGGAAGCCGCCATTCAGCAGAATCACAAATTCTGAAACACCTTTTAAAGAACCTGTTTAAAATCTTCTGT
TTAGATGGGGTCAAAGGAGATTTGTTGATTGACATCGGATCAGGTCCCACAATTTACCAACTTCTGTCCGCTTGT
GAGAGTTTTAAGGAGATCGTCGTGACGGACTATTCAGATCAAAATCTGCAAGAACTGGAGAAATGGTTAAAGAAA
GAACCAGAGGCGTTCGATTGGAGCCCAGTTGTGACCTATGTCTGTGATCTTGAAGGAAACCGCGTAAAGGGTCCT
GAAAAGGAGGAAAAGCTGCGCCAAGCCGTAAAGCAAGTATTAAAATGTGATGTTACTCAAAGCCAACCATTAGGA
GCCGTCCCACTGCCCCCGGCGGATTGTGTATTGTCAACCCTTTGCTTGTCGGCAGCTTGTCCTGACTTGCCTACA
TATTGTCGTGCATTACGCAACTTAGGAAGTTTGCTTAAACCAGGAGGGTTTCTGGTCATCATGGATATGTTGAAG
TGCTCCTACTACATGATTGGGGAACAGAAGTTCTCATCTTTACCTTTGGGCCGTGAAGCCGTAGAAGCGGCCGTA
AAGGAGGCGGGGTACACCATTGAATGGTTCGAGGTTATTTCACAGTCCTTCAGCTCCACAATGTGTAATTCCGAG
GGCTTATTTTCGCTGGTTGCTCGCAAACTGAGTCGTCCTCTTCTCGAGCACCACCACCACCACCACTGA

MESGFTSKDTYLSHFNPRDYLEKYYKFGSRHSAESQILKHLLKNLFKIFCLDGVKGDLLIDIGSGPTIYQLLSAC
ESFKEIVVTDYSDQNLQELEKWLKKEPEAFDWSPVVTYVCDLEGNRVKGPEKEEKLRQAVKQVLKCDVTQSQPLG
AVPLPPADCVLSTLCLSAACPDLPTYCRALRNLGSLLKPGGFLVIMDMLKCSYYMIGEQKFSSLPLGREAVEAAV
KEAGYTIEWFEVISQSFSSTMCNSEGLFSLVARKLSRPLLEHHHHHH

vS37 (*hsa*-N-NMT_D167P, A198M, S201C, Y242F, A247T, N249S)
ATGGAGAGTGGATTTACGAGTAAGGACACTTATTTGAGTCATTTTAACCCGCGCGACTACTTAGAAAAATACTAC
AAGTTTGGAAGCCGCCATTCAGCAGAATCACAAATTCTGAAACACCTTTTAAAGAACCTGTTTAAAATCTTCTGT
TTAGATGGGGTCAAAGGAGATTTGTTGATTGACATCGGATCAGGTCCCACAATTTACCAACTTCTGTCCGCTTGT
GAGAGTTTTAAGGAGATCGTCGTGACGGACTATTCAGATCAAAATCTGCAAGAACTGGAGAAATGGTTAAAGAAA
GAACCAGAGGCGTTCGATTGGAGCCCAGTTGTGACCTATGTCTGTGATCTTGAAGGAAACCGCGTAAAGGGTCCT
GAAAAGGAGGAAAAGCTGCGCCAAGCCGTAAAGCAAGTATTAAAATGTGATGTTACTCAAAGCCAACCATTAGGA
GCCGTCCCACTGCCCCCGGCGGATTGTGTATTGTCAACCCTTTGCTTGCCGGCAGCTTGTCCTGACTTGCCTACA
TATTGTCGTGCATTACGCAACTTAGGAAGTTTGCTTAAACCAGGAGGGTTTCTGGTCATCATGGATATGTTGAAG
TGCTCCTACTACATGATTGGGGAACAGAAGTTCTCATCTTTACCTTTGGGCCGTGAAGCCGTAGAAGCGGCCGTA
AAGGAGGCGGGGTACACCATTGAATGGTTCGAGGTTATTTCACAGTCCTTCAGCTCCACAATGACAAATTCCGAG
GGCTTATTTTCGCTGGTTGCTCGCAAACTGAGTCGTCCTCTTCTCGAGCACCACCACCACCACCACTGA

MESGFTSKDTYLSHFNPRDYLEKYYKFGSRHSAESQILKHLLKNLFKIFCLDGVKGDLLIDIGSGPTIYQLLSAC
ESFKEIVVTDYSDQNLQELEKWLKKEPEAFDWSPVVTYVCDLEGNRVKGPEKEEKLRQAVKQVLKCDVTQSQPLG
AVPLPPADCVLSTLCLPAACPDLPTYCRALRNLGSLLKPGGFLVIMDMLKCSYYMIGEQKFSSLPLGREAVEAAV
KEAGYTIEWFEVISQSFSSTMTNSEGLFSLVARKLSRPLLEHHHHHH

vS38 (*hsa*-N-NMT_D167H, A198H, S201C, Y242F, N249S)
ATGGAGAGTGGATTTACGAGTAAGGACACTTATTTGAGTCATTTTAACCCGCGCGACTACTTAGAAAAATACTAC
AAGTTTGGAAGCCGCCATTCAGCAGAATCACAAATTCTGAAACACCTTTTAAAGAACCTGTTTAAAATCTTCTGT
TTAGATGGGGTCAAAGGAGATTTGTTGATTGACATCGGATCAGGTCCCACAATTTACCAACTTCTGTCCGCTTGT
GAGAGTTTTAAGGAGATCGTCGTGACGGACTATTCAGATCAAAATCTGCAAGAACTGGAGAAATGGTTAAAGAAA
GAACCAGAGGCGTTCGATTGGAGCCCAGTTGTGACCTATGTCTGTGATCTTGAAGGAAACCGCGTAAAGGGTCCT
GAAAAGGAGGAAAAGCTGCGCCAAGCCGTAAAGCAAGTATTAAAATGTGATGTTACTCAAAGCCAACCATTAGGA
GCCGTCCCACTGCCCCCGGCGGATTGTGTATTGTCAACCCTTTGCTTGCACGCAGCTTGTCCTGACTTGCCTACA
TATTGTCGTGCATTACGCAACTTAGGAAGTTTGCTTAAACCAGGAGGGTTTCTGGTCATCATGGATCACTTGAAG
TGCTCCTACTACATGATTGGGGAACAGAAGTTCTCATCTTTACCTTTGGGCCGTGAAGCCGTAGAAGCGGCCGTA
AAGGAGGCGGGGTACACCATTGAATGGTTCGAGGTTATTTCACAGTCCTTCAGCTCCACAATGGCAAATTCCGAG
GGCTTATTTTCGCTGGTTGCTCGCAAACTGAGTCGTCCTCTTCTCGAGCACCACCACCACCACCACTGA

MESGFTSKDTYLSHFNPRDYLEKYYKFGSRHSAESQILKHLLKNLFKIFCLDGVKGDLLIDIGSGPTIYQLLSAC
ESFKEIVVTDYSDQNLQELEKWLKKEPEAFDWSPVVTYVCDLEGNRVKGPEKEEKLRQAVKQVLKCDVTQSQPLG
AVPLPPADCVLSTLCLHAACPDLPTYCRALRNLGSLLKPGGFLVIMDHLKCSYYMIGEQKFSSLPLGREAVEAAV
KEAGYTIEWFEVISQSFSSTMANSEGLFSLVARKLSRPLLEHHHHHH

vS39 (*hsa*-N-NMT_D167H, A198H, S201C, Y242P, N249S)
ATGGAGAGTGGATTTACGAGTAAGGACACTTATTTGAGTCATTTTAACCCGCGCGACTACTTAGAAAAATACTAC
AAGTTTGGAAGCCGCCATTCAGCAGAATCACAAATTCTGAAACACCTTTTAAAGAACCTGTTTAAAATCTTCTGT
TTAGATGGGGTCAAAGGAGATTTGTTGATTGACATCGGATCAGGTCCCACAATTTACCAACTTCTGTCCGCTTGT
GAGAGTTTTAAGGAGATCGTCGTGACGGACTATTCAGATCAAAATCTGCAAGAACTGGAGAAATGGTTAAAGAAA
GAACCAGAGGCGTTCGATTGGAGCCCAGTTGTGACCTATGTCTGTGATCTTGAAGGAAACCGCGTAAAGGGTCCT
GAAAAGGAGGAAAAGCTGCGCCAAGCCGTAAAGCAAGTATTAAAATGTGATGTTACTCAAAGCCAACCATTAGGA
GCCGTCCCACTGCCCCCGGCGGATTGTGTATTGTCAACCCTTTGCTTGCACGCAGCTTGTCCTGACTTGCCTACA
TATTGTCGTGCATTACGCAACTTAGGAAGTTTGCTTAAACCAGGAGGGTTTCTGGTCATCATGGATCACTTGAAG
TGCTCCTACTACATGATTGGGGAACAGAAGTTCTCATCTTTACCTTTGGGCCGTGAAGCCGTAGAAGCGGCCGTA
AAGGAGGCGGGGTACACCATTGAATGGTTCGAGGTTATTTCACAGTCCCCGAGCTCCACAATGGCAAATTCCGAG
GGCTTATTTTCGCTGGTTGCTCGCAAACTGAGTCGTCCTCTTCTCGAGCACCACCACCACCACCACTGA

MESGFTSKDTYLSHFNPRDYLEKYYKFGSRHSAESQILKHLLKNLFKIFCLDGVKGDLLIDIGSGPTIYQLLSAC
ESFKEIVVTDYSDQNLQELEKWLKKEPEAFDWSPVVTYVCDLEGNRVKGPEKEEKLRQAVKQVLKCDVTQSQPLG
AVPLPPADCVLSTLCLHAACPDLPTYCRALRNLGSLLKPGGFLVIMDHLKCSYYMIGEQKFSSLPLGREAVEAAV
KEAGYTIEWFEVISQSPSSTMANSEGLFSLVARKLSRPLLEHHHHHH

vS40 (*hsa*-N-NMT_D167H, A198H, S201C, Y242P, A247T, N249S)
ATGGAGAGTGGATTTACGAGTAAGGACACTTATTTGAGTCATTTTAACCCGCGCGACTACTTAGAAAAATACTAC
AAGTTTGGAAGCCGCCATTCAGCAGAATCACAAATTCTGAAACACCTTTTAAAGAACCTGTTTAAAATCTTCTGT
TTAGATGGGGTCAAAGGAGATTTGTTGATTGACATCGGATCAGGTCCCACAATTTACCAACTTCTGTCCGCTTGT
GAGAGTTTTAAGGAGATCGTCGTGACGGACTATTCAGATCAAAATCTGCAAGAACTGGAGAAATGGTTAAAGAAA
GAACCAGAGGCGTTCGATTGGAGCCCAGTTGTGACCTATGTCTGTGATCTTGAAGGAAACCGCGTAAAGGGTCCT
GAAAAGGAGGAAAAGCTGCGCCAAGCCGTAAAGCAAGTATTAAAATGTGATGTTACTCAAAGCCAACCATTAGGA
GCCGTCCCACTGCCCCCGGCGGATTGTGTATTGTCAACCCTTTGCTTGCACGCAGCTTGTCCTGACTTGCCTACA
TATTGTCGTGCATTACGCAACTTAGGAAGTTTGCTTAAACCAGGAGGGTTTCTGGTCATCATGGATCACTTGAAG
TGCTCCTACTACATGATTGGGGAACAGAAGTTCTCATCTTTACCTTTGGGCCGTGAAGCCGTAGAAGCGGCCGTA
AAGGAGGCGGGGTACACCATTGAATGGTTCGAGGTTATTTCACAGTCCCCGAGCTCCACAATGACAAATTCCGAG
GGCTTATTTTCGCTGGTTGCTCGCAAACTGAGTCGTCCTCTTCTCGAGCACCACCACCACCACCACTGA

MESGFTSKDTYLSHFNPRDYLEKYYKFGSRHSAESQILKHLLKNLFKIFCLDGVKGDLLIDIGSGPTIYQLLSAC
ESFKEIVVTDYSDQNLQELEKWLKKEPEAFDWSPVVTYVCDLEGNRVKGPEKEEKLRQAVKQVLKCDVTQSQPLG
AVPLPPADCVLSTLCLHAACPDLPTYCRALRNLGSLLKPGGFLVIMDHLKCSYYMIGEQKFSSLPLGREAVEAAV
KEAGYTIEWFEVISQSPSSTMTNSEGLFSLVARKLSRPLLEHHHHHH

vS41 (*hsa*-N-NMT_D167H, A198H, S201C, Y242F, A247T, N249S)

```
ATGGAGAGTGGATTTACGAGTAAGGACACTTATTTGAGTCATTTTAACCCGCGCGACTACTTAGAAAAATACTAC
AAGTTTGGAAGCCGCCATTCAGCAGAATCACAAATTCTGAAACACCTTTTAAAGAACCTGTTTAAAATCTTCTGT
TTAGATGGGGTCAAAGGAGATTTGTTGATTGACATCGGATCAGGTCCCACAATTTACCAACTTCTGTCCGCTTGT
GAGAGTTTTAAGGAGATCGTCGTGACGGACTATTCAGATCAAAATCTGCAAGAACTGGAGAAATGGTTAAAGAAA
GAACCAGAGGCGTTCGATTGGAGCCCAGTTGTGACCTATGTCTGTGATCTTGAAGGAAACCGCGTAAAGGGTCCT
GAAAAGGAGGAAAAGCTGCGCCAAGCCGTAAAGCAAGTATTAAAATGTGATGTTACTCAAAGCCAACCATTAGGA
GCCGTCCCACTGCCCCCGGCGGATTGTGTATTGTCAACCCTTTGCTTGCACGCAGCTTGTCCTGACTTGCCTACA
TATTGTCGTGCATTACGCAACTTAGGAAGTTTGCTTAAACCAGGAGGGTTTCTGGTCATCATGGATCACTTGAAG
TGCTCCTACTACATGATTGGGGAACAGAAGTTCTCATCTTTACCTTTGGGCCGTGAAGCCGTAGAAGCGGCCGTA
AAGGAGGCGGGGTACACCATTGAATGGTTCGAGGTTATTTCACAGTCCTTCAGCTCCACAATGACAAATTCCGAG
GGCTTATTTTCGCTGGTTGCTCGCAAACTGAGTCGTCCTCTTCTCGAGCACCACCACCACCACCACTGA
```

```
MESGFTSKDTYLSHFNPRDYLEKYYKFGSRHSAESQILKHLLKNLFKIFCLDGVKGDLLIDIGSGPTIYQLLSAC
ESFKEIVVTDYSDQNLQELEKWLKKEPEAFDWSPVVTYVCDLEGNRVKGPEKEEKLRQAVKQVLKCDVTQSQPLG
AVPLPPADCVLSTLCLHAACPDLPTYCRALRNLGSLLKPGGFLVIMDHLKCSYYMIGEQKFSSLPLGREAVEAAV
KEAGYTIEWFEVISQSFSSTMTNSEGLFSLVARKLSRPLLEHHHHHH
```

vS42 (*hsa*-N-NMT_D167H, A198Y, S201C, Y242P, N249S)

```
ATGGAGAGTGGATTTACGAGTAAGGACACTTATTTGAGTCATTTTAACCCGCGCGACTACTTAGAAAAATACTAC
AAGTTTGGAAGCCGCCATTCAGCAGAATCACAAATTCTGAAACACCTTTTAAAGAACCTGTTTAAAATCTTCTGT
TTAGATGGGGTCAAAGGAGATTTGTTGATTGACATCGGATCAGGTCCCACAATTTACCAACTTCTGTCCGCTTGT
GAGAGTTTTAAGGAGATCGTCGTGACGGACTATTCAGATCAAAATCTGCAAGAACTGGAGAAATGGTTAAAGAAA
GAACCAGAGGCGTTCGATTGGAGCCCAGTTGTGACCTATGTCTGTGATCTTGAAGGAAACCGCGTAAAGGGTCCT
GAAAAGGAGGAAAAGCTGCGCCAAGCCGTAAAGCAAGTATTAAAATGTGATGTTACTCAAAGCCAACCATTAGGA
GCCGTCCCACTGCCCCCGGCGGATTGTGTATTGTCAACCCTTTGCTTGCACGCAGCTTGTCCTGACTTGCCTACA
TATTGTCGTGCATTACGCAACTTAGGAAGTTTGCTTAAACCAGGAGGGTTTCTGGTCATCATGGATTATTTGAAG
TGCTCCTACTACATGATTGGGGAACAGAAGTTCTCATCTTTACCTTTGGGCCGTGAAGCCGTAGAAGCGGCCGTA
AAGGAGGCGGGGTACACCATTGAATGGTTCGAGGTTATTTCACAGTCCCCGAGCTCCACAATGGCAAATTCCGAG
GGCTTATTTTCGCTGGTTGCTCGCAAACTGAGTCGTCCTCTTCTCGAGCACCACCACCACCACCACTGA
```

```
MESGFTSKDTYLSHFNPRDYLEKYYKFGSRHSAESQILKHLLKNLFKIFCLDGVKGDLLIDIGSGPTIYQLLSAC
ESFKEIVVTDYSDQNLQELEKWLKKEPEAFDWSPVVTYVCDLEGNRVKGPEKEEKLRQAVKQVLKCDVTQSQPLG
AVPLPPADCVLSTLCLHAACPDLPTYCRALRNLGSLLKPGGFLVIMDYLKCSYYMIGEQKFSSLPLGREAVEAAV
KEAGYTIEWFEVISQSPSSTMANSEGLFSLVARKLSRPLLEHHHHHH
```

vS43 (*hsa*-N-NMT_D167H, A198Y, S201C, Y242P, A247C, N249G)

```
ATGGAGAGTGGATTTACGAGTAAGGACACTTATTTGAGTCATTTTAACCCGCGCGACTACTTAGAAAAATACTAC
AAGTTTGGAAGCCGCCATTCAGCAGAATCACAAATTCTGAAACACCTTTTAAAGAACCTGTTTAAAATCTTCTGT
TTAGATGGGGTCAAAGGAGATTTGTTGATTGACATCGGATCAGGTCCCACAATTTACCAACTTCTGTCCGCTTGT
GAGAGTTTTAAGGAGATCGTCGTGACGGACTATTCAGATCAAAATCTGCAAGAACTGGAGAAATGGTTAAAGAAA
GAACCAGAGGCGTTCGATTGGAGCCCAGTTGTGACCTATGTCTGTGATCTTGAAGGAAACCGCGTAAAGGGTCCT
GAAAAGGAGGAAAAGCTGCGCCAAGCCGTAAAGCAAGTATTAAAATGTGATGTTACTCAAAGCCAACCATTAGGA
GCCGTCCCACTGCCCCCGGCGGATTGTGTATTGTCAACCCTTTGCTTGCACGCAGCTTGTCCTGACTTGCCTACA
TATTGTCGTGCATTACGCAACTTAGGAAGTTTGCTTAAACCAGGAGGGTTTCTGGTCATCATGGATTATTTGAAG
TGCTCCTACTACATGATTGGGGAACAGAAGTTCTCATCTTTACCTTTGGGCCGTGAAGCCGTAGAAGCGGCCGTA
AAGGAGGCGGGGTACACCATTGAATGGTTCGAGGTTATTTCACAGTCCCCGAGCTCCACAATGTGTAATGGGGAG
GGCTTATTTTCGCTGGTTGCTCGCAAACTGAGTCGTCCTCTTCTCGAGCACCACCACCACCACCACTGA
```

```
MESGFTSKDTYLSHFNPRDYLEKYYKFGSRHSAESQILKHLLKNLFKIFCLDGVKGDLLIDIGSGPTIYQLLSAC
ESFKEIVVTDYSDQNLQELEKWLKKEPEAFDWSPVVTYVCDLEGNRVKGPEKEEKLRQAVKQVLKCDVTQSQPLG
AVPLPPADCVLSTLCLHAACPDLPTYCRALRNLGSLLKPGGFLVIMDYLKCSYYMIGEQKFSSLPLGREAVEAAV
KEAGYTIEWFEVISQSPSSTMCNGEGLFSLVARKLSRPLLEHHHHHH
```

vS44 (*hsa*-N-NMT_D167H, A198Y, S201C, Y242P, A247T, N249G)

ATGGAGAGTGGATTTACGAGTAAGGACACTTATTTGAGTCATTTTAACCCGCGCGACTACTTAGAAAAATACTAC
AAGTTTGGAAGCCGCCATTCAGCAGAATCACAAATTCTGAAACACCTTTTAAAGAACCTGTTTAAAATCTTCTGT
TTAGATGGGGTCAAAGGAGATTTGTTGATTGACATCGGATCAGGTCCCACAATTTACCAACTTCTGTCCGCTTGT
GAGAGTTTTAAGGAGATCGTCGTGACGGACTATTCAGATCAAAATCTGCAAGAACTGGAGAAATGGTTAAAGAAA
GAACCAGAGGCGTTCGATTGGAGCCCAGTTGTGACCTATGTCTGTGATCTTGAAGGAAACCGCGTAAAGGGTCCT
GAAAAGGAGGAAAAGCTGCGCCAAGCCGTAAAGCAAGTATTAAAATGTGATGTTACTCAAAGCCAACCATTAGGA
GCCGTCCCACTGCCCCCGGCGGATTGTGTATTGTCAACCCTTTGCTTGCACGCAGCTTGTCCTGACTTGCCTACA
TATTGTCGTGCATTACGCAACTTAGGAAGTTTGCTTAAACCAGGAGGGTTTCTGGTCATCATGGATTATTTGAAG
TGCTCCTACTACATGATTGGGGAACAGAAGTTCTCATCTTTACCTTTGGGCCGTGAAGCCGTAGAAGCGGCCGTA
AAGGAGGCGGGGTACACCATTGAATGGTTCGAGGTTATTTCACAGTCCCCGAGCTCCACAATGACAAATGGGGAG
GGCTTATTTTCGCTGGTTGCTCGCAAACTGAGTCGTCCTCTTCTCGAGCACCACCACCACCACCACTGA

MESGFTSKDTYLSHFNPRDYLEKYYKFGSRHSAESQILKHLLKNLFKIFCLDGVKGDLLIDIGSGPTIYQLLSAC
ESFKEIVVTDYSDQNLQELEKWLKKEPEAFDWSPVVTYVCDLEGNRVKGPEKEEKLRQAVKQVLKCDVTQSQPLG
AVPLPPADCVLSTLCLHAACPDLPTYCRALRNLGSLLKPGGFLVIMDYLKCSYYMIGEQKFSSLPLGREAVEAAV
KEAGYTIEWFEVISQSPSSTMTNGEGLFSLVARKLSRPLLEHHHHHH

vS45 (*hsa*-N-NMT_D167H, A198Y, S201C, Y242P, N249G)

ATGGAGAGTGGATTTACGAGTAAGGACACTTATTTGAGTCATTTTAACCCGCGCGACTACTTAGAAAAATACTAC
AAGTTTGGAAGCCGCCATTCAGCAGAATCACAAATTCTGAAACACCTTTTAAAGAACCTGTTTAAAATCTTCTGT
TTAGATGGGGTCAAAGGAGATTTGTTGATTGACATCGGATCAGGTCCCACAATTTACCAACTTCTGTCCGCTTGT
GAGAGTTTTAAGGAGATCGTCGTGACGGACTATTCAGATCAAAATCTGCAAGAACTGGAGAAATGGTTAAAGAAA
GAACCAGAGGCGTTCGATTGGAGCCCAGTTGTGACCTATGTCTGTGATCTTGAAGGAAACCGCGTAAAGGGTCCT
GAAAAGGAGGAAAAGCTGCGCCAAGCCGTAAAGCAAGTATTAAAATGTGATGTTACTCAAAGCCAACCATTAGGA
GCCGTCCCACTGCCCCCGGCGGATTGTGTATTGTCAACCCTTTGCTTGCACGCAGCTTGTCCTGACTTGCCTACA
TATTGTCGTGCATTACGCAACTTAGGAAGTTTGCTTAAACCAGGAGGGTTTCTGGTCATCATGGATTATTTGAAG
TGCTCCTACTACATGATTGGGGAACAGAAGTTCTCATCTTTACCTTTGGGCCGTGAAGCCGTAGAAGCGGCCGTA
AAGGAGGCGGGGTACACCATTGAATGGTTCGAGGTTATTTCACAGTCCCCGAGCTCCACAATGGCAAATGGGGAG
GGCTTATTTTCGCTGGTTGCTCGCAAACTGAGTCGTCCTCTTCTCGAGCACCACCACCACCACCACTGA

MESGFTSKDTYLSHFNPRDYLEKYYKFGSRHSAESQILKHLLKNLFKIFCLDGVKGDLLIDIGSGPTIYQLLSAC
ESFKEIVVTDYSDQNLQELEKWLKKEPEAFDWSPVVTYVCDLEGNRVKGPEKEEKLRQAVKQVLKCDVTQSQPLG
AVPLPPADCVLSTLCLHAACPDLPTYCRALRNLGSLLKPGGFLVIMDYLKCSYYMIGEQKFSSLPLGREAVEAAV
KEAGYTIEWFEVISQSPSSTMANGEGLFSLVARKLSRPLLEHHHHHH

Wild type 1 (A6XNE6)

ATGGACGCTGCAACTGCCGTGGAGCTTTTAGACGCACAACCACAGGTTTGGCATCACTTTCTTGGCTACATTAAT
AGTATGACCTTGCAGTGCGCGTTAGAGCTGGACATAGCGGACGTCATCCACCGGCACGGGCACCCTATACCTTTG
AACCAACTTGCTGCTGCTCTTGAGATTCCGCAAACCAAAGCCCCGTTCTTGAGTCGCTTAATGCGTATGCTGGTG
CATTTGGGATACTTCACACAAGTGATCACGAAGCCGGAAGATGAAAACGACGATGTACTTCCTAGTTACTGGTTG
GCCCCGCTGTCACGTCTGCTCCTGAAACAGAATCCTTACAATGCTCGCTCATTGACATTTTGTAGTGTGCACGAG
CACTTAGTGGACCCCTGGCGTCAAATGTCAGCTTGGCTCCGCACAGGGAAGGAAGATGGGAAGGACACACCGAAC
GCATTTGCCTTTGCCCATGAGGGCAAGAAGGTGTATGAGGTTTGTAGTGAGGACGCGAACTTCAGTCAACTTTTC
TCAGAGGGAATGGCGGGCGACTCATGGTTGTTCTCTAGAGCCTTGGTGTCCAAATGCCGAGATGCCTTCGAAGGT
TTGAGCTCTTTGGTAGACGTTGGAGGAGGCACCGGGAACACGTCAAAGGTTATCGCGGAGACCTTCCCCAATATA
CATTGTACAGTTTTCGATTTGCCACATGTGGTCTCAGGCCCGAAGCAAACGCATCCAAACCTGGATTATGAGTCC
GGTAACATGTTCACAGATGAGATCCCACACGCCGATGCCGTGTTATTTAAGTGGGTTCTTTGTGACTGGCCCGAC
GAGCCGGTGTTGAAGATGTTAAAACAATGTAAGAAGGCGTTAACGAAGAATGGTGTTAAGGGGAAGCTGATGATT
GCAGACCACGTGCTGGATCACGAATCGTGCAACGACTCTAACTCCATGGGCACTAGCTTGATTCTCGACATGCTG
TTTATGAGCTTCTTAGAGGGATCACTGCGCACTGAGAAGCAGTGGGCGAAGCTTTTCGCGGAAGCTGGATTCAAA
GACTATAAGATTACACCAGTCGGCGGCTTACGTGTACTCATCGAAGTCTACCCGCTCGAGCACCACCACCACCAC
CACTGA

MDAATAVELLDAQPQVWHHFLGYINSMTLQCALELDIADVIHRHGHPIPLNQLAAALEIPQTKAPFLSRLMRMLV
HLGYFTQVITKPEDENDDVLPSYWLAPLSRLLLKQNPYNARSLTFCSVHEHLVDPWRQMSAWLRTGKEDGKDTPN
AFAFAHEGKKVYEVCSEDANFSQLFSEGMAGDSWLFSRALVSKCRDAFEGLSSLVDVGGGTGNTSKVIAETFPNI
HCTVFDLPHVVSGPKQTHPNLDYESGNMFTDEIPHADAVLFKWVLCDWPDEPVLKMLKQCKKALTKNGVKGKLMI
ADHVLDHESCNDSNSMGTSLILDMLFMSFLEGSLRTEKQWAKLFAEAGFKDYKITPVGGLRVLIEVYPLEHHHHH
H*

## Wild type 2 (Q1WMA5)

ATGAGCTCTCACGAAGAAAAACCGTCGAGTAACAAGGAGGACGATGACCACTCGTCATATGCGTTACAACTGGTT
TTCTCTGGTGCTCTGCCGATGGTACTGAATGCGGTTATCAAACTGAATGTCTTCGAAATTATAGCCAAAGCCGGA
CCCGGTGCGAAACTGTCTCCAAGTCAAATCGTAAGCCAGATGCCGACGAAAAATCCGGAGGCTCCCGTCGTGCTG
GATCGAATGCTCAGAATGCTGGCTAGTTATAGCGTGCTGACATGCTCGGTCGTGGACTTCAGCCACGGTTCTGGA
CAACGTGTGTATGGGCTGAGCCCGGTATCAAAATATTTTGTGAAAAACGAGAACGGAGGCTGCTTTGGGCCGTTG
CTGGATTTACTGCAGGACAAGGTGCTGACCGACATTTGGTATGAACTGGCTCCCGCTGTGCTTGAGGGCGGTACC
GCGTTCAACCGGGCATACAATATGCACATTTTCAAGTATACCGGCATCAATCAGAAGTTTAACGAAACATTTAAC
ACCGCAACCATAAACCACGCGAAAGTCATCGTTCAAGAAATTTTGAAAAACTATAAGGGTTTTGAAAATTTGAAA
ACGTTAGTGGATGTCGGCGGGGGGCTGGGAGTAACACTTGATTTAATTACTTCCAAATATCCAAACCTGAAAGGC
ATTAATTACGATCTTCCGCATGTAACACAGAACGCTCCAACGTACCCCGGCGTCGTGCATGTCGGAGGAGACATG
TTCGAAAGTGTGCCTAAAGGAGATGCGATTTTTATGAAATGGATTTTACATGATTGGGACGATGAGCATTGCCTG
AAGTTACTTAAAAATTGTTATAAAGCTTTACCAGAAAACGGCAAGGTTATTGCAGTCGATGCGATCTTACCTATG
AATCCCGATAATTCTAGCTCTACCAAACACATTAGTCAAGTAGACTTGTTTACTTTAGTATTATATCACCCAGGA
GGGAAAGAACGTACAGAGAATGAGTTCCTTGCTCTGGTAGCCGAAGCTGGCTTCGGTGGTATACGCAAAGTGTGC
GTGTGCTGCGACCTTTGGGTCATGGAATTTTATAAGCTCGAGCACCACCACCACCACCACTGA

MSSHEEKPSSNKEDDDHSSYALQLVFSGALPMVLNAVIKLNVFEIIAKAGPGAKLSPSQIVSQMPTKNPEAPVVL
DRMLRMLASYSVLTCSVVDFSHGSGQRVYGLSPVSKYFVKNENGGCFGPLLDLLQDKVLTDIWYELAPAVLEGGT
AFNRAYNMHIFKYTGINQKFNETFNTATINHAKVIVQEILKNYKGFENLKTLVDVGGGLGVTLDLITSKYPNLKG
INYDLPHVTQNAPTYPGVVHVGGDMFESVPKGDAIFMKWILHDWDDEHCLKLLKNCYKALPENGKVIAVDAILPM
NPDNSSSTKHISQVDLFTLVLYHPGGKERTENEFLALVAEAGFGGIRKVCVCCDLWVMEFYKLEHHHHHH*

## Wild type 3 (Q9HWH2)

ATGAACAATTCCAACTTAGCTGCGGCGAGAAACCTCATACAGGTCGTTACCGGAGAGTGGAAGAGTCGCTGTGTG
TACGTGGCTACGCGCTTGGGCTTAGCAGATCTCATAGAAAGTGGAATAGACTCTGACGAAACTCTCGCCGCAGCG
GTCGGCTCAGACGCTGAGCGTATTCACAGACTTATGCGATTACTGGTCGCTTTCGAGATTTTCCAAGGCGACACA
AGAGATGGGTATGCAAATACCCCTACCTCTCACTTGTTACGTGATGTTGAAGGAAGTTTTCGAGACATGGTTCTT
TTCTACGGCGAGGAATTTCACGCCGCCTGGACTCCGGCTTGCGAGGCACTCCTTTCAGGCACCCCGGGATTTGAG
TTGGCCTTTGGGGAAGACTTTTACAGCTATCTGAAGCGCTGTCCTGATGCCGGCCGTCGGTTCCTGCTTGCTATG
AAAGCGAGTAACCTCGCTTTCCACGAAATACCGCGGCTCTTAGACTTCCGCGGACGTTCTTTCGTTGACGTGGGA
GGAGGTAGCGGCGAATTAACGAAAGCCATCTTGCAAGCCGAGCCATCTGCTCGCGGGGTCATGCTGGATCGCGAA
GGGAGCCTGGGTGTTGCACGCGATAACTTATCTTCTTTACTGGCTGGAGAGCGAGTATCTCTGGTGGGCGGGGAC
ATGTTACAAGAGGTGCCGTCCAATGGCGACATATATCTGTTATCTCGGATTATCGGGGACCTCGATGAAGCAGCA
TCACTGCGTTTACTTGGTAACTGCCGCGAGGCGATGGCCGGTGACGGTCGCGTAGTGGTAATTGAGCGCACAATT
TCTGCGTCTGAGCCGAGTCCCATGTCAGTTCTCTGGGATGTTCATCTGTTTATGGCGTGCGCGGGCCGTCATCGT
ACGACCGAGGAAGTAGTCGACCTTCTTGGTCGAGGTGGTTTTGCTGTGGAGCGTATAGTGGATTTGCCCATGGAA
ACCCGCATGATTGTGGCAGCAAGAGCACTCGAGCACCACCACCACCACCACTGA

MNNSNLAAARNLIQVVTGEWKSRCVYVATRLGLADLIESGIDSDETLAAAVGSDAERIHRLMRLLVAFEIFQGDT
RDGYANTPTSHLLRDVEGSFRDMVLFYGEEFHAAWTPACEALLSGTPGFELAFGEDFYSYLKRCPDAGRRFLLAM
KASNLAFHEIPRLLDFRGRSFVDVGGGSGELTKAILQAEPSARGVMLDREGSLGVARDNLSSLLAGERVSLVGGD
MLQEVPSNGDIYLLSRIIGDLDEAASLRLLGNCREAMAGDGRVVVIERTISASEPSPMSVLWDVHLFMACAGRHR
TTEEVVDLLGRGGFAVERIVDLPMETRMIVAARALEHHHHHH*

Wild type 4 (A0A166U5H3)
ATGGCAGGGATGAAGACTAGTCCTTCACAAGACGAGGAAGCGTGTGTACTGGCCATTCAGTTAGCCACATCGACA
GTGCTCCCGATGATACTGAAGTCTGCAATAGAGCTGGACATCCTTAATACTATTAGTAAGGCCGGTCCTGGTAAT
TACCTTTCCCCATCGGATCTGGCCAGTAAGTTACTCATGAGCAACCCACACGCCCCAATAATGCTTGAAAGAATA
CTGCGTGTGTTAGCAACGTATAAGGTGCTTGGGTGTAAGCCAAGTGAACTTTCAGATGGTGAGGTGGAGTGGTTA
TATTGTTGGACCCCTGTATGTAAATTTCTGAGCAACAATGAGGATGGTGCATCCATCGCGCCGTTGCTTCTTGTA
CATCAAGACCAGGTGCCAATGAAAAGCTGGTACCATCTGACAGATGCGATCCTGGACGGCGGCACAGCCTTTAAC
AAGGCATACGGCATGAACATCTTTGATTACGCATCACAGGATCCGCAATTCAATAAGGTGTTTAATCGTTCGATG
GCTGGCCACTCCACCATAACCATGAAGAAAATACTTGAAACCTATAATGGATTCGAAGGTTTAAAGTCGATTGTG
GACGTTGGTGGCGGATCAGGGGCAACCTTAAATATGATAATCTCGAAGTACCCAACCATCAAGGGGATCAATTTT
GACCTTCCACATGTCGTTGGAGATTCCCCAATACACCCAGGAGTAGAGCATGTGGGCGGTGACATGTTTGCTTCG
GTCCCTAAAGGTGATGCGATTTTCTTAAAGTGGATCTTTCACAGTTGGAGCGACGAAGATTGCTTGCGTATATTA
AAGAATTGTTACGAAGCACTGGCCGACAACAAGAAAGTTATAGTGGCAGAGTTTATCATACCTGAAGTCCCGGGC
GGTAGCGACGACGCCACGAAGAGCGTGGTACACTTGGACGCTGTTATGCTTGCTTACGTTCCTGGCGGAAAGGAG
AGAACGGAGAAAGAGTTTGAAGCCTTAGCTACGAGTGCTGGGTTTAAGAGTTTCCGCAAGGTATGTTGTGCGTTT
AACACATGGATAATGGAGTTCTCAAAGCTCGAGCACCACCACCACCACCACTGA

MAGMKTSPSQDEEACVLAIQLATSTVLPMILKSAIELDILNTISKAGPGNYLSPSDLASKLLMSNPHAPIMLERI
LRVLATYKVLGCKPSELSDGEVEWLYCWTPVCKFLSNNEDGASIAPLLLVHQDQVPMKSWYHLTDAILDGGTAFN
KAYGMNIFDYASQDPQFNKVFNRSMAGHSTITMKKILETYNGFEGLKSIVDVGGGSGATLNMIISKYPTIKGINF
DLPHVVGDSPIHPGVEHVGGDMFASVPKGDAIFLKWIFHSWSDEDCLRILKNCYEALADNKKVIVAEFIIPEVPG
GSDDATKSVVHLDAVMLAYVPGGKERTEKEFEALATSAGFKSFRKVCCAFNTWIMEFSKLEHHHHHH*

Wild type 5 (Q643C8)
ATGAGTACTGAGGTCTCAGAAGCACAAGCACGCCGTGCAGTTGCTGACATCTTTAACTCAACTCTGGCCTCTTCG
GCCATCGGCGCAGCTTGGGAGCTCGGGGCCTTAGATGAGCTGCGTGAGAACGGAAAGTTAGACGTATCCGACTTC
GCTGTACGGCATGACCTTCACGAGCCTGCGGTAGTCGGAATGTTCACTGCCTTAGCATCAGTCGGCATTGTGCGC
CGTGAAGGCGCGACCGTGGTAGTAGGTCCTTACTTCGATGAGGCAAACCACCACCGCAGTCTTTTCCATTGGCTG
AATCAAGGATCCGGGGAGCTTTTCCGACGTATGCCACAAGTGTTACCTAACGAGAACCGTACTGGTAAGTTCTAT
CAACGCGACGCTGGGGCGATCTCATATGCATGTCGTGAAATCAGTGAGCGATACTTTGATCCAGCTTTCTGGGCA
GCTGTGGATGGTCTCGGTTACACGCCGACGACGGTGGCCGACCTTGGCTCAGGTTCAGGCGAGCGACTCATCCAA
ATCGCTCGGCGTTTCCCGGGCGTTCGAGGATTGGGTGTGGATATTGCAGACGGGGCTATTGCCATGGCGGAGAAA
GAGGTAGCGGCGAAGGGCTTCGGGGACCAAATAAGCTTCGTAAGAGGTGATGCCCGCACGATCGACCAGGTTTCG
GCCCGCGGGGAATTTGCTGAGGTAGACCTGTTAACCTGTTTCATGATGGGTCACGACTTTTGGCCTCGTGAAAAC
TGCGTCCAAACCTTGCGCAAGTTGCGAGCAGCATTTCCAAATGTCCGCCGCTTTCTGCTGGGTGACGCGACTCGA
ACGGTCGGGATTCCCGACCGGGAGCTGCCTGTTTTCACTCTTGGCTTTGAGTTTGGTCATGATATGATGGGCGTG
TATCTTCCGACATTAGACGAGTGGGACGGCGTATTCGAGGAGGGAGGCTGGAGATGCGTCAAGAAGCATGCTATC
GATTCCCTGTCCGTGAGCGTGGTGTTCGAGCTCGAGCTCGAGCACCACCACCACCACCACTGA
MSTEVSEAQARRAVADIFNSTLASSAIGAAWELGALDELRENGKLDVSDFAVRHDLHEPAVVGMFTALASVGIVR
REGATVVVGPYFDEANHHRSLFHWLNQGSGELFRRMPQVLPNENRTGKFYQRDAGAISYACREISERYFDPAFWA
AVDGLGYTPTTVADLGSGSGERLIQIARRFPGVRGLGVDIADGAIAMAEKEVAAKGFGDQISFVRGDARTIDQVS
ARGEFAEVDLLTCFMMGHDFWPRENCVQTLRKLRAAFPNVRRFLLGDATRTVGIPDRELPVFTLGFEFGHDMMGV
YLPTLDEWDGVFEEGGWRCVKKHAIDSLSVSVVFELELEHHHHHH*

Wild type 6 (I1D222)

ATGACCACCACGGACAACGGCAGACGCGAATTATCTGTGGCAGACATGTTCAACTCAACTGTTGCGGCGGCCGCT
TTAGGTGCGGCGTGGGAGCTCGGGGCCCTGGACGAATTACACAGAGTTGGTGCATTAGATGTAGATTCATTCGCG
ACAGAGGGTGAGCTTCACCTGCCATCGGTGGAAGAGATGTTCCGTGCGCTGGCCTCAGTGGGGATTGTTGAGAGA
GATAATCGTAAGGTGCGTCCGGGACGCGCGTTCGCGGCTGCCTACCGAGCGAAGCCTTTGTTTTACTGGCTGTGG
CAAGGCAGTGGCGAGCTGTTTGCCAGAATGCCGGCCTTACTTCGGAATGCTAACCGGATTGGTACGTTTTATCAT
CGCGATGCAAAAGCCGTCTCATACGCGTCCCGCCAAGCTAATGCCACGTTCTTCGATCCAGTATTTCGGCAAGCG
ATGCGTCCCCTGGAAGGTAGATTTTCCACCGTAGTCGATTTAGGGTCGGGCTCAGGTGAACGGGTGATCCAGATA
GCCGAGGAAAACCCAGCAGTTCGTGCCTATGGGTTGGACATTGCAGACGAAGTGGTGGCGATGGCCAACGAGGAA
GCAGAGCGGAGAGGACTGGCGGATCGCGTGACCTTCTTAACTGCTGACGTACGGGAGTTGTCTCCTCGTCCGGAA
TTCGCCGGTACGGAGTTGTTAACGTGTTTCATGATGGGCCACGATTTGTGGCCCAGAGACGAGGCCATAGTTTCA
CTGCGGCGTTTAAGAGAGCTTTTCCCCAATGTTCGTAGATTTCTGTTAGGAGACACCGTTCGCACAGTCGGGATC
CCTGACGAGGAAATTCCTGTTTTCACTTTAGGTTTTGAGCTGGGCCATACTCTTATGGGACAGTATCTCCCGACT
GCCGAGGAGTGGCGGGGTGTCTTCGCAGAGGGTGGGTGGAGATGCGTTGGCGAACACGGCTCAGACGTTCTCGCC
GGAACAGTGGTCTTTGAGCTTGAACTCGAGCACCACCACCACCACCACTGA

MTTTDNGRRELSVADMFNSTVAAAALGAAWELGALDELHRVGALDVDSFATEGELHLPSVEEMFRALASVGIVER
DNRKVRPGRAFAAAYRAKPLFYWLWQGSGELFARMPALLRNANRIGTFYHRDAKAVSYASRQANATFFDPVFRQA
MRPLEGRFSTVVDLGSGSGERVIQIAEENPAVRAYGLDIADEVVAMANEEAERRGLADRVTFLTADVRELSPRPE
FAGTELLTCFMMGHDLWPRDEAIVSLRRLRELFPNVRRFLLGDTVRTVGIPDEEIPVFTLGFELGHTLMGQYLPT
AEEWRGVFAEGGWRCVGEHGSDVLAGTVVFELELEHHHHHH*

Wild type 7 (Q96565)

ATGGACAAAATCTCGGCACCGTTCTTCAGTGGTACTAGTCCTGCAGCGGCTTCTGTTGCCGGGGTAGATGAAGAC
GATAGACTGTGTTTCCAGGCCCAAGAACTCATGTTTGCATACAACATTAGTATGGTCCTGCGTGCGGCTATTCAA
TTGGGTCTTTTAGACGCACTCAGTGCTGCGGGCGGAAAGGCGCTGACACCGAACGAGTTAGTTGAAAACGTCGAG
ACTTCTTCAAATAAAGCAGAAGCAGCCGCCGCTGTTGACAGAATCTTACGCTATTTGTCTTGCTTCAACGTGGTT
ACATGTTCCAGTGAAGCCGCGGGCCCAGACGGGACTCTGGTTCGACGCTATACAACGGGCCCATTATGCCGCTGG
CTGACAAAGGACCGTGGTGACGGCACGTTAAGTCCATTCGCCGTCTTCGTGGTGGATCCAGACCATCTCTTCCCG
TGGCACCACATCGCAGAGGCCGTCACCGCGGGTGGTCCATCGGCATTCGAGAGAACTCAGAAATGGCCATACTAT
GAGTATATGGGAAAGAATCAGCGCCTGGGTACGCTGTTCGATAACGCCATGGCGCAGCACTCCGTAATTTTGGTA
ACTAAGATGCTTGAAAGATTCAAGGGGTTCGATGGTGTTCAACGGCTGGTGGACGTAGGCGGCGGCACAGGTTCT
ACGCTCGGCATGATTACGAGCAAGTACAAGCACATGACCGGTATAAACTATGATCTGCCCCATGTAATTGCTCAA
GGCCTGCCTTTACCAGGAGTCGAGCACGTAGCAGGGGATATGTACGAGAGCATACCGACGGGTGACGCAGTATTA
TTGCAATGGATCACGCTTATGCTCAATGATGACGAATTCGTGAAGATTTTATCGAACTGCCACAATGCACTCCCC
AAGGACGGGAAAGTTATTGTCGTTGATGGAATTTTGCCAGAGAACCCAGACTCGTCACTTACAGCCCGCGATGCA
TTCACGCTGGACATCATAATGTTCGTACTGTTCAAAGGTGCCAAACAACGCACGGAAAAGGAATTTGCACGCCTG
GCCAAACAAGCAGGTTTCACGGGCGGAATAAAGAAGACGTACATTTTCTTCAATTTCTACGCCTTGGAGTTCACC
AAGCTCGAGCACCACCACCACCACCACTGA

MDKISAPFFSGTSPAAASVAGVDEDDRLCFQAQELMFAYNISMVLRAAIQLGLLDALSAAGGKALTPNELVENVE
TSSNKAEAAAAVDRILRYLSCFNVVTCSSEAAGPDGTLVRRYTTGPLCRWLTKDRGDGTLSPFAVFVVDPDHLFP
WHHIAEAVTAGGPSAFERTQKWPYYEYMGKNQRLGTLFDNAMAQHSVILVTKMLERFKGFDGVQRLVDVGGGTGS
TLGMITSKYKHMTGINYDLPHVIAQGLPLPGVEHVAGDMYESIPTGDAVLLQWITLMLNDDEFVKILSNCHNALP
KDGKVIVVDGILPENPDSSLTARDAFTLDIIMFVLFKGAKQRTEKEFARLAKQAGFTGGIKKTYIFFNFYALEFT
KLEHHHHHH*

Wild type 8 (Q9SCP7)
ATGGAGAATGAAAGCAGCGAGAGCCGCAACCGCGCGCGACTCGCGATTATGGAGCTGGCCAATATGATCTCTGTC
CCTATGTCGCTCAATGCCGCAGTACGCTTAGGAATCGCAGACGCCATTTGGAATGGCGGAGCCAACTCTCCGTTA
TCGGCGGCGGAGATACTGCCGCGTCTTCACCTGCCGTCCCATACTACCATAGGCGGAGATCCGGAGAATTTGCAA
CGCATTTTACGTATGTTAACAAGCTATGGAGTGTTCTCGGAACACCTGGTCGGGTCCATTGAGCGGAAGTACAGT
CTTACCGATGTGGGGAAGACGTTAGTAACCGATTCAGGTGGTTTGTCCTACGCTGCATATGTCTTGCAACACCAC
CAAGAGGCTCTGATGCGTGCGTGGCCCCTTGTTCATACAGCGGTTGTAGAGCCTGAAACGGAGCCTTACGTCAAG
GCAAACGGCGAGGCGGCCTATGCACAATACGGCAAGTCAGAGGAGATGAATGGACTTATGCAAAAGGCGATGTCC
GGGGTTTCAGTCCCTTTTATGAAAGCTATCTTAGACGGGTACGATGGTTTCAAATCGGTGGACATTCTCGTTGAC
GTGGGCGGCTCCGCAGGCGACTGTCTTCGCATGATCTTACAGCAATTCCCAAACGTACGGGAAGGGATAAATTTT
GACCTGCCGGAAGTTGTTGCAAAAGCCCCAAATATACCGGGTGTTACCCACGTTGGTGGCGACATGTTTCAGTCT
GTACCAAGTGCCGATGCAATATTTATGAAATGGGTATTGACGACGTGGACAGATGAGGAGTGCAAGCAGATTATG
AAGAATTGCTACAACGCGTTGCCAGTGGGTGGGAAGTTAATTGCATGTGAGCCGGTTCTGCCGAAGGAAACCGAC
GAAAGTCATAGAACGCGGGCGTTGTTAGAGGGAGACATTTTCGTAATGACAATATACCGTACTAAGGGCAAGCAT
CGAACGGAAGAAGAGTTCATTGAGCTTGGTTTGAGTGCGGGCTTCCCCACTTTCAGACCCTTTTACATCGACTAT
TTCTACACCATATTAGAATTTCAAAAGCTCGAGCACCACCACCACCACCACTGA

MENESSESRNRARLAIMELANMISVPMSLNAAVRLGIADAIWNGGANSPLSAAEILPRLHLPSHTTIGGDPENLQ
RILRMLTSYGVFSEHLVGSIERKYSLTDVGKTLVTDSGGLSYAAYVLQHHQEALMRAWPLVHTAVVEPETEPYVK
ANGEAAYAQYGKSEEMNGLMQKAMSGVSVPFMKAILDGYDGFKSVDILVDVGGSAGDCLRMILQQFPNVREGINF
DLPEVVAKAPNIPGVTHVGGDMFQSVPSADAIFMKWVLTTWTDEECKQIMKNCYNALPVGGKLIACEPVLPKETD
ESHRTRALLEGDIFVMTIYRTKGKHRTEEEFIELGLSAGFPTFRPFYIDYFYTILEFQKLEHHHHHH*

Wild type 9 (Q6K9X3)
ATGGGCGGTGGTGGGGATGGCGAACTTAGTCCGGCCGAGGCTAGACTTGCAATGATGGAACTTGCTAACATGATA
TCGGTTCCCATGGCCTTGACAGCCGTGATACGCTTAGGTGTCCCGGCTAAGCTGTGGGCAGGCGGTGCTAACGCC
CCTTTGGCTGCCGCGGATTTGCTTCCAGCGGGTCATCCAGACCCTAGTGTTTTGGAGCGTCTCTTGCGTTTACTT
GCTAGCCGGGGCGTTTTCAGTGAACACACGGGCAGCTCCAGCCCATCTCCACGTCGCTTCTCCCTGACCGCAGTT
GGTCGAACGCTGGTCCCTGGTGGTGGTGGAAGTCCATCTGGATCAGGCGCCTCTTATGCAGACTACGTATTACAA
CACCACCAAGACGCTCTGGTCCGCGCCTGGCCTTTATTGCACGAGGCCGTACTTGACCCGAGCGGTCCGGAGCCT
TTCGCGCGTGCTAATGCAGGAGTACCAGCTTACGCGTACTACGGCAAGGATCGTGAGGCGAACGAGGTGATGCTG
AGAGCAATGACCGGTGTATCGGAACCGTTCATGGAAGCACTTTTAGAAGGGTATGGAGACGGCGGTTTCGAGGGC
GTTTCTACCTTAGTAGACGTGGGCGGTTCTTCAGGTGCCTGCCTCGAGATGATCATGCGGCGCGTACGGACTATC
CGCGACGGGGTAAACTTTGATCTTCCGGACGTCGTGGCAGCCGCCCCGCCGATTCCCGGTGTTAGACACGTAGGT
GGCGATATGTTCAAGTCCATCCCTAGCGGGGACGCTATCTTTATGAAATGGGTCCTTACAACGTGGACCAATGAG
GAGTGTACGGCGATACTTAGCAACTGTCACAAGGCTCTGCCAGGTGGTGGAAAGGTGATTGCCTGTGAACCTGTG
GTGCCGGATACTACCGACGGCAGTACCCGTACACGCGCCCTTCTGGAGAACGACATCTTTGTTATGGCCACCTAC
CGGACTCAAGGCCGTGAACGATCTGAAGAGGAATTTCGTCATCTGGGCCTGGCCGCAGGGTTTGCGAGTTTTAGA
GCTATTTACCTGGACCCGTTCTACGCAGTCTTGGAGTACACAAAGCTCGAGCACCACCACCACCACCACTGA

MGGGGDGELSPAEARLAMMELANMISVPMALTAVIRLGVPAKLWAGGANAPLAAADLLPAGHPDPSVLERLLRLL
ASRGVFSEHTGSSSPSPRRFSLTAVGRTLVPGGGGSPSGSGASYADYVLQHHQDALVRAWPLLHEAVLDPSGPEP
FARANAGVPAYAYYGKDREANEVMLRAMTGVSEPFMEALLEGYGDGGFEGVSTLVDVGGSSGACLEMIMRRVRTI
RDGVNFDLPDVVAAAPPIPGVRHVGGDMFKSIPSGDAIFMKWVLTTWTNEECTAILSNCHKALPGGGKVIACEPV
VPDTTDGSTRTRALLENDIFVMATYRTQGRERSEEEFRHLGLAAGFASFRAIYLDPFYAVLEYTKLEHHHHHH*

Wild type 10 (I1M2U5)
ATGGAGAAGGAAGAATCCACAGAGCAAAGAAAGCAAGCGCGTCTTGCGATCATGGAGCTCGCTAATATGATTTCA
GTTCCTATGGCATTAAATGCGGTTGTACGTCTTAATGTTGCTGACGCTATCTGGCAAGGTGGTGCCAACAACCCG
CTGTCTGCCGCAGAGATTTTGCCGCGCTTATTACCGGCGGGTGGTGGTGACGCAGAGAACCTGCAACGACTGCTT
CGAATGTTGGCATCGTATGGCGTTTTCTACGAGCACCTTAGCGCCGGGGAGCGCAAGTACTCGTTGACTGATGTG
GGAAAGACCCTCGTCACAGACGAACAAGGTTTGAGCTATGCGCATTATGTCCTGCAGCACCACCAAGATGCACTG
ATGCGGGCGTGGCCCATGGTGCACGAGGCCGTAGTAGACCCTACTAAGGAACCTTTCGAGCGCGCTAACGGTGAG
CCAGCATACGGCTATTACTTGAAGCACCCTGAGATGAATGATTTGATGGTACGGGCTATGAGCGGAGTTTCTGTT
CCCTTTATTCGGGCAATGTTAGAGGGCTACGACGGCTTCCAAGGAGTGGAGAAATTAGTCGATGTTGGCGGCAGT
GGTGGCGACTGTTTGAGAATGATTCTGGAGAAGCACCCAACCATAAAGGAAGGGATCAACTTCGATTTACCCGAG
GTTGTAGCTAAAGCACCCCAAATTCCGTTTGTTACTCATGTGGGCGGCGACATGTTCAAGTTTATACCACAGGGC
GATGCTATATTCATGAAATGGGTCCTTACAACGTGGACCGACGAGGAGTGTAAGCACATAATGCAGAATTGTCAC
AAGGCTCTCCCTGAAGGTGGGAAGCTCATTGCGTGTGAGCCGGTACTGCCTGAGGACTCGGACGAGTCACATAGA
ACGCGTGCACTGCTTGAAGGGGACATATTTGTCATGACCATATACCGAGCTAAGGGCAAACACAGAACTGAGGAG
CAGTTTCGCCAGTTAGCGATAGATGCGGGTTTCCCGCGTTTTAGAGCTTTTCACGTGGACCATTTCTATACTGTA
CTCGAGTTTCAAAAGCTCGAGCACCACCACCACCACCACTGA

MEKEESTEQRKQARLAIMELANMISVPMALNAVVRLNVADAIWQGGANNPLSAAEILPRLLPAGGGDAENLQRLL
RMLASYGVFYEHLSAGERKYSLTDVGKTLVTDEQGLSYAHYVLQHHQDALMRAWPMVHEAVVDPTKEPFERANGE
PAYGYYLKHPEMNDLMVRAMSGVSVPFIRAMLEGYDGFQGVEKLVDVGGSGGDCLRMILEKHPTIKEGINFDLPE
VVAKAPQIPFVTHVGGDMFKFIPQGDAIFMKWVLTTWTDEECKHIMQNCHKALPEGGKLIACEPVLPEDSDESHR
TRALLEGDIFVMTIYRAKGKHRTEEQFRQLAIDAGFPRFRAFHVDHFYTVLEFQKLEHHHHHH*

Wild type 11 (A9AWD7)
ATGACAGTATTAAGCACGGACATACCAGCCCCAAACTCTCCTTCAATCTCCGACGTTGAGGCCTACTATGATGCC
ATGGGGCCCTTTTACAAGCTTATATGGGGAGACAGTGTTCACGGCGGCTATTGGCCCGCAGGACTGGAAGATATG
TCTTTACCAGAGGCTCAAGAGCACCTGACTAATTTAATGATCGAAAAGACGCCAATTAAGCCGGGCCAGCATATG
CTTGACTTAGGCTGTGGGACAGGGCTGCCAGCGATCAGAATGGCTAGTGCTAAGCAATGTCACGTTCACGGCTTG
ACAGTCGCCCATGGGCAAGTTGCAGAAGCTCAAGCTACTATACAAGCTATGCAAATGCAAGAGTTGGTGCATATT
AACTGGGGAAACGCAATGGAACTGCCTTTCGAGGCCGACTTCTTCAATGCGGCTTGGGCTTTCGAGTCCATTTTC
CACATGCCATCGCGTCTTACCGTCTTGCAAGAGGCGAACCGTGTGCTTCAAGCGGGTAGCTACTTTGTTTTGACG
GATATTGTGGAAGTAAAATCCCTGAGTCCGGAGCAACAGCAGATTTTCTTCCCTGCCTTCCAAATTAACACATTG
ACAACTAAGCAGGGTTACTTAGACTTGTTTGCGCAGACCGGATTCGAGCAATTAGAATTAATTGACCTTACGGCA
GGAATCGAAAAGACCTTGGCACACACAAAGCTCGGGATCGAGCAGAAACGTGCTGAGCTGGCTGCTATTTACCCG
CCAGAGATGCTGGGCATGATCGAGCAAACCTGGCCCATGGTTGAGAAGATCTATGCCGAGTTCGTCCGCTATGTC
CTGATAGTGGCCAGAAAGCGGGGCCTCGAGCACCACCACCACCACCACTGA
MTVLSTDIPAPNSPSISDVEAYYDAMGPFYKLIWGDSVHGGYWPAGLEDMSLPEAQEHLTNLMIEKTPIKPGQHM
LDLGCGTGLPAIRMASAKQCHVHGLTVAHGQVAEAQATIQAMQMQELVHINWGNAMELPFEADFFNAAWAFESIF
HMPSRLTVLQEANRVLQAGSYFVLTDIVEVKSLSPEQQQIFFPAFQINTLTTKQGYLDLFAQTGFEQLELIDLTA
GIEKTLAHTKLGIEQKRAELAAIYPPEMLGMIEQTWPMVEKIYAEFVRYVLIVARKRGLEHHHHHH*

Wild type 12 (Q65YP0)
ATGTCTACCACAGCGAGATACGATTCAATAGGCGGACTGTTCGAGGACTTCACTCAAAGCGCGGCGCAGAGAGCA
ATCGAAGTACGGACTATATTCCATATGATTGGAGACGTCTCAGGAAAATCCGTCCTTGATCTTGCATGTGGTTTC
GGGTTCTTTGGACGGGAGATTTACCGCAGAGGCGCCGCAAAGGTCGTTGGTGTAGATATTTCCGAAAAGATGATT
GAGTTGGCACGGGAAGAGTCCCGCAAGTACGGCGACCCTCTTGAGTTCCACGTCCGCGATGTTGCTAACATGGAG
CCACTCGGCCAATTCGATCTTGTTAATGCCGCATGGCTTTTCAACTACGCTGACTCAGTGGAAAACCTTCGCAAG
ATGTTTAAGGTTGTGCGCGCTTCCCTTAAGCCGGACGGTAAGCTTGTTGCGTACACGGTCGACCCGGACTTTTCC
CTCGCGAAGGGAAACTTTGCAAAGTACGGGGTGAATGTATTGAACGAGAGAGCTTGGGGTCCAGGATATCGCCAT
GATGCGGAGTTCGTAACTGACCCACCCTCACAGTTTTCTTTCTACAGATGGTCGCGCGCTGACTACGAGTCTGCA
ATCGCAGACGCCGGGTTCAGTCATTTCGAATGGCAAAAGCCGTTGCTTGAGGCAGATGACATAGCCACGCACCCG
CCTGGCTTCCGCGACGTGTTTCAAAACAACTGTTTACAGACTGGACTTGTGTGCAAACCACTCGAGCACCACCAC
CACCACCACTGA

MSTTARYDSIGGLFEDFTQSAAQRAIEVRTIFHMIGDVSGKSVLDLACGFGFFGREIYRRGAAKVVGVDISEKMI
ELAREESRKYGDPLEFHVRDVANMEPLGQFDLVNAAWLFNYADSVENLRKMFKVVRASLKPDGKLVAYTVDPDFS
LAKGNFAKYGVNVLNERAWGPGYRHDAEFVTDPPSQFSFYRWSRADYESAIADAGFSHFEWQKPLLEADDIATHP
PGFRDVFQNNCLQTGLVCKPLEHHHHHH*

Wild type 13 (*dre*-H-NMT, Q6DC37)
ATGGCAGCACCTTTCAAGACTTTGGTTGAGGATTATCCACGTTATCTTAAGTCGTTCGAGCTTTTCTTAGAGCGT
TCGTCTGAACACCAATGCATGCAAGATTTCATCCACAACACATTGCCTGACATACTTGCGTCTATAGGCGGCGGT
CGTAGCGTATTCAATGTTATGGGAGTAGGTTCAGGTGCAGGGGACATCGACCTTGAGATGCTGGCACAGTTACAC
TTGAAGCACCCTCATGTAAAGGTAGATAATGAGGTGGTGGAGCCCTCAAACGACATGCTGTACAAATACAAGGCG
CGCGTTAGTACATCCCCGGACCTTGCATACATTAACTTCACCTGGAATAAGATGACAGCATCGGAATTTGAGAAA
CAATGGCAAGAGAAAACCCCCGAGAAGAAGATGGACTTCATCCATATGATACAAATGTTGTATTACGTGAAGGAT
CCTAACGCTACGGTCTCATTCTTCCGTAGCTTACTGGAGAAAGACGGGAAACTTCTCATCATACTGGTCAGTGGT
GAAAGCGGGTGGGGTAAGCTGTGGACTACATTTCGTAAGCAACTGTGTTATACAGAGATGTCACAATGCGTGACC
ATAGGAGAGATCAAATCATTCTTAGATTCCGAGGGTGTCCCGTATCGGAAGTACGTTTTACTTAGCCAAATGGAT
ATTACTGAATGCTTCACCGAAGGTGACCAAGAAGGTGAGCTGCTGCTTGACTTCTTAACAGAGGTTAAGGAATTC
TCAAAGAACGCACCCGAGCGCCTTAAGAAGGAAGTTCTGGACGTGCTGCGCCACCCGGACTGTAGTAAGGAAGTG
GACGGCCGGATCATCTTTAACAACAATTTAGAGGTCTTGGTAATTGAACCGCTCGAGCACCACCACCACCACCAC
TGA

MAAPFKTLVEDYPRYLKSFELFLERSSEHQCMQDFIHNTLPDILASIGGGRSVFNVMGVGSGAGDIDLEMLAQLH
LKHPHVKVDNEVVEPSNDMLYKYKARVSTSPDLAYINFTWNKMTASEFEKQWQEKTPEKKMDFIHMIQMLYYVKD
PNATVSFFRSLLEKDGKLLIIILVSGESGWGKLWTTFRKQLCYTEMSQCVTIGEIKSFLDSEGVPYRKYVLLSQMD
ITECFTEGDQEGELLLDFLTEVKEFSKNAPERLKKEVLDVLRHPDCSKEVDGRIIFNNNLEVLVIEPLEHHHHHH
*

Wild type 14 (Q0V9P1)
ATGGACTCTGGTTTGCGTTCTTTATTATCCGACCATTCTCGCTACGTAGAGAGCTTTCGGTTGTTTCTGCTTAAC
AGTACGGAGCACCAATGTATGCAACGGTTTATTGATACTCAATTTCCTCACATAGTAAGCTCAATCGGTAAGGAT
AAGTCGGTAATAGATATCCTTGGTATCGGGTCGGGGAGCGGGGAGATTGATTTGCAGATGATTGGAAAGATCCAA
TCCAGACATCCGGGTGTCGCGATTTCTAACCAAATAGTGGAACCTTCTGCCGAACAGATAATCGGCTACAAAGAA
CGTGTGGCCAAGGCACCAAATCTGGGCGCCGTCTCTTTTAGTTGGCACAGACAGACTAGTTCCGAGTATGAACGG
CAGGTTAACGAGGAAAAGCAAATGCGTTCTTACGATTTCATACACATGATTCAGATGCTTTATTATGTGAAGGAC
GTCCCAGCTACCCTTCGTTTCTTCAAGAGCTGCCTCGCACCTAATGGCAAGCTCCTGATAATCCTGGTTAGTGGT
AACTCTGGGTGGAGTATGTTATGGAAGAAGCATGGACCGCAATTACCATTAAACGACTTATGTTTGTACGTCACG
GCGGGAGACATCGCTCAGATGCTTAGCAGCATGGGAGCTCGATTTCAGTCGTATGAGCTGCCGAGCGACATGGAC
ATAACAGAGTGTTTCATTGAAGGTGACCGTAATGGTGAGATGCTCCTGGACTTCCTCACTGAAACTTGCGATTTT
AAGCGGAATGCCCCGGCCGACCTCAGAGAACAAATCTTATGCGACCTGAAATCACCGGAGTGTTCAACAACCCGC
GATGGTAAAGTAATTTTCAACAACAATCTGAGCGTAATAGTGGTGGAGAGAGACCTCGAGCACCACCACCACCAC
CACTGA

MDSGLRSLLSDHSRYVESFRLFLLNSTEHQCMQRFIDTQFPHIVSSIGKDKSVIDILGIGSGSGEIDLQMIGKIQ
SRHPGVAISNQIVEPSAEQIIGYKERVAKAPNLGAVSFSWHRQTSSEYERQVNEEKQMRSYDFIHMIQMLYYVKD
VPATLRFFKSCLAPNGKLLIIILVSGNSGWSMLWKKHGPQLPLNDLCLYVTAGDIAQMLSSMGARFQSYELPSDMD
ITECFIEGDRNGEMLLDFLTETCDFKRNAPADLREQILCDLKSPECSTTRDGKVIFNNNLSVIVVERDLEHHHHH
H*

Wild type 15 (Q91VF2)
ATGGCGAGCTGCATGCGCTCACTGTTTAGTGATCAAGGTCGTTATGTAGAGTCTTTTCGTCGGTTCCTGAATAAC
AGTACTGAGCACCAGTGTATGCAAGAGTTCATGGACAAGAAATTGCCTGGTATAATTGCTAGAATTGGTGAGGCC
AAGGCGGAGATCAAGATCCTCAGTGTCGGTGGTGGTGCAGGTGAGGTGGATCTGCAAATTCTGAGTAAAGTCCAG
GCACAGTATCCAGGTATTTGTATAAACAACGAAGTTGTCGAACCCTCTGCAGAGCAAATCGTGAAGTACAAAGAG
TTAGTGGCGAAGACTAGTAACATGGAGAATATAAAGTTTAGCTGGCACAAGGAAACGTCGTCCGAATACCAAAAG
CGAATGTTAGAGGAAGAGGAAGAACCGCCGAAGTGGGACTTCATCCACATGATCCAAATGCTGTACTATGTGAAG
GATATCCCTGCTACCTTAAAGTTCTTTCACGGCTTGCTGGCCGCTAGTGCCAAAATCCTGATCATACTGGTGTCC
GGCACATCGGGTTGGGAAAAGCTCTGGAAGAAGTACGGTAGTCGACTTCCTCGCGATGACCTCTGCCAGTACGTC
ACGTCGTCCGACCTCGCGCAAATCCTTGACGACTTAGGGATCAAATACGAATGTTACGATCTCGTTAGTACCATG
GATATCACGGATTGCTTCATCGACGGCAATGAAAACGGCGATCTTCTGTGGGACTTTCTGACTGAGACGTGCAAC
TTCTCAAAGACTGCACCACTGGATCTCAAGGCGGAAATTATGAAGGACTTACAAGAGCCCGAGTTTAGTGTAAAG
AAAGAGGGTAAGGTTCTGTTCAATAACAATCTTAGCTTCATTGTTGTAGAAGCGAACGTCCTCGAGCACCACCAC
CACCACCACTGA

MASCMRSLFSDQGRYVESFRRFLNNSTEHQCMQEFMDKKLPGIIARIGEAKAEIKILSVGGGAGEVDLQILSKVQ
AQYPGICINNEVVEPSAEQIVKYKELVAKTSNMENIKFSWHKETSSEYQKRMLEEEEEPPKWDFIHMIQMLYYVK
DIPATLKFFHGLLAASAKILIIILVSGTSGWEKLWKKYGSRLPRDDLCQYVTSSDLAQILDDLGIKYECYDLVSTM
DITDCFIDGNENGDLLWDFLTETCNFSKTAPLDLKAEIMKDLQEPEFSVKKEGKVLFNNNLSFIVVEANVLEHHH
HHH*

Wild type 16 (Q58DV7)
ATGGCTTCCTCGATGCGCAGTCTTTTCACTGATCACTCGCGCTACGTAGAGAGCTTCCGACGTTTTCTTAGTAAC
TCGACAGAGCACCAATGTATGCAGGAGTTCATGGACAAGAAGTTGCCCGGAATCATAGCCCGTATTGGGGATATT
AAGTCTGAAATCAAGATCCTGTCAATTGGTGGCGGTGCCGGGGAGATTGACCTGCAAATTCTTAGCAAGGTGCAA
GCCCAATACCCGGGAGTACACATAATCAACGAGGTGGTGGAGCCCAGCGCAGAGCAGATCACGAAATACAAGGAG
CTCGTGGCGAAGACGTCAAATCTCGAGAACATCAAGTTCGCTTGGCACAAGGAAACTTCCTCAGAGTACCAGAAT
CGGATGATGGAGAAGAAAGAGTTACAGCGTTGGGACTTCATCCACATGATACAGATGCTGTATTACGTTAAGGAT
ATCCCCGCAACTCTCAAATTCTTCCACTCCCTGCTGGCTACTAACGCGAAGATATTAATCATCATAGTTTCCGGA
GCGAGTTCATGGCAAAAGCTGTGGGAAAAGTACGGCAGCAGACTTCCTAGAAACGATCTGTGTCAGTACGTAACT
AGTAGTGACCTGACACAGATGCTGGATAAGTTAGGAATTAAGTACGAGTACTACGACTTGTTATCGACAATGGAT
ATCTCCGACTGCTTTATAGATGGAAACGAGAATGGTGATCTTCTGTGGGACTTTCTTACGGAGACTTGTAACTTC
AATACTACAGCCCCGCCGGACCTCAAGGCCGAAATAATGAAAGATTTACAAAAGCCAGAGTTCTCCATCAAGAAG
GAAGGTAAGGTCTTGTTTAACAACAGCCTGTCTTTCATAGTGGTTGAGGCTCTCGAGCACCACCACCACCACCAC
TGA

MASSMRSLFTDHSRYVESFRRFLSNSTEHQCMQEFMDKKLPGIIARIGDIKSEIKILSIGGGAGEIDLQILSKVQ
AQYPGVHIINEVVEPSAEQITKYKELVAKTSNLENIKFAWHKETSSEYQNRMMEKKELQRWDFIHMIQMLYYVKD
IPATLKFFHSLLATNAKILIIIVSGASSWQKLWEKYGSRLPRNDLCQYVTSSDLTQMLDKLGIKYEYYDLLSTMD
ISDCFIDGNENGDLLWDFLTETCNFNTTAPPDLKAEIMKDLQKPEFSIKKEGKVLFNNSLSFIVVEALEHHHHHH
*

Wild type 17 (Q9EST2)
ATGGCAAGTTCCATGCGTAGCCTCTTTAGCGACCATGGCAGATATTTCGAAGCATTCCGACGTTTCCTCAATAAC
TCCACTGAATATCAATGTATGCGTGAATTCATGGATAAGCAACTGCCGGGGATAATCGCGAGAATTGGTGGTTCT
AAGAGCGAAATCAAGGTATTATCCATAGGCGGCGGCGCCGGTGAAATGGACCTGCATATACTGAGCAAAGTAAAG
GCACAATACCCGGGTGTACACATTATAAACGAGGTTGTTGAGCCTAGCGCGGAACAGATTACGAAGTACAAGGAG
TTAGTCGCCAAAACCAGCAATCTTGAAAATATAAAATTCGCCTGGCACAAAGAGACTTCCTCTGAGTACCAAAAT
CGTGTCATGGAGCAAAAGGAGATCCAGAAGTGGGATTTCATACATATGATCCAAATGTTGTACTATGTTGATGAT
ATCCCGGCAACACTCAAGTTCTTCCACTCGTTGTTGGCTACGAATGCTAAAATACTTATAATATTAGTATCTGGC
AAGTCAGGGTGGTTAAAGTTCTGGAAGAAGTACAGATCGCGGCTTCCGCAAAATGATCTGTGTCAATATGTCACC
AGCTTCGACATCATCCAAATGCTGGACTCGCTGGGTATAAAATACCAGTGTTACGATCTCCTTTCCACTATGGAC
ATCACGGATTGTTTCATTGATGGAAATGAGAACGGAGAGCTTTTATGGGACTTCCTTACCGAAACCTGTAATTTC
TTAACCACTGCGCCACCCGATCTCCGCGCAGAGATTATGAAAGATCTCCAGGGCCCCGAATTTATTGTGCGTAAA
GAGGGAAAGATTCTGTTCGACAATTCTCTTAGTTTCATAACGATTGAGGCACTCGAGCACCACCACCACCACCAC
TGA

MASSMRSLFSDHGRYFEAFRRFLNNSTEYQCMREFMDKQLPGIIARIGGSKSEIKVLSIGGGAGEMDLHILSKVK
AQYPGVHIINEVVEPSAEQITKYKELVAKTSNLENIKFAWHKETSSEYQNRVMEQKEIQKWDFIHMIQMLYYVDD
IPATLKFFHSLLATNAKILIIILVSGKSGWLKFWKKYRSRLPQNDLCQYVTSFDIIQMLDSLGIKYQCYDLLSTMD
ITDCFIDGNENGELLWDFLTETCNFLTTAPPDLRAEIMKDLQGPEFIVRKEGKILFDNSLSFITIEALEHHHHHH
*

Wild type 18 (Q5U4V2)
ATGGACTCAAAGCTTCGTAGCCTGTTAAGTGATCACAGCCGATATGTTGAGTCTTTCAGACTGTTCCTGCAAAAT
AGTACGGAGCACCAATGCATGCAACACTTCATAGAGTCTAAACTTCCTAACATAATTTCTTCAATTGGAAACGAC
AAGCCCGTTATCGACGTTTTAGGTGTTGGATCCGGTAGTGGTGAGATTGACTTGCAAATGATAGCTAAAATTCAG
GCCCGTTGGCCTGGCGTGCCGATTAACAACCAAATCGTGGAGCCAAGCGCTGAACAAATCTTCGGATATAAAGAG
AGAGTTGCCAAAGCTCCCAATCTTGAAAATGTGACATTCTCGTGGCACCGCCAAACAAGCTCTGAGTTTGAATCT
CAGGTGAACGAGGACAAGCAGATGCGCAAGTTCGACTTTATTCATATGATACAAATGCTCTACTATGTAAAGGAC
GTGTTAGGAACATTGAAGTTCTTCAAGTCATGCCTTGCACCATCTGGCAAGCTCTTAATTATACTTGTTTCAGGT
AATTCTGGCTGGGCTACTCTCTGGAAGAAGTATGGGCAACGATTGCCCCTGAACGATTTGTGTTTGTATATAACA
GCAGGCGACATAGCAGAGATGCTTAGTTCCATGGGTGCTCGATTTCAGAGTCATGAGCTGCAATCAGACATGGAT
ATTACCGAGTGCTTCATCGAAGGGGACCGGGACGGCGAGTTGTTACTCGACTTCTTGACGGAAACCTGCGATTTC
AAGCGTAACGCGCCTGCTGATCTTCGGGACCAAATAATTTGCGATTTAAAGAGCCCAGGCTGCAGTACCACAAAG
GATGGAAAAGTGATCTTTAATAATAATCTGTCAGTCATTGTAGTAGAGGCGGATCTCGAGCACCACCACCACCAC
CACTGA

MDSKLRSLLSDHSRYVESFRLFLQNSTEHQCMQHFIESKLPNIISSIGNDKPVIDVLGVGSGSGEIDLQMIAKIQ
ARWPGVPINNQIVEPSAEQIFGYKERVAKAPNLENVTFSWHRQTSSEFESQVNEDKQMRKFDFIHMIQMLYYVKD
VLGTLKFFKSCLAPSGKLLIIILVSGNSGWATLWKKYGQRLPLNDLCLYITAGDIAEMLSSMGARFQSHELQSDMD
ITECFIEGDRDGELLLDFLTETCDFKRNAPADLRDQIICDLKSPGCSTTKDGKVIFNNNLSVIVVEADLEHHHHH
H*

Wild type 19 (Q4SBY6)
ATGGCATCGGCGATGCGTAGCTTAATCGAGGATGATAGCCGGTACCTCAAGAGCTTCAAGTTGTTTCTGGAACGC
AGCACCGAGCACCAATGTGTCCAGGAGTTCATCCACAACGTATTGCCCGATATTCTTGGCAGCGTTGGGAAGGGC
AAGACCCATTTGAACGTGATTGGTGTCGGGAGCGGTGCGGGTGAAGTGGACTTAGAAATACTTAGTGAATTACAC
TCACGCCACCCTGGTGCGTCCGTTGACAACGAGGTTGTGGAGCCAAGTGCCCAGCAATTGCAAGACTATAAGGCA
CTGGTTTTACAAAAGAAGGATCTGGATTATATTAGTTTCAACTGGAATAAGATGACAGCCACCGAGTTCGAAGAG
AGATGGCGGGCTAACAAAATGTCCAAGGAAGCTGATTTCATACACATGATTCAAATGTTATACTACGTGAAAGAC
CCTGAGGCAACTACTAGCTTCTTCCAATCTTTACTTTCAAAGACCGGCAAGCTTCTGATCATCCTGGTCAGCGGC
AACTCGGGTTGGGGTAAATTATGGAAGACTTATAAGAATCAGTTCTGCAATCCGGAGATCTCACAGTGCGTTACC
ACTGCAGATATCCAATCTTTCTTAGACAGTAAGGGTGCGCGCTATCAATCCTTCGAACTGCCCAGTCAGATGGAC
ATAACAGAGTGCTTTACGCAGGGAGACGAGAAGGGTGAGTTGTTATTAGACTTTCTGACAGAAGTACTTGAGTTC
AGCAAGACTGCGTCACCGGAGCTTCGAGCAGAAGTAATGGAATTACTGCGCCACCCTGACTGCTCCGTGGAGTCG
AATGGCCGAGTCATGTTCAACAACAACCTTGGCGTGATCGTGCTCGACCTCGAGCACCACCACCACCACCACTGA

MASAMRSLIEDDSRYLKSFKLFLERSTEHQCVQEFIHNVLPDILGSVGKGKTHLNVIGVGSGAGEVDLEILSELH
SRHPGASVDNEVVEPSAQQLQDYKALVLQKKDLDYISFNWNKMTATEFEERWRANKMSKEADFIHMIQMLYYVKD
PEATTSFFQSLLSKTGKLLIILVSGNSGWGKLWKTYKNQFCNPEISQCVTTADIQSFLDSKGARYQSFELPSQMD
ITECFTQGDEKGELLLDFLTEVLEFSKTASPELRAEVMELLRHPDCSVESNGRVMFNNNLGVIVLDLEHHHHHH*

Wild type 20 (A0A2U4AJA4)
ATGGCTTCCTCTATGAAAAGCCTGTTCTCTGATCACAGTCGCTACGTAGAGTCATTTCGACGTTTTCTGAACAAC
TCTACCGAGCACCAATGCATGCAGCAGTTCATGGATGAGAAATTACCCGGGATTATTGCGCGGATTGGCGACACG
AAATCAGAGATAAAGATCCTTAGCATAGGCGGTGGAGCTGAGCTGGTAGCTAAGACTTCAAACCTGGAGAATATA
AAATTCACGTGGCATAAAGAGACATCTTCGGAATACCAAAACCGCATGATGGAGAAGAAGGAATTACAGAAGTGG
GATTTCATACACATGATTCAGATGCTCTACTATGTCAAGGATATTCCAGGCACGTTGAAGTTCTTCCACAGTCTG
TTAGCGACTGGGGCAAAGATCCTCATTACTCTCGTCTCGGGTACATCTGGGTGGGACAAGCTTTGGAAGAAGTAC
GGCTCACATCTCCCTCGGGACGATCTGTGCCAATGCGTCACTTCTTCGGACTTGATGCAAATGCTTGACAAGCTT
GGGATCAAGTACGAATGCTACGACCTGTTGAGTACTATGGACATCTCCGACTGTTTCATTGACGGGAACGAGAAT
GGCGATCTCCTGTGGGACTTTCTGACTGAGACTTGGAATTTTAGTACCACTGCTCCACCTGATCTTAAGGCAGAA
ATCATGAAGGACTTGCAGGAGCCTGAGTTCTCAGTCAAGAAAGAGGGTAAAGTTCTCTTCAATAACAGCTTATCT
TTTATTGTTGCTGAGGCACTCGAGCACCACCACCACCACCACTGA

MASSMKSLFSDHSRYVESFRRFLNNSTEHQCMQQFMDEKLPGIIARIGDTKSEIKILSIGGGAELVAKTSNLENI
KFTWHKETSSEYQNRMMEKKELQKWDFIHMIQMLYYVKDIPGTLKFFHSLLATGAKILITLVSGTSGWDKLWKKY
GSHLPRDDLCQCVTSSDLMQMLDKLGIKYECYDLLSTMDISDCFIDGNENGDLLWDFLTETWNFSTTAPPDLKAE
IMKDLQEPEFSVKKEGKVLFNNSLSFIVAEALEHHHHHH*

Wild type 21 (O55239)
ATGGAATCAGGTTTCACCAGCAAGGATACATACCTGAGTCACTTTAACCCTCGCGATTATCTCGAAAAGTACTAC
TCGTTCGGCTCCCGTCATTGTGCAGAGAACGAGATCCTTAGACATCTTCTGAAGAACCTGTTCAAGATCTTTTGT
CTCGGCGCAGTTAAGGGCGAGCTTTTGATTGACATTGGAAGTGGCCCCACTATCTACCAGTTACTGAGCGCGTGC
GAAAGCTTTACTGAGATCATCGTATCGGACTATACAGACCAGAACCTGTGGGAACTTCAGAAATGGCTCAAGAAG
GAGCCGGGTGCCTTCGATTGGTCCCCCGTCGTTACCTACGTATGTGACCTCGAAGGCAACCGAATGAAGGGACCG
GAGAAAGAGGAGAAGCTTAGACGAGCAATTAAGCAAGTGCTGAAGTGCGACGTGACGCAATCCCAACCGTTAGGC
GGGGTTTCACTCCCGCCGGCCGATTGTCTCCTGTCGACGCTCTGTCTTGACGCCGCTTGTCCCGATCTTCCGGCT
TACAGAACAGCGTTAAGAAATCTTGGTTCACTGCTTAAACCCGGTGGATTCTTGGTAATGGTGGACGCGCTGAAA
AGCTCGTATTACATGATCGGTGAACAAAAGTTCAGTTCGTTGCCGCTGGGCTGGGAGACGGTGCGGGACGCTGTA
GAAGAAGCTGGATATACGATCGAACAATTCGAGGTTATCTCCCAGAATTACTCATCAACGACCAGCAATAACGAA
GGCCTGTTCAGTCTCGTGGGTCGTAAGCCGGGTCGGTCCGAGCTCGAGCACCACCACCACCACCACTGA

MESGFTSKDTYLSHFNPRDYLEKYYSFGSRHCAENEILRHLLKNLFKIFCLGAVKGELLIDIGSGPTIYQLLSAC
ESFTEIIVSDYTDQNLWELQKWLKKEPGAFDWSPVVTYVCDLEGNRMKGPEKEEKLRRAIKQVLKCDVTQSQPLG
GVSLPPADCLLSTLCLDAACPDLPAYRTALRNLGSLLKPGGFLVMVDALKSSYYMIGEQKFSSLPLGWETVRDAV
EEAGYTIEQFEVISQNYSSTTSNNEGLFSLVGRKPGRSELEHHHHHH*

### Wild type 22 (P34254)

ATGACAGGTACGACTGAGAACGATAACACTGGAGAAGATGAGAAGCCGAAAGACGAAGAATGTGCAGCTATCGAG
CACAAGGACAAGTTCAATCCAACAGCATACTTAAATTCTTTCTACAAGACGGCTTCTGAAGACACTGCCATGCAA
ATCGTGTTGTTCTTCCTGCCCGGAATACTTTACCGCTTACCGCAGAAGGTGCGCTCCGTTCTTGACCTCGGCGCG
GGACCAACTGTATATCTCCCCATTAGCTTACGTGACCGTGCTGAGAATATTTATACTTCCGACTATGCTCCTGCA
AATCGGGACACATTAATCAACTGGATAGAAGATAAGAGCGATTTTGACTGGGACAACGTTTGTTCCTGGATAGCC
AACATCGAAGCATCTATGGAAACGGGTAAGCAAATGCAAAATAAGACTCGCAAGCTCATGCGGGCAGTTCTGGAC
GTTAACGTACATGAAAGCCCGGTAGTTCAGTCCATAGTTTGGAAGGAGAACGAGCAGGTGCAAGTTCCTGATAAA
TTCCAAGTCGTGAGTACAGTATTCTGTCTGGAGTATAGTTGCGAAACGCTTGAGGCGTATTTTAGAGCAGTACGA
TCGGCATGTTCATTGATTGATGAGGGCGGTATCTTAATTCAAGGTGGTGTACTCGACGCAACCACATACAACTTC
GGCGGCAAGACCTTCAGATGTCACCGGCTTAAGCAAGCACATATAATTGAGAGCCTGAAGGCTAACGGGATGGCG
ACGACAGCGGAGCAAGGCTACAAGTTTATAACGCACGATGACATATTCTTATTAGTCTCAAAGAAGTTACTCGAG
CACCACCACCACCACCACTGA

MTGTTENDNTGEDEKPKDEECAAIEHKDKFNPTAYLNSFYKTASEDTAMQIVLFFLPGILYRLPQKVRSVLDLGA
GPTVYLPISLRDRAENIYTSDYAPANRDTLINWIEDKSDFDWDNVCSWIANIEASMETGKQMQNKTRKLMRAVLD
VNVHESPVVQSIVWKENEQVQVPDKFQVVSTVFCLEYSCETLEAYFRAVRSACSLIDEGGILIQGGVLDATTYNF
GGKTFRCHRLKQAHIIESLKANGMATTAEQGYKFITHDDIFLLVSKKLLEHHHHHH*

### Wild type 23 (P40935)

ATGAACGGCGGATCCGACCTTAAACATGCAACGGGAAGTGGATCTGACCCAAAGCACGCAGCCGAGATGGACCCA
GACAGCGATGCGGGACAAGTAGCAGTAGCTTTAGCATACCAACGGTTCGAACCACGCGCATACTTGAGAAACAAC
TACGCGCCCCCGCGCGGTGACTTATCTAATCCAGACGGAGTTGGTCCGTGGAAATTACGGTGTATGGCGCAAGTA
TTCGCTACAGGCGAGGTAAGCGGACGGGTATTAATTGATATCGGGAGTGGCCCAACCATTTATCAACTCTTATCT
GCCTGCGCCCACTTTGAAGACATAACCATGACCGATTTCTTGGAAGTTAATCGGCAAGAGCTGGGATTATGGTTA
CGGGAAGAGCCTGGTGCATTCGACTGGTCAGTATACTCGCAACATGCATGTTTAATTGAGGACAAGGGTGAGTCG
TGGCAAGAAAAGGAACGGCAGTTACGCGCCCGTGTTAAGCGTGTGCTCCCTATCGACGTGCACAAGCCGCAACCA
CTCGGCACTCCGTCGTTAGTTCCCTTACCTGCCGACGCCCTGGTCTCTGCTTTCTGCTTAGAGGCAGTATCGCCT
GATCTGACAAGCTTCCAAAGAGCTCTGCACCATATTACGACTCTTTTACGGCCGGGCGGCCACCTGCTTCTGATT
GGAGCGCTCGAGGAATCTTGGTACTTAGCCGGTGAGGCACGTTTATCAGTCGTGCCCGTAAGCGAAGAGGAAGTC
CGTGAAGCGCTCGTGTTAGGCGGTTACGAGGTACGTGAGCTGCGCACTTATATCATGCCCGCACACCTGTGTACT
GGTGTAGACGACGTAAAGGGGATATTCTTCGCATGGGCACAGAAGATGGAAGTGCAAGTTCTCGAGCACCACCAC
CACCACCACTGA

MNGGSDLKHATGSGSDPKHAAEMDPDSDAGQVAVALAYQRFEPRAYLRNNYAPPRGDLSNPDGVGPWKLRCMAQV
FATGEVSGRVLIDIGSGPTIYQLLSACAHFEDITMTDFLEVNRQELGLWLREEPGAFDWSVYSQHACLIEDKGES
WQEKERQLRARVKRVLPIDVHKPQPLGTPSLVPLPADALVSAFCLEAVSPDLTSFQRALHHITTLLRPGGHLLLI
GALEESWYLAGEARLSVVPVSEEEVREALVLGGYEVRELRTYIMPAHLCTGVDDVKGIFFAWAQKMEVQVLEHHH
HHH*

### Wild type 24 (P10938)

ATGTCAGGTACTGACCGGTCGCAGGCAGCTGGGGCTGTCCCGGATAGCGACCCTGGTCTCGCAGCTGTGAGTTCC
GCTTATCAACGCTTCGAACCGCGTGCATATCTGCGCAATAATTACGCGCCCCCTCGCGGTGACTTAAGCTGTCCC
GACGGTGTAGGACCGTGGAAGCTTCGGTGCTTGGCGCAAACATTCGCGACAGGAGAGGTCTCCGGTCGGACCTTA
ATCGACATAGGGTCGGGTCCTACCATATATCAATTGTTATCGGCTTGCGCACACTTTGAGGATATAACAATGACG
GACTTCTTAGAGGTAAACCGTCAAGAACTCCGCTTATGGTTACGGGAAGAGCCTGGAGCCTTTGATTGGTCGGTT
TACTCGCAACACGTCTGCTTGATCGAAGGTAAAGGTGAATCTTGGCAAGAGAAGGAGTGTCAACTGCGCGCACGC
GTTAAGCGGATATTGCCTATAGACGTGCACCGGCCACAACCGCTGGGTGCTGGTGGACTCGCGCCTTTGCCGGCC
GACGCCTTGGTATCGGCCTTCTGTTTAGAAGCAGTCAGTCCAGATTTAGCATCATTTCAACGCGCCCTGGACCAC
ATTACGACCCTGCTCAGACCTGGTGGCCACTTACTTTTAATCGGGGCTCTTGAGGAGTCCTGGTATTTAGCGGGT
GAGGCTCGATTGGCAGTCGTGCCTGTCCGTGAAGAGGAAGTGCGTGAGGCTCTGGTGCGCACAGCGACACGGTGC
GGAATCTGTGCACGAACACCCATGCCGGCTCACCTGCAAACCGGGGTCGACGACGTTAAGGGTATATTCTTCACG
CGTGCGCAGAAGAAGGTGGGCGTTCTCGAGCACCACCACCACCACCACTGA

MSGTDRSQAAGAVPDSDPGLAAVSSAYQRFEPRAYLRNNYAPPRGDLSCPDGVGPWKLRCLAQTFATGEVSGRTL
IDIGSGPTIYQLLSACAHFEDITMTDFLEVNRQELRLWLREEPGAFDWSVYSQHVCLIEGKGESWQEKECQLRAR
VKRILPIDVHRPQPLGAGGLAPLPADALVSAFCLEAVSPDLASFQRALDHITTLLRPGGHLLLIGALEESWYLAG
EARLAVVPVREEEVREALVRTATRCGICARTPMPAHLQTGVDDVKGIFFTRAQKKVGVLEHHHHHH*

### Wild type 25 (O95050)

ATGAAAGGCGGTTTTACTGGTGGAGACGAGTACCAAAAGCACTTCTTGCCTCGGGACTACTTAGCAACTTATTAC
TCGTTCGACGGCAGTCCCTCACCTGAGGCGGAAATGTTAAAGTTCAACCTCGAGTGTTTACACAAAACATTCGGC
CCTGGCGGGCTGCAAGGAGACACCTTGATAGATATCGGCTCTGGCCCTACGATTTACCAGGTCCTGGCGGCGTGT
GACAGCTTTCAAGACATAACATTGAGCGACTTCACAGACCGGAACAGAGAGGAGTTAGAGAAGTGGCTCAAGAAG
GAGCCCGGCGCATACGACTGGACGCCTGCAGTGAAGTTTGCGTGCGAGTTAGAGGGGAACAGCGGACGGTGGGAA
GAGAAGGAAGAGAAGTTAAGAGCTGCCGTTAAACGGGTGTTAAAATGCGATGTACACTTAGGCAACCCACTGGCT
CCGGCGGTCTTACCACTTGCTGACTGTGTCTTAACACTGCTCGCGATGGAATGTGCTTGCTGTTCGTTGGACGCT
TACCGCGCAGCATTGTGCAACCTGGCTTCATTATTGAAGCCCGGCGGCCACCTGGTAACAACTGTTACACTGCGT
CTTCCCTCTTACATGGTCGGTAAGAGAGAATTCTCTTGCGTGGCTCTCGAGAAAGAGGAAGTGGAGCAAGCGGTC
CTTGACGCCGGGTTCGACATCGAGCAATTATTGCACTCACCTCAAAGTTACAGCGTAACAAACGCAGCCAACAAC
GGAGTTTGCTTCATCGTGGCTCGCAAGAAGCCGGGTCCTCTCGAGCACCACCACCACCACCACTGA

MKGGFTGGDEYQKHFLPRDYLATYYSFDGSPSPEAEMLKFNLECLHKTFGPGGLQGDTLIDIGSGPTIYQVLAAC
DSFQDITLSDFTDRNREELEKWLKKEPGAYDWTPAVKFACELEGNSGRWEEKEEKLRAAVKRVLKCDVHLGNPLA
PAVLPLADCVLTLLAMECACCSLDAYRAALCNLASLLKPGGHLVTTVTLRLPSYMVGKREFSCVALEKEEVEQAV
LDAGFDIEQLLHSPQSYSVTNAANNGVCFIVARKKPGPLEHHHHHH*

### Wild type 26 (P40936)

ATGGAAGGCAAGGTCTATATTGGCGGCGAAGACTATGAGAAGGAATTTACACCGAAGGACTACCTGACGACCTAC
TATTCATTCCACTCGGGGCCGGTCGCCGAACAGGAAATTGTAAAGTTCAGTTTGCAAAACTTATACCAAACGTTC
AGTACCGGTGGGGTCGGCGGAGATGTTTTAATTGACATTGGGTCCGGGCCGACAATCTACCAACTTCTCAGTGCC
TGCGAGGTGTTTCGGGAGATTATTGTGACAGACTACACTCCGCAAAATTTACAAGAACTTCAAAAGTGGTTAAAG
AAGGAGCCAGGCGCTTACGACTGGTCTAGTATAGTGCAACATGCATGTGAGCTTGAGGGAGACCGCTCGCGTTGG
CAAGAGAAAGAGGCCAAGTTACGACGCACCGTTACACGGGTTCTTAGATGCGACGTAACGAAGACACCACCACTT
GGTTCCGCCCAGGTGCCCCTGGCTGATTGCGTTCTTACGTTCCTGGCTATGGAGTGTGCTTGTCCGGACATTGAC
ACATACCGCGCTGCTCTCAGACGTTTAGCTGGGTTGCTGAAGCCTGGCGGTCATTTAGTGACACTCGTTACCTTG
CGTTTCCAACATTACATGGTTGGTCCAAAGAAGTTTAGTGGTGTCTACTTGGAGAAGGAAGTTGTTGAAAAGGCC
ATACAAGACGCCGGCTGTCAAGTATTGAAGTGCAACTGTGTCTCATTAAGCTATTCGGAAGCATACTGTTCCCAT
GACGGACTGTGCTTCGTGGTAGCTCGTAAGGGCCCCAGTGCGCTCGAGCACCACCACCACCACCACTGA

MEGKVYIGGEDYEKEFTPKDYLTTYYSFHSGPVAEQEIVKFSLQNLYQTFSTGGVGGDVLIDIGSGPTIYQLLSA
CEVFREIIVTDYTPQNLQELQKWLKKEPGAYDWSSIVQHACELEGDRSRWQEKEAKLRRTVTRVLRCDVTKTPPL
GSAQVPLADCVLTFLAMECACPDIDTYRAALRRLAGLLKPGGHLVTLVTLRFQHYMVGPKKFSGVYLEKEVVEKA
IQDAGCQVLKCNCVSLSYSEAYCSHDGLCFVVARKGPSALEHHHHHH*

### Wild type 27 (A0A4R4Y3T3)

ATGGATTTACCTACATATTGGCAAAGTAACTACCAGGATCCACACGGTGACGACCTTTCATTCGTTGCCATGATT
GGTGCCTTTATGGTCGATCACCTGCAAGGTAGATCCGATCTGCAAGGAGTTGATGTGGGTACAGGTGCTAACCTT
TACCCGGCTTTCTCTATGCTGCCATTCTGCTCTGCGTTAACTCTCATCGACTTAAGTCCAAGCTCAATTGACTGG
CTGACAGGTGAGCTGGGCGGGTCACTGGCGGCATGGACACCGTTCTGGGACGAGTTTGCTAAGATACCAGCCTAC
GCAGCAGCAGGGCCATTACATGAGGCTATCGGCGCGCGTACAAGAATTGAGATAAAGCAAGCGAGCGTGTTCACG
CTCCCGGATGAACAGTGGGATGTCGGAACCATGTTCTTTGTCGCAGAATCGATTTCCGGTGACCGCGAGGAATTT
AGAGCAGCTCTGGCGCGGTTTGTAAACGCCCTGAGACCCGGCGCGCCGTTCGCGGTTGCATTCATGGAAAACTCG
GAAGGTTGGCGGGTTGGCGACCAAGACTTCCCGGCCGTCGCCGTGAGTATCGCCGACGTCATCTCAAGCATGCCC
TATTGTACACAACTCCACCACGTAAAGCGTTTATCAGCTGAGGAAGAACCCATTCGGCCAGGCTACACTGGGATG
ATCCTGGCGTGCGGGATAAAACGTAGTACTGAGTCGCTCGAGCACCACCACCACCACCACTGA

MDLPTYWQSNYQDPHGDDLSFVAMIGAFMVDHLQGRSDLQGVDVGTGANLYPAFSMLPFCSALTLIDLSPSSIDW
LTGELGGSLAAWTPFWDEFAKIPAYAAAGPLHEAIGARTRIEIKQASVFTLPDEQWDVGTMFFVAESISGDREEF
RAALARFVNALRPGAPFAVAFMENSEGWRVGDQDFPAVAVSIADVISSMPYCTQLHHVKRLSAEEEPIRPGYTGM
ILACGIKRSTESLEHHHHHH*

Wild type 28 (A0A0X3XHI7)
ATGAACGCTGACGTACCGTGGGATGCGGCCTTCGATTCGATTGCCTACCTGGACCATAACTACCGCGCTCTCCAA
GCTGAAGACGCCGAAATACTCCACATCATTCGCGATCATTTCGGCGACCACTTTCGCAAGCAGGGCGCGGGCCCT
GTCTCTGGTATAGACGTTGGGGCCGGTGTCAATCTGTATCCAGCACTTGCAATGATGCCTTGGTGCGACGAAATT
ACGCTCTTCGAGCGTAGCGTATCCAACGTAGACTATTTGACCAGCCAAATAGAAGGATACGATGAGACTTGGGAC
CCGTTCTGGAGTGCCCTGTGTCTCAAGGATTGTTACCGAAGCTTGGATATGGACCCGCGAGAACGTTTCCGCAAG
GTCGTGCGTGTTGAGCAAGGAGACATATTTGACTTATGGCGTCATGAAGGAAGATGGTCGGTTGGAACCATGTTC
TTCGTTGCAGAATCAATGACAGCTGTGCACGAGGAATTCGCACTTGGCGTCGAACGTTTCATGAGAGCCCTCGCT
CCCGGAGCCCCGTTCGCGACGGCTTTTATGGCCCACTCAAAAGGTTACCACGCTGGCGAGCACTTCTTCCCGGCA
TGTGACGTCGGAGAGTCAGAGGTCCGGGCATCTTTGGAAGCTTTCGCAGGTGATTTTAAGGTCCAGAGACTTGAG
AGCGCCGCCGCAGTTCGTGACGGGTACAGCGGTATGATAGTTGCGTACGGTTGGCGCGCCAACTCTGATAGTACC
ATACCAGTAGGTCTCGAGCACCACCACCACCACCACTGA

MNADVPWDAAFDSIAYLDHNYRALQAEDAEILHIIRDHFGDHFRKQGAGPVSGIDVGAGVNLYPALAMMPWCDEI
TLFERSVSNVDYLTSQIEGYDETWDPFWSALCLKDCYRSLDMDPRERFRKVVRVEQGDIFDLWRHEGRWSVGTMF
FVAESMTAVHEEFALGVERFMRALAPGAPFATAFMAHSKGYHAGEHFFPACDVGESEVRASLEAFAGDFKVQRLE
SAAAVRDGYSGMIVAYGWRANSDSTIPVGLEHHHHHH*

Wild type 29 (A0A0L8QTT5)
ATGGACTTAACGTCGGAGGGAGGAACTCTTTGTCTCAATAGAGACGCTCCGTGGGACAGTTTCGACTCACAGTCT
TACACGCGTCACAACTATAGCGTTTTACGTCCCGACGATGAGGAAATTATTAACGTTGTGCGCGACTATTTCGCT
GACCATTTTCGGACAAACCCAGAGCGACCCGTCAGCGGCATAGACGTGGGTGCAGGGGCAAACTTGTATCCTGCA
TTAAGCCTTTTACCCTGGTGCGATGAGATTACTTTGTACGAAAGATCCGCCCGAAACGCCGCATGGTTGCGAGAT
CAGGTACCACACTACGGGCGGTCGTGGGACCCCTTCTGGCAAGTTCTTCGGAAGGAAGAGGAATACCAAGACATA
GCCGACCCTCGTGCCCGATTTGCAGACAGTGTTACCGTTGAGGAAGGCGACCTGTTCCGTCTCCCGCATCGCCTT
CGCCATCAGGGTATTGGAACCATGTTCTTCGTTGCGGAGTCTCTTTCAACTTCGCACCGTGAATTTGAGGGAGCT
GTGGCAGCTTTCCTCGGTAGTTTAGCCCCTGGTGCGCCGTTCGCCGCTGCTTTCATGGAAAATTCAGGTGGTTAT
TTGGTTGGTACGGAACTCTTCCCAGCATGCGAGGTCGATTCCGCTGAGGTTGAAGCGAGTTTGGAGCCTCACGCA
ACGGTGGATGCGCTCGAGCGTTTCGGGCTGCCTGAAGGCCCGGTCAGAGAGGGTTATACGGGCATGATCTTGGCG
TGTGGTCGTCGTGATCCGGCCCTCGAGCACCACCACCACCACCACTGA

MDLTSEGGTLCLNRDAPWDSFDSQSYTRHNYSVLRPDDEEIINVVRDYFADHFRTNPERPVSGIDVGAGANLYPA
LSLLPWCDEITLYERSARNAAWLRDQVPHYGRSWDPFWQVLRKEEEYQDIADPRARFADSVTVEEGDLFRLPHRL
RHQGIGTMFFVAESLSTSHREFEGAVAAFLGSLAPGAPFAAAFMENSGGYLVGTELFPACEVDSAEVEASLEPHA
TVDALERFGLPEGPVREGYTGMILACGRRDPALEHHHHHH*

Wild type 30 (A0A821K491)
ATGGATTCCAGCACGTATAAGCGGTACCATGAGGACGATTTTGATTCTCGACAATCACTCGAGGACTATGTATCC
GATAAGCCTGATATGGTCTTCGCTGAGGACACGCTCATATTCCCGATTGAGAACCTGACCAAGACATTTACGGAA
GGGCATGTCAAGGGTGACATCTTGATAGACCTTACCGTTGGGTCAATGGTTCACCACTTATTCGCGGCCTGTGAG
TTCTTCAAACATATAATAGTTCTGAAGATGCGGGACCGGTGTATAATGGAACTGAAAAGATGGGTTGACAGTCGC
ACGGGAGCGTTCGAGTGGTGCCATGCGACGAAGTTACATGTAGACCGCGCAGGCAAGTCAGACCAACTTGAGGAT
AAAGAGGGTAAGGTGCGTTCGGCGCTGCAGCACGTAGTTAAATGTGACCTGAACAAGGAGAACATGATGGACCCA
ATCGTGTTGCCGCCCGCTGACTGCGTCATCTCGGGATGGTTACTTGATTATATTTGTAAGAACCAAGAGGACTTT
ATACGATACCTGCGGAAATTCTCCAGTCTGTTAAAGCCAGGTGGCCGATTTGTACTCATCGGTTGTCTTGAGATG
TCTAGTTTTATTGTTGGCAAGGACAAGTTTCACGCGTTTTCTTACAACGAGGATTTCGCGCGAAAGGCATTGGTA
GGCGAAGGATTTATAATCGACCGGTGCGAGACCAAGAAACGGACAGTGGTTTCAGACCTGACCGATTATAAGGGC
ATGATCTTTATTGCGGCCCATAAGGACACTCAAGTGCTCGAGCACCACCACCACCACCACTGA

MDSSTYKRYHEDDFDSRQSLEDYVSDKPDMVFAEDTLIFPIENLTKTFTEGHVKGDILIDLTVGSMVHHLFAACE
FFKHIIVLKMRDRCIMELKRWVDSRTGAFEWCHATKLHVDRAGKSDQLEDKEGKVRSALQHVVKCDLNKENMMDP
IVLPPADCVISGWLLDYICKNQEDFIRYLRKFSSLLKPGGRFVLIGCLEMSSFIVGKDKFHAFSYNEDFARKALV
GEGFIIDRCETKKRTVVSDLTDYKGMIFIAAHKDTQVLEHHHHHH*

Wild type 31 (B7Q5F2$)
ATGCCAGCAATGTCTGAGCGTAAACGGAGTCTCTTAGATGTTGGTTGCGGTCCGATCACTTGTAACGTCTTTCCG
GCGACCAAGAGCGTGCAAGATGTAGTTCTGAGCGACTTCCTGTTAGGCAACCGTTTAGAAGTAGAAAAGTGGCTC
AAGGGCGCACCGGACGCGATAGACTGGAGCTGTTACAGTGAGTCATTGGCCCGTTTAGAGGGTTTTAGCGACATA
AAGCGTGGAGCTGAGGAGATAACTGCACGCACTCGTAAGGCTATAAGAAAGGTGGTACCCGGTGATGTGTTGGTT
CCGGGTGTTTTACAGAAAGAACATCAGGAGAAGTTCGACGTGGTTTTATCTTGTTTCTGCCTGGAAGCCGCGTCC
CTGGACGAGGCAACATTCCGCACAGCGACACAGAACGTGGGCGACTTAGTGCATGAGGGTGGTATTCTGATCTTA
TGTGGTGTGCTGGGTCGCCATCAGTTCTCAGTGGGTGGGGTAAAATTCCCATGCTTGTGCTTAACATCCGACGCA
GTAAAGGACGCAGTGGTGAGAGCGGGATTCCGTGTGAACTTATGGCGCAGCCTGTATAAGCCAGGACCTCTGACC
CCGACCATAGGAAGCGCGTACGTCGTTGCGGCAGAGAAGCTCCTCGAGCACCACCACCACCACCACTGA

MPAMSERKRSLLDVGCGPITCNVFPATKSVQDVVLSDFLLGNRLEVEKWLKGAPDAIDWSCYSESLARLEGFSDI
KRGAEEITARTRKAIRKVVPGDVLVPGVLQKEHQEKFDVVLSCFCLEAASLDEATFRTATQNVGDLVHEGGILIL
CGVLGRHQFSVGGVKFPCLCLTSDAVKDAVVRAGFRVNLWRSLYKPGPLTPTIGSAYVVAAEKLLEHHHHHH*

Wild type 32 (C3Z5R9)
ATGGCTGCACCTATTCAACGCGGGACCGACCATCACAAGACCTTTGGAGCCAAAGCGTACCTGGCATTATACTAC
GCGACGCCGGAAGGGACGGACGAAGAGGGCGAGCTGCTGACTCCCTATTTAAAGGAATTCCACGAGATTTTCAAT
AGTGGACGGTTAAAGCCCGGGTCACGTTTATTGGATGTTGGTTCGGGCCCGACCATCCATCAGCTTATCAGCGCC
TCTCGCTTCTGTACTGAGATCGTGTGTGCGGAATACACCGAGAACAACCGCGCAGAAATCGAGAAGTGGGTCAAG
AAGGATCCTGACATGCACGACTGGACCCCATTCTTCAAATTCGTAGCCGATCTTGAGGGCGACAGCTCGTCATGG
GAAGCGAGACAAAGCCATCTGCGTGACGCAATTAAGGAAGTTATTACCTGTGATGTTACCAAGCCCGAGCCATTT
GCCCCACGACAATATCAGGAATTCGATGTTATTACGACTTCCCTTAGTCTCGAAACAGCATGCCCGGATCGCGAG
ACTTACTCAGCAGCTGTTAGAAACATCACCCGCCTTCTGAAGCCAGGCGGCACCTTCGTCTTAATAGGTGTTACC
AACCAAACTTTCTATACCGTTGGCGGATATAAGTTCTTCACATTGCCTATTGATTCTAGCTTTATGCGCGAGGTC
TTCGAGAAAGCAGGTTTTGTGGACATTAATATAAAATCATTCCCAGCTACGAATCCGGAAAACAATACGATCTCC
GACTTTGACGGCTTCGTGGTCCTGCACGCGTGTAAGGCGGAAATCCTCGAGCACCACCACCACCACCACTGA

MAAPIQRGTDHHKTFGAKAYLALYYATPEGTDEEGELLTPYLKEFHEIFNSGRLKPGSRLLDVGSGPTIHQLISA
SRFCTEIVCAEYTENNRAEIEKWVKKDPDMHDWTPFFKFVADLEGDSSSWEARQSHLRDAIKEVITCDVTKPEPF
APRQYQEFDVITTSLSLETACPDRETYSAAVRNITRLLKPGGTFVLIGVTNQTFYTVGGYKFFTLPIDSSFMREV
FEKAGFVDINIKSFPATNPENNTISDFDGFVVLHACKAEILEHHHHHH*

Wild type 33 (S4N1R9)
ATGGACTGGGACTCATGGCCGGTGGCCGACTACTTACGAGAAAATTACCGTGAGGTGCACCCAAGTGATGCGGCG
ATAATCGCCCATCACTCAGCGTTTTACCGGAGATTCCGACCAGGAGCAATCGCGCGTTCCGTGGAGTTTGGCGCT
GGGCCGAACCTGTACCCGCTGATACTTGCAAGTGCAGTCTCCCGGCGTATTGACGCGGTGGAAGCTGGTGCATCA
AATGTCGCATATCTTCGCGACCAAATCTGTCACCGTCTCGACGCCTCTTGGCTTCCATTCCATGAACTCTGCCGT
CGACTTAATCCGGACGTGCCAGCAACTCTTGCTGGAGCACTTGCGCCGGTCAACGTTGTACATGCGGACGTTAGA
ACTTTACCGGCTGGGTCCTACGAGTTGGCATCTATGCACTTCGTGGCTGAGGGTGCGACAGAGGATTTCGCCGAG
TTTGCAGATTTCTGCCGGACATTTGTGCGAACAGTGGAGCCAGGTGGTCACTTAGTTGCCGCTTTAATGGAGAAT
ATGCCGACCTACCGCATTGGACCAGCGAGTCGCTGGCCCGGGTGCCCTGTCGACCCCGCCACAGTCACAGAGGTC
TTTACGCCTCTGACTCGCGATCTCGATGTGACGCATATCGATACAGACCCTACACTTCCTGACTACGGTGATAGC
GGGATGGTCTTGTTAACAGCTGTTGCCGAAGCTGTGCTCGAGCACCACCACCACCACCACTGA

MDWDSWPVADYLRENYREVHPSDAAIIAHHSAFYRRFRPGAIARSVEFGAGPNLYPLILASAVSRRIDAVEAGAS
NVAYLRDQICHRLDASWLPFHELCRRLNPDVPATLAGALAPVNVVHADVRTLPAGSYELASMHFVAEGATEDFAE
FADFCRTFVRTVEPGGHLVAALMENMPTYRIGPASRWPGCPVDPATVTEVFTPLTRDLDVTHIDTDPTLPDYGDS
GMVLLTAVAEAVLEHHHHHH*

Wild type 34 (B5I9B3)
ATGGACACCGTGACGACGACTCATAATGACGATGTCGATTGGGACAGATGGCCAGTCGCTGACTATCTGGCGGAA
AACTACAGAGAACTCCATCACGCGGATGCGGAAGTAATTGCGCACCACTCTGCATTTTATCGCCGATTGCCGCCC
GGCGGAATTGCTCGAAGCGTAGAGTTCGGCGCTGGCCCGAATCTCTATCCACTGATTTTAGCTAGTGCTGCATCC
CGGCGTGTGGACGCCGTGGAAGCTGGTGCGGGGAACGTCGCCTACCTGCACCGACAAATCGTATGTGGACCCGAC
GCGTCGTGGTTACCTTTCGACGCGTTGTGCCGCAGATTAAATCCTGACCTGCCTCGTACCCTTGCCGCTAGCTTG
GCACAAGTGCAAGTGATTCACGCGGACGTGCGTGAGTTGGAGCCAGGCGACTACGAGCTGGCCTCGATGCACTTC
GTAGCAGAGGGAGCGACTGAGGATCGTGCTGAGTTCGCCGACCTTTGCCGTGCGTTCGCACGGTGCGTTGTGCCA
GGTGGATACCTCGTTGCGGCTTTTATGGAGAACATGCCCACATACCGAATCGGGCCAACCTCTAGATGGCCAGGT
TGTCCTGTCTCACCGGCAACAGTGACAGAGGTATTCGCTCCCCTGACCCGGGAGCTGTCTGTTACACGGGTCGAT
ACCGATCCAACGCTCCCAGACCATGGTGATTCAGGTATGGTATTATTGACGGCAGTAACGGCTGATAGAAGAGGC
CTCGAGCACCACCACCACCACCACTGA

MDTVTTTHNDDVDWDRWPVADYLAENYRELHHADAEVIAHHSAFYRRLPPGGIARSVEFGAGPNLYPLILASAAS
RRVDAVEAGAGNVAYLHRQIVCGPDASWLPFDALCRRLNPDLPRTLAASLAQVQVIHADVRELEPGDYELASMHF
VAEGATEDRAEFADLCRAFARCVVPGGYLVAAFMENMPTYRIGPTSRWPGCPVSPATVTEVFAPLTRELSVTRVD
TDPTLPDHGDSGMVLLTAVTADRRGLEHHHHHH*

Wild type 35 (A9JTQ8)
ATGTCGGGTACCGAACAAAAGTATTACTTAGACAAGGAGCTGGACCCGTGCCTGCATTTTGACACATACCTTGGA
AGTGAAATTACTTCAGCCAAGAAGGAGATGTTAGAGGATCCTCTTAGCTTCCTTTACAAGCTCTTCTCGTCAGGC
AGTGTGAAGGGCGAGACACTTATAAACATCAGTATCTCCGCTGATGTGTCCAAAACTTTCGTGGCTGCCGACTTC
TTTAAGCATATTGTATTACTGGAGTCCTCTGATAGTTCAATGAAGGCCATCGAATCGTGGATACGGAACGAGCCA
GGGGCCGAGGACCAGAGCTACGCTGCTGAATTCGTCTGTTCTCTGAAAGGTCAATCCACGGGCAGCAAGGAACAA
GAAGAGAAAGCACGCCGGGCCATCAAGCAAGTAGTAAAATGGGACTTAACCGAGGAAAACCCATTAGGTGCTGTT
GAATTACCACAAGCCGACTGTATTGTAACAGTCTACCACATGGAAGCTATTTGTAAGGATAACGACATGTATATC
AATGTCCTGAAGAAATTACTTAGCCACCTTAAGATCGGCGGCCACCTGGTGATGATGGCGGGAATCAACATGACC
TACTACATGGTTGGCCAATACAAATTTGCTGCGCTTAATCATAACGAAGAGTTCATGCAGAAGGCTGTAACCGAG
GCAGGGTGCACGGTAGTTTCTGCGGAGACACATATTCGTAAATTCGAGAGTCCCTTGATAGACTACGAGTCAATA
GCATACCTCGTATGCCGCAAGGATCGCGTTGTTCTCGAGCACCACCACCACCACCACTGA

MSGTEQKYYLDKELDPCLHFDTYLGSEITSAKKEMLEDPLSFLYKLFSSGSVKGETLINISISADVSKTFVAADF
FKHIVLLESSDSSMKAIESWIRNEPGAEDQSYAAEFVCSLKGQSTGSKEQEEKARRAIKQVVKWDLTEENPLGAV
ELPQADCIVTVYHMEAICKDNDMYINVLKKLLSHLKIGGHLVMMAGINMTYYMVGQYKFAALNHNEEFMQKAVTE
AGCTVVSAETHIRKFESPLIDYESIAYLVCRKDRVVLEHHHHHH*

Wild type 36 (A0A816AWZ6)
ATGTGCGACTATTCGACAGACAACTGTTTGCTTCACACGACGGACTACAAGTCACGTTTCGACACCGTAAGTTAC
CTTCAAAATTTCTACAGTGGAGTTGACATCAATCCTTCTGAAGCACCGTTGGCGTCATTCTTCCTGGAAAAGACA
GTTCAAATTCTGCATGATGAGCGAAACCGTTTCGGCAACCAGAAGGTGTTGGAGTTTGGTGGTGGACCGAATTTA
AGCGCGAGTCTTCTGTTGGCCCAATACGCAGACAGCATAAGATTCTGTGACTATACCCAGTCTAACTTAAATTAT
GTAACGGATTGGATAGAACAAAAGTCCACGGTTTTCGACTGGACGAACTACTTTGAGTCGGTGCTCACTATCGCT
GGCACTAGCAAGGAAAAGCGTATAGAGTGGGAATCACGCCTGCGAGACGCTCTGTCTCGCGGCGGTCTGAGCATC
TGTGACGTTAATGACCCGGGGTGTCCGATCTTGAGTGGCAAGAGTGACGATTACGACATAATTTTCAGCAGCTTC
TGTCTTGAGGCAGCATGCCTGACTATAGACATATTTAATGAAACAATAGGTAAACTCGTGCGCTTGCTGAAGCCA
GGTGGCCTTTTACTCCTTGTCATGGTCCGGAATGAGTCCTTTTATTATGTTGGTGCGGAAAAGTTCTTCTGCCTG
AGCTTAGATGAAGCGAAAGTCAAGAAGGCATTGCAAGCAACAGGTGAGCTGGTGGACATTCATATAGATTCTGTA
GACACTCGGGTTGAAGACCAAGAGAGAAACACCATGAGTGACTTTGATGGTGAGATGATAATACATGCTTTTAAG
ACGATTGAACTCGAGCACCACCACCACCACCACTGA

MCDYSTDNCLLHTTDYKSRFDTVSYLQNFYSGVDINPSEAPLASFFLEKTVQILHDERNRFGNQKVLEFGGGPNL
SASLLLAQYADSIRFCDYTQSNLNYVTDWIEQKSTVFDWTNYFESVLTIAGTSKEKRIEWESRLRDALSRGGLSI
CDVNDPGCPILSGKSDDYDIIFSSFCLEAACLTIDIFNETIGKLVRLLKPGGLLLLVMVRNESFYYVGAEKFFCL
SLDEAKVKKALQATGELVDIHIDSVDTRVEDQERNTMSDFDGEMIIHAFKTIELEHHHHHH*

Wild type 37 (Q53CN2)
ATGGAATCGGCCGACGGAGAGAATCTGAAACAAGCCATTCTTGACAGAGACACCTTCCACAAGGAGTTCAATACG
GAAGCGTACTTGAAGGACTTCTATACTAAAGTTGAGGATTCTGCAATGCAGATGGTGTTGATTTTCCTGCCCAAC
ATTGTCGCGCGCATTGGCAAAATCAAGCGCGTGCTGGACTTCGGTGCGGGGCCTACTATCCACGTTGCGGCGAGC
TTCCGCAACCAAGCGGTTGAGATCTATTTAGCAGACTACCTGCCTCAAAACCGACAAGAATTAATGCGGTGGTAT
GAGGGATCATCAAGCTTCGATTGGAGTCATCCTATGAAGATGATCCTCACGCAAGAGGGCAACCCATGGAACGAT
CTTGATGAAATGATCCGTATAACGCGTCAGAAAGTGCGCGGGATCTTTCACTGTGACTGCTTTTCATCTCCGTCA
GTTGACGTGAGCGAGCAATTGCAAGGCATGTTTGACACAGTAGTGACCATCTTTTGCGTAGAATACTGTTGTAAG
ACATACGATGAGTATAAGAATGCAATCAAGAATATCACCCAGCAAATACGCGAGGGTGGATTCTTAGTGATGGGT
GGCATTCTTGAAGAGACGTGGTGTTCATTCGGCGGTCGCATGTTCTCATGTCTTTATATCACAAAGGAGATGATG
CTGAACGCGATCGAAGAGGCTGGCTTGCGCATAGAAAATGACTGCAAATGCATTATGTACAACATTGACAACATG
TTCATGCTGTGTGCGCGTAAGGCGAACACAATATCTGACAATACGTCACTCGAGCACCACCACCACCACCACTGA

MESADGENLKQAILDRDTFHKEFNTEAYLKDFYTKVEDSAMQMVLIFLPNIVARIGKIKRVLDFGAGPTIHVAAS
FRNQAVEIYLADYLPQNRQELMRWYEGSSSFDWSHPMKMILTQEGNPWNDLDEMIRITRQKVRGIFHCDCFSSPS
VDVSEQLQGMFDTVVTIFCVEYCCKTYDEYKNAIKNITQQIREGGFLVMGGILEETWCSFGGRMFSCLYITKEMM
LNAIEEAGLRIENDCKCIMYNIDNMFMLCARKANTISDNTSLEHHHHHH*

Wild type 38 (W2TA20)
ATGTTTGAATTCCTTTTCTTTCGGGACAGTATCGAGAATGGTACTAGAATGTCGTTGTTTGCACTGCCCGTTTTC
GCGCACACCATTCAGCAGTCGCTGGCTCCGGAACAACGCGAAAGCTTACTTGATGTGGGCGCTGGGCCGACGGTT
TACTCAGCTCTTTGTTTCCGAGACGTTGTCAGACGGATTTACTTATCAGACTATCTGAGTAAGAACCTTGACGTG
CTTAAGCTGTGGCGTAATAACACATCAACTTACGACTGGAAACCGACCATCAAAGTCATTAGACGTACTGAAGGC
GGTCTGCCGTTATCAGAATCCGAGATGGACGAACTTGAGGAGAAAGCGCGCGCGGTTGTAAAGTGTGGCGGCATT
ATGTGCGCTAACGTCCACGACGACCCCGTAGTACCGGAATTGAAGGGAGAACAAGTAGACGTTTTAGTGTCGATA
TTCACCCTGGAGTCTGCGTGCCAAACGTATACCCAATATTGCCAGGCCGTCAAGAATATGATGAAGCATTTGCGC
TCGGGCGGACGCATAGTGTTAGGCAGCGTGCTCGAAGACCAGTCATACAACAGCGGAAAGGATGTTATCTTCCAC
CTGTTGCACCTCACGGAAGACCAGATCCTGAACGCCCTTGGCTCTGCCGGAATAAACCAAGACACTGTAAAGAAG
TATGTATTGAAAGAGGACGGAGTCATTTTCCTTATGGCCGTGAAGAACCTCGAGCACCACCACCACCACCACTGA

MFEFLFFRDSIENGTRMSLFALPVFAHTIQQSLAPEQRESLLDVGAGPTVYSALCFRDVVRRIYLSDYLSKNLDV
LKLWRNNTSTYDWKPTIKVIRRTEGGLPLSESEMDELEEKARAVVKCGGIMCANVHDDPVVPELKGEQVDVLVSI
FTLESACQTYTQYCQAVKNMMKHLRSGGRIVLGSVLEDQSYNSGKDVIFHLLHLTEDQILNALGSAGINQDTVKK
YVLKEDGVIFLMAVKNLEHHHHHH*

Wild type 39 (H0W3I3)
ATGGAAGGGCGGTTGTACGCTGAAGGTGAGAACTACAAGAAAGAGTTCAATCCAAAGGACTACCAAGTGACGTAC
TACGCTTTCGATTCAGGTGCCACAGCTGAGAATGAGATCCTGAAGTTCAATTTAGAAAATCTGTTTCAAATGTTT
ACGGCGGGTGGGGTCGGCTCCAACGTGTTAACTGACATTTCAATAGGACCAACGATATACCAACTGCTGTCGGCG
TGCGAGATCATAACATCGGACCACGGCGAGCAGAACCTGCGAGAATTAGGGAAGTGTTACGATTGGTGCCCAACC
ATGCAATACGTGTGTGAGCTTGAGGGAAACCGCAATCGGTGGCAGGAGAAGGAAGCGTGGCTTCAGAAGTCAGTC
AAACGCCTTCTCAAGTGTGACGTTCCGCCGGCTAACTGCGTGCTTACATTGCTGACTCTGGAAGGCGCTTGTCCG
AGCATCGATGCATATCGCGTCGCCATGCGCGGCTTAGTCAGTCTCTTGAAGCCAGGACGCCACTTGGCAACAATG
GCAGCGCTCCATGTTAAGCATTACTTAGTAGGACTGCAATTCTTCGGAATTTACCTCAAGAAGAAAATGGTGGAA
GCTGCCTTACAAGAAGCAGGATGCCAAATGTTGGGTGTGACAACTACGCCGTTAGCCGCACTTAAACTCGCTGCT
TTGATGAGAGCGTCAGCACTCCTCGAGCACCACCACCACCACCACTGA

MEGRLYAEGENYKKEFNPKDYQVTYYAFDSGATAENEILKFNLENLFQMFTAGGVGSNVLTDISIGPTIYQLLSA
CEIITSDHGEQNLRELGKCYDWCPTMQYVCELEGNRNRWQEKEAWLQKSVKRLLKCDVPPANCVLTLLTLEGACP
SIDAYRVAMRGLVSLLKPGRHLATMAALHVKHYLVGLQFFGIYLKKKMVEAALQEAGCQMLGVTTTPLAALKLAA
LMRASALLEHHHHHH*

Wild type 40 (G1PR98)
ATGTTCGAGCCTGGGGCCTACCTTCGGAACAACTACGCACCGCCGCGCGGAGACCTTCGTGCTGCAGACGGCGTT
GGCCCATGGAAACTCCGATGCTTAGCTCAAACCTTCGCCACGGGTGAGGTCTCCGGACACACACTTATCGACATA
GGAAGTGGGCCCACCGTCTACCAGCTCTTGTCGGCCTGTGCACACTTCGAGGACATAACTATGACTGACTTTCTG
GAAGTCAACCGGCAGGAACTGGGTCTGTGGCTCCGGGAAGAGCCTGGGGCCTTCGACTGGTCCGGCTACAGTCGC
CATGTATGTCTCATAGAAGACAAAGGCGAAAGTTGGCAGGAAAAGGAGCGACAACTCCGTGTCCGTGTAAAGCGC
GTCCTGCCGATAGATGTCCACCAGCCGCAGCCGCTGGGTGCTGGCTCATTAGCACCGCTCCCAGCAGACGCCCTG
GTTAGTACCTTCTGCCTTGAAGCCGTTTCGCCAGATCTTGCTAGCTTCCAGCGGGCACTTGATCACATCACGACT
TTACTTCGGCCTGGTGGACACCTGCTTTTAATAGGTGCGCTTGAGGAGTCTTGGTATTTGGCAGGCGAGGCCAGA
CTTGCTGTGGTCCCACTCTGTGAGGAAGAGGTTAGAGAAGCACTTGTTCGCAGTGGATACAAGGTTCGCGACCTC
AGAACCTATACAATGCCGGCTTATTTACGGACAGGGGTCGACGATGTTAAGGGCATCTTCTTTGCGTGGGCTCAG
AAGAAAGTGGGTGTTCTCGAGCACCACCACCACCACCACTGA

MFEPGAYLRNNYAPPRGDLRAADGVGPWKLRCLAQTFATGEVSGHTLIDIGSGPTVYQLLSACAHFEDITMTDFL
EVNRQELGLWLREEPGAFDWSGYSRHVCLIEDKGESWQEKERQLRVRVKRVLPIDVHQPQPLGAGSLAPLPADAL
VSTFCLEAVSPDLASFQRALDHITTLLRPGGHLLLIGALEESWYLAGEARLAVVPLCEEEVREALVRSGYKVRDL
RTYTMPAYLRTGVDDVKGIFFAWAQKKVGVLEHHHHHH*

Wild type 41 (K9T4R2)
ATGGTCGCGCCCCGATTCGAGATACAGTACGCGGCCTACCGAGATTGGCAACCCCAGGACTATTTAGCACAGTAC
TACGTCGACGTCAAGACAGAGGAGCTGTTAACTCTTGAATTCTTAGTTCAAAGCCTGCAAAACATGCCTACCACC
TCGGTAATGCTTGATTTTGGGTGTGGGCCCATAATATCTCACATTCTTCCCATCGTGCCGAAAGTGCAAGAGATT
CATATGGCTGAATACCTTCCGGCCAATCGGGCGGAAGTGCAGAAGTGGCTGGCTAGTACAGACGATGCGCACAAC
TGGCGCGCATTCGCTCTTGCGACCTTACGCCTTGAGGGGAACCCGAACCCGACAGAGACAGAGGCTAAGGCCCGT
GAACAACAAGCGCGCGATCGTATAAAGTCTTTATTACCGTGCGATGTTAACAACCCAGACCCCTTAGGTGCACAA
CGACGTGGCTTCTACCCGTTGGTGACTACTTCCTACTGCGCTGAAGGCGTTACCACCAGTAAAGAGAAGTGGCGG
GCCTATATGCGTAATATAGCTAGCTTGGTAAAACCTGGTGGGGTTCTGTTACTGAGCGCGGTTGGTGGGGCAGCA
AACTTTTATAGAGTAGGGGATCGATACTTCCCTTGTACCCGTCTGGACCGCCAGGATGTATTAGCGAGTCTCGGG
GAGAATGGATTCACCGACATTGATATCCGTATCCGCCAGGTCAGCGACCGCTCTCAAGAGGACTATTCGCACCTG
ATTTTCGCGCGCGCGGTCAAAGCGGGCCTCGAGCACCACCACCACCACCACTGA

MVAPRFEIQYAAYRDWQPQDYLAQYYVDVKTEELLTLEFLVQSLQNMPTTSVMLDFGCGPIISHILPIVPKVQEI
HMAEYLPANRAEVQKWLASTDDAHNWRAFALATLRLEGNPNPTETEAKAREQQARDRIKSLLPCDVNNPDPLGAQ
RRGFYPLVTTSYCAEGVTTSKEKWRAYMRNIASLVKPGGVLLLSAVGGAANFYRVGDRYFPCTRLDRQDVLASLG
ENGFTDIDIRIRQVSDRSQEDYSHLIFARAVKAGLEHHHHHH*

Wild type 42 (S4RYD2)
ATGGAAGGGCAAAATGTGACTAAACGCTATGGTCAAGACTATGCAACTCATTTTAAGGCGCGCGAGTACTTAGAC
ACCTACTATAACACAACTCCGGAAGACTCTCCTAAGCGAGACTTCTTCCCGGAATTATTGCAATTCCTTCACTCT
ACATTCAATAACGGGGAGATCCGGGGCCAGCGGTTGCTTGACGTTGGAAGTGGTCCCACCATTCATCAAGTACTG
TCCGCGAGTGAGTGGTTCCCGGAAATTTATTTATCAGACTACGCTCAATCAAACCGCGAGGAGCTGCAGAGTTGG
CTGTGCCACTCATCCGATGCTTTTGATTGGACTCGAACCATAGAATTCGTTTGCCGTCTTGAGGGCCATCGTAAG
ACTGTTGAGGAGAAGTCTGTCCACGTGCGTAAGGCAGTCAGAGCCGTGCTGCCTTGTGATGTTAACCAGGAAGAT
CCATTGCTTGGTTCTTGGTCCCAGGGCCCTTTCGATTGCATAATTTCTACGTTATGTTTCGAGGCCGCATGCCGG
TCTATTGGAGACTTCACAGCGGCGCTTGGACATGTATGCCATCTGCTTAGACCGGGTGGCTGGTTTATCCTTGCC
ACAGAGTTAGGGGAGACCTTCTACCATATCGGTTCAGAAACGTTCCACGTTTTATCCTTGAATGAGACCGTTATT
CGTCAGGTTGTTGGGGACGCCGGACTGATTATCCAAGAGCTGGAGATGCACTTAGCCCCTAAACCGAGCCCAGAT
GACACGAACGACCACCAGGCCCTGTTGTTCCTGCGCGCCCAGAAGAAGATTCGTCCAGTTGTTGTTTATCTCGAG
CACCACCACCACCACCACTGA

MEGQNVTKRYGQDYATHFKAREYLDTYYNTTPEDSPKRDFFPELLQFLHSTFNNGEIRGQRLLDVGSGPTIHQVL
SASEWFPEIYLSDYAQSNREELQSWLCHSSDAFDWTRTIEFVCRLEGHRKTVEEKSVHVRKAVRAVLPCDVNQED
PLLGSWSQGPFDCIISTLCFEAACRSIGDFTAALGHVCHLLRPGGWFILATELGETFYHIGSETFHVLSLNETVI
RQVVGDAGLIIQELEMHLAPKPSPDDTNDHQALLFLRAQKKIRPVVVYLEHHHHHH*

Wild type 43 (H9G6B1)
ATGGTCTCGTCCTTTCTGCTCAAGCTTGATCACAGTAACAATTACCACGTGCGCCGCCACCGCGAGGCGATCGAG
ATCCACAAGCATGTTGACAACTTTAACCGCAAGGAAGAAACCACTAAGATGAATAAAATTTGGCTGAGTGTTCTC
AAGAATTCTAAGATTAAGATCGTTGACGGGAACCAGCACAGCGAGGGTCGTGCACAACTGATGACCAACAAAGGT
TACGGAATTCGTGGCGACACGCTCATCGACATTGGCAGTGGACCCTCAATCTATCAGCTTTTATCCGCCTGTGAG
AGCTTTCGCGAGATTATTGTTACGGATTTCTTAGAGCAAAACCGGGTTGAGATTCGGAAATGGTTGGAAAAGGAC
CCGGAAGCGTTTGATTGGACCTCAATGGTGAAGTACACCTGTCAGATGGAAGGTGACCGAGAAAAGTGGATGGAG
AAAGAAGAGAAACTGCGGCGCACAATCAAACAAGTATTACCTTGTGACGTTACGTTGGCTAACCCGCTCGATCCT
TTGGTGTTACAACCCGTTGATTGCGTCTTCTCCAGCTACTGCCTGGAGTCAGCCTGCAACGACGTTCCCACCTAC
CGCTCGGCCGTGAAGAACGTTGGTTCATTAGTGAAGCCCGGTGGGCATCTTATATTCGCGGTCATACTCGAGGAG
ACTTACTACATGGTAGGCCCGCACAAGTTCAACTGTCTTTACTTGACTCCGGAGATTGTAACCGACGCCGTGAAA
GGCGCAGGGTTCGAGGTACTTTGGTCACAAGCACTGGGGTTTACGTTTCCGTTAGCGATTTGTGATGCCAAAGAC
ACGTTCTTCATCGTGGCCAAGAAACCTCGCACGCCCCTCGAGCACCACCACCACCACCACTGA

MVSSFLLKLDHSNNYHVRRHREAIEIHKHVDNFNRKEETTKMNKIWLSVLKNSKIKIVDGNQHSEGRAQLMTNKG
YGIRGDTLIDIGSGPSIYQLLSACESFREIIVTDFLEQNRVEIRKWLEKDPEAFDWTSMVKYTCQMEGDREKWME
KEEKLRRTIKQVLPCDVTLANPLDPLVLQPVDCVFSSYCLESACNDVPTYRSAVKNVGSLVKPGGHLIFAVILEE
TYYMVGPHKFNCLYLTPEIVTDAVKGAGFEVLWSQALGFTFPLAICDAKDTFFIVAKKPRTPLEHHHHHH*

Wild type 44 (L5KJT9)
ATGCAAATCTCGAGCATATGTCACCCGATGATGCCCGATTCTTATTTTCCGGTGCCGTATAGTAAGCCAGATGGT
GTAAACGGTGACCTTCTGATAGACATCGGATCGGGCCCAACCATTTATCAGCTGTTGTCAGCATGCGAGTCTTTT
AAGGAAATAATAGCTACTGACTATATCGACCAAAATTTGCAGGAGTTAGAAAAGTGGCTTAAGAAGGAACCCGGG
GCGTTTTCCTGGAGCTTGGTAATGACCTACGTTTGTGAATTAGAAGGAAACCGGGCAAAGGGCCCTGAGAAGGAA
GAGAAACTGAGACGAGCAATCAAGCAGGTACTCAAGTGCGACGTGACCCAGTCGAGACCGCTCGGCGCGGTGTCC
CTTCCACTTGCAGATTGCCTTTTATCAACACTGTGTTTGGACGCCGCATGTCCCGACTTACCCGCGTACCATTCG
GCACTTCGTAACCTTAGTTCGCTGCTCAAGCCAGGCGGCTACCTGGTGATCATAGACGCGCTGGAATCTTCGTAC
TATATGATAGGCGAGCAACGATTTTCAAGTCTTTGCTTACGTCAAGAGGAAGTGAAGGCCGCCGTACGCGAAGCC
GGTTACACTATCAAGCAATTTGAGGTAATTCCACAATCGTACTCATCCACACGCGCCAATAACAAGGGTCTGTTC
TTCTTAGTTGGGCAGAAACTGCGCAGTTCCGAACTCGAGCACCACCACCACCACCACTGA

MQISSICHPMMPDSYFPVPYSKPDGVNGDLLIDIGSGPTIYQLLSACESFKEIIATDYIDQNLQELEKWLKKEPG
AFSWSLVMTYVCELEGNRAKGPEKEEKLRRAIKQVLKCDVTQSRPLGAVSLPLADCLLSTLCLDAACPDLPAYHS
ALRNLSSLLKPGGYLVIIDALESSYYMIGEQRFSSLCLRQEEVKAAVREAGYTIKQFEVIPQSYSSTRANNKGLF
FLVGQKLRSSELEHHHHHH*

Wild type 45 (D2I2G0)
ATGTCTGCTGCAGATCCTTCCCCGGCTGCGGGTGCAGCGCCGAATAGCGACCAGGGGCGTGCCGCTGTTGCTTCA
GCGTATCAGCGTTTCGAACCGAGAGCGTATTTACGGAACAACTACGCCCCGCCTCGCGGAGACCTGTCATCACCG
GATGGCGTGGGCCCATGGAAGCTTCGGTGTCTGGCACAGACCTTTGCAACGGGCGAGGTAAGTGGACACAGTCTG
ATCGACATTGGGAGCGGACCCACAATATATCAGTTATTAAGCGCCTGCAGCCATTTCGAGGATATAACCATGACA
GACTTCTTAGAGGTAAATCGGCAGGAGCTGGGCTTATGGCTTCGGGAAGAACCGGGCGCTTTCGACTGGAGCGGC
TATTCTCAACACGTGTGTCTCATCGAGGGTAAGGGAGAATCCTGGCGGGAAAAGGAGCGGCAACTGCGCGCCAGA
GTCAAGCGCGTTCTTCACGTAGATGTTCACCAGCCTCAACCTCTTGGGGCAGGTAGCTTGGCCCCGCTCCCCGCT
GACGCCTTGGTCAGTGCGTTCTGCTTAGAGGCTGTGTCTCCGGATCTCCCCTCTTTTCAACGGGCACTTGATCAC
ATCACCACGCTGTTGAGACCCGGCGGGCATCTTCTGCTGATAGGCGCTCTGGAAGAATCATGGTATCTTGCGGGA
GAAGCACGCCTTGTGGTAGTGCCCGTATGCAAGGAAGAGGTCATGGAAGCCTTAGTTCGTAGTGGATACGAAGTC
CGCGATCTCCGTACCTACGTCATGCCAGCCTGTCTGCAAACTGGGGTTGACGACGTAAAAGGCGTTTTCTTTGCC
TGGGCCCAGAAGCTCGAGCACCACCACCACCACCACTGA

MSAADPSPAAGAAPNSDQGRAAVASAYQRFEPRAYLRNNYAPPRGDLSSPDGVGPWKLRCLAQTFATGEVSGHSL
IDIGSGPTIYQLLSACSHFEDITMTDFLEVNRQELGLWLREEPGAFDWSGYSQHVCLIEGKGESWREKERQLRAR
VKRVLHVDVHQPQPLGAGSLAPLPADALVSAFCLEAVSPDLPSFQRALDHITTLLRPGGHLLLIGALEESWYLAG
EARLVVVPVCKEEVMEALVRSGYEVRDLRTYVMPACLQTGVDDVKGVFFAWAQKLEHHHHHH*

Wild type 46 (F6ZPQ7)
ATGTCAGGGGCTGACCCGGACAGTGCACCCGGCCGGGCGGCCGTTGCGTCGGCCTATCAACGATTTGAACCACGG
GCTTACCTGCGAAATAATTATGCCCCGCCGCGTGGGGATCTTTCCTCGCCCGACGGCGTAGGCCCATGGAAGCTG
AGATGCTTAGCGCAAACGTTCGCAACAGGGGAAGTTAGCGGACGCACGTTGATTGACATTGGTAGCGGTCCAACC
GTATACCAGTTACTTAGCGCTTGTTCTCACTTCGAGGACATCACAATGACTGATTTCTTAGAAGTCAACCGTCAA
GAACTGGGGCGCTGGTTACGTGAAGAGCCAGGGGCTTTCAACTGGTCTGTATACTCGCAACATGCGTGCTTAATC
GAAGGGAAGGGCGAAAGTTGGCAAGAGAAAGAGCGGCAGCTGCGCGCCCGCGTTAAGCGAGTACTGCCGATAGAC
GTTCATCAATTACAGCCGTTGGGAGCAGGCTCCCCGGCTCCTCTCCCCGCCGATGCTTTAGTCAGTGCATTCTGT
TTAGAGGCAGTATCACCCGATCTGGCGTCTTTCCAACGTGCACTTGACCACATCACAACTCTGCTCCGGCCGGGT
GGACACCTTCTCCTGATTGGAGCTTTAGAAGAATCGTGGTATTTAGCTGGCGAGGCACGCCTCACCGTCGTGCCG
GTCTCTGAGGAAGAGGTTCGCGAGGCGCTTGTTCGCAGTGGATACGAGGTGCGTGACCTTCGTACCTACTCGATG
CCGGCGCGGCTTCAAACCGGCGTTGACGACGTGAAAGGCATTTTCTTCGCCTGGGCTCAGAAGAAGGTGGGACTG
CTCGAGCACCACCACCACCACCACTGA

MSGADPDSAPGRAAVASAYQRFEPRAYLRNNYAPPRGDLSSPDGVGPWKLRCLAQTFATGEVSGRTLIDIGSGPT
VYQLLSACSHFEDITMTDFLEVNRQELGRWLREEPGAFNWSVYSQHACLIEGKGESWQEKERQLRARVKRVLPID
VHQLQPLGAGSPAPLPADALVSAFCLEAVSPDLASFQRALDHITTLLRPGGHLLLIGALEESWYLAGEARLTVVP
VSEEEVREALVRSGYEVRDLRTYSMPARLQTGVDDVKGIFFAWAQKKVGLLEHHHHHH*

Wild type 47 (P50135)
ATGGCGAGCTCTATGCGCAGCCTGTTTTCGGATCATGGGAAGTATGTAGAAAGCTTCCGCCGCTTCCTGAACCAC
AGTACCGAGCATCAATGTATGCAGGAATTCATGGACAAAAAGCTTCCGGGAATTATCGGCCGCATCGGTGATACT
AAGTCGGAAATCAAAATTCTTTCTATCGGGGGCGGCGCTGGGGAGATTGACCTTCAGATCCTGTCTAAGGTACAG
GCACAATATCCTGGAGTATGTATCAATAATGAAGTGGTCGAGCCTAGTGCGGAGCAAATCGCGAAGTATAAAGAA
TTAGTAGCGAAAACTAGCAATCTGGAGAATGTCAAATTTGCGTGGCATAAAGAAACGTCGTCTGAATATCAATCC
CGCATGCTTGAAAAAAAGGAGTTACAGAAATGGGACTTCATCCACATGATTCAAATGCTTTATTACGTCAAGGAC
ATCCCTGCTACACTGAAATTCTTTCATTCTCTGTTGGGGACTAATGCTAAGATGCTTATCATTGTTGTTTCTGGT
AGTTCAGGCTGGGACAAACTGTGGAAGAAATATGGAAGTCGCTTTCCTCAGGATGATTTATGCCAGTATATCACA
TCGGACGACCTTACCCAGATGCTGGATAATTTGGGATTAAAGTATGAGTGCTATGACTTGCTGTCAACCATGGAC
ATTTCGGATTGCTTTATTGATGGCAATGAGAATGGTGATTTATTGTGGGACTTTTTAACGGAGACCTGTAATTTT
AATGCAACTGCCCCACCCGATTTACGCGCGGAATTGGGAAAAGATTTGCAGGAGCCGGAATTCTCCGCGAAGAAA
GAGGGCAAGGTTCTTTTTAATAACACATTATCTTTTATCGTGATCGAAGCTCTCGAGCACCACCACCACCACCAC
TGA

MASSMRSLFSDHGKYVESFRRFLNHSTEHQCMQEFMDKKLPGIIGRIGDTKSEIKILSIGGGAGEIDLQILSKVQ
AQYPGVCINNEVVEPSAEQIAKYKELVAKTSNLENVKFAWHKETSSEYQSRMLEKKELQKWDFIHMIQMLYYVKD
IPATLKFFHSLLGTNAKMLIIVVSGSSGWDKLWKKYGSRFPQDDLCQYITSDDLTQMLDNLGLKYECYDLLSTMD
ISDCFIDGNENGDLLWDFLTETCNFNATAPPDLRAELGKDLQEPEFSAKKEGKVLFNNTLSFIVIEALEHHHHHH
*

Wild type 48 (O97972)
ATGGAAGGGGGCTTCACGGGGGGTGATGAATACCAAAAACACTTTCTGCCTCGCGACTATCTTAATACATACTAT
AGTTTTCAGAGCGGTCCGTCTCCCGAGGCCGAAATGCTGAAATTCAACCTTGAATGCCTTCACAAAACCTTTGGG
CCGGGAGGCTTACAAGGCGATACATTGATTGATATCGGTTCCGGCCCGACAATCTACCAAGTCTTAGCCGCGTGT
GAATCTTTCAAAGACATTACCCTTTCGGATTTCACTGACCGTAACCGCGAGGAGCTTGCCAAGTGGTTAAAAAAG
GAGCCTGGAGCCTATGACTGGACCCCTGCATTGAAGTTCGCTTGTGAACTTGAAGGAAATTCAGGCCGTTGGCAA
GAAAAGGCCGAGAAACTTCGCGCCACCGTGAAACGTGTTCTGAAATGCGATGCGAACTTGAGTAATCCACTTACG
CCCGTGGTCTTGCCTCCAGCAGATTGTGTGCTGACATTGCTGGCAATGGAATGTGCATGTTGTTCACTGGATGCC
TACCGCGCAGCTCTTCGTAACTTAGCGAGTTTGTTGAAGCCAGGAGGACATTTGGTAACGACGGTAACCTTGCAA
TTATCTAGCTATATGGTTGGGGAGCGCGAATTTTCCTGCGTAGCCCTGGAAAAAGAGGAAGTTGAACAGGCGGTG
CTGGACGCGGGGTTCGACATCGAACAGTTGCTTTACTCGCCACAGTCTTATAGTGCGAGCACTGCGCCAAACCGC
GGCGTCTGTTTCCTGGTGGCCCGCAAGAAGCCTGGATCTCTCGAGCACCACCACCACCACCACTGA

MEGGFTGGDEYQKHFLPRDYLNTYYSFQSGPSPEAEMLKFNLECLHKTFGPGGLQGDTLIDIGSGPTIYQVLAAC
ESFKDITLSDFTDRNREELAKWLKKEPGAYDWTPALKFACELEGNSGRWQEKAEKLRATVKRVLKCDANLSNPLT
PVVLPPADCVLTLLAMECACCSLDAYRAALRNLASLLKPGGHLVTTVTLQLSSYMVGEREFSCVALEKEEVEQAV
LDAGFDIEQLLYSPQSYSASTAPNRGVCFLVARKKPGSLEHHHHHH*

Wild type 49 (P40261)
ATGGAGAGTGGATTTACGAGTAAGGACACTTATTTGAGTCATTTTAACCCGCGCGACTACTTAGAAAAATACTAC
AAGTTTGGAAGCCGCCATTCAGCAGAATCACAAATTCTGAAACACCTTTTAAAGAACCTGTTTAAAATCTTCTGT
TTAGATGGGGTCAAAGGAGATTTGTTGATTGACATCGGATCAGGTCCCACAATTTACCAACTTCTGTCCGCTTGT
GAGAGTTTTAAGGAGATCGTCGTGACGGACTATTCAGATCAAAATCTGCAAGAACTGGAGAAATGGTTAAAGAAA
GAACCAGAGGCGTTCGATTGGAGCCCAGTTGTGACCTATGTCTGTGATCTTGAAGGAAACCGCGTAAAGGGTCCT
GAAAAGGAGGAAAAGCTGCGCCAAGCCGTAAAGCAAGTATTAAAATGTGATGTTACTCAAAGCCAACCATTAGGA
GCCGTCCCACTGCCCCCGGCGGATTGTGTATTGTCAACCCTTTGCTTGGACGCAGCTTGTCCTGACTTGCCTACA
TATTGTCGTGCATTACGCAACTTAGGAAGTTTGCTTAAACCAGGAGGGTTTCTGGTCATCATGGATGCCTTGAAG
TCATCCTACTACATGATTGGGGAACAGAAGTTCTCATCTTTACCTTTGGGCCGTGAAGCCGTAGAAGCGGCCGTA
AAGGAGGCGGGGTACACCATTGAATGGTTCGAGGTTATTTCACAGTCCTACAGCTCCACAATGGCAAATAACGAG
GGCTTATTTTCGCTGGTTGCTCGCAAACTGAGTCGTCCTCTTCTCGAGCACCACCACCACCACCACTGA

MESGFTSKDTYLSHFNPRDYLEKYYKFGSRHSAESQILKHLLKNLFKIFCLDGVKGDLLIDIGSGPTIYQLLSAC
ESFKEIVVTDYSDQNLQELEKWLKKEPEAFDWSPVVTYVCDLEGNRVKGPEKEEKLRQAVKQVLKCDVTQSQPLG
AVPLPPADCVLSTLCLDAACPDLPTYCRALRNLGSLLKPGGFLVIMDALKSSYYMIGEQKFSSLPLGREAVEAAV
KEAGYTIEWFEVISQSYSSTMANNEGLFSLVARKLSRPLLEHHHHHH*

Wild type 50 (P11086)
ATGTCTGGCGCGGATCGTAGTCCTAATGCAGGAGCAGCCCCCGACTCAGCACCCGGTCAGGCGGCTGTCGCCAGC
GCATACCAGCGTTTTGAGCCACGCGCGTATTTGCGTAACAACTATGCACCCCCCCGTGGCGATTTATGTAACCCC
AATGGTGTGGGTCCGTGGAAACTTCGCTGTCTGGCGCAAACTTTCGCTACCGGCGAGGTGTCTGGACGCACGTTG
ATTGACATCGGCAGTGGCCCTACAGTCTACCAGTTGCTGAGCGCGTGCTCCCACTTCGAAGACATTACGATGACG
GATTTTCTTGAAGTGAACCGCCAAGAGCTTGGCCGCTGGTTGCAGGAGGAACCCGGCGCCTTTAACTGGTCAATG
TACAGTCAACATGCTTGCTTGATCGAGGGCAAGGGTGAGTGTTGGCAGGACAAAGAACGCCAACTGCGCGCCCGC
GTCAAACGCGTACTGCCTATTGATGTCCATCAACCTCAGCCCTTGGGTGCTGGGTCACCTGCCCCTTTACCAGCT
GATGCATTGGTGTCTGCCTTTTGCCTGGAAGCGGTGAGTCCAGACCTTGCTAGCTTTCAACGTGCATTAGACCAC
ATCACGACCCTGCTTCGCCCAGGAGGGCACTTGTTACTGATCGGGGCATTAGAAGAGTCATGGTATCTGGCCGGA
GAAGCCCGCCTTACCGTCGTTCCCGTTTCTGAAGAGGAAGTTCGCGAAGCATTGGTACGCTCAGGATATAAAGTG
CGTGATCTTCGCACTTACATTATGCCTGCCCACTTACAAACAGGCGTGGACGACGTGAAGGGAGTTTTCTTTGCT
TGGGCACAGAAAGTGGGATTACTCGAGCACCACCACCACCACCACTGA

MSGADRSPNAGAAPDSAPGQAAVASAYQRFEPRAYLRNNYAPPRGDLCNPNGVGPWKLRCLAQTFATGEVSGRTL
IDIGSGPTVYQLLSACSHFEDITMTDFLEVNRQELGRWLQEEPGAFNWSMYSQHACLIEGKGECWQDKERQLRAR
VKRVLPIDVHQPQPLGAGSPAPLPADALVSAFCLEAVSPDLASFQRALDHITTLLRPGGHLLLIGALEESWYLAG
EARLTVVPVSEEEVREALVRSGYKVRDLRTYIMPAHLQTGVDDVKGVFFAWAQKVGLLEHHHHHH*

dre-H-NMT_C198Y
ATGGCAGCACCTTTCAAGACTTTGGTTGAGGATTATCCACGTTATCTTAAGTCGTTCGAGCTTTTCTTAGAGCGT
TCGTCTGAACACCAATGCATGCAAGATTTCATCCACAACACATTGCCTGACATACTTGCGTCTATAGGCGGCGGT
CGTAGCGTATTCAATGTTATGGGAGTAGGTTCAGGTGCAGGGGACATCGACCTTGAGATGCTGGCACAGTTACAC
TTGAAGCACCCTCATGTAAAGGTAGATAATGAGGTGGTGGAGCCCTCAAACGACATGCTGTACAAATACAAGGCG
CGCGTTAGTACATCCCCGGACCTTGCATACATTAACTTCACCTGGAATAAGATGACAGCATCGGAATTTGAGAAA
CAATGGCAAGAGAAAACCCCCGAGAAGAAGATGGACTTCATCCATATGATACAAATGTTGTATTACGTGAAGGAT
CCTAACGCTACGGTCTCATTCTTCCGTAGCTTACTGGAGAAAGACGGGAAACTTCTCATCATACTGGTCAGTGGT
GAAAGCGGGTGGGGTAAGCTGTGGACTACATTTCGTAAGCAACTGTGTTATACAGAGATGTCACAATATGTGACC
ATAGGAGAGATCAAATCATTCTTAGATTCCGAGGGTGTCCCGTATCGGAAGTACGTTTTACTTAGCCAAATGGAT
ATTACTGAATGCTTCACCGAAGGTGACCAAGAAGGTGAGCTGCTGCTTGACTTCTTAACAGAGGTTAAGGAATTC
TCAAAGAACGCACCCGAGCGCCTTAAGAAGGAAGTTCTGGACGTGCTGCGCCACCCGGACTGTAGTAAGGAAGTG
GACGGCCGGATCATCTTTAACAACAATTTAGAGGTCTTGGTAATTGAACCGCTCGAGCACCACCACCACCACCAC
TGA

MAAPFKTLVEDYPRYLKSFELFLERSSEHQCMQDFIHNTLPDILASIGGGRSVFNVMGVGSGAGDIDLEMLAQLH
LKHPHVKVDNEVVEPSNDMLYKYKARVSTSPDLAYINFTWNKMTASEFEKQWQEKTPEKKMDFIHMIQMLYYVKD
PNATVSFFRSLLEKDGKLLIIILVSGESGWGKLWTTFRKQLCYTEMSQYVTIGEIKSFLDSEGVPYRKYVLLSQMD
ITECFTEGDQEGELLLDFLTEVKEFSKNAPERLKKEVLDVLRHPDCSKEVDGRIIFNNNLEVLVIEPLEHHHHHH
*

dre-H-NMT_C198Y/Y15A
ATGGCAGCACCTTTCAAGACTTTGGTTGAGGATTATCCACGTGCGCTTAAGTCGTTCGAGCTTTTCTTAGAGCGT
TCGTCTGAACACCAATGCATGCAAGATTTCATCCACAACACATTGCCTGACATACTTGCGTCTATAGGCGGCGGT
CGTAGCGTATTCAATGTTATGGGAGTAGGTTCAGGTGCAGGGGACATCGACCTTGAGATGCTGGCACAGTTACAC
TTGAAGCACCCTCATGTAAAGGTAGATAATGAGGTGGTGGAGCCCTCAAACGACATGCTGTACAAATACAAGGCG
CGCGTTAGTACATCCCCGGACCTTGCATACATTAACTTCACCTGGAATAAGATGACAGCATCGGAATTTGAGAAA
CAATGGCAAGAGAAAACCCCCGAGAAGAAGATGGACTTCATCCATATGATACAAATGTTGTATTACGTGAAGGAT
CCTAACGCTACGGTCTCATTCTTCCGTAGCTTACTGGAGAAAGACGGGAAACTTCTCATCATACTGGTCAGTGGT
GAAAGCGGGTGGGGTAAGCTGTGGACTACATTTCGTAAGCAACTGTGTTATACAGAGATGTCACAATATGTGACC
ATAGGAGAGATCAAATCATTCTTAGATTCCGAGGGTGTCCCGTATCGGAAGTACGTTTTACTTAGCCAAATGGAT
ATTACTGAATGCTTCACCGAAGGTGACCAAGAAGGTGAGCTGCTGCTTGACTTCTTAACAGAGGTTAAGGAATTC
TCAAAGAACGCACCCGAGCGCCTTAAGAAGGAAGTTCTGGACGTGCTGCGCCACCCGGACTGTAGTAAGGAAGTG
GACGGCCGGATCATCTTTAACAACAATTTAGAGGTCTTGGTAATTGAACCGCTCGAGCACCACCACCACCACCAC
TGA

MAAPFKTLVEDYPRALKSFELFLERSSEHQCMQDFIHNTLPDILASIGGGRSVFNVMGVGSGAGDIDLEMLAQLH
LKHPHVKVDNEVVEPSNDMLYKYKARVSTSPDLAYINFTWNKMTASEFEKQWQEKTPEKKMDFIHMIQMLYYVKD
PNATVSFFRSLLEKDGKLLIIILVSGESGWGKLWTTFRKQLCYTEMSQYVTIGEIKSFLDSEGVPYRKYVLLSQMD
ITECFTEGDQEGELLLDFLTEVKEFSKNAPERLKKEVLDVLRHPDCSKEVDGRIIFNNNLEVLVIEPLEHHHHHH
*

# IX. References


[1] L. L. Bengel, B. Aberle, A. Egler-Kemmerer, S. Kienzle, B. Hauer, S. C. Hammer, *Angew. Chem. Int. Ed.* **2021**, *60*, 5554–5560.
[2] K. H. Schülke, F. Ospina, K. Hörnschemeyer, S. Gergel, S. C. Hammer, *ChemBioChem* **2022**, *23*, e202100632.
[3] K. H. Schülke, J. S. Fröse, A. Klein, M. Garcia-Borràs, S. C. Hammer, *ChemBioChem* **2024**, *25, e202400079*.
[4] T. Baba, T. Ara, M. Hasegawa, Y. Takai, Y. Okumura, M. Baba, K. A. Datsenko, M. Tomita, B. L. Wanner, H. Mori, *Mol. Syst. Biol.* **2006**, *2*.
[5] A. Hoffmann, K. H. Schülke, S. C. Hammer, A. Rentmeister, N. V. Cornelissen, *Chem. Commun.* **2023**, *59*, 5463–5466.
[6] D. G. Gibson, L. Young, R.-Y. Chuang, J. C. Venter, C. A. Hutchison, H. O. Smith, *Nat. Methods* **2009**, *6*, 343–345.
[7] S. Kille, C. G. Acevedo-Rocha, L. P. Parra, Z. G. Zhang, D. J. Opperman, M. T. Reetz, J. P. Acevedo, *ACS Synth. Biol.* **2013**, *2*, 83–92.
[8] J. Abramson, J. Adler, J. Dunger, R. Evans, T. Green, A. Pritzel, O. Ronneberger, L. Willmore, A. J. Ballard, J. Bambrick, S. W. Bodenstein, D. A. Evans, C.-C. Hung, M. O'Neill, D. Reiman, K. Tunyasuvunakool, Z. Wu, A. Žemgulytė, E. Arvaniti, C. Beattie, O. Bertolli, A. Bridgland, A. Cherepanov, M. Congreve, A. I. Cowen-Rivers, A. Cowie, M. Figurnov, F. B. Fuchs, H. Gladman, R. Jain, Y. A. Khan, C. M. R. Low, K. Perlin, A. Potapenko, P. Savy, S. Singh, A. Stecula, A. Thillaisundaram, C. Tong, S. Yakneen, E. D. Zhong, M. Zielinski, A. Žídek, V. Bapst, P. Kohli, M. Jaderberg, D. Hassabis, J. M. Jumper, *Nature* **2024**, *630*, 493–500.
[9] J. R. Horton, K. Sawada, M. Nishibori, X. Zhang, X. Cheng, *Structure* **2001**, *9*, 837–849.
[10] N. J. Liverton, J. W. Butcher, C. F. Claiborne, D. A. Claremon, B. E. Libby, K. T. Nguyen, S. M. Pitzenberger, H. G. Selnick, G. R. Smith, A. Tebben, J. P. Vacca, S. L. Varga, L. Agarwal, K. Dancheck, A. J. Forsyth, D. S. Fletcher, B. Frantz, W. A. Hanlon, C. F. Harper, S. J. Hofsess, M. Kostura, J. Lin, S. Luell, E. A. O'Neill, C. J. Orevillo, M. Pang, J. Parsons, A. Rolando, Y. Sahly, D. M. Visco, S. J. O'Keefe, *J. Med. Chem.* **1999**, *42*, 2180–2190.
[11] W. F. De Azevedo, S. Leclerc, L. Meijer, L. Havlicek, M. Strnad, S. Kim, *Eur. J. Biochem.* **1997**, *243*, 518–526.
[12] K. Paul, A. Sharma, V. Luxami, *Bioorganic Med. Chem. Lett.* **2014**, *24*, 624–629.
[13] S. Chen, R. F. Graceffa, A. A. Boezio, *Org. Lett.* **2016**, *18*, 16–19.
[14] D. Xu, L. Frank, T. Nguyen, A. Stumpf, D. Russell, R. Angelaud, F. Gosselin, *Synlett* **2020**, *31*, 595–599.
[15] S.-J. Chen, D. L. Golden, S. W. Krska, S. S. Stahl, *J. Am. Chem. Soc.* **2021**, *143*, 14438–14444.
[16] H. J. A. Dale, G. R. Hodges, G. C. Lloyd-Jones, *J. Am. Chem. Soc.* **2019**, *141*, 7181–7193.
[17] S. P. Desai, M. T. Zambri, M. S. Taylor, *J. Org. Chem.* **2022**, *87*, 5385–5394.
[18] K. Xu, N. Thieme, B. Breit, *Angew. Chem. Int. Ed.* **2014**, *53*, 7268–7271.
[19] S. V. Sieger, I. Lubins, B. Breit, *Catalysts* **2022**, *12*, 1209.
[20] L. J. Hilpert, S. V. Sieger, A. M. Haydl, B. Breit, *Angew. Chem. Int. Ed.* **2019**, *58*, 3378–3381.
[21] A. M. Haydl, K. Xu, B. Breit, *Angew. Chem. Int. Ed.* **2015**, *54*, 7149–7153.
[22] K. Yahata, Y. Kaneko, S. Akai, *Org. Lett.* **2020**, *22*, 598–603.
[23] K. Wang, P. Chen, D. Ji, X. Zhang, G. Xu, J. Sun, *Angew. Chem. Int. Ed.* **2018**, *57*, 12489–12493.
[24] W. Jiang, D. Ji, W. Zhang, G. Zhang, X. Min, Y. Hu, X. Jiang, Q. Chen, *Angew. Chem. Int. Ed.* **2021**, *60*, 8321–8328.
[25] L. M. Stanley, J. F. Hartwig, *J. Am. Chem. Soc.* **2009**, *131*, 8971–8983.
[26] Y. Dong, B. Breit, *Org. Lett.* **2021**, *23*, 6765–6769.
[27] M. A. Fitzgerald, O. Soltani, C. Wei, D. Skliar, B. Zheng, J. Li, J. Albrecht, M. Schmidt, M. Mahoney, R. J. Fox, K. Tran, K. Zhu, M. D. Eastgate, *J. Org. Chem.* **2015**, *80*, 6001–6011.
[28] Q. Tang, C. W. Grathwol, A. S. Aslan-Üzel, S. Wu, A. Link, I. V. Pavlidis, C. P. S. Badenhorst, U. T. Bornscheuer, *Angew. Chem. Int. Ed.* **2021**, *60*, 1524–1527.
[29] N. Yu, H. Zhao, W. Wang, M. Dong, *ACS Catal.* **2024**, *14*, 6211–6216.
[30] G.-Y. Yang, G.-W. Zheng, B.-B. Zeng, J.-H. Xu, Q. Chen, *Mol. Catal.* **2023**, *550*, 113533.
[31] J. Peng, C. Liao, C. Bauer, F. P. Seebeck, *Angew. Chem. Int. Ed.* **2021**, *60*, 27178–27183.
[32] F. Ospina, K. H. Schülke, J. Soler, A. Klein, B. Prosenc, M. Garcia-Borràs, S. C. Hammer, *Angew. Chem. Int. Ed.* **2022**, *61*, e202213056.
[33] R. C. Nishad, A. Rit, *Chem. Eur. J.* **2021**, *27*, 594–599.
[34] R. C. Nishad, S. Kumar, A. Rit, *Organometallics* **2021**, *40*, 915–926.
[35] D. L. Flynn, P. A. Petillo, M. D. Kaufman (Deciphera Pharmaceuticals, LLC), WO2013036232, **2013**.
[36] Z. Yang, X. Zhang (Dizal (Jiangsu) Pharmaceutical Co.), US20220411422, **2022**.
[37] S. Li, D. Ma, *J. Org. Chem.* **2024**, *89*, 6626–6630.
[38] Z. Shao, S. Yuan, Y. Li, Q. Liu, *Chin. J. Chem.* **2022**, *40*, 1137–1143.
[39] E. Deau, M. F. Lindberg, F. Miege, D. Roche, N. George, P. George, A. Krämer, S. Knapp, L. Meijer, *J. Med. Chem.* **2023**, *66*, 10694–10714.
[40] J. E. Gillespie, C. Morrill, R. J. Phipps, *J. Am. Chem. Soc.* **2021**, *143*, 9355–9360.
[41] N. Xi, M. Li, J. Peng, X. Li, T. Zhang, H. Hu, W. Chen, C. Bai, D. Ke, P. Chen (Sunshine Lake Pharma Co., Ltd.), WO2019099311, **2019**.
